# Split drive killer-rescue provides a novel threshold-dependent gene drive

**DOI:** 10.1038/s41598-020-77544-7

**Published:** 2020-11-25

**Authors:** Matthew P. Edgington, Tim Harvey-Samuel, Luke Alphey

**Affiliations:** The Pirbright Institute, Ash Road, Woking, Surrey, Pirbright, GU24 0NF UK

**Keywords:** Genetics, Applied mathematics, Synthetic biology

## Abstract

A wide range of gene drive mechanisms have been proposed that are predicted to increase in frequency within a population even when they are deleterious to individuals carrying them. This also allows associated desirable genetic material (“cargo genes”) to increase in frequency. Gene drives have garnered much attention for their potential use against a range of globally important problems including vector borne disease, crop pests and invasive species. Here we propose a novel gene drive mechanism that could be engineered using a combination of toxin-antidote and CRISPR components, each of which are already being developed for other purposes. Population genetics mathematical models are developed here to demonstrate the threshold-dependent nature of the proposed system and its robustness to imperfect homing, incomplete penetrance of toxins and transgene fitness costs, each of which are of practical significance given that real-world components inevitably have such imperfections. We show that although end-joining repair mechanisms may cause the system to break down, under certain conditions, it should persist over time scales relevant for genetic control programs. The potential of such a system to provide localised population suppression via sex ratio distortion or female-specific lethality is also explored. Additionally, we investigate the effect on introduction thresholds of adding an extra CRISPR base element, showing that this may either increase or decrease dependent on parameter context.

## Introduction

A range of different gene drive classes have been proposed that are predicted to be capable of spreading desirable genetic traits through a population even if they confer a fitness cost upon individuals carrying them^1–3^. A wide variety of such desirable genetic traits have been discussed, one of the more high-profile being a reduced ability for mosquitoes to transmit pathogens (see for example^4–8^). Research groups worldwide are developing the components necessary to construct such systems, the majority of which rely on clustered regularly interspaced short palindromic repeats (CRISPR) technology (e.g. “global” CRISPR drives^9^, split drives^9^ and daisy drives^10^) or toxin-antidote-type systems (e.g. engineered underdominance^11^ or killer-rescue^12^). Examples of both CRISPR-based^13–16^ and toxin-antidote based^17,18^ systems have been successfully engineered and tested in laboratory cage studies but at present remain some way from testing in the field. Here we propose a novel, threshold-dependent, gene drive mechanism that could be engineered using a combination of such toxin-antidote and CRISPR components.

Threshold-dependent gene drives are those that will only spread within a target population when introduced above some required threshold frequency^1,19,20^. Conversely, if introduced at a sub-threshold frequency, the gene drive will be disadvantaged relative to wild-type and be eliminated from the population over a timescale determined by the fitness costs associated with the system. These features could be viewed as either positive or negative depending on the target species, application and/or location being considered. For example, via the same mechanism allowing the drive to spread in a target population, a threshold-dependent gene drive can be prevented from doing so in non-target neighbouring populations since these thresholds are unlikely to be exceeded by the natural migration of transgenic individuals alone^3,21^. This feature is important from a regulatory viewpoint since it could prevent the gene drive from crossing geo-political or ecological (e.g. invasive-endemic) borders^22^ which might otherwise lead to significant regulatory and political complications. Another positive aspect for both regulators and the general public is that threshold-dependent systems are more easily reversible than “global” systems^23^. Even in the absence of genetic reversal mechanisms (e.g. for underdominance^24^ or “global” CRISPR approaches^25,26^), threshold-dependent gene drive systems may be reversed by the introduction of sufficient numbers of wild-type individuals to push the transgene frequency below the threshold for that system^27,28^. As discussed above this would lead to the system being eliminated over time, at a rate depending on the system’s disadvantage relative to wild-type. Finally, threshold-dependency of gene drive systems could be viewed as a negative since large releases will often be required to achieve required threshold frequencies. While listed as a benefit in some respects above, this could also be considered disadvantageous since it would potentially result in relatively expensive control programmes due to the large numbers of individuals needed to be reared and released. Another concern associated with the relatively high cost of such systems compared to ‘global’ or extremely low threshold systems is that they might not be as equitable—i.e. their application may only be feasible for wealthier regions.

The novel system proposed here, referred to as split-drive killer-rescue (SDKR), comprises homing and toxin-antidote components, each of which have been, or are currently being developed for other designs of gene drive. These components would be split across two transgenic constructs carried at unlinked and independently segregating loci (say A and B) within the genome of the target species. The first transgenic construct (A) contains a dominant toxin (killer) and one or more single guide RNAs (sgRNAs) targeting the wild-type sequence at locus A. The other (construct B) contains a Cas9 (or other targetable) endonuclease and the antidote (rescue) for the toxin on construct A.

**Figure 1:**
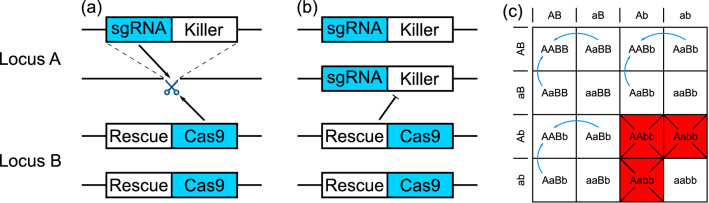
A schematic diagram of the split-drive killer-rescue (SDKR) mechanism of action. (**a**) When at low frequency the system primarily functions as a split CRISPR gene drive in that the Cas9 of construct B is directed to cleave the wild-type sequence at locus A by the sgRNA of construct A. This DSB is repaired using construct A as a template, resulting in an individual homozygous for construct A. (**b**) As construct A reaches higher frequencies, the lethal effector creates a selection pressure for individuals to also carry the rescue component from construct B, resulting in an increasing frequency of construct B. (**c**) A Punnett square demonstrating the mechanism of action of this system. Red squares indicate non-viable genotypes, i.e. those carrying lethal effectors but not the associated suppressors. Blue arrows indicate the effects of the split CRISPR drive components, turning transgene heterozygotes at locus A into transgene homozygotes.

Assuming these constructs were released into the target population at relatively low frequency then the system initially acts similarly to a split drive, although separated A alleles would suffer from the killer gene and be removed. In individuals heterozygous (transgene/wild-type) at locus A and with at least one copy of construct B, the sgRNA (from A) directs the Cas9 (from B) to induce a double stranded break (DSB) in the wild-type sequence at locus A. Homology with the sequence either side of the DSB then allows construct A to be used as a repair template (i.e. homology directed repair; HDR). This results in construct A being copied (homed) into the homologous chromosome, converting heterozygous germline or somatic cells into transgene homozygous cells. This acts to increase the frequency of A but initially does little to affect construct B. However, as construct A becomes more common, the increasing prevalence of the killer element creates a selection pressure for individuals to carry both constructs (A and B), leading to an increasing relative fitness advantage for B as the frequency of A increases.

Another way this system may be described is as having push-and-pull dynamics. In particular, the SDKR system is constantly pushing the toxin-containing element (A) to higher frequency (via homing). This in turn creates a selection pressure that pulls the antidote-carrying element (B) to higher frequencies alongside the toxin. Persistent action of these push and pull components allow the system to persist within a population—subject to various conditions explored later.

In this study we provide theoretical support for the mechanism underpinning the SDKR system via the formulation of population genetics mathematical models. These are used to demonstrate the robustness of the system to transgene fitness costs, homing rates, incomplete lethal penetrance and alternate end-joining repair mechanisms that have previously been shown to generate resistance in CRISPR-based gene drives^14,16,29–36^. We then consider the potential for this system to be used in applications targeting population suppression rather than replacement. In particular, here we show the potential of the system to provide population suppression via either sex ratio distortion or female specific lethality, however a range of alternate mechanisms may also be possible. We also show that, under certain conditions, the suppression approaches considered can affect a target population while remaining confined (i.e. not suppressing neighbouring populations)—a feature not commonly found in current gene drive designs (although some examples do exist, e.g. tethered homing^37^ and TADE^38,39^ drives). Finally, we extend our model to consider the inclusion of an additional transgenic construct (C), containing a sgRNA that directs Cas9 to cut the wild-type sequence at locus B—providing an additional mechanism for B to increase in frequency. We anticipate that SDKR should be reasonably straightforward to develop due to the similarity of genetic components to those in development for alternative purposes. We believe SDKR provides a novel and achievable route to threshold-dependent, reversible and potentially localised gene drive.

## Methods

We consider here a genotype-based population genetics model of SDKR gene drive (Fig. 1). This deterministic mathematical model is based on similar principles to many gene drive models^11,12,34,38,40–42^ in that it assumes an infinite, closed (zero migration—unless otherwise stated), panmictic (randomly mating) population with discrete, non-overlapping generations and a 1:1 (male:female) sex ratio in the initial population, released individuals and offspring of later generations. Further, we assume transgenic constructs are not sex-linked; no resistance nor mutation of transgenic constructs occurs (unless otherwise stated); and components of each construct are perfectly linked (i.e. cannot separate). Finally, for simplicity we assume equal fitness costs for each transgenic construct and that these are applied multiplicatively. This results in a set of 18 difference equations—one for each of the 9 possible genotypes in each of the male and female populations. Full details of this model are given in Supplementary Model 1.

We then consider an extension that allows for an alternative DSB repair mechanism, namely end-joining (e.g. NHEJ). Here, whenever a DSB is made there are two repair probabilities. The first, HDR (also referred to as homing) is described above, while for NHEJ the two ends of the DSB are ligated together in a process known to create indels that act as resistance alleles by preventing recognition of sgRNA target sites. The inclusion of NHEJ leads to a system of 36 difference equations—one for each of the 18 possible genotypes in each of the male and female populations. Full details of this model are given in Supplementary Model 2.

Supplementary Models 1 and 2 are primarily focused on population modification (i.e. spreading desirable genetic traits), however many applications aim for population suppression. Here we formulate an extension to Supplementary Model 1 that considers the use of a sex ratio distorter inserted at a neutral locus (i.e. Locus A). Examples of how this could be arranged include the proposed X-shredder system^43,44^ in *Anopheles* mosquitoes (vectors of malaria) in which sgRNA(s) direct an endonuclease to cleave repeated sequences on the X chromosome, producing a male sex bias; disruption/ectopic expression of the transformer^45^ or Maleness-on-the-Y genes^46^, respectively, in the Mediterranean fruitfly *Ceratitis capitata* or ectopic expression of the nix and myo-sex genes in *Aedes aegypti*^47^. Due to the relative ease with which they can be manipulated genetically, examples are predominantly insect-related however, similar situations exist for the control of invasive vertebrates, for example the incorporation of the Sry gene^48–52^ in house mice, or *aromatase* inhibitor constructs in invasive carp^51^, both of which could convert females into males. At present for mice, females converted into males are functionally sterile^48–50^, however future work may be able to overcome this issue. There are likely many alternative mechanisms in other species that could produce similar effects. Due to the preliminary nature of this study we model a non-specific, sex-ratio distortion element in which some proportion of females carrying construct A are converted into fertile males. Both dominant and recessive forms of female to male sex conversion are considered. We also model scenarios in which population suppression is achieved via either dominant or recessive female-specific lethality or sterility (as used successfully in^13–16^). In these cases, the models are identical to those for female to male sex conversion except that the conversion terms are removed from the equations for males of each genotype. Complete details are given in Supplementary Model 3.

**Figure 2:**
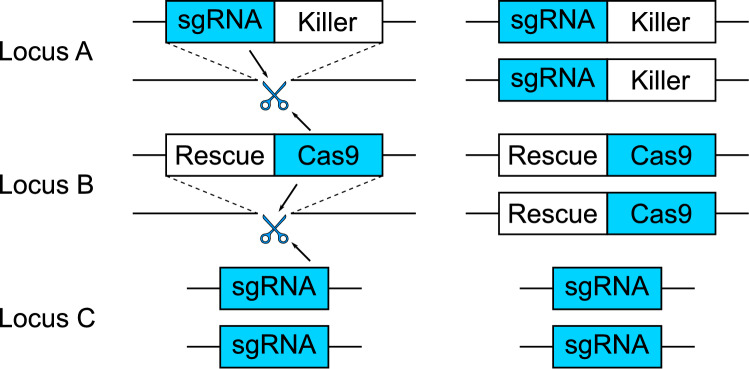
A schematic diagram of a daisy drive killer-rescue (DDKR) system. This is essentially SDKR but modified to include a third transgenic construct at an unlinked locus (C) that contains a sgRNA directing the Cas9 endonuclease to cut the wild-type sequence at locus B.

Finally, we consider the introduction of a further element at a third unlinked locus (as in Fig. 2). This construct (C) contains a sgRNA targeting the wild-type sequence at locus B, thus allowing construct B to be used as a repair template for HDR and increasing in frequency. This system is herein referred to as daisy-drive killer-rescue (DDKR). This model is similar to Supplementary Model 1 except we now have 54 difference equations—one for each of the 27 possible genotypes in each of the male and female populations. Full details of this model are given in Supplementary Model 4.

For each system, we demonstrate potential behaviours via numerical simulation for a range of parameter sets and initial conditions (i.e. release sizes of individuals homozygous for both constructs). In most cases we discretise the entire biologically feasible parameter space and perform numerical simulations at each point in the resulting parameter grid. This allows the extraction of various performance metrics under a wide range of scenarios. All numerical calculations are performed using MATLAB (R2018a, The MathWorks Inc., Natick, MA).

## Results

### Theoretical proof of concept

Before delving into a full analysis of the SDKR system, it is useful to demonstrate how the system behaves when introduced into a target population. We present some numerical simulations by way of a theoretical proof of principle that SDKR is capable of increasing in frequency even where introduced transgenes confer a fitness cost on individuals carrying them.

**Figure 3:**
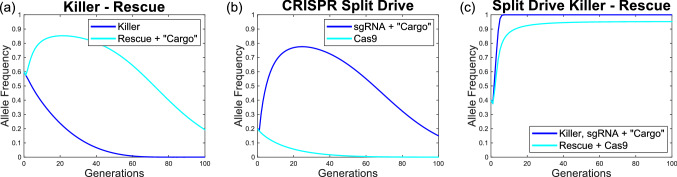
Sample numerical simulations comparing SDKR to its constitutive systems. Results are shown for (**a**) killer-rescue, (**b**) CRISPR split drive and (**c**) SDKR. All cases consider a relative fitness of $\varepsilon =0.95$ per construct, a homing rate of $\Phi =0.9$ (**b**, **c** only) and complete lethal penetrance to affected genotypes (i.e. $L=1$). Relative fitness parameters from two or more transgenic constructs are applied multiplicatively. Note that initial transgene frequencies differ between systems to emphasise their respective drive strengths. Mathematical models for killer-rescue ($\Phi =0$, $L=1$) and split drive ($\Phi >0$, $L=0$) are special cases of Supplementary Model 1. In each case we consider the release of individuals homozygous for both transgenic constructs and fitness costs that are applied multiplicatively.

Both killer-rescue and split drive display a transient increase in frequency of one transgenic construct (Fig. 3a,b). However, Fig. 3c shows the ability of SDKR to increase in frequency once introduced into a target population. In particular, construct A heads toward fixation due to the action of the CRISPR split-drive components while construct B reaches a high frequency as a result of the selection pressure for carrying the rescue transgene. However, construct B does not reach fixation since a single rescue copy fully negates any lethal effects thus setting up a situation in which the heterozygotes for this allele have an advantage over either homozygote—which typically leads to a stable equilibrium at an intermediate frequency (avoided by construct A because of homing). That one transgenic construct reaches fixation even in the face of a fitness cost may provide a slight advantage over traditionally envisaged toxin-antidote based gene drives such as two-locus engineered underdominance (UD) where each construct reaches high frequency but not fixation^41^. Therefore, if the desired genetic cargo were present on construct A then more individuals would become homozygous—a potentially important feature where desired phenotypes (e.g. disease refractoriness for population modification or lethality/sterility for population suppression) are recessive, partially dominant or display incomplete penetrance in a single copy.

### Threshold introduction frequencies

**Figure 4:**
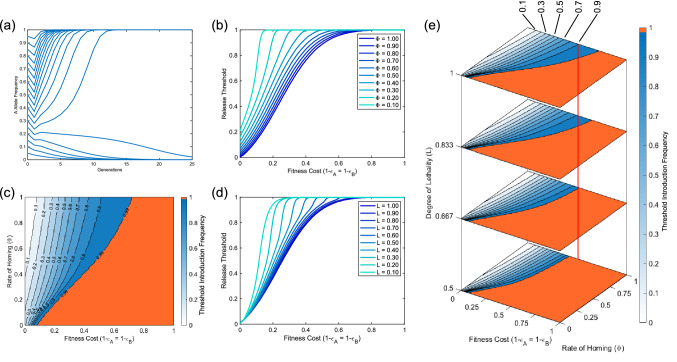
SDKR displays threshold dependent behaviour that is affected by a range of parameters. Panel (**a**) shows sample numerical simulations demonstrating threshold-dependence of SDKR (with homing rate $\Phi =0.9$, relative fitness $\varepsilon =0.85$ and lethal penetrance $L=1$). Panel (**b**) demonstrates how the introduction threshold required for the system to spread (shown on the vertical axes) changes with variation in the rate of homing ($\Phi$) and relative fitness ($\varepsilon$) parameters with $L=1$. Similarly, (**c**) demonstrates the effect on required release thresholds but over the full range of possible homing rates ($\Phi$) with $L=1$. Here an orange area denotes the parameter space in which the system gives a threshold introduction frequency $\ge$0.99 (i.e. where the system is unable to increase in frequency). Panel (**d**) demonstrates the effect of variation in relative fitness ($\varepsilon$) and the degree of lethality conferred by the lethal effector from construct A—with homing rate $\Phi =0.9$. Finally, panel (**e**) shows how initial release thresholds are affected over the full range of relative fitness ($\varepsilon$) and homing rate ($\Phi$) parameters as well as a selection of lethal penetrance parameters (*L*). Here the red line is included to aid visual distinction between the locations of contour lines. As in (**c**), here an orange area denotes parameter space where the system is unable to increase in frequency. Note that in each case we consider the release of individuals homozygous for both transgenic constructs and fitness costs that are applied multiplicatively.

Due to the similarity in mechanisms of action to UD gene drives, we would expect SDKR to display threshold-dependent behaviour. This is a desirable feature since a certain threshold must be exceeded for the system to increase in frequency. Therefore the system is less likely to spread into neighbouring populations and is reversible by simply releasing sufficient wild-type individuals to lower the transgene frequency below the introduction threshold. Preliminary numerical simulations under a range of parameter and introduction scenarios confirm that SDKR is threshold-dependent (Fig. 4a). Thus, we provide a more thorough investigation into release thresholds and how these are affected by parameter variations such as fitness cost, homing rate and lethal penetrance.

We begin by investigating the effects of imperfect homing rates (i.e. $\Phi <1$). Figure 4b demonstrates that an increase in initial release size is required to compensate for either non-zero fitness costs or imperfect homing rates. Promisingly these results show that the system can persist and increase in frequency even in the face of relatively poor rates of homing and/or large fitness costs. In particular, this system is able to tolerate larger fitness costs than a traditionally envisaged two-locus UD system even where homing rates are far from perfect. For example, such a two-locus UD system can tolerate maximum fitness costs of up to $\sim \,30\%$ per construct (when applied multiplicatively) no matter the release size^19,21,41^ (although limits for other UD designs such as TARE^39^ are less restrictive). However, here a 2:1 release (initial transgene frequency of 0.66) only requires a homing rate of $\sim \,70\%$ to tolerate a fitness cost of 30% per construct. Figure 4c shows similar results for the full range of possible homing rates. For a system with complete homing (i.e. $\Phi =1$), results show a pattern similar to *Medea*, CRISPR toxin-antidote and *Wolbachia* systems in that we find an arbitrarily low introduction threshold for zero fitness cost that increases as fitness costs rise^19,39,40,53^. However, as homing rates decrease, the threshold introduction frequencies tend more toward those observed for two-locus engineered underdominance systems^11^. Based on currently available empirical evidence, it would appear that perfect homing is not likely although rates observed vary significantly between target species.

Another potential source of imperfection is an incomplete penetrance of lethal effectors. Figure 4d shows the impact of such incomplete lethal penetrance in the context of a high homing rate ($\Phi =0.9$). Here we see that for high rates of lethal penetrance the effect is minimal, with only very small differences in tolerable fitness costs. However, as the lethal penetrance is reduced further, we see the maximum tolerable fitness costs falling. This creates a scenario more akin to UD where above some maximum tolerable fitness cost, it becomes extremely difficult to compensate with the release of additional transgenic individuals^19,41^.

Figure 4e combines each of the above parameter ranges to demonstrate their relative importance to the thresholds of the SDKR system. For clarity of presentation we only consider lethal effectors that are at least 50% penetrant. Here we see some minor difference due to increases in lethal penetrance, however it would be more beneficial to focus attention on improvements in rates of homing ($\Phi$) or fitness cost ($\varepsilon$) reductions when attempting to engineer SDKR systems for population modification purposes.

### Effects of end-joining repair mechanisms

It is assumed above that every DSB will be repaired via HDR. In reality a range of alternate ‘error-prone’ end-joining repair mechanisms may also repair DSBs, by ligating the broken ends together. Unfortunately, such end-joining repair mechanisms often lead to the generation of indels within the target sequence, preventing sgRNAs from directing the endonuclease to further cleave that sequence. This creates a locus resistant to the effects of the homing system though potentially also disrupting the normal function of the sequence at that locus. Here we use an extended model that includes the effects of NHEJ (used to represent all of the subtly different end-joining repair mechanisms) to explore the effects on SDKR efficacy.

**Figure 5:**
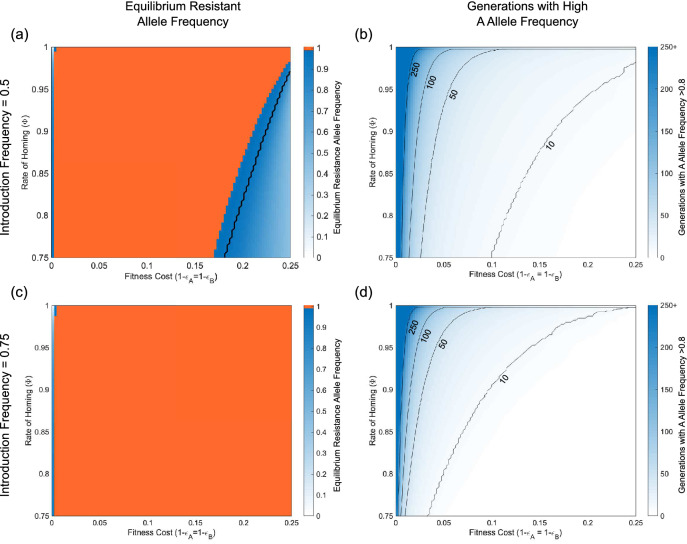
NHEJ leads to the breakdown of SDKR where resistant alleles are of higher fitness than transgenic constructs. Results of numerical simulations using an initial release frequency of (**a**, **b**) 0.5 or (**c**, **d**) 0.75 over a discretised parameter grid. Here, panels (**a**, **c**) show the equilibrium resistance allele frequency (taken after 2000 generations). Orange areas represent the region in which the equilibrium resistant allele frequency equals one. Blue regions (with values shown in colour bars) denote regions in which the equilibrium resistant allele frequency was less than one. In panel (**a**) the region below the black line is that in which the SDKR system fails to increase above its initial release frequency. In the area between the orange region and the yellow line, the drive initially increases in frequency but is then eradicated before the resistant allele frequency has time to reach fixation. Panels (**b**, **d**) show the number of generations in which the A allele frequency is above 0.8. In each case, it is assumed that DSBs are induced in 95% of eligible target sequences with the repair mechanism being indicated by the rate of homing ($\Phi$) in each panel. For example, a value of $\Phi =1$ means repair is exclusively by HDR, whereas $\Phi =0.9$ means that 90% of DSBs are repaired by HDR and the remaining 10% by NHEJ. Note that in each case we consider the release of individuals homozygous for both transgenic constructs and fitness costs that are applied multiplicatively.

Figure 5 shows numerical simulations across a discretised parameter grid for introduction frequencies of 0.5 and 0.75. These results suggest that non-zero NHEJ repair rates will lead to the breakdown of the SDKR system so long as resistant alleles confer a smaller fitness cost than transgenic constructs. Comparing Fig. 5a,c we see that higher introduction frequencies lead to higher equilibrium frequencies for resistant alleles. More importantly, Fig. 5b,d show the system can still persist for a good number of generations under certain parameter scenarios. This suggests that higher initial release frequencies lead to a greater persistence of the A allele—although the difference is relatively minor across most of the parameter space considered. Here we consider the persistence of the A allele at a frequency above 0.8 as a reasonable proxy for the period in which SDKR could be effective. For a gene at Hardy-Weinberg equilibrium, this would result in over 95% of individuals carrying at least one copy of the A allele. While we consider this a reasonable measure here, this value will likely vary significantly by application and target species.

### Potential for population suppression

Results above demonstrate the potential of SDKR as a population modification strategy. However, a number of proposed applications seek to suppress rather than modify target populations. Here we explore the potential for using SDKR to suppress a population via the incorporation of either a sex-ratio distortion element or a female-specific lethal element into construct A. We also consider whether the threshold-dependency of this system may lead to population suppression that is unable to spread beyond a target population. This is modelled via the consideration of unidirectional migration from the target population into a neighbouring population. Migrants are scaled based on the relative degrees of suppression (as measured by the genetic load) in the target and non-target populations. In practice population dynamic, behavioural and ecological traits of target populations/species will also likely have a large impact on the degree of suppression achieved. These factors are beyond the scope of this study and so representative examples are given for four approaches, namely dominant/recessive female to male sex conversion and dominant/recessive female-specific lethality. In each case the effector is located on the A allele.

**Figure 6:**
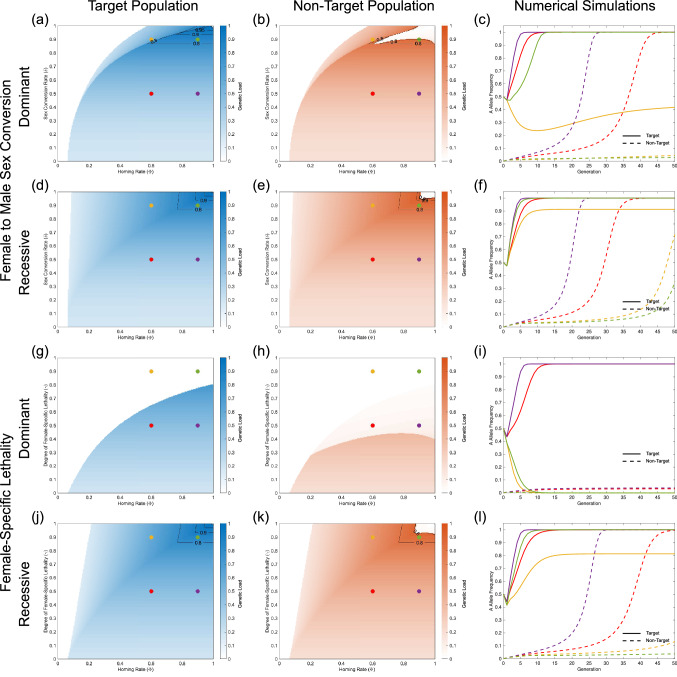
SDKR can, under certain conditions, achieve significant and localised population suppression. Results shown are for strategies based on dominant (**a**–**c**) or recessive (**d**–**f**) female to male sex conversion and dominant (**g**–**i**) or recessive (**j**–**l**) female-specific lethality. Heat maps display the equilibrium genetic load imposed on a target (left column; blue) and a non-target population (centre column; orange). Black contour lines are shown for genetic loads of 80%, 90%, 95% and 99%. Here, the equilibrium state is assessed 1000 generations after the release of transgenic individuals. The right column shows specific examples for parameter combinations indicated by coloured dots in the left and centre columns (i.e. combinations of homing rate $\Phi =0.6$ or 0.9 and female to male sex conversion ($\delta =0.5$ or 0.9) or female-specific lethality ($\gamma =0.5$ or 0.9)). Solid lines represent transgene allele frequencies in a target population and dashed lines are the equivalent for a non-target population. In all cases, simulations consider a relative fitness of $\varepsilon =0.95$ per construct (applied multiplicatively) and fully penetrant lethal effects (i.e. $L=1$). In each case we consider the release of individuals homozygous for both transgenic constructs and fitness costs that are applied multiplicatively. Migration is considered to be unidrectional at a rate of 2% per generation. It is also worth noting that fitness costs associated with transgenic constructs can produce a small genetic load even where sex conversion and/or female-specific lethality are absent. Supplementary Figures S1 and S2 show equivalent results for fitness costs of 15% and 10% per construct, respectively and demonstrate the importance of fitness costs in determining the ability of such systems to produce confined population suppression.

Figure 6a–c shows results for SDKR with dominant female to male sex conversion—i.e. individuals carrying one or more copies of construct A develop as fertile males. In the examples shown, this system can confer a large genetic load on the target population—up to $\sim \,95\%$. Despite producing a large degree of population suppression in the target population, the system displays a range of outcomes within the non-target population. For the majority of parameter space considered the suppression is of a similar degree to that in the target population, however for high rates of homing and sex conversion there is a region of parameter space in which the system may produce a negligible genetic load in the non-target population. For examples with greater fitness costs the degree of confinement is much improved, with significantly larger regions of parameter space giving confined population suppression in the target population (see Figures S1 and S2). It is also worth noting here that, even where transgenic constructs give extremely small fitness costs, this approach fails where both the rate of homing and sex conversion are 100%—essentially becoming a RIDL-with-drive system^54,55^ but with an inherent 50% fitness cost as it is not possible to arrange that all individuals inheriting the lethal allele A (100% of progeny) would also inherit a copy of the rescue allele B. It is currently unclear how feasible incomplete penetrance of sex conversion would be to arrange molecularly since non-converted individuals may more likely be intersex, sterile or non-viable. However, X-shredder systems in insects (e.g.^56^) have resulted in high, but incomplete, sex conversion with unconverted individuals appearing to be fertile.

For SDKR with recessive female to male sex conversion (Fig. 6d–f) any individual homozygous for construct A will develop as a fertile male. Here we find similar genetic loads to the dominant conversion case may be conferred on the target population, although the region of parameter space in which this can occur is much larger (Fig. 6d). Likewise, we see similar genetic loads to the dominant simulations for the non-target neighbouring population—again with an increased parameter range displaying a non-zero genetic load at equilibrium (Fig. 6e). In contrast to the dominant sex conversion case, here we are able to produce a large genetic load even in cases with 100% homing and homozygous sex conversion. This is likely because female gene drive carriers survive when the system is at low-medium frequencies and then start to be converted into males only when the drive has already spread into a large proportion of the population. As in the dominant example, it may be extremely difficult to arrange incomplete penetrance of sex conversion. In this case, where sex conversion is 100% effective and constructs confer a fitness cost of 5%, SDKR can produce a significant genetic load so long as the rate of homing is greater than $\sim \,10\%$—although this becomes much more restrictive as fitness cost increase. For example, a fitness cost of 15% per construct requires a homing rate of $\sim \,40\%$ to produce a significant genetic load where sex conversion is 100% effective (see Figure S1).

Figure 6g–i shows results for SDKR with a dominant female-specific lethal effector—i.e. females carrying one or more copies of construct A suffer a lethal effect. This system is found to be capable of conferring a genetic load of $\sim \,65\%$, which is unlikely to be sufficient to suppress a pest population to a large degree. When compared to results for female to male sex conversion, this approach also has a significantly reduced parameter space in which population suppression is observed at equilibrium. In this case, we see a large region of parameter space in which this system produces only a small genetic load on a non-target neighbouring population ($<\,0.1$ for a 2% migration rate per generation) in spite of producing a significant fitness load in the target population.

For a case with a recessive female-specific lethal effector (Fig. 6j–l)—similar to those of gene drives in anopheline mosquitoes^13–16^—we observe an ability to produce large genetic loads on a target population (Fig. 6j), of a magnitude similar to those of female to male sex conversion cases (i.e. $\sim \,95\%$). As seen in the sex conversion cases, here the genetic load imposed on a non-target population was similar to that for the target population apart from a region with high homing rates and female-specific lethality where suppression of the non-target population was minimal (Fig. 6k). In the case of 100% effective female-specific lethality and the 5% fitness cost per construct considered here, SDKR can produce a significant genetic load in the target population so long as the homing rate is greater than $\sim \,20\%$—although restrictions on the homing rate are tighter where constructs produce larger fitness costs (see Figures S1 and S2).

While results shown here demonstrate limited localised population suppression, for cases with increased fitness costs (as in Figures S1 and S2) dominant and recessive female to male sex conversion or recessive female-specific lethality display greater potential. Thus, the global or localised nature of any population suppression obtained using the SDKR system is not guaranteed and would need to be considered carefully for any application prior to development and subsequent release. Also, while the genetic load has often been used as a proxy^37,57^, the degree of population suppression observed will likely be species and/or location specific.

### Daisy-drive killer-rescue

As described above, it is possible to include an additional transgenic construct at a third unlinked locus (C) to form a DDKR system (see Fig. 2). Previous literature has shown that daisy chain gene drive systems with three components (where C drives B and B drives A) are capable of reaching higher frequencies than a simple split-drive configuration (where B drives A)^10^ from similarly sized releases. Here we explore whether a similar effect will produce lower introduction thresholds for DDKR when compared with those of SDKR.

**Figure 7:**
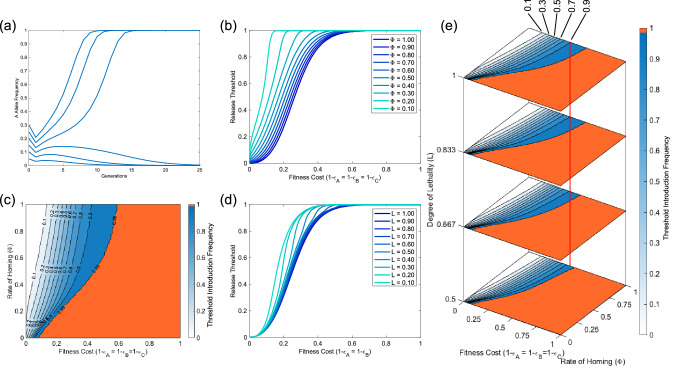
DDKR displays threshold dependent behaviour that is affected by a range of parameters. Panel (**a**) shows sample numerical simulations demonstrating the threshold-dependent nature of the DDKR system (with homing rate $\Phi =0.9$, relative fitness $\varepsilon =0.85$ and lethal penetrance $L=1$). Panel (**b**) demonstrates how the required introduction threshold changes with variation in the rate of homing parameter ($\Phi$) while $L=1$. Similarly, (**c**) demonstrates the effect on required release thresholds but over the full range of possible homing rates ($\Phi$) while $L=1$. Here an orange area denotes the parameter space in which the system gives a threshold introduction frequency $\ge$0.99 (i.e. where the system is unable to increase in frequency). Panel (**d**) demonstrates the effect of variation in the degree of lethality conferred by the lethal effector from construct A, for a homing rate $\Phi =0.9$. Finally, panel (**e**) shows how initial release thresholds are affected over the full range of relative fitness ($\varepsilon$) and homing rate ($\Phi$) parameters as well as a selection of lethal penetrance parameters (*L*). Here the red line is included to aid visual distinction between the locations of contour lines. As in (**c**), here an orange area denotes parameter space where the system is unable to increase in frequency. Note that in each case we consider the release of individuals homozygous for both transgenic constructs and fitness costs that are applied multiplicatively.

**Figure 8:**
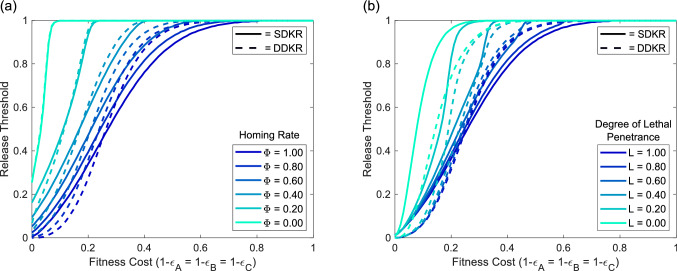
A comparison of required introduction thresholds for SDKR and DDKR. (**a**) Threshold introduction frequencies for SDKR (solid lines) and DDKR (dashed lines) under a range of different rates of homing ($\Phi$). (**b**) Threshold introduction frequencies for SDKR (solid lines) and DDKR (dashed lines) for a range of lethal penetrance (*L*) parameters. Line colours relate to the homing rate or degree of lethal penetrance considered with details given in the respective figure legends. Note that in each case we consider the release of individuals homozygous for both transgenic constructs and fitness costs that are applied multiplicatively.

Figure 7a shows sample numerical simulations demonstrating the ability of DDKR to increase in frequency and its threshold dependency. For the parameter set considered here ($\Phi =0.9$, $\varepsilon =0.85$, $L=1$) DDKR (Fig. 7) displays lower introduction thresholds than SDKR (Fig. 4a). As before, we then provide a thorough investigation into the effects of various system parameters (in Fig. 7b–e). In general, results display a similar overall pattern of behaviour to SDKR (Fig. 4). Figure 8 provides a comparison of the SDKR and DDKR systems, highlighting their main differences.

For low fitness costs we see a reduction in threshold introduction frequencies for DDKR when compared to SDKR (Fig. 8). This is true for both imperfect homing (Fig. 8a) and incomplete lethal penetrance (Fig. 8b). Conversely, for larger fitness costs DDKR actually gives larger threshold introduction frequencies than SDKR. This is likely due to a balance between the strength of the drive and additional fitness costs from carrying a third transgenic construct. At low fitness costs the increased drive strength of DDKR lowers release thresholds whereas, when fitness costs rise, the additional cost from construct C allows the overall fitness cost to dominate and increase release thresholds. This holds for most cases except those with low lethal penetrance (below $L\approx 0.5$). Here DDKR has a lower release threshold for all fitness cost parameters. While a reduction in release thresholds can be beneficial from a cost perspective, it may also be viewed as negative since it will increase the likelihood of transgenes spreading beyond the initial target population.

## Discussion

Here we have formulated and analysed population genetics models of the proposed SDKR gene drive system. Numerical simulation of this model demonstrated that this system is capable of spreading introduced transgenes to high frequency in a target population. Further investigation then demonstrated a threshold dependency and robustness to changes in fitness costs, homing rates and lethal penetrance. The introduction thresholds obtained for a system with perfect homing resemble those of *Medea*, CRISPR toxin-antidote and *Wolbachia*-type systems in that they are extremely low for small fitness costs but increase as fitness costs rise^19,39,40,53^. However, as the rate of homing decreases to levels observed in many real-world demonstrations of CRISPR/Cas9 drives^13,30,58–62^, threshold introduction frequencies rise to levels similar to those observed for an efficient two-locus engineered underdominance system^11^. A model extension then revealed that the creation of resistant alleles by end-joining repair of DSBs will eliminate SDKR over time if such alleles confer smaller fitness costs than SDKR constructs. Despite this, the system can still persist at high frequency over biologically relevant timescales. We also investigated the potential for SDKR to be used for applications aimed at population suppression. Models were formulated and analysed for SDKR systems with a dominant or recessive female to male sex conversion or female-specific lethality phenotype associated with construct A. The approach based on dominant female-specific lethality was only able to produce a genetic load of $\sim \,65\%$ in a target population which is likely inadequate to produce significant population suppression for most potential target pest species. However the other three approaches were able to produce large genetic loads (> 95%) although for the dominant female to male conversion design this was reliant on the incomplete penetrance of the conversion phenotype—a situation which may be difficult to arrange whilst preserving the fitness of those construct A carriers which are not fully converted. A limitation of these preliminary investigations was that we did not explore the impact of NHEJ resistance mutations arising at the target site. If, however, as could be envisaged for the recessive conversion/lethal designs presented here, the A construct disrupted by insertional mutagenesis its target endogenous gene, fitness advantages of NHEJ ‘escapee’ alleles could be minimised by targeting functionally constrained sequences. This approach has been demonstrated in conventional CRISPR/Cas9 homing drives^15^. The multiplexing of sgRNAs represents another feasible option^62–64^ and should ensure that resistance alleles disrupting the target sequence predominate over those that preserve function—especially useful when targeting a functionally constrained sequence. Finally, an alternate population modification system (DDKR) created by adding a third construct C (sgRNA targeting the wild-type sequence at locus B) was investigated. In general, this found lower introduction thresholds where fitness costs are small but higher thresholds when fitness costs are high. For very low levels of lethal penetrance, however, we found DDKR to produce lower thresholds for all fitness costs.

Another feature of SDKR that differs from many other systems is its tolerance of embryonic carryover (maternal deposition) of the Cas9 and/or sgRNA components. This has been shown to be a significant issue in some other CRISPR-based gene drive systems (for example^16,30,62^) but could have a lesser effect here. In our proposed system maternal deposition will likely have the greatest impact when transgenic construct A is inherited. In the absence of construct B, these individuals should be killed by unsuppressed lethal effector(s) and some degree of homing will only increase that likelihood. Conversely, one copy of construct B is intended to rescue against two copies of the lethal effector and so construct A heterozygote/homozygote mosaic individuals should survive. Deposition of both the Cas9 and sgRNA into individuals not inheriting either construct could result in some lethality—however this effect is only possible in the offspring of individuals carrying B and so it would likely lead to a slight increase in the frequency of B. The precise impact of this feature on the ability of SDKR to tolerate embryonic carryover is an area that could be addressed with further mathematical modelling.

When discussing modelling results such as those presented here it is important to place them in the context of available experimental literature. The key parameters throughout this study are the rates of homing and fitness costs of transgenic constructs. Fitness costs are likely to vary significantly depending on a wide range of factors including construct design and components as well as their respective insertion site(s) within the target species’ genome. These values could theoretically vary over the entire range from zero fitness cost to almost complete lethality and so they are hard to pin down. There are however many experimental measurements available for homing rates in a range of target species. These vary over a wide range from the $\sim$ 37–82% observed in *Drosophila melanogaster*^29,30,62,64,65^ up to the $\sim$ 89–98% seen in *Anopheles gambiae*^13,15^ and *Anopheles stephensi*^58^ with low values found in *Aedes aegypti*^59^ and mice ^60^. This range of potential homing rates is firmly within the region of parameter space allowing the SDKR and DDKR systems to perform effectively, both within population modification and suppression contexts, thus providing further support for the feasibility of these approaches.

The models studied here are based on several simplifying assumptions, discussed in the Methods section. The validity of these assumptions has also been discussed extensively elsewhere (for example^3,11,41,66^) and so is not discussed further here. There are however some more specific limitations that are worth discussing. Firstly, we assume that DSBs are repaired by either HDR or end-joining. However, experimental studies have demonstrated other outcomes including partial/incomplete homing of transgenic constructs^30,61,62^. We would anticipate this will lead to similar effects as end-joining induced resistant alleles although the fitness cost associated with such events is currently unclear. Another limitation here is the consideration of a population with zero polymorphism at sgRNA target sites; i.e. there is no standing genetic variation in the target sequence(s), which might represent pre-existing homing-resistant alleles. Functionally constrained ‘invariant’ targets are described above but when considering a neutral (i.e. ‘has no effect on adaptation because all genotypes have the same fitness’^67^) target site there is more likely to be polymorphism present in the population and the target site may even be absent in some individuals. This may be partially compensated for by multiplexing (i.e. targeting multiple sequences at the same locus^9,43^) since an individual would need to be resistant at all target sites for the drive to fail^9,68^. However, simultaneous expression of sgRNAs could lead to multiple simultaneous DSBs and the subsequent deletion of multiple sgRNA target sites in a single end-joining repair event^35,62,64,69–71^, although population-level designs may mitigate this risk^72^.

Throughout this study we have considered one base-level design for the SDKR and DDKR gene drive systems proposed. While these represent a feasible design both in terms of function and engineering there are likely alternative designs that could produce similar systems and dynamics. For example, the two-locus two-toxin design based on CRISPR TARE drives^38^ could be tweaked such that an extra sgRNA were added to one construct, allowing it to act as a homing drive and should therefore display similar properties to the SDKR drives proposed here. Of course, the sgRNAs in this alternate design would need to be arranged such that they were only usable by one of the nucleases, however this should be feasible given currently available technology.

Similarly, while we have proposed a number of designs that could produce confined population suppression under appropriate conditions, alternate designs are likely to exist. For instance, the presence of a nuclease within the basic SDKR design should allow for an approach similar to recently proposed tethered homing drives^37^. As discussed above, the basic threshold-dependent SDKR design could be used for confined population modification of a target area. A subsequent introduction of a third (or fourth in the case of DDKR) construct consisting of sgRNA(s) that would direct the nuclease of the SDKR system to also target and disrupt an essential gene, could cause lethality/sterility and thus population suppression. Since the base SDKR system would be unable to spread significantly beyond the initial target population, any suppression would also be confined to this same region as the required nuclease would not be present elsewhere. This could form an interesting basis for a future theoretical study.

This study proposes a novel and promising class of gene drive that may be engineered by combining toxin-antidote and CRISPR components already widely in development. It has also begun to explore and define the relationships between the efficacy of the system and a range of key system parameters. Additional modelling would likely be able to refine these relationships yet further.

## Supplementary information

Supplementary material 1

Supplementary material 2

Supplementary material 3

Supplementary material 4

Supplementary material 5

Supplementary material 6

## Data Availability

Details of mathematical models used to generate all results are included in the main text and associated supplementary information. All MATLAB scripts used throughout this manuscript are available via the Open Science Framework (osf.io/kfv37).
