# Supplementary material for: Split drive killer-rescue provides a novel threshold-dependent gene drive
Source: Sci Rep. 2020 Nov 25;10:20520. doi: 10.1038/s41598-020-77544-7 (PMC7689494; doi:10.1038/s41598-020-77544-7)
Supplement: Supplementary file 1 — Supplementary material 1 [file 41598_2020_77544_MOESM1_ESM.pdf]

Split drive killer-rescue provides a novel threshold-dependent gene drive

Matthew P. Edgington[1*], Tim Harvey-Samuel[1] and Luke Alphey[1]

[1]The Pirbright Institute, Ash Road, Woking, Surrey GU24 0NF, UK
[*]Corresponding author: matt.edgington@pirbright.ac.uk

Supplementary Model 1

We begin here by defining a baseline set of parameter values for use throughout the study. This is as follows:

- $L = 1$ (fully penetrant lethal effect),
- $\varepsilon_A = 0.85$ (relative to 1 in wild-type individuals - 15% fitness cost),
- $\varepsilon_B = 0.85$ (relative to 1 in wild-type individuals - 15% fitness cost),
- $\Phi = 0.9$ (90% rate of homing).

The relative fitness parameters above (ε_A and ε_B) are combined multiplicatively such that we are able to obtain a single relative fitness value for individuals of each genotype (that is assumed to be equal in both sexes). These are of the form:

$$\Omega_{bbaa} = 1, \quad \Omega_{bbAa} = (1-L)\varepsilon_A, \quad \Omega_{bbAA} = (1-L)\varepsilon_A^2, \quad \Omega_{Bbaa} = \varepsilon_B, \quad \Omega_{BbAa} = \varepsilon_B\varepsilon_A,$$
$$\Omega_{BbAA} = \varepsilon_A\varepsilon_A^2, \quad \Omega_{BBaa} = \varepsilon_B^2, \quad \Omega_{BBAa} = \varepsilon_B^2\varepsilon_A, \quad \Omega_{BBAA} = \varepsilon_B^2\varepsilon_A^2,$$

where $(1-L)$ represents the lethal effect conferred on genotypes that carry at least one copy of construct A and zero copies of construct B.

We then define a set of initial conditions that represent the genotype proportions in the population at the point where transgenic individuals are introduced. For a 1:1 (introduced:wild) release of individuals homozygous for both transgenic constructs, as is most widely considered in this study these are of the form:

$$M_{bbaa}(1) = 0.25 = F_{bbaa}(1), \quad M_{bbAa}(1) = 0.00 = F_{bbAa}(1), \quad M_{bbAA}(1) = 0.00 = F_{bbAA}(1),$$
$$M_{Bbaa}(1) = 0.00 = F_{Bbaa}(1), \quad M_{BbAa}(1) = 0.00 = F_{BbAa}(1), \quad M_{BbAA}(1) = 0.00 = F_{BbAA}(1),$$
$$M_{BBaa}(1) = 0.00 = F_{BBaa}(1), \quad M_{BBAa}(1) = 0.00 = F_{BBAa}(1), \quad M_{BBAA}(1) = 0.25 = F_{BBAA}(1).$$

With the above definitions we are then able to iteratively calculate the genotype frequencies for each subsequent generation. This process is conducted in a two step manner. The first stage is to calculate the proportional frequencies for each genotype (and for each sex), which is achieved using:

$$\begin{aligned}
M_{bbaa}^e = \left(\frac{\Omega_{bbaa}}{2}\right) & [M_{bbaa}(i-1)F_{bbaa}(i-1) + 0.5M_{bbaa}(i-1)F_{bbAa}(i-1) + 0.5M_{bbaa}(i-1)F_{Bbaa}(i-1) \\
& + 0.25M_{bbaa}(i-1)(1-\Phi)F_{BbAa}(i-1) + 0.5M_{bbAa}(i-1)F_{bbaa}(i-1) + 0.25M_{bbAa}(i-1)F_{bbAa}(i-1) \\
& + 0.25M_{bbAa}(i-1)F_{Bbaa}(i-1) + 0.125M_{bbAa}(i-1)(1-\Phi)F_{BbAa}(i-1) + 0.5M_{Bbaa}(i-1)F_{bbaa}(i-1) \\
& + 0.25M_{Bbaa}(i-1)F_{bbAa}(i-1) + 0.25M_{Bbaa}(i-1)F_{Bbaa}(i-1) + 0.125M_{Bbaa}(i-1)(1-\Phi)F_{BbAa}(i-1) \\
& + 0.25(1-\Phi)M_{BbAa}(i-1)F_{bbaa}(i-1) + 0.125(1-\Phi)M_{BbAa}(i-1)F_{bbAa}(i-1) \\
& + 0.125(1-\Phi)M_{BbAa}(i-1)F_{Bbaa}(i-1) + 0.0625(1-\Phi)M_{BbAa}(i-1)(1-\Phi)F_{BbAa}(i-1)],
\end{aligned}$$

$$\begin{aligned}
M_{bbAa}^e = \left(\frac{\Omega_{bbAa}}{2}\right) & [0.5M_{bbaa}(i-1)F_{bbAa}(i-1) + M_{bbaa}(i-1)F_{bbAA}(i-1) + 0.25M_{bbaa}(i-1)(1-\Phi)F_{BbAa}(i-1) \\
& + 0.5M_{bbaa}(i-1)\Phi F_{BbAa}(i-1) + 0.5M_{bbaa}(i-1)F_{BbAA}(i-1) + 0.5M_{bbAa}(i-1)F_{bbaa}(i-1)
\end{aligned}$$

$$
\begin{aligned}
&+0.5M_{bbAa}(i-1)F_{bbAa}(i-1)+0.5M_{bbAa}(i-1)F_{bbAA}(i-1)+0.25M_{bbAa}(i-1)F_{Bbaa}(i-1)\\
&+0.25M_{bbAa}(i-1)(1-\Phi)F_{BbAa}(i-1)+0.25M_{bbAa}(i-1)\Phi F_{BbAa}(i-1)+0.25M_{bbAa}(i-1)F_{BbAA}(i-1)\\
&+M_{bbAA}(i-1)F_{bbaa}(i-1)+0.5M_{bbAA}(i-1)F_{bbAa}(i-1)+0.5M_{bbAA}(i-1)F_{Bbaa}(i-1)\\
&+0.25M_{bbAA}(i-1)(1-\Phi)F_{BbAa}(i-1)+0.25M_{Bbaa}(i-1)F_{bbAa}(i-1)+0.5M_{Bbaa}(i-1)F_{bbAA}(i-1)\\
&+0.125M_{Bbaa}(i-1)(1-\Phi)F_{BbAa}(i-1)+0.25M_{Bbaa}(i-1)\Phi F_{BbAa}(i-1)+0.25M_{Bbaa}(i-1)F_{BbAA}(i-1)\\
&+0.25(1-\Phi)M_{BbAa}(i-1)F_{bbaa}(i-1)+0.5\Phi M_{BbAa}(i-1)F_{bbaa}(i-1)\\
&+0.25(1-\Phi)M_{BbAa}(i-1)F_{bbAa}(i-1)+0.25\Phi M_{BbAa}(i-1)F_{bbAa}(i-1)\\
&+0.25(1-\Phi)M_{BbAa}(i-1)F_{bbAA}(i-1)+0.125(1-\Phi)M_{BbAa}(i-1)F_{Bbaa}(i-1)\\
&+0.25\Phi M_{BbAa}(i-1)F_{Bbaa}(i-1)+0.125(1-\Phi)M_{BbAa}(i-1)(1-\Phi)F_{BbAa}(i-1)\\
&+0.125\Phi M_{BbAa}(i-1)(1-\Phi)F_{BbAa}(i-1)+0.125(1-\Phi)M_{BbAa}(i-1)\Phi F_{BbAa}(i-1)\\
&+0.125(1-\Phi)M_{BbAa}(i-1)F_{BbAA}(i-1)+0.125(1-\Phi)M_{BbAa}(i-1)\Phi F_{BBAa}(i-1)\\
&+0.5M_{BbAA}(i-1)F_{bbaa}(i-1)+0.25M_{BbAA}(i-1)F_{bbAa}(i-1)+0.25M_{BbAA}(i-1)F_{Bbaa}(i-1)\\
&+0.125M_{BbAA}(i-1)(1-\Phi)F_{BbAa}(i-1)],
\end{aligned}
$$

$$
\begin{aligned}
M^e_{bbAA}=\left(\frac{\Omega_{bbAA}}{2}\right)&[0.25M_{bbAa}(i-1)F_{bbAa}(i-1)+0.5M_{bbAa}(i-1)F_{bbAA}(i-1)\\
&+0.125M_{bbAa}(i-1)(1-\Phi)F_{BbAa}(i-1)+0.25M_{bbAa}(i-1)\Phi F_{BbAa}(i-1)+0.25M_{bbAa}(i-1)F_{BbAA}(i-1)\\
&+0.5M_{bbAA}(i-1)F_{bbAa}(i-1)+M_{bbAA}(i-1)F_{bbAA}(i-1)+0.25M_{bbAA}(i-1)(1-\Phi)F_{BbAa}(i-1)\\
&+0.5M_{bbAA}(i-1)\Phi F_{BbAa}(i-1)+0.5M_{bbAA}(i-1)F_{BbAA}(i-1)+0.125(1-\Phi)M_{BbAa}(i-1)F_{bbAa}(i-1)\\
&+0.25\Phi M_{BbAa}(i-1)F_{bbAa}(i-1)+0.25(1-\Phi)M_{BbAa}(i-1)F_{bbAA}(i-1)+0.5\Phi M_{BbAa}(i-1)F_{bbAA}(i-1)\\
&+0.0625(1-\Phi)M_{BbAa}(i-1)(1-\Phi)F_{BbAa}(i-1)+0.125\Phi M_{BbAa}(i-1)(1-\Phi)F_{BbAa}(i-1)\\
&+0.125(1-\Phi)M_{BbAa}(i-1)\Phi F_{BbAa}(i-1)+0.25\Phi M_{BbAa}(i-1)\Phi F_{BbAa}(i-1)\\
&+0.125(1-\Phi)M_{BbAa}(i-1)F_{BbAA}(i-1)+0.25\Phi M_{BbAa}(i-1)F_{BbAA}(i-1)\\
&+0.125(1-\Phi)M_{BbAa}(i-1)\Phi F_{BBAa}(i-1)+0.25M_{BbAA}(i-1)F_{bbAa}(i-1)+0.5M_{BbAA}(i-1)F_{bbAA}(i-1)\\
&+0.125M_{BbAA}(i-1)(1-\Phi)F_{BbAa}(i-1)+0.25M_{BbAA}(i-1)\Phi F_{BbAa}(i-1)\\
&+0.25M_{BbAA}(i-1)F_{BbAA}(i-1)],
\end{aligned}
$$

$$
\begin{aligned}
M^e_{Bbaa}=\left(\frac{\Omega_{Bbaa}}{2}\right)&[0.5M_{bbaa}(i-1)F_{Bbaa}(i-1)+0.25M_{bbaa}(i-1)(1-\Phi)F_{BbAa}(i-1)+M_{bbaa}(i-1)F_{BBaa}(i-1)\\
&+0.5M_{bbaa}(i-1)(1-\Phi)F_{BBAa}(i-1)+0.25M_{bbAa}(i-1)F_{Bbaa}(i-1)\\
&+0.125M_{bbAa}(i-1)(1-\Phi)F_{BbAa}(i-1)+0.5M_{bbAa}(i-1)F_{BBaa}(i-1)\\
&+0.25M_{bbAa}(i-1)(1-\Phi)F_{BBAa}(i-1)+0.5M_{Bbaa}(i-1)F_{bbaa}(i-1)+0.25M_{Bbaa}(i-1)F_{bbAa}(i-1)\\
&+0.5M_{Bbaa}(i-1)F_{Bbaa}(i-1)+0.25M_{Bbaa}(i-1)(1-\Phi)F_{BbAa}(i-1)+0.5M_{Bbaa}(i-1)F_{BBaa}(i-1)\\
&+0.25M_{Bbaa}(i-1)(1-\Phi)F_{BBAa}(i-1)+0.25(1-\Phi)M_{BbAa}(i-1)F_{bbaa}(i-1)\\
&+0.125(1-\Phi)M_{BbAa}(i-1)F_{bbAa}(i-1)+0.25(1-\Phi)M_{BbAa}(i-1)F_{Bbaa}(i-1)\\
&+0.125(1-\Phi)M_{BbAa}(i-1)(1-\Phi)F_{BbAa}(i-1)+0.25(1-\Phi)M_{BbAa}(i-1)F_{BBaa}(i-1)\\
&+0.125(1-\Phi)M_{BbAa}(i-1)(1-\Phi)F_{BBAa}(i-1)+M_{BBaa}(i-1)F_{bbaa}(i-1)+0.5M_{BBaa}(i-1)F_{bbAa}(i-1)\\
&+0.5M_{BBaa}(i-1)F_{Bbaa}(i-1)+0.25M_{BBaa}(i-1)(1-\Phi)F_{BbAa}(i-1)\\
&+0.5(1-\Phi)M_{BBAa}(i-1)F_{bbaa}(i-1)+0.25(1-\Phi)M_{BBAa}(i-1)F_{bbAa}(i-1)\\
&+0.25(1-\Phi)M_{BBAa}(i-1)F_{Bbaa}(i-1)+0.125(1-\Phi)M_{BBAa}(i-1)(1-\Phi)F_{BbAa}(i-1)],
\end{aligned}
$$

$$
\begin{aligned}
M^e_{BbAa}=\left(\frac{\Omega_{BbAa}}{2}\right)&[0.25M_{bbaa}(i-1)(1-\Phi)F_{BbAa}(i-1)+0.5M_{bbaa}(i-1)\Phi F_{BbAa}(i-1)\\
&+0.5M_{bbaa}(i-1)F_{BbAA}(i-1)+0.5M_{bbaa}(i-1)(1-\Phi)F_{BBAa}(i-1)+M_{bbaa}(i-1)\Phi F_{BBAa}(i-1)\\
&+M_{bbaa}(i-1)F_{BBAA}(i-1)+0.25M_{bbAa}(i-1)F_{Bbaa}(i-1)+0.25M_{bbAa}(i-1)(1-\Phi)F_{BbAa}(i-1)\\
&+0.25M_{bbAa}(i-1)\Phi F_{BbAa}(i-1)+0.25M_{bbAa}(i-1)F_{BbAA}(i-1)+0.5M_{bbAa}(i-1)F_{BBaa}(i-1)\\
&+0.5M_{bbAa}(i-1)(1-\Phi)F_{BBAa}(i-1)+0.5M_{bbAa}(i-1)\Phi F_{BBAa}(i-1)+0.5M_{bbAa}(i-1)F_{BBAA}(i-1)\\
&+0.5M_{bbAA}(i-1)F_{Bbaa}(i-1)+0.25M_{bbAA}(i-1)(1-\Phi)F_{BbAa}(i-1)+M_{bbAA}(i-1)F_{BBaa}(i-1)\\
&+0.5M_{bbAA}(i-1)(1-\Phi)F_{BBAa}(i-1)+0.25M_{Bbaa}(i-1)F_{bbAa}(i-1)+0.5M_{Bbaa}(i-1)F_{bbAA}(i-1)\\
&+0.25M_{Bbaa}(i-1)(1-\Phi)F_{BbAa}(i-1)+0.5M_{Bbaa}(i-1)\Phi F_{BbAa}(i-1)+0.5M_{Bbaa}(i-1)F_{BbAA}(i-1)
\end{aligned}
$$

$$
\begin{aligned}
&+0.25M_{Bbaa}(i-1)(1-\Phi)F_{BBAa}(i-1)+0.5M_{Bbaa}(i-1)\Phi F_{BBAa}(i-1)+0.5M_{Bbaa}(i-1)F_{BBAA}(i-1)\\
&+0.25(1-\Phi)M_{BbAa}(i-1)F_{bbAa}(i-1)+0.25\Phi M_{BbAa}(i-1)F_{bbAa}(i-1)\\
&+0.25(1-\Phi)M_{BbAa}(i-1)F_{bbAA}(i-1)+0.25(1-\Phi)M_{BbAa}(i-1)F_{Bbaa}(i-1)\\
&+0.5\Phi M_{BbAa}(i-1)F_{Bbaa}(i-1)+0.25(1-\Phi)M_{BbAa}(i-1)(1-\Phi)F_{BbAa}(i-1)\\
&+0.25\Phi M_{BbAa}(i-1)(1-\Phi)F_{BbAa}(i-1)+0.25(1-\Phi)M_{BbAa}(i-1)\Phi F_{BbAa}(i-1)\\
&+0.25(1-\Phi)M_{BbAa}(i-1)F_{BbAA}(i-1)+0.25(1-\Phi)M_{BbAa}(i-1)F_{BBaa}(i-1)\\
&+0.5\Phi M_{BbAa}(i-1)F_{BBaa}(i-1)+0.25(1-\Phi)M_{BbAa}(i-1)(1-\Phi)F_{BBAa}(i-1)\\
&+0.25\Phi M_{BbAa}(i-1)(1-\Phi)F_{BBAa}(i-1)+0.25(1-\Phi)M_{BbAa}(i-1)\Phi F_{BBAa}(i-1)\\
&+0.25(1-\Phi)M_{BbAa}(i-1)F_{BBAA}(i-1)+0.5M_{BbAA}(i-1)F_{bbaa}(i-1)+0.25M_{BbAA}(i-1)F_{bbAa}(i-1)\\
&+0.5M_{BbAA}(i-1)F_{Bbaa}(i-1)+0.25M_{BbAA}(i-1)(1-\Phi)F_{BbAa}(i-1)+0.5M_{BbAA}(i-1)F_{BBaa}(i-1)\\
&+0.25M_{BbAA}(i-1)(1-\Phi)F_{BBAa}(i-1)+0.5M_{BBaa}(i-1)F_{bbAa}(i-1)+M_{BBaa}(i-1)F_{bbAA}(i-1)\\
&+0.25M_{BBaa}(i-1)(1-\Phi)F_{BbAa}(i-1)+0.5M_{BBaa}(i-1)\Phi F_{BbAa}(i-1)+0.5M_{BBaa}(i-1)F_{BbAA}(i-1)\\
&+0.5(1-\Phi)M_{BBAa}(i-1)F_{bbaa}(i-1)+\Phi M_{BBAa}(i-1)F_{bbaa}(i-1)+0.5(1-\Phi)M_{BBAa}(i-1)F_{bbAa}(i-1)\\
&+0.5\Phi M_{BBAa}(i-1)F_{bbAa}(i-1)+0.5(1-\Phi)M_{BBAa}(i-1)F_{bbAA}(i-1)\\
&+0.25(1-\Phi)M_{BBAa}(i-1)F_{Bbaa}(i-1)+0.5\Phi M_{BBAa}(i-1)F_{Bbaa}(i-1)\\
&+0.25(1-\Phi)M_{BBAa}(i-1)(1-\Phi)F_{BbAa}(i-1)+0.25\Phi M_{BBAa}(i-1)(1-\Phi)F_{BbAa}(i-1)\\
&+0.25(1-\Phi)M_{BBAa}(i-1)\Phi F_{BbAa}(i-1)+0.25(1-\Phi)M_{BBAa}(i-1)F_{BbAA}(i-1)\\
&+M_{BBAA}(i-1)F_{bbaa}(i-1)+0.5M_{BBAA}(i-1)F_{bbAa}(i-1)+0.5M_{BBAA}(i-1)F_{Bbaa}(i-1)\\
&+0.25M_{BBAA}(i-1)(1-\Phi)F_{BbAa}(i-1)],
\end{aligned}
$$

$$
\begin{aligned}
M^e_{BbAA}=\left(\frac{\Omega_{BbAA}}{2}\right)&[0.125M_{bbAa}(i-1)(1-\Phi)F_{BbAa}(i-1)+0.25M_{bbAa}(i-1)\Phi F_{BbAa}(i-1)\\
&+0.25M_{bbAa}(i-1)F_{BbAA}(i-1)+0.25M_{bbAa}(i-1)(1-\Phi)F_{BBAa}(i-1)+0.5M_{bbAa}(i-1)\Phi F_{BBAa}(i-1)\\
&+0.5M_{bbAa}(i-1)F_{BBAA}(i-1)+0.25M_{bbAA}(i-1)(1-\Phi)F_{BbAa}(i-1)+0.5M_{bbAA}(i-1)\Phi F_{BbAa}(i-1)\\
&+0.5M_{bbAA}(i-1)F_{BbAA}(i-1)+0.5M_{bbAA}(i-1)(1-\Phi)F_{BBAa}(i-1)+M_{bbAA}(i-1)\Phi F_{BBAa}(i-1)\\
&+M_{bbAA}(i-1)F_{BBAA}(i-1)+0.125(1-\Phi)M_{BbAa}(i-1)F_{bbAa}(i-1)+0.25\Phi M_{BbAa}(i-1)F_{bbAa}(i-1)\\
&+0.25(1-\Phi)M_{BbAa}(i-1)F_{bbAA}(i-1)+0.5\Phi M_{BbAa}(i-1)F_{bbAA}(i-1)\\
&+0.125(1-\Phi)M_{BbAa}(i-1)(1-\Phi)F_{BbAa}(i-1)+0.25\Phi M_{BbAa}(i-1)(1-\Phi)F_{BbAa}(i-1)\\
&+0.25(1-\Phi)M_{BbAa}(i-1)\Phi F_{BbAa}(i-1)+0.5\Phi M_{BbAa}(i-1)\Phi F_{BbAa}(i-1)\\
&+0.25(1-\Phi)M_{BbAa}(i-1)F_{BbAA}(i-1)+0.5\Phi M_{BbAa}(i-1)F_{BbAA}(i-1)\\
&+0.125(1-\Phi)M_{BbAa}(i-1)(1-\Phi)F_{BBAa}(i-1)+0.25\Phi M_{BbAa}(i-1)(1-\Phi)F_{BBAa}(i-1)\\
&+0.25(1-\Phi)M_{BbAa}(i-1)\Phi F_{BBAa}(i-1)+0.5\Phi M_{BbAa}(i-1)\Phi F_{BBAa}(i-1)\\
&+0.25(1-\Phi)M_{BbAa}(i-1)F_{BBAA}(i-1)+0.5\Phi M_{BbAa}(i-1)F_{BBAA}(i-1)+0.25M_{BbAA}(i-1)F_{bbAa}(i-1)\\
&+0.5M_{BbAA}(i-1)F_{bbAA}(i-1)+0.25M_{BbAA}(i-1)(1-\Phi)F_{BbAa}(i-1)+0.5M_{BbAA}(i-1)\Phi F_{BbAa}(i-1)\\
&+0.5M_{BbAA}(i-1)F_{BbAA}(i-1)+0.25M_{BbAA}(i-1)(1-\Phi)F_{BBAa}(i-1)+0.5M_{BbAA}(i-1)\Phi F_{BBAa}(i-1)\\
&+0.5M_{BbAA}(i-1)F_{BBAA}(i-1)+0.25(1-\Phi)M_{BBAa}(i-1)F_{bbAa}(i-1)+0.5\Phi M_{BBAa}(i-1)F_{bbAa}(i-1)\\
&+0.5(1-\Phi)M_{BBAa}(i-1)F_{bbAA}(i-1)+\Phi M_{BBAa}(i-1)F_{bbAA}(i-1)\\
&+0.125(1-\Phi)M_{BBAa}(i-1)(1-\Phi)F_{BbAa}(i-1)+0.25\Phi M_{BBAa}(i-1)(1-\Phi)F_{BbAa}(i-1)\\
&+0.25(1-\Phi)M_{BBAa}(i-1)\Phi F_{BbAa}(i-1)+0.5\Phi M_{BBAa}(i-1)\Phi F_{BbAa}(i-1)\\
&+0.25(1-\Phi)M_{BBAa}(i-1)F_{BbAA}(i-1)+0.5\Phi M_{BBAa}(i-1)F_{BbAA}(i-1)+0.5M_{BBAA}(i-1)F_{bbAa}(i-1)\\
&+M_{BBAA}(i-1)F_{bbAA}(i-1)+0.25M_{BBAA}(i-1)(1-\Phi)F_{BbAa}(i-1)+0.5M_{BBAA}(i-1)\Phi F_{BbAa}(i-1)\\
&+0.5M_{BBAA}(i-1)F_{BbAA}(i-1)],
\end{aligned}
$$

$$
\begin{aligned}
M^e_{BBaa}=\left(\frac{\Omega_{BBaa}}{2}\right)&[0.25M_{Bbaa}(i-1)F_{Bbaa}(i-1)+0.125M_{Bbaa}(i-1)(1-\Phi)F_{BbAa}(i-1)\\
&+0.5M_{Bbaa}(i-1)F_{BBaa}(i-1)+0.25M_{Bbaa}(i-1)(1-\Phi)F_{BBAa}(i-1)\\
&+0.125(1-\Phi)M_{BbAa}(i-1)F_{Bbaa}(i-1)+0.0625(1-\Phi)M_{BbAa}(i-1)(1-\Phi)F_{BbAa}(i-1)\\
&+0.25(1-\Phi)M_{BbAa}(i-1)F_{BBaa}(i-1)+0.125(1-\Phi)M_{BbAa}(i-1)(1-\Phi)F_{BBAa}(i-1)\\
&+0.5M_{BBaa}(i-1)F_{Bbaa}(i-1)+0.25M_{BBaa}(i-1)(1-\Phi)F_{BbAa}(i-1)+M_{BBaa}(i-1)F_{BBaa}(i-1)
\end{aligned}
$$

$$
\begin{aligned}
&+ 0.5M_{BBaa}(i-1)(1-\Phi)F_{BBAa}(i-1) + 0.25(1-\Phi)M_{BBAa}(i-1)F_{Bbaa}(i-1) \\
&+ 0.125(1-\Phi)M_{BBAa}(i-1)(1-\Phi)F_{BbAa}(i-1) + 0.5(1-\Phi)M_{BBAa}(i-1)F_{BBaa}(i-1) \\
&+ 0.25(1-\Phi)M_{BBAa}(i-1)(1-\Phi)F_{BBAa}(i-1)],
\end{aligned}
$$

$$
\begin{aligned}
M^e_{BBAa} = \left(\frac{\Omega_{BBAa}}{2}\right) &[0.125M_{Bbaa}(i-1)(1-\Phi)F_{BbAa}(i-1) + 0.25M_{Bbaa}(i-1)\Phi F_{BbAa}(i-1) \\
&+ 0.25M_{Bbaa}(i-1)F_{BbAA}(i-1) + 0.25M_{Bbaa}(i-1)(1-\Phi)F_{BBAa}(i-1) + 0.5M_{Bbaa}(i-1)\Phi F_{BBAa}(i-1) \\
&+ 0.5M_{Bbaa}(i-1)F_{BBAA}(i-1) + 0.125(1-\Phi)M_{BbAa}(i-1)F_{Bbaa}(i-1) + 0.25\Phi M_{BbAa}(i-1)F_{Bbaa}(i-1) \\
&+ 0.125(1-\Phi)M_{BbAa}(i-1)(1-\Phi)F_{BbAa}(i-1) + 0.125\Phi M_{BbAa}(i-1)(1-\Phi)F_{BbAa}(i-1) \\
&+ 0.125(1-\Phi)M_{BbAa}(i-1)\Phi F_{BbAa}(i-1) + 0.125(1-\Phi)M_{BbAa}(i-1)F_{BbAA}(i-1) \\
&+ 0.25(1-\Phi)M_{BbAa}(i-1)F_{BBaa}(i-1) + 0.5\Phi M_{BbAa}(i-1)F_{BBaa}(i-1) \\
&+ 0.25(1-\Phi)M_{BbAa}(i-1)(1-\Phi)F_{BBAa}(i-1) + 0.25\Phi M_{BbAa}(i-1)(1-\Phi)F_{BBAa}(i-1) \\
&+ 0.125(1-\Phi)M_{BbAa}(i-1)\Phi F_{BBAa}(i-1) + 0.25(1-\Phi)M_{BbAa}(i-1)F_{BBAA}(i-1) \\
&+ 0.25M_{BbAA}(i-1)F_{Bbaa}(i-1) + 0.125M_{BbAA}(i-1)(1-\Phi)F_{BbAa}(i-1) + 0.5M_{BbAA}(i-1)F_{BBaa}(i-1) \\
&+ 0.25M_{BbAA}(i-1)(1-\Phi)F_{BBAa}(i-1) + 0.25M_{BBaa}(i-1)(1-\Phi)F_{BbAa}(i-1) \\
&+ 0.5M_{BBaa}(i-1)\Phi F_{BbAa}(i-1) + 0.5M_{BBaa}(i-1)F_{BbAA}(i-1) + 0.5M_{BBaa}(i-1)(1-\Phi)F_{BBAa}(i-1) \\
&+ M_{BBaa}(i-1)\Phi F_{BBAa}(i-1) + M_{BBaa}(i-1)F_{BBAA}(i-1) + 0.25(1-\Phi)M_{BBAa}(i-1)F_{Bbaa}(i-1) \\
&+ 0.5\Phi M_{BBAa}(i-1)F_{Bbaa}(i-1) + 0.25(1-\Phi)M_{BBAa}(i-1)(1-\Phi)F_{BbAa}(i-1) \\
&+ 0.25\Phi M_{BBAa}(i-1)(1-\Phi)F_{BbAa}(i-1) + 0.25(1-\Phi)M_{BBAa}(i-1)\Phi F_{BbAa}(i-1) \\
&+ 0.25(1-\Phi)M_{BBAa}(i-1)F_{BbAA}(i-1) + 0.5(1-\Phi)M_{BBAa}(i-1)F_{BBaa}(i-1) \\
&+ \Phi M_{BBAa}(i-1)F_{BBaa}(i-1) + 0.5(1-\Phi)M_{BBAa}(i-1)(1-\Phi)F_{BBAa}(i-1) \\
&+ 0.5\Phi M_{BBAa}(i-1)(1-\Phi)F_{BBAa}(i-1) + 0.5(1-\Phi)M_{BBAa}(i-1)\Phi F_{BBAa}(i-1) \\
&+ 0.5(1-\Phi)M_{BBAa}(i-1)F_{BBAA}(i-1) + 0.5M_{BBAA}(i-1)F_{Bbaa}(i-1) \\
&+ 0.25M_{BBAA}(i-1)(1-\Phi)F_{BbAa}(i-1) + M_{BBAA}(i-1)F_{BBaa}(i-1) \\
&+ 0.5M_{BBAA}(i-1)(1-\Phi)F_{BBAa}(i-1)],
\end{aligned}
$$

$$
\begin{aligned}
M^e_{BBAA} = \left(\frac{\Omega_{BBAA}}{2}\right) &[0.0625(1-\Phi)M_{BbAa}(i-1)(1-\Phi)F_{BbAa}(i-1) + 0.125\Phi M_{BbAa}(i-1)(1-\Phi)F_{BbAa}(i-1) \\
&+ 0.125(1-\Phi)M_{BbAa}(i-1)\Phi F_{BbAa}(i-1) + 0.25\Phi M_{BbAa}(i-1)\Phi F_{BbAa}(i-1) \\
&+ 0.125(1-\Phi)M_{BbAa}(i-1)F_{BbAA}(i-1) + 0.25\Phi M_{BbAa}(i-1)F_{BbAA}(i-1) \\
&+ 0.125(1-\Phi)M_{BbAa}(i-1)(1-\Phi)F_{BBAa}(i-1) + 0.25\Phi M_{BbAa}(i-1)(1-\Phi)F_{BBAa}(i-1) \\
&+ 0.125(1-\Phi)M_{BbAa}(i-1)\Phi F_{BBAa}(i-1) + 0.5\Phi M_{BbAa}(i-1)\Phi F_{BBAa}(i-1) \\
&+ 0.25(1-\Phi)M_{BbAa}(i-1)F_{BBAA}(i-1) + 0.5\Phi M_{BbAa}(i-1)F_{BBAA}(i-1) \\
&+ 0.125M_{BbAA}(i-1)(1-\Phi)F_{BbAa}(i-1) + 0.25M_{BbAA}(i-1)\Phi F_{BbAa}(i-1) \\
&+ 0.25M_{BbAA}(i-1)F_{BbAA}(i-1) + 0.25M_{BbAA}(i-1)(1-\Phi)F_{BBAa}(i-1) \\
&+ 0.5M_{BbAA}(i-1)\Phi F_{BBAa}(i-1) + 0.5M_{BbAA}(i-1)F_{BBAA}(i-1) \\
&+ 0.125(1-\Phi)M_{BBAa}(i-1)(1-\Phi)F_{BbAa}(i-1) + 0.25\Phi M_{BBAa}(i-1)(1-\Phi)F_{BbAa}(i-1) \\
&+ 0.25(1-\Phi)M_{BBAa}(i-1)\Phi F_{BbAa}(i-1) + 0.5\Phi M_{BBAa}(i-1)\Phi F_{BbAa}(i-1) \\
&+ 0.25(1-\Phi)M_{BBAa}(i-1)F_{BbAA}(i-1) + 0.5\Phi M_{BBAa}(i-1)F_{BbAA}(i-1) \\
&+ 0.25(1-\Phi)M_{BBAa}(i-1)(1-\Phi)F_{BBAa}(i-1) + 0.5\Phi M_{BBAa}(i-1)(1-\Phi)F_{BBAa}(i-1) \\
&+ 0.5(1-\Phi)M_{BBAa}(i-1)\Phi F_{BBAa}(i-1) + \Phi M_{BBAa}(i-1)\Phi F_{BBAa}(i-1) \\
&+ 0.5(1-\Phi)M_{BBAa}(i-1)F_{BBAA}(i-1) + \Phi M_{BBAa}(i-1)F_{BBAA}(i-1) \\
&+ 0.25M_{BBAA}(i-1)(1-\Phi)F_{BbAa}(i-1) + 0.5M_{BBAA}(i-1)\Phi F_{BbAa}(i-1) + 0.5M_{BBAA}(i-1)F_{BbAA}(i-1) \\
&+ 0.5M_{BBAA}(i-1)(1-\Phi)F_{BBAa}(i-1) + M_{BBAA}(i-1)\Phi F_{BBAa}(i-1) + M_{BBAA}(i-1)F_{BBAA}(i-1)],
\end{aligned}
$$

alongside an identical set of equations for females of each genotype.

The second stage is to normalise these proportional frequencies such that they fill the entire range from zero to one. To do this we begin by calculating the overall fitness of the entire population by summing

the proportional genotype frequencies resulting from the above equations to give:

$$\bar{\Omega} = M^e_{bbaa} + M^e_{bbAa} + M^e_{bbAAE} + M^e_{Bbaa} + M^e_{BbAa} + M^e_{BbAA} + M^e_{BBaa} + M^e_{BBAa} + M^e_{BBAA} + F^e_{bbaa} + F^e_{bbAa} + F^e_{bbAA} + F^e_{Bbaa} + F^e_{BbAa} + F^e_{BbAA} + F^e_{BBaa} + F^e_{BBAa} + F^e_{BBAA}.$$

This is then used as a normalising factor to give the final genotype frequencies for a particular generation as follows:

$$M_{bbaa}(i) = M^e_{bbaa}/\bar{\Omega}, \quad M_{bbAa}(i) = M^e_{bbAa}/\bar{\Omega}, \quad M_{bbAA}(i) = M^e_{bbAA}/\bar{\Omega},$$
$$M_{Bbaa}(i) = M^e_{Bbaa}/\bar{\Omega}, \quad M_{BbAa}(i) = M^e_{BbAa}/\bar{\Omega}, \quad M_{BbAA}(i) = M^e_{BbAA}/\bar{\Omega},$$
$$M_{BBaa}(i) = M^e_{BBaa}/\bar{\Omega}, \quad M_{BBAa}(i) = M^e_{BBAa}/\bar{\Omega}, \quad M_{BBAA}(i) = M^e_{BBAA}/\bar{\Omega},$$
$$F_{bbaa}(i) = F^e_{bbaa}/\bar{\Omega}, \quad F_{bbAa}(i) = F^e_{bbAa}/\bar{\Omega}, \quad F_{bbAA}(i) = F^e_{bbAA}/\bar{\Omega},$$
$$F_{Bbaa}(i) = F^e_{Bbaa}/\bar{\Omega}, \quad F_{BbAa}(i) = F^e_{BbAa}/\bar{\Omega}, \quad F_{BbAA}(i) = F^e_{BbAA}/\bar{\Omega},$$
$$F_{BBaa}(i) = F^e_{BBaa}/\bar{\Omega}, \quad F_{BBAa}(i) = F^e_{BBAa}/\bar{\Omega}, \quad F_{BBAA}(i) = F^e_{BBAA}/\bar{\Omega}.$$

We then insert these values into the proportional genotype frequency equations in order to calculate the values for the next generation and repeat this process until the desired end point is reached.
