# Supplementary material for: Split drive killer-rescue provides a novel threshold-dependent gene drive
Source: Sci Rep. 2020 Nov 25;10:20520. doi: 10.1038/s41598-020-77544-7 (PMC7689494; doi:10.1038/s41598-020-77544-7)
Supplement: Supplementary file 2 — Supplementary material 2 [file 41598_2020_77544_MOESM2_ESM.pdf]

Split drive killer-rescue provides a novel threshold-dependent gene drive

Matthew P. Edgington[1*], Tim Harvey-Samuel[1] and Luke Alphey[1]

[1]The Pirbright Institute, Ash Road, Woking, Surrey GU24 0NF, UK
*Corresponding author: matt.edgington@pirbright.ac.uk

Supplementary Model 2

We begin here by defining a set of parameter values for use throughout this study. This is as follows:

- $L = 1$ (full lethal penetrance),
- $\varepsilon_A = 0.85$ (relative to 1 in wild-type individuals - 15% fitness cost),
- $\varepsilon_B = 0.85$ (relative to 1 in wild-type individuals - 15% fitness cost),
- $\varepsilon_R = 1$ (resistant alleles give no fitness cost),
- $C = 0.9$ (90% of eligible wild-type sequences are cleaved),
- $\Phi = 0.9C$ (90% of cleaved sequences are repaired via homing of transgenic constructs),
- $\eta = 0.1C$ (10% of cleaved sequences are repaired via end-joining producing resistant alleles).

The relative fitness parameters defined above are combined multiplicatively to obtain a single overall relative fitness value for each genotype (assumed equal for both sexes). These are of the form:

$$\Omega_{bbaa}1, \quad \Omega_{bbAa} = (1-L)\varepsilon_A, \quad \Omega_{bbRa} = \varepsilon_R, \quad \Omega_{bbAA} = (1-L)\varepsilon_A^2, \quad \Omega_{bbAR} = (1-L)\varepsilon_A\varepsilon_R, \quad \Omega_{bbRR} = \varepsilon_R^2,$$

$$\Omega_{Bbaa} = \varepsilon_B, \quad \Omega_{BbAa} = \varepsilon_B\varepsilon_A, \quad \Omega_{BbRa} = \varepsilon_B\varepsilon_R, \quad \Omega_{BbAA} = \varepsilon_B\varepsilon_A^2, \quad \Omega_{BbAR} = \varepsilon_B\varepsilon_A\varepsilon_R, \quad \Omega_{BbRR} = \varepsilon_B\varepsilon_R^2,$$

$$\Omega_{BBaa} = \varepsilon_B^2, \quad \Omega_{BBAa} = \varepsilon_B^2\varepsilon_A, \quad \Omega_{BBRa} = \varepsilon_B^2\varepsilon_R, \quad \Omega_{BBAA} = \varepsilon_B^2\varepsilon_A^2, \quad \Omega_{BBAR} = \varepsilon_B^2\varepsilon_A\varepsilon_R, \quad \Omega_{BBRR} = \varepsilon_B^2\varepsilon_R^2.$$

We then define a set of initial conditions representing the genotype proportions in the population at the point of introducing transgenics into the wild population. For a 1:1 (introduced:wild) release of individuals homozygous for both transgenic constructs, as is most widely considered in this study these are of the form:

$$M_{bbaa}(1) = 0.25 = F_{bbaa}(1), \quad M_{bbAa}(1) = 0.00 = F_{bbAa}(1), \quad M_{bbRa}(1) = 0.00 = F_{bbRa}(1),$$

$$M_{bbAA}(1) = 0.00 = F_{bbAA}(1), \quad M_{bbAR}(1) = 0.00 = F_{bbAR}(1), \quad M_{bbRR}(1) = 0.00 = F_{bbRR}(1),$$

$$M_{Bbaa}(1) = 0.00 = F_{Bbaa}(1), \quad M_{BbAa}(1) = 0.00 = F_{BbAa}(1), \quad M_{BbRa}(1) = 0.00 = F_{BbRa}(1),$$

$$M_{BbAA}(1) = 0.00 = F_{BbAA}(1), \quad M_{BbAR}(1) = 0.00 = F_{BbAR}(1), \quad M_{BbRR}(1) = 0.00 = F_{BbRR}(1),$$

$$M_{BBaa}(1) = 0.00 = F_{BBaa}(1), \quad M_{BBAa}(1) = 0.00 = F_{BBAa}(1), \quad M_{BBRa}(1) = 0.00 = F_{BBRa}(1),$$

$$M_{BBAA}(1) = 0.25 = F_{BBAA}(1), \quad M_{BBAR}(1) = 0.00 = F_{BBAR}(1), \quad M_{BBRR}(1) = 0.00 = F_{BBRR}(1).$$

With the above definitions we are then able to iteratively calculate the genotype frequencies in each subsequent generation. This process is conducted in a two step manner. The first stage is to calculate the proportional frequencies for each genotype (and for each sex), which is achieved using:

$$M^e_{bbaa} = \left(\frac{\Omega_{bbaa}}{2}\right)[M_{bbaa}(i-1)F_{bbaa}(i-1) + 0.5M_{bbaa}(i-1)F_{bbAa}(i-1) + 0.5M_{bbaa}(i-1)F_{bbRa}(i-1)$$
$$+ 0.5M_{bbaa}(i-1)F_{Bbaa}(i-1) + 0.25M_{bbaa}(i-1)(1-\Phi-\eta)F_{BbAa}(i-1) + 0.25M_{bbaa}(i-1)F_{BbRa}(i-1)$$
$$+ 0.5M_{bbAa}(i-1)F_{bbaa}(i-1) + 0.25M_{bbAa}(i-1)F_{bbAa}(i-1) + 0.25M_{bbAa}(i-1)F_{bbRa}(i-1)$$

$$+ 0.25M_{bbAa}(i-1)F_{Bbaa}(i-1) + 0.125M_{bbAa}(i-1)(1-\Phi-\eta)F_{BbAa}(i-1)$$
$$+ 0.125M_{bbAa}(i-1)F_{BbRa}(i-1) + 0.5M_{bbRa}(i-1)F_{bbaa}(i-1) + 0.25M_{bbRa}(i-1)F_{bbAa}(i-1)$$
$$+ 0.25M_{bbRa}(i-1)F_{bbRa}(i-1) + 0.25M_{bbRa}(i-1)F_{Bbaa}(i-1) + 0.125M_{bbRa}(i-1)(1-\Phi-\eta)F_{BbAa}(i-1)$$
$$+ 0.125M_{bbRa}(i-1)F_{BbRa}(i-1) + 0.5M_{Bbaa}(i-1)F_{bbaa}(i-1) + 0.25M_{Bbaa}(i-1)F_{bbAa}(i-1)$$
$$+ 0.25M_{Bbaa}(i-1)F_{bbRa}(i-1) + 0.25M_{Bbaa}(i-1)F_{Bbaa}(i-1) + 0.125M_{Bbaa}(i-1)(1-\Phi-\eta)F_{BbAa}(i-1)$$
$$+ 0.125M_{Bbaa}(i-1)F_{BbRa}(i-1) + 0.25(1-\Phi-\eta)M_{BbAa}(i-1)F_{bbaa}(i-1)$$
$$+ 0.125(1-\Phi-\eta)M_{BbAa}(i-1)F_{bbAa}(i-1) + 0.125(1-\Phi-\eta)M_{BbAa}(i-1)F_{bbRa}(i-1)$$
$$+ 0.125(1-\Phi-\eta)M_{BbAa}(i-1)F_{Bbaa}(i-1) + 0.0625(1-\Phi-\eta)M_{BbAa}(i-1)(1-\Phi-\eta)F_{BbAa}(i-1)$$
$$+ 0.0625(1-\Phi-\eta)M_{BbAa}(i-1)F_{BbRa}(i-1) + 0.25M_{BbRa}(i-1)F_{bbaa}(i-1)$$
$$+ 0.125M_{BbRa}(i-1)F_{bbAa}(i-1) + 0.125M_{BbRa}(i-1)F_{bbRa}(i-1) + 0.125M_{BbRa}(i-1)F_{Bbaa}(i-1)$$
$$+ 0.0625M_{BbRa}(i-1)(1-\Phi-\eta)F_{BbAa}(i-1) + 0.0625M_{BbRa}(i-1)F_{BbRa}(i-1)],$$
$$M^e_{bbAa} = \left(\frac{\Omega_{bbAa}}{2}\right)[0.5M_{bbaa}(i-1)F_{bbAa}(i-1) + M_{bbaa}(i-1)F_{bbAA}(i-1) + 0.5M_{bbaa}(i-1)F_{bbAR}(i-1)$$
$$+ 0.25M_{bbaa}(i-1)(1-\Phi-\eta)F_{BbAa}(i-1) + 0.5M_{bbaa}(i-1)\Phi F_{BbAa}(i-1) + 0.25M_{bbaa}(i-1)\eta F_{BbAa}(i-1)$$
$$+ 0.5M_{bbaa}(i-1)F_{BbAA}(i-1) + 0.25M_{bbaa}(i-1)F_{BbAR}(i-1) + 0.5M_{bbAa}(i-1)F_{bbaa}(i-1)$$
$$+ 0.5M_{bbAa}(i-1)F_{bbAa}(i-1) + 0.25M_{bbAa}(i-1)F_{bbRa}(i-1) + 0.5M_{bbAa}(i-1)F_{bbAA}(i-1)$$
$$+ 0.25M_{bbAa}(i-1)F_{bbAR}(i-1) + 0.25M_{bbAa}(i-1)F_{Bbaa}(i-1) + 0.25M_{bbAa}(i-1)(1-\Phi-\eta)F_{BbAa}(i-1)$$
$$+ 0.25M_{bbAa}(i-1)\Phi F_{BbAa}(i-1) + 0.125M_{bbAa}(i-1)\eta F_{BbAa}(i-1) + 0.125M_{bbAa}(i-1)F_{BbRa}(i-1)$$
$$+ 0.25M_{bbAa}(i-1)F_{BbAA}(i-1) + 0.125M_{bbAa}(i-1)F_{BbAR}(i-1) + 0.25M_{bbRa}(i-1)F_{bbAa}(i-1)$$
$$+ 0.5M_{bbRa}(i-1)F_{bbAA}(i-1) + 0.25M_{bbRa}(i-1)F_{bbAR}(i-1) + 0.125M_{bbRa}(i-1)(1-\Phi-\eta)F_{BbAa}(i-1)$$
$$+ 0.25M_{bbRa}(i-1)\Phi F_{BbAa}(i-1) + 0.125M_{bbRa}(i-1)\eta F_{BbAa}(i-1) + 0.25M_{bbRa}(i-1)F_{BbAA}(i-1)$$
$$+ 0.125M_{bbRa}(i-1)F_{BbAR}(i-1) + M_{bbAA}(i-1)F_{bbaa}(i-1) + 0.5M_{bbAA}(i-1)F_{bbAa}(i-1)$$
$$+ 0.5M_{bbAA}(i-1)F_{bbRa}(i-1) + 0.5M_{bbAA}(i-1)F_{Bbaa}(i-1) + 0.25M_{bbAA}(i-1)(1-\Phi-\eta)F_{BbAa}(i-1)$$
$$+ 0.25M_{bbAA}(i-1)F_{BbRa}(i-1) + 0.5M_{bbAR}(i-1)F_{bbaa}(i-1) + 0.25M_{bbAR}(i-1)F_{bbAa}(i-1)$$
$$+ 0.25M_{bbAR}(i-1)F_{bbRa}(i-1) + 0.25M_{bbAR}(i-1)F_{Bbaa}(i-1) + 0.125M_{bbAR}(i-1)(1-\Phi-\eta)F_{BbAa}(i-1)$$
$$+ 0.125M_{bbAR}(i-1)F_{BbRa}(i-1) + 0.25M_{Bbaa}(i-1)F_{bbAa}(i-1) + 0.5M_{Bbaa}(i-1)F_{bbAA}(i-1)$$
$$+ 0.25M_{Bbaa}(i-1)F_{bbAR}(i-1) + 0.125M_{Bbaa}(i-1)(1-\Phi-\eta)F_{BbAa}(i-1)$$
$$+ 0.25M_{Bbaa}(i-1)\Phi F_{BbAa}(i-1) + 0.125M_{Bbaa}(i-1)\eta F_{BbAa}(i-1) + 0.25M_{Bbaa}(i-1)F_{BbAA}(i-1)$$
$$+ 0.125M_{Bbaa}(i-1)F_{BbAR}(i-1) + 0.25(1-\Phi-\eta)M_{BbAa}(i-1)F_{bbaa}(i-1) + 0.5\Phi M_{BbAa}(i-1)F_{bbaa}(i-1)$$
$$+ 0.25\eta M_{BbAa}(i-1)F_{bbaa}(i-1) + 0.25(1-\Phi-\eta)M_{BbAa}(i-1)F_{bbAa}(i-1) + 0.25\Phi M_{BbAa}(i-1)F_{bbAa}(i-1)$$
$$+ 0.125\eta M_{BbAa}(i-1)F_{bbAa}(i-1) + 0.125(1-\Phi-\eta)M_{BbAa}(i-1)F_{bbRa}(i-1)$$
$$+ 0.25\Phi M_{BbAa}(i-1)F_{bbRa}(i-1) + 0.125\eta M_{BbAa}(i-1)F_{bbRa}(i-1)$$
$$+ 0.25(1-\Phi-\eta)M_{BbAa}(i-1)F_{bbAA}(i-1) + 0.125(1-\Phi-\eta)M_{BbAa}(i-1)F_{bbAR}(i-1)$$
$$+ 0.125(1-\Phi-\eta)M_{BbAa}(i-1)F_{Bbaa}(i-1) + 0.25\Phi M_{BbAa}(i-1)F_{Bbaa}(i-1)$$
$$+ 0.125\eta M_{BbAa}(i-1)F_{Bbaa}(i-1) + 0.125(1-\Phi-\eta)M_{BbAa}(i-1)(1-\Phi-\eta)F_{BbAa}(i-1)$$
$$+ 0.125\Phi M_{BbAa}(i-1)(1-\Phi-\eta)F_{BbAa}(i-1) + 0.0625\eta M_{BbAa}(i-1)(1-\Phi-\eta)F_{BbAa}(i-1)$$
$$+ 0.125(1-\Phi-\eta)M_{BbAa}(i-1)\Phi F_{BbAa}(i-1) + 0.0625(1-\Phi-\eta)M_{BbAa}(i-1)\eta F_{BbAa}(i-1)$$
$$+ 0.0625(1-\Phi-\eta)M_{BbAa}(i-1)F_{BbRa}(i-1) + 0.125\Phi M_{BbAa}(i-1)F_{BbRa}(i-1)$$
$$+ 0.0625\eta M_{BbAa}(i-1)F_{BbRa}(i-1) + 0.125(1-\Phi-\eta)M_{BbAa}(i-1)F_{BbAA}(i-1)$$
$$+ 0.0625(1-\Phi-\eta)M_{BbAa}(i-1)F_{BbAR}(i-1) + 0.125(1-\Phi-\eta)M_{BbAa}(i-1)\Phi F_{BBAa}(i-1)$$
$$+ 0.0625(1-\Phi-\eta)M_{BbAa}(i-1)\eta F_{BBAa}(i-1) + 0.125M_{BbRa}(i-1)F_{bbAa}(i-1)$$
$$+ 0.25M_{BbRa}(i-1)F_{bbAA}(i-1) + 0.125M_{BbRa}(i-1)F_{bbAR}(i-1)$$
$$+ 0.0625M_{BbRa}(i-1)(1-\Phi-\eta)F_{BbAa}(i-1) + 0.125M_{BbRa}(i-1)\Phi F_{BbAa}(i-1)$$
$$+ 0.0625M_{BbRa}(i-1)\eta F_{BbAa}(i-1) + 0.125M_{BbRa}(i-1)F_{BbAA}(i-1) + 0.0625M_{BbRa}(i-1)F_{BbAR}(i-1)$$
$$+ 0.5M_{BbAA}(i-1)F_{bbaa}(i-1) + 0.25M_{BbAA}(i-1)F_{bbAa}(i-1) + 0.25M_{BbAA}(i-1)F_{bbRa}(i-1)$$
$$+ 0.25M_{BbAA}(i-1)F_{Bbaa}(i-1) + 0.125M_{BbAA}(i-1)(1-\Phi-\eta)F_{BbAa}(i-1)$$
$$+ 0.125M_{BbAA}(i-1)F_{BbRa}(i-1) + 0.25M_{BbAR}(i-1)F_{bbaa}(i-1) + 0.125M_{BbAR}(i-1)F_{bbAa}(i-1)$$

$$
\begin{aligned}
&+ 0.125M_{BbAR}(i-1)F_{bbRa}(i-1) + 0.125M_{BbAR}(i-1)F_{Bbaa}(i-1)\\
&+ 0.0625M_{BbAR}(i-1)(1-\Phi-\eta)F_{BbAa}(i-1) + 0.0625M_{BbAR}(i-1)F_{BbRa}(i-1)],
\end{aligned}
$$

$$
\begin{aligned}
M^e_{bbRa} = \left(\frac{\Omega_{bbRa}}{2}\right) &[0.5M_{bbaa}(i-1)F_{bbRa}(i-1) + 0.5M_{bbaa}(i-1)F_{bbAR}(i-1) + M_{bbaa}(i-1)F_{bbRR}(i-1)\\
&+ 0.25M_{bbaa}(i-1)\eta F_{BbAa}(i-1) + 0.25M_{bbaa}(i-1)F_{BbRa}(i-1) + 0.25M_{bbaa}(i-1)F_{BbAR}(i-1)\\
&+ 0.5M_{bbaa}(i-1)F_{BbRR}(i-1) + 0.25M_{bbAa}(i-1)F_{bbRa}(i-1) + 0.25M_{bbAa}(i-1)F_{bbAR}(i-1)\\
&+ 0.5M_{bbAa}(i-1)F_{bbRR}(i-1) + 0.125M_{bbAa}(i-1)\eta F_{BbAa}(i-1) + 0.125M_{bbAa}(i-1)F_{BbRa}(i-1)\\
&+ 0.125M_{bbAa}(i-1)F_{BbAR}(i-1) + 0.25M_{bbAa}(i-1)F_{BbRR}(i-1) + 0.5M_{bbRa}(i-1)F_{bbaa}(i-1)\\
&+ 0.25M_{bbRa}(i-1)F_{bbAa}(i-1) + 0.5M_{bbRa}(i-1)F_{bbRa}(i-1) + 0.25M_{bbRa}(i-1)F_{bbAR}(i-1)\\
&+ 0.5M_{bbRa}(i-1)F_{bbRR}(i-1) + 0.25M_{bbRa}(i-1)F_{Bbaa}(i-1) + 0.125M_{bbRa}(i-1)(1-\Phi-\eta)F_{BbAa}(i-1)\\
&+ 0.125M_{bbRa}(i-1)\eta F_{BbAa}(i-1) + 0.25M_{bbRa}(i-1)F_{BbRa}(i-1) + 0.125M_{bbRa}(i-1)F_{BbAR}(i-1)\\
&+ 0.25M_{bbRa}(i-1)F_{BbRR}(i-1) + 0.5M_{bbAR}(i-1)F_{bbaa}(i-1) + 0.25M_{bbAR}(i-1)F_{bbAa}(i-1)\\
&+ 0.25M_{bbAR}(i-1)F_{bbRa}(i-1) + 0.25M_{bbAR}(i-1)F_{Bbaa}(i-1) + 0.125M_{bbAR}(i-1)(1-\Phi-\eta)F_{BbAa}(i-1)\\
&+ 0.125M_{bbAR}(i-1)F_{BbRa}(i-1) + M_{bbRR}(i-1)F_{bbaa}(i-1) + 0.5M_{bbRR}(i-1)F_{bbAa}(i-1)\\
&+ 0.5M_{bbRR}(i-1)F_{bbRa}(i-1) + 0.5M_{bbRR}(i-1)F_{Bbaa}(i-1) + 0.25M_{bbRR}(i-1)(1-\Phi-\eta)F_{BbAa}(i-1)\\
&+ 0.25M_{bbRR}(i-1)F_{BbRa}(i-1) + 0.25M_{Bbaa}(i-1)F_{bbRa}(i-1) + 0.25M_{Bbaa}(i-1)F_{bbAR}(i-1)\\
&+ 0.5M_{Bbaa}(i-1)F_{bbRR}(i-1) + 0.125M_{Bbaa}(i-1)\eta F_{BbAa}(i-1) + 0.125M_{Bbaa}(i-1)F_{BbRa}(i-1)\\
&+ 0.125M_{Bbaa}(i-1)F_{BbAR}(i-1) + 0.25M_{Bbaa}(i-1)F_{BbRR}(i-1) + 0.25\eta M_{BbAa}(i-1)F_{bbaa}(i-1)\\
&+ 0.125\eta M_{BbAa}(i-1)F_{bbAa}(i-1) + 0.125(1-\Phi-\eta)M_{BbAa}(i-1)F_{bbRa}(i-1)\\
&+ 0.125\eta M_{BbAa}(i-1)F_{bbRa}(i-1) + 0.125(1-\Phi-\eta)M_{BbAa}(i-1)F_{bbAR}(i-1)\\
&+ 0.25(1-\Phi-\eta)M_{BbAa}(i-1)F_{bbRR}(i-1) + 0.125\eta M_{BbAa}(i-1)F_{Bbaa}(i-1)\\
&+ 0.0625\eta M_{BbAa}(i-1)(1-\Phi-\eta)F_{BbAa}(i-1) + 0.0625(1-\Phi-\eta)M_{BbAa}(i-1)\eta F_{BbAa}(i-1)\\
&+ 0.0625(1-\Phi-\eta)M_{BbAa}(i-1)F_{BbRa}(i-1) + 0.0625\eta M_{BbAa}(i-1)F_{BbRa}(i-1)\\
&+ 0.0625(1-\Phi-\eta)M_{BbAa}(i-1)F_{BbAR}(i-1) + 0.125(1-\Phi-\eta)M_{BbAa}(i-1)F_{BbRR}(i-1)\\
&+ 0.0625(1-\Phi-\eta)M_{BbAa}(i-1)\eta F_{BBAa}(i-1) + 0.25M_{BbRa}(i-1)F_{bbaa}(i-1)\\
&+ 0.125M_{BbRa}(i-1)F_{bbAa}(i-1) + 0.25M_{BbRa}(i-1)F_{bbRa}(i-1) + 0.125M_{BbRa}(i-1)F_{bbAR}(i-1)\\
&+ 0.25M_{BbRa}(i-1)F_{bbRR}(i-1) + 0.125M_{BbRa}(i-1)F_{Bbaa}(i-1)\\
&+ 0.0625M_{BbRa}(i-1)(1-\Phi-\eta)F_{BbAa}(i-1) + 0.0625M_{BbRa}(i-1)\eta F_{BbAa}(i-1)\\
&+ 0.125M_{BbRa}(i-1)F_{BbRa}(i-1) + 0.0625M_{BbRa}(i-1)F_{BbAR}(i-1) + 0.125M_{BbRa}(i-1)F_{BbRR}(i-1)\\
&+ 0.25M_{BbAR}(i-1)F_{bbaa}(i-1) + 0.125M_{BbAR}(i-1)F_{bbAa}(i-1) + 0.125M_{BbAR}(i-1)F_{bbRa}(i-1)\\
&+ 0.125M_{BbAR}(i-1)F_{Bbaa}(i-1) + 0.0625M_{BbAR}(i-1)(1-\Phi-\eta)F_{BbAa}(i-1)\\
&+ 0.0625M_{BbAR}(i-1)F_{BbRa}(i-1) + 0.5M_{BbRR}(i-1)F_{bbaa}(i-1) + 0.25M_{BbRR}(i-1)F_{bbAa}(i-1)\\
&+ 0.25M_{BbRR}(i-1)F_{bbRa}(i-1) + 0.25M_{BbRR}(i-1)F_{Bbaa}(i-1)\\
&+ 0.125M_{BbRR}(i-1)(1-\Phi-\eta)F_{BbAa}(i-1) + 0.125M_{BbRR}(i-1)F_{BbRa}(i-1)],
\end{aligned}
$$

$$
\begin{aligned}
M^e_{bbAA} = \left(\frac{\Omega_{bbAA}}{2}\right) &[0.25M_{bbAa}(i-1)F_{bbAa}(i-1) + 0.5M_{bbAa}(i-1)F_{bbAA}(i-1) + 0.25M_{bbAa}(i-1)F_{bbAR}(i-1)\\
&+ 0.125M_{bbAa}(i-1)(1-\Phi-\eta)F_{BbAa}(i-1) + 0.25M_{bbAa}(i-1)\Phi F_{BbAa}(i-1)\\
&+ 0.125M_{bbAa}(i-1)\eta F_{BbAa}(i-1) + 0.25M_{bbAa}(i-1)F_{BbAA}(i-1) + 0.125M_{bbAa}(i-1)F_{BbAR}(i-1)\\
&+ 0.5M_{bbAA}(i-1)F_{bbAa}(i-1) + M_{bbAA}(i-1)F_{bbAA}(i-1) + 0.5M_{bbAA}(i-1)F_{bbAR}(i-1)\\
&+ 0.25M_{bbAA}(i-1)(1-\Phi-\eta)F_{BbAa}(i-1) + 0.5M_{bbAA}(i-1)\Phi F_{BbAa}(i-1)\\
&+ 0.25M_{bbAA}(i-1)\eta F_{BbAa}(i-1) + 0.5M_{bbAA}(i-1)F_{BbAA}(i-1) + 0.25M_{bbAA}(i-1)F_{BbAR}(i-1)\\
&+ 0.25M_{bbAR}(i-1)F_{bbAa}(i-1) + 0.5M_{bbAR}(i-1)F_{bbAA}(i-1) + 0.25M_{bbAR}(i-1)F_{bbAR}(i-1)\\
&+ 0.125M_{bbAR}(i-1)(1-\Phi-\eta)F_{BbAa}(i-1) + 0.25M_{bbAR}(i-1)\Phi F_{BbAa}(i-1)\\
&+ 0.125M_{bbAR}(i-1)\eta F_{BbAa}(i-1) + 0.25M_{bbAR}(i-1)F_{BbAA}(i-1) + 0.125M_{bbAR}(i-1)F_{BbAR}(i-1)\\
&+ 0.125(1-\Phi-\eta)M_{BbAa}(i-1)F_{bbAa}(i-1) + 0.25\Phi M_{BbAa}(i-1)F_{bbAa}(i-1)\\
&+ 0.125\eta M_{BbAa}(i-1)F_{bbAa}(i-1) + 0.25(1-\Phi-\eta)M_{BbAa}(i-1)F_{bbAA}(i-1)
\end{aligned}
$$

$$
\begin{aligned}
&+ 0.5\Phi M_{BbAa}(i-1)F_{bbAA}(i-1) + 0.25\eta M_{BbAa}(i-1)F_{bbAA}(i-1)\\
&+ 0.125(1-\Phi-\eta)M_{BbAa}(i-1)F_{bbAR}(i-1) + 0.25\Phi M_{BbAa}(i-1)F_{bbAR}(i-1)\\
&+ 0.125\eta M_{BbAa}(i-1)F_{bbAR}(i-1) + 0.0625(1-\Phi-\eta)M_{BbAa}(i-1)(1-\Phi-\eta)F_{BbAa}(i-1)\\
&+ 0.125\Phi M_{BbAa}(i-1)(1-\Phi-\eta)F_{BbAa}(i-1) + 0.0625\eta M_{BbAa}(i-1)(1-\Phi-\eta)F_{BbAa}(i-1)\\
&+ 0.125(1-\Phi-\eta)M_{BbAa}(i-1)\Phi F_{BbAa}(i-1) + 0.0625(1-\Phi-\eta)M_{BbAa}(i-1)\eta F_{BbAa}(i-1)\\
&+ 0.25\Phi M_{BbAa}(i-1)\Phi F_{BbAa}(i-1) + 0.125\Phi M_{BbAa}(i-1)\eta F_{BbAa}(i-1) + 0.125\eta M_{BbAa}(i-1)\Phi F_{BbAa}(i-1)\\
&+ 0.0625\eta M_{BbAa}(i-1)\eta F_{BbAa}(i-1) + 0.125(1-\Phi-\eta)M_{BbAa}(i-1)F_{BbAA}(i-1)\\
&+ 0.25\Phi M_{BbAa}(i-1)F_{BbAA}(i-1) + 0.125\eta M_{BbAa}(i-1)F_{BbAA}(i-1)\\
&+ 0.0625(1-\Phi-\eta)M_{BbAa}(i-1)F_{BbAR}(i-1) + 0.125\Phi M_{BbAa}(i-1)F_{BbAR}(i-1)\\
&+ 0.0625\eta M_{BbAa}(i-1)F_{BbAR}(i-1) + 0.125(1-\Phi-\eta)M_{BbAa}(i-1)\Phi F_{BBAa}(i-1)\\
&+ 0.0625(1-\Phi-\eta)M_{BbAa}(i-1)\eta F_{BBAa}(i-1) + 0.25M_{BbAA}(i-1)F_{bbAa}(i-1)\\
&+ 0.5M_{BbAA}(i-1)F_{bbAA}(i-1) + 0.25M_{BbAA}(i-1)F_{bbAR}(i-1)\\
&+ 0.125M_{BbAA}(i-1)(1-\Phi-\eta)F_{BbAa}(i-1) + 0.25M_{BbAA}(i-1)\Phi F_{BbAa}(i-1)\\
&+ 0.125M_{BbAA}(i-1)\eta F_{BbAa}(i-1) + 0.25M_{BbAA}(i-1)F_{BbAA}(i-1) + 0.125M_{BbAA}(i-1)F_{BbAR}(i-1)\\
&+ 0.125M_{BbAR}(i-1)F_{bbAa}(i-1) + 0.25M_{BbAR}(i-1)F_{bbAA}(i-1) + 0.125M_{BbAR}(i-1)F_{bbAR}(i-1)\\
&+ 0.0625M_{BbAR}(i-1)(1-\Phi-\eta)F_{BbAa}(i-1) + 0.125M_{BbAR}(i-1)\Phi F_{BbAa}(i-1)\\
&+ 0.0625M_{BbAR}(i-1)\eta F_{BbAa}(i-1) + 0.125M_{BbAR}(i-1)F_{BbAA}(i-1) + 0.0625M_{BbAR}(i-1)F_{BbAR}(i-1)],
\end{aligned}
$$

$$
\begin{aligned}
M^{e}_{bbAR} = \left(\frac{\Omega_{bbAR}}{2}\right)&[0.25M_{bbAa}(i-1)F_{bbRa}(i-1) + 0.25M_{bbAa}(i-1)F_{bbAR}(i-1) + 0.5M_{bbAa}(i-1)F_{bbRR}(i-1)\\
&+ 0.125M_{bbAa}(i-1)\eta F_{BbAa}(i-1) + 0.125M_{bbAa}(i-1)F_{BbRa}(i-1) + 0.125M_{bbAa}(i-1)F_{BbAR}(i-1)\\
&+ 0.25M_{bbAa}(i-1)F_{BbRR}(i-1) + 0.25M_{bbRa}(i-1)F_{bbAa}(i-1) + 0.5M_{bbRa}(i-1)F_{bbAA}(i-1)\\
&+ 0.25M_{bbRa}(i-1)F_{bbAR}(i-1) + 0.125M_{bbRa}(i-1)(1-\Phi-\eta)F_{BbAa}(i-1)\\
&+ 0.25M_{bbRa}(i-1)\Phi F_{BbAa}(i-1) + 0.125M_{bbRa}(i-1)\eta F_{BbAa}(i-1) + 0.25M_{bbRa}(i-1)F_{BbAA}(i-1)\\
&+ 0.125M_{bbRa}(i-1)F_{BbAR}(i-1) + 0.5M_{bbAA}(i-1)F_{bbRa}(i-1) + 0.5M_{bbAA}(i-1)F_{bbAR}(i-1)\\
&+ M_{bbAA}(i-1)F_{bbRR}(i-1) + 0.25M_{bbAA}(i-1)\eta F_{BbAa}(i-1) + 0.25M_{bbAA}(i-1)F_{BbRa}(i-1)\\
&+ 0.25M_{bbAA}(i-1)F_{BbAR}(i-1) + 0.5M_{bbAA}(i-1)F_{BbRR}(i-1) + 0.25M_{bbAR}(i-1)F_{bbAa}(i-1)\\
&+ 0.25M_{bbAR}(i-1)F_{bbRa}(i-1) + 0.5M_{bbAR}(i-1)F_{bbAA}(i-1) + 0.5M_{bbAR}(i-1)F_{bbAR}(i-1)\\
&+ 0.5M_{bbAR}(i-1)F_{bbRR}(i-1) + 0.125M_{bbAR}(i-1)(1-\Phi-\eta)F_{BbAa}(i-1)\\
&+ 0.25M_{bbAR}(i-1)\Phi F_{BbAa}(i-1) + 0.25M_{bbAR}(i-1)\eta F_{BbAa}(i-1) + 0.125M_{bbAR}(i-1)F_{BbRa}(i-1)\\
&+ 0.25M_{bbAR}(i-1)F_{BbAA}(i-1) + 0.25M_{bbAR}(i-1)F_{BbAR}(i-1) + 0.25M_{bbAR}(i-1)F_{BbRR}(i-1)\\
&+ 0.5M_{bbRR}(i-1)F_{bbAa}(i-1) + M_{bbRR}(i-1)F_{bbAA}(i-1) + 0.5M_{bbRR}(i-1)F_{bbAR}(i-1)\\
&+ 0.25M_{bbRR}(i-1)(1-\Phi-\eta)F_{BbAa}(i-1) + 0.5M_{bbRR}(i-1)\Phi F_{BbAa}(i-1)\\
&+ 0.25M_{bbRR}(i-1)\eta F_{BbAa}(i-1) + 0.5M_{bbRR}(i-1)F_{BbAA}(i-1) + 0.25M_{bbRR}(i-1)F_{BbAR}(i-1)\\
&+ 0.125\eta M_{BbAa}(i-1)F_{bbAa}(i-1) + 0.125(1-\Phi-\eta)M_{BbAa}(i-1)F_{bbRa}(i-1)\\
&+ 0.25\Phi M_{BbAa}(i-1)F_{bbRa}(i-1) + 0.125\eta M_{BbAa}(i-1)F_{bbRa}(i-1) + 0.25\eta M_{BbAa}(i-1)F_{bbAA}(i-1)\\
&+ 0.125(1-\Phi-\eta)M_{BbAa}(i-1)F_{bbAR}(i-1) + 0.25\Phi M_{BbAa}(i-1)F_{bbAR}(i-1)\\
&+ 0.25\eta M_{BbAa}(i-1)F_{bbAR}(i-1) + 0.25(1-\Phi-\eta)M_{BbAa}(i-1)F_{bbRR}(i-1)\\
&+ 0.5\Phi M_{BbAa}(i-1)F_{bbRR}(i-1) + 0.25\eta M_{BbAa}(i-1)F_{bbRR}(i-1)\\
&+ 0.0625\eta M_{BbAa}(i-1)(1-\Phi-\eta)F_{BbAa}(i-1) + 0.0625(1-\Phi-\eta)M_{BbAa}(i-1)\eta F_{BbAa}(i-1)\\
&+ 0.125\Phi M_{BbAa}(i-1)\eta F_{BbAa}(i-1) + 0.125\eta M_{BbAa}(i-1)\Phi F_{BbAa}(i-1)\\
&+ 0.125\eta M_{BbAa}(i-1)\eta F_{BbAa}(i-1) + 0.0625(1-\Phi-\eta)M_{BbAa}(i-1)F_{BbRa}(i-1)\\
&+ 0.125\Phi M_{BbAa}(i-1)F_{BbRa}(i-1) + 0.0625\eta M_{BbAa}(i-1)F_{BbRa}(i-1) + 0.125\eta M_{BbAa}(i-1)F_{BbAA}(i-1)\\
&+ 0.0625(1-\Phi-\eta)M_{BbAa}(i-1)F_{BbAR}(i-1) + 0.125\Phi M_{BbAa}(i-1)F_{BbAR}(i-1)\\
&+ 0.125\eta M_{BbAa}(i-1)F_{BbAR}(i-1) + 0.125(1-\Phi-\eta)M_{BbAa}(i-1)F_{BbRR}(i-1)\\
&+ 0.25\Phi M_{BbAa}(i-1)F_{BbRR}(i-1) + 0.125\eta M_{BbAa}(i-1)F_{BbRR}(i-1)\\
&+ 0.0625(1-\Phi-\eta)M_{BbAa}(i-1)\eta F_{BBAa}(i-1) + 0.125M_{BbRa}(i-1)F_{bbAa}(i-1)
\end{aligned}
$$

$$
\begin{aligned}
&+ 0.25M_{BbRa}(i-1)F_{bbAA}(i-1) + 0.125M_{BbRa}(i-1)F_{bbAR}(i-1)\\
&+ 0.0625M_{BbRa}(i-1)(1-\Phi-\eta)F_{BbAa}(i-1) + 0.125M_{BbRa}(i-1)\Phi F_{BbAa}(i-1)\\
&+ 0.0625M_{BbRa}(i-1)\eta F_{BbAa}(i-1) + 0.125M_{BbRa}(i-1)F_{BbAA}(i-1) + 0.0625M_{BbRa}(i-1)F_{BbAR}(i-1)\\
&+ 0.25M_{BbAA}(i-1)F_{bbRa}(i-1) + 0.25M_{BbAA}(i-1)F_{bbAR}(i-1) + 0.5M_{BbAA}(i-1)F_{bbRR}(i-1)\\
&+ 0.125M_{BbAA}(i-1)\eta F_{BbAa}(i-1) + 0.125M_{BbAA}(i-1)F_{BbRa}(i-1) + 0.125M_{BbAA}(i-1)F_{BbAR}(i-1)\\
&+ 0.25M_{BbAA}(i-1)F_{BbRR}(i-1) + 0.125M_{BbAR}(i-1)F_{bbAa}(i-1) + 0.125M_{BbAR}(i-1)F_{bbRa}(i-1)\\
&+ 0.25M_{BbAR}(i-1)F_{bbAA}(i-1) + 0.25M_{BbAR}(i-1)F_{bbAR}(i-1) + 0.25M_{BbAR}(i-1)F_{bbRR}(i-1)\\
&+ 0.0625M_{BbAR}(i-1)(1-\Phi-\eta)F_{BbAa}(i-1) + 0.125M_{BbAR}(i-1)\Phi F_{BbAa}(i-1)\\
&+ 0.125M_{BbAR}(i-1)\eta F_{BbAa}(i-1) + 0.0625M_{BbAR}(i-1)F_{BbRa}(i-1) + 0.125M_{BbAR}(i-1)F_{BbAA}(i-1)\\
&+ 0.125M_{BbAR}(i-1)F_{BbAR}(i-1) + 0.125M_{BbAR}(i-1)F_{BbRR}(i-1) + 0.25M_{BbRR}(i-1)F_{bbAa}(i-1)\\
&+ 0.5M_{BbRR}(i-1)F_{bbAA}(i-1) + 0.25M_{BbRR}(i-1)F_{bbAR}(i-1) + 0.125M_{BbRR}(i-1)(1-\Phi-\eta)F_{BbAa}(i-1)\\
&+ 0.25M_{BbRR}(i-1)\Phi F_{BbAa}(i-1) + 0.125M_{BbRR}(i-1)\eta F_{BbAa}(i-1) + 0.25M_{BbRR}(i-1)F_{BbAA}(i-1)\\
&+ 0.125M_{BbRR}(i-1)F_{BbAR}(i-1)],
\end{aligned}
$$

$$
\begin{aligned}
M^e_{bbRR} = \left(\frac{\Omega_{bbRR}}{2}\right)&[0.25M_{bbRa}(i-1)F_{bbRa}(i-1) + 0.25M_{bbRa}(i-1)F_{bbAR}(i-1) + 0.5M_{bbRa}(i-1)F_{bbRR}(i-1)\\
&+ 0.125M_{bbRa}(i-1)\eta F_{BbAa}(i-1) + 0.125M_{bbRa}(i-1)F_{BbRa}(i-1) + 0.125M_{bbRa}(i-1)F_{BbAR}(i-1)\\
&+ 0.25M_{bbRa}(i-1)F_{BbRR}(i-1) + 0.25M_{bbAR}(i-1)F_{bbRa}(i-1) + 0.25M_{bbAR}(i-1)F_{bbAR}(i-1)\\
&+ 0.5M_{bbAR}(i-1)F_{bbRR}(i-1) + 0.125M_{bbAR}(i-1)\eta F_{BbAa}(i-1) + 0.125M_{bbAR}(i-1)F_{BbRa}(i-1)\\
&+ 0.125M_{bbAR}(i-1)F_{BbAR}(i-1) + 0.25M_{bbAR}(i-1)F_{BbRR}(i-1) + 0.5M_{bbRR}(i-1)F_{bbRa}(i-1)\\
&+ 0.5M_{bbRR}(i-1)F_{bbAR}(i-1) + M_{bbRR}(i-1)F_{bbRR}(i-1) + 0.25M_{bbRR}(i-1)\eta F_{BbAa}(i-1)\\
&+ 0.25M_{bbRR}(i-1)F_{BbRa}(i-1) + 0.25M_{bbRR}(i-1)F_{BbAR}(i-1) + 0.5M_{bbRR}(i-1)F_{BbRR}(i-1)\\
&+ 0.125\eta M_{BbAa}(i-1)F_{bbRa}(i-1) + 0.125\eta M_{BbAa}(i-1)F_{bbAR}(i-1) + 0.25\eta M_{BbAa}(i-1)F_{bbRR}(i-1)\\
&+ 0.0625\eta M_{BbAa}(i-1)\eta F_{BbAa}(i-1) + 0.0625\eta M_{BbAa}(i-1)F_{BbRa}(i-1)\\
&+ 0.0625\eta M_{BbAa}(i-1)F_{BbAR}(i-1) + 0.125\eta M_{BbAa}(i-1)F_{BbRR}(i-1) + 0.125M_{BbRa}(i-1)F_{bbRa}(i-1)\\
&+ 0.125M_{BbRa}(i-1)F_{bbAR}(i-1) + 0.25M_{BbRa}(i-1)F_{bbRR}(i-1) + 0.0625M_{BbRa}(i-1)\eta F_{BbAa}(i-1)\\
&+ 0.0625M_{BbRa}(i-1)F_{BbRa}(i-1) + 0.0625M_{BbRa}(i-1)F_{BbAR}(i-1) + 0.125M_{BbRa}(i-1)F_{BbRR}(i-1)\\
&+ 0.125M_{BbAR}(i-1)F_{bbRa}(i-1) + 0.125M_{BbAR}(i-1)F_{bbAR}(i-1) + 0.25M_{BbAR}(i-1)F_{bbRR}(i-1)\\
&+ 0.0625M_{BbAR}(i-1)\eta F_{BbAa}(i-1) + 0.0625M_{BbAR}(i-1)F_{BbRa}(i-1) + 0.0625M_{BbAR}(i-1)F_{BbAR}(i-1)\\
&+ 0.125M_{BbAR}(i-1)F_{BbRR}(i-1) + 0.25M_{BbRR}(i-1)F_{bbRa}(i-1) + 0.25M_{BbRR}(i-1)F_{bbAR}(i-1)\\
&+ 0.5M_{BbRR}(i-1)F_{bbRR}(i-1) + 0.125M_{BbRR}(i-1)\eta F_{BbAa}(i-1) + 0.125M_{BbRR}(i-1)F_{BbRa}(i-1)\\
&+ 0.125M_{BbRR}(i-1)F_{BbAR}(i-1) + 0.25M_{BbRR}(i-1)F_{BbRR}(i-1)],
\end{aligned}
$$

$$
\begin{aligned}
M^e_{Bbaa} = \left(\frac{\Omega_{Bbaa}}{2}\right)&[0.5M_{bbaa}(i-1)F_{Bbaa}(i-1) + 0.25M_{bbaa}(i-1)(1-\Phi-\eta)F_{BbAa}(i-1)\\
&+ 0.25M_{bbaa}(i-1)F_{BbRa}(i-1) + M_{bbaa}(i-1)F_{BBaa}(i-1) + 0.5M_{bbaa}(i-1)(1-\Phi-\eta)F_{BBAa}(i-1)\\
&+ 0.5M_{bbaa}(i-1)F_{BBRa}(i-1) + 0.25M_{bbAa}(i-1)F_{Bbaa}(i-1) + 0.125M_{bbAa}(i-1)(1-\Phi-\eta)F_{BbAa}(i-1)\\
&+ 0.125M_{bbAa}(i-1)F_{BbRa}(i-1) + 0.5M_{bbAa}(i-1)F_{BBaa}(i-1) + 0.25M_{bbAa}(i-1)(1-\Phi-\eta)F_{BBAa}(i-1)\\
&+ 0.25M_{bbAa}(i-1)F_{BBRa}(i-1) + 0.25M_{bbRa}(i-1)F_{Bbaa}(i-1) + 0.125M_{bbRa}(i-1)(1-\Phi-\eta)F_{BbAa}(i-1)\\
&+ 0.125M_{bbRa}(i-1)F_{BbRa}(i-1) + 0.5M_{bbRa}(i-1)F_{BBaa}(i-1) + 0.25M_{bbRa}(i-1)(1-\Phi-\eta)F_{BBAa}(i-1)\\
&+ 0.25M_{bbRa}(i-1)F_{BBRa}(i-1) + 0.5M_{Bbaa}(i-1)F_{bbaa}(i-1) + 0.25M_{Bbaa}(i-1)F_{bbAa}(i-1)\\
&+ 0.25M_{Bbaa}(i-1)F_{bbRa}(i-1) + 0.5M_{Bbaa}(i-1)F_{Bbaa}(i-1) + 0.25M_{Bbaa}(i-1)(1-\Phi-\eta)F_{BbAa}(i-1)\\
&+ 0.25M_{Bbaa}(i-1)F_{BbRa}(i-1) + 0.5M_{Bbaa}(i-1)F_{BBaa}(i-1) + 0.25M_{Bbaa}(i-1)(1-\Phi-\eta)F_{BBAa}(i-1)\\
&+ 0.25M_{Bbaa}(i-1)F_{BBRa}(i-1) + 0.25(1-\Phi-\eta)M_{BbAa}(i-1)F_{bbaa}(i-1)\\
&+ 0.125(1-\Phi-\eta)M_{BbAa}(i-1)F_{bbAa}(i-1) + 0.125(1-\Phi-\eta)M_{BbAa}(i-1)F_{bbRa}(i-1)\\
&+ 0.25(1-\Phi-\eta)M_{BbAa}(i-1)F_{Bbaa}(i-1) + 0.125(1-\Phi-\eta)M_{BbAa}(i-1)(1-\Phi-\eta)F_{BbAa}(i-1)\\
&+ 0.125(1-\Phi-\eta)M_{BbAa}(i-1)F_{BbRa}(i-1) + 0.25(1-\Phi-\eta)M_{BbAa}(i-1)F_{BBaa}(i-1)\\
&+ 0.125(1-\Phi-\eta)M_{BbAa}(i-1)(1-\Phi-\eta)F_{BBAa}(i-1) + 0.125(1-\Phi-\eta)M_{BbAa}(i-1)F_{BBRa}(i-1)\\
&+ 0.25M_{BbRa}(i-1)F_{bbaa}(i-1) + 0.125M_{BbRa}(i-1)F_{bbAa}(i-1) + 0.125M_{BbRa}(i-1)F_{bbRa}(i-1)
\end{aligned}
$$

$$
\begin{aligned}
&+ 0.25M_{BbRa}(i-1)F_{Bbaa}(i-1) + 0.125M_{BbRa}(i-1)(1-\Phi-\eta)F_{BbAa}(i-1)\\
&+ 0.125M_{BbRa}(i-1)F_{BbRa}(i-1) + 0.25M_{BbRa}(i-1)F_{BBaa}(i-1)\\
&+ 0.125M_{BbRa}(i-1)(1-\Phi-\eta)F_{BBAa}(i-1) + 0.125M_{BbRa}(i-1)F_{BBRa}(i-1) + M_{BBaa}(i-1)F_{bbaa}(i-1)\\
&+ 0.5M_{BBaa}(i-1)F_{bbAa}(i-1) + 0.5M_{BBaa}(i-1)F_{bbRa}(i-1) + 0.5M_{BBaa}(i-1)F_{Bbaa}(i-1)\\
&+ 0.25M_{BBaa}(i-1)(1-\Phi-\eta)F_{BbAa}(i-1) + 0.25M_{BBaa}(i-1)F_{BbRa}(i-1)\\
&+ 0.5(1-\Phi-\eta)M_{BBAa}(i-1)F_{bbaa}(i-1) + 0.25(1-\Phi-\eta)M_{BBAa}(i-1)F_{bbAa}(i-1)\\
&+ 0.25(1-\Phi-\eta)M_{BBAa}(i-1)F_{bbRa}(i-1) + 0.25(1-\Phi-\eta)M_{BBAa}(i-1)F_{Bbaa}(i-1)\\
&+ 0.125(1-\Phi-\eta)M_{BBAa}(i-1)(1-\Phi-\eta)F_{BbAa}(i-1) + 0.125(1-\Phi-\eta)M_{BBAa}(i-1)F_{BbRa}(i-1)\\
&+ 0.5M_{BBRa}(i-1)F_{bbaa}(i-1) + 0.25M_{BBRa}(i-1)F_{bbAa}(i-1) + 0.25M_{BBRa}(i-1)F_{bbRa}(i-1)\\
&+ 0.25M_{BBRa}(i-1)F_{Bbaa}(i-1) + 0.125M_{BBRa}(i-1)(1-\Phi-\eta)F_{BbAa}(i-1)\\
&+ 0.125M_{BBRa}(i-1)F_{BbRa}(i-1)],\\
M^{e}_{BbAa} = &\left(\frac{\Omega_{BbAa}}{2}\right)[0.25M_{bbaa}(i-1)(1-\Phi-\eta)F_{BbAa}(i-1) + 0.5M_{bbaa}(i-1)\Phi F_{BbAa}(i-1)\\
&+ 0.25M_{bbaa}(i-1)\eta F_{BbAa}(i-1) + 0.5M_{bbaa}(i-1)F_{BbAA}(i-1) + 0.25M_{bbaa}(i-1)F_{BbAR}(i-1)\\
&+ 0.5M_{bbaa}(i-1)(1-\Phi-\eta)F_{BBAa}(i-1) + M_{bbaa}(i-1)\Phi F_{BBAa}(i-1) + 0.5M_{bbaa}(i-1)\eta F_{BBAa}(i-1)\\
&+ M_{bbaa}(i-1)F_{BBAA}(i-1) + 0.5M_{bbaa}(i-1)F_{BBAR}(i-1) + 0.25M_{bbAa}(i-1)F_{Bbaa}(i-1)\\
&+ 0.25M_{bbAa}(i-1)(1-\Phi-\eta)F_{BbAa}(i-1) + 0.25M_{bbAa}(i-1)\Phi F_{BbAa}(i-1)\\
&+ 0.125M_{bbAa}(i-1)\eta F_{BbAa}(i-1) + 0.125M_{bbAa}(i-1)F_{BbRa}(i-1) + 0.25M_{bbAa}(i-1)F_{BbAA}(i-1)\\
&+ 0.125M_{bbAa}(i-1)F_{BbAR}(i-1) + 0.5M_{bbAa}(i-1)F_{BBaa}(i-1) + 0.5M_{bbAa}(i-1)(1-\Phi-\eta)F_{BBAa}(i-1)\\
&+ 0.5M_{bbAa}(i-1)\Phi F_{BBAa}(i-1) + 0.25M_{bbAa}(i-1)\eta F_{BBAa}(i-1) + 0.25M_{bbAa}(i-1)F_{BBRa}(i-1)\\
&+ 0.5M_{bbAa}(i-1)F_{BBAA}(i-1) + 0.25M_{bbAa}(i-1)F_{BBAR}(i-1)\\
&+ 0.125M_{bbRa}(i-1)(1-\Phi-\eta)F_{BbAa}(i-1) + 0.25M_{bbRa}(i-1)\Phi F_{BbAa}(i-1)\\
&+ 0.125M_{bbRa}(i-1)\eta F_{BbAa}(i-1) + 0.25M_{bbRa}(i-1)F_{BbAA}(i-1) + 0.125M_{bbRa}(i-1)F_{BbAR}(i-1)\\
&+ 0.25M_{bbRa}(i-1)(1-\Phi-\eta)F_{BBAa}(i-1) + 0.5M_{bbRa}(i-1)\Phi F_{BBAa}(i-1)\\
&+ 0.25M_{bbRa}(i-1)\eta F_{BBAa}(i-1) + 0.5M_{bbRa}(i-1)F_{BBAA}(i-1) + 0.25M_{bbRa}(i-1)F_{BBAR}(i-1)\\
&+ 0.5M_{bbAA}(i-1)F_{Bbaa}(i-1) + 0.25M_{bbAA}(i-1)(1-\Phi-\eta)F_{BbAa}(i-1) + 0.25M_{bbAA}(i-1)F_{BbRa}(i-1)\\
&+ M_{bbAA}(i-1)F_{BBaa}(i-1) + 0.5M_{bbAA}(i-1)(1-\Phi-\eta)F_{BBAa}(i-1) + 0.5M_{bbAA}(i-1)F_{BBRa}(i-1)\\
&+ 0.25M_{bbAR}(i-1)F_{Bbaa}(i-1) + 0.125M_{bbAR}(i-1)(1-\Phi-\eta)F_{BbAa}(i-1)\\
&+ 0.125M_{bbAR}(i-1)F_{BbRa}(i-1) + 0.5M_{bbAR}(i-1)F_{BBaa}(i-1) + 0.25M_{bbAR}(i-1)(1-\Phi-\eta)F_{BBAa}(i-1)\\
&+ 0.25M_{bbAR}(i-1)F_{BBRa}(i-1) + 0.25M_{Bbaa}(i-1)F_{bbAa}(i-1) + 0.5M_{Bbaa}(i-1)F_{bbAA}(i-1)\\
&+ 0.25M_{Bbaa}(i-1)F_{bbAR}(i-1) + 0.25M_{Bbaa}(i-1)(1-\Phi-\eta)F_{BbAa}(i-1) + 0.5M_{Bbaa}(i-1)\Phi F_{BbAa}(i-1)\\
&+ 0.25M_{Bbaa}(i-1)\eta F_{BbAa}(i-1) + 0.5M_{Bbaa}(i-1)F_{BbAA}(i-1) + 0.25M_{Bbaa}(i-1)F_{BbAR}(i-1)\\
&+ 0.25M_{Bbaa}(i-1)(1-\Phi-\eta)F_{BBAa}(i-1) + 0.5M_{Bbaa}(i-1)\Phi F_{BBAa}(i-1)\\
&+ 0.25M_{Bbaa}(i-1)\eta F_{BBAa}(i-1) + 0.5M_{Bbaa}(i-1)F_{BBAA}(i-1) + 0.25M_{Bbaa}(i-1)F_{BBAR}(i-1)\\
&+ 0.25(1-\Phi-\eta)M_{BbAa}(i-1)F_{bbaa}(i-1) + 0.5\Phi M_{BbAa}(i-1)F_{bbaa}(i-1) + 0.25\eta M_{BbAa}(i-1)F_{bbaa}(i-1)\\
&+ 0.25(1-\Phi-\eta)M_{BbAa}(i-1)F_{bbAa}(i-1) + 0.25\Phi M_{BbAa}(i-1)F_{bbAa}(i-1)\\
&+ 0.125\eta M_{BbAa}(i-1)F_{bbAa}(i-1) + 0.125(1-\Phi-\eta)M_{BbAa}(i-1)F_{bbRa}(i-1)\\
&+ 0.25\Phi M_{BbAa}(i-1)F_{bbRa}(i-1) + 0.125\eta M_{BbAa}(i-1)F_{bbRa}(i-1)\\
&+ 0.25(1-\Phi-\eta)M_{BbAa}(i-1)F_{bbAA}(i-1) + 0.125(1-\Phi-\eta)M_{BbAa}(i-1)F_{bbAR}(i-1)\\
&+ 0.25(1-\Phi-\eta)M_{BbAa}(i-1)F_{Bbaa}(i-1) + 0.5\Phi M_{BbAa}(i-1)F_{Bbaa}(i-1) + 0.25\eta M_{BbAa}(i-1)F_{Bbaa}(i-1)\\
&+ 0.25(1-\Phi-\eta)M_{BbAa}(i-1)(1-\Phi-\eta)F_{BbAa}(i-1) + 0.25\Phi M_{BbAa}(i-1)(1-\Phi-\eta)F_{BbAa}(i-1)\\
&+ 0.125\eta M_{BbAa}(i-1)(1-\Phi-\eta)F_{BbAa}(i-1) + 0.25(1-\Phi-\eta)M_{BbAa}(i-1)\Phi F_{BbAa}(i-1)\\
&+ 0.125(1-\Phi-\eta)M_{BbAa}(i-1)\eta F_{BbAa}(i-1) + 0.125(1-\Phi-\eta)M_{BbAa}(i-1)F_{BbRa}(i-1)\\
&+ 0.25\Phi M_{BbAa}(i-1)F_{BbRa}(i-1) + 0.125\eta M_{BbAa}(i-1)F_{BbRa}(i-1) + 0.25(1-\Phi-\eta)M_{BbAa}(i-1)F_{BbAA}(i-\\
&+ 0.125(1-\Phi-\eta)M_{BbAa}(i-1)F_{BbAR}(i-1) + 0.25(1-\Phi-\eta)M_{BbAa}(i-1)F_{BBaa}(i-1)\\
&+ 0.5\Phi M_{BbAa}(i-1)F_{BBaa}(i-1) + 0.25\eta M_{BbAa}(i-1)F_{BBaa}(i-1)\\
&+ 0.25(1-\Phi-\eta)M_{BbAa}(i-1)(1-\Phi-\eta)F_{BBAa}(i-1) + 0.25\Phi M_{BbAa}(i-1)(1-\Phi-\eta)F_{BBAa}(i-1)
\end{aligned}
$$

$$
\begin{aligned}
&+0.125\eta M_{BbAa}(i-1)(1-\Phi-\eta)F_{BBAa}(i-1)+0.25(1-\Phi-\eta)M_{BbAa}(i-1)\Phi F_{BBAa}(i-1)\\
&+0.125(1-\Phi-\eta)M_{BbAa}(i-1)\eta F_{BBAa}(i-1)+0.125(1-\Phi-\eta)M_{BbAa}(i-1)F_{BBRa}(i-1)\\
&+0.25\Phi M_{BbAa}(i-1)F_{BBRa}(i-1)+0.125\eta M_{BbAa}(i-1)F_{BBRa}(i-1)\\
&+0.25(1-\Phi-\eta)M_{BbAa}(i-1)F_{BBAA}(i-1)+0.125(1-\Phi-\eta)M_{BbAa}(i-1)F_{BBAR}(i-1)\\
&+0.125M_{BbRa}(i-1)F_{bbAa}(i-1)+0.25M_{BbRa}(i-1)F_{bbAA}(i-1)+0.125M_{BbRa}(i-1)F_{bbAR}(i-1)\\
&+0.125M_{BbRa}(i-1)(1-\Phi-\eta)F_{BbAa}(i-1)+0.25M_{BbRa}(i-1)\Phi F_{BbAa}(i-1)\\
&+0.125M_{BbRa}(i-1)\eta F_{BbAa}(i-1)+0.25M_{BbRa}(i-1)F_{BbAA}(i-1)+0.125M_{BbRa}(i-1)F_{BbAR}(i-1)\\
&+0.125M_{BbRa}(i-1)(1-\Phi-\eta)F_{BBAa}(i-1)+0.25M_{BbRa}(i-1)\Phi F_{BBAa}(i-1)\\
&+0.125M_{BbRa}(i-1)\eta F_{BBAa}(i-1)+0.25M_{BbRa}(i-1)F_{BBAA}(i-1)+0.125M_{BbRa}(i-1)F_{BBAR}(i-1)\\
&+0.5M_{BbAA}(i-1)F_{bbaa}(i-1)+0.25M_{BbAA}(i-1)F_{bbAa}(i-1)+0.25M_{BbAA}(i-1)F_{bbRa}(i-1)\\
&+0.5M_{BbAA}(i-1)F_{Bbaa}(i-1)+0.25M_{BbAA}(i-1)(1-\Phi-\eta)F_{BbAa}(i-1)+0.25M_{BbAA}(i-1)F_{BbRa}(i-1)\\
&+0.5M_{BbAA}(i-1)F_{BBaa}(i-1)+0.25M_{BbAA}(i-1)(1-\Phi-\eta)F_{BBAa}(i-1)\\
&+0.25M_{BbAA}(i-1)F_{BBRa}(i-1)+0.25M_{BbAR}(i-1)F_{bbaa}(i-1)+0.125M_{BbAR}(i-1)F_{bbAa}(i-1)\\
&+0.125M_{BbAR}(i-1)F_{bbRa}(i-1)+0.25M_{BbAR}(i-1)F_{Bbaa}(i-1)\\
&+0.125M_{BbAR}(i-1)(1-\Phi-\eta)F_{BbAa}(i-1)+0.125M_{BbAR}(i-1)F_{BbRa}(i-1)\\
&+0.25M_{BbAR}(i-1)F_{BBaa}(i-1)+0.125M_{BbAR}(i-1)(1-\Phi-\eta)F_{BBAa}(i-1)\\
&+0.125M_{BbAR}(i-1)F_{BBRa}(i-1)+0.5M_{BBaa}(i-1)F_{bbAa}(i-1)+M_{BBaa}(i-1)F_{bbAA}(i-1)\\
&+0.5M_{BBaa}(i-1)F_{bbAR}(i-1)+0.25M_{BBaa}(i-1)(1-\Phi-\eta)F_{BbAa}(i-1)+0.5M_{BBaa}(i-1)\Phi F_{BbAa}(i-1)\\
&+0.25M_{BBaa}(i-1)\eta F_{BbAa}(i-1)+0.5M_{BBaa}(i-1)F_{BbAA}(i-1)+0.25M_{BBaa}(i-1)F_{BbAR}(i-1)\\
&+0.5(1-\Phi-\eta)M_{BBAa}(i-1)F_{bbaa}(i-1)+\Phi M_{BBAa}(i-1)F_{bbaa}(i-1)+0.5\eta M_{BBAa}(i-1)F_{bbaa}(i-1)\\
&+0.5(1-\Phi-\eta)M_{BBAa}(i-1)F_{bbAa}(i-1)+0.5\Phi M_{BBAa}(i-1)F_{bbAa}(i-1)\\
&+0.25\eta M_{BBAa}(i-1)F_{bbAa}(i-1)+0.25(1-\Phi-\eta)M_{BBAa}(i-1)F_{bbRa}(i-1)\\
&+0.5\Phi M_{BBAa}(i-1)F_{bbRa}(i-1)+0.25\eta M_{BBAa}(i-1)F_{bbRa}(i-1)\\
&+0.5(1-\Phi-\eta)M_{BBAa}(i-1)F_{bbAA}(i-1)+0.25(1-\Phi-\eta)M_{BBAa}(i-1)F_{bbAR}(i-1)\\
&+0.25(1-\Phi-\eta)M_{BBAa}(i-1)F_{Bbaa}(i-1)+0.5\Phi M_{BBAa}(i-1)F_{Bbaa}(i-1)\\
&+0.25\eta M_{BBAa}(i-1)F_{Bbaa}(i-1)+0.25(1-\Phi-\eta)M_{BBAa}(i-1)(1-\Phi-\eta)F_{BbAa}(i-1)\\
&+0.25\Phi M_{BBAa}(i-1)(1-\Phi-\eta)F_{BbAa}(i-1)+0.125\eta M_{BBAa}(i-1)(1-\Phi-\eta)F_{BbAa}(i-1)\\
&+0.25(1-\Phi-\eta)M_{BBAa}(i-1)\Phi F_{BbAa}(i-1)+0.125(1-\Phi-\eta)M_{BBAa}(i-1)\eta F_{BbAa}(i-1)\\
&+0.125(1-\Phi-\eta)M_{BBAa}(i-1)F_{BbRa}(i-1)+0.25\Phi M_{BBAa}(i-1)F_{BbRa}(i-1)\\
&+0.125\eta M_{BBAa}(i-1)F_{BbRa}(i-1)+0.25(1-\Phi-\eta)M_{BBAa}(i-1)F_{BbAA}(i-1)\\
&+0.125(1-\Phi-\eta)M_{BBAa}(i-1)F_{BbAR}(i-1)+0.25M_{BBRa}(i-1)F_{bbAa}(i-1)\\
&+0.5M_{BBRa}(i-1)F_{bbAA}(i-1)+0.25M_{BBRa}(i-1)F_{bbAR}(i-1)\\
&+0.125M_{BBRa}(i-1)(1-\Phi-\eta)F_{BbAa}(i-1)+0.25M_{BBRa}(i-1)\Phi F_{BbAa}(i-1)\\
&+0.125M_{BBRa}(i-1)\eta F_{BbAa}(i-1)+0.25M_{BBRa}(i-1)F_{BbAA}(i-1)+0.125M_{BBRa}(i-1)F_{BbAR}(i-1)\\
&+M_{BBAA}(i-1)F_{bbaa}(i-1)+0.5M_{BBAA}(i-1)F_{bbAa}(i-1)+0.5M_{BBAA}(i-1)F_{bbRa}(i-1)\\
&+0.5M_{BBAA}(i-1)F_{Bbaa}(i-1)+0.25M_{BBAA}(i-1)(1-\Phi-\eta)F_{BbAa}(i-1)\\
&+0.25M_{BBAA}(i-1)F_{BbRa}(i-1)+0.5M_{BBAR}(i-1)F_{bbaa}(i-1)+0.25M_{BBAR}(i-1)F_{bbAa}(i-1)\\
&+0.25M_{BBAR}(i-1)F_{bbRa}(i-1)+0.25M_{BBAR}(i-1)F_{Bbaa}(i-1)\\
&+0.125M_{BBAR}(i-1)(1-\Phi-\eta)F_{BbAa}(i-1)+0.125M_{BBAR}(i-1)F_{BbRa}(i-1)],\\
M^e_{BbRa}&=(\Omega_{BbRa}/2)[0.25M_{bbaa}(i-1)\eta F_{BbAa}(i-1)+0.25M_{bbaa}(i-1)F_{BbRa}(i-1)+0.25M_{bbaa}(i-1)F_{BbAR}(i-1)\\
&+0.5M_{bbaa}(i-1)F_{BbRR}(i-1)+0.5M_{bbaa}(i-1)\eta F_{BBAa}(i-1)+0.5M_{bbaa}(i-1)F_{BBRa}(i-1)\\
&+0.5M_{bbaa}(i-1)F_{BBAR}(i-1)+M_{bbaa}(i-1)F_{BBRR}(i-1)+0.125M_{bbAa}(i-1)\eta F_{BbAa}(i-1)\\
&+0.125M_{bbAa}(i-1)F_{BbRa}(i-1)+0.125M_{bbAa}(i-1)F_{BbAR}(i-1)+0.25M_{bbAa}(i-1)F_{BbRR}(i-1)\\
&+0.25M_{bbAa}(i-1)\eta F_{BBAa}(i-1)+0.25M_{bbAa}(i-1)F_{BBRa}(i-1)+0.25M_{bbAa}(i-1)F_{BBAR}(i-1)\\
&+0.5M_{bbAa}(i-1)F_{BBRR}(i-1)+0.25M_{bbRa}(i-1)F_{Bbaa}(i-1)+0.125M_{bbRa}(i-1)(1-\Phi-\eta)F_{BbAa}(i-1)\\
&+0.125M_{bbRa}(i-1)\eta F_{BbAa}(i-1)+0.25M_{bbRa}(i-1)F_{BbRa}(i-1)+0.125M_{bbRa}(i-1)F_{BbAR}(i-1)\\
&+0.25M_{bbRa}(i-1)F_{BbRR}(i-1)+0.5M_{bbRa}(i-1)F_{BBaa}(i-1)+0.25M_{bbRa}(i-1)(1-\Phi-\eta)F_{BBAa}(i-1)
\end{aligned}
$$

$$+ 0.25M_{bbRa}(i-1)\eta F_{BBAa}(i-1) + 0.5M_{bbRa}(i-1)F_{BBRa}(i-1) + 0.25M_{bbRa}(i-1)F_{BBAR}(i-1)$$
$$+ 0.5M_{bbRa}(i-1)F_{BBRR}(i-1) + 0.25M_{bbAR}(i-1)F_{Bbaa}(i-1) + 0.125M_{bbAR}(i-1)(1-\Phi-\eta)F_{BbAa}(i-1)$$
$$+ 0.125M_{bbAR}(i-1)F_{BbRa}(i-1) + 0.5M_{bbAR}(i-1)F_{BBaa}(i-1) + 0.25M_{bbAR}(i-1)(1-\Phi-\eta)F_{BBAa}(i-1)$$
$$+ 0.25M_{bbAR}(i-1)F_{BBRa}(i-1) + 0.5M_{bbRR}(i-1)F_{Bbaa}(i-1) + 0.25M_{bbRR}(i-1)(1-\Phi-\eta)F_{BbAa}(i-1)$$
$$+ 0.25M_{bbRR}(i-1)F_{BbRa}(i-1) + M_{bbRR}(i-1)F_{BBaa}(i-1) + 0.5M_{bbRR}(i-1)(1-\Phi-\eta)F_{BBAa}(i-1)$$
$$+ 0.5M_{bbRR}(i-1)F_{BBRa}(i-1) + 0.25M_{Bbaa}(i-1)F_{bbRa}(i-1) + 0.25M_{Bbaa}(i-1)F_{bbAR}(i-1)$$
$$+ 0.5M_{Bbaa}(i-1)F_{bbRR}(i-1) + 0.25M_{Bbaa}(i-1)\eta F_{BbAa}(i-1) + 0.25M_{Bbaa}(i-1)F_{BbRa}(i-1)$$
$$+ 0.25M_{Bbaa}(i-1)F_{BbAR}(i-1) + 0.5M_{Bbaa}(i-1)F_{BbRR}(i-1) + 0.25M_{Bbaa}(i-1)\eta F_{BBAa}(i-1)$$
$$+ 0.25M_{Bbaa}(i-1)F_{BBRa}(i-1) + 0.25M_{Bbaa}(i-1)F_{BBAR}(i-1) + 0.5M_{Bbaa}(i-1)F_{BBRR}(i-1)$$
$$+ 0.25\eta M_{BbAa}(i-1)F_{bbaa}(i-1) + 0.125\eta M_{BbAa}(i-1)F_{bbAa}(i-1)$$
$$+ 0.125(1-\Phi-\eta)M_{BbAa}(i-1)F_{bbRa}(i-1) + 0.125\eta M_{BbAa}(i-1)F_{bbRa}(i-1)$$
$$+ 0.125(1-\Phi-\eta)M_{BbAa}(i-1)F_{bbAR}(i-1) + 0.25(1-\Phi-\eta)M_{BbAa}(i-1)F_{bbRR}(i-1)$$
$$+ 0.25\eta M_{BbAa}(i-1)F_{Bbaa}(i-1) + 0.125\eta M_{BbAa}(i-1)(1-\Phi-\eta)F_{BbAa}(i-1)$$
$$+ 0.125(1-\Phi-\eta)M_{BbAa}(i-1)\eta F_{BbAa}(i-1) + 0.125(1-\Phi-\eta)M_{BbAa}(i-1)F_{BbRa}(i-1)$$
$$+ 0.125\eta M_{BbAa}(i-1)F_{BbRa}(i-1) + 0.125(1-\Phi-\eta)M_{BbAa}(i-1)F_{BbAR}(i-1)$$
$$+ 0.25(1-\Phi-\eta)M_{BbAa}(i-1)F_{BbRR}(i-1) + 0.25\eta M_{BbAa}(i-1)F_{BBaa}(i-1)$$
$$+ 0.125\eta M_{BbAa}(i-1)(1-\Phi-\eta)F_{BBAa}(i-1) + 0.125(1-\Phi-\eta)M_{BbAa}(i-1)\eta F_{BBAa}(i-1)$$
$$+ 0.125(1-\Phi-\eta)M_{BbAa}(i-1)F_{BBRa}(i-1) + 0.125\eta M_{BbAa}(i-1)F_{BBRa}(i-1)$$
$$+ 0.125(1-\Phi-\eta)M_{BbAa}(i-1)F_{BBAR}(i-1) + 0.25(1-\Phi-\eta)M_{BbAa}(i-1)F_{BBRR}(i-1)$$
$$+ 0.25M_{BbRa}(i-1)F_{bbaa}(i-1) + 0.125M_{BbRa}(i-1)F_{bbAa}(i-1) + 0.25M_{BbRa}(i-1)F_{bbRa}(i-1)$$
$$+ 0.125M_{BbRa}(i-1)F_{bbAR}(i-1) + 0.25M_{BbRa}(i-1)F_{bbRR}(i-1) + 0.25M_{BbRa}(i-1)F_{Bbaa}(i-1)$$
$$+ 0.125M_{BbRa}(i-1)(1-\Phi-\eta)F_{BbAa}(i-1) + 0.125M_{BbRa}(i-1)\eta F_{BbAa}(i-1)$$
$$+ 0.25M_{BbRa}(i-1)F_{BbRa}(i-1) + 0.125M_{BbRa}(i-1)F_{BbAR}(i-1) + 0.25M_{BbRa}(i-1)F_{BbRR}(i-1)$$
$$+ 0.25M_{BbRa}(i-1)F_{BBaa}(i-1) + 0.125M_{BbRa}(i-1)(1-\Phi-\eta)F_{BBAa}(i-1)$$
$$+ 0.125M_{BbRa}(i-1)\eta F_{BBAa}(i-1) + 0.25M_{BbRa}(i-1)F_{BBRa}(i-1) + 0.125M_{BbRa}(i-1)F_{BBAR}(i-1)$$
$$+ 0.25M_{BbRa}(i-1)F_{BBRR}(i-1) + 0.25M_{BbAR}(i-1)F_{bbaa}(i-1) + 0.125M_{BbAR}(i-1)F_{bbAa}(i-1)$$
$$+ 0.125M_{BbAR}(i-1)F_{bbRa}(i-1) + 0.25M_{BbAR}(i-1)F_{Bbaa}(i-1)$$
$$+ 0.125M_{BbAR}(i-1)(1-\Phi-\eta)F_{BbAa}(i-1) + 0.125M_{BbAR}(i-1)F_{BbRa}(i-1)$$
$$+ 0.25M_{BbAR}(i-1)F_{BBaa}(i-1) + 0.125M_{BbAR}(i-1)(1-\Phi-\eta)F_{BBAa}(i-1)$$
$$+ 0.125M_{BbAR}(i-1)F_{BBRa}(i-1) + 0.5M_{BbRR}(i-1)F_{bbaa}(i-1) + 0.25M_{BbRR}(i-1)F_{bbAa}(i-1)$$
$$+ 0.25M_{BbRR}(i-1)F_{bbRa}(i-1) + 0.5M_{BbRR}(i-1)F_{Bbaa}(i-1) + 0.25M_{BbRR}(i-1)(1-\Phi-\eta)F_{BbAa}(i-1)$$
$$+ 0.25M_{BbRR}(i-1)F_{BbRa}(i-1) + 0.5M_{BbRR}(i-1)F_{BBaa}(i-1) + 0.25M_{BbRR}(i-1)(1-\Phi-\eta)F_{BBAa}(i-1)$$
$$+ 0.25M_{BbRR}(i-1)F_{BBRa}(i-1) + 0.5M_{BBaa}(i-1)F_{bbRa}(i-1) + 0.5M_{BBaa}(i-1)F_{bbAR}(i-1)$$
$$+ M_{BBaa}(i-1)F_{bbRR}(i-1) + 0.25M_{BBaa}(i-1)\eta F_{BbAa}(i-1) + 0.25M_{BBaa}(i-1)F_{BbRa}(i-1)$$
$$+ 0.25M_{BBaa}(i-1)F_{BbAR}(i-1) + 0.5M_{BBaa}(i-1)F_{BbRR}(i-1) + 0.5\eta M_{BBAa}(i-1)F_{bbaa}(i-1)$$
$$+ 0.25\eta M_{BBAa}(i-1)F_{bbAa}(i-1) + 0.25(1-\Phi-\eta)M_{BBAa}(i-1)F_{bbRa}(i-1)$$
$$+ 0.25\eta M_{BBAa}(i-1)F_{bbRa}(i-1) + 0.25(1-\Phi-\eta)M_{BBAa}(i-1)F_{bbAR}(i-1)$$
$$+ 0.5(1-\Phi-\eta)M_{BBAa}(i-1)F_{bbRR}(i-1) + 0.25\eta M_{BBAa}(i-1)F_{Bbaa}(i-1)$$
$$+ 0.125\eta M_{BBAa}(i-1)(1-\Phi-\eta)F_{BbAa}(i-1) + 0.125(1-\Phi-\eta)M_{BBAa}(i-1)\eta F_{BbAa}(i-1)$$
$$+ 0.125(1-\Phi-\eta)M_{BBAa}(i-1)F_{BbRa}(i-1) + 0.125\eta M_{BBAa}(i-1)F_{BbRa}(i-1)$$
$$+ 0.125(1-\Phi-\eta)M_{BBAa}(i-1)F_{BbAR}(i-1) + 0.25(1-\Phi-\eta)M_{BBAa}(i-1)F_{BbRR}(i-1)$$
$$+ 0.5M_{BBRa}(i-1)F_{bbaa}(i-1) + 0.25M_{BBRa}(i-1)F_{bbAa}(i-1) + 0.5M_{BBRa}(i-1)F_{bbRa}(i-1)$$
$$+ 0.25M_{BBRa}(i-1)F_{bbAR}(i-1) + 0.5M_{BBRa}(i-1)F_{bbRR}(i-1) + 0.25M_{BBRa}(i-1)F_{Bbaa}(i-1)$$
$$+ 0.125M_{BBRa}(i-1)(1-\Phi-\eta)F_{BbAa}(i-1) + 0.125M_{BBRa}(i-1)\eta F_{BbAa}(i-1)$$
$$+ 0.25M_{BBRa}(i-1)F_{BbRa}(i-1) + 0.125M_{BBRa}(i-1)F_{BbAR}(i-1) + 0.25M_{BBRa}(i-1)F_{BbRR}(i-1)$$
$$+ 0.5M_{BBAR}(i-1)F_{bbaa}(i-1) + 0.25M_{BBAR}(i-1)F_{bbAa}(i-1) + 0.25M_{BBAR}(i-1)F_{bbRa}(i-1)$$
$$+ 0.25M_{BBAR}(i-1)F_{Bbaa}(i-1) + 0.125M_{BBAR}(i-1)(1-\Phi-\eta)F_{BbAa}(i-1)$$

$$+ 0.125M_{BBAR}(i-1)F_{BbRa}(i-1) + M_{BBRR}(i-1)F_{bbaa}(i-1) + 0.5M_{BBRR}(i-1)F_{bbAa}(i-1)$$
$$+ 0.5M_{BBRR}(i-1)F_{bbRa}(i-1) + 0.5M_{BBRR}(i-1)F_{Bbaa}(i-1)$$
$$+ 0.25M_{BBRR}(i-1)(1-\Phi-\eta)F_{BbAa}(i-1) + 0.25M_{BBRR}(i-1)F_{BbRa}(i-1)],$$

$$M^e_{BbAA} = \left(\frac{\Omega_{BbAA}}{2}\right)[0.125M_{bbAa}(i-1)(1-\Phi-\eta)F_{BbAa}(i-1) + 0.25M_{bbAa}(i-1)\Phi F_{BbAa}(i-1)$$
$$+ 0.125M_{bbAa}(i-1)\eta F_{BbAa}(i-1) + 0.25M_{bbAa}(i-1)F_{BbAA}(i-1) + 0.125M_{bbAa}(i-1)F_{BbAR}(i-1)$$
$$+ 0.25M_{bbAa}(i-1)(1-\Phi-\eta)F_{BBAa}(i-1) + 0.5M_{bbAa}(i-1)\Phi F_{BBAa}(i-1)$$
$$+ 0.25M_{bbAa}(i-1)\eta F_{BBAa}(i-1) + 0.5M_{bbAa}(i-1)F_{BBAA}(i-1) + 0.25M_{bbAa}(i-1)F_{BBAR}(i-1)$$
$$+ 0.25M_{bbAA}(i-1)(1-\Phi-\eta)F_{BbAa}(i-1) + 0.5M_{bbAA}(i-1)\Phi F_{BbAa}(i-1)$$
$$+ 0.25M_{bbAA}(i-1)\eta F_{BbAa}(i-1) + 0.5M_{bbAA}(i-1)F_{BbAA}(i-1) + 0.25M_{bbAA}(i-1)F_{BbAR}(i-1)$$
$$+ 0.5M_{bbAA}(i-1)(1-\Phi-\eta)F_{BBAa}(i-1) + M_{bbAA}(i-1)\Phi F_{BBAa}(i-1) + 0.5M_{bbAA}(i-1)\eta F_{BBAa}(i-1)$$
$$+ M_{bbAA}(i-1)F_{BBAA}(i-1) + 0.5M_{bbAA}(i-1)F_{BBAR}(i-1) + 0.125M_{bbAR}(i-1)(1-\Phi-\eta)F_{BbAa}(i-1)$$
$$+ 0.25M_{bbAR}(i-1)\Phi F_{BbAa}(i-1) + 0.125M_{bbAR}(i-1)\eta F_{BbAa}(i-1) + 0.25M_{bbAR}(i-1)F_{BbAA}(i-1)$$
$$+ 0.125M_{bbAR}(i-1)F_{BbAR}(i-1) + 0.25M_{bbAR}(i-1)(1-\Phi-\eta)F_{BBAa}(i-1)$$
$$+ 0.5M_{bbAR}(i-1)\Phi F_{BBAa}(i-1) + 0.25M_{bbAR}(i-1)\eta F_{BBAa}(i-1) + 0.5M_{bbAR}(i-1)F_{BBAA}(i-1)$$
$$+ 0.25M_{bbAR}(i-1)F_{BBAR}(i-1) + 0.125(1-\Phi-\eta)M_{BbAa}(i-1)F_{bbAa}(i-1)$$
$$+ 0.25\Phi M_{BbAa}(i-1)F_{bbAa}(i-1) + 0.125\eta M_{BbAa}(i-1)F_{bbAa}(i-1)$$
$$+ 0.25(1-\Phi-\eta)M_{BbAa}(i-1)F_{bbAA}(i-1) + 0.5\Phi M_{BbAa}(i-1)F_{bbAA}(i-1)$$
$$+ 0.25\eta M_{BbAa}(i-1)F_{bbAA}(i-1) + 0.125(1-\Phi-\eta)M_{BbAa}(i-1)F_{bbAR}(i-1)$$
$$+ 0.25\Phi M_{BbAa}(i-1)F_{bbAR}(i-1) + 0.125\eta M_{BbAa}(i-1)F_{bbAR}(i-1)$$
$$+ 0.125(1-\Phi-\eta)M_{BbAa}(i-1)(1-\Phi-\eta)F_{BbAa}(i-1) + 0.25\Phi M_{BbAa}(i-1)(1-\Phi-\eta)F_{BbAa}(i-1)$$
$$+ 0.125\eta M_{BbAa}(i-1)(1-\Phi-\eta)F_{BbAa}(i-1) + 0.25(1-\Phi-\eta)M_{BbAa}(i-1)\Phi F_{BbAa}(i-1)$$
$$+ 0.125(1-\Phi-\eta)M_{BbAa}(i-1)\eta F_{BbAa}(i-1) + 0.5\Phi M_{BbAa}(i-1)\Phi F_{BbAa}(i-1)$$
$$+ 0.25\Phi M_{BbAa}(i-1)\eta F_{BbAa}(i-1) + 0.25\eta M_{BbAa}(i-1)\Phi F_{BbAa}(i-1) + 0.125\eta M_{BbAa}(i-1)\eta F_{BbAa}(i-1)$$
$$+ 0.25(1-\Phi-\eta)M_{BbAa}(i-1)F_{BbAA}(i-1) + 0.5\Phi M_{BbAa}(i-1)F_{BbAA}(i-1)$$
$$+ 0.25\eta M_{BbAa}(i-1)F_{BbAA}(i-1) + 0.125(1-\Phi-\eta)M_{BbAa}(i-1)F_{BbAR}(i-1)$$
$$+ 0.25\Phi M_{BbAa}(i-1)F_{BbAR}(i-1) + 0.125\eta M_{BbAa}(i-1)F_{BbAR}(i-1)$$
$$+ 0.125(1-\Phi-\eta)M_{BbAa}(i-1)(1-\Phi-\eta)F_{BBAa}(i-1) + 0.25\Phi M_{BbAa}(i-1)(1-\Phi-\eta)F_{BBAa}(i-1)$$
$$+ 0.125\eta M_{BbAa}(i-1)(1-\Phi-\eta)F_{BBAa}(i-1) + 0.25(1-\Phi-\eta)M_{BbAa}(i-1)\Phi F_{BBAa}(i-1)$$
$$+ 0.125(1-\Phi-\eta)M_{BbAa}(i-1)\eta F_{BBAa}(i-1) + 0.5\Phi M_{BbAa}(i-1)\Phi F_{BBAa}(i-1)$$
$$+ 0.25\Phi M_{BbAa}(i-1)\eta F_{BBAa}(i-1) + 0.25\eta M_{BbAa}(i-1)\Phi F_{BBAa}(i-1)$$
$$+ 0.125\eta M_{BbAa}(i-1)\eta F_{BBAa}(i-1) + 0.25(1-\Phi-\eta)M_{BbAa}(i-1)F_{BBAA}(i-1)$$
$$+ 0.5\Phi M_{BbAa}(i-1)F_{BBAA}(i-1) + 0.25\eta M_{BbAa}(i-1)F_{BBAA}(i-1)$$
$$+ 0.125(1-\Phi-\eta)M_{BbAa}(i-1)F_{BBAR}(i-1) + 0.25\Phi M_{BbAa}(i-1)F_{BBAR}(i-1)$$
$$+ 0.125\eta M_{BbAa}(i-1)F_{BBAR}(i-1) + 0.25M_{BbAA}(i-1)F_{bbAa}(i-1) + 0.5M_{BbAA}(i-1)F_{bbAA}(i-1)$$
$$+ 0.25M_{BbAA}(i-1)F_{bbAR}(i-1) + 0.25M_{BbAA}(i-1)(1-\Phi-\eta)F_{BbAa}(i-1)$$
$$+ 0.5M_{BbAA}(i-1)\Phi F_{BbAa}(i-1) + 0.25M_{BbAA}(i-1)\eta F_{BbAa}(i-1) + 0.5M_{BbAA}(i-1)F_{BbAA}(i-1)$$
$$+ 0.25M_{BbAA}(i-1)F_{BbAR}(i-1) + 0.25M_{BbAA}(i-1)(1-\Phi-\eta)F_{BBAa}(i-1)$$
$$+ 0.5M_{BbAA}(i-1)\Phi F_{BBAa}(i-1) + 0.25M_{BbAA}(i-1)\eta F_{BBAa}(i-1) + 0.5M_{BbAA}(i-1)F_{BBAA}(i-1)$$
$$+ 0.25M_{BbAA}(i-1)F_{BBAR}(i-1) + 0.125M_{BbAR}(i-1)F_{bbAa}(i-1) + 0.25M_{BbAR}(i-1)F_{bbAA}(i-1)$$
$$+ 0.125M_{BbAR}(i-1)F_{bbAR}(i-1) + 0.125M_{BbAR}(i-1)(1-\Phi-\eta)F_{BbAa}(i-1)$$
$$+ 0.25M_{BbAR}(i-1)\Phi F_{BbAa}(i-1) + 0.125M_{BbAR}(i-1)\eta F_{BbAa}(i-1) + 0.25M_{BbAR}(i-1)F_{BbAA}(i-1)$$
$$+ 0.125M_{BbAR}(i-1)F_{BbAR}(i-1) + 0.125M_{BbAR}(i-1)(1-\Phi-\eta)F_{BBAa}(i-1)$$
$$+ 0.25M_{BbAR}(i-1)\Phi F_{BBAa}(i-1) + 0.125M_{BbAR}(i-1)\eta F_{BBAa}(i-1) + 0.25M_{BbAR}(i-1)F_{BBAA}(i-1)$$
$$+ 0.125M_{BbAR}(i-1)F_{BBAR}(i-1) + 0.25(1-\Phi-\eta)M_{BBAa}(i-1)F_{bbAa}(i-1)$$
$$+ 0.5\Phi M_{BBAa}(i-1)F_{bbAa}(i-1) + 0.25\eta M_{BBAa}(i-1)F_{bbAa}(i-1)$$
$$+ 0.5(1-\Phi-\eta)M_{BBAa}(i-1)F_{bbAA}(i-1) + \Phi M_{BBAa}(i-1)F_{bbAA}(i-1) + 0.5\eta M_{BBAa}(i-1)F_{bbAA}(i-1)$$

$$+ 0.25(1-\Phi-\eta)M_{BBAa}(i-1)F_{bbAR}(i-1) + 0.5\Phi M_{BBAa}(i-1)F_{bbAR}(i-1)$$
$$+ 0.25\eta M_{BBAa}(i-1)F_{bbAR}(i-1) + 0.125(1-\Phi-\eta)M_{BBAa}(i-1)(1-\Phi-\eta)F_{BbAa}(i-1)$$
$$+ 0.25\Phi M_{BBAa}(i-1)(1-\Phi-\eta)F_{BbAa}(i-1) + 0.125\eta M_{BBAa}(i-1)(1-\Phi-\eta)F_{BbAa}(i-1)$$
$$+ 0.25(1-\Phi-\eta)M_{BBAa}(i-1)\Phi F_{BbAa}(i-1) + 0.125(1-\Phi-\eta)M_{BBAa}(i-1)\eta F_{BbAa}(i-1)$$
$$+ 0.5\Phi M_{BBAa}(i-1)\Phi F_{BbAa}(i-1) + 0.25\Phi M_{BBAa}(i-1)\eta F_{BbAa}(i-1)$$
$$+ 0.25\eta M_{BBAa}(i-1)\Phi F_{BbAa}(i-1) + 0.125\eta M_{BBAa}(i-1)\eta F_{BbAa}(i-1)$$
$$+ 0.25(1-\Phi-\eta)M_{BBAa}(i-1)F_{BbAA}(i-1) + 0.5\Phi M_{BBAa}(i-1)F_{BbAA}(i-1)$$
$$+ 0.25\eta M_{BBAa}(i-1)F_{BbAA}(i-1) + 0.125(1-\Phi-\eta)M_{BBAa}(i-1)F_{BbAR}(i-1)$$
$$+ 0.25\Phi M_{BBAa}(i-1)F_{BbAR}(i-1) + 0.125\eta M_{BBAa}(i-1)F_{BbAR}(i-1) + 0.5M_{BBAA}(i-1)F_{bbAa}(i-1)$$
$$+ M_{BBAA}(i-1)F_{bbAA}(i-1) + 0.5M_{BBAA}(i-1)F_{bbAR}(i-1) + 0.25M_{BBAA}(i-1)(1-\Phi-\eta)F_{BbAa}(i-1)$$
$$+ 0.5M_{BBAA}(i-1)\Phi F_{BbAa}(i-1) + 0.25M_{BBAA}(i-1)\eta F_{BbAa}(i-1) + 0.5M_{BBAA}(i-1)F_{BbAA}(i-1)$$
$$+ 0.25M_{BBAA}(i-1)F_{BbAR}(i-1) + 0.25M_{BBAR}(i-1)F_{bbAa}(i-1) + 0.5M_{BBAR}(i-1)F_{bbAA}(i-1)$$
$$+ 0.25M_{BBAR}(i-1)F_{bbAR}(i-1) + 0.125M_{BBAR}(i-1)(1-\Phi-\eta)F_{BbAa}(i-1)$$
$$+ 0.25M_{BBAR}(i-1)\Phi F_{BbAa}(i-1) + 0.125M_{BBAR}(i-1)\eta F_{BbAa}(i-1) + 0.25M_{BBAR}(i-1)F_{BbAA}(i-1)$$
$$+ 0.125M_{BBAR}(i-1)F_{BbAR}(i-1)],$$

$$M^e_{BbAR} = \left(\frac{\Omega_{BbAR}}{2}\right)[0.125M_{bbAa}(i-1)\eta F_{BbAa}(i-1) + 0.125M_{bbAa}(i-1)F_{BbRa}(i-1)$$
$$+ 0.125M_{bbAa}(i-1)F_{BbAR}(i-1) + 0.25M_{bbAa}(i-1)F_{BbRR}(i-1) + 0.25M_{bbAa}(i-1)\eta F_{BBAa}(i-1)$$
$$+ 0.25M_{bbAa}(i-1)F_{BBRa}(i-1) + 0.25M_{bbAa}(i-1)F_{BBAR}(i-1) + 0.5M_{bbAa}(i-1)F_{BBRR}(i-1)$$
$$+ 0.125M_{bbRa}(i-1)(1-\Phi-\eta)F_{BbAa}(i-1) + 0.25M_{bbRa}(i-1)\Phi F_{BbAa}(i-1)$$
$$+ 0.125M_{bbRa}(i-1)\eta F_{BbAa}(i-1) + 0.25M_{bbRa}(i-1)F_{BbAA}(i-1) + 0.125M_{bbRa}(i-1)F_{BbAR}(i-1)$$
$$+ 0.25M_{bbRa}(i-1)(1-\Phi-\eta)F_{BBAa}(i-1) + 0.5M_{bbRa}(i-1)\Phi F_{BBAa}(i-1)$$
$$+ 0.25M_{bbRa}(i-1)\eta F_{BBAa}(i-1) + 0.5M_{bbRa}(i-1)F_{BBAA}(i-1) + 0.25M_{bbRa}(i-1)F_{BBAR}(i-1)$$
$$+ 0.25M_{bbAA}(i-1)\eta F_{BbAa}(i-1) + 0.25M_{bbAA}(i-1)F_{BbRa}(i-1) + 0.25M_{bbAA}(i-1)F_{BbAR}(i-1)$$
$$+ 0.5M_{bbAA}(i-1)F_{BbRR}(i-1) + 0.5M_{bbAA}(i-1)\eta F_{BBAa}(i-1) + 0.5M_{bbAA}(i-1)F_{BBRa}(i-1)$$
$$+ 0.5M_{bbAA}(i-1)F_{BBAR}(i-1) + M_{bbAA}(i-1)F_{BBRR}(i-1) + 0.125M_{bbAR}(i-1)(1-\Phi-\eta)F_{BbAa}(i-1)$$
$$+ 0.25M_{bbAR}(i-1)\Phi F_{BbAa}(i-1) + 0.25M_{bbAR}(i-1)\eta F_{BbAa}(i-1) + 0.125M_{bbAR}(i-1)F_{BbRa}(i-1)$$
$$+ 0.25M_{bbAR}(i-1)F_{BbAA}(i-1) + 0.25M_{bbAR}(i-1)F_{BbAR}(i-1) + 0.25M_{bbAR}(i-1)F_{BbRR}(i-1)$$
$$+ 0.25M_{bbAR}(i-1)(1-\Phi-\eta)F_{BBAa}(i-1) + 0.5M_{bbAR}(i-1)\Phi F_{BBAa}(i-1)$$
$$+ 0.5M_{bbAR}(i-1)\eta F_{BBAa}(i-1) + 0.25M_{bbAR}(i-1)F_{BBRa}(i-1) + 0.5M_{bbAR}(i-1)F_{BBAA}(i-1)$$
$$+ 0.5M_{bbAR}(i-1)F_{BBAR}(i-1) + 0.5M_{bbAR}(i-1)F_{BBRR}(i-1) + 0.25M_{bbRR}(i-1)(1-\Phi-\eta)F_{BbAa}(i-1)$$
$$+ 0.5M_{bbRR}(i-1)\Phi F_{BbAa}(i-1) + 0.25M_{bbRR}(i-1)\eta F_{BbAa}(i-1) + 0.5M_{bbRR}(i-1)F_{BbAA}(i-1)$$
$$+ 0.25M_{bbRR}(i-1)F_{BbAR}(i-1) + 0.5M_{bbRR}(i-1)(1-\Phi-\eta)F_{BBAa}(i-1) + M_{bbRR}(i-1)\Phi F_{BBAa}(i-1)$$
$$+ 0.5M_{bbRR}(i-1)\eta F_{BBAa}(i-1) + M_{bbRR}(i-1)F_{BBAA}(i-1) + 0.5M_{bbRR}(i-1)F_{BBAR}(i-1)$$
$$+ 0.125\eta M_{BbAa}(i-1)F_{bbAa}(i-1) + 0.125(1-\Phi-\eta)M_{BbAa}(i-1)F_{bbRa}(i-1)$$
$$+ 0.25\Phi M_{BbAa}(i-1)F_{bbRa}(i-1) + 0.125\eta M_{BbAa}(i-1)F_{bbRa}(i-1) + 0.25\eta M_{BbAa}(i-1)F_{bbAA}(i-1)$$
$$+ 0.125(1-\Phi-\eta)M_{BbAa}(i-1)F_{bbAR}(i-1) + 0.25\Phi M_{BbAa}(i-1)F_{bbAR}(i-1)$$
$$+ 0.25\eta M_{BbAa}(i-1)F_{bbAR}(i-1) + 0.25(1-\Phi-\eta)M_{BbAa}(i-1)F_{bbRR}(i-1)$$
$$+ 0.5\Phi M_{BbAa}(i-1)F_{bbRR}(i-1) + 0.25\eta M_{BbAa}(i-1)F_{bbRR}(i-1)$$
$$+ 0.125\eta M_{BbAa}(i-1)(1-\Phi-\eta)F_{BbAa}(i-1) + 0.125(1-\Phi-\eta)M_{BbAa}(i-1)\eta F_{BbAa}(i-1)$$
$$+ 0.25\Phi M_{BbAa}(i-1)\eta F_{BbAa}(i-1) + 0.25\eta M_{BbAa}(i-1)\Phi F_{BbAa}(i-1) + 0.25\eta M_{BbAa}(i-1)\eta F_{BbAa}(i-1)$$
$$+ 0.125(1-\Phi-\eta)M_{BbAa}(i-1)F_{BbRa}(i-1) + 0.25\Phi M_{BbAa}(i-1)F_{BbRa}(i-1)$$
$$+ 0.125\eta M_{BbAa}(i-1)F_{BbRa}(i-1) + 0.25\eta M_{BbAa}(i-1)F_{BbAA}(i-1)$$
$$+ 0.125(1-\Phi-\eta)M_{BbAa}(i-1)F_{BbAR}(i-1) + 0.25\Phi M_{BbAa}(i-1)F_{BbAR}(i-1)$$
$$+ 0.25\eta M_{BbAa}(i-1)F_{BbAR}(i-1) + 0.25(1-\Phi-\eta)M_{BbAa}(i-1)F_{BbRR}(i-1)$$
$$+ 0.5\Phi M_{BbAa}(i-1)F_{BbRR}(i-1) + 0.25\eta M_{BbAa}(i-1)F_{BbRR}(i-1)$$
$$+ 0.125\eta M_{BbAa}(i-1)(1-\Phi-\eta)F_{BBAa}(i-1) + 0.125(1-\Phi-\eta)M_{BbAa}(i-1)\eta F_{BBAa}(i-1)$$

$$+ 0.25\Phi M_{BbAa}(i-1)\eta F_{BBAa}(i-1) + 0.25\eta M_{BbAa}(i-1)\Phi F_{BBAa}(i-1)$$
$$+ 0.25\eta M_{BbAa}(i-1)\eta F_{BBAa}(i-1) + 0.125(1-\Phi-\eta) M_{BbAa}(i-1) F_{BBRa}(i-1)$$
$$+ 0.25\Phi M_{BbAa}(i-1)F_{BBRa}(i-1) + 0.125\eta M_{BbAa}(i-1)F_{BBRa}(i-1) + 0.25\eta M_{BbAa}(i-1)F_{BBAA}(i-1)$$
$$+ 0.125(1-\Phi-\eta)M_{BbAa}(i-1)F_{BBAR}(i-1) + 0.25\Phi M_{BbAa}(i-1)F_{BBAR}(i-1)$$
$$+ 0.25\eta M_{BbAa}(i-1)F_{BBAR}(i-1) + 0.25(1-\Phi-\eta)M_{BbAa}(i-1)F_{BBRR}(i-1)$$
$$+ 0.5\Phi M_{BbAa}(i-1)F_{BBRR}(i-1) + 0.25\eta M_{BbAa}(i-1)F_{BBRR}(i-1) + 0.125 M_{BbRa}(i-1)F_{bbAa}(i-1)$$
$$+ 0.25M_{BbRa}(i-1)F_{bbAA}(i-1) + 0.125M_{BbRa}(i-1)F_{bbAR}(i-1)$$
$$+ 0.125M_{BbRa}(i-1)(1-\Phi-\eta)F_{BbAa}(i-1) + 0.25M_{BbRa}(i-1)\Phi F_{BbAa}(i-1)$$
$$+ 0.125M_{BbRa}(i-1)\eta F_{BbAa}(i-1) + 0.25M_{BbRa}(i-1)F_{BbAA}(i-1) + 0.125M_{BbRa}(i-1)F_{BbAR}(i-1)$$
$$+ 0.125M_{BbRa}(i-1)(1-\Phi-\eta)F_{BBAa}(i-1) + 0.25M_{BbRa}(i-1)\Phi F_{BBAa}(i-1)$$
$$+ 0.125M_{BbRa}(i-1)\eta F_{BBAa}(i-1) + 0.25M_{BbRa}(i-1)F_{BBAA}(i-1) + 0.125M_{BbRa}(i-1)F_{BBAR}(i-1)$$
$$+ 0.25M_{BbAA}(i-1)F_{bbRa}(i-1) + 0.25M_{BbAA}(i-1)F_{bbAR}(i-1) + 0.5M_{BbAA}(i-1)F_{bbRR}(i-1)$$
$$+ 0.25M_{BbAA}(i-1)\eta F_{BbAa}(i-1) + 0.25M_{BbAA}(i-1)F_{BbRa}(i-1) + 0.25M_{BbAA}(i-1)F_{BbAR}(i-1)$$
$$+ 0.5M_{BbAA}(i-1)F_{BbRR}(i-1) + 0.25M_{BbAA}(i-1)\eta F_{BBAa}(i-1) + 0.25M_{BbAA}(i-1)F_{BBRa}(i-1)$$
$$+ 0.25M_{BbAA}(i-1)F_{BBAR}(i-1) + 0.5M_{BbAA}(i-1)F_{BBRR}(i-1) + 0.125M_{BbAR}(i-1)F_{bbAa}(i-1)$$
$$+ 0.125M_{BbAR}(i-1)F_{bbRa}(i-1) + 0.25M_{BbAR}(i-1)F_{bbAA}(i-1) + 0.25M_{BbAR}(i-1)F_{bbAR}(i-1)$$
$$+ 0.25M_{BbAR}(i-1)F_{bbRR}(i-1) + 0.125M_{BbAR}(i-1)(1-\Phi-\eta)F_{BbAa}(i-1)$$
$$+ 0.25M_{BbAR}(i-1)\Phi F_{BbAa}(i-1) + 0.25M_{BbAR}(i-1)\eta F_{BbAa}(i-1) + 0.125M_{BbAR}(i-1)F_{BbRa}(i-1)$$
$$+ 0.25M_{BbAR}(i-1)F_{BbAA}(i-1) + 0.25M_{BbAR}(i-1)F_{BbAR}(i-1) + 0.25M_{BbAR}(i-1)F_{BbRR}(i-1)$$
$$+ 0.125M_{BbAR}(i-1)(1-\Phi-\eta)F_{BBAa}(i-1) + 0.25M_{BbAR}(i-1)\Phi F_{BBAa}(i-1)$$
$$+ 0.25M_{BbAR}(i-1)\eta F_{BBAa}(i-1) + 0.125M_{BbAR}(i-1)F_{BBRa}(i-1) + 0.25M_{BbAR}(i-1)F_{BBAA}(i-1)$$
$$+ 0.25M_{BbAR}(i-1)F_{BBAR}(i-1) + 0.25M_{BbAR}(i-1)F_{BBRR}(i-1) + 0.25M_{BbRR}(i-1)F_{bbAa}(i-1)$$
$$+ 0.5M_{BbRR}(i-1)F_{bbAA}(i-1) + 0.25M_{BbRR}(i-1)F_{bbAR}(i-1) + 0.25M_{BbRR}(i-1)(1-\Phi-\eta)F_{BbAa}(i-1)$$
$$+ 0.5M_{BbRR}(i-1)\Phi F_{BbAa}(i-1) + 0.25M_{BbRR}(i-1)\eta F_{BbAa}(i-1) + 0.5M_{BbRR}(i-1)F_{BbAA}(i-1)$$
$$+ 0.25M_{BbRR}(i-1)F_{BbAR}(i-1) + 0.25M_{BbRR}(i-1)(1-\Phi-\eta)F_{BBAa}(i-1)$$
$$+ 0.5M_{BbRR}(i-1)\Phi F_{BBAa}(i-1) + 0.25M_{BbRR}(i-1)\eta F_{BBAa}(i-1) + 0.5M_{BbRR}(i-1)F_{BBAA}(i-1)$$
$$+ 0.25M_{BbRR}(i-1)F_{BBAR}(i-1) + 0.25\eta M_{BBAa}(i-1)F_{bbAa}(i-1)$$
$$+ 0.25(1-\Phi-\eta)M_{BBAa}(i-1)F_{bbRa}(i-1) + 0.5\Phi M_{BBAa}(i-1)F_{bbRa}(i-1)$$
$$+ 0.25\eta M_{BBAa}(i-1)F_{bbRa}(i-1) + 0.5\eta M_{BBAa}(i-1)F_{bbAA}(i-1)$$
$$+ 0.25(1-\Phi-\eta)M_{BBAa}(i-1)F_{bbAR}(i-1) + 0.5\Phi M_{BBAa}(i-1)F_{bbAR}(i-1)$$
$$+ 0.5\eta M_{BBAa}(i-1)F_{bbAR}(i-1) + 0.5(1-\Phi-\eta)M_{BBAa}(i-1)F_{bbRR}(i-1) + \Phi M_{BBAa}(i-1)F_{bbRR}(i-1)$$
$$+ 0.5\eta M_{BBAa}(i-1)F_{bbRR}(i-1) + 0.125\eta M_{BBAa}(i-1)(1-\Phi-\eta)F_{BbAa}(i-1)$$
$$+ 0.125(1-\Phi-\eta)M_{BBAa}(i-1)\eta F_{BbAa}(i-1) + 0.25\Phi M_{BBAa}(i-1)\eta F_{BbAa}(i-1)$$
$$+ 0.25\eta M_{BBAa}(i-1)\Phi F_{BbAa}(i-1) + 0.25\eta M_{BBAa}(i-1)\eta F_{BbAa}(i-1)$$
$$+ 0.125(1-\Phi-\eta)M_{BBAa}(i-1)F_{BbRa}(i-1) + 0.25\Phi M_{BBAa}(i-1)F_{BbRa}(i-1)$$
$$+ 0.125\eta M_{BBAa}(i-1)F_{BbRa}(i-1) + 0.25\eta M_{BBAa}(i-1)F_{BbAA}(i-1)$$
$$+ 0.125(1-\Phi-\eta)M_{BBAa}(i-1)F_{BbAR}(i-1) + 0.25\Phi M_{BBAa}(i-1)F_{BbAR}(i-1)$$
$$+ 0.25\eta M_{BBAa}(i-1)F_{BbAR}(i-1) + 0.25(1-\Phi-\eta)M_{BBAa}(i-1)F_{BbRR}(i-1)$$
$$+ 0.5\Phi M_{BBAa}(i-1)F_{BbRR}(i-1) + 0.25\eta M_{BBAa}(i-1)F_{BbRR}(i-1) + 0.25M_{BBRa}(i-1)F_{bbAa}(i-1)$$
$$+ 0.5M_{BBRa}(i-1)F_{bbAA}(i-1) + 0.25M_{BBRa}(i-1)F_{bbAR}(i-1) + 0.125M_{BBRa}(i-1)(1-\Phi-\eta)F_{BbAa}(i-1$$
$$+ 0.125M_{BBRa}(i-1)\eta F_{BbAa}(i-1) + 0.25M_{BBRa}(i-1)F_{BbAA}(i-1) + 0.125M_{BBRa}(i-1)F_{BbAR}(i-1)$$
$$+ 0.5M_{BBAA}(i-1)F_{bbRa}(i-1) + 0.5M_{BBAA}(i-1)F_{bbAR}(i-1) + M_{BBAA}(i-1)F_{bbRR}(i-1)$$
$$+ 0.25M_{BBAA}(i-1)\eta F_{BbAa}(i-1) + 0.25M_{BBAA}(i-1)F_{BbRa}(i-1) + 0.25M_{BBAA}(i-1)F_{BbAR}(i-1)$$
$$+ 0.5M_{BBAA}(i-1)F_{BbRR}(i-1) + 0.25M_{BBAR}(i-1)F_{bbAa}(i-1) + 0.25M_{BBAR}(i-1)F_{bbRa}(i-1)$$
$$+ 0.5M_{BBAR}(i-1)F_{bbAA}(i-1) + 0.5M_{BBAR}(i-1)F_{bbAR}(i-1) + 0.5M_{BBAR}(i-1)F_{bbRR}(i-1)$$
$$+ 0.125M_{BBAR}(i-1)(1-\Phi-\eta)F_{BbAa}(i-1) + 0.25M_{BBAR}(i-1)\Phi F_{BbAa}(i-1)$$
$$+ 0.25M_{BBAR}(i-1)\eta F_{BbAa}(i-1) + 0.125M_{BBAR}(i-1)F_{BbRa}(i-1) + 0.25M_{BBAR}(i-1)F_{BbAA}(i-1)$$

$$
\begin{aligned}
&+0.25M_{BBAR}(i-1)F_{BbAR}(i-1)+0.25M_{BBAR}(i-1)F_{BbRR}(i-1)+0.5M_{BBRR}(i-1)F_{bbAa}(i-1)\\
&+M_{BBRR}(i-1)F_{bbAA}(i-1)+0.5M_{BBRR}(i-1)F_{bbAR}(i-1)+0.25M_{BBRR}(i-1)(1-\Phi-\eta)F_{BbAa}(i-1)\\
&+0.5M_{BBRR}(i-1)\Phi F_{BbAa}(i-1)+0.25M_{BBRR}(i-1)\eta F_{BbAa}(i-1)+0.5M_{BBRR}(i-1)F_{BbAA}(i-1)\\
&+0.25M_{BBRR}(i-1)F_{BbAR}(i-1)],\\
M^{e}_{BbRR}&=(\Omega_{BbRR}/2)[0.125M_{bbRa}(i-1)\eta F_{BbAa}(i-1)+0.125M_{bbRa}(i-1)F_{BbRa}(i-1)\\
&+0.125M_{bbRa}(i-1)F_{BbAR}(i-1)+0.25M_{bbRa}(i-1)F_{BbRR}(i-1)+0.25M_{bbRa}(i-1)\eta F_{BBAa}(i-1)\\
&+0.25M_{bbRa}(i-1)F_{BBRa}(i-1)+0.25M_{bbRa}(i-1)F_{BBAR}(i-1)+0.5M_{bbRa}(i-1)F_{BBRR}(i-1)\\
&+0.125M_{bbAR}(i-1)\eta F_{BbAa}(i-1)+0.125M_{bbAR}(i-1)F_{BbRa}(i-1)+0.125M_{bbAR}(i-1)F_{BbAR}(i-1)\\
&+0.25M_{bbAR}(i-1)F_{BbRR}(i-1)+0.25M_{bbAR}(i-1)\eta F_{BBAa}(i-1)+0.25M_{bbAR}(i-1)F_{BBRa}(i-1)\\
&+0.25M_{bbAR}(i-1)F_{BBAR}(i-1)+0.5M_{bbAR}(i-1)F_{BBRR}(i-1)+0.25M_{bbRR}(i-1)\eta F_{BbAa}(i-1)\\
&+0.25M_{bbRR}(i-1)F_{BbRa}(i-1)+0.25M_{bbRR}(i-1)F_{BbAR}(i-1)+0.5M_{bbRR}(i-1)F_{BbRR}(i-1)\\
&+0.5M_{bbRR}(i-1)\eta F_{BBAa}(i-1)+0.5M_{bbRR}(i-1)F_{BBRa}(i-1)+0.5M_{bbRR}(i-1)F_{BBAR}(i-1)\\
&+M_{bbRR}(i-1)F_{BBRR}(i-1)+0.125\eta M_{BbAa}(i-1)F_{bbRa}(i-1)+0.125\eta M_{BbAa}(i-1)F_{bbAR}(i-1)\\
&+0.25\eta M_{BbAa}(i-1)F_{bbRR}(i-1)+0.125\eta M_{BbAa}(i-1)\eta F_{BbAa}(i-1)+0.125\eta M_{BbAa}(i-1)F_{BbRa}(i-1)\\
&+0.125\eta M_{BbAa}(i-1)F_{BbAR}(i-1)+0.25\eta M_{BbAa}(i-1)F_{BbRR}(i-1)+0.125\eta M_{BbAa}(i-1)\eta F_{BBAa}(i-1)\\
&+0.125\eta M_{BbAa}(i-1)F_{BBRa}(i-1)+0.125\eta M_{BbAa}(i-1)F_{BBAR}(i-1)+0.25\eta M_{BbAa}(i-1)F_{BBRR}(i-1)\\
&+0.125M_{BbRa}(i-1)F_{bbRa}(i-1)+0.125M_{BbRa}(i-1)F_{bbAR}(i-1)+0.25M_{BbRa}(i-1)F_{bbRR}(i-1)\\
&+0.125M_{BbRa}(i-1)\eta F_{BbAa}(i-1)+0.125M_{BbRa}(i-1)F_{BbRa}(i-1)+0.125M_{BbRa}(i-1)F_{BbAR}(i-1)\\
&+0.25M_{BbRa}(i-1)F_{BbRR}(i-1)+0.125M_{BbRa}(i-1)\eta F_{BBAa}(i-1)+0.125M_{BbRa}(i-1)F_{BBRa}(i-1)\\
&+0.125M_{BbRa}(i-1)F_{BBAR}(i-1)+0.25M_{BbRa}(i-1)F_{BBRR}(i-1)+0.125M_{BbAR}(i-1)F_{bbRa}(i-1)\\
&+0.125M_{BbAR}(i-1)F_{bbAR}(i-1)+0.25M_{BbAR}(i-1)F_{bbRR}(i-1)+0.125M_{BbAR}(i-1)\eta F_{BbAa}(i-1)\\
&+0.125M_{BbAR}(i-1)F_{BbRa}(i-1)+0.125M_{BbAR}(i-1)F_{BbAR}(i-1)+0.25M_{BbAR}(i-1)F_{BbRR}(i-1)\\
&+0.125M_{BbAR}(i-1)\eta F_{BBAa}(i-1)+0.125M_{BbAR}(i-1)F_{BBRa}(i-1)+0.125M_{BbAR}(i-1)F_{BBAR}(i-1)\\
&+0.25M_{BbAR}(i-1)F_{BBRR}(i-1)+0.25M_{BbRR}(i-1)F_{bbRa}(i-1)+0.25M_{BbRR}(i-1)F_{bbAR}(i-1)\\
&+0.5M_{BbRR}(i-1)F_{bbRR}(i-1)+0.25M_{BbRR}(i-1)\eta F_{BbAa}(i-1)+0.25M_{BbRR}(i-1)F_{BbRa}(i-1)\\
&+0.25M_{BbRR}(i-1)F_{BbAR}(i-1)+0.5M_{BbRR}(i-1)F_{BbRR}(i-1)+0.25M_{BbRR}(i-1)\eta F_{BBAa}(i-1)\\
&+0.25M_{BbRR}(i-1)F_{BBRa}(i-1)+0.25M_{BbRR}(i-1)F_{BBAR}(i-1)+0.5M_{BbRR}(i-1)F_{BBRR}(i-1)\\
&+0.25\eta M_{BBAa}(i-1)F_{bbRa}(i-1)+0.25\eta M_{BBAa}(i-1)F_{bbAR}(i-1)+0.5\eta M_{BBAa}(i-1)F_{bbRR}(i-1)\\
&+0.125\eta M_{BBAa}(i-1)\eta F_{BbAa}(i-1)+0.125\eta M_{BBAa}(i-1)F_{BbRa}(i-1)+0.125\eta M_{BBAa}(i-1)F_{BbAR}(i-1)\\
&+0.25\eta M_{BBAa}(i-1)F_{BbRR}(i-1)+0.25M_{BBRa}(i-1)F_{bbRa}(i-1)+0.25M_{BBRa}(i-1)F_{bbAR}(i-1)\\
&+0.5M_{BBRa}(i-1)F_{bbRR}(i-1)+0.125M_{BBRa}(i-1)\eta F_{BbAa}(i-1)+0.125M_{BBRa}(i-1)F_{BbRa}(i-1)\\
&+0.125M_{BBRa}(i-1)F_{BbAR}(i-1)+0.25M_{BBRa}(i-1)F_{BbRR}(i-1)+0.25M_{BBAR}(i-1)F_{bbRa}(i-1)\\
&+0.25M_{BBAR}(i-1)F_{bbAR}(i-1)+0.5M_{BBAR}(i-1)F_{bbRR}(i-1)+0.125M_{BBAR}(i-1)\eta F_{BbAa}(i-1)\\
&+0.125M_{BBAR}(i-1)F_{BbRa}(i-1)+0.125M_{BBAR}(i-1)F_{BbAR}(i-1)+0.25M_{BBAR}(i-1)F_{BbRR}(i-1)\\
&+0.5M_{BBRR}(i-1)F_{bbRa}(i-1)+0.5M_{BBRR}(i-1)F_{bbAR}(i-1)+M_{BBRR}(i-1)F_{bbRR}(i-1)\\
&+0.25M_{BBRR}(i-1)\eta F_{BbAa}(i-1)+0.25M_{BBRR}(i-1)F_{BbRa}(i-1)+0.25M_{BBRR}(i-1)F_{BbAR}(i-1)\\
&+0.5M_{BBRR}(i-1)F_{BbRR}(i-1)],\\
M^{e}_{BBaa}&=(\Omega_{BBaa}/2)[0.25M_{Bbaa}(i-1)F_{Bbaa}(i-1)+0.125M_{Bbaa}(i-1)(1-\Phi-\eta)F_{BbAa}(i-1)\\
&+0.125M_{Bbaa}(i-1)F_{BbRa}(i-1)+0.5M_{Bbaa}(i-1)F_{BBaa}(i-1)\\
&+0.25M_{Bbaa}(i-1)(1-\Phi-\eta)F_{BBAa}(i-1)+0.25M_{Bbaa}(i-1)F_{BBRa}(i-1)\\
&+0.125(1-\Phi-\eta)M_{BbAa}(i-1)F_{Bbaa}(i-1)+0.0625(1-\Phi-\eta)M_{BbAa}(i-1)(1-\Phi-\eta)F_{BbAa}(i-1)\\
&+0.0625(1-\Phi-\eta)M_{BbAa}(i-1)F_{BbRa}(i-1)+0.25(1-\Phi-\eta)M_{BbAa}(i-1)F_{BBaa}(i-1)\\
&+0.125(1-\Phi-\eta)M_{BbAa}(i-1)(1-\Phi-\eta)F_{BBAa}(i-1)+0.125(1-\Phi-\eta)M_{BbAa}(i-1)F_{BBRa}(i-1)\\
&+0.125M_{BbRa}(i-1)F_{Bbaa}(i-1)+0.0625M_{BbRa}(i-1)(1-\Phi-\eta)F_{BbAa}(i-1)\\
&+0.0625M_{BbRa}(i-1)F_{BbRa}(i-1)+0.25M_{BbRa}(i-1)F_{BBaa}(i-1)\\
&+0.125M_{BbRa}(i-1)(1-\Phi-\eta)F_{BBAa}(i-1)+0.125M_{BbRa}(i-1)F_{BBRa}(i-1)\\
&+0.5M_{BBaa}(i-1)F_{Bbaa}(i-1)+0.25M_{BBaa}(i-1)(1-\Phi-\eta)F_{BbAa}(i-1)+0.25M_{BBaa}(i-1)F_{BbRa}(i-1)
\end{aligned}
$$

$$
\begin{aligned}
&+ M_{BBaa}(i-1)F_{BBaa}(i-1) + 0.5M_{BBaa}(i-1)(1-\Phi-\eta)F_{BBAa}(i-1) + 0.5M_{BBaa}(i-1)F_{BBRa}(i-1)\\
&+ 0.25(1-\Phi-\eta)M_{BBAa}(i-1)F_{Bbaa}(i-1) + 0.125(1-\Phi-\eta)M_{BBAa}(i-1)(1-\Phi-\eta)F_{BbAa}(i-1)\\
&+ 0.125(1-\Phi-\eta)M_{BBAa}(i-1)F_{BbRa}(i-1) + 0.5(1-\Phi-\eta)M_{BBAa}(i-1)F_{BBaa}(i-1)\\
&+ 0.25(1-\Phi-\eta)M_{BBAa}(i-1)(1-\Phi-\eta)F_{BBAa}(i-1) + 0.25(1-\Phi-\eta)M_{BBAa}(i-1)F_{BBRa}(i-1)\\
&+ 0.25M_{BBRa}(i-1)F_{Bbaa}(i-1) + 0.125M_{BBRa}(i-1)(1-\Phi-\eta)F_{BbAa}(i-1)\\
&+ 0.125M_{BBRa}(i-1)F_{BbRa}(i-1) + 0.5M_{BBRa}(i-1)F_{BBaa}(i-1)\\
&+ 0.25M_{BBRa}(i-1)(1-\Phi-\eta)F_{BBAa}(i-1) + 0.25M_{BBRa}(i-1)F_{BBRa}(i-1)],
\end{aligned}
$$

$$
\begin{aligned}
M^e_{BBAa} = \left(\frac{\Omega_{BBAa}}{2}\right)&[0.125M_{Bbaa}(i-1)(1-\Phi-\eta)F_{BbAa}(i-1) + 0.25M_{Bbaa}(i-1)\Phi F_{BbAa}(i-1)\\
&+ 0.125M_{Bbaa}(i-1)\eta F_{BbAa}(i-1) + 0.25M_{Bbaa}(i-1)F_{BbAA}(i-1) + 0.125M_{Bbaa}(i-1)F_{BbAR}(i-1)\\
&+ 0.25M_{Bbaa}(i-1)(1-\Phi-\eta)F_{BBAa}(i-1) + 0.5M_{Bbaa}(i-1)\Phi F_{BBAa}(i-1)\\
&+ 0.25M_{Bbaa}(i-1)\eta F_{BBAa}(i-1) + 0.5M_{Bbaa}(i-1)F_{BBAA}(i-1) + 0.25M_{Bbaa}(i-1)F_{BBAR}(i-1)\\
&+ 0.125(1-\Phi-\eta)M_{BbAa}(i-1)F_{Bbaa}(i-1) + 0.25\Phi M_{BbAa}(i-1)F_{Bbaa}(i-1)\\
&+ 0.125\eta M_{BbAa}(i-1)F_{Bbaa}(i-1) + 0.125(1-\Phi-\eta)M_{BbAa}(i-1)(1-\Phi-\eta)F_{BbAa}(i-1)\\
&+ 0.125\Phi M_{BbAa}(i-1)(1-\Phi-\eta)F_{BbAa}(i-1) + 0.0625\eta M_{BbAa}(i-1)(1-\Phi-\eta)F_{BbAa}(i-1)\\
&+ 0.125(1-\Phi-\eta)M_{BbAa}(i-1)\Phi F_{BbAa}(i-1) + 0.0625(1-\Phi-\eta)M_{BbAa}(i-1)\eta F_{BbAa}(i-1)\\
&+ 0.0625(1-\Phi-\eta)M_{BbAa}(i-1)F_{BbRa}(i-1) + 0.125\Phi M_{BbAa}(i-1)F_{BbRa}(i-1)\\
&+ 0.0625\eta M_{BbAa}(i-1)F_{BbRa}(i-1) + 0.125(1-\Phi-\eta)M_{BbAa}(i-1)F_{BbAA}(i-1)\\
&+ 0.0625(1-\Phi-\eta)M_{BbAa}(i-1)F_{BbAR}(i-1) + 0.25(1-\Phi-\eta)M_{BbAa}(i-1)F_{BBaa}(i-1)\\
&+ 0.5\Phi M_{BbAa}(i-1)F_{BBaa}(i-1) + 0.25\eta M_{BbAa}(i-1)F_{BBaa}(i-1)\\
&+ 0.25(1-\Phi-\eta)M_{BbAa}(i-1)(1-\Phi-\eta)F_{BBAa}(i-1) + 0.25\Phi M_{BbAa}(i-1)(1-\Phi-\eta)F_{BBAa}(i-1)\\
&+ 0.125\eta M_{BbAa}(i-1)(1-\Phi-\eta)F_{BBAa}(i-1) + 0.125(1-\Phi-\eta)M_{BbAa}(i-1)\Phi F_{BBAa}(i-1)\\
&+ 0.0625(1-\Phi-\eta)M_{BbAa}(i-1)\eta F_{BBAa}(i-1) + 0.125(1-\Phi-\eta)M_{BbAa}(i-1)F_{BBRa}(i-1)\\
&+ 0.25\Phi M_{BbAa}(i-1)F_{BBRa}(i-1) + 0.125\eta M_{BbAa}(i-1)F_{BBRa}(i-1)\\
&+ 0.25(1-\Phi-\eta)M_{BbAa}(i-1)F_{BBAA}(i-1) + 0.125(1-\Phi-\eta)M_{BbAa}(i-1)F_{BBAR}(i-1)\\
&+ 0.0625M_{BbRa}(i-1)(1-\Phi-\eta)F_{BbAa}(i-1) + 0.125M_{BbRa}(i-1)\Phi F_{BbAa}(i-1)\\
&+ 0.0625M_{BbRa}(i-1)\eta F_{BbAa}(i-1) + 0.125M_{BbRa}(i-1)F_{BbAA}(i-1) + 0.0625M_{BbRa}(i-1)F_{BbAR}(i-1)\\
&+ 0.125M_{BbRa}(i-1)(1-\Phi-\eta)F_{BBAa}(i-1) + 0.25M_{BbRa}(i-1)\Phi F_{BBAa}(i-1)\\
&+ 0.125M_{BbRa}(i-1)\eta F_{BBAa}(i-1) + 0.25M_{BbRa}(i-1)F_{BBAA}(i-1) + 0.125M_{BbRa}(i-1)F_{BBAR}(i-1)\\
&+ 0.25M_{BbAA}(i-1)F_{Bbaa}(i-1) + 0.125M_{BbAA}(i-1)(1-\Phi-\eta)F_{BbAa}(i-1)\\
&+ 0.125M_{BbAA}(i-1)F_{BbRa}(i-1) + 0.5M_{BbAA}(i-1)F_{BBaa}(i-1)\\
&+ 0.25M_{BbAA}(i-1)(1-\Phi-\eta)F_{BBAa}(i-1) + 0.25M_{BbAA}(i-1)F_{BBRa}(i-1)\\
&+ 0.125M_{BbAR}(i-1)F_{Bbaa}(i-1) + 0.0625M_{BbAR}(i-1)(1-\Phi-\eta)F_{BbAa}(i-1)\\
&+ 0.0625M_{BbAR}(i-1)F_{BbRa}(i-1) + 0.25M_{BbAR}(i-1)F_{BBaa}(i-1)\\
&+ 0.125M_{BbAR}(i-1)(1-\Phi-\eta)F_{BBAa}(i-1) + 0.125M_{BbAR}(i-1)F_{BBRa}(i-1)\\
&+ 0.25M_{BBaa}(i-1)(1-\Phi-\eta)F_{BbAa}(i-1) + 0.5M_{BBaa}(i-1)\Phi F_{BbAa}(i-1)\\
&+ 0.25M_{BBaa}(i-1)\eta F_{BbAa}(i-1) + 0.5M_{BBaa}(i-1)F_{BbAA}(i-1) + 0.25M_{BBaa}(i-1)F_{BbAR}(i-1)\\
&+ 0.5M_{BBaa}(i-1)(1-\Phi-\eta)F_{BBAa}(i-1) + M_{BBaa}(i-1)\Phi F_{BBAa}(i-1)\\
&+ 0.5M_{BBaa}(i-1)\eta F_{BBAa}(i-1) + M_{BBaa}(i-1)F_{BBAA}(i-1) + 0.5M_{BBaa}(i-1)F_{BBAR}(i-1)\\
&+ 0.25(1-\Phi-\eta)M_{BBAa}(i-1)F_{Bbaa}(i-1) + 0.5\Phi M_{BBAa}(i-1)F_{Bbaa}(i-1)\\
&+ 0.25\eta M_{BBAa}(i-1)F_{Bbaa}(i-1) + 0.25(1-\Phi-\eta)M_{BBAa}(i-1)(1-\Phi-\eta)F_{BbAa}(i-1)\\
&+ 0.25\Phi M_{BBAa}(i-1)(1-\Phi-\eta)F_{BbAa}(i-1) + 0.125\eta M_{BBAa}(i-1)(1-\Phi-\eta)F_{BbAa}(i-1)\\
&+ 0.25(1-\Phi-\eta)M_{BBAa}(i-1)\Phi F_{BbAa}(i-1) + 0.125(1-\Phi-\eta)M_{BBAa}(i-1)\eta F_{BbAa}(i-1)\\
&+ 0.125(1-\Phi-\eta)M_{BBAa}(i-1)F_{BbRa}(i-1) + 0.25\Phi M_{BBAa}(i-1)F_{BbRa}(i-1)\\
&+ 0.125\eta M_{BBAa}(i-1)F_{BbRa}(i-1) + 0.25(1-\Phi-\eta)M_{BBAa}(i-1)F_{BbAA}(i-1)\\
&+ 0.125(1-\Phi-\eta)M_{BBAa}(i-1)F_{BbAR}(i-1) + 0.5(1-\Phi-\eta)M_{BBAa}(i-1)F_{BBaa}(i-1)\\
&+ \Phi M_{BBAa}(i-1)F_{BBaa}(i-1) + 0.5\eta M_{BBAa}(i-1)F_{BBaa}(i-1)
\end{aligned}
$$

$$
\begin{aligned}
&+0.5(1-\Phi-\eta)M_{BBAa}(i-1)(1-\Phi-\eta)F_{BBAa}(i-1)+0.5\Phi M_{BBAa}(i-1)(1-\Phi-\eta)F_{BBAa}(i-1)\\
&+0.25\eta M_{BBAa}(i-1)(1-\Phi-\eta)F_{BBAa}(i-1)+0.5(1-\Phi-\eta)M_{BBAa}(i-1)\Phi F_{BBAa}(i-1)\\
&+0.25(1-\Phi-\eta)M_{BBAa}(i-1)\eta F_{BBAa}(i-1)+0.25(1-\Phi-\eta)M_{BBAa}(i-1)F_{BBRa}(i-1)\\
&+0.5\Phi M_{BBAa}(i-1)F_{BBRa}(i-1)+0.25\eta M_{BBAa}(i-1)F_{BBRa}(i-1)\\
&+0.5(1-\Phi-\eta)M_{BBAa}(i-1)F_{BBAA}(i-1)+0.25(1-\Phi-\eta)M_{BBAa}(i-1)F_{BBAR}(i-1)\\
&+0.125M_{BBRa}(i-1)(1-\Phi-\eta)F_{BbAa}(i-1)+0.25M_{BBRa}(i-1)\Phi F_{BbAa}(i-1)\\
&+0.125M_{BBRa}(i-1)\eta F_{BbAa}(i-1)+0.25M_{BBRa}(i-1)F_{BbAA}(i-1)+0.125M_{BBRa}(i-1)F_{BbAR}(i-1)\\
&+0.25M_{BBRa}(i-1)(1-\Phi-\eta)F_{BBAa}(i-1)+0.5M_{BBRa}(i-1)\Phi F_{BBAa}(i-1)\\
&+0.25M_{BBRa}(i-1)\eta F_{BBAa}(i-1)+0.5M_{BBRa}(i-1)F_{BBAA}(i-1)+0.25M_{BBRa}(i-1)F_{BBAR}(i-1)\\
&+0.5M_{BBAA}(i-1)F_{Bbaa}(i-1)+0.25M_{BBAA}(i-1)(1-\Phi-\eta)F_{BbAa}(i-1)\\
&+0.25M_{BBAA}(i-1)F_{BbRa}(i-1)+M_{BBAA}(i-1)F_{BBaa}(i-1)+0.5M_{BBAA}(i-1)(1-\Phi-\eta)F_{BBAa}(i-1)\\
&+0.5M_{BBAA}(i-1)F_{BBRa}(i-1)+0.25M_{BBAR}(i-1)F_{Bbaa}(i-1)\\
&+0.125M_{BBAR}(i-1)(1-\Phi-\eta)F_{BbAa}(i-1)+0.125M_{BBAR}(i-1)F_{BbRa}(i-1)\\
&+0.5M_{BBAR}(i-1)F_{BBaa}(i-1)+0.25M_{BBAR}(i-1)(1-\Phi-\eta)F_{BBAa}(i-1)\\
&+0.25M_{BBAR}(i-1)F_{BBRa}(i-1)],\\
M^e_{BBRa}&=(\Omega_{BBRa}/2)[0.125M_{Bbaa}(i-1)\eta F_{BbAa}(i-1)+0.125M_{Bbaa}(i-1)F_{BbRa}(i-1)\\
&+0.125M_{Bbaa}(i-1)F_{BbAR}(i-1)+0.25M_{Bbaa}(i-1)F_{BbRR}(i-1)+0.25M_{Bbaa}(i-1)\eta F_{BBAa}(i-1)\\
&+0.25M_{Bbaa}(i-1)F_{BBRa}(i-1)+0.25M_{Bbaa}(i-1)F_{BBAR}(i-1)+0.5M_{Bbaa}(i-1)F_{BBRR}(i-1)\\
&+0.125\eta M_{BbAa}(i-1)F_{Bbaa}(i-1)+0.0625\eta M_{BbAa}(i-1)(1-\Phi-\eta)F_{BbAa}(i-1)\\
&+0.0625(1-\Phi-\eta)M_{BbAa}(i-1)\eta F_{BbAa}(i-1)+0.0625(1-\Phi-\eta)M_{BbAa}(i-1)F_{BbRa}(i-1)\\
&+0.0625\eta M_{BbAa}(i-1)F_{BbRa}(i-1)+0.0625(1-\Phi-\eta)M_{BbAa}(i-1)F_{BbAR}(i-1)\\
&+0.125(1-\Phi-\eta)M_{BbAa}(i-1)F_{BbRR}(i-1)+0.25\eta M_{BbAa}(i-1)F_{BBaa}(i-1)\\
&+0.125\eta M_{BbAa}(i-1)(1-\Phi-\eta)F_{BBAa}(i-1)+0.0625(1-\Phi-\eta)M_{BbAa}(i-1)\eta F_{BBAa}(i-1)\\
&+0.125(1-\Phi-\eta)M_{BbAa}(i-1)F_{BBRa}(i-1)+0.125\eta M_{BbAa}(i-1)F_{BBRa}(i-1)\\
&+0.125(1-\Phi-\eta)M_{BbAa}(i-1)F_{BBAR}(i-1)+0.25(1-\Phi-\eta)M_{BbAa}(i-1)F_{BBRR}(i-1)\\
&+0.125M_{BbRa}(i-1)F_{Bbaa}(i-1)+0.0625M_{BbRa}(i-1)(1-\Phi-\eta)F_{BbAa}(i-1)\\
&+0.0625M_{BbRa}(i-1)\eta F_{BbAa}(i-1)+0.125M_{BbRa}(i-1)F_{BbRa}(i-1)+0.0625M_{BbRa}(i-1)F_{BbAR}(i-1)\\
&+0.125M_{BbRa}(i-1)F_{BbRR}(i-1)+0.25M_{BbRa}(i-1)F_{BBaa}(i-1)\\
&+0.125M_{BbRa}(i-1)(1-\Phi-\eta)F_{BBAa}(i-1)+0.125M_{BbRa}(i-1)\eta F_{BBAa}(i-1)\\
&+0.25M_{BbRa}(i-1)F_{BBRa}(i-1)+0.125M_{BbRa}(i-1)F_{BBAR}(i-1)+0.25M_{BbRa}(i-1)F_{BBRR}(i-1)\\
&+0.125M_{BbAR}(i-1)F_{Bbaa}(i-1)+0.0625M_{BbAR}(i-1)(1-\Phi-\eta)F_{BbAa}(i-1)\\
&+0.0625M_{BbAR}(i-1)F_{BbRa}(i-1)+0.25M_{BbAR}(i-1)F_{BBaa}(i-1)\\
&+0.125M_{BbAR}(i-1)(1-\Phi-\eta)F_{BBAa}(i-1)+0.125M_{BbAR}(i-1)F_{BBRa}(i-1)\\
&+0.25M_{BbRR}(i-1)F_{Bbaa}(i-1)+0.125M_{BbRR}(i-1)(1-\Phi-\eta)F_{BbAa}(i-1)\\
&+0.125M_{BbRR}(i-1)F_{BbRa}(i-1)+0.5M_{BbRR}(i-1)F_{BBaa}(i-1)\\
&+0.25M_{BbRR}(i-1)(1-\Phi-\eta)F_{BBAa}(i-1)+0.25M_{BbRR}(i-1)F_{BBRa}(i-1)\\
&+0.25M_{BBaa}(i-1)\eta F_{BbAa}(i-1)+0.25M_{BBaa}(i-1)F_{BbRa}(i-1)+0.25M_{BBaa}(i-1)F_{BbAR}(i-1)\\
&+0.5M_{BBaa}(i-1)F_{BbRR}(i-1)+0.5M_{BBaa}(i-1)\eta F_{BBAa}(i-1)+0.5M_{BBaa}(i-1)F_{BBRa}(i-1)\\
&+0.5M_{BBaa}(i-1)F_{BBAR}(i-1)+M_{BBaa}(i-1)F_{BBRR}(i-1)+0.25\eta M_{BBAa}(i-1)F_{Bbaa}(i-1)\\
&+0.125\eta M_{BBAa}(i-1)(1-\Phi-\eta)F_{BbAa}(i-1)+0.125(1-\Phi-\eta)M_{BBAa}(i-1)\eta F_{BbAa}(i-1)\\
&+0.125(1-\Phi-\eta)M_{BBAa}(i-1)F_{BbRa}(i-1)+0.125\eta M_{BBAa}(i-1)F_{BbRa}(i-1)\\
&+0.125(1-\Phi-\eta)M_{BBAa}(i-1)F_{BbAR}(i-1)+0.25(1-\Phi-\eta)M_{BBAa}(i-1)F_{BbRR}(i-1)\\
&+0.5\eta M_{BBAa}(i-1)F_{BBaa}(i-1)+0.25\eta M_{BBAa}(i-1)(1-\Phi-\eta)F_{BBAa}(i-1)\\
&+0.25(1-\Phi-\eta)M_{BBAa}(i-1)\eta F_{BBAa}(i-1)+0.25(1-\Phi-\eta)M_{BBAa}(i-1)F_{BBRa}(i-1)\\
&+0.25\eta M_{BBAa}(i-1)F_{BBRa}(i-1)+0.25(1-\Phi-\eta)M_{BBAa}(i-1)F_{BBAR}(i-1)\\
&+0.5(1-\Phi-\eta)M_{BBAa}(i-1)F_{BBRR}(i-1)+0.25M_{BBRa}(i-1)F_{Bbaa}(i-1)\\
&+0.125M_{BBRa}(i-1)(1-\Phi-\eta)F_{BbAa}(i-1)+0.125M_{BBRa}(i-1)\eta F_{BbAa}(i-1)
\end{aligned}
$$

$$
\begin{aligned}
&+ 0.25M_{BBRa}(i-1)F_{BbRa}(i-1) + 0.125M_{BBRa}(i-1)F_{BbAR}(i-1) + 0.25M_{BBRa}(i-1)F_{BbRR}(i-1)\\
&+ 0.5M_{BBRa}(i-1)F_{BBaa}(i-1) + 0.25M_{BBRa}(i-1)(1-\Phi-\eta)F_{BBAa}(i-1)\\
&+ 0.25M_{BBRa}(i-1)\eta F_{BBAa}(i-1) + 0.5M_{BBRa}(i-1)F_{BBRa}(i-1) + 0.25M_{BBRa}(i-1)F_{BBAR}(i-1)\\
&+ 0.5M_{BBRa}(i-1)F_{BBRR}(i-1) + 0.25M_{BBAR}(i-1)F_{Bbaa}(i-1)\\
&+ 0.125M_{BBAR}(i-1)(1-\Phi-\eta)F_{BbAa}(i-1) + 0.125M_{BBAR}(i-1)F_{BbRa}(i-1)\\
&+ 0.5M_{BBAR}(i-1)F_{BBaa}(i-1) + 0.25M_{BBAR}(i-1)(1-\Phi-\eta)F_{BBAa}(i-1)\\
&+ 0.25M_{BBAR}(i-1)F_{BBRa}(i-1) + 0.5M_{BBRR}(i-1)F_{Bbaa}(i-1)\\
&+ 0.25M_{BBRR}(i-1)(1-\Phi-\eta)F_{BbAa}(i-1) + 0.25M_{BBRR}(i-1)F_{BbRa}(i-1)\\
&+ M_{BBRR}(i-1)F_{BBaa}(i-1) + 0.5M_{BBRR}(i-1)(1-\Phi-\eta)F_{BBAa}(i-1)\\
&+ 0.5M_{BBRR}(i-1)F_{BBRa}(i-1)],
\end{aligned}
$$

$$
\begin{aligned}
M^e_{BBAA} = \left(\frac{\Omega_{BBAA}}{2}\right)&[0.0625(1-\Phi-\eta)M_{BbAa}(i-1)(1-\Phi-\eta) + 0.125\Phi M_{BbAa}(i-1)(1-\Phi-\eta)F_{BbAa}(i-1)\\
&+ 0.0625\eta M_{BbAa}(i-1)(1-\Phi-\eta)F_{BbAa}(i-1) + 0.125(1-\Phi-\eta)M_{BbAa}(i-1)\Phi F_{BbAa}(i-1)\\
&+ 0.0625(1-\Phi-\eta)M_{BbAa}(i-1)\eta F_{BbAa}(i-1) + 0.25\Phi M_{BbAa}(i-1)\Phi F_{BbAa}(i-1)\\
&+ 0.125\Phi M_{BbAa}(i-1)\eta F_{BbAa}(i-1) + 0.125\eta M_{BbAa}(i-1)\Phi F_{BbAa}(i-1)\\
&+ 0.0625\eta M_{BbAa}(i-1)\eta F_{BbAa}(i-1) + 0.125(1-\Phi-\eta)M_{BbAa}(i-1)F_{BbAA}(i-1)\\
&+ 0.25\Phi M_{BbAa}(i-1)F_{BbAA}(i-1) + 0.125\eta M_{BbAa}(i-1)F_{BbAA}(i-1)\\
&+ 0.0625(1-\Phi-\eta)M_{BbAa}(i-1)F_{BbAR}(i-1) + 0.125\Phi M_{BbAa}(i-1)F_{BbAR}(i-1)\\
&+ 0.0625\eta M_{BbAa}(i-1)F_{BbAR}(i-1) + 0.125(1-\Phi-\eta)M_{BbAa}(i-1)(1-\Phi-\eta)F_{BBAa}(i-1)\\
&+ 0.25\Phi M_{BbAa}(i-1)(1-\Phi-\eta)F_{BBAa}(i-1) + 0.125\eta M_{BbAa}(i-1)(1-\Phi-\eta)F_{BBAa}(i-1)\\
&+ 0.125(1-\Phi-\eta)M_{BbAa}(i-1)\Phi F_{BBAa}(i-1) + 0.0625(1-\Phi-\eta)M_{BbAa}(i-1)\eta F_{BBAa}(i-1)\\
&+ 0.5\Phi M_{BbAa}(i-1)\Phi F_{BBAa}(i-1) + 0.25\Phi M_{BbAa}(i-1)\eta F_{BBAa}(i-1)\\
&+ 0.25\eta M_{BbAa}(i-1)\Phi F_{BBAa}(i-1) + 0.125\eta M_{BbAa}(i-1)\eta F_{BBAa}(i-1)\\
&+ 0.25(1-\Phi-\eta)M_{BbAa}(i-1)F_{BBAA}(i-1) + 0.5\Phi M_{BbAa}(i-1)F_{BBAA}(i-1)\\
&+ 0.25\eta M_{BbAa}(i-1)F_{BBAA}(i-1) + 0.125(1-\Phi-\eta)M_{BbAa}(i-1)F_{BBAR}(i-1)\\
&+ 0.25\Phi M_{BbAa}(i-1)F_{BBAR}(i-1) + 0.125\eta M_{BbAa}(i-1)F_{BBAR}(i-1)\\
&+ 0.125M_{BbAA}(i-1)(1-\Phi-\eta)F_{BbAa}(i-1) + 0.25M_{BbAA}(i-1)\Phi F_{BbAa}(i-1)\\
&+ 0.125M_{BbAA}(i-1)\eta F_{BbAa}(i-1) + 0.25M_{BbAA}(i-1)F_{BbAA}(i-1) + 0.125M_{BbAA}(i-1)F_{BbAR}(i-1)\\
&+ 0.25M_{BbAA}(i-1)(1-\Phi-\eta)F_{BBAa}(i-1) + 0.5M_{BbAA}(i-1)\Phi F_{BBAa}(i-1)\\
&+ 0.25M_{BbAA}(i-1)\eta F_{BBAa}(i-1) + 0.5M_{BbAA}(i-1)F_{BBAA}(i-1) + 0.25M_{BbAA}(i-1)F_{BBAR}(i-1)\\
&+ 0.0625M_{BbAR}(i-1)(1-\Phi-\eta)F_{BbAa}(i-1) + 0.125M_{BbAR}(i-1)\Phi F_{BbAa}(i-1)\\
&+ 0.0625M_{BbAR}(i-1)\eta F_{BbAa}(i-1) + 0.125M_{BbAR}(i-1)F_{BbAA}(i-1) + 0.0625M_{BbAR}(i-1)F_{BbAR}(i-1)\\
&+ 0.125M_{BbAR}(i-1)(1-\Phi-\eta)F_{BBAa}(i-1) + 0.25M_{BbAR}(i-1)\Phi F_{BBAa}(i-1)\\
&+ 0.125M_{BbAR}(i-1)\eta F_{BBAa}(i-1) + 0.25M_{BbAR}(i-1)F_{BBAA}(i-1) + 0.125M_{BbAR}(i-1)F_{BBAR}(i-1)\\
&+ 0.125(1-\Phi-\eta)M_{BBAa}(i-1)(1-\Phi-\eta)F_{BbAa}(i-1) + 0.25\Phi M_{BBAa}(i-1)(1-\Phi-\eta)F_{BbAa}(i-1)\\
&+ 0.125\eta M_{BBAa}(i-1)(1-\Phi-\eta)F_{BbAa}(i-1)F_{BbAa}(i-1) + 0.25(1-\Phi-\eta)M_{BBAa}(i-1)\Phi F_{BbAa}(i-1)\\
&+ 0.125(1-\Phi-\eta)M_{BBAa}(i-1)\eta F_{BbAa}(i-1) + 0.5\Phi M_{BBAa}(i-1)\Phi F_{BbAa}(i-1)\\
&+ 0.25\Phi M_{BBAa}(i-1)\eta F_{BbAa}(i-1) + 0.25\eta M_{BBAa}(i-1)\Phi F_{BbAa}(i-1)\\
&+ 0.125\eta M_{BBAa}(i-1)\eta F_{BbAa}(i-1) + 0.25(1-\Phi-\eta)M_{BBAa}(i-1)F_{BbAA}(i-1)\\
&+ 0.5\Phi M_{BBAa}(i-1)F_{BbAA}(i-1) + 0.25\eta M_{BBAa}(i-1)F_{BbAA}(i-1)\\
&+ 0.125(1-\Phi-\eta)M_{BBAa}(i-1)F_{BbAR}(i-1) + 0.25\Phi M_{BBAa}(i-1)F_{BbAR}(i-1)\\
&+ 0.125\eta M_{BBAa}(i-1)F_{BbAR}(i-1) + 0.25(1-\Phi-\eta)M_{BBAa}(i-1)(1-\Phi-\eta)F_{BBAa}(i-1)\\
&+ 0.5\Phi M_{BBAa}(i-1)(1-\Phi-\eta)F_{BBAa}(i-1) + 0.25\eta M_{BBAa}(i-1)(1-\Phi-\eta)F_{BBAa}(i-1)\\
&+ 0.5(1-\Phi-\eta)M_{BBAa}(i-1)\Phi F_{BBAa}(i-1) + 0.25(1-\Phi-\eta)M_{BBAa}(i-1)\eta F_{BBAa}(i-1)\\
&+ \Phi M_{BBAa}(i-1)\Phi F_{BBAa}(i-1) + 0.5\Phi M_{BBAa}(i-1)\eta F_{BBAa}(i-1) + 0.5\eta M_{BBAa}(i-1)\Phi F_{BBAa}(i-1)\\
&+ 0.25\eta M_{BBAa}(i-1)\eta F_{BBAa}(i-1) + 0.5(1-\Phi-\eta)M_{BBAa}(i-1)F_{BBAA}(i-1)\\
&+ \Phi M_{BBAa}(i-1)F_{BBAA}(i-1) + 0.5\eta M_{BBAa}(i-1)F_{BBAA}(i-1)
\end{aligned}
$$

$$
\begin{aligned}
&+ 0.25(1-\Phi-\eta)M_{BBAa}(i-1)F_{BBAR}(i-1) + 0.5\Phi M_{BBAa}(i-1)F_{BBAR}(i-1) \\
&+ 0.25\eta M_{BBAa}(i-1)F_{BBAR}(i-1) + 0.25M_{BBAA}(i-1)(1-\Phi-\eta)F_{BbAa}(i-1) \\
&+ 0.5M_{BBAA}(i-1)\Phi F_{BbAa}(i-1) + 0.25M_{BBAA}(i-1)\eta F_{BbAa}(i-1) + 0.5M_{BBAA}(i-1)F_{BbAA}(i-1) \\
&+ 0.25M_{BBAA}(i-1)F_{BbAR}(i-1) + 0.5M_{BBAA}(i-1)(1-\Phi-\eta)F_{BBAa}(i-1) \\
&+ M_{BBAA}(i-1)\Phi F_{BBAa}(i-1) + 0.5M_{BBAA}(i-1)\eta F_{BBAa}(i-1) + M_{BBAA}(i-1)F_{BBAA}(i-1) \\
&+ 0.5M_{BBAA}(i-1)F_{BBAR}(i-1) + 0.125M_{BBAR}(i-1)(1-\Phi-\eta)F_{BbAa}(i-1) \\
&+ 0.25M_{BBAR}(i-1)\Phi F_{BbAa}(i-1) + 0.125M_{BBAR}(i-1)\eta F_{BbAa}(i-1) + 0.25M_{BBAR}(i-1)F_{BbAA}(i-1) \\
&+ 0.125M_{BBAR}(i-1)F_{BbAR}(i-1) + 0.25M_{BBAR}(i-1)(1-\Phi-\eta)F_{BBAa}(i-1) \\
&+ 0.5M_{BBAR}(i-1)\Phi F_{BBAa}(i-1) + 0.25M_{BBAR}(i-1)\eta F_{BBAa}(i-1) + 0.5M_{BBAR}(i-1)F_{BBAA}(i-1) \\
&+ 0.25M_{BBAR}(i-1)F_{BBAR}(i-1)],
\end{aligned}
$$

$$
\begin{aligned}
M^e_{BBAR} = \left(\frac{\Omega_{BBAR}}{2}\right)&[0.0625\eta M_{BbAa}(i-1)(1-\Phi-\eta)F_{BbAa}(i-1) + 0.0625(1-\Phi-\eta)M_{BbAa}(i-1)\eta F_{BbAa}(i-1) \\
&+ 0.125\Phi M_{BbAa}(i-1)\eta F_{BbAa}(i-1) + 0.125\eta M_{BbAa}(i-1)\Phi F_{BbAa}(i-1) \\
&+ 0.125\eta M_{BbAa}(i-1)\eta F_{BbAa}(i-1) + 0.0625(1-\Phi-\eta)M_{BbAa}(i-1)F_{BbRa}(i-1) \\
&+ 0.125\Phi M_{BbAa}(i-1)F_{BbRa}(i-1) + 0.0625\eta M_{BbAa}(i-1)F_{BbRa}(i-1) + 0.125\eta M_{BbAa}(i-1)F_{BbAA}(i-1) \\
&+ 0.0625(1-\Phi-\eta)M_{BbAa}(i-1)F_{BbAR}(i-1) + 0.125\Phi M_{BbAa}(i-1)F_{BbAR}(i-1) \\
&+ 0.125\eta M_{BbAa}(i-1)F_{BbAR}(i-1) + 0.125(1-\Phi-\eta)M_{BbAa}(i-1)F_{BbRR}(i-1) \\
&+ 0.25\Phi M_{BbAa}(i-1)F_{BbRR}(i-1) + 0.125\eta M_{BbAa}(i-1)F_{BbRR}(i-1) \\
&+ 0.125\eta M_{BbAa}(i-1)(1-\Phi-\eta)F_{BBAa}(i-1) + 0.0625(1-\Phi-\eta)M_{BbAa}(i-1)\eta F_{BBAa}(i-1) \\
&+ 0.25\Phi M_{BbAa}(i-1)\eta F_{BBAa}(i-1) + 0.25\eta M_{BbAa}(i-1)\Phi F_{BBAa}(i-1) + 0.25\eta M_{BbAa}(i-1)\eta F_{BBAa}(i-1) \\
&+ 0.125(1-\Phi-\eta)M_{BbAa}(i-1)F_{BBRa}(i-1) + 0.25\Phi M_{BbAa}(i-1)F_{BBRa}(i-1) \\
&+ 0.125\eta M_{BbAa}(i-1)F_{BBRa}(i-1) + 0.25\eta M_{BbAa}(i-1)F_{BBAA}(i-1) \\
&+ 0.125(1-\Phi-\eta)M_{BbAa}(i-1)F_{BBAR}(i-1) + 0.25\Phi M_{BbAa}(i-1)F_{BBAR}(i-1) \\
&+ 0.25\eta M_{BbAa}(i-1)F_{BBAR}(i-1) + 0.25(1-\Phi-\eta)M_{BbAa}(i-1)F_{BBRR}(i-1) \\
&+ 0.5\Phi M_{BbAa}(i-1)F_{BBRR}(i-1) + 0.25\eta M_{BbAa}(i-1)F_{BBRR}(i-1) \\
&+ 0.0625M_{BbRa}(i-1)(1-\Phi-\eta)F_{BbAa}(i-1) + 0.125M_{BbRa}(i-1)\Phi F_{BbAa}(i-1) \\
&+ 0.0625M_{BbRa}(i-1)\eta F_{BbAa}(i-1) + 0.125M_{BbRa}(i-1)F_{BbAA}(i-1) + 0.0625M_{BbRa}(i-1)F_{BbAR}(i-1) \\
&+ 0.125M_{BbRa}(i-1)(1-\Phi-\eta)F_{BBAa}(i-1) + 0.25M_{BbRa}(i-1)\Phi F_{BBAa}(i-1) \\
&+ 0.125M_{BbRa}(i-1)\eta F_{BBAa}(i-1) + 0.25M_{BbRa}(i-1)F_{BBAA}(i-1) + 0.125M_{BbRa}(i-1)F_{BBAR}(i-1) \\
&+ 0.125M_{BbAA}(i-1)\eta F_{BbAa}(i-1) + 0.125M_{BbAA}(i-1)F_{BbRa}(i-1) + 0.125M_{BbAA}(i-1)F_{BbAR}(i-1) \\
&+ 0.25M_{BbAA}(i-1)F_{BbRR}(i-1) + 0.25M_{BbAA}(i-1)\eta F_{BBAa}(i-1) + 0.25M_{BbAA}(i-1)F_{BBRa}(i-1) \\
&+ 0.25M_{BbAA}(i-1)F_{BBAR}(i-1) + 0.5M_{BbAA}(i-1)F_{BBRR}(i-1) \\
&+ 0.0625M_{BbAR}(i-1)(1-\Phi-\eta)F_{BbAa}(i-1) + 0.125M_{BbAR}(i-1)\Phi F_{BbAa}(i-1) \\
&+ 0.125M_{BbAR}(i-1)\eta F_{BbAa}(i-1) + 0.0625M_{BbAR}(i-1)F_{BbRa}(i-1) + 0.125M_{BbAR}(i-1)F_{BbAA}(i-1) \\
&+ 0.125M_{BbAR}(i-1)F_{BbAR}(i-1) + 0.125M_{BbAR}(i-1)F_{BbRR}(i-1) \\
&+ 0.125M_{BbAR}(i-1)(1-\Phi-\eta)F_{BBAa}(i-1) + 0.25M_{BbAR}(i-1)\Phi F_{BBAa}(i-1) \\
&+ 0.25M_{BbAR}(i-1)\eta F_{BBAa}(i-1) + 0.125M_{BbAR}(i-1)F_{BBRa}(i-1) + 0.25M_{BbAR}(i-1)F_{BBAA}(i-1) \\
&+ 0.25M_{BbAR}(i-1)F_{BBAR}(i-1) + 0.25M_{BbAR}(i-1)F_{BBRR}(i-1) \\
&+ 0.125M_{BbRR}(i-1)(1-\Phi-\eta)F_{BbAa}(i-1) + 0.25M_{BbRR}(i-1)\Phi F_{BbAa}(i-1) \\
&+ 0.125M_{BbRR}(i-1)\eta F_{BbAa}(i-1) + 0.25M_{BbRR}(i-1)F_{BbAA}(i-1) + 0.125M_{BbRR}(i-1)F_{BbAR}(i-1) \\
&+ 0.25M_{BbRR}(i-1)(1-\Phi-\eta)F_{BBAa}(i-1) + 0.5M_{BbRR}(i-1)\Phi F_{BBAa}(i-1) \\
&+ 0.25M_{BbRR}(i-1)\eta F_{BBAa}(i-1) + 0.5M_{BbRR}(i-1)F_{BBAA}(i-1) + 0.25M_{BbRR}(i-1)F_{BBAR}(i-1) \\
&+ 0.125\eta M_{BBAa}(i-1)(1-\Phi-\eta)F_{BbAa}(i-1) + 0.125(1-\Phi-\eta)M_{BBAa}(i-1)\eta F_{BbAa}(i-1) \\
&+ 0.25\Phi M_{BBAa}(i-1)\eta F_{BbAa}(i-1) + 0.25\eta M_{BBAa}(i-1)\Phi F_{BbAa}(i-1) + 0.25\eta M_{BBAa}(i-1)\eta F_{BbAa}(i-1) \\
&+ 0.125(1-\Phi-\eta)M_{BBAa}(i-1)F_{BbRa}(i-1) + 0.25\Phi M_{BBAa}(i-1)F_{BbRa}(i-1) \\
&+ 0.125\eta M_{BBAa}(i-1)F_{BbRa}(i-1) + 0.25\eta M_{BBAa}(i-1)F_{BbAA}(i-1) \\
&+ 0.125(1-\Phi-\eta)M_{BBAa}(i-1)F_{BbAR}(i-1) + 0.25\Phi M_{BBAa}(i-1)F_{BbAR}(i-1)
\end{aligned}
$$

$$
\begin{aligned}
&+ 0.25\eta M_{BBAa}(i-1)F_{BbAR}(i-1) + 0.25(1-\Phi-\eta)M_{BBAa}(i-1)F_{BbRR}(i-1) \\
&+ 0.5\Phi M_{BBAa}(i-1)F_{BbRR}(i-1) + 0.25\eta M_{BBAa}(i-1)F_{BbRR}(i-1) \\
&+ 0.25\eta M_{BBAa}(i-1)(1-\Phi-\eta)F_{BBAa}(i-1) + 0.25(1-\Phi-\eta)M_{BBAa}(i-1)\eta F_{BBAa}(i-1) \\
&+ 0.5\Phi M_{BBAa}(i-1)\eta F_{BBAa}(i-1) + 0.5\eta M_{BBAa}(i-1)\Phi F_{BBAa}(i-1) + 0.5\eta M_{BBAa}(i-1)\eta F_{BBAa}(i-1) \\
&+ 0.25(1-\Phi-\eta)M_{BBAa}(i-1)F_{BBRa}(i-1) + 0.5\Phi M_{BBAa}(i-1)F_{BBRa}(i-1) \\
&+ 0.25\eta M_{BBAa}(i-1)F_{BBRa}(i-1) + 0.5\eta M_{BBAa}(i-1)F_{BBAA}(i-1) \\
&+ 0.25(1-\Phi-\eta)M_{BBAa}(i-1)F_{BBAR}(i-1) + 0.5\Phi M_{BBAa}(i-1)F_{BBAR}(i-1) \\
&+ 0.5\eta M_{BBAa}(i-1)F_{BBAR}(i-1) + 0.5(1-\Phi-\eta)M_{BBAa}(i-1)F_{BBRR}(i-1) \\
&+ \Phi M_{BBAa}(i-1)F_{BBRR}(i-1) + 0.5\eta M_{BBAa}(i-1)F_{BBRR}(i-1) \\
&+ 0.125M_{BBRa}(i-1)(1-\Phi-\eta)F_{BbAa}(i-1) + 0.25M_{BBRa}(i-1)\Phi F_{BbAa}(i-1) \\
&+ 0.125M_{BBRa}(i-1)\eta F_{BbAa}(i-1) + 0.25M_{BBRa}(i-1)F_{BbAA}(i-1) + 0.125M_{BBRa}(i-1)F_{BbAR}(i-1) \\
&+ 0.25M_{BBRa}(i-1)(1-\Phi-\eta)F_{BBAa}(i-1) + 0.5M_{BBRa}(i-1)\Phi F_{BBAa}(i-1) \\
&+ 0.25M_{BBRa}(i-1)\eta F_{BBAa}(i-1) + 0.5M_{BBRa}(i-1)F_{BBAA}(i-1) + 0.25M_{BBRa}(i-1)F_{BBAR}(i-1) \\
&+ 0.25M_{BBAA}(i-1)\eta F_{BbAa}(i-1) + 0.25M_{BBAA}(i-1)F_{BbRa}(i-1) + 0.25M_{BBAA}(i-1)F_{BbAR}(i-1) \\
&+ 0.5M_{BBAA}(i-1)F_{BbRR}(i-1) + 0.5M_{BBAA}(i-1)\eta F_{BBAa}(i-1) + 0.5M_{BBAA}(i-1)F_{BBRa}(i-1) \\
&+ 0.5M_{BBAA}(i-1)F_{BBAR}(i-1) + M_{BBAA}(i-1)F_{BBRR}(i-1) \\
&+ 0.125M_{BBAR}(i-1)(1-\Phi-\eta)F_{BbAa}(i-1) + 0.25M_{BBAR}(i-1)\Phi F_{BbAa}(i-1) \\
&+ 0.25M_{BBAR}(i-1)\eta F_{BbAa}(i-1) + 0.125M_{BBAR}(i-1)F_{BbRa}(i-1) + 0.25M_{BBAR}(i-1)F_{BbAA}(i-1) \\
&+ 0.25M_{BBAR}(i-1)F_{BbAR}(i-1) + 0.25M_{BBAR}(i-1)F_{BbRR}(i-1) \\
&+ 0.25M_{BBAR}(i-1)(1-\Phi-\eta)F_{BBAa}(i-1) + 0.5M_{BBAR}(i-1)\Phi F_{BBAa}(i-1) \\
&+ 0.5M_{BBAR}(i-1)\eta F_{BBAa}(i-1) + 0.25M_{BBAR}(i-1)F_{BBRa}(i-1) + 0.5M_{BBAR}(i-1)F_{BBAA}(i-1) \\
&+ 0.5M_{BBAR}(i-1)F_{BBAR}(i-1) + 0.5M_{BBAR}(i-1)F_{BBRR}(i-1) \\
&+ 0.25M_{BBRR}(i-1)(1-\Phi-\eta)F_{BbAa}(i-1) + 0.5M_{BBRR}(i-1)\Phi F_{BbAa}(i-1) \\
&+ 0.25M_{BBRR}(i-1)\eta F_{BbAa}(i-1) + 0.5M_{BBRR}(i-1)F_{BbAA}(i-1) + 0.25M_{BBRR}(i-1)F_{BbAR}(i-1) \\
&+ 0.5M_{BBRR}(i-1)(1-\Phi-\eta)F_{BBAa}(i-1) + M_{BBRR}(i-1)\Phi F_{BBAa}(i-1) \\
&+ 0.5M_{BBRR}(i-1)\eta F_{BBAa}(i-1) + M_{BBRR}(i-1)F_{BBAA}(i-1) + 0.5M_{BBRR}(i-1)F_{BBAR}(i-1)],
\end{aligned}
$$

$$
\begin{aligned}
M^e_{BBRR} = \left(\frac{\Omega_{BBRR}}{2}\right)&[0.0625\eta M_{BbAa}(i-1)\eta F_{BbAa}(i-1) + 0.0625\eta M_{BbAa}(i-1)F_{BbRa}(i-1) \\
&+ 0.0625\eta M_{BbAa}(i-1)F_{BbAR}(i-1) + 0.125\eta M_{BbAa}(i-1)F_{BbRR}(i-1) \\
&+ 0.125\eta M_{BbAa}(i-1)\eta F_{BBAa}(i-1) + 0.125\eta M_{BbAa}(i-1)F_{BBRa}(i-1) \\
&+ 0.125\eta M_{BbAa}(i-1)F_{BBAR}(i-1) + 0.25\eta M_{BbAa}(i-1)F_{BBRR}(i-1) + 0.0625M_{BbRa}(i-1)\eta F_{BbAa}(i-1) \\
&+ 0.0625M_{BbRa}(i-1)F_{BbRa}(i-1) + 0.0625M_{BbRa}(i-1)F_{BbAR}(i-1) + 0.125M_{BbRa}(i-1)F_{BbRR}(i-1) \\
&+ 0.125M_{BbRa}(i-1)\eta F_{BBAa}(i-1) + 0.125M_{BbRa}(i-1)F_{BBRa}(i-1) + 0.125M_{BbRa}(i-1)F_{BBAR}(i-1) \\
&+ 0.25M_{BbRa}(i-1)F_{BBRR}(i-1) + 0.0625M_{BbAR}(i-1)\eta F_{BbAa}(i-1) + 0.0625M_{BbAR}(i-1)F_{BbRa}(i-1) \\
&+ 0.0625M_{BbAR}(i-1)F_{BbAR}(i-1) + 0.125M_{BbAR}(i-1)F_{BbRR}(i-1) + 0.125M_{BbAR}(i-1)\eta F_{BBAa}(i-1) \\
&+ 0.125M_{BbAR}(i-1)F_{BBRa}(i-1) + 0.125M_{BbAR}(i-1)F_{BBAR}(i-1) + 0.25M_{BbAR}(i-1)F_{BBRR}(i-1) \\
&+ 0.125M_{BbRR}(i-1)\eta F_{BbAa}(i-1) + 0.125M_{BbRR}(i-1)F_{BbRa}(i-1) + 0.125M_{BbRR}(i-1)F_{BbAR}(i-1) \\
&+ 0.25M_{BbRR}(i-1)F_{BbRR}(i-1) + 0.25M_{BbRR}(i-1)\eta F_{BBAa}(i-1) + 0.25M_{BbRR}(i-1)F_{BBRa}(i-1) \\
&+ 0.25M_{BbRR}(i-1)F_{BBAR}(i-1) + 0.5M_{BbRR}(i-1)F_{BBRR}(i-1) + 0.125\eta M_{BBAa}(i-1)\eta F_{BbAa}(i-1) \\
&+ 0.125\eta M_{BBAa}(i-1)F_{BbRa}(i-1) + 0.125\eta M_{BBAa}(i-1)F_{BbAR}(i-1) + 0.25\eta M_{BBAa}(i-1)F_{BbRR}(i-1) \\
&+ 0.25\eta M_{BBAa}(i-1)\eta F_{BBAa}(i-1) + 0.25\eta M_{BBAa}(i-1)F_{BBRa}(i-1) + 0.25\eta M_{BBAa}(i-1)F_{BBAR}(i-1) \\
&+ 0.5\eta M_{BBAa}(i-1)F_{BBRR}(i-1) + 0.125M_{BBRa}(i-1)\eta F_{BbAa}(i-1) + 0.125M_{BBRa}(i-1)F_{BbRa}(i-1) \\
&+ 0.125M_{BBRa}(i-1)F_{BbAR}(i-1) + 0.25M_{BBRa}(i-1)F_{BbRR}(i-1) + 0.25M_{BBRa}(i-1)\eta F_{BBAa}(i-1) \\
&+ 0.25M_{BBRa}(i-1)F_{BBRa}(i-1) + 0.25M_{BBRa}(i-1)F_{BBAR}(i-1) + 0.5M_{BBRa}(i-1)F_{BBRR}(i-1) \\
&+ 0.125M_{BBAR}(i-1)\eta F_{BbAa}(i-1) + 0.125M_{BBAR}(i-1)F_{BbRa}(i-1) + 0.125M_{BBAR}(i-1)F_{BbAR}(i-1) \\
&+ 0.25M_{BBAR}(i-1)F_{BbRR}(i-1) + 0.25M_{BBAR}(i-1)\eta F_{BBAa}(i-1) + 0.25M_{BBAR}(i-1)F_{BBRa}(i-1) \\
&+ 0.25M_{BBAR}(i-1)F_{BBAR}(i-1) + 0.5M_{BBAR}(i-1)F_{BBRR}(i-1) + 0.25M_{BBRR}(i-1)\eta F_{BbAa}(i-1)
\end{aligned}
$$

$$+ 0.25M_{BBRR}(i-1)F_{BbRa}(i-1) + 0.25M_{BBRR}(i-1)F_{BbAR}(i-1) + 0.5M_{BBRR}(i-1)F_{BbRR}(i-1)$$
$$+ 0.5M_{BBRR}(i-1)\eta F_{BBAa}(i-1) + 0.5M_{BBRR}(i-1)F_{BBRa}(i-1) + 0.5M_{BBRR}(i-1)F_{BBAR}(i-1)$$
$$+ M_{BBRR}(i-1)F_{BBRR}(i-1)],$$

and a matching set of equations for females of each genotype.

The second stage is to normalise these proportional frequencies to fill the range from zero to one. We begin by calculating the overall fitness of the entire population by summing the proportional genotype frequencies resulting from the above equations to give:

$$\bar{\Omega} = M^e_{bbaa} + M^e_{bbAa} + M^e_{bbRa} + M^e_{bbAA} + M^e_{bbAR} + M^e_{bbRR} + M^e_{Bbaa} + M^e_{BbAa} + M^e_{BbRa}$$
$$+ M^e_{BbAA} + M^e_{BbAR} + M^e_{BbRR} + M^e_{BBaa} + M^e_{BBAa} + M^e_{BBRa} + M^e_{BBAA} + M^e_{BBAR} + M^e_{BBRR}$$
$$+ F^e_{bbaa} + F^e_{bbAa} + F^e_{bbRa} + F^e_{bbAA} + F^e_{bbAR} + F^e_{bbRR} + F^e_{Bbaa} + F^e_{BbAa} + F^e_{BbRa}$$
$$+ F^e_{BbAA} + F^e_{BbAR} + F^e_{BbRR} + F^e_{BBaa} + F^e_{BBAa} + F^e_{BBRa} + F^e_{BBAA} + F^e_{BBAR} + F^e_{BBRR}.$$

This is used as a normalising factor to give the final genotype frequencies for a particular generation as:

$$M_{bbaa}(i) = M^e_{bbaa}/\bar{\Omega}, \quad M_{bbAa}(i) = M^e_{bbAa}, \quad M_{bbRa}(i) = M^e_{bbRa}/\bar{\Omega}, \quad M_{bbAA}(i) = M^e_{bbAA}/\bar{\Omega},$$
$$M_{bbAR}(i) = M^e_{bbAR}/\bar{\Omega}, \quad M_{bbRR}(i) = M^e_{bbRR}/\bar{\Omega}, \quad M_{Bbaa}(i) = M^e_{Bbaa}/\bar{\Omega}, \quad M_{BbAa}(i) = M^e_{BbAa}/\bar{\Omega},$$
$$M_{BbRa}(i) = M^e_{BbRa}/\bar{\Omega}, \quad M_{BbAA}(i) = M^e_{BbAA}/\bar{\Omega}, \quad M_{BbAR}(i) = M^e_{BbAR}/\bar{\Omega}, \quad M_{BbRR}(i) = M^e_{BbRR}/\bar{\Omega},$$
$$M_{BBaa}(i) = M^e_{BBaa}/\bar{\Omega}, \quad M_{BBAa}(i) = M^e_{BBAa}/\bar{\Omega}, \quad M_{BBRa}(i) = M^e_{BBRa}/\bar{\Omega}, \quad M_{BBAA}(i) = M^e_{BBAA}/\bar{\Omega},$$
$$M_{BBAR}(i) = M^e_{BBAR}/\bar{\Omega}, \quad M_{BBRR}(i) = M^e_{BBRR}/\bar{\Omega}, \quad F_{bbaa}(i) = F^e_{bbaa}/\bar{\Omega}, \quad F_{bbAa}(i) = F^e_{bbAa}/\bar{\Omega},$$
$$F_{bbRa}(i) = F^e_{bbRa}/\bar{\Omega}, \quad F_{bbAA}(i) = F^e_{bbAA}/\bar{\Omega}, \quad F_{bbAR}(i) = F^e_{bbAR}/\bar{\Omega}, \quad F_{bbRR}(i) = F^e_{bbRR}/\bar{\Omega},$$
$$F_{Bbaa}(i) = F^e_{Bbaa}/\bar{\Omega}, \quad F_{BbAa}(i) = F^e_{BbAa}/\bar{\Omega}, \quad F_{BbRa}(i) = F^e_{BbRa}/\bar{\Omega}, \quad F_{BbAA}(i) = F^e_{BbAA}/\bar{\Omega},$$
$$F_{BbAR}(i) = F^e_{BbAR}/\bar{\Omega}, \quad F_{BbRR}(i) = F^e_{BbRR}/\bar{\Omega}, \quad F_{BBaa}(i) = F^e_{BBaa}/\bar{\Omega}, \quad F_{BBAa}(i) = F^e_{BBAa}/\bar{\Omega},$$
$$F_{BBRa}(i) = F^e_{BBRa}/\bar{\Omega}, \quad F_{BBAA}(i) = F^e_{BBAA}/\bar{\Omega}, \quad F_{BBAR}(i) = F^e_{BBAR}/\bar{\Omega}, \quad F_{BBRR}(i) = F^e_{BBRR}/\bar{\Omega}.$$

We then insert these values into the proportional genotype frequency equations to calculate the values for the next generation and so on until the desired end point is reached.
