# Supplementary material for: Split drive killer-rescue provides a novel threshold-dependent gene drive
Source: Sci Rep. 2020 Nov 25;10:20520. doi: 10.1038/s41598-020-77544-7 (PMC7689494; doi:10.1038/s41598-020-77544-7)
Supplement: Supplementary file 3 — Supplementary material 3 [file 41598_2020_77544_MOESM3_ESM.pdf]

Split drive killer-rescue provides a novel threshold-dependent gene drive

Matthew P. Edgington[1*], Tim Harvey-Samuel[1] and Luke Alphey[1]

[1]The Pirbright Institute, Ash Road, Woking, Surrey GU24 0NF, UK
[*]Corresponding author: matt.edgington@pirbright.ac.uk

Supplementary Model 3

We begin here by defining a baseline parameter set for use throughout this study. This is as follows:

- $L = 1$ (full lethal penetrance),
- $\varepsilon_A = 0.85$ (relative to 1 in wild-type individuals - 15% fitness cost),
- $\varepsilon_B = 0.85$ (relative to 1 in wild-type individuals - 15% fitness cost),
- $\Phi = 0.9$ (90% homing rate),
- $\delta = 0.9$ (90% conversion of A homozygotes from female to male),
- $\delta_D = \delta$ (conversion rate of A heterozygotes - delta if conversion is dominant or zero if recessive),
- $\mu = 0.02$ (rate of migration from the target into the non-target population).

The relative fitness parameters defined here are combined multiplicatively to obtain a single overall relative fitness value for individuals of each genotype (assumed equal for both sexes). These are of the form:

$$\Omega_{bbaa} = 1, \quad \Omega_{bbAa} = (1-L)\varepsilon_A, \quad \Omega_{bbAA} = (1-L)\varepsilon_A^2, \quad \Omega_{Bbaa} = \varepsilon_B, \quad \Omega_{BbAa} = \varepsilon_B\varepsilon_A,$$
$$\Omega_{BbAA} = \varepsilon_B\varepsilon_A^2, \quad \Omega_{BBaa} = \varepsilon_B^2, \quad \Omega_{BBAa} = \varepsilon_B^2\varepsilon_A, \quad \Omega_{BBAA} = \varepsilon_B^2\varepsilon_A^2.$$

We then define a set of initial conditions representing the genotype proportions in the population when transgenic individuals are introduced into the wild population. For a 1:1 (introduced:wild) release of individuals homozygous for both transgenic constructs, as is most widely considered in this study, these are of the form:

$$M_{bbaa}(1) = 0.25 = F_{bbaa}(1), \quad M_{bbAa}(1) = 0.00 = F_{bbAa}(1), \quad M_{bbAA}(1) = 0.00 = F_{bbAA}(1),$$
$$M_{Bbaa}(1) = 0.00 = F_{Bbaa}(1), \quad M_{BbAa}(1) = 0.00 = F_{BbAa}(1), \quad M_{BbAA}(1) = 0.00 = F_{BbAA}(1),$$
$$M_{BBaa}(1) = 0.00 = F_{BBaa}(1), \quad M_{BBAa}(1) = 0.00 = F_{BBAa}(1), \quad M_{BBAA}(1) = 0.25 = F_{BBAA}(1).$$

With the above definitions we then iteratively calculate the genotype frequencies in each subsequent generation. This process is conducted in a two step manner. The first stage is to calculate the proportional frequencies for each genotype (and for each sex), which is achieved using:

$$\begin{aligned}
M^e_{bbaa} = \left(\frac{\Omega_{bbaa}}{2}\right) & [M_{bbaa}(i-1)F_{bbaa}(i-1) + 0.5M_{bbaa}(i-1)F_{bbAa}(i-1) + 0.5M_{bbaa}(i-1)F_{Bbaa}(i-1) \\
& + 0.25M_{bbaa}(i-1)(1-\Phi)F_{BbAa}(i-1) + 0.5M_{bbAa}(i-1)F_{bbaa}(i-1) + 0.25M_{bbAa}(i-1)F_{bbAa}(i-1) \\
& + 0.25M_{bbAa}(i-1)F_{Bbaa}(i-1) + 0.125M_{bbAa}(i-1)(1-\Phi)F_{BbAa}(i-1) + 0.5M_{Bbaa}(i-1)F_{bbaa}(i-1) \\
& + 0.25M_{Bbaa}(i-1)F_{bbAa}(i-1) + 0.25M_{Bbaa}(i-1)F_{Bbaa}(i-1) + 0.125M_{Bbaa}(i-1)(1-\Phi)F_{BbAa}(i-1) \\
& + 0.25(1-\Phi)M_{BbAa}(i-1)F_{bbaa}(i-1) + 0.125(1-\Phi)M_{BbAa}(i-1)F_{bbAa}(i-1) \\
& + 0.125(1-\Phi)M_{BbAa}(i-1)F_{Bbaa}(i-1) + 0.0625(1-\Phi)M_{BbAa}(i-1)(1-\Phi)F_{BbAa}(i-1)],
\end{aligned}$$

$$\begin{aligned}
M^e_{bbAa} = \left((1+\delta_D)\frac{\Omega_{bbAa}}{2}\right) & [0.5M_{bbaa}(i-1)F_{bbAa}(i-1) + M_{bbaa}(i-1)F_{bbAA}(i-1) \\
& + 0.25M_{bbaa}(i-1)(1-\Phi)F_{BbAa}(i-1) + 0.5M_{bbaa}(i-1)\Phi F_{BbAa}(i-1) + 0.5M_{bbaa}(i-1)F_{BbAA}(i-1) \\
& + 0.5M_{bbAa}(i-1)F_{bbaa}(i-1) + 0.5M_{bbAa}(i-1)F_{bbAa}(i-1) + 0.5M_{bbAa}(i-1)F_{bbAA}(i-1)
\end{aligned}$$

$$+ 0.25M_{bbAa}(i-1)F_{Bbaa}(i-1) + 0.25M_{bbAa}(i-1)(1-\Phi)F_{BbAa}(i-1) + 0.25M_{bbAa}(i-1)\Phi F_{BbAa}(i-1)$$
$$+ 0.25M_{bbAa}(i-1)F_{BbAA}(i-1) + M_{bbAA}(i-1)F_{bbaa}(i-1) + 0.5M_{bbAA}(i-1)F_{bbAa}(i-1)$$
$$+ 0.5M_{bbAA}(i-1)F_{Bbaa}(i-1) + 0.25M_{bbAA}(i-1)(1-\Phi)F_{BbAa}(i-1) + 0.25M_{Bbaa}(i-1)F_{bbAa}(i-1)$$
$$+ 0.5M_{Bbaa}(i-1)F_{bbAA}(i-1) + 0.125M_{Bbaa}(i-1)(1-\Phi)F_{BbAa}(i-1) + 0.25M_{Bbaa}(i-1)\Phi F_{BbAa}(i-1)$$
$$+ 0.25M_{Bbaa}(i-1)F_{BbAA}(i-1) + 0.25(1-\Phi)M_{BbAa}(i-1)F_{bbaa}(i-1) + 0.5\Phi M_{BbAa}(i-1)F_{bbaa}(i-1)$$
$$+ 0.25(1-\Phi)M_{BbAa}(i-1)F_{bbAa}(i-1) + 0.25\Phi M_{BbAa}(i-1)F_{bbAa}(i-1)$$
$$+ 0.25(1-\Phi)M_{BbAa}(i-1)F_{bbAA}(i-1) + 0.125(1-\Phi)M_{BbAa}(i-1)F_{Bbaa}(i-1)$$
$$+ 0.25\Phi M_{BbAa}(i-1)F_{Bbaa}(i-1) + 0.125(1-\Phi)M_{BbAa}(i-1)(1-\Phi)F_{BbAa}(i-1)$$
$$+ 0.125\Phi M_{BbAa}(i-1)(1-\Phi)F_{BbAa}(i-1) + 0.125(1-\Phi)M_{BbAa}(i-1)\Phi F_{BbAa}(i-1)$$
$$+ 0.125(1-\Phi)M_{BbAa}(i-1)F_{BbAA}(i-1) + 0.125(1-\Phi)M_{BbAa}(i-1)\Phi F_{BBAa}(i-1)$$
$$+ 0.5M_{BbAA}(i-1)F_{bbaa}(i-1) + 0.25M_{BbAA}(i-1)F_{bbAa}(i-1) + 0.25M_{BbAA}(i-1)F_{Bbaa}(i-1)$$
$$+ 0.125M_{BbAA}(i-1)(1-\Phi)F_{BbAa}(i-1)],$$

$$M^e_{bbAA} = \left((1+\delta)\frac{\Omega_{bbAA}}{2}\right)[0.25M_{bbAa}(i-1)F_{bbAa}(i-1) + 0.5M_{bbAa}(i-1)F_{bbAA}(i-1)$$
$$+ 0.125M_{bbAa}(i-1)(1-\Phi)F_{BbAa}(i-1) + 0.25M_{bbAa}(i-1)\Phi F_{BbAa}(i-1) + 0.25M_{bbAa}(i-1)F_{BbAA}(i-1)$$
$$+ 0.5M_{bbAA}(i-1)F_{bbAa}(i-1) + M_{bbAA}(i-1)F_{bbAA}(i-1) + 0.25M_{bbAA}(i-1)(1-\Phi)F_{BbAa}(i-1)$$
$$+ 0.5M_{bbAA}(i-1)\Phi F_{BbAa}(i-1) + 0.5M_{bbAA}(i-1)F_{BbAA}(i-1) + 0.125(1-\Phi)M_{BbAa}(i-1)F_{bbAa}(i-1)$$
$$+ 0.25\Phi M_{BbAa}(i-1)F_{bbAa}(i-1) + 0.25(1-\Phi)M_{BbAa}(i-1)F_{bbAA}(i-1) + 0.5\Phi M_{BbAa}(i-1)F_{bbAA}(i-1)$$
$$+ 0.0625(1-\Phi)M_{BbAa}(i-1)(1-\Phi)F_{BbAa}(i-1) + 0.125\Phi M_{BbAa}(i-1)(1-\Phi)F_{BbAa}(i-1)$$
$$+ 0.125(1-\Phi)M_{BbAa}(i-1)\Phi F_{BbAa}(i-1) + 0.25\Phi M_{BbAa}(i-1)\Phi F_{BbAa}(i-1)$$
$$+ 0.125(1-\Phi)M_{BbAa}(i-1)F_{BbAA}(i-1) + 0.25\Phi M_{BbAa}(i-1)F_{BbAA}(i-1)$$
$$+ 0.125(1-\Phi)M_{BbAa}(i-1)\Phi F_{BBAa}(i-1) + 0.25M_{BbAA}(i-1)F_{bbAa}(i-1) + 0.5M_{BbAA}(i-1)F_{bbAA}(i-1)$$
$$+ 0.125M_{BbAA}(i-1)(1-\Phi)F_{BbAa}(i-1) + 0.25M_{BbAA}(i-1)\Phi F_{BbAa}(i-1)$$
$$+ 0.25M_{BbAA}(i-1)F_{BbAA}(i-1)],$$

$$M^e_{Bbaa} = \left(\frac{\Omega_{Bbaa}}{2}\right)[0.5M_{bbaa}(i-1)F_{Bbaa}(i-1) + 0.25M_{bbaa}(i-1)(1-\Phi)F_{BbAa}(i-1) + M_{bbaa}(i-1)F_{BBaa}(i-1)$$
$$+ 0.5M_{bbaa}(i-1)(1-\Phi)F_{BBAa}(i-1) + 0.25M_{bbAa}(i-1)F_{Bbaa}(i-1)$$
$$+ 0.125M_{bbAa}(i-1)(1-\Phi)F_{BbAa}(i-1) + 0.5M_{bbAa}(i-1)F_{BBaa}(i-1)$$
$$+ 0.25M_{bbAa}(i-1)(1-\Phi)F_{BBAa}(i-1) + 0.5M_{Bbaa}(i-1)F_{bbaa}(i-1) + 0.25M_{Bbaa}(i-1)F_{bbAa}(i-1)$$
$$+ 0.5M_{Bbaa}(i-1)F_{Bbaa}(i-1) + 0.25M_{Bbaa}(i-1)(1-\Phi)F_{BbAa}(i-1) + 0.5M_{Bbaa}(i-1)F_{BBaa}(i-1)$$
$$+ 0.25M_{Bbaa}(i-1)(1-\Phi)F_{BBAa}(i-1) + 0.25(1-\Phi)M_{BbAa}(i-1)F_{bbaa}(i-1)$$
$$+ 0.125(1-\Phi)M_{BbAa}(i-1)F_{bbAa}(i-1) + 0.25(1-\Phi)M_{BbAa}(i-1)F_{Bbaa}(i-1)$$
$$+ 0.125(1-\Phi)M_{BbAa}(i-1)(1-\Phi)F_{BbAa}(i-1) + 0.25(1-\Phi)M_{BbAa}(i-1)F_{BBaa}(i-1)$$
$$+ 0.125(1-\Phi)M_{BbAa}(i-1)(1-\Phi)F_{BBAa}(i-1) + M_{BBaa}(i-1)F_{bbaa}(i-1) + 0.5M_{BBaa}(i-1)F_{bbAa}(i-1)$$
$$+ 0.5M_{BBaa}(i-1)F_{Bbaa}(i-1) + 0.25M_{BBaa}(i-1)(1-\Phi)F_{BbAa}(i-1)$$
$$+ 0.5(1-\Phi)M_{BBAa}(i-1)F_{bbaa}(i-1) + 0.25(1-\Phi)M_{BBAa}(i-1)F_{bbAa}(i-1)$$
$$+ 0.25(1-\Phi)M_{BBAa}(i-1)F_{Bbaa}(i-1) + 0.125(1-\Phi)M_{BBAa}(i-1)(1-\Phi)F_{BbAa}(i-1)],$$

$$M^e_{BbAa} = \left((1+\delta_D)\frac{\Omega_{BbAa}}{2}\right)[0.25M_{bbaa}(i-1)(1-\Phi)F_{BbAa}(i-1) + 0.5M_{bbaa}(i-1)\Phi F_{BbAa}(i-1)$$
$$+ 0.5M_{bbaa}(i-1)F_{BbAA}(i-1) + 0.5M_{bbaa}(i-1)(1-\Phi)F_{BBAa}(i-1) + M_{bbaa}(i-1)\Phi F_{BBAa}(i-1)$$
$$+ M_{bbaa}(i-1)F_{BBAA}(i-1) + 0.25M_{bbAa}(i-1)F_{Bbaa}(i-1) + 0.25M_{bbAa}(i-1)(1-\Phi)F_{BbAa}(i-1)$$
$$+ 0.25M_{bbAa}(i-1)\Phi F_{BbAa}(i-1) + 0.25M_{bbAa}(i-1)F_{BbAA}(i-1) + 0.5M_{bbAa}(i-1)F_{BBaa}(i-1)$$
$$+ 0.5M_{bbAa}(i-1)(1-\Phi)F_{BBAa}(i-1) + 0.5M_{bbAa}(i-1)\Phi F_{BBAa}(i-1) + 0.5M_{bbAa}(i-1)F_{BBAA}(i-1)$$
$$+ 0.5M_{bbAA}(i-1)F_{Bbaa}(i-1) + 0.25M_{bbAA}(i-1)(1-\Phi)F_{BbAa}(i-1) + M_{bbAA}(i-1)F_{BBaa}(i-1)$$
$$+ 0.5M_{bbAA}(i-1)(1-\Phi)F_{BBAa}(i-1) + 0.25M_{Bbaa}(i-1)F_{bbAa}(i-1) + 0.5M_{Bbaa}(i-1)F_{bbAA}(i-1)$$
$$+ 0.25M_{Bbaa}(i-1)(1-\Phi)F_{BbAa}(i-1) + 0.5M_{Bbaa}(i-1)\Phi F_{BbAa}(i-1) + 0.5M_{Bbaa}(i-1)F_{BbAA}(i-1)$$
$$+ 0.25M_{Bbaa}(i-1)(1-\Phi)F_{BBAa}(i-1) + 0.5M_{Bbaa}(i-1)\Phi F_{BBAa}(i-1) + 0.5M_{Bbaa}(i-1)F_{BBAA}(i-1)$$

$$
\begin{aligned}
&+ 0.25(1-\Phi)M_{BbAa}(i-1)F_{bbaa}(i-1) + 0.5\Phi M_{BbAa}(i-1)F_{bbaa}(i-1) \\
&+ 0.25(1-\Phi)M_{BbAa}(i-1)F_{bbAa}(i-1) + 0.25\Phi M_{BbAa}(i-1)F_{bbAa}(i-1) \\
&+ 0.25(1-\Phi)M_{BbAa}(i-1)F_{bbAA}(i-1) + 0.25(1-\Phi)M_{BbAa}(i-1)F_{Bbaa}(i-1) \\
&+ 0.5\Phi M_{BbAa}(i-1)F_{Bbaa}(i-1) + 0.25(1-\Phi)M_{BbAa}(i-1)(1-\Phi)F_{BbAa}(i-1) \\
&+ 0.25\Phi M_{BbAa}(i-1)(1-\Phi)F_{BbAa}(i-1) + 0.25(1-\Phi)M_{BbAa}(i-1)\Phi F_{BbAa}(i-1) \\
&+ 0.25(1-\Phi)M_{BbAa}(i-1)F_{BbAA}(i-1) + 0.25(1-\Phi)M_{BbAa}(i-1)F_{BBaa}(i-1) \\
&+ 0.5\Phi M_{BbAa}(i-1)F_{BBaa}(i-1) + 0.25(1-\Phi)M_{BbAa}(i-1)(1-\Phi)F_{BBAa}(i-1) \\
&+ 0.25\Phi M_{BbAa}(i-1)(1-\Phi)F_{BBAa}(i-1) + 0.25(1-\Phi)M_{BbAa}(i-1)\Phi F_{BBAa}(i-1) \\
&+ 0.25(1-\Phi)M_{BbAa}(i-1)F_{BBAA}(i-1) + 0.5M_{BbAA}(i-1)F_{bbaa}(i-1) + 0.25M_{BbAA}(i-1)F_{bbAa}(i-1) \\
&+ 0.5M_{BbAA}(i-1)F_{Bbaa}(i-1) + 0.25M_{BbAA}(i-1)(1-\Phi)F_{BbAa}(i-1) + 0.5M_{BbAA}(i-1)F_{BBaa}(i-1) \\
&+ 0.25M_{BbAA}(i-1)(1-\Phi)F_{BBAa}(i-1) + 0.5M_{BBaa}(i-1)F_{bbAa}(i-1) + M_{BBaa}(i-1)F_{bbAA}(i-1) \\
&+ 0.25M_{BBaa}(i-1)(1-\Phi)F_{BbAa}(i-1) + 0.5M_{BBaa}(i-1)\Phi F_{BbAa}(i-1) + 0.5M_{BBaa}(i-1)F_{BbAA}(i-1) \\
&+ 0.5(1-\Phi)M_{BBAa}(i-1)F_{bbaa}(i-1) + \Phi M_{BBAa}(i-1)F_{bbaa}(i-1) + 0.5(1-\Phi)M_{BBAa}(i-1)F_{bbAa}(i-1) \\
&+ 0.5\Phi M_{BBAa}(i-1)F_{bbAa}(i-1) + 0.5(1-\Phi)M_{BBAa}(i-1)F_{bbAA}(i-1) \\
&+ 0.25(1-\Phi)M_{BBAa}(i-1)F_{Bbaa}(i-1) + 0.5\Phi M_{BBAa}(i-1)F_{Bbaa}(i-1) \\
&+ 0.25(1-\Phi)M_{BBAa}(i-1)(1-\Phi)F_{BbAa}(i-1) + 0.25\Phi M_{BBAa}(i-1)(1-\Phi)F_{BbAa}(i-1) \\
&+ 0.25(1-\Phi)M_{BBAa}(i-1)\Phi F_{BbAa}(i-1) + 0.25(1-\Phi)M_{BBAa}(i-1)F_{BbAA}(i-1) \\
&+ M_{BBAA}(i-1)F_{bbaa}(i-1) + 0.5M_{BBAA}(i-1)F_{bbAa}(i-1) + 0.5M_{BBAA}(i-1)F_{Bbaa}(i-1) \\
&+ 0.25M_{BBAA}(i-1)(1-\Phi)F_{BbAa}(i-1)],
\end{aligned}
$$

$$
\begin{aligned}
M^e_{BbAA} = &\left((1+\delta)\frac{\Omega_{BbAA}}{2}\right)[0.125M_{bbAa}(i-1)(1-\Phi)F_{BbAa}(i-1) + 0.25M_{bbAa}(i-1)\Phi F_{BbAa}(i-1) \\
&+ 0.25M_{bbAa}(i-1)F_{BbAA}(i-1) + 0.25M_{bbAa}(i-1)(1-\Phi)F_{BBAa}(i-1) + 0.5M_{bbAa}(i-1)\Phi F_{BBAa}(i-1) \\
&+ 0.5M_{bbAa}(i-1)F_{BBAA}(i-1) + 0.25M_{bbAA}(i-1)(1-\Phi)F_{BbAa}(i-1) + 0.5M_{bbAA}(i-1)\Phi F_{BbAa}(i-1) \\
&+ 0.5M_{bbAA}(i-1)F_{BbAA}(i-1) + 0.5M_{bbAA}(i-1)(1-\Phi)F_{BBAa}(i-1) + M_{bbAA}(i-1)\Phi F_{BBAa}(i-1) \\
&+ M_{bbAA}(i-1)F_{BBAA}(i-1) + 0.125(1-\Phi)M_{BbAa}(i-1)F_{bbAa}(i-1) + 0.25\Phi M_{BbAa}(i-1)F_{bbAa}(i-1) \\
&+ 0.25(1-\Phi)M_{BbAa}(i-1)F_{bbAA}(i-1) + 0.5\Phi M_{BbAa}(i-1)F_{bbAA}(i-1) \\
&+ 0.125(1-\Phi)M_{BbAa}(i-1)(1-\Phi)F_{BbAa}(i-1) + 0.25\Phi M_{BbAa}(i-1)(1-\Phi)F_{BbAa}(i-1) \\
&+ 0.25(1-\Phi)M_{BbAa}(i-1)\Phi F_{BbAa}(i-1) + 0.5\Phi M_{BbAa}(i-1)\Phi F_{BbAa}(i-1) \\
&+ 0.25(1-\Phi)M_{BbAa}(i-1)F_{BbAA}(i-1) + 0.5\Phi M_{BbAa}(i-1)F_{BbAA}(i-1) \\
&+ 0.125(1-\Phi)M_{BbAa}(i-1)(1-\Phi)F_{BBAa}(i-1) + 0.25\Phi M_{BbAa}(i-1)(1-\Phi)F_{BBAa}(i-1) \\
&+ 0.25(1-\Phi)M_{BbAa}(i-1)\Phi F_{BBAa}(i-1) + 0.5\Phi M_{BbAa}(i-1)\Phi F_{BBAa}(i-1) \\
&+ 0.25(1-\Phi)M_{BbAa}(i-1)F_{BBAA}(i-1) + 0.5\Phi M_{BbAa}(i-1)F_{BBAA}(i-1) + 0.25M_{BbAA}(i-1)F_{bbAa}(i-1) \\
&+ 0.5M_{BbAA}(i-1)F_{bbAA}(i-1) + 0.25M_{BbAA}(i-1)(1-\Phi)F_{BbAa}(i-1) + 0.5M_{BbAA}(i-1)\Phi F_{BbAa}(i-1) \\
&+ 0.5M_{BbAA}(i-1)F_{BbAA}(i-1) + 0.25M_{BbAA}(i-1)(1-\Phi)F_{BBAa}(i-1) + 0.5M_{BbAA}(i-1)\Phi F_{BBAa}(i-1) \\
&+ 0.5M_{BbAA}(i-1)F_{BBAA}(i-1) + 0.25(1-\Phi)M_{BBAa}(i-1)F_{bbAa}(i-1) + 0.5\Phi M_{BBAa}(i-1)F_{bbAa}(i-1) \\
&+ 0.5(1-\Phi)M_{BBAa}(i-1)F_{bbAA}(i-1) + \Phi M_{BBAa}(i-1)F_{bbAA}(i-1) \\
&+ 0.125(1-\Phi)M_{BBAa}(i-1)(1-\Phi)F_{BbAa}(i-1) + 0.25\Phi M_{BBAa}(i-1)(1-\Phi)F_{BbAa}(i-1) \\
&+ 0.25(1-\Phi)M_{BBAa}(i-1)\Phi F_{BbAa}(i-1) + 0.5\Phi M_{BBAa}(i-1)\Phi F_{BbAa}(i-1) \\
&+ 0.25(1-\Phi)M_{BBAa}(i-1)F_{BbAA}(i-1) + 0.5\Phi M_{BBAa}(i-1)F_{BbAA}(i-1) + 0.5M_{BBAA}(i-1)F_{bbAa}(i-1) \\
&+ M_{BBAA}(i-1)F_{bbAA}(i-1) + 0.25M_{BBAA}(i-1)(1-\Phi)F_{BbAa}(i-1) + 0.5M_{BBAA}(i-1)\Phi F_{BbAa}(i-1) \\
&+ 0.5M_{BBAA}(i-1)F_{BbAA}(i-1)],
\end{aligned}
$$

$$
\begin{aligned}
M^e_{BBaa} = &\left(\frac{\Omega_{BBaa}}{2}\right)[0.25M_{Bbaa}(i-1)F_{Bbaa}(i-1) + 0.125M_{Bbaa}(i-1)(1-\Phi)F_{BbAa}(i-1) \\
&+ 0.5M_{Bbaa}(i-1)F_{BBaa}(i-1) + 0.25M_{Bbaa}(i-1)(1-\Phi)F_{BBAa}(i-1) \\
&+ 0.125(1-\Phi)M_{BbAa}(i-1)F_{Bbaa}(i-1) + 0.0625(1-\Phi)M_{BbAa}(i-1)(1-\Phi)F_{BbAa}(i-1) \\
&+ 0.25(1-\Phi)M_{BbAa}(i-1)F_{BBaa}(i-1) + 0.125(1-\Phi)M_{BbAa}(i-1)(1-\Phi)F_{BBAa}(i-1) \\
&+ 0.5M_{BBaa}(i-1)F_{Bbaa}(i-1) + 0.25M_{BBaa}(i-1)(1-\Phi)F_{BbAa}(i-1) + M_{BBaa}(i-1)F_{BBaa}(i-1)
\end{aligned}
$$

$$+ 0.5M_{BBaa}(i-1)(1-\Phi)F_{BBAa}(i-1) + 0.25(1-\Phi)M_{BBAa}(i-1)F_{Bbaa}(i-1)$$
$$+ 0.125(1-\Phi)M_{BBAa}(i-1)(1-\Phi)F_{BbAa}(i-1) + 0.5(1-\Phi)M_{BBAa}(i-1)F_{BBaa}(i-1)$$
$$+ 0.25(1-\Phi)M_{BBAa}(i-1)(1-\Phi)F_{BBAa}(i-1)],$$

$$M^e_{BBAa} = \left((1+\delta_D)\frac{\Omega_{BBAa}}{2}\right)[0.125M_{Bbaa}(i-1)(1-\Phi)F_{BbAa}(i-1) + 0.25M_{Bbaa}(i-1)\Phi F_{BbAa}(i-1)$$
$$+ 0.25M_{Bbaa}(i-1)F_{BbAA}(i-1) + 0.25M_{Bbaa}(i-1)(1-\Phi)F_{BBAa}(i-1) + 0.5M_{Bbaa}(i-1)\Phi F_{BBAa}(i-1)$$
$$+ 0.5M_{Bbaa}(i-1)F_{BBAA}(i-1) + 0.125(1-\Phi)M_{BbAa}(i-1)F_{Bbaa}(i-1) + 0.25\Phi M_{BbAa}(i-1)F_{Bbaa}(i-1)$$
$$+ 0.125(1-\Phi)M_{BbAa}(i-1)(1-\Phi)F_{BbAa}(i-1) + 0.125\Phi M_{BbAa}(i-1)(1-\Phi)F_{BbAa}(i-1)$$
$$+ 0.125(1-\Phi)M_{BbAa}(i-1)\Phi F_{BbAa}(i-1) + 0.125(1-\Phi)M_{BbAa}(i-1)F_{BbAA}(i-1)$$
$$+ 0.25(1-\Phi)M_{BbAa}(i-1)F_{BBaa}(i-1) + 0.5\Phi M_{BbAa}(i-1)F_{BBaa}(i-1)$$
$$+ 0.25(1-\Phi)M_{BbAa}(i-1)(1-\Phi)F_{BBAa}(i-1) + 0.25\Phi M_{BbAa}(i-1)(1-\Phi)F_{BBAa}(i-1)$$
$$+ 0.125(1-\Phi)M_{BbAa}(i-1)\Phi F_{BBAa}(i-1) + 0.25(1-\Phi)M_{BbAa}(i-1)F_{BBAA}(i-1)$$
$$+ 0.25M_{BbAA}(i-1)F_{Bbaa}(i-1) + 0.125M_{BbAA}(i-1)(1-\Phi)F_{BbAa}(i-1) + 0.5M_{BbAA}(i-1)F_{BBaa}(i-1)$$
$$+ 0.25M_{BbAA}(i-1)(1-\Phi)F_{BBAa}(i-1) + 0.25M_{BBaa}(i-1)(1-\Phi)F_{BbAa}(i-1)$$
$$+ 0.5M_{BBaa}(i-1)\Phi F_{BbAa}(i-1) + 0.5M_{BBaa}(i-1)F_{BbAA}(i-1) + 0.5M_{BBaa}(i-1)(1-\Phi)F_{BBAa}(i-1)$$
$$+ M_{BBaa}(i-1)\Phi F_{BBAa}(i-1) + M_{BBaa}(i-1)F_{BBAA}(i-1) + 0.25(1-\Phi)M_{BBAa}(i-1)F_{Bbaa}(i-1)$$
$$+ 0.5\Phi M_{BBAa}(i-1)F_{Bbaa}(i-1) + 0.25(1-\Phi)M_{BBAa}(i-1)(1-\Phi)F_{BbAa}(i-1)$$
$$+ 0.25\Phi M_{BBAa}(i-1)(1-\Phi)F_{BbAa}(i-1) + 0.25(1-\Phi)M_{BBAa}(i-1)\Phi F_{BbAa}(i-1)$$
$$+ 0.25(1-\Phi)M_{BBAa}(i-1)F_{BbAA}(i-1) + 0.5(1-\Phi)M_{BBAa}(i-1)F_{BBaa}(i-1)$$
$$+ \Phi M_{BBAa}(i-1)F_{BBaa}(i-1) + 0.5(1-\Phi)M_{BBAa}(i-1)(1-\Phi)F_{BBAa}(i-1)$$
$$+ 0.5\Phi M_{BBAa}(i-1)(1-\Phi)F_{BBAa}(i-1) + 0.5(1-\Phi)M_{BBAa}(i-1)\Phi F_{BBAa}(i-1)$$
$$+ 0.5(1-\Phi)M_{BBAa}(i-1)F_{BBAA}(i-1) + 0.5M_{BBAA}(i-1)F_{Bbaa}(i-1)$$
$$+ 0.25M_{BBAA}(i-1)(1-\Phi)F_{BbAa}(i-1) + M_{BBAA}(i-1)F_{BBaa}(i-1)$$
$$+ 0.5M_{BBAA}(i-1)(1-\Phi)F_{BBAa}(i-1)],$$

$$M^e_{BBAA} = \left((1+\delta)\frac{\Omega_{BBAA}}{2}\right)[0.0625(1-\Phi)M_{BbAa}(i-1)(1-\Phi)F_{BbAa}(i-1)$$
$$+ 0.125\Phi M_{BbAa}(i-1)(1-\Phi)F_{BbAa}(i-1) + 0.125(1-\Phi)M_{BbAa}(i-1)\Phi F_{BbAa}(i-1)$$
$$+ 0.25\Phi M_{BbAa}(i-1)\Phi F_{BbAa}(i-1) + 0.125(1-\Phi)M_{BbAa}(i-1)F_{BbAA}(i-1)$$
$$+ 0.25\Phi M_{BbAa}(i-1)F_{BbAA}(i-1) + 0.125(1-\Phi)M_{BbAa}(i-1)(1-\Phi)F_{BBAa}(i-1)$$
$$+ 0.25\Phi M_{BbAa}(i-1)(1-\Phi)F_{BBAa}(i-1) + 0.125(1-\Phi)M_{BbAa}(i-1)\Phi F_{BBAa}(i-1)$$
$$+ 0.5\Phi M_{BbAa}(i-1)\Phi F_{BBAa}(i-1) + 0.25(1-\Phi)M_{BbAa}(i-1)F_{BBAA}(i-1)$$
$$+ 0.5\Phi M_{BbAa}(i-1)F_{BBAA}(i-1) + 0.125M_{BbAA}(i-1)(1-\Phi)F_{BbAa}(i-1)$$
$$+ 0.25M_{BbAA}(i-1)\Phi F_{BbAa}(i-1) + 0.25M_{BbAA}(i-1)F_{BbAA}(i-1)$$
$$+ 0.25M_{BbAA}(i-1)(1-\Phi)F_{BBAa}(i-1) + 0.5M_{BbAA}(i-1)\Phi F_{BBAa}(i-1) + 0.5M_{BbAA}(i-1)F_{BBAA}(i-1)$$
$$+ 0.125(1-\Phi)M_{BBAa}(i-1)(1-\Phi)F_{BbAa}(i-1) + 0.25\Phi M_{BBAa}(i-1)(1-\Phi)F_{BbAa}(i-1)$$
$$+ 0.25(1-\Phi)M_{BBAa}(i-1)\Phi F_{BbAa}(i-1) + 0.5\Phi M_{BBAa}(i-1)\Phi F_{BbAa}(i-1)$$
$$+ 0.25(1-\Phi)M_{BBAa}(i-1)F_{BbAA}(i-1) + 0.5\Phi M_{BBAa}(i-1)F_{BbAA}(i-1)$$
$$+ 0.25(1-\Phi)M_{BBAa}(i-1)(1-\Phi)F_{BBAa}(i-1) + 0.5\Phi M_{BBAa}(i-1)(1-\Phi)F_{BBAa}(i-1)$$
$$+ 0.5(1-\Phi)M_{BBAa}(i-1)\Phi F_{BBAa}(i-1) + \Phi M_{BBAa}(i-1)\Phi F_{BBAa}(i-1)$$
$$+ 0.5(1-\Phi)M_{BBAa}(i-1)F_{BBAA}(i-1) + \Phi M_{BBAa}(i-1)F_{BBAA}(i-1)$$
$$+ 0.25M_{BBAA}(i-1)(1-\Phi)F_{BbAa}(i-1) + 0.5M_{BBAA}(i-1)\Phi F_{BbAa}(i-1) + 0.5M_{BBAA}(i-1)F_{BbAA}(i-1)$$
$$+ 0.5M_{BBAA}(i-1)(1-\Phi)F_{BBAa}(i-1) + M_{BBAA}(i-1)\Phi F_{BBAa}(i-1)$$
$$+ M_{BBAA}(i-1)F_{BBAA}(i-1)],$$

alongside an identical set of equations for females of each genotype.

The second stage is to then normalise these proportional frequencies to fill the range from zero to one. We begin by calculating the overall fitness of the entire population by summing the proportional genotype

frequencies resulting from the above equations to give:

$$\bar{\Omega} = M^e_{bbaa} + M^e_{bbAa} + M^e_{bbAA} + M^e_{Bbaa} + M^e_{BbAa} + M^e_{BbAA} + M^e_{BBaa} + M^e_{BBAa} + M^e_{BBAA} + F^e_{bbaa} + F^e_{bbAa} + F^e_{bbAA} + F^e_{Bbaa} + F^e_{BbAa} + F^e_{BbAA} + F^e_{BBaa} + F^e_{BBAa} + F^e_{BBAA}.$$

This is then used as a normalising factor to give the final genotype frequencies for a particular generation as follows:

$$M_{bbaa}(i) = M^e_{bbaa}/\bar{\Omega}, \quad M_{bbAa}(i) = M^e_{bbAa}/\bar{\Omega}, \quad M_{bbAA}(i) = M^e_{bbAA}/\bar{\Omega}, \quad M_{Bbaa}(i) = M^e_{Bbaa}/\bar{\Omega},$$

$$M_{BbAa}(i) = M^e_{BbAa}/\bar{\Omega}, \quad M_{BbAA}(i) = M^e_{BbAA}/\bar{\Omega}, \quad M_{BBaa}(i) = M^e_{BBaa}/\bar{\Omega}, \quad M_{BBAa}(i) = M^e_{BBAa}/\bar{\Omega},$$

$$M_{BBAA}(i) = M^e_{BBAA}/\bar{\Omega}, \quad F_{bbaa}(i) = F^e_{bbaa}/\bar{\Omega}, \quad F_{bbAa}(i) = F^e_{bbAa}/\bar{\Omega}, \quad F_{bbAA}(i) = F^e_{bbAA}/\bar{\Omega},$$

$$F_{Bbaa}(i) = F^e_{Bbaa}/\bar{\Omega}, \quad F_{BbAa}(i) = F^e_{BbAa}/\bar{\Omega}, \quad F_{BbAA}(i) = F^e_{BbAA}/\bar{\Omega}, \quad F_{BBaa}(i) = F^e_{BBaa}/\bar{\Omega},$$

$$F_{BBAa}(i) = F^e_{BBAa}/\bar{\Omega}, \quad F_{BBAA}(i) = F^e_{BBAA}/\bar{\Omega}.$$

We then insert these values into the proportional genotype frequency equations in order to calculate the values for the next generation and so on until the desired end point is reached.

Note that in the case of a two population model we consider a non-target population signified by the use of lower case m and f (replacing M and F in the target population). This results in an identical set of equations at all stages above, except for the use of alternate initial conditions and the normalisation process for the non-target population (note that due to the consideration of unidirectional migration, the normalisation process for the target population remains unchanged). Here the altered set of initial conditions for the non-target population represent a fully wild-type population, i.e.

$$m_{bbaa}(1) = 0.50 = f_{bbaa}(1), \quad m_{bbAa}(1) = 0.00 = f_{bbAa}(1), \quad m_{bbAA}(1) = 0.00 = f_{bbAA}(1),$$

$$m_{Bbaa}(1) = 0.00 = f_{Bbaa}(1), \quad m_{BbAa}(1) = 0.00 = f_{BbAa}(1), \quad m_{BbAA}(1) = 0.00 = f_{BbAA}(1),$$

$$m_{BBaa}(1) = 0.00 = f_{BBaa}(1), \quad m_{BBAa}(1) = 0.00 = f_{BBAa}(1), \quad m_{BBAA}(1) = 0.00 = f_{BBAA}(1).$$

The normalisation process for the non-target population is the given by

$$m_{bbaa}(i) = (\mu M^e_{bbaa} + m^e_{bbaa})/\bar{\Omega}_{\text{Mig}}, \quad m_{bbAa}(i) = (\mu M^e_{bbAa} + m^e_{bbAa})/\bar{\Omega}_{\text{Mig}},$$

$$m_{bbAA}(i) = (\mu M^e_{bbAA} + m^e_{bbAA})/\bar{\Omega}_{\text{Mig}}, \quad m_{Bbaa}(i) = (\mu M^e_{bbaa} + m^e_{Bbaa})/\bar{\Omega}_{\text{Mig}},$$

$$m_{BbAa}(i) = (\mu M^e_{bbAa} + m^e_{BbAa})/\bar{\Omega}_{\text{Mig}}, \quad m_{BbAA}(i) = (\mu M^e_{bbAA} + m^e_{BbAA})/\bar{\Omega}_{\text{Mig}},$$

$$m_{BBaa}(i) = (\mu M^e_{bbaa} + m^e_{BBaa})/\bar{\Omega}_{\text{Mig}}, \quad m_{BBAa}(i) = (\mu M^e_{bbAa} + m^e_{BBAa})/\bar{\Omega}_{\text{Mig}},$$

$$m_{BBAA}(i) = (\mu M^e_{bbAA} + m^e_{BBAA})/\bar{\Omega}_{\text{Mig}}, \quad f_{bbaa}(i) = (\mu F^e_{bbaa} + f^e_{bbaa})/\bar{\Omega}_{\text{Mig}},$$

$$f_{bbAa}(i) = (\mu F^e_{bbAa} + f^e_{bbAa})/\bar{\Omega}_{\text{Mig}}, \quad f_{bbAA}(i) = (\mu F^e_{bbAA} + f^e_{bbAA})/\bar{\Omega}_{\text{Mig}},$$

$$f_{Bbaa}(i) = (\mu F^e_{bbaa} + f^e_{Bbaa})/\bar{\Omega}_{\text{Mig}}, \quad f_{BbAa}(i) = (\mu F^e_{bbAa} + f^e_{BbAa})/\bar{\Omega}_{\text{Mig}},$$

$$f_{BbAA}(i) = (\mu F^e_{bbAA} + f^e_{BbAA})/\bar{\Omega}_{\text{Mig}}, \quad f_{BBaa}(i) = (\mu F^e_{bbaa} + f^e_{BBaa})/\bar{\Omega}_{\text{Mig}},$$

$$f_{BBAa}(i) = (\mu F^e_{bbAa} + f^e_{BBAa})/\bar{\Omega}_{\text{Mig}}, \quad f_{BBAA}(i) = (\mu F^e_{bbAA} + f^e_{BBAA})/\bar{\Omega}_{\text{Mig}},$$

where

$$\bar{\Omega}_{\text{Mig}} = \bar{\omega} + \mu\bar{\Omega},$$

and $\bar{\omega}$ denotes the normalisation factor for the non-target population in absence of migration.
