# Supplementary material for: Split drive killer-rescue provides a novel threshold-dependent gene drive
Source: Sci Rep. 2020 Nov 25;10:20520. doi: 10.1038/s41598-020-77544-7 (PMC7689494; doi:10.1038/s41598-020-77544-7)
Supplement: Supplementary file 4 — Supplementary material 4 [file 41598_2020_77544_MOESM4_ESM.pdf]

Split drive killer-rescue provides a novel threshold-dependent gene drive

Matthew P. Edgington[1*], Tim Harvey-Samuel[1] and Luke Alphey[1]

[1]The Pirbright Institute, Ash Road, Woking, Surrey GU24 0NF, UK
*Corresponding author: matt.edgington@pirbright.ac.uk

Supplementary Model 4

We begin by defining a base parameter set for use throughout this study. This is as follows:

- $L = 1$ (full lethal penetrance),
- $\varepsilon_A = 0.85$ (relative to 1 in wild-type individuals - 15% fitness cost),
- $\varepsilon_B = 0.85$ (relative to 1 in wild-type individuals - 15% fitness cost),
- $\varepsilon_C = 0.85$ (relative to 1 in wild-type individuals - 15% fitness cost),
- $\Phi = 0.9$ (90% homing rate).

The relative fitness parameters above are then combined multiplicatively to obtain a single overall relative fitness value for individuals of each genotype (assumed equal for both sexes). These are of the form:

$$\Omega_{ccbbaa} = 1, \quad \Omega_{ccbbAa} = (1-L)\varepsilon_A, \quad \Omega_{ccbbAA} = (1-L)\varepsilon_A^2, \quad \Omega_{ccBbaa} = \varepsilon_B, \quad \Omega_{ccBbAa} = \varepsilon_B\varepsilon_A,$$

$$\Omega_{ccBbAA} = \varepsilon_B\varepsilon_A^2, \quad \Omega_{ccBBaa} = \varepsilon_B^2, \quad \Omega_{ccBBAa} = \varepsilon_B^2\varepsilon_A, \quad \Omega_{ccBBAA} = \varepsilon_B^2\varepsilon_A^2, \quad \Omega_{Ccbbaa} = \varepsilon_C,$$

$$\Omega_{CcbbAa} = (1-L)\varepsilon_C\varepsilon_A, \quad \Omega_{CcbbAA} = (1-L)\varepsilon_C\varepsilon_A^2, \quad \Omega_{CcBbaa} = \varepsilon_C\varepsilon_B, \quad \Omega_{CcBbAa} = \varepsilon_C\varepsilon_B\varepsilon_A, \quad \Omega_{CcBbAA} = \varepsilon_C\varepsilon_B\varepsilon_A^2,$$

$$\Omega_{CcBBaa} = \varepsilon_C\varepsilon_B^2, \quad \Omega_{CcBBAa} = \varepsilon_C\varepsilon_B^2\varepsilon_A, \quad \Omega_{CcBBAA} = \varepsilon_C\varepsilon_B^2\varepsilon_A^2, \quad \Omega_{CCbbaa} = \varepsilon_C^2, \quad \Omega_{CCbbAa} = (1-L)\varepsilon_C^2\varepsilon_A,$$

$$\Omega_{CCbbAA} = (1-L)\varepsilon_C^2\varepsilon_A^2, \quad \Omega_{CCBbaa} = \varepsilon_C^2\varepsilon_B, \quad \Omega_{CCBbAa} = \varepsilon_C^2\varepsilon_B\varepsilon_A, \quad \Omega_{CCBbAA} = \varepsilon_C^2\varepsilon_B\varepsilon_A^2, \quad \Omega_{CCBBaa} = \varepsilon_C^2\varepsilon_B^2,$$

$$\Omega_{CCBBAa} = \varepsilon_C^2\varepsilon_B^2\varepsilon_A, \quad \Omega_{CCBBAA} = \varepsilon_C^2\varepsilon_B^2\varepsilon_A^2.$$

We then define a set of initial conditions representing the genotype proportions in the population at the point of releasing transgenic individuals. For a 1:1 (introduced:wild) release of individuals homozygous for all transgenic constructs, as is most widely considered in this study these are of the form:

$$M_{ccbbaa}(1) = 0.25 = F_{ccbbaa}(1), \quad M_{ccbbAa}(1) = 0.00 = F_{ccbbAa}(1), \quad M_{ccbbAA}(1) = 0.00 = F_{ccbbAA}(1),$$

$$M_{ccBbaa}(1) = 0.00 = F_{ccBbaa}(1), \quad M_{ccBbAa}(1) = 0.00 = F_{ccBbAa}(1), \quad M_{ccBbAA}(1) = 0.00 = F_{ccBbAA}(1),$$

$$M_{ccBBaa}(1) = 0.00 = F_{ccBBaa}(1), \quad M_{ccBBAa}(1) = 0.00 = F_{ccBBAa}(1), \quad M_{ccBBAA}(1) = 0.00 = F_{ccBBAA}(1),$$

$$M_{Ccbbaa}(1) = 0.25 = F_{Ccbbaa}(1), \quad M_{CcbbAa}(1) = 0.00 = F_{CcbbAa}(1), \quad M_{CcbbAA}(1) = 0.00 = F_{CcbbAA}(1),$$

$$M_{CcBbaa}(1) = 0.00 = F_{CcBbaa}(1), \quad M_{CcBbAa}(1) = 0.00 = F_{CcBbAa}(1), \quad M_{CcBbAA}(1) = 0.00 = F_{CcBbAA}(1),$$

$$M_{CcBBaa}(1) = 0.00 = F_{CcBBaa}(1), \quad M_{CcBBAa}(1) = 0.00 = F_{CcBBAa}(1), \quad M_{CcBBAA}(1) = 0.00 = F_{CcBBAA}(1),$$

$$M_{CCbbaa}(1) = 0.25 = F_{CCbbaa}(1), \quad M_{CCbbAa}(1) = 0.00 = F_{CCbbAa}(1), \quad M_{CCbbAA}(1) = 0.00 = F_{CCbbAA}(1),$$

$$M_{CCBbaa}(1) = 0.00 = F_{CCBbaa}(1), \quad M_{CCBbAa}(1) = 0.00 = F_{CCBbAa}(1), \quad M_{CCBbAA}(1) = 0.00 = F_{CCBbAA}(1),$$

$$M_{CCBBaa}(1) = 0.00 = F_{CCBBaa}(1), \quad M_{CCBBAa}(1) = 0.00 = F_{CCBBAa}(1), \quad M_{CCBBAA}(1) = 0.25 = F_{CCBBAA}(1).$$

With the above definitions then iteratively calculate the genotype frequencies in each subsequent generation. This process is conducted in a two step manner. The first stage is to calculate the proportional frequencies for each genotype (and for each sex), which is achieved using:

$$\mathrm{M}^e_{ccbbaa} = \left(\frac{\Omega_{ccbbaa}}{2}\right)[M_{ccbbaa}(i-1)F_{ccbbaa}(i-1) + 0.5M_{ccbbaa}(i-1)F_{ccbbAa}(i-1) + 0.5M_{ccbbaa}(i-1)F_{ccBbaa}(i-1) + 0.25M_{ccbbaa}(i-1)(1-\Phi)F_{ccBbAa}(i-1) + 0.5M_{ccbbaa}(i-1)F_{Ccbbaa}(i-1) + 0.25M_{ccbbaa}(i-1)F_{CcbbAa}(i-1) + 0.25M_{ccbbaa}(i-1)(1-\Phi)F_{CcBbaa}(i-1) + 0.125M_{ccbbaa}(i-1)(1-\Phi)(1-\Phi)F_{CcBbAa}(i-1) + 0.5M_{ccbbAa}(i-1)F_{ccbbaa}(i-1) + 0.25M_{ccbbAa}(i-1)F_{ccbbAa}(i-1) + 0.25M_{ccbbAa}(i-1)F_{ccBbaa}(i-1) + 0.125M_{ccbbAa}(i-1)(1-\Phi)F_{ccBbAa}(i-1) + 0.25M_{ccbbAa}(i-1)F_{Ccbbaa}(i-1) + 0.125M_{ccbbAa}(i-1)F_{CcbbAa}(i-1) + 0.125M_{ccbbAa}(i-1)(1-\Phi)F_{CcBbaa}(i-1) + 0.0625M_{ccbbAa}(i-1)(1-\Phi)(1-\Phi)F_{CcBbAa}(i-1) + 0.5M_{ccBbaa}(i-1)F_{ccbbaa}(i-1) + 0.25M_{ccBbaa}(i-1)F_{ccbbAa}(i-1) + 0.25M_{ccBbaa}(i-1)F_{ccBbaa}(i-1) + 0.125M_{ccBbaa}(i-1)(1-\Phi)F_{ccBbAa}(i-1) + 0.25M_{ccBbaa}(i-1)F_{Ccbbaa}(i-1) + 0.125M_{ccBbaa}(i-1)F_{CcbbAa}(i-1) + 0.125M_{ccBbaa}(i-1)(1-\Phi)F_{CcBbaa}(i-1) + 0.0625M_{ccBbaa}(i-1)(1-\Phi)(1-\Phi)F_{CcBbAa}(i-1) + 0.25(1-\Phi)M_{ccBbAa}(i-1)F_{ccbbaa}(i-1) + 0.125(1-\Phi)M_{ccBbAa}(i-1)F_{ccbbAa}(i-1) + 0.125(1-\Phi)M_{ccBbAa}(i-1)F_{ccBbaa}(i-1) + 0.0625(1-\Phi)M_{ccBbAa}(i-1)(1-\Phi)F_{ccBbAa}(i-1) + 0.125(1-\Phi)M_{ccBbAa}(i-1)F_{Ccbbaa}(i-1) + 0.0625(1-\Phi)M_{ccBbAa}(i-1)F_{CcbbAa}(i-1) + 0.0625(1-\Phi)M_{ccBbAa}(i-1)(1-\Phi)F_{CcBbaa}(i-1) + 0.03125(1-\Phi)M_{ccBbAa}(i-1)(1-\Phi)(1-\Phi)F_{CcBbAa}(i-1) + 0.5M_{Ccbbaa}(i-1)F_{ccbbaa}(i-1) + 0.25M_{Ccbbaa}(i-1)F_{ccbbAa}(i-1) + 0.25M_{Ccbbaa}(i-1)F_{ccBbaa}(i-1) + 0.125M_{Ccbbaa}(i-1)(1-\Phi)F_{ccBbAa}(i-1) + 0.25M_{Ccbbaa}(i-1)F_{Ccbbaa}(i-1) + 0.125M_{Ccbbaa}(i-1)F_{CcbbAa}(i-1) + 0.125M_{Ccbbaa}(i-1)(1-\Phi)F_{CcBbaa}(i-1) + 0.0625M_{Ccbbaa}(i-1)(1-\Phi)(1-\Phi)F_{CcBbAa}(i-1) + 0.25M_{CcbbAa}(i-1)F_{ccbbaa}(i-1) + 0.125M_{CcbbAa}(i-1)F_{ccbbAa}(i-1) + 0.125M_{CcbbAa}(i-1)F_{ccBbaa}(i-1) + 0.0625M_{CcbbAa}(i-1)(1-\Phi)F_{ccBbAa}(i-1) + 0.125M_{CcbbAa}(i-1)F_{Ccbbaa}(i-1) + 0.0625M_{CcbbAa}(i-1)F_{CcbbAa}(i-1) + 0.0625M_{CcbbAa}(i-1)(1-\Phi)F_{CcBbaa}(i-1) + 0.03125M_{CcbbAa}(i-1)(1-\Phi)(1-\Phi)F_{CcBbAa}(i-1) + 0.25(1-\Phi)M_{CcBbaa}(i-1)F_{ccbbaa}(i-1) + 0.125(1-\Phi)M_{CcBbaa}(i-1)F_{ccbbAa}(i-1) + 0.125(1-\Phi)M_{CcBbaa}(i-1)F_{ccBbaa}(i-1) + 0.0625(1-\Phi)M_{CcBbaa}(i-1)(1-\Phi)F_{ccBbAa}(i-1) + 0.125(1-\Phi)M_{CcBbaa}(i-1)F_{Ccbbaa}(i-1) + 0.0625(1-\Phi)M_{CcBbaa}(i-1)F_{CcbbAa}(i-1) + 0.0625(1-\Phi)M_{CcBbaa}(i-1)(1-\Phi)F_{CcBbaa}(i-1) + 0.03125(1-\Phi)M_{CcBbaa}(i-1)(1-\Phi)(1-\Phi)F_{CcBbAa}(i-1) + 0.125(1-\Phi)(1-\Phi)M_{CcBbAa}(i-1)F_{ccbbaa}(i-1) + 0.0625(1-\Phi)(1-\Phi)M_{CcBbAa}(i-1)F_{ccbbAa}(i-1) + 0.0625(1-\Phi)(1-\Phi)M_{CcBbAa}(i-1)F_{ccBbaa}(i-1) + 0.03125(1-\Phi)(1-\Phi)M_{CcBbAa}(i-1)(1-\Phi)F_{ccBbAa}(i-1) + 0.0625(1-\Phi)(1-\Phi)M_{CcBbAa}(i-1)F_{Ccbbaa}(i-1) + 0.03125(1-\Phi)(1-\Phi)M_{CcBbAa}(i-1)F_{CcbbAa}(i-1) + 0.03125(1-\Phi)(1-\Phi)M_{CcBbAa}(i-1)(1-\Phi)F_{CcBbaa}(i-1) + 0.015625(1-\Phi)(1-\Phi)M_{CcBbAa}(i-1)(1-\Phi)(1-\Phi)F_{CcBbAa}(i-1)],$$

$$\mathrm{M}^e_{ccbbAa} = \left(\frac{\Omega_{ccbbAa}}{2}\right)[0.5M_{ccbbaa}(i-1)F_{ccbbAa}(i-1) + M_{ccbbaa}(i-1)F_{ccbbAA}(i-1) + 0.25M_{ccbbaa}(i-1)(1-\Phi)F_{ccBbAa}(i-1) + 0.5M_{ccbbaa}(i-1)\Phi F_{ccBbAa}(i-1) + 0.5M_{ccbbaa}(i-1)F_{ccBbAA}(i-1) + 0.25M_{ccbbaa}(i-1)F_{CcbbAa}(i-1) + 0.5M_{ccbbaa}(i-1)F_{CcbbAA}(i-1) + 0.125M_{ccbbaa}(i-1)(1-\Phi)(1-\Phi)F_{CcBbAa}(i-1) + 0.25M_{ccbbaa}(i-1)(1-\Phi)\Phi F_{CcBbAa}(i-1) + 0.25M_{ccbbaa}(i-1)(1-\Phi)F_{CcBbAA}(i-1) + 0.5M_{ccbbAa}(i-1)F_{ccbbaa}(i-1) + 0.5M_{ccbbAa}(i-1)F_{ccbbAa}(i-1) + 0.5M_{ccbbAa}(i-1)F_{ccbbAA}(i-1) + 0.25M_{ccbbAa}(i-1)F_{ccBbaa}(i-1) + 0.25M_{ccbbAa}(i-1)(1-\Phi)F_{ccBbAa}(i-1) + 0.25M_{ccbbAa}(i-1)\Phi F_{ccBbAa}(i-1) + 0.25M_{ccbbAa}(i-1)F_{ccBbAA}(i-1) + 0.25M_{ccbbAa}(i-1)F_{Ccbbaa}(i-1) + 0.25M_{ccbbAa}(i-1)F_{CcbbAa}(i-1) + 0.25M_{ccbbAa}(i-1)F_{CcbbAA}(i-1) + 0.125M_{ccbbAa}(i-1)(1-\Phi)F_{CcBbaa}(i-1) + 0.125M_{ccbbAa}(i-1)(1-\Phi)(1-\Phi)F_{CcBbAa}(i-1) + 0.125M_{ccbbAa}(i-1)(1-\Phi)\Phi F_{CcBbAa}(i-1) + 0.125M_{ccbbAa}(i-1)(1-\Phi)F_{CcBbAA}(i-1) + M_{ccbbAA}(i-1)F_{ccbbaa}(i-1) + 0.5M_{ccbbAA}(i-1)F_{ccbbAa}(i-1) + 0.5M_{ccbbAA}(i-1)F_{ccBbaa}(i-1) + 0.25M_{ccbbAA}(i-1)(1-\Phi)F_{ccBbAa}(i-1) + 0.5M_{ccbbAA}(i-1)F_{Ccbbaa}(i-1) + 0.25M_{ccbbAA}(i-1)F_{CcbbAa}(i-1) + 0.25M_{ccbbAA}(i-1)(1-\Phi)F_{CcBbaa}(i-1) + 0.125M_{ccbbAA}(i-1)(1-\Phi)(1-\Phi)F_{CcBbAa}(i-1) + 0.25M_{ccBbaa}(i-1)F_{ccbbAa}(i-1) + 0.5M_{ccBbaa}(i-1)F_{ccbbAA}(i-1) + 0.125M_{ccBbaa}(i-1)(1-\Phi)F_{ccBbAa}(i-1) + 0.25M_{ccBbaa}(i-1)\Phi F_{ccBbAa}(i-1) + 0.25M_{ccBbaa}(i-1)F_{ccBbAA}(i-1) + 0.125M_{ccBbaa}(i-1)F_{CcbbAa}(i-1) + 0.25M_{ccBbaa}(i-1)F_{CcbbAA}(i-1) + 0.0625M_{ccBbaa}(i-1)(1-\Phi)(1-\Phi)F_{CcBbAa}(i-1) + 0.125M_{ccBbaa}(i-1)(1-\Phi)\Phi F_{CcBbAa}(i-1) + 0.125M_{ccBbaa}(i-1)(1-\Phi)F_{CcBbAA}(i-1) + 0.25(1-\Phi)M_{ccBbAa}(i-1)F_{ccbbaa}(i-1) + 0.25(1-\Phi)M_{ccBbAa}(i-1)F_{ccbbAa}(i-1) + 0.25(1-\Phi)M_{ccBbAa}(i-1)F_{ccbbAA}(i-1) + 0.125(1-\Phi)M_{ccBbAa}(i-1)F_{ccBbaa}(i-1) + 0.125(1-\Phi)M_{ccBbAa}(i-1)(1-\Phi)F_{ccBbAa}(i-1) + 0.125(1-\Phi)M_{ccBbAa}(i-1)\Phi F_{ccBbAa}(i-1) + 0.125(1-\Phi)M_{ccBbAa}(i-1)F_{ccBbAA}(i-1) + 0.125(1-\Phi)M_{ccBbAa}(i-1)F_{Ccbbaa}(i-1) + 0.125(1-\Phi)M_{ccBbAa}(i-1)F_{CcbbAa}(i-1) + 0.125(1-\Phi)M_{ccBbAa}(i-1)F_{CcbbAA}(i-1) + 0.0625(1-\Phi)M_{ccBbAa}(i-1)(1-\Phi)F_{CcBbaa}(i-1) + 0.0625(1-\Phi)M_{ccBbAa}(i-1)(1-\Phi)(1-\Phi)F_{CcBbAa}(i-1) + 0.0625(1-\Phi)M_{ccBbAa}(i-1)(1-\Phi)\Phi F_{CcBbAa}(i-1) + 0.0625(1-\Phi)M_{ccBbAa}(i-1)(1-\Phi)F_{CcBbAA}(i-1) + 0.5\Phi M_{ccBbAa}(i-1)F_{ccbbaa}(i-1) + 0.25\Phi M_{ccBbAa}(i-1)F_{ccbbAa}(i-1) + 0.25\Phi M_{ccBbAa}(i-1)F_{ccBbaa}(i-1) + 0.125\Phi M_{ccBbAa}(i-1)(1-\Phi)F_{ccBbAa}(i-1) + 0.25\Phi M_{ccBbAa}(i-1)F_{Ccbbaa}(i-1) + 0.125\Phi M_{ccBbAa}(i-1)F_{CcbbAa}(i-1) + 0.125\Phi M_{ccBbAa}(i-1)(1-\Phi)F_{CcBbaa}(i-1) + 0.0625\Phi M_{ccBbAa}(i-1)(1-\Phi)(1-\Phi)F_{CcBbAa}(i-1) + 0.5M_{ccBbAA}(i-1)F_{ccbbaa}(i-1) + 0.25M_{ccBbAA}(i-1)F_{ccbbAa}(i-1) + 0.25M_{ccBbAA}(i-1)F_{ccBbaa}(i-1) + 0.125M_{ccBbAA}(i-1)(1-\Phi)F_{ccBbAa}(i-1) + 0.25M_{ccBbAA}(i-1)F_{Ccbbaa}(i-1) + 0.125M_{ccBbAA}(i-1)F_{CcbbAa}(i-1) + 0.125M_{ccBbAA}(i-1)(1-\Phi)F_{CcBbaa}(i-1) + 0.0625M_{ccBbAA}(i-1)(1-\Phi)(1-\Phi)F_{CcBbAa}(i-1) + 0.25M_{Ccbbaa}(i-1)F_{ccbbAa}(i-1) + 0.5M_{Ccbbaa}(i-1)F_{ccbbAA}(i-1) + 0.125M_{Ccbbaa}(i-1)(1-\Phi)F_{ccBbAa}(i-$$

$1)+0.25M_{Ccbbaa}(i-1)\Phi F_{ccBbAa}(i-1)+0.25M_{Ccbbaa}(i-1)F_{ccBbAA}(i-1)+0.125M_{Ccbbaa}(i-1)F_{CcbbAa}(i-$
$1)+0.25M_{Ccbbaa}(i-1)F_{CcbbAA}(i-1)+0.0625M_{Ccbbaa}(i-1)(1-\Phi)(1-\Phi)F_{CcBbAa}(i-1)+0.125M_{Ccbbaa}(i-$
$1)(1-\Phi)\Phi F_{CcBbAa}(i-1)+0.125M_{Ccbbaa}(i-1)(1-\Phi)F_{CcBbAA}(i-1)+0.25M_{CcbbAa}(i-1)F_{ccbbaa}(i-$
$1)+0.25M_{CcbbAa}(i-1)F_{ccbbAa}(i-1)+0.25M_{CcbbAa}(i-1)F_{ccbbAA}(i-1)+0.125M_{CcbbAa}(i-1)F_{ccBbaa}(i-$
$1)+0.125M_{CcbbAa}(i-1)(1-\Phi)F_{ccBbAa}(i-1)+0.125M_{CcbbAa}(i-1)\Phi F_{ccBbAa}(i-1)+0.125M_{CcbbAa}(i-$
$1)F_{ccBbAA}(i-1)+0.125M_{CcbbAa}(i-1)F_{Ccbbaa}(i-1)+0.125M_{CcbbAa}(i-1)F_{CcbbAa}(i-1)+0.125M_{CcbbAa}(i-$
$1)F_{CcbbAA}(i-1)+0.0625M_{CcbbAa}(i-1)(1-\Phi)F_{CcBbaa}(i-1)+0.0625M_{CcbbAa}(i-1)(1-\Phi)(1-\Phi)F_{CcBbAa}(i-$
$1)+0.0625M_{CcbbAa}(i-1)(1-\Phi)\Phi F_{CcBbAa}(i-1)+0.0625M_{CcbbAa}(i-1)(1-\Phi)F_{CcBbAA}(i-1)+0.5M_{CcbbAA}(i-$
$1)F_{ccbbaa}(i-1)+0.25M_{CcbbAA}(i-1)F_{ccbbAa}(i-1)+0.25M_{CcbbAA}(i-1)F_{ccBbaa}(i-1)+0.125M_{CcbbAA}(i-$
$1)(1-\Phi)F_{ccBbAa}(i-1)+0.25M_{CcbbAA}(i-1)F_{Ccbbaa}(i-1)+0.125M_{CcbbAA}(i-1)F_{CcbbAa}(i-1)+$
$0.125M_{CcbbAA}(i-1)(1-\Phi)F_{CcBbaa}(i-1)+0.0625M_{CcbbAA}(i-1)(1-\Phi)(1-\Phi)F_{CcBbAa}(i-1)+0.125(1-$
$\Phi)M_{CcBbaa}(i-1)F_{ccbbAa}(i-1)+0.25(1-\Phi)M_{CcBbaa}(i-1)F_{ccbbAA}(i-1)+0.0625(1-\Phi)M_{CcBbaa}(i-1)(1-$
$\Phi)F_{ccBbAa}(i-1)+0.125(1-\Phi)M_{CcBbaa}(i-1)\Phi F_{ccBbAa}(i-1)+0.125(1-\Phi)M_{CcBbaa}(i-1)F_{ccBbAA}(i-$
$1)+0.0625(1-\Phi)M_{CcBbaa}(i-1)F_{CcbbAa}(i-1)+0.125(1-\Phi)M_{CcBbaa}(i-1)F_{CcbbAA}(i-1)+0.03125(1-$
$\Phi)M_{CcBbaa}(i-1)(1-\Phi)(1-\Phi)F_{CcBbAa}(i-1)+0.0625(1-\Phi)M_{CcBbaa}(i-1)(1-\Phi)\Phi F_{CcBbAa}(i-1)+$
$0.0625(1-\Phi)M_{CcBbaa}(i-1)(1-\Phi)F_{CcBbAA}(i-1)+0.125(1-\Phi)(1-\Phi)M_{CcBbAa}(i-1)F_{ccbbaa}(i-1)+$
$0.125(1-\Phi)(1-\Phi)M_{CcBbAa}(i-1)F_{ccbbAa}(i-1)+0.125(1-\Phi)(1-\Phi)M_{CcBbAa}(i-1)F_{ccbbAA}(i-1)+0.0625(1-$
$\Phi)(1-\Phi)M_{CcBbAa}(i-1)F_{ccBbaa}(i-1)+0.0625(1-\Phi)(1-\Phi)M_{CcBbAa}(i-1)(1-\Phi)F_{ccBbAa}(i-1)+0.0625(1-$
$\Phi)(1-\Phi)M_{CcBbAa}(i-1)\Phi F_{ccBbAa}(i-1)+0.0625(1-\Phi)(1-\Phi)M_{CcBbAa}(i-1)F_{ccBbAA}(i-1)+0.0625(1-$
$\Phi)(1-\Phi)M_{CcBbAa}(i-1)F_{Ccbbaa}(i-1)+0.0625(1-\Phi)(1-\Phi)M_{CcBbAa}(i-1)F_{CcbbAa}(i-1)+0.0625(1-$
$\Phi)(1-\Phi)M_{CcBbAa}(i-1)F_{CcbbAA}(i-1)+0.03125(1-\Phi)(1-\Phi)M_{CcBbAa}(i-1)(1-\Phi)F_{CcBbaa}(i-1)+$
$0.03125(1-\Phi)(1-\Phi)M_{CcBbAa}(i-1)(1-\Phi)(1-\Phi)F_{CcBbAa}(i-1)+0.03125(1-\Phi)(1-\Phi)M_{CcBbAa}(i-1)(1-$
$\Phi)\Phi F_{CcBbAa}(i-1)+0.03125(1-\Phi)(1-\Phi)M_{CcBbAa}(i-1)(1-\Phi)F_{CcBbAA}(i-1)+0.25(1-\Phi)\Phi M_{CcBbAa}(i-$
$1)F_{ccbbaa}(i-1)+0.125(1-\Phi)\Phi M_{CcBbAa}(i-1)F_{ccbbAa}(i-1)+0.125(1-\Phi)\Phi M_{CcBbAa}(i-1)F_{ccBbaa}(i-1)+$
$0.0625(1-\Phi)\Phi M_{CcBbAa}(i-1)(1-\Phi)F_{ccBbAa}(i-1)+0.125(1-\Phi)\Phi M_{CcBbAa}(i-1)F_{Ccbbaa}(i-1)+0.0625(1-$
$\Phi)\Phi M_{CcBbAa}(i-1)F_{CcbbAa}(i-1)+0.0625(1-\Phi)\Phi M_{CcBbAa}(i-1)(1-\Phi)F_{CcBbaa}(i-1)+0.03125(1-$
$\Phi)\Phi M_{CcBbAa}(i-1)(1-\Phi)(1-\Phi)F_{CcBbAa}(i-1)+0.25(1-\Phi)M_{CcBbAA}(i-1)F_{ccbbaa}(i-1)+0.125(1-$
$\Phi)M_{CcBbAA}(i-1)F_{ccbbAa}(i-1)+0.125(1-\Phi)M_{CcBbAA}(i-1)F_{ccBbaa}(i-1)+0.0625(1-\Phi)M_{CcBbAA}(i-$
$1)(1-\Phi)F_{ccBbAa}(i-1)+0.125(1-\Phi)M_{CcBbAA}(i-1)F_{Ccbbaa}(i-1)+0.0625(1-\Phi)M_{CcBbAA}(i-1)F_{CcbbAa}(i-$
$1)+0.0625(1-\Phi)M_{CcBbAA}(i-1)(1-\Phi)F_{CcBbaa}(i-1)+0.03125(1-\Phi)M_{CcBbAA}(i-1)(1-\Phi)(1-$
$\Phi)F_{CcBbAa}(i-1)],$

$\mathrm{M}^e_{ccbbAA}=\left(\frac{\bar{\Omega}_{ccbbAA}}{2}\right)[0.25M_{ccbbAa}(i-1)F_{ccbbAa}(i-1)+0.5M_{ccbbAa}(i-1)F_{ccbbAA}(i-1)+0.125M_{ccbbAa}(i-$
$1)(1-\Phi)F_{ccBbAa}(i-1)+0.25M_{ccbbAa}(i-1)\Phi F_{ccBbAa}(i-1)+0.25M_{ccbbAa}(i-1)F_{ccBbAA}(i-1)+0.125M_{ccbbAa}(i-$
$1)F_{CcbbAa}(i-1)+0.25M_{ccbbAa}(i-1)F_{CcbbAA}(i-1)+0.0625M_{ccbbAa}(i-1)(1-\Phi)(1-\Phi)F_{CcBbAa}(i-1)+$
$0.125M_{ccbbAa}(i-1)(1-\Phi)\Phi F_{CcBbAa}(i-1)+0.125M_{ccbbAa}(i-1)(1-\Phi)F_{CcBbAA}(i-1)+0.5M_{ccbbAA}(i-$
$1)F_{ccbbAa}(i-1)+M_{ccbbAA}(i-1)F_{ccbbAA}(i-1)+0.25M_{ccbbAA}(i-1)(1-\Phi)F_{ccBbAa}(i-1)+0.5M_{ccbbAA}(i-$
$1)\Phi F_{ccBbAa}(i-1)+0.5M_{ccbbAA}(i-1)F_{ccBbAA}(i-1)+0.25M_{ccbbAA}(i-1)F_{CcbbAa}(i-1)+0.5M_{ccbbAA}(i-$
$1)F_{CcbbAA}(i-1)+0.125M_{ccbbAA}(i-1)(1-\Phi)(1-\Phi)F_{CcBbAa}(i-1)+0.25M_{ccbbAA}(i-1)(1-\Phi)\Phi F_{CcBbAa}(i-$
$1)+0.25M_{ccbbAA}(i-1)(1-\Phi)F_{CcBbAA}(i-1)+0.125(1-\Phi)M_{ccBbAa}(i-1)F_{ccbbAa}(i-1)+0.25(1-$
$\Phi)M_{ccBbAa}(i-1)F_{ccbbAA}(i-1)+0.0625(1-\Phi)M_{ccBbAa}(i-1)(1-\Phi)F_{ccBbAa}(i-1)+0.125(1-\Phi)M_{ccBbAa}(i-$
$1)\Phi F_{ccBbAa}(i-1)+0.125(1-\Phi)M_{ccBbAa}(i-1)F_{ccBbAA}(i-1)+0.0625(1-\Phi)M_{ccBbAa}(i-1)F_{CcbbAa}(i-$
$1)+0.125(1-\Phi)M_{ccBbAa}(i-1)F_{CcbbAA}(i-1)+0.03125(1-\Phi)M_{ccBbAa}(i-1)(1-\Phi)(1-\Phi)F_{CcBbAa}(i-$
$1)+0.0625(1-\Phi)M_{ccBbAa}(i-1)(1-\Phi)\Phi F_{CcBbAa}(i-1)+0.0625(1-\Phi)M_{ccBbAa}(i-1)(1-\Phi)F_{CcBbAA}(i-$
$1)+0.25\Phi M_{ccBbAa}(i-1)F_{ccbbAa}(i-1)+0.5\Phi M_{ccBbAa}(i-1)F_{ccbbAA}(i-1)+0.125\Phi M_{ccBbAa}(i-1)(1-$
$\Phi)F_{ccBbAa}(i-1)+0.25\Phi M_{ccBbAa}(i-1)\Phi F_{ccBbAa}(i-1)+0.25\Phi M_{ccBbAa}(i-1)F_{ccBbAA}(i-1)+0.125\Phi M_{ccBbAa}(i-$
$1)F_{CcbbAa}(i-1)+0.25\Phi M_{ccBbAa}(i-1)F_{CcbbAA}(i-1)+0.0625\Phi M_{ccBbAa}(i-1)(1-\Phi)(1-\Phi)F_{CcBbAa}(i-$
$1)+0.125\Phi M_{ccBbAa}(i-1)(1-\Phi)\Phi F_{CcBbAa}(i-1)+0.125\Phi M_{ccBbAa}(i-1)(1-\Phi)F_{CcBbAA}(i-1)+$
$0.25M_{ccBbAA}(i-1)F_{ccbbAa}(i-1)+0.5M_{ccBbAA}(i-1)F_{ccbbAA}(i-1)+0.125M_{ccBbAA}(i-1)(1-\Phi)F_{ccBbAa}(i-$
$1)+0.25M_{ccBbAA}(i-1)\Phi F_{ccBbAa}(i-1)+0.25M_{ccBbAA}(i-1)F_{ccBbAA}(i-1)+0.125M_{ccBbAA}(i-1)F_{CcbbAa}(i-$
$1)+0.25M_{ccBbAA}(i-1)F_{CcbbAA}(i-1)+0.0625M_{ccBbAA}(i-1)(1-\Phi)(1-\Phi)F_{CcBbAa}(i-1)+0.125M_{ccBbAA}(i-$
$1)(1-\Phi)\Phi F_{CcBbAa}(i-1)+0.125M_{ccBbAA}(i-1)(1-\Phi)F_{CcBbAA}(i-1)+0.125M_{CcbbAa}(i-1)F_{ccbbAa}(i-$
$1)+0.25M_{CcbbAa}(i-1)F_{ccbbAA}(i-1)+0.0625M_{CcbbAa}(i-1)(1-\Phi)F_{ccBbAa}(i-1)+0.125M_{CcbbAa}(i-$
$1)\Phi F_{ccBbAa}(i-1)+0.125M_{CcbbAa}(i-1)F_{ccBbAA}(i-1)+0.0625M_{CcbbAa}(i-1)F_{CcbbAa}(i-1)+0.125M_{CcbbAa}(i-$
$1)F_{CcbbAA}(i-1)+0.03125M_{CcbbAa}(i-1)(1-\Phi)(1-\Phi)F_{CcBbAa}(i-1)+0.0625M_{CcbbAa}(i-1)(1-$
$\Phi)\Phi F_{CcBbAa}(i-1)+0.0625M_{CcbbAa}(i-1)(1-\Phi)F_{CcBbAA}(i-1)+0.25M_{CcbbAA}(i-1)F_{ccbbAa}(i-1)+$
$0.5M_{CcbbAA}(i-1)F_{ccbbAA}(i-1)+0.125M_{CcbbAA}(i-1)(1-\Phi)F_{ccBbAa}(i-1)+0.25M_{CcbbAA}(i-1)\Phi F_{ccBbAa}(i-$

$1)+0.25M_{CcbbAA}(i-1)F_{ccBbAA}(i-1)+0.125M_{CcbbAA}(i-1)F_{CcbbAa}(i-1)+0.25M_{CcbbAA}(i-1)F_{CcbbAA}(i-$
$1)+0.0625M_{CcbbAA}(i-1)(1-\Phi)(1-\Phi)F_{CcBbAa}(i-1)+0.125M_{CcbbAA}(i-1)(1-\Phi)\Phi F_{CcBbAa}(i-1)+$
$0.125M_{CcbbAA}(i-1)(1-\Phi)F_{CcBbAA}(i-1)+0.0625(1-\Phi)(1-\Phi)M_{CcBbAa}(i-1)F_{ccbbAa}(i-1)+0.125(1-$
$\Phi)(1-\Phi)M_{CcBbAa}(i-1)F_{ccbbAA}(i-1)+0.03125(1-\Phi)(1-\Phi)M_{CcBbAa}(i-1)(1-\Phi)F_{ccBbAa}(i-1)+$
$0.0625(1-\Phi)(1-\Phi)M_{CcBbAa}(i-1)\Phi F_{ccBbAa}(i-1)+0.0625(1-\Phi)(1-\Phi)M_{CcBbAa}(i-1)F_{ccBbAA}(i-1)+$
$0.03125(1-\Phi)(1-\Phi)M_{CcBbAa}(i-1)F_{CcbbAa}(i-1)+0.0625(1-\Phi)(1-\Phi)M_{CcBbAa}(i-1)F_{CcbbAA}(i-1)+$
$0.015625(1-\Phi)(1-\Phi)M_{CcBbAa}(i-1)(1-\Phi)(1-\Phi)F_{CcBbAa}(i-1)+0.03125(1-\Phi)(1-\Phi)M_{CcBbAa}(i-$
$1)(1-\Phi)\Phi F_{CcBbAa}(i-1)+0.03125(1-\Phi)(1-\Phi)M_{CcBbAa}(i-1)(1-\Phi)F_{CcBbAA}(i-1)+0.125(1-$
$\Phi)\Phi M_{CcBbAa}(i-1)F_{ccbbAa}(i-1)+0.25(1-\Phi)\Phi M_{CcBbAa}(i-1)F_{ccbbAA}(i-1)+0.0625(1-\Phi)\Phi M_{CcBbAa}(i-$
$1)(1-\Phi)F_{ccBbAa}(i-1)+0.125(1-\Phi)\Phi M_{CcBbAa}(i-1)\Phi F_{ccBbAa}(i-1)+0.125(1-\Phi)\Phi M_{CcBbAa}(i-$
$1)F_{ccBbAA}(i-1)+0.0625(1-\Phi)\Phi M_{CcBbAa}(i-1)F_{CcbbAa}(i-1)+0.125(1-\Phi)\Phi M_{CcBbAa}(i-1)F_{CcbbAA}(i-$
$1)+0.03125(1-\Phi)\Phi M_{CcBbAa}(i-1)(1-\Phi)(1-\Phi)F_{CcBbAa}(i-1)+0.0625(1-\Phi)\Phi M_{CcBbAa}(i-1)(1-$
$\Phi)\Phi F_{CcBbAa}(i-1)+0.0625(1-\Phi)\Phi M_{CcBbAa}(i-1)(1-\Phi)F_{CcBbAA}(i-1)+0.125(1-\Phi)M_{CcBbAA}(i-$
$1)F_{ccbbAa}(i-1)+0.25(1-\Phi)M_{CcBbAA}(i-1)F_{ccbbAA}(i-1)+0.0625(1-\Phi)M_{CcBbAA}(i-1)(1-\Phi)F_{ccBbAa}(i-$
$1)+0.125(1-\Phi)M_{CcBbAA}(i-1)\Phi F_{ccBbAa}(i-1)+0.125(1-\Phi)M_{CcBbAA}(i-1)F_{ccBbAA}(i-1)+0.0625(1-$
$\Phi)M_{CcBbAA}(i-1)F_{CcbbAa}(i-1)+0.125(1-\Phi)M_{CcBbAA}(i-1)F_{CcbbAA}(i-1)+0.03125(1-\Phi)M_{CcBbAA}(i-$
$1)(1-\Phi)(1-\Phi)F_{CcBbAa}(i-1)+0.0625(1-\Phi)M_{CcBbAA}(i-1)(1-\Phi)\Phi F_{CcBbAa}(i-1)+0.0625(1-$
$\Phi)M_{CcBbAA}(i-1)(1-\Phi)F_{CcBbAA}(i-1)],$

$\mathrm{M}^{e}_{ccBbaa}=\left(\frac{\bar{\Omega}_{ccBbaa}}{2}\right)[0.5M_{ccbbaa}(i-1)F_{ccBbaa}(i-1)+0.25M_{ccbbaa}(i-1)(1-\Phi)F_{ccBbAa}(i-1)+$
$M_{ccbbaa}(i-1)F_{ccBBaa}(i-1)+0.5M_{ccbbaa}(i-1)(1-\Phi)F_{ccBBAa}(i-1)+0.25M_{ccbbaa}(i-1)(1-\Phi)F_{CcBbaa}(i-$
$1)+0.5M_{ccbbaa}(i-1)\Phi F_{CcBbaa}(i-1)+0.125M_{ccbbaa}(i-1)(1-\Phi)(1-\Phi)F_{CcBbAa}(i-1)+0.25M_{ccbbaa}(i-$
$1)\Phi(1-\Phi)F_{CcBbAa}(i-1)+0.5M_{ccbbaa}(i-1)F_{CcBBaa}(i-1)+0.25M_{ccbbaa}(i-1)(1-\Phi)F_{CcBBAa}(i-1)+$
$0.25M_{ccbbAa}(i-1)F_{ccBbaa}(i-1)+0.125M_{ccbbAa}(i-1)(1-\Phi)F_{ccBbAa}(i-1)+0.5M_{ccbbAa}(i-1)F_{ccBBaa}(i-$
$1)+0.25M_{ccbbAa}(i-1)(1-\Phi)F_{ccBBAa}(i-1)+0.125M_{ccbbAa}(i-1)(1-\Phi)F_{CcBbaa}(i-1)+0.25M_{ccbbAa}(i-$
$1)\Phi F_{CcBbaa}(i-1)+0.0625M_{ccbbAa}(i-1)(1-\Phi)(1-\Phi)F_{CcBbAa}(i-1)+0.125M_{ccbbAa}(i-1)\Phi(1-$
$\Phi)F_{CcBbAa}(i-1)+0.25M_{ccbbAa}(i-1)F_{CcBBaa}(i-1)+0.125M_{ccbbAa}(i-1)(1-\Phi)F_{CcBBAa}(i-1)+$
$0.5M_{ccBbaa}(i-1)F_{ccbbaa}(i-1)+0.25M_{ccBbaa}(i-1)F_{ccbbAa}(i-1)+0.5M_{ccBbaa}(i-1)F_{ccBbaa}(i-1)+$
$0.25M_{ccBbaa}(i-1)(1-\Phi)F_{ccBbAa}(i-1)+0.5M_{ccBbaa}(i-1)F_{ccBBaa}(i-1)+0.25M_{ccBbaa}(i-1)(1-$
$\Phi)F_{ccBBAa}(i-1)+0.25M_{ccBbaa}(i-1)F_{Ccbbaa}(i-1)+0.125M_{ccBbaa}(i-1)F_{CcbbAa}(i-1)+0.25M_{ccBbaa}(i-$
$1)(1-\Phi)F_{CcBbaa}(i-1)+0.25M_{ccBbaa}(i-1)\Phi F_{CcBbaa}(i-1)+0.125M_{ccBbaa}(i-1)(1-\Phi)(1-\Phi)F_{CcBbAa}(i-$
$1)+0.125M_{ccBbaa}(i-1)\Phi(1-\Phi)F_{CcBbAa}(i-1)+0.25M_{ccBbaa}(i-1)F_{CcBBaa}(i-1)+0.125M_{ccBbaa}(i-$
$1)(1-\Phi)F_{CcBBAa}(i-1)+0.25(1-\Phi)M_{ccBbAa}(i-1)F_{ccbbaa}(i-1)+0.125(1-\Phi)M_{ccBbAa}(i-1)F_{ccbbAa}(i-$
$1)+0.25(1-\Phi)M_{ccBbAa}(i-1)F_{ccBbaa}(i-1)+0.125(1-\Phi)M_{ccBbAa}(i-1)(1-\Phi)F_{ccBbAa}(i-1)+0.25(1-$
$\Phi)M_{ccBbAa}(i-1)F_{ccBBaa}(i-1)+0.125(1-\Phi)M_{ccBbAa}(i-1)(1-\Phi)F_{ccBBAa}(i-1)+0.125(1-\Phi)M_{ccBbAa}(i-$
$1)F_{Ccbbaa}(i-1)+0.0625(1-\Phi)M_{ccBbAa}(i-1)F_{CcbbAa}(i-1)+0.125(1-\Phi)M_{ccBbAa}(i-1)(1-\Phi)F_{CcBbaa}(i-$
$1)+0.125(1-\Phi)M_{ccBbAa}(i-1)\Phi F_{CcBbaa}(i-1)+0.0625(1-\Phi)M_{ccBbAa}(i-1)(1-\Phi)(1-\Phi)F_{CcBbAa}(i-1)+$
$0.0625(1-\Phi)M_{ccBbAa}(i-1)\Phi(1-\Phi)F_{CcBbAa}(i-1)+0.125(1-\Phi)M_{ccBbAa}(i-1)F_{CcBBaa}(i-1)+0.0625(1-$
$\Phi)M_{ccBbAa}(i-1)(1-\Phi)F_{CcBBAa}(i-1)+M_{ccBBaa}(i-1)F_{ccbbaa}(i-1)+0.5M_{ccBBaa}(i-1)F_{ccbbAa}(i-1)+$
$0.5M_{ccBBaa}(i-1)F_{ccBbaa}(i-1)+0.25M_{ccBBaa}(i-1)(1-\Phi)F_{ccBbAa}(i-1)+0.5M_{ccBBaa}(i-1)F_{Ccbbaa}(i-$
$1)+0.25M_{ccBBaa}(i-1)F_{CcbbAa}(i-1)+0.25M_{ccBBaa}(i-1)(1-\Phi)F_{CcBbaa}(i-1)+0.125M_{ccBBaa}(i-1)(1-$
$\Phi)(1-\Phi)F_{CcBbAa}(i-1)+0.5(1-\Phi)M_{ccBBAa}(i-1)F_{ccbbaa}(i-1)+0.25(1-\Phi)M_{ccBBAa}(i-1)F_{ccbbAa}(i-$
$1)+0.25(1-\Phi)M_{ccBBAa}(i-1)F_{ccBbaa}(i-1)+0.125(1-\Phi)M_{ccBBAa}(i-1)(1-\Phi)F_{ccBbAa}(i-1)+0.25(1-$
$\Phi)M_{ccBBAa}(i-1)F_{Ccbbaa}(i-1)+0.125(1-\Phi)M_{ccBBAa}(i-1)F_{CcbbAa}(i-1)+0.125(1-\Phi)M_{ccBBAa}(i-$
$1)(1-\Phi)F_{CcBbaa}(i-1)+0.0625(1-\Phi)M_{ccBBAa}(i-1)(1-\Phi)(1-\Phi)F_{CcBbAa}(i-1)+0.25M_{Ccbbaa}(i-$
$1)F_{ccBbaa}(i-1)+0.125M_{Ccbbaa}(i-1)(1-\Phi)F_{ccBbAa}(i-1)+0.5M_{Ccbbaa}(i-1)F_{ccBBaa}(i-1)+0.25M_{Ccbbaa}(i-$
$1)(1-\Phi)F_{ccBBAa}(i-1)+0.125M_{Ccbbaa}(i-1)(1-\Phi)F_{CcBbaa}(i-1)+0.25M_{Ccbbaa}(i-1)\Phi F_{CcBbaa}(i-$
$1)+0.0625M_{Ccbbaa}(i-1)(1-\Phi)(1-\Phi)F_{CcBbAa}(i-1)+0.125M_{Ccbbaa}(i-1)\Phi(1-\Phi)F_{CcBbAa}(i-1)+$
$0.25M_{Ccbbaa}(i-1)F_{CcBBaa}(i-1)+0.125M_{Ccbbaa}(i-1)(1-\Phi)F_{CcBBAa}(i-1)+0.125M_{CcbbAa}(i-$
$1)F_{ccBbaa}(i-1)+0.0625M_{CcbbAa}(i-1)(1-\Phi)F_{ccBbAa}(i-1)+0.25M_{CcbbAa}(i-1)F_{ccBBaa}(i-1)+$
$0.125M_{CcbbAa}(i-1)(1-\Phi)F_{ccBBAa}(i-1)+0.0625M_{CcbbAa}(i-1)(1-\Phi)F_{CcBbaa}(i-1)+0.125M_{CcbbAa}(i-$
$1)\Phi F_{CcBbaa}(i-1)+0.03125M_{CcbbAa}(i-1)(1-\Phi)(1-\Phi)F_{CcBbAa}(i-1)+0.0625M_{CcbbAa}(i-1)\Phi(1-$
$\Phi)F_{CcBbAa}(i-1)+0.125M_{CcbbAa}(i-1)F_{CcBBaa}(i-1)+0.0625M_{CcbbAa}(i-1)(1-\Phi)F_{CcBBAa}(i-1)+$
$0.25(1-\Phi)M_{CcBbaa}(i-1)F_{ccbbaa}(i-1)+0.125(1-\Phi)M_{CcBbaa}(i-1)F_{ccbbAa}(i-1)+0.25(1-\Phi)M_{CcBbaa}(i-$
$1)F_{ccBbaa}(i-1)+0.125(1-\Phi)M_{CcBbaa}(i-1)(1-\Phi)F_{ccBbAa}(i-1)+0.25(1-\Phi)M_{CcBbaa}(i-1)F_{ccBBaa}(i-$
$1)+0.125(1-\Phi)M_{CcBbaa}(i-1)(1-\Phi)F_{ccBBAa}(i-1)+0.125(1-\Phi)M_{CcBbaa}(i-1)F_{Ccbbaa}(i-1)+0.0625(1-$
$\Phi)M_{CcBbaa}(i-1)F_{CcbbAa}(i-1)+0.125(1-\Phi)M_{CcBbaa}(i-1)(1-\Phi)F_{CcBbaa}(i-1)+0.125(1-\Phi)M_{CcBbaa}(i-$

$$
\begin{aligned}
&1)\Phi F_{CcBbaa}(i-1)+0.0625(1-\Phi)M_{CcBbaa}(i-1)(1-\Phi)(1-\Phi)F_{CcBbAa}(i-1)+0.0625(1-\Phi)M_{CcBbaa}(i-\\
&1)\Phi(1-\Phi)F_{CcBbAa}(i-1)+0.125(1-\Phi)M_{CcBbaa}(i-1)F_{CcBBaa}(i-1)+0.0625(1-\Phi)M_{CcBbaa}(i-1)(1-\\
&\Phi)F_{CcBBAa}(i-1)+0.5\Phi M_{CcBbaa}(i-1)F_{ccbbaa}(i-1)+0.25\Phi M_{CcBbaa}(i-1)F_{ccbbAa}(i-1)+0.25\Phi M_{CcBbaa}(i-\\
&1)F_{ccBbaa}(i-1)+0.125\Phi M_{CcBbaa}(i-1)(1-\Phi)F_{ccBbAa}(i-1)+0.25\Phi M_{CcBbaa}(i-1)F_{Ccbbaa}(i-1)+\\
&0.125\Phi M_{CcBbaa}(i-1)F_{CcbbAa}(i-1)+0.125\Phi M_{CcBbaa}(i-1)(1-\Phi)F_{CcBbaa}(i-1)+0.0625\Phi M_{CcBbaa}(i-\\
&1)(1-\Phi)(1-\Phi)F_{CcBbAa}(i-1)+0.125(1-\Phi)(1-\Phi)M_{CcBbAa}(i-1)F_{ccbbaa}(i-1)+0.0625(1-\Phi)(1-\\
&\Phi)M_{CcBbAa}(i-1)F_{ccbbAa}(i-1)+0.125(1-\Phi)(1-\Phi)M_{CcBbAa}(i-1)F_{ccBbaa}(i-1)+0.0625(1-\Phi)(1-\\
&\Phi)M_{CcBbAa}(i-1)(1-\Phi)F_{ccBbAa}(i-1)+0.125(1-\Phi)(1-\Phi)M_{CcBbAa}(i-1)F_{ccBBaa}(i-1)+0.0625(1-\\
&\Phi)(1-\Phi)M_{CcBbAa}(i-1)(1-\Phi)F_{ccBBAa}(i-1)+0.0625(1-\Phi)(1-\Phi)M_{CcBbAa}(i-1)F_{Ccbbaa}(i-1)+\\
&0.03125(1-\Phi)(1-\Phi)M_{CcBbAa}(i-1)F_{CcbbAa}(i-1)+0.0625(1-\Phi)(1-\Phi)M_{CcBbAa}(i-1)(1-\Phi)F_{CcBbaa}(i-\\
&1)+0.0625(1-\Phi)(1-\Phi)M_{CcBbAa}(i-1)\Phi F_{CcBbaa}(i-1)+0.03125(1-\Phi)(1-\Phi)M_{CcBbAa}(i-1)(1-\Phi)(1-\\
&\Phi)F_{CcBbAa}(i-1)+0.03125(1-\Phi)(1-\Phi)M_{CcBbAa}(i-1)\Phi(1-\Phi)F_{CcBbAa}(i-1)+0.0625(1-\Phi)(1-\\
&\Phi)M_{CcBbAa}(i-1)F_{CcBBaa}(i-1)+0.03125(1-\Phi)(1-\Phi)M_{CcBbAa}(i-1)(1-\Phi)F_{CcBBAa}(i-1)+0.25\Phi(1-\\
&\Phi)M_{CcBbAa}(i-1)F_{ccbbaa}(i-1)+0.125\Phi(1-\Phi)M_{CcBbAa}(i-1)F_{ccbbAa}(i-1)+0.125\Phi(1-\Phi)M_{CcBbAa}(i-\\
&1)F_{ccBbaa}(i-1)+0.0625\Phi(1-\Phi)M_{CcBbAa}(i-1)(1-\Phi)F_{ccBbAa}(i-1)+0.125\Phi(1-\Phi)M_{CcBbAa}(i-\\
&1)F_{Ccbbaa}(i-1)+0.0625\Phi(1-\Phi)M_{CcBbAa}(i-1)F_{CcbbAa}(i-1)+0.0625\Phi(1-\Phi)M_{CcBbAa}(i-1)(1-\\
&\Phi)F_{CcBbaa}(i-1)+0.03125\Phi(1-\Phi)M_{CcBbAa}(i-1)(1-\Phi)(1-\Phi)F_{CcBbAa}(i-1)+0.5M_{CcBBaa}(i-1)F_{ccbbaa}(i-\\
&1)+0.25M_{CcBBaa}(i-1)F_{ccbbAa}(i-1)+0.25M_{CcBBaa}(i-1)F_{ccBbaa}(i-1)+0.125M_{CcBBaa}(i-1)(1-\\
&\Phi)F_{ccBbAa}(i-1)+0.25M_{CcBBaa}(i-1)F_{Ccbbaa}(i-1)+0.125M_{CcBBaa}(i-1)F_{CcbbAa}(i-1)+0.125M_{CcBBaa}(i-\\
&1)(1-\Phi)F_{CcBbaa}(i-1)+0.0625M_{CcBBaa}(i-1)(1-\Phi)(1-\Phi)F_{CcBbAa}(i-1)+0.25(1-\Phi)M_{CcBBAa}(i-\\
&1)F_{ccbbaa}(i-1)+0.125(1-\Phi)M_{CcBBAa}(i-1)F_{ccbbAa}(i-1)+0.125(1-\Phi)M_{CcBBAa}(i-1)F_{ccBbaa}(i-\\
&1)+0.0625(1-\Phi)M_{CcBBAa}(i-1)(1-\Phi)F_{ccBbAa}(i-1)+0.125(1-\Phi)M_{CcBBAa}(i-1)F_{Ccbbaa}(i-1)+\\
&0.0625(1-\Phi)M_{CcBBAa}(i-1)F_{CcbbAa}(i-1)+0.0625(1-\Phi)M_{CcBBAa}(i-1)(1-\Phi)F_{CcBbaa}(i-1)+\\
&0.03125(1-\Phi)M_{CcBBAa}(i-1)(1-\Phi)(1-\Phi)F_{CcBbAa}(i-1)],
\end{aligned}
$$

$$
\begin{aligned}
&\mathrm{M}^{e}_{ccBbAa}=\left(\frac{\bar{\Omega}_{ccBbAa}}{2}\right)[0.25M_{ccbbaa}(i-1)(1-\Phi)F_{ccBbAa}(i-1)+0.5M_{ccbbaa}(i-1)\Phi F_{ccBbAa}(i-1)+\\
&0.5M_{ccbbaa}(i-1)F_{ccBbAA}(i-1)+0.5M_{ccbbaa}(i-1)(1-\Phi)F_{ccBBAa}(i-1)+M_{ccbbaa}(i-1)\Phi F_{ccBBAa}(i-\\
&1)+M_{ccbbaa}(i-1)F_{ccBBAA}(i-1)+0.125M_{ccbbaa}(i-1)(1-\Phi)(1-\Phi)F_{CcBbAa}(i-1)+0.25M_{ccbbaa}(i-\\
&1)\Phi(1-\Phi)F_{CcBbAa}(i-1)+0.25M_{ccbbaa}(i-1)(1-\Phi)\Phi F_{CcBbAa}(i-1)+0.5M_{ccbbaa}(i-1)\Phi\Phi F_{CcBbAa}(i-\\
&1)+0.25M_{ccbbaa}(i-1)(1-\Phi)F_{CcBbAA}(i-1)+0.5M_{ccbbaa}(i-1)\Phi F_{CcBbAA}(i-1)+0.25M_{ccbbaa}(i-\\
&1)(1-\Phi)F_{CcBBAa}(i-1)+0.5M_{ccbbaa}(i-1)\Phi F_{CcBBAa}(i-1)+0.5M_{ccbbaa}(i-1)F_{CcBBAA}(i-1)+\\
&0.25M_{ccbbAa}(i-1)F_{ccBbaa}(i-1)+0.25M_{ccbbAa}(i-1)(1-\Phi)F_{ccBbAa}(i-1)+0.25M_{ccbbAa}(i-1)\Phi F_{ccBbAa}(i-\\
&1)+0.25M_{ccbbAa}(i-1)F_{ccBbAA}(i-1)+0.5M_{ccbbAa}(i-1)F_{ccBBaa}(i-1)+0.5M_{ccbbAa}(i-1)(1-\Phi)F_{ccBBAa}(i-\\
&1)+0.5M_{ccbbAa}(i-1)\Phi F_{ccBBAa}(i-1)+0.5M_{ccbbAa}(i-1)F_{ccBBAA}(i-1)+0.125M_{ccbbAa}(i-1)(1-\\
&\Phi)F_{CcBbaa}(i-1)+0.25M_{ccbbAa}(i-1)\Phi F_{CcBbaa}(i-1)+0.125M_{ccbbAa}(i-1)(1-\Phi)(1-\Phi)F_{CcBbAa}(i-1)+\\
&0.25M_{ccbbAa}(i-1)\Phi(1-\Phi)F_{CcBbAa}(i-1)+0.125M_{ccbbAa}(i-1)(1-\Phi)\Phi F_{CcBbAa}(i-1)+0.25M_{ccbbAa}(i-\\
&1)\Phi\Phi F_{CcBbAa}(i-1)+0.125M_{ccbbAa}(i-1)(1-\Phi)F_{CcBbAA}(i-1)+0.25M_{ccbbAa}(i-1)\Phi F_{CcBbAA}(i-\\
&1)+0.25M_{ccbbAa}(i-1)F_{CcBBaa}(i-1)+0.25M_{ccbbAa}(i-1)(1-\Phi)F_{CcBBAa}(i-1)+0.25M_{ccbbAa}(i-\\
&1)\Phi F_{CcBBAa}(i-1)+0.25M_{ccbbAa}(i-1)F_{CcBBAA}(i-1)+0.5M_{ccbbAA}(i-1)F_{ccBbaa}(i-1)+0.25M_{ccbbAA}(i-\\
&1)(1-\Phi)F_{ccBbAa}(i-1)+M_{ccbbAA}(i-1)F_{ccBBaa}(i-1)+0.5M_{ccbbAA}(i-1)(1-\Phi)F_{ccBBAa}(i-1)+\\
&0.25M_{ccbbAA}(i-1)(1-\Phi)F_{CcBbaa}(i-1)+0.5M_{ccbbAA}(i-1)\Phi F_{CcBbaa}(i-1)+0.125M_{ccbbAA}(i-1)(1-\\
&\Phi)(1-\Phi)F_{CcBbAa}(i-1)+0.25M_{ccbbAA}(i-1)\Phi(1-\Phi)F_{CcBbAa}(i-1)+0.5M_{ccbbAA}(i-1)F_{CcBBaa}(i-1)+\\
&0.25M_{ccbbAA}(i-1)(1-\Phi)F_{CcBBAa}(i-1)+0.25M_{ccBbaa}(i-1)F_{ccbbAa}(i-1)+0.5M_{ccBbaa}(i-1)F_{ccbbAA}(i-\\
&1)+0.25M_{ccBbaa}(i-1)(1-\Phi)F_{ccBbAa}(i-1)+0.5M_{ccBbaa}(i-1)\Phi F_{ccBbAa}(i-1)+0.5M_{ccBbaa}(i-\\
&1)F_{ccBbAA}(i-1)+0.25M_{ccBbaa}(i-1)(1-\Phi)F_{ccBBAa}(i-1)+0.5M_{ccBbaa}(i-1)\Phi F_{ccBBAa}(i-1)+\\
&0.5M_{ccBbaa}(i-1)F_{ccBBAA}(i-1)+0.125M_{ccBbaa}(i-1)F_{CcbbAa}(i-1)+0.25M_{ccBbaa}(i-1)F_{CcbbAA}(i-\\
&1)+0.125M_{ccBbaa}(i-1)(1-\Phi)(1-\Phi)F_{CcBbAa}(i-1)+0.125M_{ccBbaa}(i-1)\Phi(1-\Phi)F_{CcBbAa}(i-1)+\\
&0.25M_{ccBbaa}(i-1)(1-\Phi)\Phi F_{CcBbAa}(i-1)+0.25M_{ccBbaa}(i-1)\Phi\Phi F_{CcBbAa}(i-1)+0.25M_{ccBbaa}(i-\\
&1)(1-\Phi)F_{CcBbAA}(i-1)+0.25M_{ccBbaa}(i-1)\Phi F_{CcBbAA}(i-1)+0.125M_{ccBbaa}(i-1)(1-\Phi)F_{CcBBAa}(i-\\
&1)+0.25M_{ccBbaa}(i-1)\Phi F_{CcBBAa}(i-1)+0.25M_{ccBbaa}(i-1)F_{CcBBAA}(i-1)+0.25(1-\Phi)M_{ccBbAa}(i-\\
&1)F_{ccbbaa}(i-1)+0.25(1-\Phi)M_{ccBbAa}(i-1)F_{ccbbAa}(i-1)+0.25(1-\Phi)M_{ccBbAa}(i-1)F_{ccbbAA}(i-1)+\\
&0.25(1-\Phi)M_{ccBbAa}(i-1)F_{ccBbaa}(i-1)+0.25(1-\Phi)M_{ccBbAa}(i-1)(1-\Phi)F_{ccBbAa}(i-1)+0.25(1-\\
&\Phi)M_{ccBbAa}(i-1)\Phi F_{ccBbAa}(i-1)+0.25(1-\Phi)M_{ccBbAa}(i-1)F_{ccBbAA}(i-1)+0.25(1-\Phi)M_{ccBbAa}(i-\\
&1)F_{ccBBaa}(i-1)+0.25(1-\Phi)M_{ccBbAa}(i-1)(1-\Phi)F_{ccBBAa}(i-1)+0.25(1-\Phi)M_{ccBbAa}(i-1)\Phi F_{ccBBAa}(i-\\
&1)+0.25(1-\Phi)M_{ccBbAa}(i-1)F_{ccBBAA}(i-1)+0.125(1-\Phi)M_{ccBbAa}(i-1)F_{Ccbbaa}(i-1)+0.125(1-\\
&\Phi)M_{ccBbAa}(i-1)F_{CcbbAa}(i-1)+0.125(1-\Phi)M_{ccBbAa}(i-1)F_{CcbbAA}(i-1)+0.125(1-\Phi)M_{ccBbAa}(i-\\
&1)(1-\Phi)F_{CcBbaa}(i-1)+0.125(1-\Phi)M_{ccBbAa}(i-1)\Phi F_{CcBbaa}(i-1)+0.125(1-\Phi)M_{ccBbAa}(i-1)(1-
\end{aligned}
$$

$\Phi)(1-\Phi)F_{CcBbAa}(i-1) + 0.125(1-\Phi)M_{ccBbAa}(i-1)\Phi(1-\Phi)F_{CcBbAa}(i-1) + 0.125(1-\Phi)M_{ccBbAa}(i-$
$1)(1-\Phi)\Phi F_{CcBbAa}(i-1) + 0.125(1-\Phi)M_{ccBbAa}(i-1)\Phi\Phi F_{CcBbAa}(i-1) + 0.125(1-\Phi)M_{ccBbAa}(i-1)(1-$
$\Phi)F_{CcBbAA}(i-1) + 0.125(1-\Phi)M_{ccBbAa}(i-1)\Phi F_{CcBbAA}(i-1) + 0.125(1-\Phi)M_{ccBbAa}(i-1)F_{CcBBaa}(i-1) +$
$0.125(1-\Phi)M_{ccBbAa}(i-1)(1-\Phi)F_{CcBBAa}(i-1) + 0.125(1-\Phi)M_{ccBbAa}(i-1)\Phi F_{CcBBAa}(i-1) + 0.125(1-$
$\Phi)M_{ccBbAa}(i-1)F_{CcBBAA}(i-1) + 0.5\Phi M_{ccBbAa}(i-1)F_{ccbbaa}(i-1) + 0.25\Phi M_{ccBbAa}(i-1)F_{ccbbAa}(i-$
$1) + 0.5\Phi M_{ccBbAa}(i-1)F_{ccBbaa}(i-1) + 0.25\Phi M_{ccBbAa}(i-1)(1-\Phi)F_{ccBbAa}(i-1) + 0.5\Phi M_{ccBbAa}(i-$
$1)F_{ccBBaa}(i-1) + 0.25\Phi M_{ccBbAa}(i-1)(1-\Phi)F_{ccBBAa}(i-1) + 0.25\Phi M_{ccBbAa}(i-1)F_{Ccbbaa}(i-1) +$
$0.125\Phi M_{ccBbAa}(i-1)F_{CcbbAa}(i-1) + 0.25\Phi M_{ccBbAa}(i-1)(1-\Phi)F_{CcBbaa}(i-1) + 0.25\Phi M_{ccBbAa}(i-$
$1)\Phi F_{CcBbaa}(i-1) + 0.125\Phi M_{ccBbAa}(i-1)(1-\Phi)(1-\Phi)F_{CcBbAa}(i-1) + 0.125\Phi M_{ccBbAa}(i-1)\Phi(1-$
$\Phi)F_{CcBbAa}(i-1) + 0.25\Phi M_{ccBbAa}(i-1)F_{CcBBaa}(i-1) + 0.125\Phi M_{ccBbAa}(i-1)(1-\Phi)F_{CcBBAa}(i-$
$1) + 0.5M_{ccBbAA}(i-1)F_{ccbbaa}(i-1) + 0.25M_{ccBbAA}(i-1)F_{ccbbAa}(i-1) + 0.5M_{ccBbAA}(i-1)F_{ccBbaa}(i-$
$1) + 0.25M_{ccBbAA}(i-1)(1-\Phi)F_{ccBbAa}(i-1) + 0.5M_{ccBbAA}(i-1)F_{ccBBaa}(i-1) + 0.25M_{ccBbAA}(i-$
$1)(1-\Phi)F_{ccBBAa}(i-1) + 0.25M_{ccBbAA}(i-1)F_{Ccbbaa}(i-1) + 0.125M_{ccBbAA}(i-1)F_{CcbbAa}(i-1) +$
$0.25M_{ccBbAA}(i-1)(1-\Phi)F_{CcBbaa}(i-1) + 0.25M_{ccBbAA}(i-1)\Phi F_{CcBbaa}(i-1) + 0.125M_{ccBbAA}(i-1)(1-$
$\Phi)(1-\Phi)F_{CcBbAa}(i-1) + 0.125M_{ccBbAA}(i-1)\Phi(1-\Phi)F_{CcBbAa}(i-1) + 0.25M_{ccBbAA}(i-1)F_{CcBBaa}(i-1) +$
$0.125M_{ccBbAA}(i-1)(1-\Phi)F_{CcBBAa}(i-1) + 0.5M_{ccBBaa}(i-1)F_{ccbbAa}(i-1) + M_{ccBBaa}(i-1)F_{ccbbAA}(i-$
$1) + 0.25M_{ccBBaa}(i-1)(1-\Phi)F_{ccBbAa}(i-1) + 0.5M_{ccBBaa}(i-1)\Phi F_{ccBbAa}(i-1) + 0.5M_{ccBBaa}(i-$
$1)F_{ccBbAA}(i-1) + 0.25M_{ccBBaa}(i-1)F_{CcbbAa}(i-1) + 0.5M_{ccBBaa}(i-1)F_{CcbbAA}(i-1) + 0.125M_{ccBBaa}(i-$
$1)(1-\Phi)(1-\Phi)F_{CcBbAa}(i-1) + 0.25M_{ccBBaa}(i-1)(1-\Phi)\Phi F_{CcBbAa}(i-1) + 0.25M_{ccBBaa}(i-1)(1-$
$\Phi)F_{CcBbAA}(i-1) + 0.5(1-\Phi)M_{ccBBAa}(i-1)F_{ccbbaa}(i-1) + 0.5(1-\Phi)M_{ccBBAa}(i-1)F_{ccbbAa}(i-$
$1) + 0.5(1-\Phi)M_{ccBBAa}(i-1)F_{ccbbAA}(i-1) + 0.25(1-\Phi)M_{ccBBAa}(i-1)F_{ccBbaa}(i-1) + 0.25(1-$
$\Phi)M_{ccBBAa}(i-1)(1-\Phi)F_{ccBbAa}(i-1) + 0.25(1-\Phi)M_{ccBBAa}(i-1)\Phi F_{ccBbAa}(i-1) + 0.25(1-\Phi)M_{ccBBAa}(i-$
$1)F_{ccBbAA}(i-1) + 0.25(1-\Phi)M_{ccBBAa}(i-1)F_{Ccbbaa}(i-1) + 0.25(1-\Phi)M_{ccBBAa}(i-1)F_{CcbbAa}(i-1) +$
$0.25(1-\Phi)M_{ccBBAa}(i-1)F_{CcbbAA}(i-1) + 0.125(1-\Phi)M_{ccBBAa}(i-1)(1-\Phi)F_{CcBbaa}(i-1) + 0.125(1-$
$\Phi)M_{ccBBAa}(i-1)(1-\Phi)(1-\Phi)F_{CcBbAa}(i-1) + 0.125(1-\Phi)M_{ccBBAa}(i-1)(1-\Phi)\Phi F_{CcBbAa}(i-1) +$
$0.125(1-\Phi)M_{ccBBAa}(i-1)(1-\Phi)F_{CcBbAA}(i-1) + \Phi M_{ccBBAa}(i-1)F_{ccbbaa}(i-1) + 0.5\Phi M_{ccBBAa}(i-$
$1)F_{ccbbAa}(i-1) + 0.5\Phi M_{ccBBAa}(i-1)F_{ccBbaa}(i-1) + 0.25\Phi M_{ccBBAa}(i-1)(1-\Phi)F_{ccBbAa}(i-1) +$
$0.5\Phi M_{ccBBAa}(i-1)F_{Ccbbaa}(i-1) + 0.25\Phi M_{ccBBAa}(i-1)F_{CcbbAa}(i-1) + 0.25\Phi M_{ccBBAa}(i-1)(1-$
$\Phi)F_{CcBbaa}(i-1) + 0.125\Phi M_{ccBBAa}(i-1)(1-\Phi)(1-\Phi)F_{CcBbAa}(i-1) + M_{ccBBAA}(i-1)F_{ccbbaa}(i-$
$1) + 0.5M_{ccBBAA}(i-1)F_{ccbbAa}(i-1) + 0.5M_{ccBBAA}(i-1)F_{ccBbaa}(i-1) + 0.25M_{ccBBAA}(i-1)(1-$
$\Phi)F_{ccBbAa}(i-1) + 0.5M_{ccBBAA}(i-1)F_{Ccbbaa}(i-1) + 0.25M_{ccBBAA}(i-1)F_{CcbbAa}(i-1) + 0.25M_{ccBBAA}(i-$
$1)(1-\Phi)F_{CcBbaa}(i-1) + 0.125M_{ccBBAA}(i-1)(1-\Phi)(1-\Phi)F_{CcBbAa}(i-1) + 0.125M_{Ccbbaa}(i-1)(1-$
$\Phi)F_{ccBbAa}(i-1) + 0.25M_{Ccbbaa}(i-1)\Phi F_{ccBbAa}(i-1) + 0.25M_{Ccbbaa}(i-1)F_{ccBbAA}(i-1) + 0.25M_{Ccbbaa}(i-$
$1)(1-\Phi)F_{ccBBAa}(i-1) + 0.5M_{Ccbbaa}(i-1)\Phi F_{ccBBAa}(i-1) + 0.5M_{Ccbbaa}(i-1)F_{ccBBAA}(i-1) +$
$0.0625M_{Ccbbaa}(i-1)(1-\Phi)(1-\Phi)F_{CcBbAa}(i-1) + 0.125M_{Ccbbaa}(i-1)\Phi(1-\Phi)F_{CcBbAa}(i-1) +$
$0.125M_{Ccbbaa}(i-1)(1-\Phi)\Phi F_{CcBbAa}(i-1) + 0.25M_{Ccbbaa}(i-1)\Phi\Phi F_{CcBbAa}(i-1) + 0.125M_{Ccbbaa}(i-$
$1)(1-\Phi)F_{CcBbAA}(i-1) + 0.25M_{Ccbbaa}(i-1)\Phi F_{CcBbAA}(i-1) + 0.125M_{Ccbbaa}(i-1)(1-\Phi)F_{CcBBAa}(i-1) +$
$0.25M_{Ccbbaa}(i-1)\Phi F_{CcBBAa}(i-1) + 0.25M_{Ccbbaa}(i-1)F_{CcBBAA}(i-1) + 0.125M_{CcbbAa}(i-1)F_{ccBbaa}(i-$
$1) + 0.125M_{CcbbAa}(i-1)(1-\Phi)F_{ccBbAa}(i-1) + 0.125M_{CcbbAa}(i-1)\Phi F_{ccBbAa}(i-1) + 0.125M_{CcbbAa}(i-$
$1)F_{ccBbAA}(i-1) + 0.25M_{CcbbAa}(i-1)F_{ccBBaa}(i-1) + 0.25M_{CcbbAa}(i-1)(1-\Phi)F_{ccBBAa}(i-1) +$
$0.25M_{CcbbAa}(i-1)\Phi F_{ccBBAa}(i-1) + 0.25M_{CcbbAa}(i-1)F_{ccBBAA}(i-1) + 0.0625M_{CcbbAa}(i-1)(1-$
$\Phi)F_{CcBbaa}(i-1) + 0.125M_{CcbbAa}(i-1)\Phi F_{CcBbaa}(i-1) + 0.0625M_{CcbbAa}(i-1)(1-\Phi)(1-\Phi)F_{CcBbAa}(i-$
$1) + 0.125M_{CcbbAa}(i-1)\Phi(1-\Phi)F_{CcBbAa}(i-1) + 0.0625M_{CcbbAa}(i-1)(1-\Phi)\Phi F_{CcBbAa}(i-1) +$
$0.125M_{CcbbAa}(i-1)\Phi\Phi F_{CcBbAa}(i-1) + 0.0625M_{CcbbAa}(i-1)(1-\Phi)F_{CcBbAA}(i-1) + 0.125M_{CcbbAa}(i-$
$1)\Phi F_{CcBbAA}(i-1) + 0.125M_{CcbbAa}(i-1)F_{CcBBaa}(i-1) + 0.125M_{CcbbAa}(i-1)(1-\Phi)F_{CcBBAa}(i-1) +$
$0.125M_{CcbbAa}(i-1)\Phi F_{CcBBAa}(i-1) + 0.125M_{CcbbAa}(i-1)F_{CcBBAA}(i-1) + 0.25M_{CcbbAA}(i-1)F_{ccBbaa}(i-$
$1) + 0.125M_{CcbbAA}(i-1)(1-\Phi)F_{ccBbAa}(i-1) + 0.5M_{CcbbAA}(i-1)F_{ccBBaa}(i-1) + 0.25M_{CcbbAA}(i-$
$1)(1-\Phi)F_{ccBBAa}(i-1) + 0.125M_{CcbbAA}(i-1)(1-\Phi)F_{CcBbaa}(i-1) + 0.25M_{CcbbAA}(i-1)\Phi F_{CcBbaa}(i-$
$1) + 0.0625M_{CcbbAA}(i-1)(1-\Phi)(1-\Phi)F_{CcBbAa}(i-1) + 0.125M_{CcbbAA}(i-1)\Phi(1-\Phi)F_{CcBbAa}(i-1) +$
$0.25M_{CcbbAA}(i-1)F_{CcBBaa}(i-1) + 0.125M_{CcbbAA}(i-1)(1-\Phi)F_{CcBBAa}(i-1) + 0.125(1-\Phi)M_{CcBbaa}(i-$
$1)F_{ccbbAa}(i-1) + 0.25(1-\Phi)M_{CcBbaa}(i-1)F_{ccbbAA}(i-1) + 0.125(1-\Phi)M_{CcBbaa}(i-1)(1-\Phi)F_{ccBbAa}(i-$
$1) + 0.25(1-\Phi)M_{CcBbaa}(i-1)\Phi F_{ccBbAa}(i-1) + 0.25(1-\Phi)M_{CcBbaa}(i-1)F_{ccBbAA}(i-1) + 0.125(1-$
$\Phi)M_{CcBbaa}(i-1)(1-\Phi)F_{ccBBAa}(i-1) + 0.25(1-\Phi)M_{CcBbaa}(i-1)\Phi F_{ccBBAa}(i-1) + 0.25(1-\Phi)M_{CcBbaa}(i-$
$1)F_{ccBBAA}(i-1) + 0.0625(1-\Phi)M_{CcBbaa}(i-1)F_{CcbbAa}(i-1) + 0.125(1-\Phi)M_{CcBbaa}(i-1)F_{CcbbAA}(i-1) +$
$0.0625(1-\Phi)M_{CcBbaa}(i-1)(1-\Phi)(1-\Phi)F_{CcBbAa}(i-1) + 0.0625(1-\Phi)M_{CcBbaa}(i-1)\Phi(1-\Phi)F_{CcBbAa}(i-$
$1) + 0.125(1-\Phi)M_{CcBbaa}(i-1)(1-\Phi)\Phi F_{CcBbAa}(i-1) + 0.125(1-\Phi)M_{CcBbaa}(i-1)\Phi\Phi F_{CcBbAa}(i-$
$1) + 0.125(1-\Phi)M_{CcBbaa}(i-1)(1-\Phi)F_{CcBbAA}(i-1) + 0.125(1-\Phi)M_{CcBbaa}(i-1)\Phi F_{CcBbAA}(i-1) +$

$$
0.0625(1-\Phi)M_{CcBbaa}(i-1)(1-\Phi)F_{CcBBAa}(i-1)+0.125(1-\Phi)M_{CcBbaa}(i-1)\Phi F_{CcBBAa}(i-1)+
0.125(1-\Phi)M_{CcBbaa}(i-1)F_{CcBBAA}(i-1)+0.25\Phi M_{CcBbaa}(i-1)F_{ccbbAa}(i-1)+0.5\Phi M_{CcBbaa}(i-
1)F_{ccbbAA}(i-1)+0.125\Phi M_{CcBbaa}(i-1)(1-\Phi)F_{ccBbAa}(i-1)+0.25\Phi M_{CcBbaa}(i-1)\Phi F_{ccBbAa}(i-1)+
0.25\Phi M_{CcBbaa}(i-1)F_{ccBbAA}(i-1)+0.125\Phi M_{CcBbaa}(i-1)F_{CcbbAa}(i-1)+0.25\Phi M_{CcBbaa}(i-1)F_{CcbbAA}(i-
1)+0.0625\Phi M_{CcBbaa}(i-1)(1-\Phi)(1-\Phi)F_{CcBbAa}(i-1)+0.125\Phi M_{CcBbaa}(i-1)(1-\Phi)\Phi F_{CcBbAa}(i-1)+
0.125\Phi M_{CcBbaa}(i-1)(1-\Phi)F_{CcBbAA}(i-1)+0.125(1-\Phi)(1-\Phi)M_{CcBbAa}(i-1)F_{ccbbaa}(i-1)+0.125(1-
\Phi)(1-\Phi)M_{CcBbAa}(i-1)F_{ccbbAa}(i-1)+0.125(1-\Phi)(1-\Phi)M_{CcBbAa}(i-1)F_{ccbbAA}(i-1)+0.125(1-\Phi)(1-
\Phi)M_{CcBbAa}(i-1)F_{ccBbaa}(i-1)+0.125(1-\Phi)(1-\Phi)M_{CcBbAa}(i-1)(1-\Phi)F_{ccBbAa}(i-1)+0.125(1-\Phi)(1-
\Phi)M_{CcBbAa}(i-1)\Phi F_{ccBbAa}(i-1)+0.125(1-\Phi)(1-\Phi)M_{CcBbAa}(i-1)F_{ccBbAA}(i-1)+0.125(1-\Phi)(1-
\Phi)M_{CcBbAa}(i-1)F_{ccBBaa}(i-1)+0.125(1-\Phi)(1-\Phi)M_{CcBbAa}(i-1)(1-\Phi)F_{ccBBAa}(i-1)+0.125(1-\Phi)(1-
\Phi)M_{CcBbAa}(i-1)\Phi F_{ccBBAa}(i-1)+0.125(1-\Phi)(1-\Phi)M_{CcBbAa}(i-1)F_{ccBBAA}(i-1)+0.0625(1-\Phi)(1-
\Phi)M_{CcBbAa}(i-1)F_{Ccbbaa}(i-1)+0.0625(1-\Phi)(1-\Phi)M_{CcBbAa}(i-1)F_{CcbbAa}(i-1)+0.0625(1-\Phi)(1-
\Phi)M_{CcBbAa}(i-1)F_{CcbbAA}(i-1)+0.0625(1-\Phi)(1-\Phi)M_{CcBbAa}(i-1)(1-\Phi)F_{CcBbaa}(i-1)+0.0625(1-
\Phi)(1-\Phi)M_{CcBbAa}(i-1)\Phi F_{CcBbaa}(i-1)+0.0625(1-\Phi)(1-\Phi)M_{CcBbAa}(i-1)(1-\Phi)(1-\Phi)F_{CcBbAa}(i-
1)+0.0625(1-\Phi)(1-\Phi)M_{CcBbAa}(i-1)\Phi(1-\Phi)F_{CcBbAa}(i-1)+0.0625(1-\Phi)(1-\Phi)M_{CcBbAa}(i-1)(1-
\Phi)\Phi F_{CcBbAa}(i-1)+0.0625(1-\Phi)(1-\Phi)M_{CcBbAa}(i-1)\Phi\Phi F_{CcBbAa}(i-1)+0.0625(1-\Phi)(1-\Phi)M_{CcBbAa}(i-
1)(1-\Phi)F_{CcBbAA}(i-1)+0.0625(1-\Phi)(1-\Phi)M_{CcBbAa}(i-1)\Phi F_{CcBbAA}(i-1)+0.0625(1-\Phi)(1-
\Phi)M_{CcBbAa}(i-1)F_{CcBBaa}(i-1)+0.0625(1-\Phi)(1-\Phi)M_{CcBbAa}(i-1)(1-\Phi)F_{CcBBAa}(i-1)+0.0625(1-
\Phi)(1-\Phi)M_{CcBbAa}(i-1)\Phi F_{CcBBAa}(i-1)+0.0625(1-\Phi)(1-\Phi)M_{CcBbAa}(i-1)F_{CcBBAA}(i-1)+0.25\Phi(1-
\Phi)M_{CcBbAa}(i-1)F_{ccbbaa}(i-1)+0.25\Phi(1-\Phi)M_{CcBbAa}(i-1)F_{ccbbAa}(i-1)+0.25\Phi(1-\Phi)M_{CcBbAa}(i-
1)F_{ccbbAA}(i-1)+0.125\Phi(1-\Phi)M_{CcBbAa}(i-1)F_{ccBbaa}(i-1)+0.125\Phi(1-\Phi)M_{CcBbAa}(i-1)(1-
\Phi)F_{ccBbAa}(i-1)+0.125\Phi(1-\Phi)M_{CcBbAa}(i-1)\Phi F_{ccBbAa}(i-1)+0.125\Phi(1-\Phi)M_{CcBbAa}(i-1)F_{ccBbAA}(i-
1)+0.125\Phi(1-\Phi)M_{CcBbAa}(i-1)F_{Ccbbaa}(i-1)+0.125\Phi(1-\Phi)M_{CcBbAa}(i-1)F_{CcbbAa}(i-1)+0.125\Phi(1-
\Phi)M_{CcBbAa}(i-1)F_{CcbbAA}(i-1)+0.0625\Phi(1-\Phi)M_{CcBbAa}(i-1)(1-\Phi)F_{CcBbaa}(i-1)+0.0625\Phi(1-
\Phi)M_{CcBbAa}(i-1)(1-\Phi)(1-\Phi)F_{CcBbAa}(i-1)+0.0625\Phi(1-\Phi)M_{CcBbAa}(i-1)(1-\Phi)\Phi F_{CcBbAa}(i-1)+
0.0625\Phi(1-\Phi)M_{CcBbAa}(i-1)(1-\Phi)F_{CcBbAA}(i-1)+0.25(1-\Phi)\Phi M_{CcBbAa}(i-1)F_{ccbbaa}(i-1)+0.125(1-
\Phi)\Phi M_{CcBbAa}(i-1)F_{ccbbAa}(i-1)+0.25(1-\Phi)\Phi M_{CcBbAa}(i-1)F_{ccBbaa}(i-1)+0.125(1-\Phi)\Phi M_{CcBbAa}(i-
1)(1-\Phi)F_{ccBbAa}(i-1)+0.25(1-\Phi)\Phi M_{CcBbAa}(i-1)F_{ccBBaa}(i-1)+0.125(1-\Phi)\Phi M_{CcBbAa}(i-
1)(1-\Phi)F_{ccBBAa}(i-1)+0.125(1-\Phi)\Phi M_{CcBbAa}(i-1)F_{Ccbbaa}(i-1)+0.0625(1-\Phi)\Phi M_{CcBbAa}(i-
1)F_{CcbbAa}(i-1)+0.125(1-\Phi)\Phi M_{CcBbAa}(i-1)(1-\Phi)F_{CcBbaa}(i-1)+0.125(1-\Phi)\Phi M_{CcBbAa}(i-
1)\Phi F_{CcBbaa}(i-1)+0.0625(1-\Phi)\Phi M_{CcBbAa}(i-1)(1-\Phi)(1-\Phi)F_{CcBbAa}(i-1)+0.0625(1-\Phi)\Phi M_{CcBbAa}(i-
1)\Phi(1-\Phi)F_{CcBbAa}(i-1)+0.125(1-\Phi)\Phi M_{CcBbAa}(i-1)F_{CcBBaa}(i-1)+0.0625(1-\Phi)\Phi M_{CcBbAa}(i-
1)(1-\Phi)F_{CcBBAa}(i-1)+0.5\Phi\Phi M_{CcBbAa}(i-1)F_{ccbbaa}(i-1)+0.25\Phi\Phi M_{CcBbAa}(i-1)F_{ccbbAa}(i-1)+
0.25\Phi\Phi M_{CcBbAa}(i-1)F_{ccBbaa}(i-1)+0.125\Phi\Phi M_{CcBbAa}(i-1)(1-\Phi)F_{ccBbAa}(i-1)+0.25\Phi\Phi M_{CcBbAa}(i-
1)F_{Ccbbaa}(i-1)+0.125\Phi\Phi M_{CcBbAa}(i-1)F_{CcbbAa}(i-1)+0.125\Phi\Phi M_{CcBbAa}(i-1)(1-\Phi)F_{CcBbaa}(i-1)+
0.0625\Phi\Phi M_{CcBbAa}(i-1)(1-\Phi)(1-\Phi)F_{CcBbAa}(i-1)+0.25(1-\Phi)M_{CcBbAA}(i-1)F_{ccbbaa}(i-1)+0.125(1-
\Phi)M_{CcBbAA}(i-1)F_{ccbbAa}(i-1)+0.25(1-\Phi)M_{CcBbAA}(i-1)F_{ccBbaa}(i-1)+0.125(1-\Phi)M_{CcBbAA}(i-
1)(1-\Phi)F_{ccBbAa}(i-1)+0.25(1-\Phi)M_{CcBbAA}(i-1)F_{ccBBaa}(i-1)+0.125(1-\Phi)M_{CcBbAA}(i-1)(1-
\Phi)F_{ccBBAa}(i-1)+0.125(1-\Phi)M_{CcBbAA}(i-1)F_{Ccbbaa}(i-1)+0.0625(1-\Phi)M_{CcBbAA}(i-1)F_{CcbbAa}(i-
1)+0.125(1-\Phi)M_{CcBbAA}(i-1)(1-\Phi)F_{CcBbaa}(i-1)+0.125(1-\Phi)M_{CcBbAA}(i-1)\Phi F_{CcBbaa}(i-1)+
0.0625(1-\Phi)M_{CcBbAA}(i-1)(1-\Phi)(1-\Phi)F_{CcBbAa}(i-1)+0.0625(1-\Phi)M_{CcBbAA}(i-1)\Phi(1-\Phi)F_{CcBbAa}(i-
1)+0.125(1-\Phi)M_{CcBbAA}(i-1)F_{CcBBaa}(i-1)+0.0625(1-\Phi)M_{CcBbAA}(i-1)(1-\Phi)F_{CcBBAa}(i-1)+
0.5\Phi M_{CcBbAA}(i-1)F_{ccbbaa}(i-1)+0.25\Phi M_{CcBbAA}(i-1)F_{ccbbAa}(i-1)+0.25\Phi M_{CcBbAA}(i-1)F_{ccBbaa}(i-1)+
0.125\Phi M_{CcBbAA}(i-1)(1-\Phi)F_{ccBbAa}(i-1)+0.25\Phi M_{CcBbAA}(i-1)F_{Ccbbaa}(i-1)+0.125\Phi M_{CcBbAA}(i-
1)F_{CcbbAa}(i-1)+0.125\Phi M_{CcBbAA}(i-1)(1-\Phi)F_{CcBbaa}(i-1)+0.0625\Phi M_{CcBbAA}(i-1)(1-\Phi)(1-
\Phi)F_{CcBbAa}(i-1)+0.25M_{CcBBaa}(i-1)F_{ccbbAa}(i-1)+0.5M_{CcBBaa}(i-1)F_{ccbbAA}(i-1)+0.125M_{CcBBaa}(i-
1)(1-\Phi)F_{ccBbAa}(i-1)+0.25M_{CcBBaa}(i-1)\Phi F_{ccBbAa}(i-1)+0.25M_{CcBBaa}(i-1)F_{ccBbAA}(i-1)+
0.125M_{CcBBaa}(i-1)F_{CcbbAa}(i-1)+0.25M_{CcBBaa}(i-1)F_{CcbbAA}(i-1)+0.0625M_{CcBBaa}(i-1)(1-\Phi)(1-
\Phi)F_{CcBbAa}(i-1)+0.125M_{CcBBaa}(i-1)(1-\Phi)\Phi F_{CcBbAa}(i-1)+0.125M_{CcBBaa}(i-1)(1-\Phi)F_{CcBbAA}(i-1)+
0.25(1-\Phi)M_{CcBBAa}(i-1)F_{ccbbaa}(i-1)+0.25(1-\Phi)M_{CcBBAa}(i-1)F_{ccbbAa}(i-1)+0.25(1-\Phi)M_{CcBBAa}(i-
1)F_{ccbbAA}(i-1)+0.125(1-\Phi)M_{CcBBAa}(i-1)F_{ccBbaa}(i-1)+0.125(1-\Phi)M_{CcBBAa}(i-1)(1-\Phi)F_{ccBbAa}(i-
1)+0.125(1-\Phi)M_{CcBBAa}(i-1)\Phi F_{ccBbAa}(i-1)+0.125(1-\Phi)M_{CcBBAa}(i-1)F_{ccBbAA}(i-1)+0.125(1-
\Phi)M_{CcBBAa}(i-1)F_{Ccbbaa}(i-1)+0.125(1-\Phi)M_{CcBBAa}(i-1)F_{CcbbAa}(i-1)+0.125(1-\Phi)M_{CcBBAa}(i-
1)F_{CcbbAA}(i-1)+0.0625(1-\Phi)M_{CcBBAa}(i-1)(1-\Phi)F_{CcBbaa}(i-1)+0.0625(1-\Phi)M_{CcBBAa}(i-1)(1-
\Phi)(1-\Phi)F_{CcBbAa}(i-1)+0.0625(1-\Phi)M_{CcBBAa}(i-1)(1-\Phi)\Phi F_{CcBbAa}(i-1)+0.0625(1-\Phi)M_{CcBBAa}(i-
1)(1-\Phi)F_{CcBbAA}(i-1)+0.5\Phi M_{CcBBAa}(i-1)F_{ccbbaa}(i-1)+0.25\Phi M_{CcBBAa}(i-1)F_{ccbbAa}(i-1)+
0.25\Phi M_{CcBBAa}(i-1)F_{ccBbaa}(i-1)+0.125\Phi M_{CcBBAa}(i-1)(1-\Phi)F_{ccBbAa}(i-1)+0.25\Phi M_{CcBBAa}(i-
$$

$1)F_{Ccbbaa}(i-1)+0.125\Phi M_{CcBBAa}(i-1)F_{CcbbAa}(i-1)+0.125\Phi M_{CcBBAa}(i-1)(1-\Phi)F_{CcBbaa}(i-1)+$
$0.0625\Phi M_{CcBBAa}(i-1)(1-\Phi)(1-\Phi)F_{CcBbAa}(i-1)+0.5M_{CcBBAA}(i-1)F_{ccbbaa}(i-1)+0.25M_{CcBBAA}(i-$
$1)F_{ccbbAa}(i-1)+0.25M_{CcBBAA}(i-1)F_{ccBbaa}(i-1)+0.125M_{CcBBAA}(i-1)(1-\Phi)F_{ccBbAa}(i-1)+$
$0.25M_{CcBBAA}(i-1)F_{Ccbbaa}(i-1)+0.125M_{CcBBAA}(i-1)F_{CcbbAa}(i-1)+0.125M_{CcBBAA}(i-1)(1-$
$\Phi)F_{CcBbaa}(i-1)+0.0625M_{CcBBAA}(i-1)(1-\Phi)(1-\Phi)F_{CcBbAa}(i-1)],$

$\mathrm{M}^{e}_{ccBbAA}=\left(\frac{\bar{\Omega}_{ccBbAA}}{2}\right)[0.125M_{ccbbAa}(i-1)(1-\Phi)F_{ccBbAa}(i-1)+0.25M_{ccbbAa}(i-1)\Phi F_{ccBbAa}(i-1)+$
$0.25M_{ccbbAa}(i-1)F_{ccBbAA}(i-1)+0.25M_{ccbbAa}(i-1)(1-\Phi)F_{ccBBAa}(i-1)+0.5M_{ccbbAa}(i-1)\Phi F_{ccBBAa}(i-$
$1)+0.5M_{ccbbAa}(i-1)F_{ccBBAA}(i-1)+0.0625M_{ccbbAa}(i-1)(1-\Phi)(1-\Phi)F_{CcBbAa}(i-1)+0.125M_{ccbbAa}(i-$
$1)\Phi(1-\Phi)F_{CcBbAa}(i-1)+0.125M_{ccbbAa}(i-1)(1-\Phi)\Phi F_{CcBbAa}(i-1)+0.25M_{ccbbAa}(i-1)\Phi\Phi F_{CcBbAa}(i-$
$1)+0.125M_{ccbbAa}(i-1)(1-\Phi)F_{CcBbAA}(i-1)+0.25M_{ccbbAa}(i-1)\Phi F_{CcBbAA}(i-1)+0.125M_{ccbbAa}(i-$
$1)(1-\Phi)F_{CcBBAa}(i-1)+0.25M_{ccbbAa}(i-1)\Phi F_{CcBBAa}(i-1)+0.25M_{ccbbAa}(i-1)F_{CcBBAA}(i-1)+$
$0.25M_{ccbbAA}(i-1)(1-\Phi)F_{ccBbAa}(i-1)+0.5M_{ccbbAA}(i-1)\Phi F_{ccBbAa}(i-1)+0.5M_{ccbbAA}(i-1)F_{ccBbAA}(i-$
$1)+0.5M_{ccbbAA}(i-1)(1-\Phi)F_{ccBBAa}(i-1)+M_{ccbbAA}(i-1)\Phi F_{ccBBAa}(i-1)+M_{ccbbAA}(i-1)F_{ccBBAA}(i-1)+$
$0.125M_{ccbbAA}(i-1)(1-\Phi)(1-\Phi)F_{CcBbAa}(i-1)+0.25M_{ccbbAA}(i-1)\Phi(1-\Phi)F_{CcBbAa}(i-1)+0.25M_{ccbbAA}(i-$
$1)(1-\Phi)\Phi F_{CcBbAa}(i-1)+0.5M_{ccbbAA}(i-1)\Phi\Phi F_{CcBbAa}(i-1)+0.25M_{ccbbAA}(i-1)(1-\Phi)F_{CcBbAA}(i-$
$1)+0.5M_{ccbbAA}(i-1)\Phi F_{CcBbAA}(i-1)+0.25M_{ccbbAA}(i-1)(1-\Phi)F_{CcBBAa}(i-1)+0.5M_{ccbbAA}(i-$
$1)\Phi F_{CcBBAa}(i-1)+0.5M_{ccbbAA}(i-1)F_{CcBBAA}(i-1)+0.125(1-\Phi)M_{ccBbAa}(i-1)F_{ccbbAa}(i-1)+$
$0.25(1-\Phi)M_{ccBbAa}(i-1)F_{ccbbAA}(i-1)+0.125(1-\Phi)M_{ccBbAa}(i-1)(1-\Phi)F_{ccBbAa}(i-1)+0.25(1-$
$\Phi)M_{ccBbAa}(i-1)\Phi F_{ccBbAa}(i-1)+0.25(1-\Phi)M_{ccBbAa}(i-1)F_{ccBbAA}(i-1)+0.125(1-\Phi)M_{ccBbAa}(i-1)(1-$
$\Phi)F_{ccBBAa}(i-1)+0.25(1-\Phi)M_{ccBbAa}(i-1)\Phi F_{ccBBAa}(i-1)+0.25(1-\Phi)M_{ccBbAa}(i-1)F_{ccBBAA}(i-$
$1)+0.0625(1-\Phi)M_{ccBbAa}(i-1)F_{CcbbAa}(i-1)+0.125(1-\Phi)M_{ccBbAa}(i-1)F_{CcbbAA}(i-1)+0.0625(1-$
$\Phi)M_{ccBbAa}(i-1)(1-\Phi)(1-\Phi)F_{CcBbAa}(i-1)+0.0625(1-\Phi)M_{ccBbAa}(i-1)\Phi(1-\Phi)F_{CcBbAa}(i-$
$1)+0.125(1-\Phi)M_{ccBbAa}(i-1)(1-\Phi)\Phi F_{CcBbAa}(i-1)+0.125(1-\Phi)M_{ccBbAa}(i-1)\Phi\Phi F_{CcBbAa}(i-$
$1)+0.125(1-\Phi)M_{ccBbAa}(i-1)(1-\Phi)F_{CcBbAA}(i-1)+0.125(1-\Phi)M_{ccBbAa}(i-1)\Phi F_{CcBbAA}(i-1)+$
$0.0625(1-\Phi)M_{ccBbAa}(i-1)(1-\Phi)F_{CcBBAa}(i-1)+0.125(1-\Phi)M_{ccBbAa}(i-1)\Phi F_{CcBBAa}(i-1)+0.125(1-$
$\Phi)M_{ccBbAa}(i-1)F_{CcBBAA}(i-1)+0.25\Phi M_{ccBbAa}(i-1)F_{ccbbAa}(i-1)+0.5\Phi M_{ccBbAa}(i-1)F_{ccbbAA}(i-$
$1)+0.25\Phi M_{ccBbAa}(i-1)(1-\Phi)F_{ccBbAa}(i-1)+0.5\Phi M_{ccBbAa}(i-1)\Phi F_{ccBbAa}(i-1)+0.5\Phi M_{ccBbAa}(i-$
$1)F_{ccBbAA}(i-1)+0.25\Phi M_{ccBbAa}(i-1)(1-\Phi)F_{ccBBAa}(i-1)+0.5\Phi M_{ccBbAa}(i-1)\Phi F_{ccBBAa}(i-1)+$
$0.5\Phi M_{ccBbAa}(i-1)F_{ccBBAA}(i-1)+0.125\Phi M_{ccBbAa}(i-1)F_{CcbbAa}(i-1)+0.25\Phi M_{ccBbAa}(i-1)F_{CcbbAA}(i-$
$1)+0.125\Phi M_{ccBbAa}(i-1)(1-\Phi)(1-\Phi)F_{CcBbAa}(i-1)+0.125\Phi M_{ccBbAa}(i-1)\Phi(1-\Phi)F_{CcBbAa}(i-1)+$
$0.25\Phi M_{ccBbAa}(i-1)(1-\Phi)\Phi F_{CcBbAa}(i-1)+0.25\Phi M_{ccBbAa}(i-1)\Phi\Phi F_{CcBbAa}(i-1)+0.25\Phi M_{ccBbAa}(i-$
$1)(1-\Phi)F_{CcBbAA}(i-1)+0.25\Phi M_{ccBbAa}(i-1)\Phi F_{CcBbAA}(i-1)+0.125\Phi M_{ccBbAa}(i-1)(1-\Phi)F_{CcBBAa}(i-$
$1)+0.25\Phi M_{ccBbAa}(i-1)\Phi F_{CcBBAa}(i-1)+0.25\Phi M_{ccBbAa}(i-1)F_{CcBBAA}(i-1)+0.25M_{ccBbAA}(i-$
$1)F_{ccbbAa}(i-1)+0.5M_{ccBbAA}(i-1)F_{ccbbAA}(i-1)+0.25M_{ccBbAA}(i-1)(1-\Phi)F_{ccBbAa}(i-1)+0.5M_{ccBbAA}(i-$
$1)\Phi F_{ccBbAa}(i-1)+0.5M_{ccBbAA}(i-1)F_{ccBbAA}(i-1)+0.25M_{ccBbAA}(i-1)(1-\Phi)F_{ccBBAa}(i-1)+$
$0.5M_{ccBbAA}(i-1)\Phi F_{ccBBAa}(i-1)+0.5M_{ccBbAA}(i-1)F_{ccBBAA}(i-1)+0.125M_{ccBbAA}(i-1)F_{CcbbAa}(i-$
$1)+0.25M_{ccBbAA}(i-1)F_{CcbbAA}(i-1)+0.125M_{ccBbAA}(i-1)(1-\Phi)(1-\Phi)F_{CcBbAa}(i-1)+0.125M_{ccBbAA}(i-$
$1)\Phi(1-\Phi)F_{CcBbAa}(i-1)+0.25M_{ccBbAA}(i-1)(1-\Phi)\Phi F_{CcBbAa}(i-1)+0.25M_{ccBbAA}(i-1)\Phi\Phi F_{CcBbAa}(i-$
$1)+0.25M_{ccBbAA}(i-1)(1-\Phi)F_{CcBbAA}(i-1)+0.25M_{ccBbAA}(i-1)\Phi F_{CcBbAA}(i-1)+0.125M_{ccBbAA}(i-$
$1)(1-\Phi)F_{CcBBAa}(i-1)+0.25M_{ccBbAA}(i-1)\Phi F_{CcBBAa}(i-1)+0.25M_{ccBbAA}(i-1)F_{CcBBAA}(i-1)+$
$0.25(1-\Phi)M_{ccBBAa}(i-1)F_{ccbbAa}(i-1)+0.5(1-\Phi)M_{ccBBAa}(i-1)F_{ccbbAA}(i-1)+0.125(1-\Phi)M_{ccBBAa}(i-$
$1)(1-\Phi)F_{ccBbAa}(i-1)+0.25(1-\Phi)M_{ccBBAa}(i-1)\Phi F_{ccBbAa}(i-1)+0.25(1-\Phi)M_{ccBBAa}(i-1)F_{ccBbAA}(i-$
$1)+0.125(1-\Phi)M_{ccBBAa}(i-1)F_{CcbbAa}(i-1)+0.25(1-\Phi)M_{ccBBAa}(i-1)F_{CcbbAA}(i-1)+0.0625(1-$
$\Phi)M_{ccBBAa}(i-1)(1-\Phi)(1-\Phi)F_{CcBbAa}(i-1)+0.125(1-\Phi)M_{ccBBAa}(i-1)(1-\Phi)\Phi F_{CcBbAa}(i-1)+$
$0.125(1-\Phi)M_{ccBBAa}(i-1)(1-\Phi)F_{CcBbAA}(i-1)+0.5\Phi M_{ccBBAa}(i-1)F_{ccbbAa}(i-1)+\Phi M_{ccBBAa}(i-$
$1)F_{ccbbAA}(i-1)+0.25\Phi M_{ccBBAa}(i-1)(1-\Phi)F_{ccBbAa}(i-1)+0.5\Phi M_{ccBBAa}(i-1)\Phi F_{ccBbAa}(i-1)+$
$0.5\Phi M_{ccBBAa}(i-1)F_{ccBbAA}(i-1)+0.25\Phi M_{ccBBAa}(i-1)F_{CcbbAa}(i-1)+0.5\Phi M_{ccBBAa}(i-1)F_{CcbbAA}(i-$
$1)+0.125\Phi M_{ccBBAa}(i-1)(1-\Phi)(1-\Phi)F_{CcBbAa}(i-1)+0.25\Phi M_{ccBBAa}(i-1)(1-\Phi)\Phi F_{CcBbAa}(i-$
$1)+0.25\Phi M_{ccBBAa}(i-1)(1-\Phi)F_{CcBbAA}(i-1)+0.5M_{ccBBAA}(i-1)F_{ccbbAa}(i-1)+M_{ccBBAA}(i-$
$1)F_{ccbbAA}(i-1)+0.25M_{ccBBAA}(i-1)(1-\Phi)F_{ccBbAa}(i-1)+0.5M_{ccBBAA}(i-1)\Phi F_{ccBbAa}(i-1)+$
$0.5M_{ccBBAA}(i-1)F_{ccBbAA}(i-1)+0.25M_{ccBBAA}(i-1)F_{CcbbAa}(i-1)+0.5M_{ccBBAA}(i-1)F_{CcbbAA}(i-$
$1)+0.125M_{ccBBAA}(i-1)(1-\Phi)(1-\Phi)F_{CcBbAa}(i-1)+0.25M_{ccBBAA}(i-1)(1-\Phi)\Phi F_{CcBbAa}(i-1)+$
$0.25M_{ccBBAA}(i-1)(1-\Phi)F_{CcBbAA}(i-1)+0.0625M_{CcbbAa}(i-1)(1-\Phi)F_{ccBbAa}(i-1)+0.125M_{CcbbAa}(i-$
$1)\Phi F_{ccBbAa}(i-1)+0.125M_{CcbbAa}(i-1)F_{ccBbAA}(i-1)+0.125M_{CcbbAa}(i-1)(1-\Phi)F_{ccBBAa}(i-1)+$
$0.25M_{CcbbAa}(i-1)\Phi F_{ccBBAa}(i-1)+0.25M_{CcbbAa}(i-1)F_{ccBBAA}(i-1)+0.03125M_{CcbbAa}(i-1)(1-\Phi)(1-$
$\Phi)F_{CcBbAa}(i-1)+0.0625M_{CcbbAa}(i-1)\Phi(1-\Phi)F_{CcBbAa}(i-1)+0.0625M_{CcbbAa}(i-1)(1-\Phi)\Phi F_{CcBbAa}(i-$

$1)+0.125M_{CcbbAa}(i-1)\Phi\Phi F_{CcBbAa}(i-1)+0.0625M_{CcbbAa}(i-1)(1-\Phi)F_{CcBbAA}(i-1)+0.125M_{CcbbAa}(i-$
$1)\Phi F_{CcBbAA}(i-1)+0.0625M_{CcbbAa}(i-1)(1-\Phi)F_{CcBBAa}(i-1)+0.125M_{CcbbAa}(i-1)\Phi F_{CcBBAa}(i-$
$1)+0.125M_{CcbbAa}(i-1)F_{CcBBAA}(i-1)+0.125M_{CcbbAA}(i-1)(1-\Phi)F_{ccBbAa}(i-1)+0.25M_{CcbbAA}(i-$
$1)\Phi F_{ccBbAa}(i-1)+0.25M_{CcbbAA}(i-1)F_{ccBbAA}(i-1)+0.25M_{CcbbAA}(i-1)(1-\Phi)F_{ccBBAa}(i-1)+$
$0.5M_{CcbbAA}(i-1)\Phi F_{ccBBAa}(i-1)+0.5M_{CcbbAA}(i-1)F_{ccBBAA}(i-1)+0.0625M_{CcbbAA}(i-1)(1-\Phi)(1-$
$\Phi)F_{CcBbAa}(i-1)+0.125M_{CcbbAA}(i-1)\Phi(1-\Phi)F_{CcBbAa}(i-1)+0.125M_{CcbbAA}(i-1)(1-\Phi)\Phi F_{CcBbAa}(i-$
$1)+0.25M_{CcbbAA}(i-1)\Phi\Phi F_{CcBbAa}(i-1)+0.125M_{CcbbAA}(i-1)(1-\Phi)F_{CcBbAA}(i-1)+0.25M_{CcbbAA}(i-$
$1)\Phi F_{CcBbAA}(i-1)+0.125M_{CcbbAA}(i-1)(1-\Phi)F_{CcBBAa}(i-1)+0.25M_{CcbbAA}(i-1)\Phi F_{CcBBAa}(i-1)+$
$0.25M_{CcbbAA}(i-1)F_{CcBBAA}(i-1)+0.0625(1-\Phi)(1-\Phi)M_{CcBbAa}(i-1)F_{ccbbAa}(i-1)+0.125(1-\Phi)(1-$
$\Phi)M_{CcBbAa}(i-1)F_{ccbbAA}(i-1)+0.0625(1-\Phi)(1-\Phi)M_{CcBbAa}(i-1)(1-\Phi)F_{ccBbAa}(i-1)+0.125(1-\Phi)(1-$
$\Phi)M_{CcBbAa}(i-1)\Phi F_{ccBbAa}(i-1)+0.125(1-\Phi)(1-\Phi)M_{CcBbAa}(i-1)F_{ccBbAA}(i-1)+0.0625(1-\Phi)(1-$
$\Phi)M_{CcBbAa}(i-1)(1-\Phi)F_{ccBBAa}(i-1)+0.125(1-\Phi)(1-\Phi)M_{CcBbAa}(i-1)\Phi F_{ccBBAa}(i-1)+0.125(1-$
$\Phi)(1-\Phi)M_{CcBbAa}(i-1)F_{ccBBAA}(i-1)+0.03125(1-\Phi)(1-\Phi)M_{CcBbAa}(i-1)F_{CcbbAa}(i-1)+0.0625(1-$
$\Phi)(1-\Phi)M_{CcBbAa}(i-1)F_{CcbbAA}(i-1)+0.03125(1-\Phi)(1-\Phi)M_{CcBbAa}(i-1)(1-\Phi)(1-\Phi)F_{CcBbAa}(i-$
$1)+0.03125(1-\Phi)(1-\Phi)M_{CcBbAa}(i-1)\Phi(1-\Phi)F_{CcBbAa}(i-1)+0.0625(1-\Phi)(1-\Phi)M_{CcBbAa}(i-$
$1)(1-\Phi)\Phi F_{CcBbAa}(i-1)+0.0625(1-\Phi)(1-\Phi)M_{CcBbAa}(i-1)\Phi\Phi F_{CcBbAa}(i-1)+0.0625(1-\Phi)(1-$
$\Phi)M_{CcBbAa}(i-1)(1-\Phi)F_{CcBbAA}(i-1)+0.0625(1-\Phi)(1-\Phi)M_{CcBbAa}(i-1)\Phi F_{CcBbAA}(i-1)+0.03125(1-$
$\Phi)(1-\Phi)M_{CcBbAa}(i-1)(1-\Phi)F_{CcBBAa}(i-1)+0.0625(1-\Phi)(1-\Phi)M_{CcBbAa}(i-1)\Phi F_{CcBBAa}(i-$
$1)+0.0625(1-\Phi)(1-\Phi)M_{CcBbAa}(i-1)F_{CcBBAA}(i-1)+0.125\Phi(1-\Phi)M_{CcBbAa}(i-1)F_{ccbbAa}(i-1)+$
$0.25\Phi(1-\Phi)M_{CcBbAa}(i-1)F_{ccbbAA}(i-1)+0.0625\Phi(1-\Phi)M_{CcBbAa}(i-1)(1-\Phi)F_{ccBbAa}(i-1)+0.125\Phi(1-$
$\Phi)M_{CcBbAa}(i-1)\Phi F_{ccBbAa}(i-1)+0.125\Phi(1-\Phi)M_{CcBbAa}(i-1)F_{ccBbAA}(i-1)+0.0625\Phi(1-\Phi)M_{CcBbAa}(i-$
$1)F_{CcbbAa}(i-1)+0.125\Phi(1-\Phi)M_{CcBbAa}(i-1)F_{CcbbAA}(i-1)+0.03125\Phi(1-\Phi)M_{CcBbAa}(i-1)(1-\Phi)(1-$
$\Phi)F_{CcBbAa}(i-1)+0.0625\Phi(1-\Phi)M_{CcBbAa}(i-1)(1-\Phi)\Phi F_{CcBbAa}(i-1)+0.0625\Phi(1-\Phi)M_{CcBbAa}(i-1)(1-$
$\Phi)F_{CcBbAA}(i-1)+0.125(1-\Phi)\Phi M_{CcBbAa}(i-1)F_{ccbbAa}(i-1)+0.25(1-\Phi)\Phi M_{CcBbAa}(i-1)F_{ccbbAA}(i-$
$1)+0.125(1-\Phi)\Phi M_{CcBbAa}(i-1)(1-\Phi)F_{ccBbAa}(i-1)+0.25(1-\Phi)\Phi M_{CcBbAa}(i-1)\Phi F_{ccBbAa}(i-$
$1)+0.25(1-\Phi)\Phi M_{CcBbAa}(i-1)F_{ccBbAA}(i-1)+0.125(1-\Phi)\Phi M_{CcBbAa}(i-1)(1-\Phi)F_{ccBBAa}(i-$
$1)+0.25(1-\Phi)\Phi M_{CcBbAa}(i-1)\Phi F_{ccBBAa}(i-1)+0.25(1-\Phi)\Phi M_{CcBbAa}(i-1)F_{ccBBAA}(i-1)+$
$0.0625(1-\Phi)\Phi M_{CcBbAa}(i-1)F_{CcbbAa}(i-1)+0.125(1-\Phi)\Phi M_{CcBbAa}(i-1)F_{CcbbAA}(i-1)+0.0625(1-$
$\Phi)\Phi M_{CcBbAa}(i-1)(1-\Phi)(1-\Phi)F_{CcBbAa}(i-1)+0.0625(1-\Phi)\Phi M_{CcBbAa}(i-1)\Phi(1-\Phi)F_{CcBbAa}(i-$
$1)+0.125(1-\Phi)\Phi M_{CcBbAa}(i-1)(1-\Phi)\Phi F_{CcBbAa}(i-1)+0.125(1-\Phi)\Phi M_{CcBbAa}(i-1)\Phi\Phi F_{CcBbAa}(i-$
$1)+0.125(1-\Phi)\Phi M_{CcBbAa}(i-1)(1-\Phi)F_{CcBbAA}(i-1)+0.125(1-\Phi)\Phi M_{CcBbAa}(i-1)\Phi F_{CcBbAA}(i-1)+$
$0.0625(1-\Phi)\Phi M_{CcBbAa}(i-1)(1-\Phi)F_{CcBBAa}(i-1)+0.125(1-\Phi)\Phi M_{CcBbAa}(i-1)\Phi F_{CcBBAa}(i-1)+$
$0.125(1-\Phi)\Phi M_{CcBbAa}(i-1)F_{CcBBAA}(i-1)+0.25\Phi\Phi M_{CcBbAa}(i-1)F_{ccbbAa}(i-1)+0.5\Phi\Phi M_{CcBbAa}(i-$
$1)F_{ccbbAA}(i-1)+0.125\Phi\Phi M_{CcBbAa}(i-1)(1-\Phi)F_{ccBbAa}(i-1)+0.25\Phi\Phi M_{CcBbAa}(i-1)\Phi F_{ccBbAa}(i-$
$1)+0.25\Phi\Phi M_{CcBbAa}(i-1)F_{ccBbAA}(i-1)+0.125\Phi\Phi M_{CcBbAa}(i-1)F_{CcbbAa}(i-1)+0.25\Phi\Phi M_{CcBbAa}(i-$
$1)F_{CcbbAA}(i-1)+0.0625\Phi\Phi M_{CcBbAa}(i-1)(1-\Phi)(1-\Phi)F_{CcBbAa}(i-1)+0.125\Phi\Phi M_{CcBbAa}(i-1)(1-$
$\Phi)\Phi F_{CcBbAa}(i-1)+0.125\Phi\Phi M_{CcBbAa}(i-1)(1-\Phi)F_{CcBbAA}(i-1)+0.125(1-\Phi)M_{CcBbAA}(i-1)F_{ccbbAa}(i-$
$1)+0.25(1-\Phi)M_{CcBbAA}(i-1)F_{ccbbAA}(i-1)+0.125(1-\Phi)M_{CcBbAA}(i-1)(1-\Phi)F_{ccBbAa}(i-1)+0.25(1-$
$\Phi)M_{CcBbAA}(i-1)\Phi F_{ccBbAa}(i-1)+0.25(1-\Phi)M_{CcBbAA}(i-1)F_{ccBbAA}(i-1)+0.125(1-\Phi)M_{CcBbAA}(i-$
$1)(1-\Phi)F_{ccBBAa}(i-1)+0.25(1-\Phi)M_{CcBbAA}(i-1)\Phi F_{ccBBAa}(i-1)+0.25(1-\Phi)M_{CcBbAA}(i-1)F_{ccBBAA}(i-$
$1)+0.0625(1-\Phi)M_{CcBbAA}(i-1)F_{CcbbAa}(i-1)+0.125(1-\Phi)M_{CcBbAA}(i-1)F_{CcbbAA}(i-1)+0.0625(1-$
$\Phi)M_{CcBbAA}(i-1)(1-\Phi)(1-\Phi)F_{CcBbAa}(i-1)+0.0625(1-\Phi)M_{CcBbAA}(i-1)\Phi(1-\Phi)F_{CcBbAa}(i-$
$1)+0.125(1-\Phi)M_{CcBbAA}(i-1)(1-\Phi)\Phi F_{CcBbAa}(i-1)+0.125(1-\Phi)M_{CcBbAA}(i-1)\Phi\Phi F_{CcBbAa}(i-$
$1)+0.125(1-\Phi)M_{CcBbAA}(i-1)(1-\Phi)F_{CcBbAA}(i-1)+0.125(1-\Phi)M_{CcBbAA}(i-1)\Phi F_{CcBbAA}(i-1)+$
$0.0625(1-\Phi)M_{CcBbAA}(i-1)(1-\Phi)F_{CcBBAa}(i-1)+0.125(1-\Phi)M_{CcBbAA}(i-1)\Phi F_{CcBBAa}(i-1)+$
$0.125(1-\Phi)M_{CcBbAA}(i-1)F_{CcBBAA}(i-1)+0.25\Phi M_{CcBbAA}(i-1)F_{ccbbAa}(i-1)+0.5\Phi M_{CcBbAA}(i-$
$1)F_{ccbbAA}(i-1)+0.125\Phi M_{CcBbAA}(i-1)(1-\Phi)F_{ccBbAa}(i-1)+0.25\Phi M_{CcBbAA}(i-1)\Phi F_{ccBbAa}(i-$
$1)+0.25\Phi M_{CcBbAA}(i-1)F_{ccBbAA}(i-1)+0.125\Phi M_{CcBbAA}(i-1)F_{CcbbAa}(i-1)+0.25\Phi M_{CcBbAA}(i-$
$1)F_{CcbbAA}(i-1)+0.0625\Phi M_{CcBbAA}(i-1)(1-\Phi)(1-\Phi)F_{CcBbAa}(i-1)+0.125\Phi M_{CcBbAA}(i-1)(1-$
$\Phi)\Phi F_{CcBbAa}(i-1)+0.125\Phi M_{CcBbAA}(i-1)(1-\Phi)F_{CcBbAA}(i-1)+0.125(1-\Phi)M_{CcBBAa}(i-1)F_{ccbbAa}(i-$
$1)+0.25(1-\Phi)M_{CcBBAa}(i-1)F_{ccbbAA}(i-1)+0.0625(1-\Phi)M_{CcBBAa}(i-1)(1-\Phi)F_{ccBbAa}(i-1)+$
$0.125(1-\Phi)M_{CcBBAa}(i-1)\Phi F_{ccBbAa}(i-1)+0.125(1-\Phi)M_{CcBBAa}(i-1)F_{ccBbAA}(i-1)+0.0625(1-$
$\Phi)M_{CcBBAa}(i-1)F_{CcbbAa}(i-1)+0.125(1-\Phi)M_{CcBBAa}(i-1)F_{CcbbAA}(i-1)+0.03125(1-\Phi)M_{CcBBAa}(i-$
$1)(1-\Phi)(1-\Phi)F_{CcBbAa}(i-1)+0.0625(1-\Phi)M_{CcBBAa}(i-1)(1-\Phi)\Phi F_{CcBbAa}(i-1)+0.0625(1-$
$\Phi)M_{CcBBAa}(i-1)(1-\Phi)F_{CcBbAA}(i-1)+0.25\Phi M_{CcBBAa}(i-1)F_{ccbbAa}(i-1)+0.5\Phi M_{CcBBAa}(i-$
$1)F_{ccbbAA}(i-1)+0.125\Phi M_{CcBBAa}(i-1)(1-\Phi)F_{ccBbAa}(i-1)+0.25\Phi M_{CcBBAa}(i-1)\Phi F_{ccBbAa}(i-$
$1)+0.25\Phi M_{CcBBAa}(i-1)F_{ccBbAA}(i-1)+0.125\Phi M_{CcBBAa}(i-1)F_{CcbbAa}(i-1)+0.25\Phi M_{CcBBAa}(i-$

$1)F_{CcbbAA}(i-1) + 0.0625\Phi M_{CcBBAa}(i-1)(1-\Phi)(1-\Phi)F_{CcBbAa}(i-1) + 0.125\Phi M_{CcBBAa}(i-1)(1-$
$\Phi)\Phi F_{CcBbAa}(i-1) + 0.125\Phi M_{CcBBAa}(i-1)(1-\Phi)F_{CcBbAA}(i-1) + 0.25M_{CcBBAA}(i-1)F_{ccbbAa}(i-$
$1) + 0.5M_{CcBBAA}(i-1)F_{ccbbAA}(i-1) + 0.125M_{CcBBAA}(i-1)(1-\Phi)F_{ccBbAa}(i-1) + 0.25M_{CcBBAA}(i-$
$1)\Phi F_{ccBbAa}(i-1)+0.25M_{CcBBAA}(i-1)F_{ccBbAA}(i-1)+0.125M_{CcBBAA}(i-1)F_{CcbbAa}(i-1)+0.25M_{CcBBAA}(i-$
$1)F_{CcbbAA}(i-1) + 0.0625M_{CcBBAA}(i-1)(1-\Phi)(1-\Phi)F_{CcBbAa}(i-1) + 0.125M_{CcBBAA}(i-1)(1-$
$\Phi)\Phi F_{CcBbAa}(i-1) + 0.125M_{CcBBAA}(i-1)(1-\Phi)F_{CcBbAA}(i-1)],$

$\mathrm{M}^e_{ccBBaa} = \left(\frac{\bar{\Omega}_{ccBBaa}}{2}\right)[0.25M_{ccBbaa}(i-1)F_{ccBbaa}(i-1)+0.125M_{ccBbaa}(i-1)(1-\Phi)F_{ccBbAa}(i-1)+$
$0.5M_{ccBbaa}(i-1)F_{ccBBaa}(i-1)+0.25M_{ccBbaa}(i-1)(1-\Phi)F_{ccBBAa}(i-1)+0.125M_{ccBbaa}(i-1)(1-$
$\Phi)F_{CcBbaa}(i-1)+0.25M_{ccBbaa}(i-1)\Phi F_{CcBbaa}(i-1)+0.0625M_{ccBbaa}(i-1)(1-\Phi)(1-\Phi)F_{CcBbAa}(i-1)+$
$0.125M_{ccBbaa}(i-1)\Phi(1-\Phi)F_{CcBbAa}(i-1)+0.25M_{ccBbaa}(i-1)F_{CcBBaa}(i-1)+0.125M_{ccBbaa}(i-1)(1-$
$\Phi)F_{CcBBAa}(i-1)+0.125(1-\Phi)M_{ccBbAa}(i-1)F_{ccBbaa}(i-1)+0.0625(1-\Phi)M_{ccBbAa}(i-1)(1-\Phi)F_{ccBbAa}(i-$
$1)+0.25(1-\Phi)M_{ccBbAa}(i-1)F_{ccBBaa}(i-1)+0.125(1-\Phi)M_{ccBbAa}(i-1)(1-\Phi)F_{ccBBAa}(i-1)+$
$0.0625(1-\Phi)M_{ccBbAa}(i-1)(1-\Phi)F_{CcBbaa}(i-1)+0.125(1-\Phi)M_{ccBbAa}(i-1)\Phi F_{CcBbaa}(i-1)+0.03125(1-$
$\Phi)M_{ccBbAa}(i-1)(1-\Phi)(1-\Phi)F_{CcBbAa}(i-1)+0.0625(1-\Phi)M_{ccBbAa}(i-1)\Phi(1-\Phi)F_{CcBbAa}(i-1)+0.125(1-$
$\Phi)M_{ccBbAa}(i-1)F_{CcBBaa}(i-1)+0.0625(1-\Phi)M_{ccBbAa}(i-1)(1-\Phi)F_{CcBBAa}(i-1)+0.5M_{ccBBaa}(i-$
$1)F_{ccBbaa}(i-1)+0.25M_{ccBBaa}(i-1)(1-\Phi)F_{ccBbAa}(i-1)+M_{ccBBaa}(i-1)F_{ccBBaa}(i-1)+0.5M_{ccBBaa}(i-$
$1)(1-\Phi)F_{ccBBAa}(i-1)+0.25M_{ccBBaa}(i-1)(1-\Phi)F_{CcBbaa}(i-1)+0.5M_{ccBBaa}(i-1)\Phi F_{CcBbaa}(i-$
$1)+0.125M_{ccBBaa}(i-1)(1-\Phi)(1-\Phi)F_{CcBbAa}(i-1)+0.25M_{ccBBaa}(i-1)\Phi(1-\Phi)F_{CcBbAa}(i-1)+$
$0.5M_{ccBBaa}(i-1)F_{CcBBaa}(i-1)+0.25M_{ccBBaa}(i-1)(1-\Phi)F_{CcBBAa}(i-1)+0.25(1-\Phi)M_{ccBBAa}(i-$
$1)F_{ccBbaa}(i-1)+0.125(1-\Phi)M_{ccBBAa}(i-1)(1-\Phi)F_{ccBbAa}(i-1)+0.5(1-\Phi)M_{ccBBAa}(i-1)F_{ccBBaa}(i-$
$1)+0.25(1-\Phi)M_{ccBBAa}(i-1)(1-\Phi)F_{ccBBAa}(i-1)+0.125(1-\Phi)M_{ccBBAa}(i-1)(1-\Phi)F_{CcBbaa}(i-$
$1)+0.25(1-\Phi)M_{ccBBAa}(i-1)\Phi F_{CcBbaa}(i-1)+0.0625(1-\Phi)M_{ccBBAa}(i-1)(1-\Phi)(1-\Phi)F_{CcBbAa}(i-$
$1)+0.125(1-\Phi)M_{ccBBAa}(i-1)\Phi(1-\Phi)F_{CcBbAa}(i-1)+0.25(1-\Phi)M_{ccBBAa}(i-1)F_{CcBBaa}(i-1)+$
$0.125(1-\Phi)M_{ccBBAa}(i-1)(1-\Phi)F_{CcBBAa}(i-1)+0.125(1-\Phi)M_{CcBbaa}(i-1)F_{ccBbaa}(i-1)+0.0625(1-$
$\Phi)M_{CcBbaa}(i-1)(1-\Phi)F_{ccBbAa}(i-1)+0.25(1-\Phi)M_{CcBbaa}(i-1)F_{ccBBaa}(i-1)+0.125(1-\Phi)M_{CcBbaa}(i-$
$1)(1-\Phi)F_{ccBBAa}(i-1)+0.0625(1-\Phi)M_{CcBbaa}(i-1)(1-\Phi)F_{CcBbaa}(i-1)+0.125(1-\Phi)M_{CcBbaa}(i-$
$1)\Phi F_{CcBbaa}(i-1)+0.03125(1-\Phi)M_{CcBbaa}(i-1)(1-\Phi)(1-\Phi)F_{CcBbAa}(i-1)+0.0625(1-\Phi)M_{CcBbaa}(i-$
$1)\Phi(1-\Phi)F_{CcBbAa}(i-1)+0.125(1-\Phi)M_{CcBbaa}(i-1)F_{CcBBaa}(i-1)+0.0625(1-\Phi)M_{CcBbaa}(i-1)(1-$
$\Phi)F_{CcBBAa}(i-1)+0.25\Phi M_{CcBbaa}(i-1)F_{ccBbaa}(i-1)+0.125\Phi M_{CcBbaa}(i-1)(1-\Phi)F_{ccBbAa}(i-1)+$
$0.5\Phi M_{CcBbaa}(i-1)F_{ccBBaa}(i-1)+0.25\Phi M_{CcBbaa}(i-1)(1-\Phi)F_{ccBBAa}(i-1)+0.125\Phi M_{CcBbaa}(i-$
$1)(1-\Phi)F_{CcBbaa}(i-1)+0.25\Phi M_{CcBbaa}(i-1)\Phi F_{CcBbaa}(i-1)+0.0625\Phi M_{CcBbaa}(i-1)(1-\Phi)(1-$
$\Phi)F_{CcBbAa}(i-1)+0.125\Phi M_{CcBbaa}(i-1)\Phi(1-\Phi)F_{CcBbAa}(i-1)+0.25\Phi M_{CcBbaa}(i-1)F_{CcBBaa}(i-$
$1)+0.125\Phi M_{CcBbaa}(i-1)(1-\Phi)F_{CcBBAa}(i-1)+0.0625(1-\Phi)(1-\Phi)M_{CcBbAa}(i-1)F_{ccBbaa}(i-1)+$
$0.03125(1-\Phi)(1-\Phi)M_{CcBbAa}(i-1)(1-\Phi)F_{ccBbAa}(i-1)+0.125(1-\Phi)(1-\Phi)M_{CcBbAa}(i-1)F_{ccBBaa}(i-$
$1)+0.0625(1-\Phi)(1-\Phi)M_{CcBbAa}(i-1)(1-\Phi)F_{ccBBAa}(i-1)+0.03125(1-\Phi)(1-\Phi)M_{CcBbAa}(i-1)(1-$
$\Phi)F_{CcBbaa}(i-1)+0.0625(1-\Phi)(1-\Phi)M_{CcBbAa}(i-1)\Phi F_{CcBbaa}(i-1)+0.015625(1-\Phi)(1-\Phi)M_{CcBbAa}(i-$
$1)(1-\Phi)(1-\Phi)F_{CcBbAa}(i-1)+0.03125(1-\Phi)(1-\Phi)M_{CcBbAa}(i-1)\Phi(1-\Phi)F_{CcBbAa}(i-1)+0.0625(1-$
$\Phi)(1-\Phi)M_{CcBbAa}(i-1)F_{CcBBaa}(i-1)+0.03125(1-\Phi)(1-\Phi)M_{CcBbAa}(i-1)(1-\Phi)F_{CcBBAa}(i-1)+$
$0.125\Phi(1-\Phi)M_{CcBbAa}(i-1)F_{ccBbaa}(i-1)+0.0625\Phi(1-\Phi)M_{CcBbAa}(i-1)(1-\Phi)F_{ccBbAa}(i-1)+0.25\Phi(1-$
$\Phi)M_{CcBbAa}(i-1)F_{ccBBaa}(i-1)+0.125\Phi(1-\Phi)M_{CcBbAa}(i-1)(1-\Phi)F_{ccBBAa}(i-1)+0.0625\Phi(1-$
$\Phi)M_{CcBbAa}(i-1)(1-\Phi)F_{CcBbaa}(i-1)+0.125\Phi(1-\Phi)M_{CcBbAa}(i-1)\Phi F_{CcBbaa}(i-1)+0.03125\Phi(1-$
$\Phi)M_{CcBbAa}(i-1)(1-\Phi)(1-\Phi)F_{CcBbAa}(i-1)+0.0625\Phi(1-\Phi)M_{CcBbAa}(i-1)\Phi(1-\Phi)F_{CcBbAa}(i-$
$1)+0.125\Phi(1-\Phi)M_{CcBbAa}(i-1)F_{CcBBaa}(i-1)+0.0625\Phi(1-\Phi)M_{CcBbAa}(i-1)(1-\Phi)F_{CcBBAa}(i-$
$1)+0.25M_{CcBBaa}(i-1)F_{ccBbaa}(i-1)+0.125M_{CcBBaa}(i-1)(1-\Phi)F_{ccBbAa}(i-1)+0.5M_{CcBBaa}(i-$
$1)F_{ccBBaa}(i-1)+0.25M_{CcBBaa}(i-1)(1-\Phi)F_{ccBBAa}(i-1)+0.125M_{CcBBaa}(i-1)(1-\Phi)F_{CcBbaa}(i-1)+$
$0.25M_{CcBBaa}(i-1)\Phi F_{CcBbaa}(i-1)+0.0625M_{CcBBaa}(i-1)(1-\Phi)(1-\Phi)F_{CcBbAa}(i-1)+0.125M_{CcBBaa}(i-$
$1)\Phi(1-\Phi)F_{CcBbAa}(i-1)+0.25M_{CcBBaa}(i-1)F_{CcBBaa}(i-1)+0.125M_{CcBBaa}(i-1)(1-\Phi)F_{CcBBAa}(i-$
$1)+0.125(1-\Phi)M_{CcBBAa}(i-1)F_{ccBbaa}(i-1)+0.0625(1-\Phi)M_{CcBBAa}(i-1)(1-\Phi)F_{ccBbAa}(i-1)+$
$0.25(1-\Phi)M_{CcBBAa}(i-1)F_{ccBBaa}(i-1)+0.125(1-\Phi)M_{CcBBAa}(i-1)(1-\Phi)F_{ccBBAa}(i-1)+0.0625(1-$
$\Phi)M_{CcBBAa}(i-1)(1-\Phi)F_{CcBbaa}(i-1)+0.125(1-\Phi)M_{CcBBAa}(i-1)\Phi F_{CcBbaa}(i-1)+0.03125(1-$
$\Phi)M_{CcBBAa}(i-1)(1-\Phi)(1-\Phi)F_{CcBbAa}(i-1)+0.0625(1-\Phi)M_{CcBBAa}(i-1)\Phi(1-\Phi)F_{CcBbAa}(i-1)+$
$0.125(1-\Phi)M_{CcBBAa}(i-1)F_{CcBBaa}(i-1)+0.0625(1-\Phi)M_{CcBBAa}(i-1)(1-\Phi)F_{CcBBAa}(i-1)],$

$\mathrm{M}^e_{ccBBAa} = \left(\frac{\bar{\Omega}_{ccBBAa}}{2}\right)[0.125M_{ccBbaa}(i-1)(1-\Phi)F_{ccBbAa}(i-1)+0.25M_{ccBbaa}(i-1)\Phi F_{ccBbAa}(i-1)+$
$0.25M_{ccBbaa}(i-1)F_{ccBbAA}(i-1)+0.25M_{ccBbaa}(i-1)(1-\Phi)F_{ccBBAa}(i-1)+0.5M_{ccBbaa}(i-1)\Phi F_{ccBBAa}(i-$
$1)+0.5M_{ccBbaa}(i-1)F_{ccBBAA}(i-1)+0.0625M_{ccBbaa}(i-1)(1-\Phi)(1-\Phi)F_{CcBbAa}(i-1)+0.125M_{ccBbaa}(i-$

$1)\Phi(1-\Phi)F_{CcBbAa}(i-1)+0.125M_{ccBbaa}(i-1)(1-\Phi)\Phi F_{CcBbAa}(i-1)+0.25M_{ccBbaa}(i-1)\Phi\Phi F_{CcBbAa}(i-$
$1)+0.125M_{ccBbaa}(i-1)(1-\Phi)F_{CcBbAA}(i-1)+0.25M_{ccBbaa}(i-1)\Phi F_{CcBbAA}(i-1)+0.125M_{ccBbaa}(i-$
$1)(1-\Phi)F_{CcBBAa}(i-1)+0.25M_{ccBbaa}(i-1)\Phi F_{CcBBAa}(i-1)+0.25M_{ccBbaa}(i-1)F_{CcBBAA}(i-1)+$
$0.125(1-\Phi)M_{ccBbAa}(i-1)F_{ccBbaa}(i-1)+0.125(1-\Phi)M_{ccBbAa}(i-1)(1-\Phi)F_{ccBbAa}(i-1)+0.125(1-$
$\Phi)M_{ccBbAa}(i-1)\Phi F_{ccBbAa}(i-1)+0.125(1-\Phi)M_{ccBbAa}(i-1)F_{ccBbAA}(i-1)+0.25(1-\Phi)M_{ccBbAa}(i-$
$1)F_{ccBBaa}(i-1)+0.25(1-\Phi)M_{ccBbAa}(i-1)(1-\Phi)F_{ccBBAa}(i-1)+0.25(1-\Phi)M_{ccBbAa}(i-1)\Phi F_{ccBBAa}(i-$
$1)+0.25(1-\Phi)M_{ccBbAa}(i-1)F_{ccBBAA}(i-1)+0.0625(1-\Phi)M_{ccBbAa}(i-1)(1-\Phi)F_{CcBbaa}(i-1)+0.125(1-$
$\Phi)M_{ccBbAa}(i-1)\Phi F_{CcBbaa}(i-1)+0.0625(1-\Phi)M_{ccBbAa}(i-1)(1-\Phi)(1-\Phi)F_{CcBbAa}(i-1)+0.125(1-$
$\Phi)M_{ccBbAa}(i-1)\Phi(1-\Phi)F_{CcBbAa}(i-1)+0.0625(1-\Phi)M_{ccBbAa}(i-1)(1-\Phi)\Phi F_{CcBbAa}(i-1)+0.125(1-$
$\Phi)M_{ccBbAa}(i-1)\Phi\Phi F_{CcBbAa}(i-1)+0.0625(1-\Phi)M_{ccBbAa}(i-1)(1-\Phi)F_{CcBbAA}(i-1)+0.125(1-$
$\Phi)M_{ccBbAa}(i-1)\Phi F_{CcBbAA}(i-1)+0.125(1-\Phi)M_{ccBbAa}(i-1)F_{CcBBaa}(i-1)+0.125(1-\Phi)M_{ccBbAa}(i-$
$1)(1-\Phi)F_{CcBBAa}(i-1)+0.125(1-\Phi)M_{ccBbAa}(i-1)\Phi F_{CcBBAa}(i-1)+0.125(1-\Phi)M_{ccBbAa}(i-$
$1)F_{CcBBAA}(i-1)+0.25\Phi M_{ccBbAa}(i-1)F_{ccBbaa}(i-1)+0.125\Phi M_{ccBbAa}(i-1)(1-\Phi)F_{ccBbAa}(i-1)+$
$0.5\Phi M_{ccBbAa}(i-1)F_{ccBBaa}(i-1)+0.25\Phi M_{ccBbAa}(i-1)(1-\Phi)F_{ccBBAa}(i-1)+0.125\Phi M_{ccBbAa}(i-1)(1-$
$\Phi)F_{CcBbaa}(i-1)+0.25\Phi M_{ccBbAa}(i-1)\Phi F_{CcBbaa}(i-1)+0.0625\Phi M_{ccBbAa}(i-1)(1-\Phi)(1-\Phi)F_{CcBbAa}(i-$
$1)+0.125\Phi M_{ccBbAa}(i-1)\Phi(1-\Phi)F_{CcBbAa}(i-1)+0.25\Phi M_{ccBbAa}(i-1)F_{CcBBaa}(i-1)+0.125\Phi M_{ccBbAa}(i-$
$1)(1-\Phi)F_{CcBBAa}(i-1)+0.25M_{ccBbAA}(i-1)F_{ccBbaa}(i-1)+0.125M_{ccBbAA}(i-1)(1-\Phi)F_{ccBbAa}(i-1)+$
$0.5M_{ccBbAA}(i-1)F_{ccBBaa}(i-1)+0.25M_{ccBbAA}(i-1)(1-\Phi)F_{ccBBAa}(i-1)+0.125M_{ccBbAA}(i-1)(1-$
$\Phi)F_{CcBbaa}(i-1)+0.25M_{ccBbAA}(i-1)\Phi F_{CcBbaa}(i-1)+0.0625M_{ccBbAA}(i-1)(1-\Phi)(1-\Phi)F_{CcBbAa}(i-$
$1)+0.125M_{ccBbAA}(i-1)\Phi(1-\Phi)F_{CcBbAa}(i-1)+0.25M_{ccBbAA}(i-1)F_{CcBBaa}(i-1)+0.125M_{ccBbAA}(i-$
$1)(1-\Phi)F_{CcBBAa}(i-1)+0.25M_{ccBBaa}(i-1)(1-\Phi)F_{ccBbAa}(i-1)+0.5M_{ccBBaa}(i-1)\Phi F_{ccBbAa}(i-1)+$
$0.5M_{ccBBaa}(i-1)F_{ccBbAA}(i-1)+0.5M_{ccBBaa}(i-1)(1-\Phi)F_{ccBBAa}(i-1)+M_{ccBBaa}(i-1)\Phi F_{ccBBAa}(i-$
$1)+M_{ccBBaa}(i-1)F_{ccBBAA}(i-1)+0.125M_{ccBBaa}(i-1)(1-\Phi)(1-\Phi)F_{CcBbAa}(i-1)+0.25M_{ccBBaa}(i-$
$1)\Phi(1-\Phi)F_{CcBbAa}(i-1)+0.25M_{ccBBaa}(i-1)(1-\Phi)\Phi F_{CcBbAa}(i-1)+0.5M_{ccBBaa}(i-1)\Phi\Phi F_{CcBbAa}(i-$
$1)+0.25M_{ccBBaa}(i-1)(1-\Phi)F_{CcBbAA}(i-1)+0.5M_{ccBBaa}(i-1)\Phi F_{CcBbAA}(i-1)+0.25M_{ccBBaa}(i-$
$1)(1-\Phi)F_{CcBBAa}(i-1)+0.5M_{ccBBaa}(i-1)\Phi F_{CcBBAa}(i-1)+0.5M_{ccBBaa}(i-1)F_{CcBBAA}(i-1)+$
$0.25(1-\Phi)M_{ccBBAa}(i-1)F_{ccBbaa}(i-1)+0.25(1-\Phi)M_{ccBBAa}(i-1)(1-\Phi)F_{ccBbAa}(i-1)+0.25(1-$
$\Phi)M_{ccBBAa}(i-1)\Phi F_{ccBbAa}(i-1)+0.25(1-\Phi)M_{ccBBAa}(i-1)F_{ccBbAA}(i-1)+0.5(1-\Phi)M_{ccBBAa}(i-$
$1)F_{ccBBaa}(i-1)+0.5(1-\Phi)M_{ccBBAa}(i-1)(1-\Phi)F_{ccBBAa}(i-1)+0.5(1-\Phi)M_{ccBBAa}(i-1)\Phi F_{ccBBAa}(i-$
$1)+0.5(1-\Phi)M_{ccBBAa}(i-1)F_{ccBBAA}(i-1)+0.125(1-\Phi)M_{ccBBAa}(i-1)(1-\Phi)F_{CcBbaa}(i-1)+0.25(1-$
$\Phi)M_{ccBBAa}(i-1)\Phi F_{CcBbaa}(i-1)+0.125(1-\Phi)M_{ccBBAa}(i-1)(1-\Phi)(1-\Phi)F_{CcBbAa}(i-1)+0.25(1-$
$\Phi)M_{ccBBAa}(i-1)\Phi(1-\Phi)F_{CcBbAa}(i-1)+0.125(1-\Phi)M_{ccBBAa}(i-1)(1-\Phi)\Phi F_{CcBbAa}(i-1)+0.25(1-$
$\Phi)M_{ccBBAa}(i-1)\Phi\Phi F_{CcBbAa}(i-1)+0.125(1-\Phi)M_{ccBBAa}(i-1)(1-\Phi)F_{CcBbAA}(i-1)+0.25(1-$
$\Phi)M_{ccBBAa}(i-1)\Phi F_{CcBbAA}(i-1)+0.25(1-\Phi)M_{ccBBAa}(i-1)F_{CcBBaa}(i-1)+0.25(1-\Phi)M_{ccBBAa}(i-$
$1)(1-\Phi)F_{CcBBAa}(i-1)+0.25(1-\Phi)M_{ccBBAa}(i-1)\Phi F_{CcBBAa}(i-1)+0.25(1-\Phi)M_{ccBBAa}(i-$
$1)F_{CcBBAA}(i-1)+0.5\Phi M_{ccBBAa}(i-1)F_{ccBbaa}(i-1)+0.25\Phi M_{ccBBAa}(i-1)(1-\Phi)F_{ccBbAa}(i-1)+$
$\Phi M_{ccBBAa}(i-1)F_{ccBBaa}(i-1)+0.5\Phi M_{ccBBAa}(i-1)(1-\Phi)F_{ccBBAa}(i-1)+0.25\Phi M_{ccBBAa}(i-1)(1-$
$\Phi)F_{CcBbaa}(i-1)+0.5\Phi M_{ccBBAa}(i-1)\Phi F_{CcBbaa}(i-1)+0.125\Phi M_{ccBBAa}(i-1)(1-\Phi)(1-\Phi)F_{CcBbAa}(i-$
$1)+0.25\Phi M_{ccBBAa}(i-1)\Phi(1-\Phi)F_{CcBbAa}(i-1)+0.5\Phi M_{ccBBAa}(i-1)F_{CcBBaa}(i-1)+0.25\Phi M_{ccBBAa}(i-$
$1)(1-\Phi)F_{CcBBAa}(i-1)+0.5M_{ccBBAA}(i-1)F_{ccBbaa}(i-1)+0.25M_{ccBBAA}(i-1)(1-\Phi)F_{ccBbAa}(i-$
$1)+M_{ccBBAA}(i-1)F_{ccBBaa}(i-1)+0.5M_{ccBBAA}(i-1)(1-\Phi)F_{ccBBAa}(i-1)+0.25M_{ccBBAA}(i-1)(1-$
$\Phi)F_{CcBbaa}(i-1)+0.5M_{ccBBAA}(i-1)\Phi F_{CcBbaa}(i-1)+0.125M_{ccBBAA}(i-1)(1-\Phi)(1-\Phi)F_{CcBbAa}(i-$
$1)+0.25M_{ccBBAA}(i-1)\Phi(1-\Phi)F_{CcBbAa}(i-1)+0.5M_{ccBBAA}(i-1)F_{CcBBaa}(i-1)+0.25M_{ccBBAA}(i-$
$1)(1-\Phi)F_{CcBBAa}(i-1)+0.0625(1-\Phi)M_{CcBbaa}(i-1)(1-\Phi)F_{ccBbAa}(i-1)+0.125(1-\Phi)M_{CcBbaa}(i-$
$1)\Phi F_{ccBbAa}(i-1)+0.125(1-\Phi)M_{CcBbaa}(i-1)F_{ccBbAA}(i-1)+0.125(1-\Phi)M_{CcBbaa}(i-1)(1-\Phi)F_{ccBBAa}(i-$
$1)+0.25(1-\Phi)M_{CcBbaa}(i-1)\Phi F_{ccBBAa}(i-1)+0.25(1-\Phi)M_{CcBbaa}(i-1)F_{ccBBAA}(i-1)+0.03125(1-$
$\Phi)M_{CcBbaa}(i-1)(1-\Phi)(1-\Phi)F_{CcBbAa}(i-1)+0.0625(1-\Phi)M_{CcBbaa}(i-1)\Phi(1-\Phi)F_{CcBbAa}(i-$
$1)+0.0625(1-\Phi)M_{CcBbaa}(i-1)(1-\Phi)\Phi F_{CcBbAa}(i-1)+0.125(1-\Phi)M_{CcBbaa}(i-1)\Phi\Phi F_{CcBbAa}(i-$
$1)+0.0625(1-\Phi)M_{CcBbaa}(i-1)(1-\Phi)F_{CcBbAA}(i-1)+0.125(1-\Phi)M_{CcBbaa}(i-1)\Phi F_{CcBbAA}(i-$
$1)+0.0625(1-\Phi)M_{CcBbaa}(i-1)(1-\Phi)F_{CcBBAa}(i-1)+0.125(1-\Phi)M_{CcBbaa}(i-1)\Phi F_{CcBBAa}(i-1)+$
$0.125(1-\Phi)M_{CcBbaa}(i-1)F_{CcBBAA}(i-1)+0.125\Phi M_{CcBbaa}(i-1)(1-\Phi)F_{ccBbAa}(i-1)+0.25\Phi M_{CcBbaa}(i-$
$1)\Phi F_{ccBbAa}(i-1)+0.25\Phi M_{CcBbaa}(i-1)F_{ccBbAA}(i-1)+0.25\Phi M_{CcBbaa}(i-1)(1-\Phi)F_{ccBBAa}(i-1)+$
$0.5\Phi M_{CcBbaa}(i-1)\Phi F_{ccBBAa}(i-1)+0.5\Phi M_{CcBbaa}(i-1)F_{ccBBAA}(i-1)+0.0625\Phi M_{CcBbaa}(i-1)(1-$
$\Phi)(1-\Phi)F_{CcBbAa}(i-1)+0.125\Phi M_{CcBbaa}(i-1)\Phi(1-\Phi)F_{CcBbAa}(i-1)+0.125\Phi M_{CcBbaa}(i-1)(1-$
$\Phi)\Phi F_{CcBbAa}(i-1)+0.25\Phi M_{CcBbaa}(i-1)\Phi\Phi F_{CcBbAa}(i-1)+0.125\Phi M_{CcBbaa}(i-1)(1-\Phi)F_{CcBbAA}(i-1)+$
$0.25\Phi M_{CcBbaa}(i-1)\Phi F_{CcBbAA}(i-1)+0.125\Phi M_{CcBbaa}(i-1)(1-\Phi)F_{CcBBAa}(i-1)+0.25\Phi M_{CcBbaa}(i-$
$1)\Phi F_{CcBBAa}(i-1)+0.25\Phi M_{CcBbaa}(i-1)F_{CcBBAA}(i-1)+0.0625(1-\Phi)(1-\Phi)M_{CcBbAa}(i-1)F_{ccBbaa}(i-$

$1)+0.0625(1-\Phi)(1-\Phi)M_{CcBbAa}(i-1)(1-\Phi)F_{ccBbAa}(i-1)+0.0625(1-\Phi)(1-\Phi)M_{CcBbAa}(i-$
$1)\Phi F_{ccBbAa}(i-1)+0.0625(1-\Phi)(1-\Phi)M_{CcBbAa}(i-1)F_{ccBbAA}(i-1)+0.125(1-\Phi)(1-\Phi)M_{CcBbAa}(i-$
$1)F_{ccBBaa}(i-1)+0.125(1-\Phi)(1-\Phi)M_{CcBbAa}(i-1)(1-\Phi)F_{ccBBAa}(i-1)+0.125(1-\Phi)(1-\Phi)M_{CcBbAa}(i-$
$1)\Phi F_{ccBBAa}(i-1)+0.125(1-\Phi)(1-\Phi)M_{CcBbAa}(i-1)F_{ccBBAA}(i-1)+0.03125(1-\Phi)(1-\Phi)M_{CcBbAa}(i-$
$1)(1-\Phi)F_{CcBbaa}(i-1)+0.0625(1-\Phi)(1-\Phi)M_{CcBbAa}(i-1)\Phi F_{CcBbaa}(i-1)+0.03125(1-\Phi)(1-$
$\Phi)M_{CcBbAa}(i-1)(1-\Phi)(1-\Phi)F_{CcBbAa}(i-1)+0.0625(1-\Phi)(1-\Phi)M_{CcBbAa}(i-1)\Phi(1-\Phi)F_{CcBbAa}(i-$
$1)+0.03125(1-\Phi)(1-\Phi)M_{CcBbAa}(i-1)(1-\Phi)\Phi F_{CcBbAa}(i-1)+0.0625(1-\Phi)(1-\Phi)M_{CcBbAa}(i-$
$1)\Phi\Phi F_{CcBbAa}(i-1)+0.03125(1-\Phi)(1-\Phi)M_{CcBbAa}(i-1)(1-\Phi)F_{CcBbAA}(i-1)+0.0625(1-\Phi)(1-$
$\Phi)M_{CcBbAa}(i-1)\Phi F_{CcBbAA}(i-1)+0.0625(1-\Phi)(1-\Phi)M_{CcBbAa}(i-1)F_{CcBBaa}(i-1)+0.0625(1-$
$\Phi)(1-\Phi)M_{CcBbAa}(i-1)(1-\Phi)F_{CcBBAa}(i-1)+0.0625(1-\Phi)(1-\Phi)M_{CcBbAa}(i-1)\Phi F_{CcBBAa}(i-$
$1)+0.0625(1-\Phi)(1-\Phi)M_{CcBbAa}(i-1)F_{CcBBAA}(i-1)+0.125\Phi(1-\Phi)M_{CcBbAa}(i-1)F_{ccBbaa}(i-1)+$
$0.125\Phi(1-\Phi)M_{CcBbAa}(i-1)(1-\Phi)F_{ccBbAa}(i-1)+0.125\Phi(1-\Phi)M_{CcBbAa}(i-1)\Phi F_{ccBbAa}(i-1)+$
$0.125\Phi(1-\Phi)M_{CcBbAa}(i-1)F_{ccBbAA}(i-1)+0.25\Phi(1-\Phi)M_{CcBbAa}(i-1)F_{ccBBaa}(i-1)+0.25\Phi(1-$
$\Phi)M_{CcBbAa}(i-1)(1-\Phi)F_{ccBBAa}(i-1)+0.25\Phi(1-\Phi)M_{CcBbAa}(i-1)\Phi F_{ccBBAa}(i-1)+0.25\Phi(1-$
$\Phi)M_{CcBbAa}(i-1)F_{ccBBAA}(i-1)+0.0625\Phi(1-\Phi)M_{CcBbAa}(i-1)(1-\Phi)F_{CcBbaa}(i-1)+0.125\Phi(1-$
$\Phi)M_{CcBbAa}(i-1)\Phi F_{CcBbaa}(i-1)+0.0625\Phi(1-\Phi)M_{CcBbAa}(i-1)(1-\Phi)(1-\Phi)F_{CcBbAa}(i-1)+0.125\Phi(1-$
$\Phi)M_{CcBbAa}(i-1)\Phi(1-\Phi)F_{CcBbAa}(i-1)+0.0625\Phi(1-\Phi)M_{CcBbAa}(i-1)(1-\Phi)\Phi F_{CcBbAa}(i-1)+$
$0.125\Phi(1-\Phi)M_{CcBbAa}(i-1)\Phi\Phi F_{CcBbAa}(i-1)+0.0625\Phi(1-\Phi)M_{CcBbAa}(i-1)(1-\Phi)F_{CcBbAA}(i-1)+$
$0.125\Phi(1-\Phi)M_{CcBbAa}(i-1)\Phi F_{CcBbAA}(i-1)+0.125\Phi(1-\Phi)M_{CcBbAa}(i-1)F_{CcBBaa}(i-1)+0.125\Phi(1-$
$\Phi)M_{CcBbAa}(i-1)(1-\Phi)F_{CcBBAa}(i-1)+0.125\Phi(1-\Phi)M_{CcBbAa}(i-1)\Phi F_{CcBBAa}(i-1)+0.125\Phi(1-$
$\Phi)M_{CcBbAa}(i-1)F_{CcBBAA}(i-1)+0.125(1-\Phi)\Phi M_{CcBbAa}(i-1)F_{ccBbaa}(i-1)+0.0625(1-\Phi)\Phi M_{CcBbAa}(i-$
$1)(1-\Phi)F_{ccBbAa}(i-1)+0.25(1-\Phi)\Phi M_{CcBbAa}(i-1)F_{ccBBaa}(i-1)+0.125(1-\Phi)\Phi M_{CcBbAa}(i-1)(1-$
$\Phi)F_{ccBBAa}(i-1)+0.0625(1-\Phi)\Phi M_{CcBbAa}(i-1)(1-\Phi)F_{CcBbaa}(i-1)+0.125(1-\Phi)\Phi M_{CcBbAa}(i-$
$1)\Phi F_{CcBbaa}(i-1)+0.03125(1-\Phi)\Phi M_{CcBbAa}(i-1)(1-\Phi)(1-\Phi)F_{CcBbAa}(i-1)+0.0625(1-\Phi)\Phi M_{CcBbAa}(i-$
$1)\Phi(1-\Phi)F_{CcBbAa}(i-1)+0.125(1-\Phi)\Phi M_{CcBbAa}(i-1)F_{CcBBaa}(i-1)+0.0625(1-\Phi)\Phi M_{CcBbAa}(i-1)(1-$
$\Phi)F_{CcBBAa}(i-1)+0.25\Phi\Phi M_{CcBbAa}(i-1)F_{ccBbaa}(i-1)+0.125\Phi\Phi M_{CcBbAa}(i-1)(1-\Phi)F_{ccBbAa}(i-1)+$
$0.5\Phi\Phi M_{CcBbAa}(i-1)F_{ccBBaa}(i-1)+0.25\Phi\Phi M_{CcBbAa}(i-1)(1-\Phi)F_{ccBBAa}(i-1)+0.125\Phi\Phi M_{CcBbAa}(i-$
$1)(1-\Phi)F_{CcBbaa}(i-1)+0.25\Phi\Phi M_{CcBbAa}(i-1)\Phi F_{CcBbaa}(i-1)+0.0625\Phi\Phi M_{CcBbAa}(i-1)(1-\Phi)(1-$
$\Phi)F_{CcBbAa}(i-1)+0.125\Phi\Phi M_{CcBbAa}(i-1)\Phi(1-\Phi)F_{CcBbAa}(i-1)+0.25\Phi\Phi M_{CcBbAa}(i-1)F_{CcBBaa}(i-$
$1)+0.125\Phi\Phi M_{CcBbAa}(i-1)(1-\Phi)F_{CcBBAa}(i-1)+0.125(1-\Phi)M_{CcBbAA}(i-1)F_{ccBbaa}(i-1)+0.0625(1-$
$\Phi)M_{CcBbAA}(i-1)(1-\Phi)F_{ccBbAa}(i-1)+0.25(1-\Phi)M_{CcBbAA}(i-1)F_{ccBBaa}(i-1)+0.125(1-\Phi)M_{CcBbAA}(i-$
$1)(1-\Phi)F_{ccBBAa}(i-1)+0.0625(1-\Phi)M_{CcBbAA}(i-1)(1-\Phi)F_{CcBbaa}(i-1)+0.125(1-\Phi)M_{CcBbAA}(i-$
$1)\Phi F_{CcBbaa}(i-1)+0.03125(1-\Phi)M_{CcBbAA}(i-1)(1-\Phi)(1-\Phi)F_{CcBbAa}(i-1)+0.0625(1-\Phi)M_{CcBbAA}(i-$
$1)\Phi(1-\Phi)F_{CcBbAa}(i-1)+0.125(1-\Phi)M_{CcBbAA}(i-1)F_{CcBBaa}(i-1)+0.0625(1-\Phi)M_{CcBbAA}(i-1)(1-$
$\Phi)F_{CcBBAa}(i-1)+0.25\Phi M_{CcBbAA}(i-1)F_{ccBbaa}(i-1)+0.125\Phi M_{CcBbAA}(i-1)(1-\Phi)F_{ccBbAa}(i-1)+$
$0.5\Phi M_{CcBbAA}(i-1)F_{ccBBaa}(i-1)+0.25\Phi M_{CcBbAA}(i-1)(1-\Phi)F_{ccBBAa}(i-1)+0.125\Phi M_{CcBbAA}(i-$
$1)(1-\Phi)F_{CcBbaa}(i-1)+0.25\Phi M_{CcBbAA}(i-1)\Phi F_{CcBbaa}(i-1)+0.0625\Phi M_{CcBbAA}(i-1)(1-\Phi)(1-$
$\Phi)F_{CcBbAa}(i-1)+0.125\Phi M_{CcBbAA}(i-1)\Phi(1-\Phi)F_{CcBbAa}(i-1)+0.25\Phi M_{CcBbAA}(i-1)F_{CcBBaa}(i-1)+$
$0.125\Phi M_{CcBbAA}(i-1)(1-\Phi)F_{CcBBAa}(i-1)+0.125M_{CcBBaa}(i-1)(1-\Phi)F_{ccBbAa}(i-1)+0.25M_{CcBBaa}(i-$
$1)\Phi F_{ccBbAa}(i-1)+0.25M_{CcBBaa}(i-1)F_{ccBbAA}(i-1)+0.25M_{CcBBaa}(i-1)(1-\Phi)F_{ccBBAa}(i-1)+$
$0.5M_{CcBBaa}(i-1)\Phi F_{ccBBAa}(i-1)+0.5M_{CcBBaa}(i-1)F_{ccBBAA}(i-1)+0.0625M_{CcBBaa}(i-1)(1-$
$\Phi)(1-\Phi)F_{CcBbAa}(i-1)+0.125M_{CcBBaa}(i-1)\Phi(1-\Phi)F_{CcBbAa}(i-1)+0.125M_{CcBBaa}(i-1)(1-$
$\Phi)\Phi F_{CcBbAa}(i-1)+0.25M_{CcBBaa}(i-1)\Phi\Phi F_{CcBbAa}(i-1)+0.125M_{CcBBaa}(i-1)(1-\Phi)F_{CcBbAA}(i-1)+$
$0.25M_{CcBBaa}(i-1)\Phi F_{CcBbAA}(i-1)+0.125M_{CcBBaa}(i-1)(1-\Phi)F_{CcBBAa}(i-1)+0.25M_{CcBBaa}(i-$
$1)\Phi F_{CcBBAa}(i-1)+0.25M_{CcBBaa}(i-1)F_{CcBBAA}(i-1)+0.125(1-\Phi)M_{CcBBAa}(i-1)F_{ccBbaa}(i-1)+$
$0.125(1-\Phi)M_{CcBBAa}(i-1)(1-\Phi)F_{ccBbAa}(i-1)+0.125(1-\Phi)M_{CcBBAa}(i-1)\Phi F_{ccBbAa}(i-1)+0.125(1-$
$\Phi)M_{CcBBAa}(i-1)F_{ccBbAA}(i-1)+0.25(1-\Phi)M_{CcBBAa}(i-1)F_{ccBBaa}(i-1)+0.25(1-\Phi)M_{CcBBAa}(i-1)(1-$
$\Phi)F_{ccBBAa}(i-1)+0.25(1-\Phi)M_{CcBBAa}(i-1)\Phi F_{ccBBAa}(i-1)+0.25(1-\Phi)M_{CcBBAa}(i-1)F_{ccBBAA}(i-$
$1)+0.0625(1-\Phi)M_{CcBBAa}(i-1)(1-\Phi)F_{CcBbaa}(i-1)+0.125(1-\Phi)M_{CcBBAa}(i-1)\Phi F_{CcBbaa}(i-1)+$
$0.0625(1-\Phi)M_{CcBBAa}(i-1)(1-\Phi)(1-\Phi)F_{CcBbAa}(i-1)+0.125(1-\Phi)M_{CcBBAa}(i-1)\Phi(1-\Phi)F_{CcBbAa}(i-$
$1)+0.0625(1-\Phi)M_{CcBBAa}(i-1)(1-\Phi)\Phi F_{CcBbAa}(i-1)+0.125(1-\Phi)M_{CcBBAa}(i-1)\Phi\Phi F_{CcBbAa}(i-$
$1)+0.0625(1-\Phi)M_{CcBBAa}(i-1)(1-\Phi)F_{CcBbAA}(i-1)+0.125(1-\Phi)M_{CcBBAa}(i-1)\Phi F_{CcBbAA}(i-$
$1)+0.125(1-\Phi)M_{CcBBAa}(i-1)F_{CcBBaa}(i-1)+0.125(1-\Phi)M_{CcBBAa}(i-1)(1-\Phi)F_{CcBBAa}(i-$
$1)+0.125(1-\Phi)M_{CcBBAa}(i-1)\Phi F_{CcBBAa}(i-1)+0.125(1-\Phi)M_{CcBBAa}(i-1)F_{CcBBAA}(i-1)+$
$0.25\Phi M_{CcBBAa}(i-1)F_{ccBbaa}(i-1)+0.125\Phi M_{CcBBAa}(i-1)(1-\Phi)F_{ccBbAa}(i-1)+0.5\Phi M_{CcBBAa}(i-$
$1)F_{ccBBaa}(i-1)+0.25\Phi M_{CcBBAa}(i-1)(1-\Phi)F_{ccBBAa}(i-1)+0.125\Phi M_{CcBBAa}(i-1)(1-\Phi)F_{CcBbaa}(i-$
$1)+0.25\Phi M_{CcBBAa}(i-1)\Phi F_{CcBbaa}(i-1)+0.0625\Phi M_{CcBBAa}(i-1)(1-\Phi)(1-\Phi)F_{CcBbAa}(i-1)+$

$0.125\Phi M_{CcBBAa}(i-1)\Phi(1-\Phi)F_{CcBbAa}(i-1)+0.25\Phi M_{CcBBAa}(i-1)F_{CcBBaa}(i-1)+0.125\Phi M_{CcBBAa}(i-$
$1)(1-\Phi)F_{CcBBAa}(i-1)+0.25M_{CcBBAA}(i-1)F_{ccBbaa}(i-1)+0.125M_{CcBBAA}(i-1)(1-\Phi)F_{ccBbAa}(i-$
$1)+0.5M_{CcBBAA}(i-1)F_{ccBBaa}(i-1)+0.25M_{CcBBAA}(i-1)(1-\Phi)F_{ccBBAa}(i-1)+0.125M_{CcBBAA}(i-$
$1)(1-\Phi)F_{CcBbaa}(i-1)+0.25M_{CcBBAA}(i-1)\Phi F_{CcBbaa}(i-1)+0.0625M_{CcBBAA}(i-1)(1-\Phi)(1-$
$\Phi)F_{CcBbAa}(i-1)+0.125M_{CcBBAA}(i-1)\Phi(1-\Phi)F_{CcBbAa}(i-1)+0.25M_{CcBBAA}(i-1)F_{CcBBaa}(i-1)+$
$0.125M_{CcBBAA}(i-1)(1-\Phi)F_{CcBBAa}(i-1)],$

$\mathrm{M}^e_{ccBBAA}=\left(\frac{\bar{\Omega}_{ccBBAA}}{2}\right)[0.0625(1-\Phi)M_{ccBbAa}(i-1)(1-\Phi)F_{ccBbAa}(i-1)+0.125(1-\Phi)M_{ccBbAa}(i-$
$1)\Phi F_{ccBbAa}(i-1)+0.125(1-\Phi)M_{ccBbAa}(i-1)F_{ccBbAA}(i-1)+0.125(1-\Phi)M_{ccBbAa}(i-1)(1-\Phi)F_{ccBBAa}(i-$
$1)+0.25(1-\Phi)M_{ccBbAa}(i-1)\Phi F_{ccBBAa}(i-1)+0.25(1-\Phi)M_{ccBbAa}(i-1)F_{ccBBAA}(i-1)+0.03125(1-$
$\Phi)M_{ccBbAa}(i-1)(1-\Phi)(1-\Phi)F_{CcBbAa}(i-1)+0.0625(1-\Phi)M_{ccBbAa}(i-1)\Phi(1-\Phi)F_{CcBbAa}(i-1)+$
$0.0625(1-\Phi)M_{ccBbAa}(i-1)(1-\Phi)\Phi F_{CcBbAa}(i-1)+0.125(1-\Phi)M_{ccBbAa}(i-1)\Phi\Phi F_{CcBbAa}(i-1)+$
$0.0625(1-\Phi)M_{ccBbAa}(i-1)(1-\Phi)F_{CcBbAA}(i-1)+0.125(1-\Phi)M_{ccBbAa}(i-1)\Phi F_{CcBbAA}(i-1)+$
$0.0625(1-\Phi)M_{ccBbAa}(i-1)(1-\Phi)F_{CcBBAa}(i-1)+0.125(1-\Phi)M_{ccBbAa}(i-1)\Phi F_{CcBBAa}(i-1)+$
$0.125(1-\Phi)M_{ccBbAa}(i-1)F_{CcBBAA}(i-1)+0.125\Phi M_{ccBbAa}(i-1)(1-\Phi)F_{ccBbAa}(i-1)+0.25\Phi M_{ccBbAa}(i-$
$1)\Phi F_{ccBbAa}(i-1)+0.25\Phi M_{ccBbAa}(i-1)F_{ccBbAA}(i-1)+0.25\Phi M_{ccBbAa}(i-1)(1-\Phi)F_{ccBBAa}(i-1)+$
$0.5\Phi M_{ccBbAa}(i-1)\Phi F_{ccBBAa}(i-1)+0.5\Phi M_{ccBbAa}(i-1)F_{ccBBAA}(i-1)+0.0625\Phi M_{ccBbAa}(i-1)(1-\Phi)(1-$
$\Phi)F_{CcBbAa}(i-1)+0.125\Phi M_{ccBbAa}(i-1)\Phi(1-\Phi)F_{CcBbAa}(i-1)+0.125\Phi M_{ccBbAa}(i-1)(1-\Phi)\Phi F_{CcBbAa}(i-$
$1)+0.25\Phi M_{ccBbAa}(i-1)\Phi\Phi F_{CcBbAa}(i-1)+0.125\Phi M_{ccBbAa}(i-1)(1-\Phi)F_{CcBbAA}(i-1)+0.25\Phi M_{ccBbAa}(i-$
$1)\Phi F_{CcBbAA}(i-1)+0.125\Phi M_{ccBbAa}(i-1)(1-\Phi)F_{CcBBAa}(i-1)+0.25\Phi M_{ccBbAa}(i-1)\Phi F_{CcBBAa}(i-$
$1)+0.25\Phi M_{ccBbAa}(i-1)F_{CcBBAA}(i-1)+0.125M_{ccBbAA}(i-1)(1-\Phi)F_{ccBbAa}(i-1)+0.25M_{ccBbAA}(i-$
$1)\Phi F_{ccBbAa}(i-1)+0.25M_{ccBbAA}(i-1)F_{ccBbAA}(i-1)+0.25M_{ccBbAA}(i-1)(1-\Phi)F_{ccBBAa}(i-1)+$
$0.5M_{ccBbAA}(i-1)\Phi F_{ccBBAa}(i-1)+0.5M_{ccBbAA}(i-1)F_{ccBBAA}(i-1)+0.0625M_{ccBbAA}(i-1)(1-\Phi)(1-$
$\Phi)F_{CcBbAa}(i-1)+0.125M_{ccBbAA}(i-1)\Phi(1-\Phi)F_{CcBbAa}(i-1)+0.125M_{ccBbAA}(i-1)(1-\Phi)\Phi F_{CcBbAa}(i-$
$1)+0.25M_{ccBbAA}(i-1)\Phi\Phi F_{CcBbAa}(i-1)+0.125M_{ccBbAA}(i-1)(1-\Phi)F_{CcBbAA}(i-1)+0.25M_{ccBbAA}(i-$
$1)\Phi F_{CcBbAA}(i-1)+0.125M_{ccBbAA}(i-1)(1-\Phi)F_{CcBBAa}(i-1)+0.25M_{ccBbAA}(i-1)\Phi F_{CcBBAa}(i-$
$1)+0.25M_{ccBbAA}(i-1)F_{CcBBAA}(i-1)+0.125(1-\Phi)M_{ccBBAa}(i-1)(1-\Phi)F_{ccBbAa}(i-1)+0.25(1-$
$\Phi)M_{ccBBAa}(i-1)\Phi F_{ccBbAa}(i-1)+0.25(1-\Phi)M_{ccBBAa}(i-1)F_{ccBbAA}(i-1)+0.25(1-\Phi)M_{ccBBAa}(i-1)(1-$
$\Phi)F_{ccBBAa}(i-1)+0.5(1-\Phi)M_{ccBBAa}(i-1)\Phi F_{ccBBAa}(i-1)+0.5(1-\Phi)M_{ccBBAa}(i-1)F_{ccBBAA}(i-1)+$
$0.0625(1-\Phi)M_{ccBBAa}(i-1)(1-\Phi)(1-\Phi)F_{CcBbAa}(i-1)+0.125(1-\Phi)M_{ccBBAa}(i-1)\Phi(1-\Phi)F_{CcBbAa}(i-$
$1)+0.125(1-\Phi)M_{ccBBAa}(i-1)(1-\Phi)\Phi F_{CcBbAa}(i-1)+0.25(1-\Phi)M_{ccBBAa}(i-1)\Phi\Phi F_{CcBbAa}(i-$
$1)+0.125(1-\Phi)M_{ccBBAa}(i-1)(1-\Phi)F_{CcBbAA}(i-1)+0.25(1-\Phi)M_{ccBBAa}(i-1)\Phi F_{CcBbAA}(i-1)+$
$0.125(1-\Phi)M_{ccBBAa}(i-1)(1-\Phi)F_{CcBBAa}(i-1)+0.25(1-\Phi)M_{ccBBAa}(i-1)\Phi F_{CcBBAa}(i-1)+$
$0.25(1-\Phi)M_{ccBBAa}(i-1)F_{CcBBAA}(i-1)+0.25\Phi M_{ccBBAa}(i-1)(1-\Phi)F_{ccBbAa}(i-1)+0.5\Phi M_{ccBBAa}(i-$
$1)\Phi F_{ccBbAa}(i-1)+0.5\Phi M_{ccBBAa}(i-1)F_{ccBbAA}(i-1)+0.5\Phi M_{ccBBAa}(i-1)(1-\Phi)F_{ccBBAa}(i-1)+$
$\Phi M_{ccBBAa}(i-1)\Phi F_{ccBBAa}(i-1)+\Phi M_{ccBBAa}(i-1)F_{ccBBAA}(i-1)+0.125\Phi M_{ccBBAa}(i-1)(1-\Phi)(1-$
$\Phi)F_{CcBbAa}(i-1)+0.25\Phi M_{ccBBAa}(i-1)\Phi(1-\Phi)F_{CcBbAa}(i-1)+0.25\Phi M_{ccBBAa}(i-1)(1-\Phi)\Phi F_{CcBbAa}(i-$
$1)+0.5\Phi M_{ccBBAa}(i-1)\Phi\Phi F_{CcBbAa}(i-1)+0.25\Phi M_{ccBBAa}(i-1)(1-\Phi)F_{CcBbAA}(i-1)+0.5\Phi M_{ccBBAa}(i-$
$1)\Phi F_{CcBbAA}(i-1)+0.25\Phi M_{ccBBAa}(i-1)(1-\Phi)F_{CcBBAa}(i-1)+0.5\Phi M_{ccBBAa}(i-1)\Phi F_{CcBBAa}(i-$
$1)+0.5\Phi M_{ccBBAa}(i-1)F_{CcBBAA}(i-1)+0.25M_{ccBBAA}(i-1)(1-\Phi)F_{ccBbAa}(i-1)+0.5M_{ccBBAA}(i-$
$1)\Phi F_{ccBbAa}(i-1)+0.5M_{ccBBAA}(i-1)F_{ccBbAA}(i-1)+0.5M_{ccBBAA}(i-1)(1-\Phi)F_{ccBBAa}(i-1)+$
$M_{ccBBAA}(i-1)\Phi F_{ccBBAa}(i-1)+M_{ccBBAA}(i-1)F_{ccBBAA}(i-1)+0.125M_{ccBBAA}(i-1)(1-\Phi)(1-$
$\Phi)F_{CcBbAa}(i-1)+0.25M_{ccBBAA}(i-1)\Phi(1-\Phi)F_{CcBbAa}(i-1)+0.25M_{ccBBAA}(i-1)(1-\Phi)\Phi F_{CcBbAa}(i-$
$1)+0.5M_{ccBBAA}(i-1)\Phi\Phi F_{CcBbAa}(i-1)+0.25M_{ccBBAA}(i-1)(1-\Phi)F_{CcBbAA}(i-1)+0.5M_{ccBBAA}(i-$
$1)\Phi F_{CcBbAA}(i-1)+0.25M_{ccBBAA}(i-1)(1-\Phi)F_{CcBBAa}(i-1)+0.5M_{ccBBAA}(i-1)\Phi F_{CcBBAa}(i-1)+$
$0.5M_{ccBBAA}(i-1)F_{CcBBAA}(i-1)+0.03125(1-\Phi)(1-\Phi)M_{CcBbAa}(i-1)(1-\Phi)F_{ccBbAa}(i-1)+0.0625(1-$
$\Phi)(1-\Phi)M_{CcBbAa}(i-1)\Phi F_{ccBbAa}(i-1)+0.0625(1-\Phi)(1-\Phi)M_{CcBbAa}(i-1)F_{ccBbAA}(i-1)+0.0625(1-$
$\Phi)(1-\Phi)M_{CcBbAa}(i-1)(1-\Phi)F_{ccBBAa}(i-1)+0.125(1-\Phi)(1-\Phi)M_{CcBbAa}(i-1)\Phi F_{ccBBAa}(i-1)+0.125(1-$
$\Phi)(1-\Phi)M_{CcBbAa}(i-1)F_{ccBBAA}(i-1)+0.015625(1-\Phi)(1-\Phi)M_{CcBbAa}(i-1)(1-\Phi)(1-\Phi)F_{CcBbAa}(i-$
$1)+0.03125(1-\Phi)(1-\Phi)M_{CcBbAa}(i-1)\Phi(1-\Phi)F_{CcBbAa}(i-1)+0.03125(1-\Phi)(1-\Phi)M_{CcBbAa}(i-$
$1)(1-\Phi)\Phi F_{CcBbAa}(i-1)+0.0625(1-\Phi)(1-\Phi)M_{CcBbAa}(i-1)\Phi\Phi F_{CcBbAa}(i-1)+0.03125(1-\Phi)(1-$
$\Phi)M_{CcBbAa}(i-1)(1-\Phi)F_{CcBbAA}(i-1)+0.0625(1-\Phi)(1-\Phi)M_{CcBbAa}(i-1)\Phi F_{CcBbAA}(i-1)+0.03125(1-$
$\Phi)(1-\Phi)M_{CcBbAa}(i-1)(1-\Phi)F_{CcBBAa}(i-1)+0.0625(1-\Phi)(1-\Phi)M_{CcBbAa}(i-1)\Phi F_{CcBBAa}(i-1)+$
$0.0625(1-\Phi)(1-\Phi)M_{CcBbAa}(i-1)F_{CcBBAA}(i-1)+0.0625\Phi(1-\Phi)M_{CcBbAa}(i-1)(1-\Phi)F_{ccBbAa}(i-1)+$
$0.125\Phi(1-\Phi)M_{CcBbAa}(i-1)\Phi F_{ccBbAa}(i-1)+0.125\Phi(1-\Phi)M_{CcBbAa}(i-1)F_{ccBbAA}(i-1)+0.125\Phi(1-$
$\Phi)M_{CcBbAa}(i-1)(1-\Phi)F_{ccBBAa}(i-1)+0.25\Phi(1-\Phi)M_{CcBbAa}(i-1)\Phi F_{ccBBAa}(i-1)+0.25\Phi(1-$
$\Phi)M_{CcBbAa}(i-1)F_{ccBBAA}(i-1)+0.03125\Phi(1-\Phi)M_{CcBbAa}(i-1)(1-\Phi)(1-\Phi)F_{CcBbAa}(i-1)+$

$0.0625\Phi(1-\Phi)M_{CcBbAa}(i-1)\Phi(1-\Phi)F_{CcBbAa}(i-1)+0.0625\Phi(1-\Phi)M_{CcBbAa}(i-1)(1-\Phi)\Phi F_{CcBbAa}(i-$
$1)+0.125\Phi(1-\Phi)M_{CcBbAa}(i-1)\Phi\Phi F_{CcBbAa}(i-1)+0.0625\Phi(1-\Phi)M_{CcBbAa}(i-1)(1-\Phi)F_{CcBbAA}(i-$
$1)+0.125\Phi(1-\Phi)M_{CcBbAa}(i-1)\Phi F_{CcBbAA}(i-1)+0.0625\Phi(1-\Phi)M_{CcBbAa}(i-1)(1-\Phi)F_{CcBBAa}(i-$
$1)+0.125\Phi(1-\Phi)M_{CcBbAa}(i-1)\Phi F_{CcBBAa}(i-1)+0.125\Phi(1-\Phi)M_{CcBbAa}(i-1)F_{CcBBAA}(i-1)+$
$0.0625(1-\Phi)\Phi M_{CcBbAa}(i-1)(1-\Phi)F_{ccBbAa}(i-1)+0.125(1-\Phi)\Phi M_{CcBbAa}(i-1)\Phi F_{ccBbAa}(i-1)+$
$0.125(1-\Phi)\Phi M_{CcBbAa}(i-1)F_{ccBbAA}(i-1)+0.125(1-\Phi)\Phi M_{CcBbAa}(i-1)(1-\Phi)F_{ccBBAa}(i-1)+$
$0.25(1-\Phi)\Phi M_{CcBbAa}(i-1)\Phi F_{ccBBAa}(i-1)+0.25(1-\Phi)\Phi M_{CcBbAa}(i-1)F_{ccBBAA}(i-1)+0.03125(1-$
$\Phi)\Phi M_{CcBbAa}(i-1)(1-\Phi)(1-\Phi)F_{CcBbAa}(i-1)+0.0625(1-\Phi)\Phi M_{CcBbAa}(i-1)\Phi(1-\Phi)F_{CcBbAa}(i-$
$1)+0.0625(1-\Phi)\Phi M_{CcBbAa}(i-1)(1-\Phi)\Phi F_{CcBbAa}(i-1)+0.125(1-\Phi)\Phi M_{CcBbAa}(i-1)\Phi\Phi F_{CcBbAa}(i-$
$1)+0.0625(1-\Phi)\Phi M_{CcBbAa}(i-1)(1-\Phi)F_{CcBbAA}(i-1)+0.125(1-\Phi)\Phi M_{CcBbAa}(i-1)\Phi F_{CcBbAA}(i-$
$1)+0.0625(1-\Phi)\Phi M_{CcBbAa}(i-1)(1-\Phi)F_{CcBBAa}(i-1)+0.125(1-\Phi)\Phi M_{CcBbAa}(i-1)\Phi F_{CcBBAa}(i-$
$1)+0.125(1-\Phi)\Phi M_{CcBbAa}(i-1)F_{CcBBAA}(i-1)+0.125\Phi\Phi M_{CcBbAa}(i-1)(1-\Phi)F_{ccBbAa}(i-1)+$
$0.25\Phi\Phi M_{CcBbAa}(i-1)\Phi F_{ccBbAa}(i-1)+0.25\Phi\Phi M_{CcBbAa}(i-1)F_{ccBbAA}(i-1)+0.25\Phi\Phi M_{CcBbAa}(i-$
$1)(1-\Phi)F_{ccBBAa}(i-1)+0.5\Phi\Phi M_{CcBbAa}(i-1)\Phi F_{ccBBAa}(i-1)+0.5\Phi\Phi M_{CcBbAa}(i-1)F_{ccBBAA}(i-$
$1)+0.0625\Phi\Phi M_{CcBbAa}(i-1)(1-\Phi)(1-\Phi)F_{CcBbAa}(i-1)+0.125\Phi\Phi M_{CcBbAa}(i-1)\Phi(1-\Phi)F_{CcBbAa}(i-1)+$
$0.125\Phi\Phi M_{CcBbAa}(i-1)(1-\Phi)\Phi F_{CcBbAa}(i-1)+0.25\Phi\Phi M_{CcBbAa}(i-1)\Phi\Phi F_{CcBbAa}(i-1)+0.125\Phi\Phi M_{CcBbAa}(i-$
$1)(1-\Phi)F_{CcBbAA}(i-1)+0.25\Phi\Phi M_{CcBbAa}(i-1)\Phi F_{CcBbAA}(i-1)+0.125\Phi\Phi M_{CcBbAa}(i-1)(1-\Phi)F_{CcBBAa}(i-$
$1)+0.25\Phi\Phi M_{CcBbAa}(i-1)\Phi F_{CcBBAa}(i-1)+0.25\Phi\Phi M_{CcBbAa}(i-1)F_{CcBBAA}(i-1)+0.0625(1-$
$\Phi)M_{CcBbAA}(i-1)(1-\Phi)F_{ccBbAa}(i-1)+0.125(1-\Phi)M_{CcBbAA}(i-1)\Phi F_{ccBbAa}(i-1)+0.125(1-$
$\Phi)M_{CcBbAA}(i-1)F_{ccBbAA}(i-1)+0.125(1-\Phi)M_{CcBbAA}(i-1)(1-\Phi)F_{ccBBAa}(i-1)+0.25(1-\Phi)M_{CcBbAA}(i-$
$1)\Phi F_{ccBBAa}(i-1)+0.25(1-\Phi)M_{CcBbAA}(i-1)F_{ccBBAA}(i-1)+0.03125(1-\Phi)M_{CcBbAA}(i-1)(1-\Phi)(1-$
$\Phi)F_{CcBbAa}(i-1)+0.0625(1-\Phi)M_{CcBbAA}(i-1)\Phi(1-\Phi)F_{CcBbAa}(i-1)+0.0625(1-\Phi)M_{CcBbAA}(i-$
$1)(1-\Phi)\Phi F_{CcBbAa}(i-1)+0.125(1-\Phi)M_{CcBbAA}(i-1)\Phi\Phi F_{CcBbAa}(i-1)+0.0625(1-\Phi)M_{CcBbAA}(i-$
$1)(1-\Phi)F_{CcBbAA}(i-1)+0.125(1-\Phi)M_{CcBbAA}(i-1)\Phi F_{CcBbAA}(i-1)+0.0625(1-\Phi)M_{CcBbAA}(i-$
$1)(1-\Phi)F_{CcBBAa}(i-1)+0.125(1-\Phi)M_{CcBbAA}(i-1)\Phi F_{CcBBAa}(i-1)+0.125(1-\Phi)M_{CcBbAA}(i-$
$1)F_{CcBBAA}(i-1)+0.125\Phi M_{CcBbAA}(i-1)(1-\Phi)F_{ccBbAa}(i-1)+0.25\Phi M_{CcBbAA}(i-1)\Phi F_{ccBbAa}(i-1)+$
$0.25\Phi M_{CcBbAA}(i-1)F_{ccBbAA}(i-1)+0.25\Phi M_{CcBbAA}(i-1)(1-\Phi)F_{ccBBAa}(i-1)+0.5\Phi M_{CcBbAA}(i-$
$1)\Phi F_{ccBBAa}(i-1)+0.5\Phi M_{CcBbAA}(i-1)F_{ccBBAA}(i-1)+0.0625\Phi M_{CcBbAA}(i-1)(1-\Phi)(1-\Phi)F_{CcBbAa}(i-$
$1)+0.125\Phi M_{CcBbAA}(i-1)\Phi(1-\Phi)F_{CcBbAa}(i-1)+0.125\Phi M_{CcBbAA}(i-1)(1-\Phi)\Phi F_{CcBbAa}(i-1)+$
$0.25\Phi M_{CcBbAA}(i-1)\Phi\Phi F_{CcBbAa}(i-1)+0.125\Phi M_{CcBbAA}(i-1)(1-\Phi)F_{CcBbAA}(i-1)+0.25\Phi M_{CcBbAA}(i-$
$1)\Phi F_{CcBbAA}(i-1)+0.125\Phi M_{CcBbAA}(i-1)(1-\Phi)F_{CcBBAa}(i-1)+0.25\Phi M_{CcBbAA}(i-1)\Phi F_{CcBBAa}(i-$
$1)+0.25\Phi M_{CcBbAA}(i-1)F_{CcBBAA}(i-1)+0.0625(1-\Phi)M_{CcBBAa}(i-1)(1-\Phi)F_{ccBbAa}(i-1)+$
$0.125(1-\Phi)M_{CcBBAa}(i-1)\Phi F_{ccBbAa}(i-1)+0.125(1-\Phi)M_{CcBBAa}(i-1)F_{ccBbAA}(i-1)+0.125(1-$
$\Phi)M_{CcBBAa}(i-1)(1-\Phi)F_{ccBBAa}(i-1)+0.25(1-\Phi)M_{CcBBAa}(i-1)\Phi F_{ccBBAa}(i-1)+0.25(1-$
$\Phi)M_{CcBBAa}(i-1)F_{ccBBAA}(i-1)+0.03125(1-\Phi)M_{CcBBAa}(i-1)(1-\Phi)(1-\Phi)F_{CcBbAa}(i-1)+0.0625(1-$
$\Phi)M_{CcBBAa}(i-1)\Phi(1-\Phi)F_{CcBbAa}(i-1)+0.0625(1-\Phi)M_{CcBBAa}(i-1)(1-\Phi)\Phi F_{CcBbAa}(i-1)+0.125(1-$
$\Phi)M_{CcBBAa}(i-1)\Phi\Phi F_{CcBbAa}(i-1)+0.0625(1-\Phi)M_{CcBBAa}(i-1)(1-\Phi)F_{CcBbAA}(i-1)+0.125(1-$
$\Phi)M_{CcBBAa}(i-1)\Phi F_{CcBbAA}(i-1)+0.0625(1-\Phi)M_{CcBBAa}(i-1)(1-\Phi)F_{CcBBAa}(i-1)+0.125(1-$
$\Phi)M_{CcBBAa}(i-1)\Phi F_{CcBBAa}(i-1)+0.125(1-\Phi)M_{CcBBAa}(i-1)F_{CcBBAA}(i-1)+0.125\Phi M_{CcBBAa}(i-$
$1)(1-\Phi)F_{ccBbAa}(i-1)+0.25\Phi M_{CcBBAa}(i-1)\Phi F_{ccBbAa}(i-1)+0.25\Phi M_{CcBBAa}(i-1)F_{ccBbAA}(i-1)+$
$0.25\Phi M_{CcBBAa}(i-1)(1-\Phi)F_{ccBBAa}(i-1)+0.5\Phi M_{CcBBAa}(i-1)\Phi F_{ccBBAa}(i-1)+0.5\Phi M_{CcBBAa}(i-$
$1)F_{ccBBAA}(i-1)+0.0625\Phi M_{CcBBAa}(i-1)(1-\Phi)(1-\Phi)F_{CcBbAa}(i-1)+0.125\Phi M_{CcBBAa}(i-1)\Phi(1-$
$\Phi)F_{CcBbAa}(i-1)+0.125\Phi M_{CcBBAa}(i-1)(1-\Phi)\Phi F_{CcBbAa}(i-1)+0.25\Phi M_{CcBBAa}(i-1)\Phi\Phi F_{CcBbAa}(i-1)+$
$0.125\Phi M_{CcBBAa}(i-1)(1-\Phi)F_{CcBbAA}(i-1)+0.25\Phi M_{CcBBAa}(i-1)\Phi F_{CcBbAA}(i-1)+0.125\Phi M_{CcBBAa}(i-$
$1)(1-\Phi)F_{CcBBAa}(i-1)+0.25\Phi M_{CcBBAa}(i-1)\Phi F_{CcBBAa}(i-1)+0.25\Phi M_{CcBBAa}(i-1)F_{CcBBAA}(i-$
$1)+0.125M_{CcBBAA}(i-1)(1-\Phi)F_{ccBbAa}(i-1)+0.25M_{CcBBAA}(i-1)\Phi F_{ccBbAa}(i-1)+0.25M_{CcBBAA}(i-$
$1)F_{ccBbAA}(i-1)+0.25M_{CcBBAA}(i-1)(1-\Phi)F_{ccBBAa}(i-1)+0.5M_{CcBBAA}(i-1)\Phi F_{ccBBAa}(i-1)+$
$0.5M_{CcBBAA}(i-1)F_{ccBBAA}(i-1)+0.0625M_{CcBBAA}(i-1)(1-\Phi)(1-\Phi)F_{CcBbAa}(i-1)+0.125M_{CcBBAA}(i-$
$1)\Phi(1-\Phi)F_{CcBbAa}(i-1)+0.125M_{CcBBAA}(i-1)(1-\Phi)\Phi F_{CcBbAa}(i-1)+0.25M_{CcBBAA}(i-1)\Phi\Phi F_{CcBbAa}(i-$
$1)+0.125M_{CcBBAA}(i-1)(1-\Phi)F_{CcBbAA}(i-1)+0.25M_{CcBBAA}(i-1)\Phi F_{CcBbAA}(i-1)+0.125M_{CcBBAA}(i-$
$1)(1-\Phi)F_{CcBBAa}(i-1)+0.25M_{CcBBAA}(i-1)\Phi F_{CcBBAa}(i-1)+0.25M_{CcBBAA}(i-1)F_{CcBBAA}(i-1)],$

$\mathrm{M}^{e}_{Ccbbaa}=\left(\frac{\bar{\Omega}_{Ccbbaa}}{2}\right)[0.5M_{ccbbaa}(i-1)F_{Ccbbaa}(i-1)+0.25M_{ccbbaa}(i-1)F_{CcbbAa}(i-1)+0.25M_{ccbbaa}(i-$
$1)(1-\Phi)F_{CcBbaa}(i-1)+0.125M_{ccbbaa}(i-1)(1-\Phi)(1-\Phi)F_{CcBbAa}(i-1)+M_{ccbbaa}(i-1)F_{CCbbaa}(i-1)+$
$0.5M_{ccbbaa}(i-1)F_{CCbbAa}(i-1)+0.5M_{ccbbaa}(i-1)(1-\Phi)F_{CCBbaa}(i-1)+0.25M_{ccbbaa}(i-1)(1-\Phi)(1-$
$\Phi)F_{CCBbAa}(i-1)+0.25M_{ccbbAa}(i-1)F_{Ccbbaa}(i-1)+0.125M_{ccbbAa}(i-1)F_{CcbbAa}(i-1)+0.125M_{ccbbAa}(i-$
$1)(1-\Phi)F_{CcBbaa}(i-1)+0.0625M_{ccbbAa}(i-1)(1-\Phi)(1-\Phi)F_{CcBbAa}(i-1)+0.5M_{ccbbAa}(i-1)F_{CCbbaa}(i-1)+$

$$0.25M_{ccbbAa}(i-1)F_{CCbbAa}(i-1)+0.25M_{ccbbAa}(i-1)(1-\Phi)F_{CCBbaa}(i-1)+0.125M_{ccbbAa}(i-1)(1-\Phi)(1-\Phi)F_{CCBbAa}(i-1)+0.25M_{ccBbaa}(i-1)F_{Ccbbaa}(i-1)+0.125M_{ccBbaa}(i-1)F_{CcbbAa}(i-1)+0.125M_{ccBbaa}(i-1)(1-\Phi)F_{CcBbaa}(i-1)+0.0625M_{ccBbaa}(i-1)(1-\Phi)(1-\Phi)F_{CcBbAa}(i-1)+0.5M_{ccBbaa}(i-1)F_{CCbbaa}(i-1)+$$

$$0.25M_{ccBbaa}(i-1)F_{CCbbAa}(i-1)+0.25M_{ccBbaa}(i-1)(1-\Phi)F_{CCBbaa}(i-1)+0.125M_{ccBbaa}(i-1)(1-\Phi)(1-\Phi)F_{CCBbAa}(i-1)+0.125(1-\Phi)M_{ccBbAa}(i-1)F_{Ccbbaa}(i-1)+0.0625(1-\Phi)M_{ccBbAa}(i-1)F_{CcbbAa}(i-1)+$$

$$0.0625(1-\Phi)M_{ccBbAa}(i-1)(1-\Phi)F_{CcBbaa}(i-1)+0.03125(1-\Phi)M_{ccBbAa}(i-1)(1-\Phi)(1-\Phi)F_{CcBbAa}(i-1)+$$

$$0.25(1-\Phi)M_{ccBbAa}(i-1)F_{CCbbaa}(i-1)+0.125(1-\Phi)M_{ccBbAa}(i-1)F_{CCbbAa}(i-1)+0.125(1-\Phi)M_{ccBbAa}(i-1)(1-\Phi)F_{CCBbaa}(i-1)+0.0625(1-\Phi)M_{ccBbAa}(i-1)(1-\Phi)(1-\Phi)F_{CCBbAa}(i-1)+0.5M_{Ccbbaa}(i-1)F_{ccbbaa}(i-1)+0.25M_{Ccbbaa}(i-1)F_{ccbbAa}(i-1)+0.25M_{Ccbbaa}(i-1)F_{ccBbaa}(i-1)+0.125M_{Ccbbaa}(i-1)(1-\Phi)F_{ccBbAa}(i-1)+0.5M_{Ccbbaa}(i-1)F_{Ccbbaa}(i-1)+0.25M_{Ccbbaa}(i-1)F_{CcbbAa}(i-1)+0.25M_{Ccbbaa}(i-1)(1-\Phi)F_{CcBbaa}(i-1)+0.125M_{Ccbbaa}(i-1)(1-\Phi)(1-\Phi)F_{CcBbAa}(i-1)+0.5M_{Ccbbaa}(i-1)F_{CCbbaa}(i-1)+0.25M_{Ccbbaa}(i-1)F_{CCbbAa}(i-1)+0.25M_{Ccbbaa}(i-1)(1-\Phi)F_{CCBbaa}(i-1)+0.125M_{Ccbbaa}(i-1)(1-\Phi)(1-\Phi)F_{CCBbAa}(i-1)+0.25M_{CcbbAa}(i-1)F_{ccbbaa}(i-1)+0.125M_{CcbbAa}(i-1)F_{ccbbAa}(i-1)+$$

$$0.125M_{CcbbAa}(i-1)F_{ccBbaa}(i-1)+0.0625M_{CcbbAa}(i-1)(1-\Phi)F_{ccBbAa}(i-1)+0.25M_{CcbbAa}(i-1)F_{Ccbbaa}(i-1)+0.125M_{CcbbAa}(i-1)F_{CcbbAa}(i-1)+0.125M_{CcbbAa}(i-1)(1-\Phi)F_{CcBbaa}(i-1)+0.0625M_{CcbbAa}(i-1)(1-\Phi)(1-\Phi)F_{CcBbAa}(i-1)+0.25M_{CcbbAa}(i-1)F_{CCbbaa}(i-1)+0.125M_{CcbbAa}(i-1)F_{CCbbAa}(i-1)+$$

$$0.125M_{CcbbAa}(i-1)(1-\Phi)F_{CCBbaa}(i-1)+0.0625M_{CcbbAa}(i-1)(1-\Phi)(1-\Phi)F_{CCBbAa}(i-1)+0.25(1-\Phi)M_{CcBbaa}(i-1)F_{ccbbaa}(i-1)+0.125(1-\Phi)M_{CcBbaa}(i-1)F_{ccbbAa}(i-1)+0.125(1-\Phi)M_{CcBbaa}(i-1)F_{ccBbaa}(i-1)+0.0625(1-\Phi)M_{CcBbaa}(i-1)(1-\Phi)F_{ccBbAa}(i-1)+0.25(1-\Phi)M_{CcBbaa}(i-1)F_{Ccbbaa}(i-1)+0.125(1-\Phi)M_{CcBbaa}(i-1)F_{CcbbAa}(i-1)+0.125(1-\Phi)M_{CcBbaa}(i-1)(1-\Phi)F_{CcBbaa}(i-1)+$$

$$0.0625(1-\Phi)M_{CcBbaa}(i-1)(1-\Phi)(1-\Phi)F_{CcBbAa}(i-1)+0.25(1-\Phi)M_{CcBbaa}(i-1)F_{CCbbaa}(i-1)+$$

$$0.125(1-\Phi)M_{CcBbaa}(i-1)F_{CCbbAa}(i-1)+0.125(1-\Phi)M_{CcBbaa}(i-1)(1-\Phi)F_{CCBbaa}(i-1)+0.0625(1-\Phi)M_{CcBbaa}(i-1)(1-\Phi)(1-\Phi)F_{CCBbAa}(i-1)+0.125(1-\Phi)(1-\Phi)M_{CcBbAa}(i-1)F_{ccbbaa}(i-1)+0.0625(1-\Phi)(1-\Phi)M_{CcBbAa}(i-1)F_{ccbbAa}(i-1)+0.0625(1-\Phi)(1-\Phi)M_{CcBbAa}(i-1)F_{ccBbaa}(i-1)+0.03125(1-\Phi)(1-\Phi)M_{CcBbAa}(i-1)(1-\Phi)F_{ccBbAa}(i-1)+0.125(1-\Phi)(1-\Phi)M_{CcBbAa}(i-1)F_{Ccbbaa}(i-1)+0.0625(1-\Phi)(1-\Phi)M_{CcBbAa}(i-1)F_{CcbbAa}(i-1)+0.0625(1-\Phi)(1-\Phi)M_{CcBbAa}(i-1)(1-\Phi)F_{CcBbaa}(i-1)+$$

$$0.03125(1-\Phi)(1-\Phi)M_{CcBbAa}(i-1)(1-\Phi)(1-\Phi)F_{CcBbAa}(i-1)+0.125(1-\Phi)(1-\Phi)M_{CcBbAa}(i-1)F_{CCbbaa}(i-1)+0.0625(1-\Phi)(1-\Phi)M_{CcBbAa}(i-1)F_{CCbbAa}(i-1)+0.0625(1-\Phi)(1-\Phi)M_{CcBbAa}(i-1)(1-\Phi)F_{CCBbaa}(i-1)+0.03125(1-\Phi)(1-\Phi)M_{CcBbAa}(i-1)(1-\Phi)(1-\Phi)F_{CCBbAa}(i-1)+M_{CCbbaa}(i-1)F_{ccbbaa}(i-1)+0.5M_{CCbbaa}(i-1)F_{ccbbAa}(i-1)+0.5M_{CCbbaa}(i-1)F_{ccBbaa}(i-1)+0.25M_{CCbbaa}(i-1)(1-\Phi)F_{ccBbAa}(i-1)+0.5M_{CCbbaa}(i-1)F_{Ccbbaa}(i-1)+0.25M_{CCbbaa}(i-1)F_{CcbbAa}(i-1)+0.25M_{CCbbaa}(i-1)(1-\Phi)F_{CcBbaa}(i-1)+0.125M_{CCbbaa}(i-1)(1-\Phi)(1-\Phi)F_{CcBbAa}(i-1)+0.5M_{CCbbAa}(i-1)F_{ccbbaa}(i-1)+0.25M_{CCbbAa}(i-1)F_{ccbbAa}(i-1)+0.25M_{CCbbAa}(i-1)F_{ccBbaa}(i-1)+0.125M_{CCbbAa}(i-1)(1-\Phi)F_{ccBbAa}(i-1)+0.25M_{CCbbAa}(i-1)F_{Ccbbaa}(i-1)+0.125M_{CCbbAa}(i-1)F_{CcbbAa}(i-1)+0.125M_{CCbbAa}(i-1)(1-\Phi)F_{CcBbaa}(i-1)+0.0625M_{CCbbAa}(i-1)(1-\Phi)(1-\Phi)F_{CcBbAa}(i-1)+0.5(1-\Phi)M_{CCBbaa}(i-1)F_{ccbbaa}(i-1)+0.25(1-\Phi)M_{CCBbaa}(i-1)F_{ccbbAa}(i-1)+0.25(1-\Phi)M_{CCBbaa}(i-1)F_{ccBbaa}(i-1)+0.125(1-\Phi)M_{CCBbaa}(i-1)(1-\Phi)F_{ccBbAa}(i-1)+0.25(1-\Phi)M_{CCBbaa}(i-1)F_{Ccbbaa}(i-1)+$$

$$0.125(1-\Phi)M_{CCBbaa}(i-1)F_{CcbbAa}(i-1)+0.125(1-\Phi)M_{CCBbaa}(i-1)(1-\Phi)F_{CcBbaa}(i-1)+0.0625(1-\Phi)M_{CCBbaa}(i-1)(1-\Phi)(1-\Phi)F_{CcBbAa}(i-1)+0.25(1-\Phi)(1-\Phi)M_{CCBbAa}(i-1)F_{ccbbaa}(i-1)+0.125(1-\Phi)(1-\Phi)M_{CCBbAa}(i-1)F_{ccbbAa}(i-1)+0.125(1-\Phi)(1-\Phi)M_{CCBbAa}(i-1)F_{ccBbaa}(i-1)+0.0625(1-\Phi)(1-\Phi)M_{CCBbAa}(i-1)(1-\Phi)F_{ccBbAa}(i-1)+0.125(1-\Phi)(1-\Phi)M_{CCBbAa}(i-1)F_{Ccbbaa}(i-1)+$$

$$0.0625(1-\Phi)(1-\Phi)M_{CCBbAa}(i-1)F_{CcbbAa}(i-1)+0.0625(1-\Phi)(1-\Phi)M_{CCBbAa}(i-1)(1-\Phi)F_{CcBbaa}(i-1)+0.03125(1-\Phi)(1-\Phi)M_{CCBbAa}(i-1)(1-\Phi)(1-\Phi)F_{CcBbAa}(i-1)],$$

$$\mathrm{M}^e_{CcbbAa}=\left(\frac{\bar{\Omega}_{CcbbAa}}{2}\right)[0.25M_{ccbbaa}(i-1)F_{CcbbAa}(i-1)+0.5M_{ccbbaa}(i-1)F_{CcbbAA}(i-1)+0.125M_{ccbbaa}(i-1)(1-\Phi)(1-\Phi)F_{CcBbAa}(i-1)+0.25M_{ccbbaa}(i-1)(1-\Phi)\Phi F_{CcBbAa}(i-1)+0.25M_{ccbbaa}(i-1)(1-\Phi)F_{CcBbAA}(i-1)+0.5M_{ccbbaa}(i-1)F_{CCbbAa}(i-1)+M_{ccbbaa}(i-1)F_{CCbbAA}(i-1)+0.25M_{ccbbaa}(i-1)(1-\Phi)(1-\Phi)F_{CCBbAa}(i-1)+0.5M_{ccbbaa}(i-1)(1-\Phi)F_{CCBbAA}(i-1)+0.25M_{ccbbAa}(i-1)F_{Ccbbaa}(i-1)+$$

$$0.25M_{ccbbAa}(i-1)F_{CcbbAa}(i-1)+0.25M_{ccbbAa}(i-1)F_{CcbbAA}(i-1)+0.125M_{ccbbAa}(i-1)(1-\Phi)F_{CcBbaa}(i-1)+0.125M_{ccbbAa}(i-1)(1-\Phi)(1-\Phi)F_{CcBbAa}(i-1)+0.125M_{ccbbAa}(i-1)(1-\Phi)\Phi F_{CcBbAa}(i-1)+$$

$$0.125M_{ccbbAa}(i-1)(1-\Phi)F_{CcBbAA}(i-1)+0.5M_{ccbbAa}(i-1)F_{CCbbaa}(i-1)+0.5M_{ccbbAa}(i-1)F_{CCbbAa}(i-1)+0.5M_{ccbbAa}(i-1)F_{CCbbAA}(i-1)+0.25M_{ccbbAa}(i-1)(1-\Phi)F_{CCBbaa}(i-1)+0.25M_{ccbbAa}(i-1)(1-\Phi)(1-\Phi)F_{CCBbAa}(i-1)+0.25M_{ccbbAa}(i-1)(1-\Phi)F_{CCBbAA}(i-1)+0.5M_{ccbbAA}(i-1)F_{Ccbbaa}(i-1)+$$

$$0.25M_{ccbbAA}(i-1)F_{CcbbAa}(i-1)+0.25M_{ccbbAA}(i-1)(1-\Phi)F_{CcBbaa}(i-1)+0.125M_{ccbbAA}(i-1)(1-\Phi)(1-\Phi)F_{CcBbAa}(i-1)+M_{ccbbAA}(i-1)F_{CCbbaa}(i-1)+0.5M_{ccbbAA}(i-1)F_{CCbbAa}(i-1)+0.5M_{ccbbAA}(i-1)(1-\Phi)F_{CCBbaa}(i-1)+0.25M_{ccbbAA}(i-1)(1-\Phi)(1-\Phi)F_{CCBbAa}(i-1)+0.125M_{ccBbaa}(i-1)F_{CcbbAa}(i-1)+$$

$$0.25M_{ccBbaa}(i-1)F_{CcbbAA}(i-1)+0.0625M_{ccBbaa}(i-1)(1-\Phi)(1-\Phi)F_{CcBbAa}(i-1)+0.125M_{ccBbaa}(i-$$

$1)(1-\Phi)\Phi F_{CcBbAa}(i-1)+0.125M_{ccBbaa}(i-1)(1-\Phi)F_{CcBbAA}(i-1)+0.25M_{ccBbaa}(i-1)F_{CCbbAa}(i-1)+$
$0.5M_{ccBbaa}(i-1)F_{CCbbAA}(i-1)+0.125M_{ccBbaa}(i-1)(1-\Phi)(1-\Phi)F_{CCBbAa}(i-1)+0.25M_{ccBbaa}(i-1)(1-$
$\Phi)F_{CCBbAA}(i-1)+0.125(1-\Phi)M_{ccBbAa}(i-1)F_{Ccbbaa}(i-1)+0.125(1-\Phi)M_{ccBbAa}(i-1)F_{CcbbAa}(i-1)+$
$0.125(1-\Phi)M_{ccBbAa}(i-1)F_{CcbbAA}(i-1)+0.0625(1-\Phi)M_{ccBbAa}(i-1)(1-\Phi)F_{CcBbaa}(i-1)+0.0625(1-$
$\Phi)M_{ccBbAa}(i-1)(1-\Phi)(1-\Phi)F_{CcBbAa}(i-1)+0.0625(1-\Phi)M_{ccBbAa}(i-1)(1-\Phi)\Phi F_{CcBbAa}(i-1)+$
$0.0625(1-\Phi)M_{ccBbAa}(i-1)(1-\Phi)F_{CcBbAA}(i-1)+0.25(1-\Phi)M_{ccBbAa}(i-1)F_{CCbbaa}(i-1)+0.25(1-$
$\Phi)M_{ccBbAa}(i-1)F_{CCbbAa}(i-1)+0.25(1-\Phi)M_{ccBbAa}(i-1)F_{CCbbAA}(i-1)+0.125(1-\Phi)M_{ccBbAa}(i-1)(1-$
$\Phi)F_{CCBbaa}(i-1)+0.125(1-\Phi)M_{ccBbAa}(i-1)(1-\Phi)(1-\Phi)F_{CCBbAa}(i-1)+0.125(1-\Phi)M_{ccBbAa}(i-$
$1)(1-\Phi)F_{CCBbAA}(i-1)+0.25\Phi M_{ccBbAa}(i-1)F_{Ccbbaa}(i-1)+0.125\Phi M_{ccBbAa}(i-1)F_{CcbbAa}(i-1)+$
$0.125\Phi M_{ccBbAa}(i-1)(1-\Phi)F_{CcBbaa}(i-1)+0.0625\Phi M_{ccBbAa}(i-1)(1-\Phi)(1-\Phi)F_{CcBbAa}(i-1)+$
$0.5\Phi M_{ccBbAa}(i-1)F_{CCbbaa}(i-1)+0.25\Phi M_{ccBbAa}(i-1)F_{CCbbAa}(i-1)+0.25\Phi M_{ccBbAa}(i-1)(1-$
$\Phi)F_{CCBbaa}(i-1)+0.125\Phi M_{ccBbAa}(i-1)(1-\Phi)(1-\Phi)F_{CCBbAa}(i-1)+0.25M_{ccBbAA}(i-1)F_{Ccbbaa}(i-$
$1)+0.125M_{ccBbAA}(i-1)F_{CcbbAa}(i-1)+0.125M_{ccBbAA}(i-1)(1-\Phi)F_{CcBbaa}(i-1)+0.0625M_{ccBbAA}(i-$
$1)(1-\Phi)(1-\Phi)F_{CcBbAa}(i-1)+0.5M_{ccBbAA}(i-1)F_{CCbbaa}(i-1)+0.25M_{ccBbAA}(i-1)F_{CCbbAa}(i-$
$1)+0.25M_{ccBbAA}(i-1)(1-\Phi)F_{CCBbaa}(i-1)+0.125M_{ccBbAA}(i-1)(1-\Phi)(1-\Phi)F_{CCBbAa}(i-1)+$
$0.25M_{Ccbbaa}(i-1)F_{ccbbAa}(i-1)+0.5M_{Ccbbaa}(i-1)F_{ccbbAA}(i-1)+0.125M_{Ccbbaa}(i-1)(1-\Phi)F_{ccBbAa}(i-$
$1)+0.25M_{Ccbbaa}(i-1)\Phi F_{ccBbAa}(i-1)+0.25M_{Ccbbaa}(i-1)F_{ccBbAA}(i-1)+0.25M_{Ccbbaa}(i-1)F_{CcbbAa}(i-$
$1)+0.5M_{Ccbbaa}(i-1)F_{CcbbAA}(i-1)+0.125M_{Ccbbaa}(i-1)(1-\Phi)(1-\Phi)F_{CcBbAa}(i-1)+0.25M_{Ccbbaa}(i-$
$1)(1-\Phi)\Phi F_{CcBbAa}(i-1)+0.25M_{Ccbbaa}(i-1)(1-\Phi)F_{CcBbAA}(i-1)+0.25M_{Ccbbaa}(i-1)F_{CCbbAa}(i-1)+$
$0.5M_{Ccbbaa}(i-1)F_{CCbbAA}(i-1)+0.125M_{Ccbbaa}(i-1)(1-\Phi)(1-\Phi)F_{CCBbAa}(i-1)+0.25M_{Ccbbaa}(i-1)(1-$
$\Phi)F_{CCBbAA}(i-1)+0.25M_{CcbbAa}(i-1)F_{ccbbaa}(i-1)+0.25M_{CcbbAa}(i-1)F_{ccbbAa}(i-1)+0.25M_{CcbbAa}(i-$
$1)F_{ccbbAA}(i-1)+0.125M_{CcbbAa}(i-1)F_{ccBbaa}(i-1)+0.125M_{CcbbAa}(i-1)(1-\Phi)F_{ccBbAa}(i-1)+$
$0.125M_{CcbbAa}(i-1)\Phi F_{ccBbAa}(i-1)+0.125M_{CcbbAa}(i-1)F_{ccBbAA}(i-1)+0.25M_{CcbbAa}(i-1)F_{Ccbbaa}(i-$
$1)+0.25M_{CcbbAa}(i-1)F_{CcbbAa}(i-1)+0.25M_{CcbbAa}(i-1)F_{CcbbAA}(i-1)+0.125M_{CcbbAa}(i-1)(1-$
$\Phi)F_{CcBbaa}(i-1)+0.125M_{CcbbAa}(i-1)(1-\Phi)(1-\Phi)F_{CcBbAa}(i-1)+0.125M_{CcbbAa}(i-1)(1-\Phi)\Phi F_{CcBbAa}(i-$
$1)+0.125M_{CcbbAa}(i-1)(1-\Phi)F_{CcBbAA}(i-1)+0.25M_{CcbbAa}(i-1)F_{CCbbaa}(i-1)+0.25M_{CcbbAa}(i-$
$1)F_{CCbbAa}(i-1)+0.25M_{CcbbAa}(i-1)F_{CCbbAA}(i-1)+0.125M_{CcbbAa}(i-1)(1-\Phi)F_{CCBbaa}(i-1)+$
$0.125M_{CcbbAa}(i-1)(1-\Phi)(1-\Phi)F_{CCBbAa}(i-1)+0.125M_{CcbbAa}(i-1)(1-\Phi)F_{CCBbAA}(i-1)+0.5M_{CcbbAA}(i-$
$1)F_{ccbbaa}(i-1)+0.25M_{CcbbAA}(i-1)F_{ccbbAa}(i-1)+0.25M_{CcbbAA}(i-1)F_{ccBbaa}(i-1)+0.125M_{CcbbAA}(i-$
$1)(1-\Phi)F_{ccBbAa}(i-1)+0.5M_{CcbbAA}(i-1)F_{Ccbbaa}(i-1)+0.25M_{CcbbAA}(i-1)F_{CcbbAa}(i-1)+0.25M_{CcbbAA}(i-$
$1)(1-\Phi)F_{CcBbaa}(i-1)+0.125M_{CcbbAA}(i-1)(1-\Phi)(1-\Phi)F_{CcBbAa}(i-1)+0.5M_{CcbbAA}(i-1)F_{CCbbaa}(i-$
$1)+0.25M_{CcbbAA}(i-1)F_{CCbbAa}(i-1)+0.25M_{CcbbAA}(i-1)(1-\Phi)F_{CCBbaa}(i-1)+0.125M_{CcbbAA}(i-$
$1)(1-\Phi)(1-\Phi)F_{CCBbAa}(i-1)+0.125(1-\Phi)M_{CcBbaa}(i-1)F_{ccbbAa}(i-1)+0.25(1-\Phi)M_{CcBbaa}(i-$
$1)F_{ccbbAA}(i-1)+0.0625(1-\Phi)M_{CcBbaa}(i-1)(1-\Phi)F_{ccBbAa}(i-1)+0.125(1-\Phi)M_{CcBbaa}(i-1)\Phi F_{ccBbAa}(i-$
$1)+0.125(1-\Phi)M_{CcBbaa}(i-1)F_{ccBbAA}(i-1)+0.125(1-\Phi)M_{CcBbaa}(i-1)F_{CcbbAa}(i-1)+0.25(1-$
$\Phi)M_{CcBbaa}(i-1)F_{CcbbAA}(i-1)+0.0625(1-\Phi)M_{CcBbaa}(i-1)(1-\Phi)(1-\Phi)F_{CcBbAa}(i-1)+0.125(1-$
$\Phi)M_{CcBbaa}(i-1)(1-\Phi)\Phi F_{CcBbAa}(i-1)+0.125(1-\Phi)M_{CcBbaa}(i-1)(1-\Phi)F_{CcBbAA}(i-1)+0.125(1-$
$\Phi)M_{CcBbaa}(i-1)F_{CCbbAa}(i-1)+0.25(1-\Phi)M_{CcBbaa}(i-1)F_{CCbbAA}(i-1)+0.0625(1-\Phi)M_{CcBbaa}(i-$
$1)(1-\Phi)(1-\Phi)F_{CCBbAa}(i-1)+0.125(1-\Phi)M_{CcBbaa}(i-1)(1-\Phi)F_{CCBbAA}(i-1)+0.125(1-\Phi)(1-$
$\Phi)M_{CcBbAa}(i-1)F_{ccbbaa}(i-1)+0.125(1-\Phi)(1-\Phi)M_{CcBbAa}(i-1)F_{ccbbAa}(i-1)+0.125(1-\Phi)(1-$
$\Phi)M_{CcBbAa}(i-1)F_{ccbbAA}(i-1)+0.0625(1-\Phi)(1-\Phi)M_{CcBbAa}(i-1)F_{ccBbaa}(i-1)+0.0625(1-\Phi)(1-$
$\Phi)M_{CcBbAa}(i-1)(1-\Phi)F_{ccBbAa}(i-1)+0.0625(1-\Phi)(1-\Phi)M_{CcBbAa}(i-1)\Phi F_{ccBbAa}(i-1)+0.0625(1-$
$\Phi)(1-\Phi)M_{CcBbAa}(i-1)F_{ccBbAA}(i-1)+0.125(1-\Phi)(1-\Phi)M_{CcBbAa}(i-1)F_{Ccbbaa}(i-1)+0.125(1-\Phi)(1-$
$\Phi)M_{CcBbAa}(i-1)F_{CcbbAa}(i-1)+0.125(1-\Phi)(1-\Phi)M_{CcBbAa}(i-1)F_{CcbbAA}(i-1)+0.0625(1-\Phi)(1-$
$\Phi)M_{CcBbAa}(i-1)(1-\Phi)F_{CcBbaa}(i-1)+0.0625(1-\Phi)(1-\Phi)M_{CcBbAa}(i-1)(1-\Phi)(1-\Phi)F_{CcBbAa}(i-$
$1)+0.0625(1-\Phi)(1-\Phi)M_{CcBbAa}(i-1)(1-\Phi)\Phi F_{CcBbAa}(i-1)+0.0625(1-\Phi)(1-\Phi)M_{CcBbAa}(i-1)(1-$
$\Phi)F_{CcBbAA}(i-1)+0.125(1-\Phi)(1-\Phi)M_{CcBbAa}(i-1)F_{CCbbaa}(i-1)+0.125(1-\Phi)(1-\Phi)M_{CcBbAa}(i-$
$1)F_{CCbbAa}(i-1)+0.125(1-\Phi)(1-\Phi)M_{CcBbAa}(i-1)F_{CCbbAA}(i-1)+0.0625(1-\Phi)(1-\Phi)M_{CcBbAa}(i-$
$1)(1-\Phi)F_{CCBbaa}(i-1)+0.0625(1-\Phi)(1-\Phi)M_{CcBbAa}(i-1)(1-\Phi)(1-\Phi)F_{CCBbAa}(i-1)+0.0625(1-$
$\Phi)(1-\Phi)M_{CcBbAa}(i-1)(1-\Phi)F_{CCBbAA}(i-1)+0.25(1-\Phi)\Phi M_{CcBbAa}(i-1)F_{ccbbaa}(i-1)+0.125(1-$
$\Phi)\Phi M_{CcBbAa}(i-1)F_{ccbbAa}(i-1)+0.125(1-\Phi)\Phi M_{CcBbAa}(i-1)F_{ccBbaa}(i-1)+0.0625(1-\Phi)\Phi M_{CcBbAa}(i-$
$1)(1-\Phi)F_{ccBbAa}(i-1)+0.25(1-\Phi)\Phi M_{CcBbAa}(i-1)F_{Ccbbaa}(i-1)+0.125(1-\Phi)\Phi M_{CcBbAa}(i-1)F_{CcbbAa}(i-$
$1)+0.125(1-\Phi)\Phi M_{CcBbAa}(i-1)(1-\Phi)F_{CcBbaa}(i-1)+0.0625(1-\Phi)\Phi M_{CcBbAa}(i-1)(1-\Phi)(1-$
$\Phi)F_{CcBbAa}(i-1)+0.25(1-\Phi)\Phi M_{CcBbAa}(i-1)F_{CCbbaa}(i-1)+0.125(1-\Phi)\Phi M_{CcBbAa}(i-1)F_{CCbbAa}(i-$
$1)+0.125(1-\Phi)\Phi M_{CcBbAa}(i-1)(1-\Phi)F_{CCBbaa}(i-1)+0.0625(1-\Phi)\Phi M_{CcBbAa}(i-1)(1-\Phi)(1-$
$\Phi)F_{CCBbAa}(i-1)+0.25(1-\Phi)M_{CcBbAA}(i-1)F_{ccbbaa}(i-1)+0.125(1-\Phi)M_{CcBbAA}(i-1)F_{ccbbAa}(i-$
$1)+0.125(1-\Phi)M_{CcBbAA}(i-1)F_{ccBbaa}(i-1)+0.0625(1-\Phi)M_{CcBbAA}(i-1)(1-\Phi)F_{ccBbAa}(i-1)+$

$0.25(1-\Phi)M_{CcBbAA}(i-1)F_{Ccbbaa}(i-1)+0.125(1-\Phi)M_{CcBbAA}(i-1)F_{CcbbAa}(i-1)+0.125(1-$
$\Phi)M_{CcBbAA}(i-1)(1-\Phi)F_{CcBbaa}(i-1)+0.0625(1-\Phi)M_{CcBbAA}(i-1)(1-\Phi)(1-\Phi)F_{CcBbAa}(i-1)+$
$0.25(1-\Phi)M_{CcBbAA}(i-1)F_{CCbbaa}(i-1)+0.125(1-\Phi)M_{CcBbAA}(i-1)F_{CCbbAa}(i-1)+0.125(1-$
$\Phi)M_{CcBbAA}(i-1)(1-\Phi)F_{CCBbaa}(i-1)+0.0625(1-\Phi)M_{CcBbAA}(i-1)(1-\Phi)(1-\Phi)F_{CCBbAa}(i-1)+$
$0.5M_{CCbbaa}(i-1)F_{ccbbAa}(i-1)+M_{CCbbaa}(i-1)F_{ccbbAA}(i-1)+0.25M_{CCbbaa}(i-1)(1-\Phi)F_{ccBbAa}(i-1)+$
$0.5M_{CCbbaa}(i-1)\Phi F_{ccBbAa}(i-1)+0.5M_{CCbbaa}(i-1)F_{ccBbAA}(i-1)+0.25M_{CCbbaa}(i-1)F_{CcbbAa}(i-1)+$
$0.5M_{CCbbaa}(i-1)F_{CcbbAA}(i-1)+0.125M_{CCbbaa}(i-1)(1-\Phi)(1-\Phi)F_{CcBbAa}(i-1)+0.25M_{CCbbaa}(i-$
$1)(1-\Phi)\Phi F_{CcBbAa}(i-1)+0.25M_{CCbbaa}(i-1)(1-\Phi)F_{CcBbAA}(i-1)+0.5M_{CCbbAa}(i-1)F_{ccbbaa}(i-$
$1)+0.5M_{CCbbAa}(i-1)F_{ccbbAa}(i-1)+0.5M_{CCbbAa}(i-1)F_{ccbbAA}(i-1)+0.25M_{CCbbAa}(i-1)F_{ccBbaa}(i-$
$1)+0.25M_{CCbbAa}(i-1)(1-\Phi)F_{ccBbAa}(i-1)+0.25M_{CCbbAa}(i-1)\Phi F_{ccBbAa}(i-1)+0.25M_{CCbbAa}(i-$
$1)F_{ccBbAA}(i-1)+0.25M_{CCbbAa}(i-1)F_{Ccbbaa}(i-1)+0.25M_{CCbbAa}(i-1)F_{CcbbAa}(i-1)+0.25M_{CCbbAa}(i-$
$1)F_{CcbbAA}(i-1)+0.125M_{CCbbAa}(i-1)(1-\Phi)F_{CcBbaa}(i-1)+0.125M_{CCbbAa}(i-1)(1-\Phi)(1-\Phi)F_{CcBbAa}(i-$
$1)+0.125M_{CCbbAa}(i-1)(1-\Phi)\Phi F_{CcBbAa}(i-1)+0.125M_{CCbbAa}(i-1)(1-\Phi)F_{CcBbAA}(i-1)+M_{CCbbAA}(i-$
$1)F_{ccbbaa}(i-1)+0.5M_{CCbbAA}(i-1)F_{ccbbAa}(i-1)+0.5M_{CCbbAA}(i-1)F_{ccBbaa}(i-1)+0.25M_{CCbbAA}(i-1)(1-$
$\Phi)F_{ccBbAa}(i-1)+0.5M_{CCbbAA}(i-1)F_{Ccbbaa}(i-1)+0.25M_{CCbbAA}(i-1)F_{CcbbAa}(i-1)+0.25M_{CCbbAA}(i-$
$1)(1-\Phi)F_{CcBbaa}(i-1)+0.125M_{CCbbAA}(i-1)(1-\Phi)(1-\Phi)F_{CcBbAa}(i-1)+0.25(1-\Phi)M_{CCBbaa}(i-$
$1)F_{ccbbAa}(i-1)+0.5(1-\Phi)M_{CCBbaa}(i-1)F_{ccbbAA}(i-1)+0.125(1-\Phi)M_{CCBbaa}(i-1)(1-\Phi)F_{ccBbAa}(i-$
$1)+0.25(1-\Phi)M_{CCBbaa}(i-1)\Phi F_{ccBbAa}(i-1)+0.25(1-\Phi)M_{CCBbaa}(i-1)F_{ccBbAA}(i-1)+0.125(1-$
$\Phi)M_{CCBbaa}(i-1)F_{CcbbAa}(i-1)+0.25(1-\Phi)M_{CCBbaa}(i-1)F_{CcbbAA}(i-1)+0.0625(1-\Phi)M_{CCBbaa}(i-1)(1-$
$\Phi)(1-\Phi)F_{CcBbAa}(i-1)+0.125(1-\Phi)M_{CCBbaa}(i-1)(1-\Phi)\Phi F_{CcBbAa}(i-1)+0.125(1-\Phi)M_{CCBbaa}(i-$
$1)(1-\Phi)F_{CcBbAA}(i-1)+0.25(1-\Phi)(1-\Phi)M_{CCBbAa}(i-1)F_{ccbbaa}(i-1)+0.25(1-\Phi)(1-\Phi)M_{CCBbAa}(i-$
$1)F_{ccbbAa}(i-1)+0.25(1-\Phi)(1-\Phi)M_{CCBbAa}(i-1)F_{ccbbAA}(i-1)+0.125(1-\Phi)(1-\Phi)M_{CCBbAa}(i-$
$1)F_{ccBbaa}(i-1)+0.125(1-\Phi)(1-\Phi)M_{CCBbAa}(i-1)(1-\Phi)F_{ccBbAa}(i-1)+0.125(1-\Phi)(1-\Phi)M_{CCBbAa}(i-$
$1)\Phi F_{ccBbAa}(i-1)+0.125(1-\Phi)(1-\Phi)M_{CCBbAa}(i-1)F_{ccBbAA}(i-1)+0.125(1-\Phi)(1-\Phi)M_{CCBbAa}(i-$
$1)F_{Ccbbaa}(i-1)+0.125(1-\Phi)(1-\Phi)M_{CCBbAa}(i-1)F_{CcbbAa}(i-1)+0.125(1-\Phi)(1-\Phi)M_{CCBbAa}(i-$
$1)F_{CcbbAA}(i-1)+0.0625(1-\Phi)(1-\Phi)M_{CCBbAa}(i-1)(1-\Phi)F_{CcBbaa}(i-1)+0.0625(1-\Phi)(1-$
$\Phi)M_{CCBbAa}(i-1)(1-\Phi)(1-\Phi)F_{CcBbAa}(i-1)+0.0625(1-\Phi)(1-\Phi)M_{CCBbAa}(i-1)(1-\Phi)\Phi F_{CcBbAa}(i-$
$1)+0.0625(1-\Phi)(1-\Phi)M_{CCBbAa}(i-1)(1-\Phi)F_{CcBbAA}(i-1)+0.25(1-\Phi)\Phi M_{CCBbAa}(i-1)F_{ccbbAa}(i-$
$1)+0.25(1-\Phi)\Phi M_{CCBbAa}(i-1)F_{ccBbaa}(i-1)+0.125(1-\Phi)\Phi M_{CCBbAa}(i-1)(1-\Phi)F_{ccBbAa}(i-1)+$
$0.25(1-\Phi)\Phi M_{CCBbAa}(i-1)F_{Ccbbaa}(i-1)+0.125(1-\Phi)\Phi M_{CCBbAa}(i-1)F_{CcbbAa}(i-1)+0.125(1-$
$\Phi)\Phi M_{CCBbAa}(i-1)(1-\Phi)F_{CcBbaa}(i-1)+0.0625(1-\Phi)\Phi M_{CCBbAa}(i-1)(1-\Phi)(1-\Phi)F_{CcBbAa}(i-1)+$
$0.5(1-\Phi)M_{CCBbAA}(i-1)F_{ccbbaa}(i-1)+0.25(1-\Phi)M_{CCBbAA}(i-1)F_{ccbbAa}(i-1)+0.25(1-\Phi)M_{CCBbAA}(i-$
$1)F_{ccBbaa}(i-1)+0.125(1-\Phi)M_{CCBbAA}(i-1)(1-\Phi)F_{ccBbAa}(i-1)+0.25(1-\Phi)M_{CCBbAA}(i-1)F_{Ccbbaa}(i-$
$1)+0.125(1-\Phi)M_{CCBbAA}(i-1)F_{CcbbAa}(i-1)+0.125(1-\Phi)M_{CCBbAA}(i-1)(1-\Phi)F_{CcBbaa}(i-1)+$
$0.0625(1-\Phi)M_{CCBbAA}(i-1)(1-\Phi)(1-\Phi)F_{CcBbAa}(i-1)],$

$\mathrm{M}^{e}_{CcbbAA}=\left(\frac{\bar{\Omega}_{CcbbAA}}{2}\right)[0.125M_{ccbbAa}(i-1)F_{CcbbAa}(i-1)+0.25M_{ccbbAa}(i-1)F_{CcbbAA}(i-1)+0.0625M_{ccbbAa}(i-$
$1)(1-\Phi)(1-\Phi)F_{CcBbAa}(i-1)+0.125M_{ccbbAa}(i-1)(1-\Phi)\Phi F_{CcBbAa}(i-1)+0.125M_{ccbbAa}(i-1)(1-$
$\Phi)F_{CcBbAA}(i-1)+0.25M_{ccbbAa}(i-1)F_{CCbbAa}(i-1)+0.5M_{ccbbAa}(i-1)F_{CCbbAA}(i-1)+0.125M_{ccbbAa}(i-$
$1)(1-\Phi)(1-\Phi)F_{CCBbAa}(i-1)+0.25M_{ccbbAa}(i-1)(1-\Phi)F_{CCBbAA}(i-1)+0.25M_{ccbbAA}(i-1)F_{CcbbAa}(i-$
$1)+0.5M_{ccbbAA}(i-1)F_{CcbbAA}(i-1)+0.125M_{ccbbAA}(i-1)(1-\Phi)(1-\Phi)F_{CcBbAa}(i-1)+0.25M_{ccbbAA}(i-$
$1)(1-\Phi)\Phi F_{CcBbAa}(i-1)+0.25M_{ccbbAA}(i-1)(1-\Phi)F_{CcBbAA}(i-1)+0.5M_{ccbbAA}(i-1)F_{CCbbAa}(i-1)+$
$M_{ccbbAA}(i-1)F_{CCbbAA}(i-1)+0.25M_{ccbbAA}(i-1)(1-\Phi)(1-\Phi)F_{CCBbAa}(i-1)+0.5M_{ccbbAA}(i-1)(1-$
$\Phi)F_{CCBbAA}(i-1)+0.0625(1-\Phi)M_{ccBbAa}(i-1)F_{CcbbAa}(i-1)+0.125(1-\Phi)M_{ccBbAa}(i-1)F_{CcbbAA}(i-1)+$
$0.03125(1-\Phi)M_{ccBbAa}(i-1)(1-\Phi)(1-\Phi)F_{CcBbAa}(i-1)+0.0625(1-\Phi)M_{ccBbAa}(i-1)(1-\Phi)\Phi F_{CcBbAa}(i-$
$1)+0.0625(1-\Phi)M_{ccBbAa}(i-1)(1-\Phi)F_{CcBbAA}(i-1)+0.125(1-\Phi)M_{ccBbAa}(i-1)F_{CCbbAa}(i-1)+$
$0.25(1-\Phi)M_{ccBbAa}(i-1)F_{CCbbAA}(i-1)+0.0625(1-\Phi)M_{ccBbAa}(i-1)(1-\Phi)(1-\Phi)F_{CCBbAa}(i-1)+$
$0.125(1-\Phi)M_{ccBbAa}(i-1)(1-\Phi)F_{CCBbAA}(i-1)+0.125\Phi M_{ccBbAa}(i-1)F_{CcbbAa}(i-1)+0.25\Phi M_{ccBbAa}(i-$
$1)F_{CcbbAA}(i-1)+0.0625\Phi M_{ccBbAa}(i-1)(1-\Phi)(1-\Phi)F_{CcBbAa}(i-1)+0.125\Phi M_{ccBbAa}(i-1)(1-$
$\Phi)\Phi F_{CcBbAa}(i-1)+0.125\Phi M_{ccBbAa}(i-1)(1-\Phi)F_{CcBbAA}(i-1)+0.25\Phi M_{ccBbAa}(i-1)F_{CCbbAa}(i-1)+$
$0.5\Phi M_{ccBbAa}(i-1)F_{CCbbAA}(i-1)+0.125\Phi M_{ccBbAa}(i-1)(1-\Phi)(1-\Phi)F_{CCBbAa}(i-1)+0.25\Phi M_{ccBbAa}(i-$
$1)(1-\Phi)F_{CCBbAA}(i-1)+0.125M_{ccBbAA}(i-1)F_{CcbbAa}(i-1)+0.25M_{ccBbAA}(i-1)F_{CcbbAA}(i-1)+$
$0.0625M_{ccBbAA}(i-1)(1-\Phi)(1-\Phi)F_{CcBbAa}(i-1)+0.125M_{ccBbAA}(i-1)(1-\Phi)\Phi F_{CcBbAa}(i-1)+$
$0.125M_{ccBbAA}(i-1)(1-\Phi)F_{CcBbAA}(i-1)+0.25M_{ccBbAA}(i-1)F_{CCbbAa}(i-1)+0.5M_{ccBbAA}(i-1)F_{CCbbAA}(i-$
$1)+0.125M_{ccBbAA}(i-1)(1-\Phi)(1-\Phi)F_{CCBbAa}(i-1)+0.25M_{ccBbAA}(i-1)(1-\Phi)F_{CCBbAA}(i-1)+$
$0.125M_{CcbbAa}(i-1)F_{ccbbAa}(i-1)+0.25M_{CcbbAa}(i-1)F_{ccbbAA}(i-1)+0.0625M_{CcbbAa}(i-1)(1-\Phi)F_{ccBbAa}(i-$
$1)+0.125M_{CcbbAa}(i-1)\Phi F_{ccBbAa}(i-1)+0.125M_{CcbbAa}(i-1)F_{ccBbAA}(i-1)+0.125M_{CcbbAa}(i-1)F_{CcbbAa}(i-$

$$1)+0.25M_{CcbbAa}(i-1)F_{CcbbAA}(i-1)+0.0625M_{CcbbAa}(i-1)(1-\Phi)(1-\Phi)F_{CcBbAa}(i-1)+0.125M_{CcbbAa}(i-1)(1-\Phi)\Phi F_{CcBbAa}(i-1)+0.125M_{CcbbAa}(i-1)(1-\Phi)F_{CcBbAA}(i-1)+0.125M_{CcbbAa}(i-1)F_{CCbbAa}(i-1)+0.25M_{CcbbAa}(i-1)F_{CCbbAA}(i-1)+0.0625M_{CcbbAa}(i-1)(1-\Phi)(1-\Phi)F_{CCBbAa}(i-1)+0.125M_{CcbbAa}(i-1)(1-\Phi)F_{CCBbAA}(i-1)+0.25M_{CcbbAA}(i-1)F_{ccbbAa}(i-1)+0.5M_{CcbbAA}(i-1)F_{ccbbAA}(i-1)+0.125M_{CcbbAA}(i-1)(1-\Phi)F_{ccBbAa}(i-1)+0.25M_{CcbbAA}(i-1)\Phi F_{ccBbAa}(i-1)+0.25M_{CcbbAA}(i-1)F_{ccBbAA}(i-1)+0.25M_{CcbbAA}(i-1)F_{CcbbAa}(i-1)+0.5M_{CcbbAA}(i-1)F_{CcbbAA}(i-1)+0.125M_{CcbbAA}(i-1)(1-\Phi)(1-\Phi)F_{CcBbAa}(i-1)+0.25M_{CcbbAA}(i-1)(1-\Phi)\Phi F_{CcBbAa}(i-1)+0.25M_{CcbbAA}(i-1)(1-\Phi)F_{CcBbAA}(i-1)+0.25M_{CcbbAA}(i-1)F_{CCbbAa}(i-1)+0.5M_{CcbbAA}(i-1)F_{CCbbAA}(i-1)+0.125M_{CcbbAA}(i-1)(1-\Phi)(1-\Phi)F_{CCBbAa}(i-1)+0.25M_{CcbbAA}(i-1)(1-\Phi)F_{CCBbAA}(i-1)+0.0625(1-\Phi)(1-\Phi)M_{CcBbAa}(i-1)F_{ccbbAa}(i-1)+0.125(1-\Phi)(1-\Phi)M_{CcBbAa}(i-1)F_{ccbbAA}(i-1)+0.03125(1-\Phi)(1-\Phi)M_{CcBbAa}(i-1)(1-\Phi)F_{ccBbAa}(i-1)+0.0625(1-\Phi)(1-\Phi)M_{CcBbAa}(i-1)\Phi F_{ccBbAa}(i-1)+0.0625(1-\Phi)(1-\Phi)M_{CcBbAa}(i-1)F_{ccBbAA}(i-1)+0.0625(1-\Phi)(1-\Phi)M_{CcBbAa}(i-1)F_{CcbbAa}(i-1)+0.125(1-\Phi)(1-\Phi)M_{CcBbAa}(i-1)F_{CcbbAA}(i-1)+0.03125(1-\Phi)(1-\Phi)M_{CcBbAa}(i-1)(1-\Phi)(1-\Phi)F_{CcBbAa}(i-1)+0.0625(1-\Phi)(1-\Phi)M_{CcBbAa}(i-1)(1-\Phi)\Phi F_{CcBbAa}(i-1)+0.0625(1-\Phi)(1-\Phi)M_{CcBbAa}(i-1)(1-\Phi)F_{CcBbAA}(i-1)+0.0625(1-\Phi)(1-\Phi)M_{CcBbAa}(i-1)F_{CCbbAa}(i-1)+0.125(1-\Phi)(1-\Phi)M_{CcBbAa}(i-1)F_{CCbbAA}(i-1)+0.03125(1-\Phi)(1-\Phi)M_{CcBbAa}(i-1)(1-\Phi)(1-\Phi)F_{CCBbAa}(i-1)+0.0625(1-\Phi)(1-\Phi)M_{CcBbAa}(i-1)(1-\Phi)F_{CCBbAA}(i-1)+0.125(1-\Phi)\Phi M_{CcBbAa}(i-1)F_{ccbbAa}(i-1)+0.25(1-\Phi)\Phi M_{CcBbAa}(i-1)F_{ccbbAA}(i-1)+0.0625(1-\Phi)\Phi M_{CcBbAa}(i-1)(1-\Phi)F_{ccBbAa}(i-1)+0.125(1-\Phi)\Phi M_{CcBbAa}(i-1)\Phi F_{ccBbAa}(i-1)+0.125(1-\Phi)\Phi M_{CcBbAa}(i-1)F_{ccBbAA}(i-1)+0.125(1-\Phi)\Phi M_{CcBbAa}(i-1)F_{CcbbAa}(i-1)+0.25(1-\Phi)\Phi M_{CcBbAa}(i-1)F_{CcbbAA}(i-1)+0.0625(1-\Phi)\Phi M_{CcBbAa}(i-1)(1-\Phi)(1-\Phi)F_{CcBbAa}(i-1)+0.125(1-\Phi)\Phi M_{CcBbAa}(i-1)(1-\Phi)\Phi F_{CcBbAa}(i-1)+0.125(1-\Phi)\Phi M_{CcBbAa}(i-1)(1-\Phi)F_{CcBbAA}(i-1)+0.125(1-\Phi)\Phi M_{CcBbAa}(i-1)F_{CCbbAa}(i-1)+0.25(1-\Phi)\Phi M_{CcBbAa}(i-1)F_{CCbbAA}(i-1)+0.0625(1-\Phi)\Phi M_{CcBbAa}(i-1)(1-\Phi)(1-\Phi)F_{CCBbAa}(i-1)+0.125(1-\Phi)\Phi M_{CcBbAa}(i-1)(1-\Phi)F_{CCBbAA}(i-1)+0.125(1-\Phi)M_{CcBbAA}(i-1)F_{ccbbAa}(i-1)+0.25(1-\Phi)M_{CcBbAA}(i-1)F_{ccbbAA}(i-1)+0.0625(1-\Phi)M_{CcBbAA}(i-1)(1-\Phi)F_{ccBbAa}(i-1)+0.125(1-\Phi)M_{CcBbAA}(i-1)\Phi F_{ccBbAa}(i-1)+0.125(1-\Phi)M_{CcBbAA}(i-1)F_{ccBbAA}(i-1)+0.125(1-\Phi)M_{CcBbAA}(i-1)F_{CcbbAa}(i-1)+0.25(1-\Phi)M_{CcBbAA}(i-1)F_{CcbbAA}(i-1)+0.0625(1-\Phi)M_{CcBbAA}(i-1)(1-\Phi)(1-\Phi)F_{CcBbAa}(i-1)+0.125(1-\Phi)M_{CcBbAA}(i-1)(1-\Phi)\Phi F_{CcBbAa}(i-1)+0.125(1-\Phi)M_{CcBbAA}(i-1)(1-\Phi)F_{CcBbAA}(i-1)+0.125(1-\Phi)M_{CcBbAA}(i-1)F_{CCbbAa}(i-1)+0.25(1-\Phi)M_{CcBbAA}(i-1)F_{CCbbAA}(i-1)+0.0625(1-\Phi)M_{CcBbAA}(i-1)(1-\Phi)(1-\Phi)F_{CCBbAa}(i-1)+0.125(1-\Phi)M_{CcBbAA}(i-1)(1-\Phi)F_{CCBbAA}(i-1)+0.25M_{CCbbAa}(i-1)F_{ccbbAa}(i-1)+0.5M_{CCbbAa}(i-1)F_{ccbbAA}(i-1)+0.125M_{CCbbAa}(i-1)(1-\Phi)F_{ccBbAa}(i-1)+0.25M_{CCbbAa}(i-1)\Phi F_{ccBbAa}(i-1)+0.25M_{CCbbAa}(i-1)F_{ccBbAA}(i-1)+0.125M_{CCbbAa}(i-1)F_{CcbbAa}(i-1)+0.25M_{CCbbAa}(i-1)F_{CcbbAA}(i-1)+0.0625M_{CCbbAa}(i-1)(1-\Phi)(1-\Phi)F_{CcBbAa}(i-1)+0.125M_{CCbbAa}(i-1)(1-\Phi)\Phi F_{CcBbAa}(i-1)+0.125M_{CCbbAa}(i-1)(1-\Phi)F_{CcBbAA}(i-1)+0.5M_{CCbbAA}(i-1)F_{ccbbAa}(i-1)+M_{CCbbAA}(i-1)F_{ccbbAA}(i-1)+0.25M_{CCbbAA}(i-1)(1-\Phi)F_{ccBbAa}(i-1)+0.5M_{CCbbAA}(i-1)\Phi F_{ccBbAa}(i-1)+0.5M_{CCbbAA}(i-1)F_{ccBbAA}(i-1)+0.25M_{CCbbAA}(i-1)F_{CcbbAa}(i-1)+0.5M_{CCbbAA}(i-1)F_{CcbbAA}(i-1)+0.125M_{CCbbAA}(i-1)(1-\Phi)(1-\Phi)F_{CcBbAa}(i-1)+0.25M_{CCbbAA}(i-1)(1-\Phi)\Phi F_{CcBbAa}(i-1)+0.25M_{CCbbAA}(i-1)(1-\Phi)F_{CcBbAA}(i-1)+0.125(1-\Phi)(1-\Phi)M_{CCBbAa}(i-1)F_{ccbbAa}(i-1)+0.25(1-\Phi)(1-\Phi)M_{CCBbAa}(i-1)F_{ccbbAA}(i-1)+0.0625(1-\Phi)(1-\Phi)M_{CCBbAa}(i-1)(1-\Phi)F_{ccBbAa}(i-1)+0.125(1-\Phi)(1-\Phi)M_{CCBbAa}(i-1)\Phi F_{ccBbAa}(i-1)+0.125(1-\Phi)(1-\Phi)M_{CCBbAa}(i-1)F_{ccBbAA}(i-1)+0.0625(1-\Phi)(1-\Phi)M_{CCBbAa}(i-1)F_{CcbbAa}(i-1)+0.125(1-\Phi)(1-\Phi)M_{CCBbAa}(i-1)F_{CcbbAA}(i-1)+0.03125(1-\Phi)(1-\Phi)M_{CCBbAa}(i-1)(1-\Phi)(1-\Phi)F_{CcBbAa}(i-1)+0.0625(1-\Phi)(1-\Phi)M_{CCBbAa}(i-1)(1-\Phi)\Phi F_{CcBbAa}(i-1)+0.0625(1-\Phi)(1-\Phi)M_{CCBbAa}(i-1)(1-\Phi)F_{CcBbAA}(i-1)+0.25(1-\Phi)\Phi M_{CCBbAa}(i-1)F_{ccbbAa}(i-1)+0.5(1-\Phi)\Phi M_{CCBbAa}(i-1)F_{ccbbAA}(i-1)+0.125(1-\Phi)\Phi M_{CCBbAa}(i-1)(1-\Phi)F_{ccBbAa}(i-1)+0.25(1-\Phi)\Phi M_{CCBbAa}(i-1)\Phi F_{ccBbAa}(i-1)+0.25(1-\Phi)\Phi M_{CCBbAa}(i-1)F_{ccBbAA}(i-1)+0.125(1-\Phi)\Phi M_{CCBbAa}(i-1)F_{CcbbAa}(i-1)+0.25(1-\Phi)\Phi M_{CCBbAa}(i-1)F_{CcbbAA}(i-1)+0.0625(1-\Phi)\Phi M_{CCBbAa}(i-1)(1-\Phi)(1-\Phi)F_{CcBbAa}(i-1)+0.125(1-\Phi)\Phi M_{CCBbAa}(i-1)(1-\Phi)\Phi F_{CcBbAa}(i-1)+0.125(1-\Phi)\Phi M_{CCBbAa}(i-1)(1-\Phi)F_{CcBbAA}(i-1)+0.25(1-\Phi)M_{CCBbAA}(i-1)F_{ccbbAa}(i-1)+0.5(1-\Phi)M_{CCBbAA}(i-1)F_{ccbbAA}(i-1)+0.125(1-\Phi)M_{CCBbAA}(i-1)(1-\Phi)F_{ccBbAa}(i-1)+0.25(1-\Phi)M_{CCBbAA}(i-1)\Phi F_{ccBbAa}(i-1)+0.25(1-\Phi)M_{CCBbAA}(i-1)F_{ccBbAA}(i-1)+0.125(1-\Phi)M_{CCBbAA}(i-1)F_{CcbbAa}(i-1)+0.25(1-\Phi)M_{CCBbAA}(i-1)F_{CcbbAA}(i-1)+0.0625(1-\Phi)M_{CCBbAA}(i-1)(1-\Phi)(1-\Phi)F_{CcBbAa}(i-1)+0.125(1-\Phi)M_{CCBbAA}(i-1)(1-\Phi)\Phi F_{CcBbAa}(i-1)+0.125(1-\Phi)M_{CCBbAA}(i-1)(1-\Phi)F_{CcBbAA}(i-1)],$$

$$\mathrm{M}^{e}_{CcBbaa} = \left(\frac{\bar{\Omega}_{CcBbaa}}{2}\right)[0.25M_{ccbbaa}(i-1)(1-\Phi)F_{CcBbaa}(i-1)+0.5M_{ccbbaa}(i-1)\Phi F_{CcBbaa}(i-1)+0.125M_{ccbbaa}(i-1)(1-\Phi)(1-\Phi)F_{CcBbAa}(i-1)+0.25M_{ccbbaa}(i-1)\Phi(1-\Phi)F_{CcBbAa}(i-1)+0.5M_{ccbbaa}(i-1)F_{CcBBaa}(i-1)+0.25M_{ccbbaa}(i-1)(1-\Phi)F_{CcBBAa}(i-1)+0.5M_{ccbbaa}(i-1)(1-$$

$\Phi)F_{CCBbaa}(i-1) + M_{ccbbaa}(i-1)\Phi F_{CCBbaa}(i-1) + 0.25M_{ccbbaa}(i-1)(1-\Phi)(1-\Phi)F_{CCBbAa}(i-$
$1) + 0.5M_{ccbbaa}(i-1)\Phi(1-\Phi)F_{CCBbAa}(i-1) + 0.5M_{ccbbaa}(i-1)(1-\Phi)\Phi F_{CCBbAa}(i-1) + M_{ccbbaa}(i-$
$1)F_{CCBBaa}(i-1) + 0.5M_{ccbbaa}(i-1)(1-\Phi)F_{CCBBAa}(i-1) + 0.125M_{ccbbAa}(i-1)(1-\Phi)F_{CcBbaa}(i-1) +$
$0.25M_{ccbbAa}(i-1)\Phi F_{CcBbaa}(i-1) + 0.0625M_{ccbbAa}(i-1)(1-\Phi)(1-\Phi)F_{CcBbAa}(i-1) + 0.125M_{ccbbAa}(i-$
$1)\Phi(1-\Phi)F_{CcBbAa}(i-1) + 0.25M_{ccbbAa}(i-1)F_{CcBBaa}(i-1) + 0.125M_{ccbbAa}(i-1)(1-\Phi)F_{CcBBAa}(i-$
$1) + 0.25M_{ccbbAa}(i-1)(1-\Phi)F_{CCBbaa}(i-1) + 0.5M_{ccbbAa}(i-1)\Phi F_{CCBbaa}(i-1) + 0.125M_{ccbbAa}(i-$
$1)(1-\Phi)(1-\Phi)F_{CCBbAa}(i-1) + 0.25M_{ccbbAa}(i-1)\Phi(1-\Phi)F_{CCBbAa}(i-1) + 0.25M_{ccbbAa}(i-1)(1-$
$\Phi)\Phi F_{CCBbAa}(i-1) + 0.5M_{ccbbAa}(i-1)F_{CCBBaa}(i-1) + 0.25M_{ccbbAa}(i-1)(1-\Phi)F_{CCBBAa}(i-1) +$
$0.25M_{ccBbaa}(i-1)F_{Ccbbaa}(i-1) + 0.125M_{ccBbaa}(i-1)F_{CcbbAa}(i-1) + 0.25M_{ccBbaa}(i-1)(1-\Phi)F_{CcBbaa}(i-$
$1) + 0.25M_{ccBbaa}(i-1)\Phi F_{CcBbaa}(i-1) + 0.125M_{ccBbaa}(i-1)(1-\Phi)(1-\Phi)F_{CcBbAa}(i-1) + 0.125M_{ccBbaa}(i-$
$1)\Phi(1-\Phi)F_{CcBbAa}(i-1) + 0.25M_{ccBbaa}(i-1)F_{CcBBaa}(i-1) + 0.125M_{ccBbaa}(i-1)(1-\Phi)F_{CcBBAa}(i-1) +$
$0.5M_{ccBbaa}(i-1)F_{CCbbaa}(i-1) + 0.25M_{ccBbaa}(i-1)F_{CCbbAa}(i-1) + 0.5M_{ccBbaa}(i-1)(1-\Phi)F_{CCBbaa}(i-$
$1) + 0.5M_{ccBbaa}(i-1)\Phi F_{CCBbaa}(i-1) + 0.25M_{ccBbaa}(i-1)(1-\Phi)(1-\Phi)F_{CCBbAa}(i-1) + 0.25M_{ccBbaa}(i-$
$1)\Phi(1-\Phi)F_{CCBbAa}(i-1) + 0.25M_{ccBbaa}(i-1)(1-\Phi)\Phi F_{CCBbAa}(i-1) + 0.5M_{ccBbaa}(i-1)F_{CCBBaa}(i-$
$1) + 0.25M_{ccBbaa}(i-1)(1-\Phi)F_{CCBBAa}(i-1) + 0.125(1-\Phi)M_{ccBbAa}(i-1)F_{Ccbbaa}(i-1) + 0.0625(1-$
$\Phi)M_{ccBbAa}(i-1)F_{CcbbAa}(i-1) + 0.125(1-\Phi)M_{ccBbAa}(i-1)(1-\Phi)F_{CcBbaa}(i-1) + 0.125(1-\Phi)M_{ccBbAa}(i-$
$1)\Phi F_{CcBbaa}(i-1) + 0.0625(1-\Phi)M_{ccBbAa}(i-1)(1-\Phi)(1-\Phi)F_{CcBbAa}(i-1) + 0.0625(1-\Phi)M_{ccBbAa}(i-$
$1)\Phi(1-\Phi)F_{CcBbAa}(i-1) + 0.125(1-\Phi)M_{ccBbAa}(i-1)F_{CcBBaa}(i-1) + 0.0625(1-\Phi)M_{ccBbAa}(i-1)(1-$
$\Phi)F_{CcBBAa}(i-1) + 0.25(1-\Phi)M_{ccBbAa}(i-1)F_{CCbbaa}(i-1) + 0.125(1-\Phi)M_{ccBbAa}(i-1)F_{CCbbAa}(i-1) +$
$0.25(1-\Phi)M_{ccBbAa}(i-1)(1-\Phi)F_{CCBbaa}(i-1) + 0.25(1-\Phi)M_{ccBbAa}(i-1)\Phi F_{CCBbaa}(i-1) + 0.125(1-$
$\Phi)M_{ccBbAa}(i-1)(1-\Phi)(1-\Phi)F_{CCBbAa}(i-1) + 0.125(1-\Phi)M_{ccBbAa}(i-1)\Phi(1-\Phi)F_{CCBbAa}(i-1) +$
$0.125(1-\Phi)M_{ccBbAa}(i-1)(1-\Phi)\Phi F_{CCBbAa}(i-1) + 0.25(1-\Phi)M_{ccBbAa}(i-1)F_{CCBBaa}(i-1) + 0.125(1-$
$\Phi)M_{ccBbAa}(i-1)(1-\Phi)F_{CCBBAa}(i-1) + 0.5M_{ccBBaa}(i-1)F_{Ccbbaa}(i-1) + 0.25M_{ccBBaa}(i-1)F_{CcbbAa}(i-$
$1) + 0.25M_{ccBBaa}(i-1)(1-\Phi)F_{CcBbaa}(i-1) + 0.125M_{ccBBaa}(i-1)(1-\Phi)(1-\Phi)F_{CcBbAa}(i-1) +$
$M_{ccBBaa}(i-1)F_{CCbbaa}(i-1) + 0.5M_{ccBBaa}(i-1)F_{CCbbAa}(i-1) + 0.5M_{ccBBaa}(i-1)(1-\Phi)F_{CCBbaa}(i-$
$1) + 0.25M_{ccBBaa}(i-1)(1-\Phi)(1-\Phi)F_{CCBbAa}(i-1) + 0.25(1-\Phi)M_{ccBBAa}(i-1)F_{Ccbbaa}(i-1) +$
$0.125(1-\Phi)M_{ccBBAa}(i-1)F_{CcbbAa}(i-1) + 0.125(1-\Phi)M_{ccBBAa}(i-1)(1-\Phi)F_{CcBbaa}(i-1) + 0.0625(1-$
$\Phi)M_{ccBBAa}(i-1)(1-\Phi)(1-\Phi)F_{CcBbAa}(i-1) + 0.5(1-\Phi)M_{ccBBAa}(i-1)F_{CCbbaa}(i-1) + 0.25(1-$
$\Phi)M_{ccBBAa}(i-1)F_{CCbbAa}(i-1) + 0.25(1-\Phi)M_{ccBBAa}(i-1)(1-\Phi)F_{CCBbaa}(i-1) + 0.125(1-\Phi)M_{ccBBAa}(i-$
$1)(1-\Phi)(1-\Phi)F_{CCBbAa}(i-1) + 0.25M_{Ccbbaa}(i-1)F_{ccBbaa}(i-1) + 0.125M_{Ccbbaa}(i-1)(1-\Phi)F_{ccBbAa}(i-$
$1) + 0.5M_{Ccbbaa}(i-1)F_{ccBBaa}(i-1) + 0.25M_{Ccbbaa}(i-1)(1-\Phi)F_{ccBBAa}(i-1) + 0.25M_{Ccbbaa}(i-1)(1-$
$\Phi)F_{CcBbaa}(i-1) + 0.5M_{Ccbbaa}(i-1)\Phi F_{CcBbaa}(i-1) + 0.125M_{Ccbbaa}(i-1)(1-\Phi)(1-\Phi)F_{CcBbAa}(i-$
$1) + 0.25M_{Ccbbaa}(i-1)\Phi(1-\Phi)F_{CcBbAa}(i-1) + 0.5M_{Ccbbaa}(i-1)F_{CcBBaa}(i-1) + 0.25M_{Ccbbaa}(i-$
$1)(1-\Phi)F_{CcBBAa}(i-1) + 0.25M_{Ccbbaa}(i-1)(1-\Phi)F_{CCBbaa}(i-1) + 0.5M_{Ccbbaa}(i-1)\Phi F_{CCBbaa}(i-$
$1) + 0.125M_{Ccbbaa}(i-1)(1-\Phi)(1-\Phi)F_{CCBbAa}(i-1) + 0.25M_{Ccbbaa}(i-1)\Phi(1-\Phi)F_{CCBbAa}(i-1) +$
$0.25M_{Ccbbaa}(i-1)(1-\Phi)\Phi F_{CCBbAa}(i-1) + 0.5M_{Ccbbaa}(i-1)F_{CCBBaa}(i-1) + 0.25M_{Ccbbaa}(i-1)(1-$
$\Phi)F_{CCBBAa}(i-1) + 0.125M_{CcbbAa}(i-1)F_{ccBbaa}(i-1) + 0.0625M_{CcbbAa}(i-1)(1-\Phi)F_{ccBbAa}(i-1) +$
$0.25M_{CcbbAa}(i-1)F_{ccBBaa}(i-1) + 0.125M_{CcbbAa}(i-1)(1-\Phi)F_{ccBBAa}(i-1) + 0.125M_{CcbbAa}(i-1)(1-$
$\Phi)F_{CcBbaa}(i-1) + 0.25M_{CcbbAa}(i-1)\Phi F_{CcBbaa}(i-1) + 0.0625M_{CcbbAa}(i-1)(1-\Phi)(1-\Phi)F_{CcBbAa}(i-$
$1) + 0.125M_{CcbbAa}(i-1)\Phi(1-\Phi)F_{CcBbAa}(i-1) + 0.25M_{CcbbAa}(i-1)F_{CcBBaa}(i-1) + 0.125M_{CcbbAa}(i-$
$1)(1-\Phi)F_{CcBBAa}(i-1) + 0.125M_{CcbbAa}(i-1)(1-\Phi)F_{CCBbaa}(i-1) + 0.25M_{CcbbAa}(i-1)\Phi F_{CCBbaa}(i-$
$1) + 0.0625M_{CcbbAa}(i-1)(1-\Phi)(1-\Phi)F_{CCBbAa}(i-1) + 0.125M_{CcbbAa}(i-1)\Phi(1-\Phi)F_{CCBbAa}(i-1) +$
$0.125M_{CcbbAa}(i-1)(1-\Phi)\Phi F_{CCBbAa}(i-1) + 0.25M_{CcbbAa}(i-1)F_{CCBBaa}(i-1) + 0.125M_{CcbbAa}(i-1)(1-$
$\Phi)F_{CCBBAa}(i-1) + 0.25(1-\Phi)M_{CcBbaa}(i-1)F_{ccbbaa}(i-1) + 0.125(1-\Phi)M_{CcBbaa}(i-1)F_{ccbbAa}(i-1) +$
$0.25(1-\Phi)M_{CcBbaa}(i-1)F_{ccBbaa}(i-1) + 0.125(1-\Phi)M_{CcBbaa}(i-1)(1-\Phi)F_{ccBbAa}(i-1) + 0.25(1-$
$\Phi)M_{CcBbaa}(i-1)F_{ccBBaa}(i-1) + 0.125(1-\Phi)M_{CcBbaa}(i-1)(1-\Phi)F_{ccBBAa}(i-1) + 0.25(1-\Phi)M_{CcBbaa}(i-$
$1)F_{Ccbbaa}(i-1) + 0.125(1-\Phi)M_{CcBbaa}(i-1)F_{CcbbAa}(i-1) + 0.25(1-\Phi)M_{CcBbaa}(i-1)(1-\Phi)F_{CcBbaa}(i-$
$1) + 0.25(1-\Phi)M_{CcBbaa}(i-1)\Phi F_{CcBbaa}(i-1) + 0.125(1-\Phi)M_{CcBbaa}(i-1)(1-\Phi)(1-\Phi)F_{CcBbAa}(i-$
$1) + 0.125(1-\Phi)M_{CcBbaa}(i-1)\Phi(1-\Phi)F_{CcBbAa}(i-1) + 0.25(1-\Phi)M_{CcBbaa}(i-1)F_{CcBBaa}(i-1) +$
$0.125(1-\Phi)M_{CcBbaa}(i-1)(1-\Phi)F_{CcBBAa}(i-1) + 0.25(1-\Phi)M_{CcBbaa}(i-1)F_{CCbbaa}(i-1) + 0.125(1-$
$\Phi)M_{CcBbaa}(i-1)F_{CCbbAa}(i-1) + 0.25(1-\Phi)M_{CcBbaa}(i-1)(1-\Phi)F_{CCBbaa}(i-1) + 0.25(1-\Phi)M_{CcBbaa}(i-$
$1)\Phi F_{CCBbaa}(i-1) + 0.125(1-\Phi)M_{CcBbaa}(i-1)(1-\Phi)(1-\Phi)F_{CCBbAa}(i-1) + 0.125(1-\Phi)M_{CcBbaa}(i-$
$1)\Phi(1-\Phi)F_{CCBbAa}(i-1) + 0.125(1-\Phi)M_{CcBbaa}(i-1)(1-\Phi)\Phi F_{CCBbAa}(i-1) + 0.25(1-\Phi)M_{CcBbaa}(i-$
$1)F_{CCBBaa}(i-1) + 0.125(1-\Phi)M_{CcBbaa}(i-1)(1-\Phi)F_{CCBBAa}(i-1) + 0.5\Phi M_{CcBbaa}(i-1)F_{ccbbaa}(i-$
$1) + 0.25\Phi M_{CcBbaa}(i-1)F_{ccbbAa}(i-1) + 0.25\Phi M_{CcBbaa}(i-1)F_{ccBbaa}(i-1) + 0.125\Phi M_{CcBbaa}(i-1)(1-$
$\Phi)F_{ccBbAa}(i-1) + 0.5\Phi M_{CcBbaa}(i-1)F_{Ccbbaa}(i-1) + 0.25\Phi M_{CcBbaa}(i-1)F_{CcbbAa}(i-1) + 0.25\Phi M_{CcBbaa}(i-$
$1)(1-\Phi)F_{CcBbaa}(i-1) + 0.125\Phi M_{CcBbaa}(i-1)(1-\Phi)(1-\Phi)F_{CcBbAa}(i-1) + 0.5\Phi M_{CcBbaa}(i-1)F_{CCbbaa}(i-$

$1)+0.25\Phi M_{CcBbaa}(i-1)F_{CCbbAa}(i-1)+0.25\Phi M_{CcBbaa}(i-1)(1-\Phi)F_{CCBbaa}(i-1)+0.125\Phi M_{CcBbaa}(i-$
$1)(1-\Phi)(1-\Phi)F_{CCBbAa}(i-1)+0.125(1-\Phi)(1-\Phi)M_{CcBbAa}(i-1)F_{ccbbaa}(i-1)+0.0625(1-\Phi)(1-$
$\Phi)M_{CcBbAa}(i-1)F_{ccbbAa}(i-1)+0.125(1-\Phi)(1-\Phi)M_{CcBbAa}(i-1)F_{ccBbaa}(i-1)+0.0625(1-\Phi)(1-$
$\Phi)M_{CcBbAa}(i-1)(1-\Phi)F_{ccBbAa}(i-1)+0.125(1-\Phi)(1-\Phi)M_{CcBbAa}(i-1)F_{ccBBaa}(i-1)+0.0625(1-$
$\Phi)(1-\Phi)M_{CcBbAa}(i-1)(1-\Phi)F_{ccBBAa}(i-1)+0.125(1-\Phi)(1-\Phi)M_{CcBbAa}(i-1)F_{Ccbbaa}(i-1)+$
$0.0625(1-\Phi)(1-\Phi)M_{CcBbAa}(i-1)F_{CcbbAa}(i-1)+0.125(1-\Phi)(1-\Phi)M_{CcBbAa}(i-1)(1-\Phi)F_{CcBbaa}(i-$
$1)+0.125(1-\Phi)(1-\Phi)M_{CcBbAa}(i-1)\Phi F_{CcBbaa}(i-1)+0.0625(1-\Phi)(1-\Phi)M_{CcBbAa}(i-1)(1-\Phi)(1-$
$\Phi)F_{CcBbAa}(i-1)+0.0625(1-\Phi)(1-\Phi)M_{CcBbAa}(i-1)\Phi(1-\Phi)F_{CcBbAa}(i-1)+0.125(1-\Phi)(1-$
$\Phi)M_{CcBbAa}(i-1)F_{CcBBaa}(i-1)+0.0625(1-\Phi)(1-\Phi)M_{CcBbAa}(i-1)(1-\Phi)F_{CcBBAa}(i-1)+0.125(1-$
$\Phi)(1-\Phi)M_{CcBbAa}(i-1)F_{CCbbaa}(i-1)+0.0625(1-\Phi)(1-\Phi)M_{CcBbAa}(i-1)F_{CCbbAa}(i-1)+0.125(1-$
$\Phi)(1-\Phi)M_{CcBbAa}(i-1)(1-\Phi)F_{CCBbaa}(i-1)+0.125(1-\Phi)(1-\Phi)M_{CcBbAa}(i-1)\Phi F_{CCBbaa}(i-1)+$
$0.0625(1-\Phi)(1-\Phi)M_{CcBbAa}(i-1)(1-\Phi)(1-\Phi)F_{CCBbAa}(i-1)+0.0625(1-\Phi)(1-\Phi)M_{CcBbAa}(i-$
$1)\Phi(1-\Phi)F_{CCBbAa}(i-1)+0.0625(1-\Phi)(1-\Phi)M_{CcBbAa}(i-1)(1-\Phi)\Phi F_{CCBbAa}(i-1)+0.125(1-\Phi)(1-$
$\Phi)M_{CcBbAa}(i-1)F_{CCBBaa}(i-1)+0.0625(1-\Phi)(1-\Phi)M_{CcBbAa}(i-1)(1-\Phi)F_{CCBBAa}(i-1)+0.25\Phi(1-$
$\Phi)M_{CcBbAa}(i-1)F_{ccbbaa}(i-1)+0.125\Phi(1-\Phi)M_{CcBbAa}(i-1)F_{ccbbAa}(i-1)+0.125\Phi(1-\Phi)M_{CcBbAa}(i-$
$1)F_{ccBbaa}(i-1)+0.0625\Phi(1-\Phi)M_{CcBbAa}(i-1)(1-\Phi)F_{ccBbAa}(i-1)+0.25\Phi(1-\Phi)M_{CcBbAa}(i-$
$1)F_{Ccbbaa}(i-1)+0.125\Phi(1-\Phi)M_{CcBbAa}(i-1)F_{CcbbAa}(i-1)+0.125\Phi(1-\Phi)M_{CcBbAa}(i-1)(1-$
$\Phi)F_{CcBbaa}(i-1)+0.0625\Phi(1-\Phi)M_{CcBbAa}(i-1)(1-\Phi)(1-\Phi)F_{CcBbAa}(i-1)+0.25\Phi(1-\Phi)M_{CcBbAa}(i-$
$1)F_{CCbbaa}(i-1)+0.125\Phi(1-\Phi)M_{CcBbAa}(i-1)F_{CCbbAa}(i-1)+0.125\Phi(1-\Phi)M_{CcBbAa}(i-1)(1-$
$\Phi)F_{CCBbaa}(i-1)+0.0625\Phi(1-\Phi)M_{CcBbAa}(i-1)(1-\Phi)(1-\Phi)F_{CCBbAa}(i-1)+0.5M_{CcBBaa}(i-1)F_{ccbbaa}(i-$
$1)+0.25M_{CcBBaa}(i-1)F_{ccbbAa}(i-1)+0.25M_{CcBBaa}(i-1)F_{ccBbaa}(i-1)+0.125M_{CcBBaa}(i-1)(1-$
$\Phi)F_{ccBbAa}(i-1)+0.5M_{CcBBaa}(i-1)F_{Ccbbaa}(i-1)+0.25M_{CcBBaa}(i-1)F_{CcbbAa}(i-1)+0.25M_{CcBBaa}(i-$
$1)(1-\Phi)F_{CcBbaa}(i-1)+0.125M_{CcBBaa}(i-1)(1-\Phi)(1-\Phi)F_{CcBbAa}(i-1)+0.5M_{CcBBaa}(i-1)F_{CCbbaa}(i-$
$1)+0.25M_{CcBBaa}(i-1)F_{CCbbAa}(i-1)+0.25M_{CcBBaa}(i-1)(1-\Phi)F_{CCBbaa}(i-1)+0.125M_{CcBBaa}(i-$
$1)(1-\Phi)(1-\Phi)F_{CCBbAa}(i-1)+0.25(1-\Phi)M_{CcBBAa}(i-1)F_{ccbbaa}(i-1)+0.125(1-\Phi)M_{CcBBAa}(i-$
$1)F_{ccbbAa}(i-1)+0.125(1-\Phi)M_{CcBBAa}(i-1)F_{ccBbaa}(i-1)+0.0625(1-\Phi)M_{CcBBAa}(i-1)(1-\Phi)F_{ccBbAa}(i-$
$1)+0.25(1-\Phi)M_{CcBBAa}(i-1)F_{Ccbbaa}(i-1)+0.125(1-\Phi)M_{CcBBAa}(i-1)F_{CcbbAa}(i-1)+0.125(1-$
$\Phi)M_{CcBBAa}(i-1)(1-\Phi)F_{CcBbaa}(i-1)+0.0625(1-\Phi)M_{CcBBAa}(i-1)(1-\Phi)(1-\Phi)F_{CcBbAa}(i-1)+0.25(1-$
$\Phi)M_{CcBBAa}(i-1)F_{CCbbaa}(i-1)+0.125(1-\Phi)M_{CcBBAa}(i-1)F_{CCbbAa}(i-1)+0.125(1-\Phi)M_{CcBBAa}(i-$
$1)(1-\Phi)F_{CCBbaa}(i-1)+0.0625(1-\Phi)M_{CcBBAa}(i-1)(1-\Phi)(1-\Phi)F_{CCBbAa}(i-1)+0.5M_{CCbbaa}(i-$
$1)F_{ccBbaa}(i-1)+0.25M_{CCbbaa}(i-1)(1-\Phi)F_{ccBbAa}(i-1)+M_{CCbbaa}(i-1)F_{ccBBaa}(i-1)+0.5M_{CCbbaa}(i-$
$1)(1-\Phi)F_{ccBBAa}(i-1)+0.25M_{CCbbaa}(i-1)(1-\Phi)F_{CcBbaa}(i-1)+0.5M_{CCbbaa}(i-1)\Phi F_{CcBbaa}(i-$
$1)+0.125M_{CCbbaa}(i-1)(1-\Phi)(1-\Phi)F_{CcBbAa}(i-1)+0.25M_{CCbbaa}(i-1)\Phi(1-\Phi)F_{CcBbAa}(i-1)+$
$0.5M_{CCbbaa}(i-1)F_{CcBBaa}(i-1)+0.25M_{CCbbaa}(i-1)(1-\Phi)F_{CcBBAa}(i-1)+0.25M_{CCbbAa}(i-1)F_{ccBbaa}(i-$
$1)+0.125M_{CCbbAa}(i-1)(1-\Phi)F_{ccBbAa}(i-1)+0.5M_{CCbbAa}(i-1)F_{ccBBaa}(i-1)+0.25M_{CCbbAa}(i-$
$1)(1-\Phi)F_{ccBBAa}(i-1)+0.125M_{CCbbAa}(i-1)(1-\Phi)F_{CcBbaa}(i-1)+0.25M_{CCbbAa}(i-1)\Phi F_{CcBbaa}(i-$
$1)+0.0625M_{CCbbAa}(i-1)(1-\Phi)(1-\Phi)F_{CcBbAa}(i-1)+0.125M_{CCbbAa}(i-1)\Phi(1-\Phi)F_{CcBbAa}(i-1)+$
$0.25M_{CCbbAa}(i-1)F_{CcBBaa}(i-1)+0.125M_{CCbbAa}(i-1)(1-\Phi)F_{CcBBAa}(i-1)+0.5(1-\Phi)M_{CCBbaa}(i-$
$1)F_{ccbbaa}(i-1)+0.25(1-\Phi)M_{CCBbaa}(i-1)F_{ccbbAa}(i-1)+0.5(1-\Phi)M_{CCBbaa}(i-1)F_{ccBbaa}(i-1)+0.25(1-$
$\Phi)M_{CCBbaa}(i-1)(1-\Phi)F_{ccBbAa}(i-1)+0.5(1-\Phi)M_{CCBbaa}(i-1)F_{ccBBaa}(i-1)+0.25(1-\Phi)M_{CCBbaa}(i-$
$1)(1-\Phi)F_{ccBBAa}(i-1)+0.25(1-\Phi)M_{CCBbaa}(i-1)F_{Ccbbaa}(i-1)+0.125(1-\Phi)M_{CCBbaa}(i-1)F_{CcbbAa}(i-$
$1)+0.25(1-\Phi)M_{CCBbaa}(i-1)(1-\Phi)F_{CcBbaa}(i-1)+0.25(1-\Phi)M_{CCBbaa}(i-1)\Phi F_{CcBbaa}(i-1)+0.125(1-$
$\Phi)M_{CCBbaa}(i-1)(1-\Phi)(1-\Phi)F_{CcBbAa}(i-1)+0.125(1-\Phi)M_{CCBbaa}(i-1)\Phi(1-\Phi)F_{CcBbAa}(i-1)+0.25(1-$
$\Phi)M_{CCBbaa}(i-1)F_{CcBBaa}(i-1)+0.125(1-\Phi)M_{CCBbaa}(i-1)(1-\Phi)F_{CcBBAa}(i-1)+\Phi M_{CCBbaa}(i-$
$1)F_{ccbbaa}(i-1)+0.5\Phi M_{CCBbaa}(i-1)F_{ccbbAa}(i-1)+0.5\Phi M_{CCBbaa}(i-1)F_{ccBbaa}(i-1)+0.25\Phi M_{CCBbaa}(i-$
$1)(1-\Phi)F_{ccBbAa}(i-1)+0.5\Phi M_{CCBbaa}(i-1)F_{Ccbbaa}(i-1)+0.25\Phi M_{CCBbaa}(i-1)F_{CcbbAa}(i-1)+$
$0.25\Phi M_{CCBbaa}(i-1)(1-\Phi)F_{CcBbaa}(i-1)+0.125\Phi M_{CCBbaa}(i-1)(1-\Phi)(1-\Phi)F_{CcBbAa}(i-1)+0.25(1-$
$\Phi)(1-\Phi)M_{CCBbAa}(i-1)F_{ccbbaa}(i-1)+0.125(1-\Phi)(1-\Phi)M_{CCBbAa}(i-1)F_{ccbbAa}(i-1)+0.25(1-\Phi)(1-$
$\Phi)M_{CCBbAa}(i-1)F_{ccBbaa}(i-1)+0.125(1-\Phi)(1-\Phi)M_{CCBbAa}(i-1)(1-\Phi)F_{ccBbAa}(i-1)+0.25(1-\Phi)(1-$
$\Phi)M_{CCBbAa}(i-1)F_{ccBBaa}(i-1)+0.125(1-\Phi)(1-\Phi)M_{CCBbAa}(i-1)(1-\Phi)F_{ccBBAa}(i-1)+0.125(1-$
$\Phi)(1-\Phi)M_{CCBbAa}(i-1)F_{Ccbbaa}(i-1)+0.0625(1-\Phi)(1-\Phi)M_{CCBbAa}(i-1)F_{CcbbAa}(i-1)+0.125(1-$
$\Phi)(1-\Phi)M_{CCBbAa}(i-1)(1-\Phi)F_{CcBbaa}(i-1)+0.125(1-\Phi)(1-\Phi)M_{CCBbAa}(i-1)\Phi F_{CcBbaa}(i-1)+$
$0.0625(1-\Phi)(1-\Phi)M_{CCBbAa}(i-1)(1-\Phi)(1-\Phi)F_{CcBbAa}(i-1)+0.0625(1-\Phi)(1-\Phi)M_{CCBbAa}(i-1)\Phi(1-$
$\Phi)F_{CcBbAa}(i-1)+0.125(1-\Phi)(1-\Phi)M_{CCBbAa}(i-1)F_{CcBBaa}(i-1)+0.0625(1-\Phi)(1-\Phi)M_{CCBbAa}(i-$
$1)(1-\Phi)F_{CcBBAa}(i-1)+0.5\Phi(1-\Phi)M_{CCBbAa}(i-1)F_{ccbbaa}(i-1)+0.25\Phi(1-\Phi)M_{CCBbAa}(i-1)F_{ccbbAa}(i-$
$1)+0.25\Phi(1-\Phi)M_{CCBbAa}(i-1)F_{ccBbaa}(i-1)+0.125\Phi(1-\Phi)M_{CCBbAa}(i-1)(1-\Phi)F_{ccBbAa}(i-1)+$
$0.25\Phi(1-\Phi)M_{CCBbAa}(i-1)F_{Ccbbaa}(i-1)+0.125\Phi(1-\Phi)M_{CCBbAa}(i-1)F_{CcbbAa}(i-1)+0.125\Phi(1-$

$$\Phi)M_{CCBbAa}(i-1)(1-\Phi)F_{CcBbaa}(i-1)+0.0625\Phi(1-\Phi)M_{CCBbAa}(i-1)(1-\Phi)(1-\Phi)F_{CcBbAa}(i-1)+0.5(1-\Phi)\Phi M_{CCBbAa}(i-1)F_{ccbbaa}(i-1)+M_{CCBBaa}(i-1)F_{ccbbaa}(i-1)+0.5M_{CCBBaa}(i-1)F_{ccbbAa}(i-1)+0.5M_{CCBBaa}(i-1)F_{ccBbaa}(i-1)+0.25M_{CCBBaa}(i-1)(1-\Phi)F_{ccBbAa}(i-1)+0.5M_{CCBBaa}(i-1)F_{Ccbbaa}(i-1)+0.25M_{CCBBaa}(i-1)F_{CcbbAa}(i-1)+0.25M_{CCBBaa}(i-1)(1-\Phi)F_{CcBbaa}(i-1)+0.125M_{CCBBaa}(i-1)(1-\Phi)(1-\Phi)F_{CcBbAa}(i-1)+0.5(1-\Phi)M_{CCBBAa}(i-1)F_{ccbbaa}(i-1)+0.25(1-\Phi)M_{CCBBAa}(i-1)F_{ccbbAa}(i-1)+0.25(1-\Phi)M_{CCBBAa}(i-1)F_{ccBbaa}(i-1)+0.125(1-\Phi)M_{CCBBAa}(i-1)(1-\Phi)F_{ccBbAa}(i-1)+0.25(1-\Phi)M_{CCBBAa}(i-1)F_{Ccbbaa}(i-1)+0.125(1-\Phi)M_{CCBBAa}(i-1)F_{CcbbAa}(i-1)+0.125(1-\Phi)M_{CCBBAa}(i-1)(1-\Phi)F_{CcBbaa}(i-1)+0.0625(1-\Phi)M_{CCBBAa}(i-1)(1-\Phi)(1-\Phi)F_{CcBbAa}(i-1)],$$

$$\mathrm{M}^{e}_{CcBbAa}=\left(\frac{\bar{\Omega}_{CcBbAa}}{2}\right)[0.125M_{ccbbaa}(i-1)(1-\Phi)(1-\Phi)F_{CcBbAa}(i-1)+0.25M_{ccbbaa}(i-1)\Phi(1-\Phi)F_{CcBbAa}(i-1)+0.25M_{ccbbaa}(i-1)(1-\Phi)\Phi F_{CcBbAa}(i-1)+0.5M_{ccbbaa}(i-1)\Phi\Phi F_{CcBbAa}(i-1)+0.25M_{ccbbaa}(i-1)(1-\Phi)F_{CcBbAA}(i-1)+0.5M_{ccbbaa}(i-1)\Phi F_{CcBbAA}(i-1)+0.25M_{ccbbaa}(i-1)(1-\Phi)F_{CcBBAa}(i-1)+0.5M_{ccbbaa}(i-1)\Phi F_{CcBBAa}(i-1)+0.5M_{ccbbaa}(i-1)F_{CcBBAA}(i-1)+0.25M_{ccbbaa}(i-1)(1-\Phi)(1-\Phi)F_{CCBbAa}(i-1)+0.5M_{ccbbaa}(i-1)\Phi(1-\Phi)F_{CCBbAa}(i-1)+0.5M_{ccbbaa}(i-1)(1-\Phi)\Phi F_{CCBbAa}(i-1)+M_{ccbbaa}(i-1)\Phi\Phi F_{CCBbAa}(i-1)+0.5M_{ccbbaa}(i-1)(1-\Phi)F_{CCBbAA}(i-1)+M_{ccbbaa}(i-1)\Phi F_{CCBbAA}(i-1)+0.5M_{ccbbaa}(i-1)(1-\Phi)F_{CCBBAa}(i-1)+M_{ccbbaa}(i-1)\Phi F_{CCBBAa}(i-1)+M_{ccbbaa}(i-1)F_{CCBBAA}(i-1)+0.125M_{ccbbAa}(i-1)(1-\Phi)F_{CcBbaa}(i-1)+0.25M_{ccbbAa}(i-1)\Phi F_{CcBbaa}(i-1)+0.125M_{ccbbAa}(i-1)(1-\Phi)(1-\Phi)F_{CcBbAa}(i-1)+0.25M_{ccbbAa}(i-1)\Phi(1-\Phi)F_{CcBbAa}(i-1)+0.125M_{ccbbAa}(i-1)(1-\Phi)\Phi F_{CcBbAa}(i-1)+0.25M_{ccbbAa}(i-1)\Phi\Phi F_{CcBbAa}(i-1)+0.125M_{ccbbAa}(i-1)(1-\Phi)F_{CcBbAA}(i-1)+0.25M_{ccbbAa}(i-1)\Phi F_{CcBbAA}(i-1)+0.25M_{ccbbAa}(i-1)F_{CcBBaa}(i-1)+0.25M_{ccbbAa}(i-1)(1-\Phi)F_{CcBBAa}(i-1)+0.25M_{ccbbAa}(i-1)\Phi F_{CcBBAa}(i-1)+0.25M_{ccbbAa}(i-1)F_{CcBBAA}(i-1)+0.25M_{ccbbAa}(i-1)(1-\Phi)F_{CCBbaa}(i-1)+0.5M_{ccbbAa}(i-1)\Phi F_{CCBbaa}(i-1)+0.25M_{ccbbAa}(i-1)(1-\Phi)(1-\Phi)F_{CCBbAa}(i-1)+0.5M_{ccbbAa}(i-1)\Phi(1-\Phi)F_{CCBbAa}(i-1)+0.5M_{ccbbAa}(i-1)(1-\Phi)\Phi F_{CCBbAa}(i-1)+0.5M_{ccbbAa}(i-1)\Phi\Phi F_{CCBbAa}(i-1)+0.25M_{ccbbAa}(i-1)(1-\Phi)F_{CCBbAA}(i-1)+0.5M_{ccbbAa}(i-1)\Phi F_{CCBbAA}(i-1)+0.5M_{ccbbAa}(i-1)F_{CCBBaa}(i-1)+0.5M_{ccbbAa}(i-1)(1-\Phi)F_{CCBBAa}(i-1)+0.5M_{ccbbAa}(i-1)\Phi F_{CCBBAa}(i-1)+0.5M_{ccbbAa}(i-1)F_{CCBBAA}(i-1)+0.25M_{ccbbAA}(i-1)(1-\Phi)F_{CcBbaa}(i-1)+0.5M_{ccbbAA}(i-1)\Phi F_{CcBbaa}(i-1)+0.125M_{ccbbAA}(i-1)(1-\Phi)(1-\Phi)F_{CcBbAa}(i-1)+0.25M_{ccbbAA}(i-1)\Phi(1-\Phi)F_{CcBbAa}(i-1)+0.5M_{ccbbAA}(i-1)F_{CcBBaa}(i-1)+0.25M_{ccbbAA}(i-1)(1-\Phi)F_{CcBBAa}(i-1)+0.5M_{ccbbAA}(i-1)(1-\Phi)F_{CCBbaa}(i-1)+M_{ccbbAA}(i-1)\Phi F_{CCBbaa}(i-1)+0.25M_{ccbbAA}(i-1)(1-\Phi)(1-\Phi)F_{CCBbAa}(i-1)+0.5M_{ccbbAA}(i-1)\Phi(1-\Phi)F_{CCBbAa}(i-1)+0.5M_{ccbbAA}(i-1)(1-\Phi)\Phi F_{CCBbAa}(i-1)+M_{ccbbAA}(i-1)F_{CCBBaa}(i-1)+0.5M_{ccbbAA}(i-1)(1-\Phi)F_{CCBBAa}(i-1)+0.125M_{ccBbaa}(i-1)F_{CcbbAa}(i-1)+0.25M_{ccBbaa}(i-1)F_{CcbbAA}(i-1)+0.125M_{ccBbaa}(i-1)(1-\Phi)(1-\Phi)F_{CcBbAa}(i-1)+0.125M_{ccBbaa}(i-1)\Phi(1-\Phi)F_{CcBbAa}(i-1)+0.25M_{ccBbaa}(i-1)(1-\Phi)\Phi F_{CcBbAa}(i-1)+0.25M_{ccBbaa}(i-1)\Phi\Phi F_{CcBbAa}(i-1)+0.25M_{ccBbaa}(i-1)(1-\Phi)F_{CcBbAA}(i-1)+0.25M_{ccBbaa}(i-1)\Phi F_{CcBbAA}(i-1)+0.125M_{ccBbaa}(i-1)(1-\Phi)F_{CcBBAa}(i-1)+0.25M_{ccBbaa}(i-1)\Phi F_{CcBBAa}(i-1)+0.25M_{ccBbaa}(i-1)F_{CcBBAA}(i-1)+0.25M_{ccBbaa}(i-1)F_{CCbbAa}(i-1)+0.5M_{ccBbaa}(i-1)F_{CCbbAA}(i-1)+0.25M_{ccBbaa}(i-1)(1-\Phi)(1-\Phi)F_{CCBbAa}(i-1)+0.25M_{ccBbaa}(i-1)\Phi(1-\Phi)F_{CCBbAa}(i-1)+0.25M_{ccBbaa}(i-1)(1-\Phi)\Phi F_{CCBbAa}(i-1)+0.5M_{ccBbaa}(i-1)\Phi\Phi F_{CCBbAa}(i-1)+0.5M_{ccBbaa}(i-1)(1-\Phi)F_{CCBbAA}(i-1)+0.5M_{ccBbaa}(i-1)\Phi F_{CCBbAA}(i-1)+0.25M_{ccBbaa}(i-1)(1-\Phi)F_{CCBBAa}(i-1)+0.5M_{ccBbaa}(i-1)\Phi F_{CCBBAa}(i-1)+0.5M_{ccBbaa}(i-1)F_{CCBBAA}(i-1)+0.125(1-\Phi)M_{ccBbAa}(i-1)F_{Ccbbaa}(i-1)+0.125(1-\Phi)M_{ccBbAa}(i-1)F_{CcbbAa}(i-1)+0.125(1-\Phi)M_{ccBbAa}(i-1)F_{CcbbAA}(i-1)+0.125(1-\Phi)M_{ccBbAa}(i-1)(1-\Phi)F_{CcBbaa}(i-1)+0.125(1-\Phi)M_{ccBbAa}(i-1)\Phi F_{CcBbaa}(i-1)+0.125(1-\Phi)M_{ccBbAa}(i-1)(1-\Phi)(1-\Phi)F_{CcBbAa}(i-1)+0.125(1-\Phi)M_{ccBbAa}(i-1)\Phi(1-\Phi)F_{CcBbAa}(i-1)+0.125(1-\Phi)M_{ccBbAa}(i-1)(1-\Phi)\Phi F_{CcBbAa}(i-1)+0.125(1-\Phi)M_{ccBbAa}(i-1)\Phi\Phi F_{CcBbAa}(i-1)+0.125(1-\Phi)M_{ccBbAa}(i-1)(1-\Phi)F_{CcBbAA}(i-1)+0.125(1-\Phi)M_{ccBbAa}(i-1)\Phi F_{CcBbAA}(i-1)+0.125(1-\Phi)M_{ccBbAa}(i-1)F_{CcBBaa}(i-1)+0.125(1-\Phi)M_{ccBbAa}(i-1)(1-\Phi)F_{CcBBAa}(i-1)+0.125(1-\Phi)M_{ccBbAa}(i-1)\Phi F_{CcBBAa}(i-1)+0.125(1-\Phi)M_{ccBbAa}(i-1)F_{CcBBAA}(i-1)+0.25(1-\Phi)M_{ccBbAa}(i-1)F_{CCbbaa}(i-1)+0.25(1-\Phi)M_{ccBbAa}(i-1)F_{CCbbAa}(i-1)+0.25(1-\Phi)M_{ccBbAa}(i-1)F_{CCbbAA}(i-1)+0.25(1-\Phi)M_{ccBbAa}(i-1)(1-\Phi)F_{CCBbaa}(i-1)+0.25(1-\Phi)M_{ccBbAa}(i-1)\Phi F_{CCBbaa}(i-1)+0.25(1-\Phi)M_{ccBbAa}(i-1)(1-\Phi)(1-\Phi)F_{CCBbAa}(i-1)+0.25(1-\Phi)M_{ccBbAa}(i-1)\Phi(1-\Phi)F_{CCBbAa}(i-1)+0.25(1-\Phi)M_{ccBbAa}(i-1)(1-\Phi)\Phi F_{CCBbAa}(i-1)+0.25(1-\Phi)M_{ccBbAa}(i-1)\Phi\Phi F_{CCBbAa}(i-1)+0.25(1-\Phi)M_{ccBbAa}(i-1)(1-\Phi)F_{CCBbAA}(i-1)+0.25(1-\Phi)M_{ccBbAa}(i-1)\Phi F_{CCBbAA}(i-1)+0.25(1-\Phi)M_{ccBbAa}(i-1)F_{CCBBaa}(i-1)+0.25(1-\Phi)M_{ccBbAa}(i-1)(1-\Phi)F_{CCBBAa}(i-1)+0.25(1-\Phi)M_{ccBbAa}(i-1)\Phi F_{CCBBAa}(i-1)+0.25(1-\Phi)M_{ccBbAa}(i-1)F_{CCBBAA}(i-1)+0.25\Phi M_{ccBbAa}(i-1)F_{Ccbbaa}(i-1)+0.125\Phi M_{ccBbAa}(i-1)F_{CcbbAa}(i-1)+0.25\Phi M_{ccBbAa}(i-1)(1-\Phi)F_{CcBbaa}(i-1)+0.25\Phi M_{ccBbAa}(i-1)\Phi F_{CcBbaa}(i-1)+0.125\Phi M_{ccBbAa}(i-1)(1-\Phi)(1-\Phi)F_{CcBbAa}(i-1)+0.125\Phi M_{ccBbAa}(i-1)\Phi(1-\Phi)F_{CcBbAa}(i-1)+0.25\Phi M_{ccBbAa}(i-1)F_{CcBBaa}(i-1)+0.125\Phi M_{ccBbAa}(i-1)(1-\Phi)F_{CcBBAa}(i-1)+0.5\Phi M_{ccBbAa}(i-1)F_{CCbbaa}(i-1)+0.25\Phi M_{ccBbAa}(i-1)F_{CCbbAa}(i-1)+$$

$$
0.5\Phi M_{ccBbAa}(i-1)(1-\Phi)F_{CCBbaa}(i-1) + 0.5\Phi M_{ccBbAa}(i-1)\Phi F_{CCBbaa}(i-1) + 0.25\Phi M_{ccBbAa}(i-
1)(1-\Phi)(1-\Phi)F_{CCBbAa}(i-1)+0.25\Phi M_{ccBbAa}(i-1)\Phi(1-\Phi)F_{CCBbAa}(i-1)+0.25\Phi M_{ccBbAa}(i-1)(1-
\Phi)\Phi F_{CCBbAa}(i-1) + 0.5\Phi M_{ccBbAa}(i-1)F_{CCBBaa}(i-1) + 0.25\Phi M_{ccBbAa}(i-1)(1-\Phi)F_{CCBBAa}(i-
1) + 0.25M_{ccBbAA}(i-1)F_{Ccbbaa}(i-1) + 0.125M_{ccBbAA}(i-1)F_{CcbbAa}(i-1) + 0.25M_{ccBbAA}(i-1)(1-
\Phi)F_{CcBbaa}(i-1) + 0.25M_{ccBbAA}(i-1)\Phi F_{CcBbaa}(i-1) + 0.125M_{ccBbAA}(i-1)(1-\Phi)(1-\Phi)F_{CcBbAa}(i-
1)+0.125M_{ccBbAA}(i-1)\Phi(1-\Phi)F_{CcBbAa}(i-1)+0.25M_{ccBbAA}(i-1)F_{CcBBaa}(i-1)+0.125M_{ccBbAA}(i-
1)(1-\Phi)F_{CcBBAa}(i-1) + 0.5M_{ccBbAA}(i-1)F_{CCbbaa}(i-1) + 0.25M_{ccBbAA}(i-1)F_{CCbbAa}(i-1) +
0.5M_{ccBbAA}(i-1)(1-\Phi)F_{CCBbaa}(i-1)+0.5M_{ccBbAA}(i-1)\Phi F_{CCBbaa}(i-1)+0.25M_{ccBbAA}(i-1)(1-\Phi)(1-
\Phi)F_{CCBbAa}(i-1)+0.25M_{ccBbAA}(i-1)\Phi(1-\Phi)F_{CCBbAa}(i-1)+0.25M_{ccBbAA}(i-1)(1-\Phi)\Phi F_{CCBbAa}(i-
1) + 0.5M_{ccBbAA}(i-1)F_{CCBBaa}(i-1) + 0.25M_{ccBbAA}(i-1)(1-\Phi)F_{CCBBAa}(i-1) + 0.25M_{ccBBaa}(i-
1)F_{CcbbAa}(i-1) + 0.5M_{ccBBaa}(i-1)F_{CcbbAA}(i-1) + 0.125M_{ccBBaa}(i-1)(1-\Phi)(1-\Phi)F_{CcBbAa}(i-1) +
0.25M_{ccBBaa}(i-1)(1-\Phi)\Phi F_{CcBbAa}(i-1) + 0.25M_{ccBBaa}(i-1)(1-\Phi)F_{CcBbAA}(i-1) + 0.5M_{ccBBaa}(i-
1)F_{CCbbAa}(i-1) + M_{ccBBaa}(i-1)F_{CCbbAA}(i-1) + 0.25M_{ccBBaa}(i-1)(1-\Phi)(1-\Phi)F_{CCBbAa}(i-
1) + 0.5M_{ccBBaa}(i-1)(1-\Phi)F_{CCBbAA}(i-1) + 0.25(1-\Phi)M_{ccBBAa}(i-1)F_{Ccbbaa}(i-1) + 0.25(1-
\Phi)M_{ccBBAa}(i-1)F_{CcbbAa}(i-1) + 0.25(1-\Phi)M_{ccBBAa}(i-1)F_{CcbbAA}(i-1) + 0.125(1-\Phi)M_{ccBBAa}(i-
1)(1-\Phi)F_{CcBbaa}(i-1)+0.125(1-\Phi)M_{ccBBAa}(i-1)(1-\Phi)(1-\Phi)F_{CcBbAa}(i-1)+0.125(1-\Phi)M_{ccBBAa}(i-
1)(1-\Phi)\Phi F_{CcBbAa}(i-1) + 0.125(1-\Phi)M_{ccBBAa}(i-1)(1-\Phi)F_{CcBbAA}(i-1) + 0.5(1-\Phi)M_{ccBBAa}(i-
1)F_{CCbbaa}(i-1) + 0.5(1-\Phi)M_{ccBBAa}(i-1)F_{CCbbAa}(i-1) + 0.5(1-\Phi)M_{ccBBAa}(i-1)F_{CCbbAA}(i-1) +
0.25(1-\Phi)M_{ccBBAa}(i-1)(1-\Phi)F_{CCBbaa}(i-1)+0.25(1-\Phi)M_{ccBBAa}(i-1)(1-\Phi)(1-\Phi)F_{CCBbAa}(i-1)+
0.25(1-\Phi)M_{ccBBAa}(i-1)(1-\Phi)F_{CCBbAA}(i-1)+0.5\Phi M_{ccBBAa}(i-1)F_{Ccbbaa}(i-1)+0.25\Phi M_{ccBBAa}(i-
1)F_{CcbbAa}(i-1) + 0.25\Phi M_{ccBBAa}(i-1)(1-\Phi)F_{CcBbaa}(i-1) + 0.125\Phi M_{ccBBAa}(i-1)(1-\Phi)(1-
\Phi)F_{CcBbAa}(i-1)+\Phi M_{ccBBAa}(i-1)F_{CCbbaa}(i-1)+0.5\Phi M_{ccBBAa}(i-1)F_{CCbbAa}(i-1)+0.5\Phi M_{ccBBAa}(i-
1)(1-\Phi)F_{CCBbaa}(i-1)+0.25\Phi M_{ccBBAa}(i-1)(1-\Phi)(1-\Phi)F_{CCBbAa}(i-1)+0.5M_{ccBBAA}(i-1)F_{Ccbbaa}(i-
1)+0.25M_{ccBBAA}(i-1)F_{CcbbAa}(i-1)+0.25M_{ccBBAA}(i-1)(1-\Phi)F_{CcBbaa}(i-1)+0.125M_{ccBBAA}(i-1)(1-
\Phi)(1-\Phi)F_{CcBbAa}(i-1)+M_{ccBBAA}(i-1)F_{CCbbaa}(i-1)+0.5M_{ccBBAA}(i-1)F_{CCbbAa}(i-1)+0.5M_{ccBBAA}(i-
1)(1-\Phi)F_{CCBbaa}(i-1) + 0.25M_{ccBBAA}(i-1)(1-\Phi)(1-\Phi)F_{CCBbAa}(i-1) + 0.125M_{Ccbbaa}(i-1)(1-
\Phi)F_{ccBbAa}(i-1)+0.25M_{Ccbbaa}(i-1)\Phi F_{ccBbAa}(i-1)+0.25M_{Ccbbaa}(i-1)F_{ccBbAA}(i-1)+0.25M_{Ccbbaa}(i-
1)(1-\Phi)F_{ccBBAa}(i-1) + 0.5M_{Ccbbaa}(i-1)\Phi F_{ccBBAa}(i-1) + 0.5M_{Ccbbaa}(i-1)F_{ccBBAA}(i-1) +
0.125M_{Ccbbaa}(i-1)(1-\Phi)(1-\Phi)F_{CcBbAa}(i-1)+0.25M_{Ccbbaa}(i-1)\Phi(1-\Phi)F_{CcBbAa}(i-1)+0.25M_{Ccbbaa}(i-
1)(1-\Phi)\Phi F_{CcBbAa}(i-1) + 0.5M_{Ccbbaa}(i-1)\Phi\Phi F_{CcBbAa}(i-1) + 0.25M_{Ccbbaa}(i-1)(1-\Phi)F_{CcBbAA}(i-
1) + 0.5M_{Ccbbaa}(i-1)\Phi F_{CcBbAA}(i-1) + 0.25M_{Ccbbaa}(i-1)(1-\Phi)F_{CcBBAa}(i-1) + 0.5M_{Ccbbaa}(i-
1)\Phi F_{CcBBAa}(i-1)+0.5M_{Ccbbaa}(i-1)F_{CcBBAA}(i-1)+0.125M_{Ccbbaa}(i-1)(1-\Phi)(1-\Phi)F_{CCBbAa}(i-1)+
0.25M_{Ccbbaa}(i-1)\Phi(1-\Phi)F_{CCBbAa}(i-1) + 0.25M_{Ccbbaa}(i-1)(1-\Phi)\Phi F_{CCBbAa}(i-1) + 0.5M_{Ccbbaa}(i-
1)\Phi\Phi F_{CCBbAa}(i-1) + 0.25M_{Ccbbaa}(i-1)(1-\Phi)F_{CCBbAA}(i-1) + 0.5M_{Ccbbaa}(i-1)\Phi F_{CCBbAA}(i-
1) + 0.25M_{Ccbbaa}(i-1)(1-\Phi)F_{CCBBAa}(i-1) + 0.5M_{Ccbbaa}(i-1)\Phi F_{CCBBAa}(i-1) + 0.5M_{Ccbbaa}(i-
1)F_{CCBBAA}(i-1) + 0.125M_{CcbbAa}(i-1)F_{ccBbaa}(i-1) + 0.125M_{CcbbAa}(i-1)(1-\Phi)F_{ccBbAa}(i-1) +
0.125M_{CcbbAa}(i-1)\Phi F_{ccBbAa}(i-1)+0.125M_{CcbbAa}(i-1)F_{ccBbAA}(i-1)+0.25M_{CcbbAa}(i-1)F_{ccBBaa}(i-
1) + 0.25M_{CcbbAa}(i-1)(1-\Phi)F_{ccBBAa}(i-1) + 0.25M_{CcbbAa}(i-1)\Phi F_{ccBBAa}(i-1) + 0.25M_{CcbbAa}(i-
1)F_{ccBBAA}(i-1) + 0.125M_{CcbbAa}(i-1)(1-\Phi)F_{CcBbaa}(i-1) + 0.25M_{CcbbAa}(i-1)\Phi F_{CcBbaa}(i-1) +
0.125M_{CcbbAa}(i-1)(1-\Phi)(1-\Phi)F_{CcBbAa}(i-1)+0.25M_{CcbbAa}(i-1)\Phi(1-\Phi)F_{CcBbAa}(i-1)+0.125M_{CcbbAa}(i-
1)(1-\Phi)\Phi F_{CcBbAa}(i-1)+0.25M_{CcbbAa}(i-1)\Phi\Phi F_{CcBbAa}(i-1)+0.125M_{CcbbAa}(i-1)(1-\Phi)F_{CcBbAA}(i-
1) + 0.25M_{CcbbAa}(i-1)\Phi F_{CcBbAA}(i-1) + 0.25M_{CcbbAa}(i-1)F_{CcBBaa}(i-1) + 0.25M_{CcbbAa}(i-1)(1-
\Phi)F_{CcBBAa}(i-1)+0.25M_{CcbbAa}(i-1)\Phi F_{CcBBAa}(i-1)+0.25M_{CcbbAa}(i-1)F_{CcBBAA}(i-1)+0.125M_{CcbbAa}(i-
1)(1-\Phi)F_{CCBbaa}(i-1)+0.25M_{CcbbAa}(i-1)\Phi F_{CCBbaa}(i-1)+0.125M_{CcbbAa}(i-1)(1-\Phi)(1-\Phi)F_{CCBbAa}(i-
1)+0.25M_{CcbbAa}(i-1)\Phi(1-\Phi)F_{CCBbAa}(i-1)+0.25M_{CcbbAa}(i-1)(1-\Phi)\Phi F_{CCBbAa}(i-1)+0.25M_{CcbbAa}(i-
1)\Phi\Phi F_{CCBbAa}(i-1) + 0.125M_{CcbbAa}(i-1)(1-\Phi)F_{CCBbAA}(i-1) + 0.25M_{CcbbAa}(i-1)\Phi F_{CCBbAA}(i-
1) + 0.25M_{CcbbAa}(i-1)F_{CCBBaa}(i-1) + 0.25M_{CcbbAa}(i-1)(1-\Phi)F_{CCBBAa}(i-1) + 0.25M_{CcbbAa}(i-
1)\Phi F_{CCBBAa}(i-1)+0.25M_{CcbbAa}(i-1)F_{CCBBAA}(i-1)+0.25M_{CcbbAA}(i-1)F_{ccBbaa}(i-1)+0.125M_{CcbbAA}(i-
1)(1-\Phi)F_{ccBbAa}(i-1) + 0.5M_{CcbbAA}(i-1)F_{ccBBaa}(i-1) + 0.25M_{CcbbAA}(i-1)(1-\Phi)F_{ccBBAa}(i-1) +
0.25M_{CcbbAA}(i-1)(1-\Phi)F_{CcBbaa}(i-1) + 0.5M_{CcbbAA}(i-1)\Phi F_{CcBbaa}(i-1) + 0.125M_{CcbbAA}(i-1)(1-
\Phi)(1-\Phi)F_{CcBbAa}(i-1)+0.25M_{CcbbAA}(i-1)\Phi(1-\Phi)F_{CcBbAa}(i-1)+0.5M_{CcbbAA}(i-1)F_{CcBBaa}(i-1)+
0.25M_{CcbbAA}(i-1)(1-\Phi)F_{CcBBAa}(i-1) + 0.25M_{CcbbAA}(i-1)(1-\Phi)F_{CCBbaa}(i-1) + 0.5M_{CcbbAA}(i-
1)\Phi F_{CCBbaa}(i-1) + 0.125M_{CcbbAA}(i-1)(1-\Phi)(1-\Phi)F_{CCBbAa}(i-1) + 0.25M_{CcbbAA}(i-1)\Phi(1-
\Phi)F_{CCBbAa}(i-1) + 0.25M_{CcbbAA}(i-1)(1-\Phi)\Phi F_{CCBbAa}(i-1) + 0.5M_{CcbbAA}(i-1)F_{CCBBaa}(i-1) +
0.25M_{CcbbAA}(i-1)(1-\Phi)F_{CCBBAa}(i-1)+0.125(1-\Phi)M_{CcBbaa}(i-1)F_{ccbbAa}(i-1)+0.25(1-\Phi)M_{CcBbaa}(i-
1)F_{ccbbAA}(i-1)+0.125(1-\Phi)M_{CcBbaa}(i-1)(1-\Phi)F_{ccBbAa}(i-1)+0.25(1-\Phi)M_{CcBbaa}(i-1)\Phi F_{ccBbAa}(i-
1) + 0.25(1-\Phi)M_{CcBbaa}(i-1)F_{ccBbAA}(i-1) + 0.125(1-\Phi)M_{CcBbaa}(i-1)(1-\Phi)F_{ccBBAa}(i-1) +
$$

$0.25(1-\Phi)M_{CcBbaa}(i-1)\Phi F_{ccBBAa}(i-1)+0.25(1-\Phi)M_{CcBbaa}(i-1)F_{ccBBAA}(i-1)+0.125(1-$
$\Phi)M_{CcBbaa}(i-1)F_{CcbbAa}(i-1)+0.25(1-\Phi)M_{CcBbaa}(i-1)F_{CcbbAA}(i-1)+0.125(1-\Phi)M_{CcBbaa}(i-1)(1-$
$\Phi)(1-\Phi)F_{CcBbAa}(i-1)+0.125(1-\Phi)M_{CcBbaa}(i-1)\Phi(1-\Phi)F_{CcBbAa}(i-1)+0.25(1-\Phi)M_{CcBbaa}(i-$
$1)(1-\Phi)\Phi F_{CcBbAa}(i-1)+0.25(1-\Phi)M_{CcBbaa}(i-1)\Phi\Phi F_{CcBbAa}(i-1)+0.25(1-\Phi)M_{CcBbaa}(i-$
$1)(1-\Phi)F_{CcBbAA}(i-1)+0.25(1-\Phi)M_{CcBbaa}(i-1)\Phi F_{CcBbAA}(i-1)+0.125(1-\Phi)M_{CcBbaa}(i-1)(1-$
$\Phi)F_{CcBBAa}(i-1)+0.25(1-\Phi)M_{CcBbaa}(i-1)\Phi F_{CcBBAa}(i-1)+0.25(1-\Phi)M_{CcBbaa}(i-1)F_{CcBBAA}(i-$
$1)+0.125(1-\Phi)M_{CcBbaa}(i-1)F_{CCbbAa}(i-1)+0.25(1-\Phi)M_{CcBbaa}(i-1)F_{CCbbAA}(i-1)+0.125(1-$
$\Phi)M_{CcBbaa}(i-1)(1-\Phi)(1-\Phi)F_{CCBbAa}(i-1)+0.125(1-\Phi)M_{CcBbaa}(i-1)\Phi(1-\Phi)F_{CCBbAa}(i-$
$1)+0.125(1-\Phi)M_{CcBbaa}(i-1)(1-\Phi)\Phi F_{CCBbAa}(i-1)+0.25(1-\Phi)M_{CcBbaa}(i-1)\Phi\Phi F_{CCBbAa}(i-$
$1)+0.25(1-\Phi)M_{CcBbaa}(i-1)(1-\Phi)F_{CCBbAA}(i-1)+0.25(1-\Phi)M_{CcBbaa}(i-1)\Phi F_{CCBbAA}(i-1)+$
$0.125(1-\Phi)M_{CcBbaa}(i-1)(1-\Phi)F_{CCBBAa}(i-1)+0.25(1-\Phi)M_{CcBbaa}(i-1)\Phi F_{CCBBAa}(i-1)+$
$0.25(1-\Phi)M_{CcBbaa}(i-1)F_{CCBBAA}(i-1)+0.25\Phi M_{CcBbaa}(i-1)F_{ccbbAa}(i-1)+0.5\Phi M_{CcBbaa}(i-$
$1)F_{ccbbAA}(i-1)+0.125\Phi M_{CcBbaa}(i-1)(1-\Phi)F_{ccBbAa}(i-1)+0.25\Phi M_{CcBbaa}(i-1)\Phi F_{ccBbAa}(i-1)+$
$0.25\Phi M_{CcBbaa}(i-1)F_{ccBbAA}(i-1)+0.25\Phi M_{CcBbaa}(i-1)F_{CcbbAa}(i-1)+0.5\Phi M_{CcBbaa}(i-1)F_{CcbbAA}(i-$
$1)+0.125\Phi M_{CcBbaa}(i-1)(1-\Phi)(1-\Phi)F_{CcBbAa}(i-1)+0.25\Phi M_{CcBbaa}(i-1)(1-\Phi)\Phi F_{CcBbAa}(i-1)+$
$0.25\Phi M_{CcBbaa}(i-1)(1-\Phi)F_{CcBbAA}(i-1)+0.25\Phi M_{CcBbaa}(i-1)F_{CCbbAa}(i-1)+0.5\Phi M_{CcBbaa}(i-$
$1)F_{CCbbAA}(i-1)+0.125\Phi M_{CcBbaa}(i-1)(1-\Phi)(1-\Phi)F_{CCBbAa}(i-1)+0.25\Phi M_{CcBbaa}(i-1)(1-$
$\Phi)F_{CCBbAA}(i-1)+0.125(1-\Phi)(1-\Phi)M_{CcBbAa}(i-1)F_{ccbbaa}(i-1)+0.125(1-\Phi)(1-\Phi)M_{CcBbAa}(i-$
$1)F_{ccbbAa}(i-1)+0.125(1-\Phi)(1-\Phi)M_{CcBbAa}(i-1)F_{ccbbAA}(i-1)+0.125(1-\Phi)(1-\Phi)M_{CcBbAa}(i-$
$1)F_{ccBbaa}(i-1)+0.125(1-\Phi)(1-\Phi)M_{CcBbAa}(i-1)(1-\Phi)F_{ccBbAa}(i-1)+0.125(1-\Phi)(1-\Phi)M_{CcBbAa}(i-$
$1)\Phi F_{ccBbAa}(i-1)+0.125(1-\Phi)(1-\Phi)M_{CcBbAa}(i-1)F_{ccBbAA}(i-1)+0.125(1-\Phi)(1-\Phi)M_{CcBbAa}(i-$
$1)F_{ccBBaa}(i-1)+0.125(1-\Phi)(1-\Phi)M_{CcBbAa}(i-1)(1-\Phi)F_{ccBBAa}(i-1)+0.125(1-\Phi)(1-\Phi)M_{CcBbAa}(i-$
$1)\Phi F_{ccBBAa}(i-1)+0.125(1-\Phi)(1-\Phi)M_{CcBbAa}(i-1)F_{ccBBAA}(i-1)+0.125(1-\Phi)(1-\Phi)M_{CcBbAa}(i-$
$1)F_{Ccbbaa}(i-1)+0.125(1-\Phi)(1-\Phi)M_{CcBbAa}(i-1)F_{CcbbAa}(i-1)+0.125(1-\Phi)(1-\Phi)M_{CcBbAa}(i-$
$1)F_{CcbbAA}(i-1)+0.125(1-\Phi)(1-\Phi)M_{CcBbAa}(i-1)(1-\Phi)F_{CcBbaa}(i-1)+0.125(1-\Phi)(1-\Phi)M_{CcBbAa}(i-$
$1)\Phi F_{CcBbaa}(i-1)+0.125(1-\Phi)(1-\Phi)M_{CcBbAa}(i-1)(1-\Phi)(1-\Phi)F_{CcBbAa}(i-1)+0.125(1-\Phi)(1-$
$\Phi)M_{CcBbAa}(i-1)\Phi(1-\Phi)F_{CcBbAa}(i-1)+0.125(1-\Phi)(1-\Phi)M_{CcBbAa}(i-1)(1-\Phi)\Phi F_{CcBbAa}(i-1)+$
$0.125(1-\Phi)(1-\Phi)M_{CcBbAa}(i-1)\Phi\Phi F_{CcBbAa}(i-1)+0.125(1-\Phi)(1-\Phi)M_{CcBbAa}(i-1)(1-\Phi)F_{CcBbAA}(i-$
$1)+0.125(1-\Phi)(1-\Phi)M_{CcBbAa}(i-1)\Phi F_{CcBbAA}(i-1)+0.125(1-\Phi)(1-\Phi)M_{CcBbAa}(i-1)F_{CcBBaa}(i-1)+$
$0.125(1-\Phi)(1-\Phi)M_{CcBbAa}(i-1)(1-\Phi)F_{CcBBAa}(i-1)+0.125(1-\Phi)(1-\Phi)M_{CcBbAa}(i-1)\Phi F_{CcBBAa}(i-$
$1)+0.125(1-\Phi)(1-\Phi)M_{CcBbAa}(i-1)F_{CcBBAA}(i-1)+0.125(1-\Phi)(1-\Phi)M_{CcBbAa}(i-1)F_{CCbbaa}(i-$
$1)+0.125(1-\Phi)(1-\Phi)M_{CcBbAa}(i-1)F_{CCbbAa}(i-1)+0.125(1-\Phi)(1-\Phi)M_{CcBbAa}(i-1)F_{CCbbAA}(i-1)+$
$0.125(1-\Phi)(1-\Phi)M_{CcBbAa}(i-1)(1-\Phi)F_{CCBbaa}(i-1)+0.125(1-\Phi)(1-\Phi)M_{CcBbAa}(i-1)\Phi F_{CCBbaa}(i-$
$1)+0.125(1-\Phi)(1-\Phi)M_{CcBbAa}(i-1)(1-\Phi)(1-\Phi)F_{CCBbAa}(i-1)+0.125(1-\Phi)(1-\Phi)M_{CcBbAa}(i-$
$1)\Phi(1-\Phi)F_{CCBbAa}(i-1)+0.125(1-\Phi)(1-\Phi)M_{CcBbAa}(i-1)(1-\Phi)\Phi F_{CCBbAa}(i-1)+0.125(1-\Phi)(1-$
$\Phi)M_{CcBbAa}(i-1)\Phi\Phi F_{CCBbAa}(i-1)+0.125(1-\Phi)(1-\Phi)M_{CcBbAa}(i-1)(1-\Phi)F_{CCBbAA}(i-1)+0.125(1-$
$\Phi)(1-\Phi)M_{CcBbAa}(i-1)\Phi F_{CCBbAA}(i-1)+0.125(1-\Phi)(1-\Phi)M_{CcBbAa}(i-1)F_{CCBBaa}(i-1)+0.125(1-$
$\Phi)(1-\Phi)M_{CcBbAa}(i-1)(1-\Phi)F_{CCBBAa}(i-1)+0.125(1-\Phi)(1-\Phi)M_{CcBbAa}(i-1)\Phi F_{CCBBAa}(i-$
$1)+0.125(1-\Phi)(1-\Phi)M_{CcBbAa}(i-1)F_{CCBBAA}(i-1)+0.25\Phi(1-\Phi)M_{CcBbAa}(i-1)F_{ccbbaa}(i-1)+$
$0.25\Phi(1-\Phi)M_{CcBbAa}(i-1)F_{ccbbAa}(i-1)+0.25\Phi(1-\Phi)M_{CcBbAa}(i-1)F_{ccbbAA}(i-1)+0.125\Phi(1-$
$\Phi)M_{CcBbAa}(i-1)F_{ccBbaa}(i-1)+0.125\Phi(1-\Phi)M_{CcBbAa}(i-1)(1-\Phi)F_{ccBbAa}(i-1)+0.125\Phi(1-$
$\Phi)M_{CcBbAa}(i-1)\Phi F_{ccBbAa}(i-1)+0.125\Phi(1-\Phi)M_{CcBbAa}(i-1)F_{ccBbAA}(i-1)+0.25\Phi(1-\Phi)M_{CcBbAa}(i-$
$1)F_{Ccbbaa}(i-1)+0.25\Phi(1-\Phi)M_{CcBbAa}(i-1)F_{CcbbAa}(i-1)+0.25\Phi(1-\Phi)M_{CcBbAa}(i-1)F_{CcbbAA}(i-$
$1)+0.125\Phi(1-\Phi)M_{CcBbAa}(i-1)(1-\Phi)F_{CcBbaa}(i-1)+0.125\Phi(1-\Phi)M_{CcBbAa}(i-1)(1-\Phi)(1-$
$\Phi)F_{CcBbAa}(i-1)+0.125\Phi(1-\Phi)M_{CcBbAa}(i-1)(1-\Phi)\Phi F_{CcBbAa}(i-1)+0.125\Phi(1-\Phi)M_{CcBbAa}(i-1)(1-$
$\Phi)F_{CcBbAA}(i-1)+0.25\Phi(1-\Phi)M_{CcBbAa}(i-1)F_{CCbbaa}(i-1)+0.25\Phi(1-\Phi)M_{CcBbAa}(i-1)F_{CCbbAa}(i-$
$1)+0.25\Phi(1-\Phi)M_{CcBbAa}(i-1)F_{CCbbAA}(i-1)+0.125\Phi(1-\Phi)M_{CcBbAa}(i-1)(1-\Phi)F_{CCBbaa}(i-$
$1)+0.125\Phi(1-\Phi)M_{CcBbAa}(i-1)(1-\Phi)(1-\Phi)F_{CCBbAa}(i-1)+0.125\Phi(1-\Phi)M_{CcBbAa}(i-1)(1-$
$\Phi)F_{CCBbAA}(i-1)+0.25(1-\Phi)\Phi M_{CcBbAa}(i-1)F_{ccbbaa}(i-1)+0.125(1-\Phi)\Phi M_{CcBbAa}(i-1)F_{ccbbAa}(i-$
$1)+0.25(1-\Phi)\Phi M_{CcBbAa}(i-1)F_{ccBbaa}(i-1)+0.125(1-\Phi)\Phi M_{CcBbAa}(i-1)(1-\Phi)F_{ccBbAa}(i-1)+$
$0.25(1-\Phi)\Phi M_{CcBbAa}(i-1)F_{ccBBaa}(i-1)+0.125(1-\Phi)\Phi M_{CcBbAa}(i-1)(1-\Phi)F_{ccBBAa}(i-1)+$
$0.25(1-\Phi)\Phi M_{CcBbAa}(i-1)F_{Ccbbaa}(i-1)+0.125(1-\Phi)\Phi M_{CcBbAa}(i-1)F_{CcbbAa}(i-1)+0.25(1-$
$\Phi)\Phi M_{CcBbAa}(i-1)(1-\Phi)F_{CcBbaa}(i-1)+0.25(1-\Phi)\Phi M_{CcBbAa}(i-1)\Phi F_{CcBbaa}(i-1)+0.125(1-$
$\Phi)\Phi M_{CcBbAa}(i-1)(1-\Phi)(1-\Phi)F_{CcBbAa}(i-1)+0.125(1-\Phi)\Phi M_{CcBbAa}(i-1)\Phi(1-\Phi)F_{CcBbAa}(i-1)+$
$0.25(1-\Phi)\Phi M_{CcBbAa}(i-1)F_{CcBBaa}(i-1)+0.125(1-\Phi)\Phi M_{CcBbAa}(i-1)(1-\Phi)F_{CcBBAa}(i-1)+0.25(1-$
$\Phi)\Phi M_{CcBbAa}(i-1)F_{CCbbaa}(i-1)+0.125(1-\Phi)\Phi M_{CcBbAa}(i-1)F_{CCbbAa}(i-1)+0.25(1-\Phi)\Phi M_{CcBbAa}(i-$
$1)(1-\Phi)F_{CCBbaa}(i-1)+0.25(1-\Phi)\Phi M_{CcBbAa}(i-1)\Phi F_{CCBbaa}(i-1)+0.125(1-\Phi)\Phi M_{CcBbAa}(i-$

$1)(1-\Phi)(1-\Phi)F_{CCBbAa}(i-1)+0.125(1-\Phi)\Phi M_{CcBbAa}(i-1)\Phi(1-\Phi)F_{CCBbAa}(i-1)+0.125(1-$
$\Phi)\Phi M_{CcBbAa}(i-1)(1-\Phi)\Phi F_{CCBbAa}(i-1)+0.25(1-\Phi)\Phi M_{CcBbAa}(i-1)F_{CCBBaa}(i-1)+0.125(1-$
$\Phi)\Phi M_{CcBbAa}(i-1)(1-\Phi)F_{CCBBAa}(i-1)+0.5\Phi\Phi M_{CcBbAa}(i-1)F_{ccbbaa}(i-1)+0.25\Phi\Phi M_{CcBbAa}(i-$
$1)F_{ccbbAa}(i-1)+0.25\Phi\Phi M_{CcBbAa}(i-1)F_{ccBbaa}(i-1)+0.125\Phi\Phi M_{CcBbAa}(i-1)(1-\Phi)F_{ccBbAa}(i-$
$1)+0.5\Phi\Phi M_{CcBbAa}(i-1)F_{Ccbbaa}(i-1)+0.25\Phi\Phi M_{CcBbAa}(i-1)F_{CcbbAa}(i-1)+0.25\Phi\Phi M_{CcBbAa}(i-$
$1)(1-\Phi)F_{CcBbaa}(i-1)+0.125\Phi\Phi M_{CcBbAa}(i-1)(1-\Phi)(1-\Phi)F_{CcBbAa}(i-1)+0.5\Phi\Phi M_{CcBbAa}(i-$
$1)F_{CCbbaa}(i-1)+0.25\Phi\Phi M_{CcBbAa}(i-1)F_{CCbbAa}(i-1)+0.25\Phi\Phi M_{CcBbAa}(i-1)(1-\Phi)F_{CCBbaa}(i-1)+$
$0.125\Phi\Phi M_{CcBbAa}(i-1)(1-\Phi)(1-\Phi)F_{CCBbAa}(i-1)+0.25(1-\Phi)M_{CcBbAA}(i-1)F_{ccbbaa}(i-1)+0.125(1-$
$\Phi)M_{CcBbAA}(i-1)F_{ccbbAa}(i-1)+0.25(1-\Phi)M_{CcBbAA}(i-1)F_{ccBbaa}(i-1)+0.125(1-\Phi)M_{CcBbAA}(i-$
$1)(1-\Phi)F_{ccBbAa}(i-1)+0.25(1-\Phi)M_{CcBbAA}(i-1)F_{ccBBaa}(i-1)+0.125(1-\Phi)M_{CcBbAA}(i-1)(1-$
$\Phi)F_{ccBBAa}(i-1)+0.25(1-\Phi)M_{CcBbAA}(i-1)F_{Ccbbaa}(i-1)+0.125(1-\Phi)M_{CcBbAA}(i-1)F_{CcbbAa}(i-1)+$
$0.25(1-\Phi)M_{CcBbAA}(i-1)(1-\Phi)F_{CcBbaa}(i-1)+0.25(1-\Phi)M_{CcBbAA}(i-1)\Phi F_{CcBbaa}(i-1)+0.125(1-$
$\Phi)M_{CcBbAA}(i-1)(1-\Phi)(1-\Phi)F_{CcBbAa}(i-1)+0.125(1-\Phi)M_{CcBbAA}(i-1)\Phi(1-\Phi)F_{CcBbAa}(i-1)+$
$0.25(1-\Phi)M_{CcBbAA}(i-1)F_{CcBBaa}(i-1)+0.125(1-\Phi)M_{CcBbAA}(i-1)(1-\Phi)F_{CcBBAa}(i-1)+0.25(1-$
$\Phi)M_{CcBbAA}(i-1)F_{CCbbaa}(i-1)+0.125(1-\Phi)M_{CcBbAA}(i-1)F_{CCbbAa}(i-1)+0.25(1-\Phi)M_{CcBbAA}(i-$
$1)(1-\Phi)F_{CCBbaa}(i-1)+0.25(1-\Phi)M_{CcBbAA}(i-1)\Phi F_{CCBbaa}(i-1)+0.125(1-\Phi)M_{CcBbAA}(i-1)(1-\Phi)(1-$
$\Phi)F_{CCBbAa}(i-1)+0.125(1-\Phi)M_{CcBbAA}(i-1)\Phi(1-\Phi)F_{CCBbAa}(i-1)+0.125(1-\Phi)M_{CcBbAA}(i-$
$1)(1-\Phi)\Phi F_{CCBbAa}(i-1)+0.25(1-\Phi)M_{CcBbAA}(i-1)F_{CCBBaa}(i-1)+0.125(1-\Phi)M_{CcBbAA}(i-$
$1)(1-\Phi)F_{CCBBAa}(i-1)+0.5\Phi M_{CcBbAA}(i-1)F_{ccbbaa}(i-1)+0.25\Phi M_{CcBbAA}(i-1)F_{ccbbAa}(i-1)+$
$0.25\Phi M_{CcBbAA}(i-1)F_{ccBbaa}(i-1)+0.125\Phi M_{CcBbAA}(i-1)(1-\Phi)F_{ccBbAa}(i-1)+0.5\Phi M_{CcBbAA}(i-$
$1)F_{Ccbbaa}(i-1)+0.25\Phi M_{CcBbAA}(i-1)F_{CcbbAa}(i-1)+0.25\Phi M_{CcBbAA}(i-1)(1-\Phi)F_{CcBbaa}(i-1)+$
$0.125\Phi M_{CcBbAA}(i-1)(1-\Phi)(1-\Phi)F_{CcBbAa}(i-1)+0.5\Phi M_{CcBbAA}(i-1)F_{CCbbaa}(i-1)+0.25\Phi M_{CcBbAA}(i-$
$1)F_{CCbbAa}(i-1)+0.25\Phi M_{CcBbAA}(i-1)(1-\Phi)F_{CCBbaa}(i-1)+0.125\Phi M_{CcBbAA}(i-1)(1-\Phi)(1-$
$\Phi)F_{CCBbAa}(i-1)+0.25M_{CcBBaa}(i-1)F_{ccbbAa}(i-1)+0.5M_{CcBBaa}(i-1)F_{ccbbAA}(i-1)+0.125M_{CcBBaa}(i-$
$1)(1-\Phi)F_{ccBbAa}(i-1)+0.25M_{CcBBaa}(i-1)\Phi F_{ccBbAa}(i-1)+0.25M_{CcBBaa}(i-1)F_{ccBbAA}(i-1)+$
$0.25M_{CcBBaa}(i-1)F_{CcbbAa}(i-1)+0.5M_{CcBBaa}(i-1)F_{CcbbAA}(i-1)+0.125M_{CcBBaa}(i-1)(1-\Phi)(1-$
$\Phi)F_{CcBbAa}(i-1)+0.25M_{CcBBaa}(i-1)(1-\Phi)\Phi F_{CcBbAa}(i-1)+0.25M_{CcBBaa}(i-1)(1-\Phi)F_{CcBbAA}(i-$
$1)+0.25M_{CcBBaa}(i-1)F_{CCbbAa}(i-1)+0.5M_{CcBBaa}(i-1)F_{CCbbAA}(i-1)+0.125M_{CcBBaa}(i-1)(1-$
$\Phi)(1-\Phi)F_{CCBbAa}(i-1)+0.25M_{CcBBaa}(i-1)(1-\Phi)F_{CCBbAA}(i-1)+0.25(1-\Phi)M_{CcBBAa}(i-$
$1)F_{ccbbaa}(i-1)+0.25(1-\Phi)M_{CcBBAa}(i-1)F_{ccbbAa}(i-1)+0.25(1-\Phi)M_{CcBBAa}(i-1)F_{ccbbAA}(i-$
$1)+0.125(1-\Phi)M_{CcBBAa}(i-1)F_{ccBbaa}(i-1)+0.125(1-\Phi)M_{CcBBAa}(i-1)(1-\Phi)F_{ccBbAa}(i-1)+$
$0.125(1-\Phi)M_{CcBBAa}(i-1)\Phi F_{ccBbAa}(i-1)+0.125(1-\Phi)M_{CcBBAa}(i-1)F_{ccBbAA}(i-1)+0.25(1-$
$\Phi)M_{CcBBAa}(i-1)F_{Ccbbaa}(i-1)+0.25(1-\Phi)M_{CcBBAa}(i-1)F_{CcbbAa}(i-1)+0.25(1-\Phi)M_{CcBBAa}(i-$
$1)F_{CcbbAA}(i-1)+0.125(1-\Phi)M_{CcBBAa}(i-1)(1-\Phi)F_{CcBbaa}(i-1)+0.125(1-\Phi)M_{CcBBAa}(i-1)(1-\Phi)(1-$
$\Phi)F_{CcBbAa}(i-1)+0.125(1-\Phi)M_{CcBBAa}(i-1)(1-\Phi)\Phi F_{CcBbAa}(i-1)+0.125(1-\Phi)M_{CcBBAa}(i-1)(1-$
$\Phi)F_{CcBbAA}(i-1)+0.25(1-\Phi)M_{CcBBAa}(i-1)F_{CCbbaa}(i-1)+0.25(1-\Phi)M_{CcBBAa}(i-1)F_{CCbbAa}(i-1)+$
$0.25(1-\Phi)M_{CcBBAa}(i-1)F_{CCbbAA}(i-1)+0.125(1-\Phi)M_{CcBBAa}(i-1)(1-\Phi)F_{CCBbaa}(i-1)+0.125(1-$
$\Phi)M_{CcBBAa}(i-1)(1-\Phi)(1-\Phi)F_{CCBbAa}(i-1)+0.125(1-\Phi)M_{CcBBAa}(i-1)(1-\Phi)F_{CCBbAA}(i-1)+$
$0.5\Phi M_{CcBBAa}(i-1)F_{ccbbaa}(i-1)+0.25\Phi M_{CcBBAa}(i-1)F_{ccbbAa}(i-1)+0.25\Phi M_{CcBBAa}(i-1)F_{ccBbaa}(i-$
$1)+0.125\Phi M_{CcBBAa}(i-1)(1-\Phi)F_{ccBbAa}(i-1)+0.5\Phi M_{CcBBAa}(i-1)F_{Ccbbaa}(i-1)+0.25\Phi M_{CcBBAa}(i-$
$1)F_{CcbbAa}(i-1)+0.25\Phi M_{CcBBAa}(i-1)(1-\Phi)F_{CcBbaa}(i-1)+0.125\Phi M_{CcBBAa}(i-1)(1-\Phi)(1-$
$\Phi)F_{CcBbAa}(i-1)+0.5\Phi M_{CcBBAa}(i-1)F_{CCbbaa}(i-1)+0.25\Phi M_{CcBBAa}(i-1)F_{CCbbAa}(i-1)+0.25\Phi M_{CcBBAa}(i-$
$1)(1-\Phi)F_{CCBbaa}(i-1)+0.125\Phi M_{CcBBAa}(i-1)(1-\Phi)(1-\Phi)F_{CCBbAa}(i-1)+0.5M_{CcBBAA}(i-$
$1)F_{ccbbaa}(i-1)+0.25M_{CcBBAA}(i-1)F_{ccbbAa}(i-1)+0.25M_{CcBBAA}(i-1)F_{ccBbaa}(i-1)+0.125M_{CcBBAA}(i-$
$1)(1-\Phi)F_{ccBbAa}(i-1)+0.5M_{CcBBAA}(i-1)F_{Ccbbaa}(i-1)+0.25M_{CcBBAA}(i-1)F_{CcbbAa}(i-1)+$
$0.25M_{CcBBAA}(i-1)(1-\Phi)F_{CcBbaa}(i-1)+0.125M_{CcBBAA}(i-1)(1-\Phi)(1-\Phi)F_{CcBbAa}(i-1)+$
$0.5M_{CcBBAA}(i-1)F_{CCbbaa}(i-1)+0.25M_{CcBBAA}(i-1)F_{CCbbAa}(i-1)+0.25M_{CcBBAA}(i-1)(1-$
$\Phi)F_{CCBbaa}(i-1)+0.125M_{CcBBAA}(i-1)(1-\Phi)(1-\Phi)F_{CCBbAa}(i-1)+0.25M_{CCbbaa}(i-1)(1-\Phi)F_{ccBbAa}(i-$
$1)+0.5M_{CCbbaa}(i-1)\Phi F_{ccBbAa}(i-1)+0.5M_{CCbbaa}(i-1)F_{ccBbAA}(i-1)+0.5M_{CCbbaa}(i-1)(1-$
$\Phi)F_{ccBBAa}(i-1)+M_{CCbbaa}(i-1)\Phi F_{ccBBAa}(i-1)+M_{CCbbaa}(i-1)F_{ccBBAA}(i-1)+0.125M_{CCbbaa}(i-$
$1)(1-\Phi)(1-\Phi)F_{CcBbAa}(i-1)+0.25M_{CCbbaa}(i-1)\Phi(1-\Phi)F_{CcBbAa}(i-1)+0.25M_{CCbbaa}(i-1)(1-$
$\Phi)\Phi F_{CcBbAa}(i-1)+0.5M_{CCbbaa}(i-1)\Phi\Phi F_{CcBbAa}(i-1)+0.25M_{CCbbaa}(i-1)(1-\Phi)F_{CcBbAA}(i-$
$1)+0.5M_{CCbbaa}(i-1)\Phi F_{CcBbAA}(i-1)+0.25M_{CCbbaa}(i-1)(1-\Phi)F_{CcBBAa}(i-1)+0.5M_{CCbbaa}(i-$
$1)\Phi F_{CcBBAa}(i-1)+0.5M_{CCbbaa}(i-1)F_{CcBBAA}(i-1)+0.25M_{CCbbAa}(i-1)F_{ccBbaa}(i-1)+0.25M_{CCbbAa}(i-$
$1)(1-\Phi)F_{ccBbAa}(i-1)+0.25M_{CCbbAa}(i-1)\Phi F_{ccBbAa}(i-1)+0.25M_{CCbbAa}(i-1)F_{ccBbAA}(i-1)+$
$0.5M_{CCbbAa}(i-1)F_{ccBBaa}(i-1)+0.5M_{CCbbAa}(i-1)(1-\Phi)F_{ccBBAa}(i-1)+0.5M_{CCbbAa}(i-1)\Phi F_{ccBBAa}(i-$
$1)+0.5M_{CCbbAa}(i-1)F_{ccBBAA}(i-1)+0.125M_{CCbbAa}(i-1)(1-\Phi)F_{CcBbaa}(i-1)+0.25M_{CCbbAa}(i-$

$1)\Phi F_{CcBbaa}(i-1)+0.125M_{CCbbAa}(i-1)(1-\Phi)(1-\Phi)F_{CcBbAa}(i-1)+0.25M_{CCbbAa}(i-1)\Phi(1-$
$\Phi)F_{CcBbAa}(i-1)+0.125M_{CCbbAa}(i-1)(1-\Phi)\Phi F_{CcBbAa}(i-1)+0.25M_{CCbbAa}(i-1)\Phi\Phi F_{CcBbAa}(i-$
$1)+0.125M_{CCbbAa}(i-1)(1-\Phi)F_{CcBbAA}(i-1)+0.25M_{CCbbAa}(i-1)\Phi F_{CcBbAA}(i-1)+0.25M_{CCbbAa}(i-$
$1)F_{CcBBaa}(i-1)+0.25M_{CCbbAa}(i-1)(1-\Phi)F_{CcBBAa}(i-1)+0.25M_{CCbbAa}(i-1)\Phi F_{CcBBAa}(i-1)+$
$0.25M_{CCbbAa}(i-1)F_{CcBBAA}(i-1)+0.5M_{CCbbAA}(i-1)F_{ccBbaa}(i-1)+0.25M_{CCbbAA}(i-1)(1-\Phi)F_{ccBbAa}(i-$
$1)+M_{CCbbAA}(i-1)F_{ccBBaa}(i-1)+0.5M_{CCbbAA}(i-1)(1-\Phi)F_{ccBBAa}(i-1)+0.25M_{CCbbAA}(i-1)(1-$
$\Phi)F_{CcBbaa}(i-1)+0.5M_{CCbbAA}(i-1)\Phi F_{CcBbaa}(i-1)+0.125M_{CCbbAA}(i-1)(1-\Phi)(1-\Phi)F_{CcBbAa}(i-$
$1)+0.25M_{CCbbAA}(i-1)\Phi(1-\Phi)F_{CcBbAa}(i-1)+0.5M_{CCbbAA}(i-1)F_{CcBBaa}(i-1)+0.25M_{CCbbAA}(i-$
$1)(1-\Phi)F_{CcBBAa}(i-1)+0.25(1-\Phi)M_{CCBbaa}(i-1)F_{ccbbAa}(i-1)+0.5(1-\Phi)M_{CCBbaa}(i-1)F_{ccbbAA}(i-$
$1)+0.25(1-\Phi)M_{CCBbaa}(i-1)(1-\Phi)F_{ccBbAa}(i-1)+0.5(1-\Phi)M_{CCBbaa}(i-1)\Phi F_{ccBbAa}(i-1)+$
$0.5(1-\Phi)M_{CCBbaa}(i-1)F_{ccBbAA}(i-1)+0.25(1-\Phi)M_{CCBbaa}(i-1)(1-\Phi)F_{ccBBAa}(i-1)+0.5(1-$
$\Phi)M_{CCBbaa}(i-1)\Phi F_{ccBBAa}(i-1)+0.5(1-\Phi)M_{CCBbaa}(i-1)F_{ccBBAA}(i-1)+0.125(1-\Phi)M_{CCBbaa}(i-$
$1)F_{CcbbAa}(i-1)+0.25(1-\Phi)M_{CCBbaa}(i-1)F_{CcbbAA}(i-1)+0.125(1-\Phi)M_{CCBbaa}(i-1)(1-\Phi)(1-$
$\Phi)F_{CcBbAa}(i-1)+0.125(1-\Phi)M_{CCBbaa}(i-1)\Phi(1-\Phi)F_{CcBbAa}(i-1)+0.25(1-\Phi)M_{CCBbaa}(i-1)(1-$
$\Phi)\Phi F_{CcBbAa}(i-1)+0.25(1-\Phi)M_{CCBbaa}(i-1)\Phi\Phi F_{CcBbAa}(i-1)+0.25(1-\Phi)M_{CCBbaa}(i-1)(1-$
$\Phi)F_{CcBbAA}(i-1)+0.25(1-\Phi)M_{CCBbaa}(i-1)\Phi F_{CcBbAA}(i-1)+0.125(1-\Phi)M_{CCBbaa}(i-1)(1-$
$\Phi)F_{CcBBAa}(i-1)+0.25(1-\Phi)M_{CCBbaa}(i-1)\Phi F_{CcBBAa}(i-1)+0.25(1-\Phi)M_{CCBbaa}(i-1)F_{CcBBAA}(i-1)+$
$0.5\Phi M_{CCBbaa}(i-1)F_{ccbbAa}(i-1)+\Phi M_{CCBbaa}(i-1)F_{ccbbAA}(i-1)+0.25\Phi M_{CCBbaa}(i-1)(1-\Phi)F_{ccBbAa}(i-$
$1)+0.5\Phi M_{CCBbaa}(i-1)\Phi F_{ccBbAa}(i-1)+0.5\Phi M_{CCBbaa}(i-1)F_{ccBbAA}(i-1)+0.25\Phi M_{CCBbaa}(i-$
$1)F_{CcbbAa}(i-1)+0.5\Phi M_{CCBbaa}(i-1)F_{CcbbAA}(i-1)+0.125\Phi M_{CCBbaa}(i-1)(1-\Phi)(1-\Phi)F_{CcBbAa}(i-$
$1)+0.25\Phi M_{CCBbaa}(i-1)(1-\Phi)\Phi F_{CcBbAa}(i-1)+0.25\Phi M_{CCBbaa}(i-1)(1-\Phi)F_{CcBbAA}(i-1)+0.25(1-$
$\Phi)(1-\Phi)M_{CCBbAa}(i-1)F_{ccbbaa}(i-1)+0.25(1-\Phi)(1-\Phi)M_{CCBbAa}(i-1)F_{ccbbAa}(i-1)+0.25(1-\Phi)(1-$
$\Phi)M_{CCBbAa}(i-1)F_{ccbbAA}(i-1)+0.25(1-\Phi)(1-\Phi)M_{CCBbAa}(i-1)F_{ccBbaa}(i-1)+0.25(1-\Phi)(1-$
$\Phi)M_{CCBbAa}(i-1)(1-\Phi)F_{ccBbAa}(i-1)+0.25(1-\Phi)(1-\Phi)M_{CCBbAa}(i-1)\Phi F_{ccBbAa}(i-1)+0.25(1-$
$\Phi)(1-\Phi)M_{CCBbAa}(i-1)F_{ccBbAA}(i-1)+0.25(1-\Phi)(1-\Phi)M_{CCBbAa}(i-1)F_{ccBBaa}(i-1)+0.25(1-$
$\Phi)(1-\Phi)M_{CCBbAa}(i-1)(1-\Phi)F_{ccBBAa}(i-1)+0.25(1-\Phi)(1-\Phi)M_{CCBbAa}(i-1)\Phi F_{ccBBAa}(i-1)+$
$0.25(1-\Phi)(1-\Phi)M_{CCBbAa}(i-1)F_{ccBBAA}(i-1)+0.125(1-\Phi)(1-\Phi)M_{CCBbAa}(i-1)F_{Ccbbaa}(i-1)+$
$0.125(1-\Phi)(1-\Phi)M_{CCBbAa}(i-1)F_{CcbbAa}(i-1)+0.125(1-\Phi)(1-\Phi)M_{CCBbAa}(i-1)F_{CcbbAA}(i-1)+$
$0.125(1-\Phi)(1-\Phi)M_{CCBbAa}(i-1)(1-\Phi)F_{CcBbaa}(i-1)+0.125(1-\Phi)(1-\Phi)M_{CCBbAa}(i-1)\Phi F_{CcBbaa}(i-$
$1)+0.125(1-\Phi)(1-\Phi)M_{CCBbAa}(i-1)(1-\Phi)(1-\Phi)F_{CcBbAa}(i-1)+0.125(1-\Phi)(1-\Phi)M_{CCBbAa}(i-$
$1)\Phi(1-\Phi)F_{CcBbAa}(i-1)+0.125(1-\Phi)(1-\Phi)M_{CCBbAa}(i-1)(1-\Phi)\Phi F_{CcBbAa}(i-1)+0.125(1-$
$\Phi)(1-\Phi)M_{CCBbAa}(i-1)\Phi\Phi F_{CcBbAa}(i-1)+0.125(1-\Phi)(1-\Phi)M_{CCBbAa}(i-1)(1-\Phi)F_{CcBbAA}(i-1)+$
$0.125(1-\Phi)(1-\Phi)M_{CCBbAa}(i-1)\Phi F_{CcBbAA}(i-1)+0.125(1-\Phi)(1-\Phi)M_{CCBbAa}(i-1)F_{CcBBaa}(i-$
$1)+0.125(1-\Phi)(1-\Phi)M_{CCBbAa}(i-1)(1-\Phi)F_{CcBBAa}(i-1)+0.125(1-\Phi)(1-\Phi)M_{CCBbAa}(i-$
$1)\Phi F_{CcBBAa}(i-1)+0.125(1-\Phi)(1-\Phi)M_{CCBbAa}(i-1)F_{CcBBAA}(i-1)+0.5\Phi(1-\Phi)M_{CCBbAa}(i-$
$1)F_{ccbbaa}(i-1)+0.5\Phi(1-\Phi)M_{CCBbAa}(i-1)F_{ccbbAa}(i-1)+0.5\Phi(1-\Phi)M_{CCBbAa}(i-1)F_{ccbbAA}(i-1)+$
$0.25\Phi(1-\Phi)M_{CCBbAa}(i-1)F_{ccBbaa}(i-1)+0.25\Phi(1-\Phi)M_{CCBbAa}(i-1)(1-\Phi)F_{ccBbAa}(i-1)+0.25\Phi(1-$
$\Phi)M_{CCBbAa}(i-1)\Phi F_{ccBbAa}(i-1)+0.25\Phi(1-\Phi)M_{CCBbAa}(i-1)F_{ccBbAA}(i-1)+0.25\Phi(1-\Phi)M_{CCBbAa}(i-$
$1)F_{Ccbbaa}(i-1)+0.25\Phi(1-\Phi)M_{CCBbAa}(i-1)F_{CcbbAa}(i-1)+0.25\Phi(1-\Phi)M_{CCBbAa}(i-1)F_{CcbbAA}(i-$
$1)+0.125\Phi(1-\Phi)M_{CCBbAa}(i-1)(1-\Phi)F_{CcBbaa}(i-1)+0.125\Phi(1-\Phi)M_{CCBbAa}(i-1)(1-\Phi)(1-$
$\Phi)F_{CcBbAa}(i-1)+0.125\Phi(1-\Phi)M_{CCBbAa}(i-1)(1-\Phi)\Phi F_{CcBbAa}(i-1)+0.125\Phi(1-\Phi)M_{CCBbAa}(i-$
$1)(1-\Phi)F_{CcBbAA}(i-1)+0.5(1-\Phi)\Phi M_{CCBbAa}(i-1)F_{ccbbaa}(i-1)+0.25(1-\Phi)\Phi M_{CCBbAa}(i-1)F_{ccbbAa}(i-$
$1)+0.5(1-\Phi)\Phi M_{CCBbAa}(i-1)F_{ccBbaa}(i-1)+0.25(1-\Phi)\Phi M_{CCBbAa}(i-1)(1-\Phi)F_{ccBbAa}(i-1)+$
$0.5(1-\Phi)\Phi M_{CCBbAa}(i-1)F_{ccBBaa}(i-1)+0.25(1-\Phi)\Phi M_{CCBbAa}(i-1)(1-\Phi)F_{ccBBAa}(i-1)+0.25(1-$
$\Phi)\Phi M_{CCBbAa}(i-1)F_{Ccbbaa}(i-1)+0.125(1-\Phi)\Phi M_{CCBbAa}(i-1)F_{CcbbAa}(i-1)+0.25(1-\Phi)\Phi M_{CCBbAa}(i-$
$1)(1-\Phi)F_{CcBbaa}(i-1)+0.25(1-\Phi)\Phi M_{CCBbAa}(i-1)\Phi F_{CcBbaa}(i-1)+0.125(1-\Phi)\Phi M_{CCBbAa}(i-1)(1-$
$\Phi)(1-\Phi)F_{CcBbAa}(i-1)+0.125(1-\Phi)\Phi M_{CCBbAa}(i-1)\Phi(1-\Phi)F_{CcBbAa}(i-1)+0.25(1-\Phi)\Phi M_{CCBbAa}(i-$
$1)F_{CcBBaa}(i-1)+0.125(1-\Phi)\Phi M_{CCBbAa}(i-1)(1-\Phi)F_{CcBBAa}(i-1)+\Phi\Phi M_{CCBbAa}(i-1)F_{ccbbaa}(i-$
$1)+0.5\Phi\Phi M_{CCBbAa}(i-1)F_{ccbbAa}(i-1)+0.5\Phi\Phi M_{CCBbAa}(i-1)F_{ccBbaa}(i-1)+0.25\Phi\Phi M_{CCBbAa}(i-$
$1)(1-\Phi)F_{ccBbAa}(i-1)+0.5\Phi\Phi M_{CCBbAa}(i-1)F_{Ccbbaa}(i-1)+0.25\Phi\Phi M_{CCBbAa}(i-1)F_{CcbbAa}(i-1)+$
$0.25\Phi\Phi M_{CCBbAa}(i-1)(1-\Phi)F_{CcBbaa}(i-1)+0.125\Phi\Phi M_{CCBbAa}(i-1)(1-\Phi)(1-\Phi)F_{CcBbAa}(i-1)+$
$0.5(1-\Phi)M_{CCBbAA}(i-1)F_{ccbbaa}(i-1)+0.25(1-\Phi)M_{CCBbAA}(i-1)F_{ccbbAa}(i-1)+0.5(1-\Phi)M_{CCBbAA}(i-$
$1)F_{ccBbaa}(i-1)+0.25(1-\Phi)M_{CCBbAA}(i-1)(1-\Phi)F_{ccBbAa}(i-1)+0.5(1-\Phi)M_{CCBbAA}(i-1)F_{ccBBaa}(i-$
$1)+0.25(1-\Phi)M_{CCBbAA}(i-1)(1-\Phi)F_{ccBBAa}(i-1)+0.25(1-\Phi)M_{CCBbAA}(i-1)F_{Ccbbaa}(i-1)+$
$0.125(1-\Phi)M_{CCBbAA}(i-1)F_{CcbbAa}(i-1)+0.25(1-\Phi)M_{CCBbAA}(i-1)(1-\Phi)F_{CcBbaa}(i-1)+0.25(1-$
$\Phi)M_{CCBbAA}(i-1)\Phi F_{CcBbaa}(i-1)+0.125(1-\Phi)M_{CCBbAA}(i-1)(1-\Phi)(1-\Phi)F_{CcBbAa}(i-1)+0.125(1-$
$\Phi)M_{CCBbAA}(i-1)\Phi(1-\Phi)F_{CcBbAa}(i-1)+0.25(1-\Phi)M_{CCBbAA}(i-1)F_{CcBBaa}(i-1)+0.125(1-$

$\Phi)M_{CCBbAA}(i-1)(1-\Phi)F_{CcBBAa}(i-1)+\Phi M_{CCBbAA}(i-1)F_{ccbbaa}(i-1)+0.5\Phi M_{CCBbAA}(i-1)F_{ccbbAa}(i-$
$1)+0.5\Phi M_{CCBbAA}(i-1)F_{ccBbaa}(i-1)+0.25\Phi M_{CCBbAA}(i-1)(1-\Phi)F_{ccBbAa}(i-1)+0.5\Phi M_{CCBbAA}(i-$
$1)F_{Ccbbaa}(i-1)+0.25\Phi M_{CCBbAA}(i-1)F_{CcbbAa}(i-1)+0.25\Phi M_{CCBbAA}(i-1)(1-\Phi)F_{CcBbaa}(i-1)+$
$0.125\Phi M_{CCBbAA}(i-1)(1-\Phi)(1-\Phi)F_{CcBbAa}(i-1)+0.5M_{CCBBaa}(i-1)F_{ccbbAa}(i-1)+M_{CCBBaa}(i-$
$1)F_{ccbbAA}(i-1)+0.25M_{CCBBaa}(i-1)(1-\Phi)F_{ccBbAa}(i-1)+0.5M_{CCBBaa}(i-1)\Phi F_{ccBbAa}(i-1)+$
$0.5M_{CCBBaa}(i-1)F_{ccBbAA}(i-1)+0.25M_{CCBBaa}(i-1)F_{CcbbAa}(i-1)+0.5M_{CCBBaa}(i-1)F_{CcbbAA}(i-$
$1)+0.125M_{CCBBaa}(i-1)(1-\Phi)(1-\Phi)F_{CcBbAa}(i-1)+0.25M_{CCBBaa}(i-1)(1-\Phi)\Phi F_{CcBbAa}(i-1)+$
$0.25M_{CCBBaa}(i-1)(1-\Phi)F_{CcBbAA}(i-1)+0.5(1-\Phi)M_{CCBBAa}(i-1)F_{ccbbaa}(i-1)+0.5(1-\Phi)M_{CCBBAa}(i-$
$1)F_{ccbbAa}(i-1)+0.5(1-\Phi)M_{CCBBAa}(i-1)F_{ccbbAA}(i-1)+0.25(1-\Phi)M_{CCBBAa}(i-1)F_{ccBbaa}(i-1)+$
$0.25(1-\Phi)M_{CCBBAa}(i-1)(1-\Phi)F_{ccBbAa}(i-1)+0.25(1-\Phi)M_{CCBBAa}(i-1)\Phi F_{ccBbAa}(i-1)+0.25(1-$
$\Phi)M_{CCBBAa}(i-1)F_{ccBbAA}(i-1)+0.25(1-\Phi)M_{CCBBAa}(i-1)F_{Ccbbaa}(i-1)+0.25(1-\Phi)M_{CCBBAa}(i-$
$1)F_{CcbbAa}(i-1)+0.25(1-\Phi)M_{CCBBAa}(i-1)F_{CcbbAA}(i-1)+0.125(1-\Phi)M_{CCBBAa}(i-1)(1-\Phi)F_{CcBbaa}(i-$
$1)+0.125(1-\Phi)M_{CCBBAa}(i-1)(1-\Phi)(1-\Phi)F_{CcBbAa}(i-1)+0.125(1-\Phi)M_{CCBBAa}(i-1)(1-$
$\Phi)\Phi F_{CcBbAa}(i-1)+0.125(1-\Phi)M_{CCBBAa}(i-1)(1-\Phi)F_{CcBbAA}(i-1)+\Phi M_{CCBBAA}(i-1)F_{ccbbaa}(i-$
$1)+0.5\Phi M_{CCBBAA}(i-1)F_{ccbbAa}(i-1)+0.5\Phi M_{CCBBAA}(i-1)F_{ccBbaa}(i-1)+0.25\Phi M_{CCBBAA}(i-$
$1)(1-\Phi)F_{ccBbAa}(i-1)+0.5\Phi M_{CCBBAA}(i-1)F_{Ccbbaa}(i-1)+0.25\Phi M_{CCBBAA}(i-1)F_{CcbbAa}(i-1)+$
$0.25\Phi M_{CCBBAA}(i-1)(1-\Phi)F_{CcBbaa}(i-1)+0.125\Phi M_{CCBBAA}(i-1)(1-\Phi)(1-\Phi)F_{CcBbAa}(i-1)+$
$M_{CCBBAA}(i-1)F_{ccbbaa}(i-1)+0.5M_{CCBBAA}(i-1)F_{ccbbAa}(i-1)+0.5M_{CCBBAA}(i-1)F_{ccBbaa}(i-1)+$
$0.25M_{CCBBAA}(i-1)(1-\Phi)F_{ccBbAa}(i-1)+0.5M_{CCBBAA}(i-1)F_{Ccbbaa}(i-1)+0.25M_{CCBBAA}(i-$
$1)F_{CcbbAa}(i-1)+0.25M_{CCBBAA}(i-1)(1-\Phi)F_{CcBbaa}(i-1)+0.125M_{CCBBAA}(i-1)(1-\Phi)(1-$
$\Phi)F_{CcBbAa}(i-1)],$

$\mathrm{M}^{e}_{CcBbAA}=\left(\frac{\bar{\Omega}_{CcBbAA}}{2}\right)[0.0625M_{ccbbAa}(i-1)(1-\Phi)(1-\Phi)F_{CcBbAa}(i-1)+0.125M_{ccbbAa}(i-1)\Phi(1-$
$\Phi)F_{CcBbAa}(i-1)+0.125M_{ccbbAa}(i-1)(1-\Phi)\Phi F_{CcBbAa}(i-1)+0.25M_{ccbbAa}(i-1)\Phi\Phi F_{CcBbAa}(i-$
$1)+0.125M_{ccbbAa}(i-1)(1-\Phi)F_{CcBbAA}(i-1)+0.25M_{ccbbAa}(i-1)\Phi F_{CcBbAA}(i-1)+0.125M_{ccbbAa}(i-$
$1)(1-\Phi)F_{CcBBAa}(i-1)+0.25M_{ccbbAa}(i-1)\Phi F_{CcBBAa}(i-1)+0.25M_{ccbbAa}(i-1)F_{CcBBAA}(i-1)+$
$0.125M_{ccbbAa}(i-1)(1-\Phi)(1-\Phi)F_{CCBbAa}(i-1)+0.25M_{ccbbAa}(i-1)\Phi(1-\Phi)F_{CCBbAa}(i-1)+0.25M_{ccbbAa}(i-$
$1)(1-\Phi)\Phi F_{CCBbAa}(i-1)+0.5M_{ccbbAa}(i-1)\Phi\Phi F_{CCBbAa}(i-1)+0.25M_{ccbbAa}(i-1)(1-\Phi)F_{CCBbAA}(i-$
$1)+0.5M_{ccbbAa}(i-1)\Phi F_{CCBbAA}(i-1)+0.25M_{ccbbAa}(i-1)(1-\Phi)F_{CCBBAa}(i-1)+0.5M_{ccbbAa}(i-$
$1)\Phi F_{CCBBAa}(i-1)+0.5M_{ccbbAa}(i-1)F_{CCBBAA}(i-1)+0.125M_{ccbbAA}(i-1)(1-\Phi)(1-\Phi)F_{CcBbAa}(i-$
$1)+0.25M_{ccbbAA}(i-1)\Phi(1-\Phi)F_{CcBbAa}(i-1)+0.25M_{ccbbAA}(i-1)(1-\Phi)\Phi F_{CcBbAa}(i-1)+0.5M_{ccbbAA}(i-$
$1)\Phi\Phi F_{CcBbAa}(i-1)+0.25M_{ccbbAA}(i-1)(1-\Phi)F_{CcBbAA}(i-1)+0.5M_{ccbbAA}(i-1)\Phi F_{CcBbAA}(i-1)+$
$0.25M_{ccbbAA}(i-1)(1-\Phi)F_{CcBBAa}(i-1)+0.5M_{ccbbAA}(i-1)\Phi F_{CcBBAa}(i-1)+0.5M_{ccbbAA}(i-1)F_{CcBBAA}(i-$
$1)+0.25M_{ccbbAA}(i-1)(1-\Phi)(1-\Phi)F_{CCBbAa}(i-1)+0.5M_{ccbbAA}(i-1)\Phi(1-\Phi)F_{CCBbAa}(i-1)+$
$0.5M_{ccbbAA}(i-1)(1-\Phi)\Phi F_{CCBbAa}(i-1)+M_{ccbbAA}(i-1)\Phi\Phi F_{CCBbAa}(i-1)+0.5M_{ccbbAA}(i-1)(1-$
$\Phi)F_{CCBbAA}(i-1)+M_{ccbbAA}(i-1)\Phi F_{CCBbAA}(i-1)+0.5M_{ccbbAA}(i-1)(1-\Phi)F_{CCBBAa}(i-1)+M_{ccbbAA}(i-$
$1)\Phi F_{CCBBAa}(i-1)+M_{ccbbAA}(i-1)F_{CCBBAA}(i-1)+0.0625(1-\Phi)M_{ccBbAa}(i-1)F_{CcbbAa}(i-1)+$
$0.125(1-\Phi)M_{ccBbAa}(i-1)F_{CcbbAA}(i-1)+0.0625(1-\Phi)M_{ccBbAa}(i-1)(1-\Phi)(1-\Phi)F_{CcBbAa}(i-1)+$
$0.0625(1-\Phi)M_{ccBbAa}(i-1)\Phi(1-\Phi)F_{CcBbAa}(i-1)+0.125(1-\Phi)M_{ccBbAa}(i-1)(1-\Phi)\Phi F_{CcBbAa}(i-$
$1)+0.125(1-\Phi)M_{ccBbAa}(i-1)\Phi\Phi F_{CcBbAa}(i-1)+0.125(1-\Phi)M_{ccBbAa}(i-1)(1-\Phi)F_{CcBbAA}(i-1)+$
$0.125(1-\Phi)M_{ccBbAa}(i-1)\Phi F_{CcBbAA}(i-1)+0.0625(1-\Phi)M_{ccBbAa}(i-1)(1-\Phi)F_{CcBBAa}(i-1)+$
$0.125(1-\Phi)M_{ccBbAa}(i-1)\Phi F_{CcBBAa}(i-1)+0.125(1-\Phi)M_{ccBbAa}(i-1)F_{CcBBAA}(i-1)+0.125(1-$
$\Phi)M_{ccBbAa}(i-1)F_{CCbbAa}(i-1)+0.25(1-\Phi)M_{ccBbAa}(i-1)F_{CCbbAA}(i-1)+0.125(1-\Phi)M_{ccBbAa}(i-1)(1-$
$\Phi)(1-\Phi)F_{CCBbAa}(i-1)+0.125(1-\Phi)M_{ccBbAa}(i-1)\Phi(1-\Phi)F_{CCBbAa}(i-1)+0.125(1-\Phi)M_{ccBbAa}(i-$
$1)(1-\Phi)\Phi F_{CCBbAa}(i-1)+0.25(1-\Phi)M_{ccBbAa}(i-1)\Phi\Phi F_{CCBbAa}(i-1)+0.25(1-\Phi)M_{ccBbAa}(i-$
$1)(1-\Phi)F_{CCBbAA}(i-1)+0.25(1-\Phi)M_{ccBbAa}(i-1)\Phi F_{CCBbAA}(i-1)+0.125(1-\Phi)M_{ccBbAa}(i-1)(1-$
$\Phi)F_{CCBBAa}(i-1)+0.25(1-\Phi)M_{ccBbAa}(i-1)\Phi F_{CCBBAa}(i-1)+0.25(1-\Phi)M_{ccBbAa}(i-1)F_{CCBBAA}(i-1)+$
$0.125\Phi M_{ccBbAa}(i-1)F_{CcbbAa}(i-1)+0.25\Phi M_{ccBbAa}(i-1)F_{CcbbAA}(i-1)+0.125\Phi M_{ccBbAa}(i-1)(1-\Phi)(1-$
$\Phi)F_{CcBbAa}(i-1)+0.125\Phi M_{ccBbAa}(i-1)\Phi(1-\Phi)F_{CcBbAa}(i-1)+0.25\Phi M_{ccBbAa}(i-1)(1-\Phi)\Phi F_{CcBbAa}(i-$
$1)+0.25\Phi M_{ccBbAa}(i-1)\Phi\Phi F_{CcBbAa}(i-1)+0.25\Phi M_{ccBbAa}(i-1)(1-\Phi)F_{CcBbAA}(i-1)+0.25\Phi M_{ccBbAa}(i-$
$1)\Phi F_{CcBbAA}(i-1)+0.125\Phi M_{ccBbAa}(i-1)(1-\Phi)F_{CcBBAa}(i-1)+0.25\Phi M_{ccBbAa}(i-1)\Phi F_{CcBBAa}(i-1)+$
$0.25\Phi M_{ccBbAa}(i-1)F_{CcBBAA}(i-1)+0.25\Phi M_{ccBbAa}(i-1)F_{CCbbAa}(i-1)+0.5\Phi M_{ccBbAa}(i-1)F_{CCbbAA}(i-$
$1)+0.25\Phi M_{ccBbAa}(i-1)(1-\Phi)(1-\Phi)F_{CCBbAa}(i-1)+0.25\Phi M_{ccBbAa}(i-1)\Phi(1-\Phi)F_{CCBbAa}(i-1)+$
$0.25\Phi M_{ccBbAa}(i-1)(1-\Phi)\Phi F_{CCBbAa}(i-1)+0.5\Phi M_{ccBbAa}(i-1)\Phi\Phi F_{CCBbAa}(i-1)+0.5\Phi M_{ccBbAa}(i-$
$1)(1-\Phi)F_{CCBbAA}(i-1)+0.5\Phi M_{ccBbAa}(i-1)\Phi F_{CCBbAA}(i-1)+0.25\Phi M_{ccBbAa}(i-1)(1-\Phi)F_{CCBBAa}(i-$
$1)+0.5\Phi M_{ccBbAa}(i-1)\Phi F_{CCBBAa}(i-1)+0.5\Phi M_{ccBbAa}(i-1)F_{CCBBAA}(i-1)+0.125M_{ccBbAA}(i-$
$1)F_{CcbbAa}(i-1)+0.25M_{ccBbAA}(i-1)F_{CcbbAA}(i-1)+0.125M_{ccBbAA}(i-1)(1-\Phi)(1-\Phi)F_{CcBbAa}(i-1)+$

$$
0.125M_{ccBbAA}(i-1)\Phi(1-\Phi)F_{CcBbAa}(i-1)+0.25M_{ccBbAA}(i-1)(1-\Phi)\Phi F_{CcBbAa}(i-1)+0.25M_{ccBbAA}(i-
1)\Phi\Phi F_{CcBbAa}(i-1)+0.25M_{ccBbAA}(i-1)(1-\Phi)F_{CcBbAA}(i-1)+0.25M_{ccBbAA}(i-1)\Phi F_{CcBbAA}(i-1)+
0.125M_{ccBbAA}(i-1)(1-\Phi)F_{CcBBAa}(i-1)+0.25M_{ccBbAA}(i-1)\Phi F_{CcBBAa}(i-1)+0.25M_{ccBbAA}(i-
1)F_{CcBBAA}(i-1)+0.25M_{ccBbAA}(i-1)F_{CCbbAa}(i-1)+0.5M_{ccBbAA}(i-1)F_{CCbbAA}(i-1)+0.25M_{ccBbAA}(i-
1)(1-\Phi)(1-\Phi)F_{CCBbAa}(i-1)+0.25M_{ccBbAA}(i-1)\Phi(1-\Phi)F_{CCBbAa}(i-1)+0.25M_{ccBbAA}(i-1)(1-
\Phi)\Phi F_{CCBbAa}(i-1)+0.5M_{ccBbAA}(i-1)\Phi\Phi F_{CCBbAa}(i-1)+0.5M_{ccBbAA}(i-1)(1-\Phi)F_{CCBbAA}(i-
1)+0.5M_{ccBbAA}(i-1)\Phi F_{CCBbAA}(i-1)+0.25M_{ccBbAA}(i-1)(1-\Phi)F_{CCBBAa}(i-1)+0.5M_{ccBbAA}(i-
1)\Phi F_{CCBBAa}(i-1)+0.5M_{ccBbAA}(i-1)F_{CCBBAA}(i-1)+0.125(1-\Phi)M_{ccBBAa}(i-1)F_{CcbbAa}(i-1)+
0.25(1-\Phi)M_{ccBBAa}(i-1)F_{CcbbAA}(i-1)+0.0625(1-\Phi)M_{ccBBAa}(i-1)(1-\Phi)(1-\Phi)F_{CcBbAa}(i-1)+
0.125(1-\Phi)M_{ccBBAa}(i-1)(1-\Phi)\Phi F_{CcBbAa}(i-1)+0.125(1-\Phi)M_{ccBBAa}(i-1)(1-\Phi)F_{CcBbAA}(i-
1)+0.25(1-\Phi)M_{ccBBAa}(i-1)F_{CCbbAa}(i-1)+0.5(1-\Phi)M_{ccBBAa}(i-1)F_{CCbbAA}(i-1)+0.125(1-
\Phi)M_{ccBBAa}(i-1)(1-\Phi)(1-\Phi)F_{CCBbAa}(i-1)+0.25(1-\Phi)M_{ccBBAa}(i-1)(1-\Phi)F_{CCBbAA}(i-1)+
0.25\Phi M_{ccBBAa}(i-1)F_{CcbbAa}(i-1)+0.5\Phi M_{ccBBAa}(i-1)F_{CcbbAA}(i-1)+0.125\Phi M_{ccBBAa}(i-1)(1-\Phi)(1-
\Phi)F_{CcBbAa}(i-1)+0.25\Phi M_{ccBBAa}(i-1)(1-\Phi)\Phi F_{CcBbAa}(i-1)+0.25\Phi M_{ccBBAa}(i-1)(1-\Phi)F_{CcBbAA}(i-
1)+0.5\Phi M_{ccBBAa}(i-1)F_{CCbbAa}(i-1)+\Phi M_{ccBBAa}(i-1)F_{CCbbAA}(i-1)+0.25\Phi M_{ccBBAa}(i-1)(1-
\Phi)(1-\Phi)F_{CCBbAa}(i-1)+0.5\Phi M_{ccBBAa}(i-1)(1-\Phi)F_{CCBbAA}(i-1)+0.25M_{ccBBAA}(i-1)F_{CcbbAa}(i-1)+
0.5M_{ccBBAA}(i-1)F_{CcbbAA}(i-1)+0.125M_{ccBBAA}(i-1)(1-\Phi)(1-\Phi)F_{CcBbAa}(i-1)+0.25M_{ccBBAA}(i-
1)(1-\Phi)\Phi F_{CcBbAa}(i-1)+0.25M_{ccBBAA}(i-1)(1-\Phi)F_{CcBbAA}(i-1)+0.5M_{ccBBAA}(i-1)F_{CCbbAa}(i-
1)+M_{ccBBAA}(i-1)F_{CCbbAA}(i-1)+0.25M_{ccBBAA}(i-1)(1-\Phi)(1-\Phi)F_{CCBbAa}(i-1)+0.5M_{ccBBAA}(i-
1)(1-\Phi)F_{CCBbAA}(i-1)+0.0625M_{CcbbAa}(i-1)(1-\Phi)F_{ccBbAa}(i-1)+0.125M_{CcbbAa}(i-1)\Phi F_{ccBbAa}(i-
1)+0.125M_{CcbbAa}(i-1)F_{ccBbAA}(i-1)+0.125M_{CcbbAa}(i-1)(1-\Phi)F_{ccBBAa}(i-1)+0.25M_{CcbbAa}(i-
1)\Phi F_{ccBBAa}(i-1)+0.25M_{CcbbAa}(i-1)F_{ccBBAA}(i-1)+0.0625M_{CcbbAa}(i-1)(1-\Phi)(1-\Phi)F_{CcBbAa}(i-1)+
0.125M_{CcbbAa}(i-1)\Phi(1-\Phi)F_{CcBbAa}(i-1)+0.125M_{CcbbAa}(i-1)(1-\Phi)\Phi F_{CcBbAa}(i-1)+0.25M_{CcbbAa}(i-
1)\Phi\Phi F_{CcBbAa}(i-1)+0.125M_{CcbbAa}(i-1)(1-\Phi)F_{CcBbAA}(i-1)+0.25M_{CcbbAa}(i-1)\Phi F_{CcBbAA}(i-1)+
0.125M_{CcbbAa}(i-1)(1-\Phi)F_{CcBBAa}(i-1)+0.25M_{CcbbAa}(i-1)\Phi F_{CcBBAa}(i-1)+0.25M_{CcbbAa}(i-
1)F_{CcBBAA}(i-1)+0.0625M_{CcbbAa}(i-1)(1-\Phi)(1-\Phi)F_{CCBbAa}(i-1)+0.125M_{CcbbAa}(i-1)\Phi(1-
\Phi)F_{CCBbAa}(i-1)+0.125M_{CcbbAa}(i-1)(1-\Phi)\Phi F_{CCBbAa}(i-1)+0.25M_{CcbbAa}(i-1)\Phi\Phi F_{CCBbAa}(i-
1)+0.125M_{CcbbAa}(i-1)(1-\Phi)F_{CCBbAA}(i-1)+0.25M_{CcbbAa}(i-1)\Phi F_{CCBbAA}(i-1)+0.125M_{CcbbAa}(i-
1)(1-\Phi)F_{CCBBAa}(i-1)+0.25M_{CcbbAa}(i-1)\Phi F_{CCBBAa}(i-1)+0.25M_{CcbbAa}(i-1)F_{CCBBAA}(i-
1)+0.125M_{CcbbAA}(i-1)(1-\Phi)F_{ccBbAa}(i-1)+0.25M_{CcbbAA}(i-1)\Phi F_{ccBbAa}(i-1)+0.25M_{CcbbAA}(i-
1)F_{ccBbAA}(i-1)+0.25M_{CcbbAA}(i-1)(1-\Phi)F_{ccBBAa}(i-1)+0.5M_{CcbbAA}(i-1)\Phi F_{ccBBAa}(i-1)+
0.5M_{CcbbAA}(i-1)F_{ccBBAA}(i-1)+0.125M_{CcbbAA}(i-1)(1-\Phi)(1-\Phi)F_{CcBbAa}(i-1)+0.25M_{CcbbAA}(i-
1)\Phi(1-\Phi)F_{CcBbAa}(i-1)+0.25M_{CcbbAA}(i-1)(1-\Phi)\Phi F_{CcBbAa}(i-1)+0.5M_{CcbbAA}(i-1)\Phi\Phi F_{CcBbAa}(i-
1)+0.25M_{CcbbAA}(i-1)(1-\Phi)F_{CcBbAA}(i-1)+0.5M_{CcbbAA}(i-1)\Phi F_{CcBbAA}(i-1)+0.25M_{CcbbAA}(i-
1)(1-\Phi)F_{CcBBAa}(i-1)+0.5M_{CcbbAA}(i-1)\Phi F_{CcBBAa}(i-1)+0.5M_{CcbbAA}(i-1)F_{CcBBAA}(i-1)+
0.125M_{CcbbAA}(i-1)(1-\Phi)(1-\Phi)F_{CCBbAa}(i-1)+0.25M_{CcbbAA}(i-1)\Phi(1-\Phi)F_{CCBbAa}(i-1)+
0.25M_{CcbbAA}(i-1)(1-\Phi)\Phi F_{CCBbAa}(i-1)+0.5M_{CcbbAA}(i-1)\Phi\Phi F_{CCBbAa}(i-1)+0.25M_{CcbbAA}(i-
1)(1-\Phi)F_{CCBbAA}(i-1)+0.5M_{CcbbAA}(i-1)\Phi F_{CCBbAA}(i-1)+0.25M_{CcbbAA}(i-1)(1-\Phi)F_{CCBBAa}(i-
1)+0.5M_{CcbbAA}(i-1)\Phi F_{CCBBAa}(i-1)+0.5M_{CcbbAA}(i-1)F_{CCBBAA}(i-1)+0.0625(1-\Phi)(1-
\Phi)M_{CcBbAa}(i-1)F_{ccbbAa}(i-1)+0.125(1-\Phi)(1-\Phi)M_{CcBbAa}(i-1)F_{ccbbAA}(i-1)+0.0625(1-\Phi)(1-
\Phi)M_{CcBbAa}(i-1)(1-\Phi)F_{ccBbAa}(i-1)+0.125(1-\Phi)(1-\Phi)M_{CcBbAa}(i-1)\Phi F_{ccBbAa}(i-1)+0.125(1-
\Phi)(1-\Phi)M_{CcBbAa}(i-1)F_{ccBbAA}(i-1)+0.0625(1-\Phi)(1-\Phi)M_{CcBbAa}(i-1)(1-\Phi)F_{ccBBAa}(i-1)+
0.125(1-\Phi)(1-\Phi)M_{CcBbAa}(i-1)\Phi F_{ccBBAa}(i-1)+0.125(1-\Phi)(1-\Phi)M_{CcBbAa}(i-1)F_{ccBBAA}(i-
1)+0.0625(1-\Phi)(1-\Phi)M_{CcBbAa}(i-1)F_{CcbbAa}(i-1)+0.125(1-\Phi)(1-\Phi)M_{CcBbAa}(i-1)F_{CcbbAA}(i-
1)+0.0625(1-\Phi)(1-\Phi)M_{CcBbAa}(i-1)(1-\Phi)(1-\Phi)F_{CcBbAa}(i-1)+0.0625(1-\Phi)(1-\Phi)M_{CcBbAa}(i-
1)\Phi(1-\Phi)F_{CcBbAa}(i-1)+0.125(1-\Phi)(1-\Phi)M_{CcBbAa}(i-1)(1-\Phi)\Phi F_{CcBbAa}(i-1)+0.125(1-\Phi)(1-
\Phi)M_{CcBbAa}(i-1)\Phi\Phi F_{CcBbAa}(i-1)+0.125(1-\Phi)(1-\Phi)M_{CcBbAa}(i-1)(1-\Phi)F_{CcBbAA}(i-1)+0.125(1-
\Phi)(1-\Phi)M_{CcBbAa}(i-1)\Phi F_{CcBbAA}(i-1)+0.0625(1-\Phi)(1-\Phi)M_{CcBbAa}(i-1)(1-\Phi)F_{CcBBAa}(i-1)+
0.125(1-\Phi)(1-\Phi)M_{CcBbAa}(i-1)\Phi F_{CcBBAa}(i-1)+0.125(1-\Phi)(1-\Phi)M_{CcBbAa}(i-1)F_{CcBBAA}(i-
1)+0.0625(1-\Phi)(1-\Phi)M_{CcBbAa}(i-1)F_{CCbbAa}(i-1)+0.125(1-\Phi)(1-\Phi)M_{CcBbAa}(i-1)F_{CCbbAA}(i-
1)+0.0625(1-\Phi)(1-\Phi)M_{CcBbAa}(i-1)(1-\Phi)(1-\Phi)F_{CCBbAa}(i-1)+0.0625(1-\Phi)(1-\Phi)M_{CcBbAa}(i-
1)\Phi(1-\Phi)F_{CCBbAa}(i-1)+0.0625(1-\Phi)(1-\Phi)M_{CcBbAa}(i-1)(1-\Phi)\Phi F_{CCBbAa}(i-1)+0.125(1-
\Phi)(1-\Phi)M_{CcBbAa}(i-1)\Phi\Phi F_{CCBbAa}(i-1)+0.125(1-\Phi)(1-\Phi)M_{CcBbAa}(i-1)(1-\Phi)F_{CCBbAA}(i-
1)+0.125(1-\Phi)(1-\Phi)M_{CcBbAa}(i-1)\Phi F_{CCBbAA}(i-1)+0.0625(1-\Phi)(1-\Phi)M_{CcBbAa}(i-1)(1-
\Phi)F_{CCBBAa}(i-1)+0.125(1-\Phi)(1-\Phi)M_{CcBbAa}(i-1)\Phi F_{CCBBAa}(i-1)+0.125(1-\Phi)(1-\Phi)M_{CcBbAa}(i-
1)F_{CCBBAA}(i-1)+0.125\Phi(1-\Phi)M_{CcBbAa}(i-1)F_{ccbbAa}(i-1)+0.25\Phi(1-\Phi)M_{CcBbAa}(i-1)F_{ccbbAA}(i-
1)+0.0625\Phi(1-\Phi)M_{CcBbAa}(i-1)(1-\Phi)F_{ccBbAa}(i-1)+0.125\Phi(1-\Phi)M_{CcBbAa}(i-1)\Phi F_{ccBbAa}(i-1)+
$$

$$0.125\Phi(1-\Phi)M_{CcBbAa}(i-1)F_{ccBbAA}(i-1)+0.125\Phi(1-\Phi)M_{CcBbAa}(i-1)F_{CcbbAa}(i-1)+0.25\Phi(1-\Phi)M_{CcBbAa}(i-1)F_{CcbbAA}(i-1)+0.0625\Phi(1-\Phi)M_{CcBbAa}(i-1)(1-\Phi)(1-\Phi)F_{CcBbAa}(i-1)+0.125\Phi(1-\Phi)M_{CcBbAa}(i-1)(1-\Phi)\Phi F_{CcBbAa}(i-1)+0.125\Phi(1-\Phi)M_{CcBbAa}(i-1)(1-\Phi)F_{CcBbAA}(i-1)+0.125\Phi(1-\Phi)M_{CcBbAa}(i-1)F_{CCbbAa}(i-1)+0.25\Phi(1-\Phi)M_{CcBbAa}(i-1)F_{CCbbAA}(i-1)+0.0625\Phi(1-\Phi)M_{CcBbAa}(i-1)(1-\Phi)(1-\Phi)F_{CCBbAa}(i-1)+0.125\Phi(1-\Phi)M_{CcBbAa}(i-1)(1-\Phi)F_{CCBbAA}(i-1)+0.125(1-\Phi)\Phi M_{CcBbAa}(i-1)F_{ccbbAa}(i-1)+0.25(1-\Phi)\Phi M_{CcBbAa}(i-1)F_{ccbbAA}(i-1)+0.125(1-\Phi)\Phi M_{CcBbAa}(i-1)(1-\Phi)F_{ccBbAa}(i-1)+0.25(1-\Phi)\Phi M_{CcBbAa}(i-1)\Phi F_{ccBbAa}(i-1)+0.25(1-\Phi)\Phi M_{CcBbAa}(i-1)F_{ccBbAA}(i-1)+0.125(1-\Phi)\Phi M_{CcBbAa}(i-1)(1-\Phi)F_{ccBBAa}(i-1)+0.25(1-\Phi)\Phi M_{CcBbAa}(i-1)\Phi F_{ccBBAa}(i-1)+0.25(1-\Phi)\Phi M_{CcBbAa}(i-1)F_{ccBBAA}(i-1)+0.125(1-\Phi)\Phi M_{CcBbAa}(i-1)F_{CcbbAa}(i-1)+0.25(1-\Phi)\Phi M_{CcBbAa}(i-1)F_{CcbbAA}(i-1)+0.125(1-\Phi)\Phi M_{CcBbAa}(i-1)(1-\Phi)(1-\Phi)F_{CcBbAa}(i-1)+0.125(1-\Phi)\Phi M_{CcBbAa}(i-1)\Phi(1-\Phi)F_{CcBbAa}(i-1)+0.25(1-\Phi)\Phi M_{CcBbAa}(i-1)(1-\Phi)\Phi F_{CcBbAa}(i-1)+0.25(1-\Phi)\Phi M_{CcBbAa}(i-1)\Phi\Phi F_{CcBbAa}(i-1)+0.25(1-\Phi)\Phi M_{CcBbAa}(i-1)(1-\Phi)F_{CcBbAA}(i-1)+0.25(1-\Phi)\Phi M_{CcBbAa}(i-1)\Phi F_{CcBbAA}(i-1)+0.125(1-\Phi)\Phi M_{CcBbAa}(i-1)(1-\Phi)F_{CcBBAa}(i-1)+0.25(1-\Phi)\Phi M_{CcBbAa}(i-1)\Phi F_{CcBBAa}(i-1)+0.25(1-\Phi)\Phi M_{CcBbAa}(i-1)F_{CcBBAA}(i-1)+0.125(1-\Phi)\Phi M_{CcBbAa}(i-1)F_{CCbbAa}(i-1)+0.25(1-\Phi)\Phi M_{CcBbAa}(i-1)F_{CCbbAA}(i-1)+0.125(1-\Phi)\Phi M_{CcBbAa}(i-1)(1-\Phi)(1-\Phi)F_{CCBbAa}(i-1)+0.125(1-\Phi)\Phi M_{CcBbAa}(i-1)\Phi(1-\Phi)F_{CCBbAa}(i-1)+0.125(1-\Phi)\Phi M_{CcBbAa}(i-1)(1-\Phi)\Phi F_{CCBbAa}(i-1)+0.25(1-\Phi)\Phi M_{CcBbAa}(i-1)\Phi\Phi F_{CCBbAa}(i-1)+0.25(1-\Phi)\Phi M_{CcBbAa}(i-1)(1-\Phi)F_{CCBbAA}(i-1)+0.25(1-\Phi)\Phi M_{CcBbAa}(i-1)\Phi F_{CCBbAA}(i-1)+0.125(1-\Phi)\Phi M_{CcBbAa}(i-1)(1-\Phi)F_{CCBBAa}(i-1)+0.25(1-\Phi)\Phi M_{CcBbAa}(i-1)\Phi F_{CCBBAa}(i-1)+0.25(1-\Phi)\Phi M_{CcBbAa}(i-1)F_{CCBBAA}(i-1)+0.25\Phi\Phi M_{CcBbAa}(i-1)F_{ccbbAa}(i-1)+0.5\Phi\Phi M_{CcBbAa}(i-1)F_{ccbbAA}(i-1)+0.125\Phi\Phi M_{CcBbAa}(i-1)(1-\Phi)F_{ccBbAa}(i-1)+0.25\Phi\Phi M_{CcBbAa}(i-1)\Phi F_{ccBbAa}(i-1)+0.25\Phi\Phi M_{CcBbAa}(i-1)F_{ccBbAA}(i-1)+0.25\Phi\Phi M_{CcBbAa}(i-1)F_{CcbbAa}(i-1)+0.5\Phi\Phi M_{CcBbAa}(i-1)F_{CcbbAA}(i-1)+0.125\Phi\Phi M_{CcBbAa}(i-1)(1-\Phi)(1-\Phi)F_{CcBbAa}(i-1)+0.25\Phi\Phi M_{CcBbAa}(i-1)(1-\Phi)\Phi F_{CcBbAa}(i-1)+0.25\Phi\Phi M_{CcBbAa}(i-1)(1-\Phi)F_{CcBbAA}(i-1)+0.25\Phi\Phi M_{CcBbAa}(i-1)F_{CCbbAa}(i-1)+0.5\Phi\Phi M_{CcBbAa}(i-1)F_{CCbbAA}(i-1)+0.125\Phi\Phi M_{CcBbAa}(i-1)(1-\Phi)(1-\Phi)F_{CCBbAa}(i-1)+0.25\Phi\Phi M_{CcBbAa}(i-1)(1-\Phi)F_{CCBbAA}(i-1)+0.125(1-\Phi)M_{CcBbAA}(i-1)F_{ccbbAa}(i-1)+0.25(1-\Phi)M_{CcBbAA}(i-1)F_{ccbbAA}(i-1)+0.125(1-\Phi)M_{CcBbAA}(i-1)(1-\Phi)F_{ccBbAa}(i-1)+0.25(1-\Phi)M_{CcBbAA}(i-1)\Phi F_{ccBbAa}(i-1)+0.25(1-\Phi)M_{CcBbAA}(i-1)F_{ccBbAA}(i-1)+0.125(1-\Phi)M_{CcBbAA}(i-1)(1-\Phi)F_{ccBBAa}(i-1)+0.25(1-\Phi)M_{CcBbAA}(i-1)\Phi F_{ccBBAa}(i-1)+0.25(1-\Phi)M_{CcBbAA}(i-1)F_{ccBBAA}(i-1)+0.125(1-\Phi)M_{CcBbAA}(i-1)F_{CcbbAa}(i-1)+0.25(1-\Phi)M_{CcBbAA}(i-1)F_{CcbbAA}(i-1)+0.125(1-\Phi)M_{CcBbAA}(i-1)(1-\Phi)(1-\Phi)F_{CcBbAa}(i-1)+0.125(1-\Phi)M_{CcBbAA}(i-1)\Phi(1-\Phi)F_{CcBbAa}(i-1)+0.25(1-\Phi)M_{CcBbAA}(i-1)(1-\Phi)\Phi F_{CcBbAa}(i-1)+0.25(1-\Phi)M_{CcBbAA}(i-1)\Phi\Phi F_{CcBbAa}(i-1)+0.25(1-\Phi)M_{CcBbAA}(i-1)(1-\Phi)F_{CcBbAA}(i-1)+0.25(1-\Phi)M_{CcBbAA}(i-1)\Phi F_{CcBbAA}(i-1)+0.125(1-\Phi)M_{CcBbAA}(i-1)(1-\Phi)F_{CcBBAa}(i-1)+0.25(1-\Phi)M_{CcBbAA}(i-1)\Phi F_{CcBBAa}(i-1)+0.25(1-\Phi)M_{CcBbAA}(i-1)F_{CcBBAA}(i-1)+0.125(1-\Phi)M_{CcBbAA}(i-1)F_{CCbbAa}(i-1)+0.25(1-\Phi)M_{CcBbAA}(i-1)F_{CCbbAA}(i-1)+0.125(1-\Phi)M_{CcBbAA}(i-1)(1-\Phi)(1-\Phi)F_{CCBbAa}(i-1)+0.125(1-\Phi)M_{CcBbAA}(i-1)\Phi(1-\Phi)F_{CCBbAa}(i-1)+0.125(1-\Phi)M_{CcBbAA}(i-1)(1-\Phi)\Phi F_{CCBbAa}(i-1)+0.25(1-\Phi)M_{CcBbAA}(i-1)\Phi\Phi F_{CCBbAa}(i-1)+0.25(1-\Phi)M_{CcBbAA}(i-1)(1-\Phi)F_{CCBbAA}(i-1)+0.25(1-\Phi)M_{CcBbAA}(i-1)\Phi F_{CCBbAA}(i-1)+0.125(1-\Phi)M_{CcBbAA}(i-1)(1-\Phi)F_{CCBBAa}(i-1)+0.25(1-\Phi)M_{CcBbAA}(i-1)\Phi F_{CCBBAa}(i-1)+0.25(1-\Phi)M_{CcBbAA}(i-1)F_{CCBBAA}(i-1)+0.25\Phi M_{CcBbAA}(i-1)F_{ccbbAa}(i-1)+0.5\Phi M_{CcBbAA}(i-1)F_{ccbbAA}(i-1)+0.125\Phi M_{CcBbAA}(i-1)(1-\Phi)F_{ccBbAa}(i-1)+0.25\Phi M_{CcBbAA}(i-1)\Phi F_{ccBbAa}(i-1)+0.25\Phi M_{CcBbAA}(i-1)F_{ccBbAA}(i-1)+0.25\Phi M_{CcBbAA}(i-1)F_{CcbbAa}(i-1)+0.5\Phi M_{CcBbAA}(i-1)F_{CcbbAA}(i-1)+0.125\Phi M_{CcBbAA}(i-1)(1-\Phi)(1-\Phi)F_{CcBbAa}(i-1)+0.25\Phi M_{CcBbAA}(i-1)(1-\Phi)\Phi F_{CcBbAa}(i-1)+0.25\Phi M_{CcBbAA}(i-1)(1-\Phi)F_{CcBbAA}(i-1)+0.25\Phi M_{CcBbAA}(i-1)F_{CCbbAa}(i-1)+0.5\Phi M_{CcBbAA}(i-1)F_{CCbbAA}(i-1)+0.125\Phi M_{CcBbAA}(i-1)(1-\Phi)(1-\Phi)F_{CCBbAa}(i-1)+0.25\Phi M_{CcBbAA}(i-1)(1-\Phi)F_{CCBbAA}(i-1)+0.125(1-\Phi)M_{CcBBAa}(i-1)F_{ccbbAa}(i-1)+0.25(1-\Phi)M_{CcBBAa}(i-1)F_{ccbbAA}(i-1)+0.0625(1-\Phi)M_{CcBBAa}(i-1)(1-\Phi)F_{ccBbAa}(i-1)+0.125(1-\Phi)M_{CcBBAa}(i-1)\Phi F_{ccBbAa}(i-1)+0.125(1-\Phi)M_{CcBBAa}(i-1)F_{ccBbAA}(i-1)+0.125(1-\Phi)M_{CcBBAa}(i-1)F_{CcbbAa}(i-1)+0.25(1-\Phi)M_{CcBBAa}(i-1)F_{CcbbAA}(i-1)+0.0625(1-\Phi)M_{CcBBAa}(i-1)(1-\Phi)(1-\Phi)F_{CcBbAa}(i-1)+0.125(1-\Phi)M_{CcBBAa}(i-1)(1-\Phi)\Phi F_{CcBbAa}(i-1)+0.125(1-\Phi)M_{CcBBAa}(i-1)(1-\Phi)F_{CcBbAA}(i-1)+0.125(1-\Phi)M_{CcBBAa}(i-1)F_{CCbbAa}(i-1)+0.25(1-\Phi)M_{CcBBAa}(i-1)F_{CCbbAA}(i-1)+0.0625(1-\Phi)M_{CcBBAa}(i-1)(1-\Phi)(1-\Phi)F_{CCBbAa}(i-1)+0.125(1-\Phi)M_{CcBBAa}(i-1)(1-\Phi)F_{CCBbAA}(i-1)+0.25\Phi M_{CcBBAa}(i-1)F_{ccbbAa}(i-1)+0.5\Phi M_{CcBBAa}(i-1)F_{ccbbAA}(i-1)+0.125\Phi M_{CcBBAa}(i-1)(1-\Phi)F_{ccBbAa}(i-1)+0.25\Phi M_{CcBBAa}(i-1)\Phi F_{ccBbAa}(i-1)+0.25\Phi M_{CcBBAa}(i-1)F_{ccBbAA}(i-1)+0.25\Phi M_{CcBBAa}(i-1)F_{CcbbAa}(i-1)+0.5\Phi M_{CcBBAa}(i-1)F_{CcbbAA}(i-1)+0.125\Phi M_{CcBBAa}(i-1)(1-\Phi)(1-\Phi)F_{CcBbAa}(i-1)+0.25\Phi M_{CcBBAa}(i-1)(1-\Phi)\Phi F_{CcBbAa}(i-1)+0.25\Phi M_{CcBBAa}(i-1)(1-\Phi)F_{CcBbAA}(i-1)+0.25\Phi M_{CcBBAa}(i-1)F_{CCbbAa}(i-1)+0.5\Phi M_{CcBBAa}(i-1)F_{CCbbAA}(i-1)+0.125\Phi M_{CcBBAa}(i-$$

$1)(1-\Phi)(1-\Phi)F_{CCBbAa}(i-1)+0.25\Phi M_{CcBBAa}(i-1)(1-\Phi)F_{CCBbAA}(i-1)+0.25M_{CcBBAA}(i-$
$1)F_{ccbbAa}(i-1)+0.5M_{CcBBAA}(i-1)F_{ccbbAA}(i-1)+0.125M_{CcBBAA}(i-1)(1-\Phi)F_{ccBbAa}(i-1)+$
$0.25M_{CcBBAA}(i-1)\Phi F_{ccBbAa}(i-1)+0.25M_{CcBBAA}(i-1)F_{ccBbAA}(i-1)+0.25M_{CcBBAA}(i-1)F_{CcbbAa}(i-$
$1)+0.5M_{CcBBAA}(i-1)F_{CcbbAA}(i-1)+0.125M_{CcBBAA}(i-1)(1-\Phi)(1-\Phi)F_{CcBbAa}(i-1)+0.25M_{CcBBAA}(i-$
$1)(1-\Phi)\Phi F_{CcBbAa}(i-1)+0.25M_{CcBBAA}(i-1)(1-\Phi)F_{CcBbAA}(i-1)+0.25M_{CcBBAA}(i-1)F_{CCbbAa}(i-1)+$
$0.5M_{CcBBAA}(i-1)F_{CCbbAA}(i-1)+0.125M_{CcBBAA}(i-1)(1-\Phi)(1-\Phi)F_{CCBbAa}(i-1)+0.25M_{CcBBAA}(i-$
$1)(1-\Phi)F_{CCBbAA}(i-1)+0.125M_{CCbbAa}(i-1)(1-\Phi)F_{ccBbAa}(i-1)+0.25M_{CCbbAa}(i-1)\Phi F_{ccBbAa}(i-$
$1)+0.25M_{CCbbAa}(i-1)F_{ccBbAA}(i-1)+0.25M_{CCbbAa}(i-1)(1-\Phi)F_{ccBBAa}(i-1)+0.5M_{CCbbAa}(i-$
$1)\Phi F_{ccBBAa}(i-1)+0.5M_{CCbbAa}(i-1)F_{ccBBAA}(i-1)+0.0625M_{CCbbAa}(i-1)(1-\Phi)(1-\Phi)F_{CcBbAa}(i-1)+$
$0.125M_{CCbbAa}(i-1)\Phi(1-\Phi)F_{CcBbAa}(i-1)+0.125M_{CCbbAa}(i-1)(1-\Phi)\Phi F_{CcBbAa}(i-1)+0.25M_{CCbbAa}(i-$
$1)\Phi\Phi F_{CcBbAa}(i-1)+0.125M_{CCbbAa}(i-1)(1-\Phi)F_{CcBbAA}(i-1)+0.25M_{CCbbAa}(i-1)\Phi F_{CcBbAA}(i-1)+$
$0.125M_{CCbbAa}(i-1)(1-\Phi)F_{CcBBAa}(i-1)+0.25M_{CCbbAa}(i-1)\Phi F_{CcBBAa}(i-1)+0.25M_{CCbbAa}(i-$
$1)F_{CcBBAA}(i-1)+0.25M_{CCbbAA}(i-1)(1-\Phi)F_{ccBbAa}(i-1)+0.5M_{CCbbAA}(i-1)\Phi F_{ccBbAa}(i-1)+$
$0.5M_{CCbbAA}(i-1)F_{ccBbAA}(i-1)+0.5M_{CCbbAA}(i-1)(1-\Phi)F_{ccBBAa}(i-1)+M_{CCbbAA}(i-1)\Phi F_{ccBBAa}(i-$
$1)+M_{CCbbAA}(i-1)F_{ccBBAA}(i-1)+0.125M_{CCbbAA}(i-1)(1-\Phi)(1-\Phi)F_{CcBbAa}(i-1)+0.25M_{CCbbAA}(i-$
$1)\Phi(1-\Phi)F_{CcBbAa}(i-1)+0.25M_{CCbbAA}(i-1)(1-\Phi)\Phi F_{CcBbAa}(i-1)+0.5M_{CCbbAA}(i-1)\Phi\Phi F_{CcBbAa}(i-$
$1)+0.25M_{CCbbAA}(i-1)(1-\Phi)F_{CcBbAA}(i-1)+0.5M_{CCbbAA}(i-1)\Phi F_{CcBbAA}(i-1)+0.25M_{CCbbAA}(i-$
$1)(1-\Phi)F_{CcBBAa}(i-1)+0.5M_{CCbbAA}(i-1)\Phi F_{CcBBAa}(i-1)+0.5M_{CCbbAA}(i-1)F_{CcBBAA}(i-1)+$
$0.125(1-\Phi)(1-\Phi)M_{CCBbAa}(i-1)F_{ccbbAa}(i-1)+0.25(1-\Phi)(1-\Phi)M_{CCBbAa}(i-1)F_{ccbbAA}(i-1)+$
$0.125(1-\Phi)(1-\Phi)M_{CCBbAa}(i-1)(1-\Phi)F_{ccBbAa}(i-1)+0.25(1-\Phi)(1-\Phi)M_{CCBbAa}(i-1)\Phi F_{ccBbAa}(i-$
$1)+0.25(1-\Phi)(1-\Phi)M_{CCBbAa}(i-1)F_{ccBbAA}(i-1)+0.125(1-\Phi)(1-\Phi)M_{CCBbAa}(i-1)(1-\Phi)F_{ccBBAa}(i-$
$1)+0.25(1-\Phi)(1-\Phi)M_{CCBbAa}(i-1)\Phi F_{ccBBAa}(i-1)+0.25(1-\Phi)(1-\Phi)M_{CCBbAa}(i-1)F_{ccBBAA}(i-$
$1)+0.0625(1-\Phi)(1-\Phi)M_{CCBbAa}(i-1)F_{CcbbAa}(i-1)+0.125(1-\Phi)(1-\Phi)M_{CCBbAa}(i-1)F_{CcbbAA}(i-$
$1)+0.0625(1-\Phi)(1-\Phi)M_{CCBbAa}(i-1)(1-\Phi)(1-\Phi)F_{CcBbAa}(i-1)+0.0625(1-\Phi)(1-\Phi)M_{CCBbAa}(i-$
$1)\Phi(1-\Phi)F_{CcBbAa}(i-1)+0.125(1-\Phi)(1-\Phi)M_{CCBbAa}(i-1)(1-\Phi)\Phi F_{CcBbAa}(i-1)+0.125(1-$
$\Phi)(1-\Phi)M_{CCBbAa}(i-1)\Phi\Phi F_{CcBbAa}(i-1)+0.125(1-\Phi)(1-\Phi)M_{CCBbAa}(i-1)(1-\Phi)F_{CcBbAA}(i-$
$1)+0.125(1-\Phi)(1-\Phi)M_{CCBbAa}(i-1)\Phi F_{CcBbAA}(i-1)+0.0625(1-\Phi)(1-\Phi)M_{CCBbAa}(i-1)(1-$
$\Phi)F_{CcBBAa}(i-1)+0.125(1-\Phi)(1-\Phi)M_{CCBbAa}(i-1)\Phi F_{CcBBAa}(i-1)+0.125(1-\Phi)(1-\Phi)M_{CCBbAa}(i-$
$1)F_{CcBBAA}(i-1)+0.25\Phi(1-\Phi)M_{CCBbAa}(i-1)F_{ccbbAa}(i-1)+0.5\Phi(1-\Phi)M_{CCBbAa}(i-1)F_{ccbbAA}(i-$
$1)+0.125\Phi(1-\Phi)M_{CCBbAa}(i-1)(1-\Phi)F_{ccBbAa}(i-1)+0.25\Phi(1-\Phi)M_{CCBbAa}(i-1)\Phi F_{ccBbAa}(i-1)+$
$0.25\Phi(1-\Phi)M_{CCBbAa}(i-1)F_{ccBbAA}(i-1)+0.125\Phi(1-\Phi)M_{CCBbAa}(i-1)F_{CcbbAa}(i-1)+0.25\Phi(1-$
$\Phi)M_{CCBbAa}(i-1)F_{CcbbAA}(i-1)+0.0625\Phi(1-\Phi)M_{CCBbAa}(i-1)(1-\Phi)(1-\Phi)F_{CcBbAa}(i-1)+0.125\Phi(1-$
$\Phi)M_{CCBbAa}(i-1)(1-\Phi)\Phi F_{CcBbAa}(i-1)+0.125\Phi(1-\Phi)M_{CCBbAa}(i-1)(1-\Phi)F_{CcBbAA}(i-1)+0.25(1-$
$\Phi)\Phi M_{CCBbAa}(i-1)F_{ccbbAa}(i-1)+0.5(1-\Phi)\Phi M_{CCBbAa}(i-1)F_{ccbbAA}(i-1)+0.25(1-\Phi)\Phi M_{CCBbAa}(i-$
$1)(1-\Phi)F_{ccBbAa}(i-1)+0.5(1-\Phi)\Phi M_{CCBbAa}(i-1)\Phi F_{ccBbAa}(i-1)+0.5(1-\Phi)\Phi M_{CCBbAa}(i-1)F_{ccBbAA}(i-$
$1)+0.25(1-\Phi)\Phi M_{CCBbAa}(i-1)(1-\Phi)F_{ccBBAa}(i-1)+0.5(1-\Phi)\Phi M_{CCBbAa}(i-1)\Phi F_{ccBBAa}(i-1)+$
$0.5(1-\Phi)\Phi M_{CCBbAa}(i-1)F_{ccBBAA}(i-1)+0.125(1-\Phi)\Phi M_{CCBbAa}(i-1)F_{CcbbAa}(i-1)+0.25(1-$
$\Phi)\Phi M_{CCBbAa}(i-1)F_{CcbbAA}(i-1)+0.125(1-\Phi)\Phi M_{CCBbAa}(i-1)(1-\Phi)(1-\Phi)F_{CcBbAa}(i-1)+0.125(1-$
$\Phi)\Phi M_{CCBbAa}(i-1)\Phi(1-\Phi)F_{CcBbAa}(i-1)+0.25(1-\Phi)\Phi M_{CCBbAa}(i-1)(1-\Phi)\Phi F_{CcBbAa}(i-1)+0.25(1-$
$\Phi)\Phi M_{CCBbAa}(i-1)\Phi\Phi F_{CcBbAa}(i-1)+0.25(1-\Phi)\Phi M_{CCBbAa}(i-1)(1-\Phi)F_{CcBbAA}(i-1)+0.25(1-$
$\Phi)\Phi M_{CCBbAa}(i-1)\Phi F_{CcBbAA}(i-1)+0.125(1-\Phi)\Phi M_{CCBbAa}(i-1)(1-\Phi)F_{CcBBAa}(i-1)+0.25(1-$
$\Phi)\Phi M_{CCBbAa}(i-1)\Phi F_{CcBBAa}(i-1)+0.25(1-\Phi)\Phi M_{CCBbAa}(i-1)F_{CcBBAA}(i-1)+0.5\Phi\Phi M_{CCBbAa}(i-$
$1)F_{ccbbAa}(i-1)+\Phi\Phi M_{CCBbAa}(i-1)F_{ccbbAA}(i-1)+0.25\Phi\Phi M_{CCBbAa}(i-1)(1-\Phi)F_{ccBbAa}(i-1)+$
$0.5\Phi\Phi M_{CCBbAa}(i-1)\Phi F_{ccBbAa}(i-1)+0.5\Phi\Phi M_{CCBbAa}(i-1)F_{ccBbAA}(i-1)+0.25\Phi\Phi M_{CCBbAa}(i-$
$1)F_{CcbbAa}(i-1)+0.5\Phi\Phi M_{CCBbAa}(i-1)F_{CcbbAA}(i-1)+0.125\Phi\Phi M_{CCBbAa}(i-1)(1-\Phi)(1-\Phi)F_{CcBbAa}(i-$
$1)+0.25\Phi\Phi M_{CCBbAa}(i-1)(1-\Phi)\Phi F_{CcBbAa}(i-1)+0.25\Phi\Phi M_{CCBbAa}(i-1)(1-\Phi)F_{CcBbAA}(i-1)+0.25(1-$
$\Phi)M_{CCBbAA}(i-1)F_{ccbbAa}(i-1)+0.5(1-\Phi)M_{CCBbAA}(i-1)F_{ccbbAA}(i-1)+0.25(1-\Phi)M_{CCBbAA}(i-1)(1-$
$\Phi)F_{ccBbAa}(i-1)+0.5(1-\Phi)M_{CCBbAA}(i-1)\Phi F_{ccBbAa}(i-1)+0.5(1-\Phi)M_{CCBbAA}(i-1)F_{ccBbAA}(i-1)+$
$0.25(1-\Phi)M_{CCBbAA}(i-1)(1-\Phi)F_{ccBBAa}(i-1)+0.5(1-\Phi)M_{CCBbAA}(i-1)\Phi F_{ccBBAa}(i-1)+0.5(1-$
$\Phi)M_{CCBbAA}(i-1)F_{ccBBAA}(i-1)+0.125(1-\Phi)M_{CCBbAA}(i-1)F_{CcbbAa}(i-1)+0.25(1-\Phi)M_{CCBbAA}(i-$
$1)F_{CcbbAA}(i-1)+0.125(1-\Phi)M_{CCBbAA}(i-1)(1-\Phi)(1-\Phi)F_{CcBbAa}(i-1)+0.125(1-\Phi)M_{CCBbAA}(i-$
$1)\Phi(1-\Phi)F_{CcBbAa}(i-1)+0.25(1-\Phi)M_{CCBbAA}(i-1)(1-\Phi)\Phi F_{CcBbAa}(i-1)+0.25(1-\Phi)M_{CCBbAA}(i-$
$1)\Phi\Phi F_{CcBbAa}(i-1)+0.25(1-\Phi)M_{CCBbAA}(i-1)(1-\Phi)F_{CcBbAA}(i-1)+0.25(1-\Phi)M_{CCBbAA}(i-$
$1)\Phi F_{CcBbAA}(i-1)+0.125(1-\Phi)M_{CCBbAA}(i-1)(1-\Phi)F_{CcBBAa}(i-1)+0.25(1-\Phi)M_{CCBbAA}(i-$
$1)\Phi F_{CcBBAa}(i-1)+0.25(1-\Phi)M_{CCBbAA}(i-1)F_{CcBBAA}(i-1)+0.5\Phi M_{CCBbAA}(i-1)F_{ccbbAa}(i-$
$1)+\Phi M_{CCBbAA}(i-1)F_{ccbbAA}(i-1)+0.25\Phi M_{CCBbAA}(i-1)(1-\Phi)F_{ccBbAa}(i-1)+0.5\Phi M_{CCBbAA}(i-$
$1)\Phi F_{ccBbAa}(i-1)+0.5\Phi M_{CCBbAA}(i-1)F_{ccBbAA}(i-1)+0.25\Phi M_{CCBbAA}(i-1)F_{CcbbAa}(i-1)+0.5\Phi M_{CCBbAA}(i-$

$$1)F_{CcbbAA}(i-1)+0.125\Phi M_{CCBbAA}(i-1)(1-\Phi)(1-\Phi)F_{CcBbAa}(i-1)+0.25\Phi M_{CCBbAA}(i-1)(1-\Phi)\Phi F_{CcBbAa}(i-1)+0.25\Phi M_{CCBbAA}(i-1)(1-\Phi)F_{CcBbAA}(i-1)+0.25(1-\Phi)M_{CCBBAa}(i-1)F_{ccbbAa}(i-1)+0.5(1-\Phi)M_{CCBBAa}(i-1)F_{ccbbAA}(i-1)+0.125(1-\Phi)M_{CCBBAa}(i-1)(1-\Phi)F_{ccBbAa}(i-1)+0.25(1-\Phi)M_{CCBBAa}(i-1)\Phi F_{ccBbAa}(i-1)+0.25(1-\Phi)M_{CCBBAa}(i-1)F_{ccBbAA}(i-1)+0.125(1-\Phi)M_{CCBBAa}(i-1)F_{CcbbAa}(i-1)+0.25(1-\Phi)M_{CCBBAa}(i-1)F_{CcbbAA}(i-1)+0.0625(1-\Phi)M_{CCBBAa}(i-1)(1-\Phi)(1-\Phi)F_{CcBbAa}(i-1)+0.125(1-\Phi)M_{CCBBAa}(i-1)(1-\Phi)\Phi F_{CcBbAa}(i-1)+0.125(1-\Phi)M_{CCBBAa}(i-1)(1-\Phi)F_{CcBbAA}(i-1)+0.5\Phi M_{CCBBAa}(i-1)F_{ccbbAa}(i-1)+\Phi M_{CCBBAa}(i-1)F_{ccbbAA}(i-1)+0.25\Phi M_{CCBBAa}(i-1)(1-\Phi)F_{ccBbAa}(i-1)+0.5\Phi M_{CCBBAa}(i-1)\Phi F_{ccBbAa}(i-1)+0.5\Phi M_{CCBBAa}(i-1)F_{ccBbAA}(i-1)+0.25\Phi M_{CCBBAa}(i-1)F_{CcbbAa}(i-1)+0.5\Phi M_{CCBBAa}(i-1)F_{CcbbAA}(i-1)+0.125\Phi M_{CCBBAa}(i-1)(1-\Phi)(1-\Phi)F_{CcBbAa}(i-1)+0.25\Phi M_{CCBBAa}(i-1)(1-\Phi)\Phi F_{CcBbAa}(i-1)+0.25\Phi M_{CCBBAa}(i-1)(1-\Phi)F_{CcBbAA}(i-1)+0.5M_{CCBBAA}(i-1)F_{ccbbAa}(i-1)+M_{CCBBAA}(i-1)F_{ccbbAA}(i-1)+0.25M_{CCBBAA}(i-1)(1-\Phi)F_{ccBbAa}(i-1)+0.5M_{CCBBAA}(i-1)\Phi F_{ccBbAa}(i-1)+0.5M_{CCBBAA}(i-1)F_{ccBbAA}(i-1)+0.25M_{CCBBAA}(i-1)F_{CcbbAa}(i-1)+0.5M_{CCBBAA}(i-1)F_{CcbbAA}(i-1)+0.125M_{CCBBAA}(i-1)(1-\Phi)(1-\Phi)F_{CcBbAa}(i-1)+0.25M_{CCBBAA}(i-1)(1-\Phi)\Phi F_{CcBbAa}(i-1)+0.25M_{CCBBAA}(i-1)(1-\Phi)F_{CcBbAA}(i-1)],$$

$$\mathrm{M}^e_{CcBBaa}=\left(\frac{\bar{\Omega}_{CcBBaa}}{2}\right)[0.125M_{ccBbaa}(i-1)(1-\Phi)F_{CcBbaa}(i-1)+0.25M_{ccBbaa}(i-1)\Phi F_{CcBbaa}(i-1)+0.0625M_{ccBbaa}(i-1)(1-\Phi)(1-\Phi)F_{CcBbAa}(i-1)+0.125M_{ccBbaa}(i-1)\Phi(1-\Phi)F_{CcBbAa}(i-1)+0.25M_{ccBbaa}(i-1)F_{CcBBaa}(i-1)+0.125M_{ccBbaa}(i-1)(1-\Phi)F_{CcBBAa}(i-1)+0.25M_{ccBbaa}(i-1)(1-\Phi)F_{CCBbaa}(i-1)+0.5M_{ccBbaa}(i-1)\Phi F_{CCBbaa}(i-1)+0.125M_{ccBbaa}(i-1)(1-\Phi)(1-\Phi)F_{CCBbAa}(i-1)+0.25M_{ccBbaa}(i-1)\Phi(1-\Phi)F_{CCBbAa}(i-1)+0.25M_{ccBbaa}(i-1)(1-\Phi)\Phi F_{CCBbAa}(i-1)+0.5M_{ccBbaa}(i-1)F_{CCBBaa}(i-1)+0.25M_{ccBbaa}(i-1)(1-\Phi)F_{CCBBAa}(i-1)+0.0625(1-\Phi)M_{ccBbAa}(i-1)(1-\Phi)F_{CcBbaa}(i-1)+0.125(1-\Phi)M_{ccBbAa}(i-1)\Phi F_{CcBbaa}(i-1)+0.03125(1-\Phi)M_{ccBbAa}(i-1)(1-\Phi)(1-\Phi)F_{CcBbAa}(i-1)+0.0625(1-\Phi)M_{ccBbAa}(i-1)\Phi(1-\Phi)F_{CcBbAa}(i-1)+0.125(1-\Phi)M_{ccBbAa}(i-1)F_{CcBBaa}(i-1)+0.0625(1-\Phi)M_{ccBbAa}(i-1)(1-\Phi)F_{CcBBAa}(i-1)+0.125(1-\Phi)M_{ccBbAa}(i-1)(1-\Phi)F_{CCBbaa}(i-1)+0.25(1-\Phi)M_{ccBbAa}(i-1)\Phi F_{CCBbaa}(i-1)+0.0625(1-\Phi)M_{ccBbAa}(i-1)(1-\Phi)(1-\Phi)F_{CCBbAa}(i-1)+0.125(1-\Phi)M_{ccBbAa}(i-1)\Phi(1-\Phi)F_{CCBbAa}(i-1)+0.125(1-\Phi)M_{ccBbAa}(i-1)(1-\Phi)\Phi F_{CCBbAa}(i-1)+0.25(1-\Phi)M_{ccBbAa}(i-1)F_{CCBBaa}(i-1)+0.125(1-\Phi)M_{ccBbAa}(i-1)(1-\Phi)F_{CCBBAa}(i-1)+0.25M_{ccBBaa}(i-1)(1-\Phi)F_{CcBbaa}(i-1)+0.5M_{ccBBaa}(i-1)\Phi F_{CcBbaa}(i-1)+0.125M_{ccBBaa}(i-1)(1-\Phi)(1-\Phi)F_{CcBbAa}(i-1)+0.25M_{ccBBaa}(i-1)\Phi(1-\Phi)F_{CcBbAa}(i-1)+0.5M_{ccBBaa}(i-1)F_{CcBBaa}(i-1)+0.25M_{ccBBaa}(i-1)(1-\Phi)F_{CcBBAa}(i-1)+0.5M_{ccBBaa}(i-1)(1-\Phi)F_{CCBbaa}(i-1)+M_{ccBBaa}(i-1)\Phi F_{CCBbaa}(i-1)+0.25M_{ccBBaa}(i-1)(1-\Phi)(1-\Phi)F_{CCBbAa}(i-1)+0.5M_{ccBBaa}(i-1)\Phi(1-\Phi)F_{CCBbAa}(i-1)+0.5M_{ccBBaa}(i-1)(1-\Phi)\Phi F_{CCBbAa}(i-1)+M_{ccBBaa}(i-1)F_{CCBBaa}(i-1)+0.5M_{ccBBaa}(i-1)(1-\Phi)F_{CCBBAa}(i-1)+0.125(1-\Phi)M_{ccBBAa}(i-1)(1-\Phi)F_{CcBbaa}(i-1)+0.25(1-\Phi)M_{ccBBAa}(i-1)\Phi F_{CcBbaa}(i-1)+0.0625(1-\Phi)M_{ccBBAa}(i-1)(1-\Phi)(1-\Phi)F_{CcBbAa}(i-1)+0.125(1-\Phi)M_{ccBBAa}(i-1)\Phi(1-\Phi)F_{CcBbAa}(i-1)+0.25(1-\Phi)M_{ccBBAa}(i-1)F_{CcBBaa}(i-1)+0.125(1-\Phi)M_{ccBBAa}(i-1)(1-\Phi)F_{CcBBAa}(i-1)+0.25(1-\Phi)M_{ccBBAa}(i-1)(1-\Phi)F_{CCBbaa}(i-1)+0.5(1-\Phi)M_{ccBBAa}(i-1)\Phi F_{CCBbaa}(i-1)+0.125(1-\Phi)M_{ccBBAa}(i-1)(1-\Phi)(1-\Phi)F_{CCBbAa}(i-1)+0.25(1-\Phi)M_{ccBBAa}(i-1)\Phi(1-\Phi)F_{CCBbAa}(i-1)+0.25(1-\Phi)M_{ccBBAa}(i-1)(1-\Phi)\Phi F_{CCBbAa}(i-1)+0.5(1-\Phi)M_{ccBBAa}(i-1)F_{CCBBaa}(i-1)+0.25(1-\Phi)M_{ccBBAa}(i-1)(1-\Phi)F_{CCBBAa}(i-1)+0.125(1-\Phi)M_{CcBbaa}(i-1)F_{ccBbaa}(i-1)+0.0625(1-\Phi)M_{CcBbaa}(i-1)(1-\Phi)F_{ccBbAa}(i-1)+0.25(1-\Phi)M_{CcBbaa}(i-1)F_{ccBBaa}(i-1)+0.125(1-\Phi)M_{CcBbaa}(i-1)(1-\Phi)F_{ccBBAa}(i-1)+0.125(1-\Phi)M_{CcBbaa}(i-1)(1-\Phi)F_{CcBbaa}(i-1)+0.25(1-\Phi)M_{CcBbaa}(i-1)\Phi F_{CcBbaa}(i-1)+0.0625(1-\Phi)M_{CcBbaa}(i-1)(1-\Phi)(1-\Phi)F_{CcBbAa}(i-1)+0.125(1-\Phi)M_{CcBbaa}(i-1)\Phi(1-\Phi)F_{CcBbAa}(i-1)+0.25(1-\Phi)M_{CcBbaa}(i-1)F_{CcBBaa}(i-1)+0.125(1-\Phi)M_{CcBbaa}(i-1)(1-\Phi)F_{CcBBAa}(i-1)+0.125(1-\Phi)M_{CcBbaa}(i-1)(1-\Phi)F_{CCBbaa}(i-1)+0.25(1-\Phi)M_{CcBbaa}(i-1)\Phi F_{CCBbaa}(i-1)+0.0625(1-\Phi)M_{CcBbaa}(i-1)(1-\Phi)(1-\Phi)F_{CCBbAa}(i-1)+0.125(1-\Phi)M_{CcBbaa}(i-1)\Phi(1-\Phi)F_{CCBbAa}(i-1)+0.125(1-\Phi)M_{CcBbaa}(i-1)(1-\Phi)\Phi F_{CCBbAa}(i-1)+0.25(1-\Phi)M_{CcBbaa}(i-1)F_{CCBBaa}(i-1)+0.125(1-\Phi)M_{CcBbaa}(i-1)(1-\Phi)F_{CCBBAa}(i-1)+0.25\Phi M_{CcBbaa}(i-1)F_{ccBbaa}(i-1)+0.125\Phi M_{CcBbaa}(i-1)(1-\Phi)F_{ccBbAa}(i-1)+0.5\Phi M_{CcBbaa}(i-1)F_{ccBBaa}(i-1)+0.25\Phi M_{CcBbaa}(i-1)(1-\Phi)F_{ccBBAa}(i-1)+0.25\Phi M_{CcBbaa}(i-1)(1-\Phi)F_{CcBbaa}(i-1)+0.5\Phi M_{CcBbaa}(i-1)\Phi F_{CcBbaa}(i-1)+0.125\Phi M_{CcBbaa}(i-1)(1-\Phi)(1-\Phi)F_{CcBbAa}(i-1)+0.25\Phi M_{CcBbaa}(i-1)\Phi(1-\Phi)F_{CcBbAa}(i-1)+0.5\Phi M_{CcBbaa}(i-1)F_{CcBBaa}(i-1)+0.25\Phi M_{CcBbaa}(i-1)(1-\Phi)F_{CcBBAa}(i-1)+0.25\Phi M_{CcBbaa}(i-1)(1-\Phi)F_{CCBbaa}(i-1)+0.5\Phi M_{CcBbaa}(i-1)\Phi F_{CCBbaa}(i-1)+0.125\Phi M_{CcBbaa}(i-1)(1-\Phi)(1-\Phi)F_{CCBbAa}(i-1)+0.25\Phi M_{CcBbaa}(i-1)\Phi(1-\Phi)F_{CCBbAa}(i-1)+0.25\Phi M_{CcBbaa}(i-1)(1-\Phi)\Phi F_{CCBbAa}(i-1)+0.5\Phi M_{CcBbaa}(i-1)F_{CCBBaa}(i-1)+0.25\Phi M_{CcBbaa}(i-1)(1-\Phi)F_{CCBBAa}(i-1)+0.0625(1-\Phi)(1-\Phi)M_{CcBbAa}(i-1)F_{ccBbaa}(i-1)+0.03125(1-\Phi)(1-\Phi)M_{CcBbAa}(i-1)(1-\Phi)F_{ccBbAa}(i-1)+0.125(1-\Phi)(1-\Phi)M_{CcBbAa}(i-1)F_{ccBBaa}(i-1)+0.0625(1-\Phi)(1-\Phi)M_{CcBbAa}(i-1)(1-\Phi)F_{ccBBAa}(i-1)+0.0625(1-\Phi)(1-\Phi)M_{CcBbAa}(i-1)(1-\Phi)F_{CcBbaa}(i-1)+0.125(1-\Phi)(1-$$

$$\Phi)M_{CcBbAa}(i-1)\Phi F_{CcBbaa}(i-1)+0.03125(1-\Phi)(1-\Phi)M_{CcBbAa}(i-1)(1-\Phi)(1-\Phi)F_{CcBbAa}(i-1)+$$
$$0.0625(1-\Phi)(1-\Phi)M_{CcBbAa}(i-1)\Phi(1-\Phi)F_{CcBbAa}(i-1)+0.125(1-\Phi)(1-\Phi)M_{CcBbAa}(i-1)F_{CcBBaa}(i-$$
$$1)+0.0625(1-\Phi)(1-\Phi)M_{CcBbAa}(i-1)(1-\Phi)F_{CcBBAa}(i-1)+0.0625(1-\Phi)(1-\Phi)M_{CcBbAa}(i-1)(1-$$
$$\Phi)F_{CCBbaa}(i-1)+0.125(1-\Phi)(1-\Phi)M_{CcBbAa}(i-1)\Phi F_{CCBbaa}(i-1)+0.03125(1-\Phi)(1-\Phi)M_{CcBbAa}(i-$$
$$1)(1-\Phi)(1-\Phi)F_{CCBbAa}(i-1)+0.0625(1-\Phi)(1-\Phi)M_{CcBbAa}(i-1)\Phi(1-\Phi)F_{CCBbAa}(i-1)+0.0625(1-$$
$$\Phi)(1-\Phi)M_{CcBbAa}(i-1)(1-\Phi)\Phi F_{CCBbAa}(i-1)+0.125(1-\Phi)(1-\Phi)M_{CcBbAa}(i-1)F_{CCBBaa}(i-1)+$$
$$0.0625(1-\Phi)(1-\Phi)M_{CcBbAa}(i-1)(1-\Phi)F_{CCBBAa}(i-1)+0.125\Phi(1-\Phi)M_{CcBbAa}(i-1)F_{ccBbaa}(i-$$
$$1)+0.0625\Phi(1-\Phi)M_{CcBbAa}(i-1)(1-\Phi)F_{ccBbAa}(i-1)+0.25\Phi(1-\Phi)M_{CcBbAa}(i-1)F_{ccBBaa}(i-1)+$$
$$0.125\Phi(1-\Phi)M_{CcBbAa}(i-1)(1-\Phi)F_{ccBBAa}(i-1)+0.125\Phi(1-\Phi)M_{CcBbAa}(i-1)(1-\Phi)F_{CcBbaa}(i-1)+$$
$$0.25\Phi(1-\Phi)M_{CcBbAa}(i-1)\Phi F_{CcBbaa}(i-1)+0.0625\Phi(1-\Phi)M_{CcBbAa}(i-1)(1-\Phi)(1-\Phi)F_{CcBbAa}(i-$$
$$1)+0.125\Phi(1-\Phi)M_{CcBbAa}(i-1)\Phi(1-\Phi)F_{CcBbAa}(i-1)+0.25\Phi(1-\Phi)M_{CcBbAa}(i-1)F_{CcBBaa}(i-1)+$$
$$0.125\Phi(1-\Phi)M_{CcBbAa}(i-1)(1-\Phi)F_{CcBBAa}(i-1)+0.125\Phi(1-\Phi)M_{CcBbAa}(i-1)(1-\Phi)F_{CCBbaa}(i-1)+$$
$$0.25\Phi(1-\Phi)M_{CcBbAa}(i-1)\Phi F_{CCBbaa}(i-1)+0.0625\Phi(1-\Phi)M_{CcBbAa}(i-1)(1-\Phi)(1-\Phi)F_{CCBbAa}(i-1)+$$
$$0.125\Phi(1-\Phi)M_{CcBbAa}(i-1)\Phi(1-\Phi)F_{CCBbAa}(i-1)+0.125\Phi(1-\Phi)M_{CcBbAa}(i-1)(1-\Phi)\Phi F_{CCBbAa}(i-$$
$$1)+0.25\Phi(1-\Phi)M_{CcBbAa}(i-1)F_{CCBBaa}(i-1)+0.125\Phi(1-\Phi)M_{CcBbAa}(i-1)(1-\Phi)F_{CCBBAa}(i-$$
$$1)+0.25M_{CcBBaa}(i-1)F_{ccBbaa}(i-1)+0.125M_{CcBBaa}(i-1)(1-\Phi)F_{ccBbAa}(i-1)+0.5M_{CcBBaa}(i-$$
$$1)F_{ccBBaa}(i-1)+0.25M_{CcBBaa}(i-1)(1-\Phi)F_{ccBBAa}(i-1)+0.25M_{CcBBaa}(i-1)(1-\Phi)F_{CcBbaa}(i-1)+$$
$$0.5M_{CcBBaa}(i-1)\Phi F_{CcBbaa}(i-1)+0.125M_{CcBBaa}(i-1)(1-\Phi)(1-\Phi)F_{CcBbAa}(i-1)+0.25M_{CcBBaa}(i-$$
$$1)\Phi(1-\Phi)F_{CcBbAa}(i-1)+0.5M_{CcBBaa}(i-1)F_{CcBBaa}(i-1)+0.25M_{CcBBaa}(i-1)(1-\Phi)F_{CcBBAa}(i-$$
$$1)+0.25M_{CcBBaa}(i-1)(1-\Phi)F_{CCBbaa}(i-1)+0.5M_{CcBBaa}(i-1)\Phi F_{CCBbaa}(i-1)+0.125M_{CcBBaa}(i-$$
$$1)(1-\Phi)(1-\Phi)F_{CCBbAa}(i-1)+0.25M_{CcBBaa}(i-1)\Phi(1-\Phi)F_{CCBbAa}(i-1)+0.25M_{CcBBaa}(i-1)(1-$$
$$\Phi)\Phi F_{CCBbAa}(i-1)+0.5M_{CcBBaa}(i-1)F_{CCBBaa}(i-1)+0.25M_{CcBBaa}(i-1)(1-\Phi)F_{CCBBAa}(i-$$
$$1)+0.125(1-\Phi)M_{CcBBAa}(i-1)F_{ccBbaa}(i-1)+0.0625(1-\Phi)M_{CcBBAa}(i-1)(1-\Phi)F_{ccBbAa}(i-1)+$$
$$0.25(1-\Phi)M_{CcBBAa}(i-1)F_{ccBBaa}(i-1)+0.125(1-\Phi)M_{CcBBAa}(i-1)(1-\Phi)F_{ccBBAa}(i-1)+0.125(1-$$
$$\Phi)M_{CcBBAa}(i-1)(1-\Phi)F_{CcBbaa}(i-1)+0.25(1-\Phi)M_{CcBBAa}(i-1)\Phi F_{CcBbaa}(i-1)+0.0625(1-$$
$$\Phi)M_{CcBBAa}(i-1)(1-\Phi)(1-\Phi)F_{CcBbAa}(i-1)+0.125(1-\Phi)M_{CcBBAa}(i-1)\Phi(1-\Phi)F_{CcBbAa}(i-$$
$$1)+0.25(1-\Phi)M_{CcBBAa}(i-1)F_{CcBBaa}(i-1)+0.125(1-\Phi)M_{CcBBAa}(i-1)(1-\Phi)F_{CcBBAa}(i-1)+$$
$$0.125(1-\Phi)M_{CcBBAa}(i-1)(1-\Phi)F_{CCBbaa}(i-1)+0.25(1-\Phi)M_{CcBBAa}(i-1)\Phi F_{CCBbaa}(i-1)+0.0625(1-$$
$$\Phi)M_{CcBBAa}(i-1)(1-\Phi)(1-\Phi)F_{CCBbAa}(i-1)+0.125(1-\Phi)M_{CcBBAa}(i-1)\Phi(1-\Phi)F_{CCBbAa}(i-$$
$$1)+0.125(1-\Phi)M_{CcBBAa}(i-1)(1-\Phi)\Phi F_{CCBbAa}(i-1)+0.25(1-\Phi)M_{CcBBAa}(i-1)F_{CCBBaa}(i-1)+$$
$$0.125(1-\Phi)M_{CcBBAa}(i-1)(1-\Phi)F_{CCBBAa}(i-1)+0.25(1-\Phi)M_{CCBbaa}(i-1)F_{ccBbaa}(i-1)+0.125(1-$$
$$\Phi)M_{CCBbaa}(i-1)(1-\Phi)F_{ccBbAa}(i-1)+0.5(1-\Phi)M_{CCBbaa}(i-1)F_{ccBBaa}(i-1)+0.25(1-\Phi)M_{CCBbaa}(i-$$
$$1)(1-\Phi)F_{ccBBAa}(i-1)+0.125(1-\Phi)M_{CCBbaa}(i-1)(1-\Phi)F_{CcBbaa}(i-1)+0.25(1-\Phi)M_{CCBbaa}(i-$$
$$1)\Phi F_{CcBbaa}(i-1)+0.0625(1-\Phi)M_{CCBbaa}(i-1)(1-\Phi)(1-\Phi)F_{CcBbAa}(i-1)+0.125(1-\Phi)M_{CCBbaa}(i-$$
$$1)\Phi(1-\Phi)F_{CcBbAa}(i-1)+0.25(1-\Phi)M_{CCBbaa}(i-1)F_{CcBBaa}(i-1)+0.125(1-\Phi)M_{CCBbaa}(i-$$
$$1)(1-\Phi)F_{CcBBAa}(i-1)+0.5\Phi M_{CCBbaa}(i-1)F_{ccBbaa}(i-1)+0.25\Phi M_{CCBbaa}(i-1)(1-\Phi)F_{ccBbAa}(i-$$
$$1)+\Phi M_{CCBbaa}(i-1)F_{ccBBaa}(i-1)+0.5\Phi M_{CCBbaa}(i-1)(1-\Phi)F_{ccBBAa}(i-1)+0.25\Phi M_{CCBbaa}(i-$$
$$1)(1-\Phi)F_{CcBbaa}(i-1)+0.5\Phi M_{CCBbaa}(i-1)\Phi F_{CcBbaa}(i-1)+0.125\Phi M_{CCBbaa}(i-1)(1-\Phi)(1-$$
$$\Phi)F_{CcBbAa}(i-1)+0.25\Phi M_{CCBbaa}(i-1)\Phi(1-\Phi)F_{CcBbAa}(i-1)+0.5\Phi M_{CCBbaa}(i-1)F_{CcBBaa}(i-1)+$$
$$0.25\Phi M_{CCBbaa}(i-1)(1-\Phi)F_{CcBBAa}(i-1)+0.125(1-\Phi)(1-\Phi)M_{CCBbAa}(i-1)F_{ccBbaa}(i-1)+0.0625(1-$$
$$\Phi)(1-\Phi)M_{CCBbAa}(i-1)(1-\Phi)F_{ccBbAa}(i-1)+0.25(1-\Phi)(1-\Phi)M_{CCBbAa}(i-1)F_{ccBBaa}(i-1)+$$
$$0.125(1-\Phi)(1-\Phi)M_{CCBbAa}(i-1)(1-\Phi)F_{ccBBAa}(i-1)+0.0625(1-\Phi)(1-\Phi)M_{CCBbAa}(i-1)(1-$$
$$\Phi)F_{CcBbaa}(i-1)+0.125(1-\Phi)(1-\Phi)M_{CCBbAa}(i-1)\Phi F_{CcBbaa}(i-1)+0.03125(1-\Phi)(1-\Phi)M_{CCBbAa}(i-$$
$$1)(1-\Phi)(1-\Phi)F_{CcBbAa}(i-1)+0.0625(1-\Phi)(1-\Phi)M_{CCBbAa}(i-1)\Phi(1-\Phi)F_{CcBbAa}(i-1)+0.125(1-$$
$$\Phi)(1-\Phi)M_{CCBbAa}(i-1)F_{CcBBaa}(i-1)+0.0625(1-\Phi)(1-\Phi)M_{CCBbAa}(i-1)(1-\Phi)F_{CcBBAa}(i-$$
$$1)+0.25\Phi(1-\Phi)M_{CCBbAa}(i-1)F_{ccBbaa}(i-1)+0.125\Phi(1-\Phi)M_{CCBbAa}(i-1)(1-\Phi)F_{ccBbAa}(i-$$
$$1)+0.5\Phi(1-\Phi)M_{CCBbAa}(i-1)F_{ccBBaa}(i-1)+0.25\Phi(1-\Phi)M_{CCBbAa}(i-1)(1-\Phi)F_{ccBBAa}(i-1)+$$
$$0.125\Phi(1-\Phi)M_{CCBbAa}(i-1)(1-\Phi)F_{CcBbaa}(i-1)+0.25\Phi(1-\Phi)M_{CCBbAa}(i-1)\Phi F_{CcBbaa}(i-1)+$$
$$0.0625\Phi(1-\Phi)M_{CCBbAa}(i-1)(1-\Phi)(1-\Phi)F_{CcBbAa}(i-1)+0.125\Phi(1-\Phi)M_{CCBbAa}(i-1)\Phi(1-$$
$$\Phi)F_{CcBbAa}(i-1)+0.25\Phi(1-\Phi)M_{CCBbAa}(i-1)F_{CcBBaa}(i-1)+0.125\Phi(1-\Phi)M_{CCBbAa}(i-1)(1-$$
$$\Phi)F_{CcBBAa}(i-1)+0.5M_{CCBBaa}(i-1)F_{ccBbaa}(i-1)+0.25M_{CCBBaa}(i-1)(1-\Phi)F_{ccBbAa}(i-1)+$$
$$M_{CCBBaa}(i-1)F_{ccBBaa}(i-1)+0.5M_{CCBBaa}(i-1)(1-\Phi)F_{ccBBAa}(i-1)+0.25M_{CCBBaa}(i-1)(1-$$
$$\Phi)F_{CcBbaa}(i-1)+0.5M_{CCBBaa}(i-1)\Phi F_{CcBbaa}(i-1)+0.125M_{CCBBaa}(i-1)(1-\Phi)(1-\Phi)F_{CcBbAa}(i-$$
$$1)+0.25M_{CCBBaa}(i-1)\Phi(1-\Phi)F_{CcBbAa}(i-1)+0.5M_{CCBBaa}(i-1)F_{CcBBaa}(i-1)+0.25M_{CCBBaa}(i-$$
$$1)(1-\Phi)F_{CcBBAa}(i-1)+0.25(1-\Phi)M_{CCBBAa}(i-1)F_{ccBbaa}(i-1)+0.125(1-\Phi)M_{CCBBAa}(i-1)(1-$$
$$\Phi)F_{ccBbAa}(i-1)+0.5(1-\Phi)M_{CCBBAa}(i-1)F_{ccBBaa}(i-1)+0.25(1-\Phi)M_{CCBBAa}(i-1)(1-\Phi)F_{ccBBAa}(i-$$
$$1)+0.125(1-\Phi)M_{CCBBAa}(i-1)(1-\Phi)F_{CcBbaa}(i-1)+0.25(1-\Phi)M_{CCBBAa}(i-1)\Phi F_{CcBbaa}(i-1)+$$

$$0.0625(1-\Phi)M_{CCBBAa}(i-1)(1-\Phi)(1-\Phi)F_{CcBbAa}(i-1)+0.125(1-\Phi)M_{CCBBAa}(i-1)\Phi(1-\Phi)F_{CcBbAa}(i-1)+0.25(1-\Phi)M_{CCBBAa}(i-1)F_{CcBBaa}(i-1)+0.125(1-\Phi)M_{CCBBAa}(i-1)(1-\Phi)F_{CcBBAa}(i-1)],$$

$$\mathrm{M}^{e}_{CcBBAa}=\left(\frac{\bar{\Omega}_{CcBBAa}}{2}\right)[0.0625M_{ccBbaa}(i-1)(1-\Phi)(1-\Phi)F_{CcBbAa}(i-1)+0.125M_{ccBbaa}(i-1)\Phi(1-\Phi)F_{CcBbAa}(i-1)+0.125M_{ccBbaa}(i-1)(1-\Phi)\Phi F_{CcBbAa}(i-1)+0.25M_{ccBbaa}(i-1)\Phi\Phi F_{CcBbAa}(i-1)+0.125M_{ccBbaa}(i-1)(1-\Phi)F_{CcBbAA}(i-1)+0.25M_{ccBbaa}(i-1)\Phi F_{CcBbAA}(i-1)+0.125M_{ccBbaa}(i-1)(1-\Phi)F_{CcBBAa}(i-1)+0.25M_{ccBbaa}(i-1)\Phi F_{CcBBAa}(i-1)+0.25M_{ccBbaa}(i-1)F_{CcBBAA}(i-1)+0.125M_{ccBbaa}(i-1)(1-\Phi)(1-\Phi)F_{CCBbAa}(i-1)+0.25M_{ccBbaa}(i-1)\Phi(1-\Phi)F_{CCBbAa}(i-1)+0.25M_{ccBbaa}(i-1)(1-\Phi)\Phi F_{CCBbAa}(i-1)+0.5M_{ccBbaa}(i-1)\Phi\Phi F_{CCBbAa}(i-1)+0.25M_{ccBbaa}(i-1)(1-\Phi)F_{CCBbAA}(i-1)+0.5M_{ccBbaa}(i-1)\Phi F_{CCBbAA}(i-1)+0.25M_{ccBbaa}(i-1)(1-\Phi)F_{CCBBAa}(i-1)+0.5M_{ccBbaa}(i-1)\Phi F_{CCBBAa}(i-1)+0.5M_{ccBbaa}(i-1)F_{CCBBAA}(i-1)+0.0625(1-\Phi)M_{ccBbAa}(i-1)(1-\Phi)F_{CcBbaa}(i-1)+0.125(1-\Phi)M_{ccBbAa}(i-1)\Phi F_{CcBbaa}(i-1)+0.0625(1-\Phi)M_{ccBbAa}(i-1)(1-\Phi)(1-\Phi)F_{CcBbAa}(i-1)+0.125(1-\Phi)M_{ccBbAa}(i-1)\Phi(1-\Phi)F_{CcBbAa}(i-1)+0.0625(1-\Phi)M_{ccBbAa}(i-1)(1-\Phi)\Phi F_{CcBbAa}(i-1)+0.125(1-\Phi)M_{ccBbAa}(i-1)\Phi\Phi F_{CcBbAa}(i-1)+0.0625(1-\Phi)M_{ccBbAa}(i-1)(1-\Phi)F_{CcBbAA}(i-1)+0.125(1-\Phi)M_{ccBbAa}(i-1)\Phi F_{CcBbAA}(i-1)+0.125(1-\Phi)M_{ccBbAa}(i-1)F_{CcBBaa}(i-1)+0.125(1-\Phi)M_{ccBbAa}(i-1)(1-\Phi)F_{CcBBAa}(i-1)+0.125(1-\Phi)M_{ccBbAa}(i-1)\Phi F_{CcBBAa}(i-1)+0.125(1-\Phi)M_{ccBbAa}(i-1)F_{CcBBAA}(i-1)+0.125(1-\Phi)M_{ccBbAa}(i-1)(1-\Phi)F_{CCBbaa}(i-1)+0.25(1-\Phi)M_{ccBbAa}(i-1)\Phi F_{CCBbaa}(i-1)+0.125(1-\Phi)M_{ccBbAa}(i-1)(1-\Phi)(1-\Phi)F_{CCBbAa}(i-1)+0.25(1-\Phi)M_{ccBbAa}(i-1)\Phi(1-\Phi)F_{CCBbAa}(i-1)+0.25(1-\Phi)M_{ccBbAa}(i-1)(1-\Phi)\Phi F_{CCBbAa}(i-1)+0.25(1-\Phi)M_{ccBbAa}(i-1)\Phi\Phi F_{CCBbAa}(i-1)+0.125(1-\Phi)M_{ccBbAa}(i-1)(1-\Phi)F_{CCBbAA}(i-1)+0.25(1-\Phi)M_{ccBbAa}(i-1)\Phi F_{CCBbAA}(i-1)+0.25(1-\Phi)M_{ccBbAa}(i-1)F_{CCBBaa}(i-1)+0.25(1-\Phi)M_{ccBbAa}(i-1)(1-\Phi)F_{CCBBAa}(i-1)+0.25(1-\Phi)M_{ccBbAa}(i-1)\Phi F_{CCBBAa}(i-1)+0.25(1-\Phi)M_{ccBbAa}(i-1)F_{CCBBAA}(i-1)+0.125\Phi M_{ccBbAa}(i-1)(1-\Phi)F_{CcBbaa}(i-1)+0.25\Phi M_{ccBbAa}(i-1)\Phi F_{CcBbaa}(i-1)+0.0625\Phi M_{ccBbAa}(i-1)(1-\Phi)(1-\Phi)F_{CcBbAa}(i-1)+0.125\Phi M_{ccBbAa}(i-1)\Phi(1-\Phi)F_{CcBbAa}(i-1)+0.25\Phi M_{ccBbAa}(i-1)F_{CcBBaa}(i-1)+0.125\Phi M_{ccBbAa}(i-1)(1-\Phi)F_{CcBBAa}(i-1)+0.25\Phi M_{ccBbAa}(i-1)(1-\Phi)F_{CCBbaa}(i-1)+0.5\Phi M_{ccBbAa}(i-1)\Phi F_{CCBbaa}(i-1)+0.125\Phi M_{ccBbAa}(i-1)(1-\Phi)(1-\Phi)F_{CCBbAa}(i-1)+0.25\Phi M_{ccBbAa}(i-1)\Phi(1-\Phi)F_{CCBbAa}(i-1)+0.25\Phi M_{ccBbAa}(i-1)(1-\Phi)\Phi F_{CCBbAa}(i-1)+0.5\Phi M_{ccBbAa}(i-1)F_{CCBBaa}(i-1)+0.25\Phi M_{ccBbAa}(i-1)(1-\Phi)F_{CCBBAa}(i-1)+0.125M_{ccBbAA}(i-1)(1-\Phi)F_{CcBbaa}(i-1)+0.25M_{ccBbAA}(i-1)\Phi F_{CcBbaa}(i-1)+0.0625M_{ccBbAA}(i-1)(1-\Phi)(1-\Phi)F_{CcBbAa}(i-1)+0.125M_{ccBbAA}(i-1)\Phi(1-\Phi)F_{CcBbAa}(i-1)+0.25M_{ccBbAA}(i-1)F_{CcBBaa}(i-1)+0.125M_{ccBbAA}(i-1)(1-\Phi)F_{CcBBAa}(i-1)+0.25M_{ccBbAA}(i-1)(1-\Phi)F_{CCBbaa}(i-1)+0.5M_{ccBbAA}(i-1)\Phi F_{CCBbaa}(i-1)+0.125M_{ccBbAA}(i-1)(1-\Phi)(1-\Phi)F_{CCBbAa}(i-1)+0.25M_{ccBbAA}(i-1)\Phi(1-\Phi)F_{CCBbAa}(i-1)+0.25M_{ccBbAA}(i-1)(1-\Phi)\Phi F_{CCBbAa}(i-1)+0.5M_{ccBbAA}(i-1)F_{CCBBaa}(i-1)+0.25M_{ccBbAA}(i-1)(1-\Phi)F_{CCBBAa}(i-1)+0.125M_{ccBBaa}(i-1)(1-\Phi)(1-\Phi)F_{CcBbAa}(i-1)+0.25M_{ccBBaa}(i-1)\Phi(1-\Phi)F_{CcBbAa}(i-1)+0.25M_{ccBBaa}(i-1)(1-\Phi)\Phi F_{CcBbAa}(i-1)+0.5M_{ccBBaa}(i-1)\Phi\Phi F_{CcBbAa}(i-1)+0.25M_{ccBBaa}(i-1)(1-\Phi)F_{CcBbAA}(i-1)+0.5M_{ccBBaa}(i-1)\Phi F_{CcBbAA}(i-1)+0.25M_{ccBBaa}(i-1)(1-\Phi)F_{CcBBAa}(i-1)+0.5M_{ccBBaa}(i-1)\Phi F_{CcBBAa}(i-1)+0.5M_{ccBBaa}(i-1)F_{CcBBAA}(i-1)+0.25M_{ccBBaa}(i-1)(1-\Phi)(1-\Phi)F_{CCBbAa}(i-1)+0.5M_{ccBBaa}(i-1)\Phi(1-\Phi)F_{CCBbAa}(i-1)+0.5M_{ccBBaa}(i-1)(1-\Phi)\Phi F_{CCBbAa}(i-1)+M_{ccBBaa}(i-1)\Phi\Phi F_{CCBbAa}(i-1)+0.5M_{ccBBaa}(i-1)(1-\Phi)F_{CCBbAA}(i-1)+M_{ccBBaa}(i-1)\Phi F_{CCBbAA}(i-1)+0.5M_{ccBBaa}(i-1)(1-\Phi)F_{CCBBAa}(i-1)+M_{ccBBaa}(i-1)\Phi F_{CCBBAa}(i-1)+M_{ccBBaa}(i-1)F_{CCBBAA}(i-1)+0.125(1-\Phi)M_{ccBBAa}(i-1)(1-\Phi)F_{CcBbaa}(i-1)+0.25(1-\Phi)M_{ccBBAa}(i-1)\Phi F_{CcBbaa}(i-1)+0.125(1-\Phi)M_{ccBBAa}(i-1)(1-\Phi)(1-\Phi)F_{CcBbAa}(i-1)+0.25(1-\Phi)M_{ccBBAa}(i-1)\Phi(1-\Phi)F_{CcBbAa}(i-1)+0.125(1-\Phi)M_{ccBBAa}(i-1)(1-\Phi)\Phi F_{CcBbAa}(i-1)+0.25(1-\Phi)M_{ccBBAa}(i-1)\Phi\Phi F_{CcBbAa}(i-1)+0.125(1-\Phi)M_{ccBBAa}(i-1)(1-\Phi)F_{CcBbAA}(i-1)+0.25(1-\Phi)M_{ccBBAa}(i-1)\Phi F_{CcBbAA}(i-1)+0.25(1-\Phi)M_{ccBBAa}(i-1)F_{CcBBaa}(i-1)+0.25(1-\Phi)M_{ccBBAa}(i-1)(1-\Phi)F_{CcBBAa}(i-1)+0.25(1-\Phi)M_{ccBBAa}(i-1)\Phi F_{CcBBAa}(i-1)+0.25(1-\Phi)M_{ccBBAa}(i-1)F_{CcBBAA}(i-1)+0.25(1-\Phi)M_{ccBBAa}(i-1)(1-\Phi)F_{CCBbaa}(i-1)+0.5(1-\Phi)M_{ccBBAa}(i-1)\Phi F_{CCBbaa}(i-1)+0.25(1-\Phi)M_{ccBBAa}(i-1)(1-\Phi)(1-\Phi)F_{CCBbAa}(i-1)+0.5(1-\Phi)M_{ccBBAa}(i-1)\Phi(1-\Phi)F_{CCBbAa}(i-1)+0.5(1-\Phi)M_{ccBBAa}(i-1)(1-\Phi)\Phi F_{CCBbAa}(i-1)+0.5(1-\Phi)M_{ccBBAa}(i-1)\Phi\Phi F_{CCBbAa}(i-1)+0.25(1-\Phi)M_{ccBBAa}(i-1)(1-\Phi)F_{CCBbAA}(i-1)+0.5(1-\Phi)M_{ccBBAa}(i-1)\Phi F_{CCBbAA}(i-1)+0.5(1-\Phi)M_{ccBBAa}(i-1)F_{CCBBaa}(i-1)+0.5(1-\Phi)M_{ccBBAa}(i-1)(1-\Phi)F_{CCBBAa}(i-1)+0.5(1-\Phi)M_{ccBBAa}(i-1)\Phi F_{CCBBAa}(i-1)+0.5(1-\Phi)M_{ccBBAa}(i-1)F_{CCBBAA}(i-1)+0.25\Phi M_{ccBBAa}(i-1)(1-\Phi)F_{CcBbaa}(i-1)+0.5\Phi M_{ccBBAa}(i-1)\Phi F_{CcBbaa}(i-1)+0.125\Phi M_{ccBBAa}(i-1)(1-\Phi)(1-\Phi)F_{CcBbAa}(i-1)+0.25\Phi M_{ccBBAa}(i-1)\Phi(1-\Phi)F_{CcBbAa}(i-1)+0.5\Phi M_{ccBBAa}(i-1)F_{CcBBaa}(i-1)+0.25\Phi M_{ccBBAa}(i-1)(1-\Phi)F_{CcBBAa}(i-1)+0.5\Phi M_{ccBBAa}(i-1)(1-\Phi)F_{CCBbaa}(i-1)+\Phi M_{ccBBAa}(i-1)\Phi F_{CCBbaa}(i-1)+0.25\Phi M_{ccBBAa}(i-1)(1-\Phi)(1-\Phi)F_{CCBbAa}(i-1)+0.5\Phi M_{ccBBAa}(i-1)\Phi(1-\Phi)F_{CCBbAa}(i-1)+0.5\Phi M_{ccBBAa}(i-1)(1-\Phi)\Phi F_{CCBbAa}(i-1)+\Phi M_{ccBBAa}(i-1)F_{CCBBaa}(i-1)+0.5\Phi M_{ccBBAa}(i-1)(1-\Phi)F_{CCBBAa}(i-1)+$$

$0.25M_{ccBBAA}(i-1)(1-\Phi)F_{CcBbaa}(i-1)+0.5M_{ccBBAA}(i-1)\Phi F_{CcBbaa}(i-1)+0.125M_{ccBBAA}(i-1)(1-$
$\Phi)(1-\Phi)F_{CcBbAa}(i-1)+0.25M_{ccBBAA}(i-1)\Phi(1-\Phi)F_{CcBbAa}(i-1)+0.5M_{ccBBAA}(i-1)F_{CcBBaa}(i-$
$1)+0.25M_{ccBBAA}(i-1)(1-\Phi)F_{CcBBAa}(i-1)+0.5M_{ccBBAA}(i-1)(1-\Phi)F_{CCBbaa}(i-1)+M_{ccBBAA}(i-$
$1)\Phi F_{CCBbaa}(i-1)+0.25M_{ccBBAA}(i-1)(1-\Phi)(1-\Phi)F_{CCBbAa}(i-1)+0.5M_{ccBBAA}(i-1)\Phi(1-$
$\Phi)F_{CCBbAa}(i-1)+0.5M_{ccBBAA}(i-1)(1-\Phi)\Phi F_{CCBbAa}(i-1)+M_{ccBBAA}(i-1)F_{CCBBaa}(i-1)+$
$0.5M_{ccBBAA}(i-1)(1-\Phi)F_{CCBBAa}(i-1)+0.0625(1-\Phi)M_{CcBbaa}(i-1)(1-\Phi)F_{ccBbAa}(i-1)+0.125(1-$
$\Phi)M_{CcBbaa}(i-1)\Phi F_{ccBbAa}(i-1)+0.125(1-\Phi)M_{CcBbaa}(i-1)F_{ccBbAA}(i-1)+0.125(1-\Phi)M_{CcBbaa}(i-1)(1-$
$\Phi)F_{ccBBAa}(i-1)+0.25(1-\Phi)M_{CcBbaa}(i-1)\Phi F_{ccBBAa}(i-1)+0.25(1-\Phi)M_{CcBbaa}(i-1)F_{ccBBAA}(i-1)+$
$0.0625(1-\Phi)M_{CcBbaa}(i-1)(1-\Phi)(1-\Phi)F_{CcBbAa}(i-1)+0.125(1-\Phi)M_{CcBbaa}(i-1)\Phi(1-\Phi)F_{CcBbAa}(i-$
$1)+0.125(1-\Phi)M_{CcBbaa}(i-1)(1-\Phi)\Phi F_{CcBbAa}(i-1)+0.25(1-\Phi)M_{CcBbaa}(i-1)\Phi\Phi F_{CcBbAa}(i-$
$1)+0.125(1-\Phi)M_{CcBbaa}(i-1)(1-\Phi)F_{CcBbAA}(i-1)+0.25(1-\Phi)M_{CcBbaa}(i-1)\Phi F_{CcBbAA}(i-1)+$
$0.125(1-\Phi)M_{CcBbaa}(i-1)(1-\Phi)F_{CcBBAa}(i-1)+0.25(1-\Phi)M_{CcBbaa}(i-1)\Phi F_{CcBBAa}(i-1)+0.25(1-$
$\Phi)M_{CcBbaa}(i-1)F_{CcBBAA}(i-1)+0.0625(1-\Phi)M_{CcBbaa}(i-1)(1-\Phi)(1-\Phi)F_{CCBbAa}(i-1)+0.125(1-$
$\Phi)M_{CcBbaa}(i-1)\Phi(1-\Phi)F_{CCBbAa}(i-1)+0.125(1-\Phi)M_{CcBbaa}(i-1)(1-\Phi)\Phi F_{CCBbAa}(i-1)+0.25(1-$
$\Phi)M_{CcBbaa}(i-1)\Phi\Phi F_{CCBbAa}(i-1)+0.125(1-\Phi)M_{CcBbaa}(i-1)(1-\Phi)F_{CCBbAA}(i-1)+0.25(1-$
$\Phi)M_{CcBbaa}(i-1)\Phi F_{CCBbAA}(i-1)+0.125(1-\Phi)M_{CcBbaa}(i-1)(1-\Phi)F_{CCBBAa}(i-1)+0.25(1-$
$\Phi)M_{CcBbaa}(i-1)\Phi F_{CCBBAa}(i-1)+0.25(1-\Phi)M_{CcBbaa}(i-1)F_{CCBBAA}(i-1)+0.125\Phi M_{CcBbaa}(i-$
$1)(1-\Phi)F_{ccBbAa}(i-1)+0.25\Phi M_{CcBbaa}(i-1)\Phi F_{ccBbAa}(i-1)+0.25\Phi M_{CcBbaa}(i-1)F_{ccBbAA}(i-1)+$
$0.25\Phi M_{CcBbaa}(i-1)(1-\Phi)F_{ccBBAa}(i-1)+0.5\Phi M_{CcBbaa}(i-1)\Phi F_{ccBBAa}(i-1)+0.5\Phi M_{CcBbaa}(i-$
$1)F_{ccBBAA}(i-1)+0.125\Phi M_{CcBbaa}(i-1)(1-\Phi)(1-\Phi)F_{CcBbAa}(i-1)+0.25\Phi M_{CcBbaa}(i-1)\Phi(1-$
$\Phi)F_{CcBbAa}(i-1)+0.25\Phi M_{CcBbaa}(i-1)(1-\Phi)\Phi F_{CcBbAa}(i-1)+0.5\Phi M_{CcBbaa}(i-1)\Phi\Phi F_{CcBbAa}(i-1)+$
$0.25\Phi M_{CcBbaa}(i-1)(1-\Phi)F_{CcBbAA}(i-1)+0.5\Phi M_{CcBbaa}(i-1)\Phi F_{CcBbAA}(i-1)+0.25\Phi M_{CcBbaa}(i-$
$1)(1-\Phi)F_{CcBBAa}(i-1)+0.5\Phi M_{CcBbaa}(i-1)\Phi F_{CcBBAa}(i-1)+0.5\Phi M_{CcBbaa}(i-1)F_{CcBBAA}(i-1)+$
$0.125\Phi M_{CcBbaa}(i-1)(1-\Phi)(1-\Phi)F_{CCBbAa}(i-1)+0.25\Phi M_{CcBbaa}(i-1)\Phi(1-\Phi)F_{CCBbAa}(i-1)+$
$0.25\Phi M_{CcBbaa}(i-1)(1-\Phi)\Phi F_{CCBbAa}(i-1)+0.5\Phi M_{CcBbaa}(i-1)\Phi\Phi F_{CCBbAa}(i-1)+0.25\Phi M_{CcBbaa}(i-$
$1)(1-\Phi)F_{CCBbAA}(i-1)+0.5\Phi M_{CcBbaa}(i-1)\Phi F_{CCBbAA}(i-1)+0.25\Phi M_{CcBbaa}(i-1)(1-\Phi)F_{CCBBAa}(i-$
$1)+0.5\Phi M_{CcBbaa}(i-1)\Phi F_{CCBBAa}(i-1)+0.5\Phi M_{CcBbaa}(i-1)F_{CCBBAA}(i-1)+0.0625(1-\Phi)(1-$
$\Phi)M_{CcBbAa}(i-1)F_{ccBbaa}(i-1)+0.0625(1-\Phi)(1-\Phi)M_{CcBbAa}(i-1)(1-\Phi)F_{ccBbAa}(i-1)+0.0625(1-$
$\Phi)(1-\Phi)M_{CcBbAa}(i-1)\Phi F_{ccBbAa}(i-1)+0.0625(1-\Phi)(1-\Phi)M_{CcBbAa}(i-1)F_{ccBbAA}(i-1)+0.125(1-$
$\Phi)(1-\Phi)M_{CcBbAa}(i-1)F_{ccBBaa}(i-1)+0.125(1-\Phi)(1-\Phi)M_{CcBbAa}(i-1)(1-\Phi)F_{ccBBAa}(i-1)+$
$0.125(1-\Phi)(1-\Phi)M_{CcBbAa}(i-1)\Phi F_{ccBBAa}(i-1)+0.125(1-\Phi)(1-\Phi)M_{CcBbAa}(i-1)F_{ccBBAA}(i-1)+$
$0.0625(1-\Phi)(1-\Phi)M_{CcBbAa}(i-1)(1-\Phi)F_{CcBbaa}(i-1)+0.125(1-\Phi)(1-\Phi)M_{CcBbAa}(i-1)\Phi F_{CcBbaa}(i-$
$1)+0.0625(1-\Phi)(1-\Phi)M_{CcBbAa}(i-1)(1-\Phi)(1-\Phi)F_{CcBbAa}(i-1)+0.125(1-\Phi)(1-\Phi)M_{CcBbAa}(i-$
$1)\Phi(1-\Phi)F_{CcBbAa}(i-1)+0.0625(1-\Phi)(1-\Phi)M_{CcBbAa}(i-1)(1-\Phi)\Phi F_{CcBbAa}(i-1)+0.125(1-\Phi)(1-$
$\Phi)M_{CcBbAa}(i-1)\Phi\Phi F_{CcBbAa}(i-1)+0.0625(1-\Phi)(1-\Phi)M_{CcBbAa}(i-1)(1-\Phi)F_{CcBbAA}(i-1)+0.125(1-$
$\Phi)(1-\Phi)M_{CcBbAa}(i-1)\Phi F_{CcBbAA}(i-1)+0.125(1-\Phi)(1-\Phi)M_{CcBbAa}(i-1)F_{CcBBaa}(i-1)+0.125(1-$
$\Phi)(1-\Phi)M_{CcBbAa}(i-1)(1-\Phi)F_{CcBBAa}(i-1)+0.125(1-\Phi)(1-\Phi)M_{CcBbAa}(i-1)\Phi F_{CcBBAa}(i-1)+$
$0.125(1-\Phi)(1-\Phi)M_{CcBbAa}(i-1)F_{CcBBAA}(i-1)+0.0625(1-\Phi)(1-\Phi)M_{CcBbAa}(i-1)(1-\Phi)F_{CCBbaa}(i-$
$1)+0.125(1-\Phi)(1-\Phi)M_{CcBbAa}(i-1)\Phi F_{CCBbaa}(i-1)+0.0625(1-\Phi)(1-\Phi)M_{CcBbAa}(i-1)(1-$
$\Phi)(1-\Phi)F_{CCBbAa}(i-1)+0.125(1-\Phi)(1-\Phi)M_{CcBbAa}(i-1)\Phi(1-\Phi)F_{CCBbAa}(i-1)+0.125(1-\Phi)(1-$
$\Phi)M_{CcBbAa}(i-1)(1-\Phi)\Phi F_{CCBbAa}(i-1)+0.125(1-\Phi)(1-\Phi)M_{CcBbAa}(i-1)\Phi\Phi F_{CCBbAa}(i-1)+0.0625(1-$
$\Phi)(1-\Phi)M_{CcBbAa}(i-1)(1-\Phi)F_{CCBbAA}(i-1)+0.125(1-\Phi)(1-\Phi)M_{CcBbAa}(i-1)\Phi F_{CCBbAA}(i-1)+$
$0.125(1-\Phi)(1-\Phi)M_{CcBbAa}(i-1)F_{CCBBaa}(i-1)+0.125(1-\Phi)(1-\Phi)M_{CcBbAa}(i-1)(1-\Phi)F_{CCBBAa}(i-$
$1)+0.125(1-\Phi)(1-\Phi)M_{CcBbAa}(i-1)\Phi F_{CCBBAa}(i-1)+0.125(1-\Phi)(1-\Phi)M_{CcBbAa}(i-1)F_{CCBBAA}(i-$
$1)+0.125\Phi(1-\Phi)M_{CcBbAa}(i-1)F_{ccBbaa}(i-1)+0.125\Phi(1-\Phi)M_{CcBbAa}(i-1)(1-\Phi)F_{ccBbAa}(i-$
$1)+0.125\Phi(1-\Phi)M_{CcBbAa}(i-1)\Phi F_{ccBbAa}(i-1)+0.125\Phi(1-\Phi)M_{CcBbAa}(i-1)F_{ccBbAA}(i-1)+$
$0.25\Phi(1-\Phi)M_{CcBbAa}(i-1)F_{ccBBaa}(i-1)+0.25\Phi(1-\Phi)M_{CcBbAa}(i-1)(1-\Phi)F_{ccBBAa}(i-1)+0.25\Phi(1-$
$\Phi)M_{CcBbAa}(i-1)\Phi F_{ccBBAa}(i-1)+0.25\Phi(1-\Phi)M_{CcBbAa}(i-1)F_{ccBBAA}(i-1)+0.125\Phi(1-\Phi)M_{CcBbAa}(i-$
$1)(1-\Phi)F_{CcBbaa}(i-1)+0.25\Phi(1-\Phi)M_{CcBbAa}(i-1)\Phi F_{CcBbaa}(i-1)+0.125\Phi(1-\Phi)M_{CcBbAa}(i-1)(1-$
$\Phi)(1-\Phi)F_{CcBbAa}(i-1)+0.25\Phi(1-\Phi)M_{CcBbAa}(i-1)\Phi(1-\Phi)F_{CcBbAa}(i-1)+0.125\Phi(1-\Phi)M_{CcBbAa}(i-$
$1)(1-\Phi)\Phi F_{CcBbAa}(i-1)+0.25\Phi(1-\Phi)M_{CcBbAa}(i-1)\Phi\Phi F_{CcBbAa}(i-1)+0.125\Phi(1-\Phi)M_{CcBbAa}(i-$
$1)(1-\Phi)F_{CcBbAA}(i-1)+0.25\Phi(1-\Phi)M_{CcBbAa}(i-1)\Phi F_{CcBbAA}(i-1)+0.25\Phi(1-\Phi)M_{CcBbAa}(i-$
$1)F_{CcBBaa}(i-1)+0.25\Phi(1-\Phi)M_{CcBbAa}(i-1)(1-\Phi)F_{CcBBAa}(i-1)+0.25\Phi(1-\Phi)M_{CcBbAa}(i-$
$1)\Phi F_{CcBBAa}(i-1)+0.25\Phi(1-\Phi)M_{CcBbAa}(i-1)F_{CcBBAA}(i-1)+0.125\Phi(1-\Phi)M_{CcBbAa}(i-1)(1-$
$\Phi)F_{CCBbaa}(i-1)+0.25\Phi(1-\Phi)M_{CcBbAa}(i-1)\Phi F_{CCBbaa}(i-1)+0.125\Phi(1-\Phi)M_{CcBbAa}(i-1)(1-\Phi)(1-$
$\Phi)F_{CCBbAa}(i-1)+0.25\Phi(1-\Phi)M_{CcBbAa}(i-1)\Phi(1-\Phi)F_{CCBbAa}(i-1)+0.25\Phi(1-\Phi)M_{CcBbAa}(i-$
$1)(1-\Phi)\Phi F_{CCBbAa}(i-1)+0.25\Phi(1-\Phi)M_{CcBbAa}(i-1)\Phi\Phi F_{CCBbAa}(i-1)+0.125\Phi(1-\Phi)M_{CcBbAa}(i-$

$1)(1-\Phi)F_{CCBbAA}(i-1)+0.25\Phi(1-\Phi)M_{CcBbAa}(i-1)\Phi F_{CCBbAA}(i-1)+0.25\Phi(1-\Phi)M_{CcBbAa}(i-$
$1)F_{CCBBaa}(i-1)+0.25\Phi(1-\Phi)M_{CcBbAa}(i-1)(1-\Phi)F_{CCBBAa}(i-1)+0.25\Phi(1-\Phi)M_{CcBbAa}(i-$
$1)\Phi F_{CCBBAa}(i-1)+0.25\Phi(1-\Phi)M_{CcBbAa}(i-1)F_{CCBBAA}(i-1)+0.125(1-\Phi)\Phi M_{CcBbAa}(i-1)F_{ccBbaa}(i-$
$1)+0.0625(1-\Phi)\Phi M_{CcBbAa}(i-1)(1-\Phi)F_{ccBbAa}(i-1)+0.25(1-\Phi)\Phi M_{CcBbAa}(i-1)F_{ccBBaa}(i-1)+$
$0.125(1-\Phi)\Phi M_{CcBbAa}(i-1)(1-\Phi)F_{ccBBAa}(i-1)+0.125(1-\Phi)\Phi M_{CcBbAa}(i-1)(1-\Phi)F_{CcBbaa}(i-1)+$
$0.25(1-\Phi)\Phi M_{CcBbAa}(i-1)\Phi F_{CcBbaa}(i-1)+0.0625(1-\Phi)\Phi M_{CcBbAa}(i-1)(1-\Phi)(1-\Phi)F_{CcBbAa}(i-$
$1)+0.125(1-\Phi)\Phi M_{CcBbAa}(i-1)\Phi(1-\Phi)F_{CcBbAa}(i-1)+0.25(1-\Phi)\Phi M_{CcBbAa}(i-1)F_{CcBBaa}(i-1)+$
$0.125(1-\Phi)\Phi M_{CcBbAa}(i-1)(1-\Phi)F_{CcBBAa}(i-1)+0.125(1-\Phi)\Phi M_{CcBbAa}(i-1)(1-\Phi)F_{CCBbaa}(i-1)+$
$0.25(1-\Phi)\Phi M_{CcBbAa}(i-1)\Phi F_{CCBbaa}(i-1)+0.0625(1-\Phi)\Phi M_{CcBbAa}(i-1)(1-\Phi)(1-\Phi)F_{CCBbAa}(i-1)+$
$0.125(1-\Phi)\Phi M_{CcBbAa}(i-1)\Phi(1-\Phi)F_{CCBbAa}(i-1)+0.125(1-\Phi)\Phi M_{CcBbAa}(i-1)(1-\Phi)\Phi F_{CCBbAa}(i-$
$1)+0.25(1-\Phi)\Phi M_{CcBbAa}(i-1)F_{CCBBaa}(i-1)+0.125(1-\Phi)\Phi M_{CcBbAa}(i-1)(1-\Phi)F_{CCBBAa}(i-1)+$
$0.25\Phi\Phi M_{CcBbAa}(i-1)F_{ccBbaa}(i-1)+0.125\Phi\Phi M_{CcBbAa}(i-1)(1-\Phi)F_{ccBbAa}(i-1)+0.5\Phi\Phi M_{CcBbAa}(i-$
$1)F_{ccBBaa}(i-1)+0.25\Phi\Phi M_{CcBbAa}(i-1)(1-\Phi)F_{ccBBAa}(i-1)+0.25\Phi\Phi M_{CcBbAa}(i-1)(1-\Phi)F_{CcBbaa}(i-$
$1)+0.5\Phi\Phi M_{CcBbAa}(i-1)\Phi F_{CcBbaa}(i-1)+0.125\Phi\Phi M_{CcBbAa}(i-1)(1-\Phi)(1-\Phi)F_{CcBbAa}(i-1)+$
$0.25\Phi\Phi M_{CcBbAa}(i-1)\Phi(1-\Phi)F_{CcBbAa}(i-1)+0.5\Phi\Phi M_{CcBbAa}(i-1)F_{CcBBaa}(i-1)+0.25\Phi\Phi M_{CcBbAa}(i-$
$1)(1-\Phi)F_{CcBBAa}(i-1)+0.25\Phi\Phi M_{CcBbAa}(i-1)(1-\Phi)F_{CCBbaa}(i-1)+0.5\Phi\Phi M_{CcBbAa}(i-1)\Phi F_{CCBbaa}(i-$
$1)+0.125\Phi\Phi M_{CcBbAa}(i-1)(1-\Phi)(1-\Phi)F_{CCBbAa}(i-1)+0.25\Phi\Phi M_{CcBbAa}(i-1)\Phi(1-\Phi)F_{CCBbAa}(i-1)+$
$0.25\Phi\Phi M_{CcBbAa}(i-1)(1-\Phi)\Phi F_{CCBbAa}(i-1)+0.5\Phi\Phi M_{CcBbAa}(i-1)F_{CCBBaa}(i-1)+0.25\Phi\Phi M_{CcBbAa}(i-$
$1)(1-\Phi)F_{CCBBAa}(i-1)+0.125(1-\Phi)M_{CcBbAA}(i-1)F_{ccBbaa}(i-1)+0.0625(1-\Phi)M_{CcBbAA}(i-1)(1-$
$\Phi)F_{ccBbAa}(i-1)+0.25(1-\Phi)M_{CcBbAA}(i-1)F_{ccBBaa}(i-1)+0.125(1-\Phi)M_{CcBbAA}(i-1)(1-\Phi)F_{ccBBAa}(i-$
$1)+0.125(1-\Phi)M_{CcBbAA}(i-1)(1-\Phi)F_{CcBbaa}(i-1)+0.25(1-\Phi)M_{CcBbAA}(i-1)\Phi F_{CcBbaa}(i-1)+$
$0.0625(1-\Phi)M_{CcBbAA}(i-1)(1-\Phi)(1-\Phi)F_{CcBbAa}(i-1)+0.125(1-\Phi)M_{CcBbAA}(i-1)\Phi(1-\Phi)F_{CcBbAa}(i-$
$1)+0.25(1-\Phi)M_{CcBbAA}(i-1)F_{CcBBaa}(i-1)+0.125(1-\Phi)M_{CcBbAA}(i-1)(1-\Phi)F_{CcBBAa}(i-1)+$
$0.125(1-\Phi)M_{CcBbAA}(i-1)(1-\Phi)F_{CCBbaa}(i-1)+0.25(1-\Phi)M_{CcBbAA}(i-1)\Phi F_{CCBbaa}(i-1)+0.0625(1-$
$\Phi)M_{CcBbAA}(i-1)(1-\Phi)(1-\Phi)F_{CCBbAa}(i-1)+0.125(1-\Phi)M_{CcBbAA}(i-1)\Phi(1-\Phi)F_{CCBbAa}(i-1)+$
$0.125(1-\Phi)M_{CcBbAA}(i-1)(1-\Phi)\Phi F_{CCBbAa}(i-1)+0.25(1-\Phi)M_{CcBbAA}(i-1)F_{CCBBaa}(i-1)+0.125(1-$
$\Phi)M_{CcBbAA}(i-1)(1-\Phi)F_{CCBBAa}(i-1)+0.25\Phi M_{CcBbAA}(i-1)F_{ccBbaa}(i-1)+0.125\Phi M_{CcBbAA}(i-$
$1)(1-\Phi)F_{ccBbAa}(i-1)+0.5\Phi M_{CcBbAA}(i-1)F_{ccBBaa}(i-1)+0.25\Phi M_{CcBbAA}(i-1)(1-\Phi)F_{ccBBAa}(i-1)+$
$0.25\Phi M_{CcBbAA}(i-1)(1-\Phi)F_{CcBbaa}(i-1)+0.5\Phi M_{CcBbAA}(i-1)\Phi F_{CcBbaa}(i-1)+0.125\Phi M_{CcBbAA}(i-1)(1-$
$\Phi)(1-\Phi)F_{CcBbAa}(i-1)+0.25\Phi M_{CcBbAA}(i-1)\Phi(1-\Phi)F_{CcBbAa}(i-1)+0.5\Phi M_{CcBbAA}(i-1)F_{CcBBaa}(i-1)+$
$0.25\Phi M_{CcBbAA}(i-1)(1-\Phi)F_{CcBBAa}(i-1)+0.25\Phi M_{CcBbAA}(i-1)(1-\Phi)F_{CCBbaa}(i-1)+0.5\Phi M_{CcBbAA}(i-$
$1)\Phi F_{CCBbaa}(i-1)+0.125\Phi M_{CcBbAA}(i-1)(1-\Phi)(1-\Phi)F_{CCBbAa}(i-1)+0.25\Phi M_{CcBbAA}(i-1)\Phi(1-$
$\Phi)F_{CCBbAa}(i-1)+0.25\Phi M_{CcBbAA}(i-1)(1-\Phi)\Phi F_{CCBbAa}(i-1)+0.5\Phi M_{CcBbAA}(i-1)F_{CCBBaa}(i-1)+$
$0.25\Phi M_{CcBbAA}(i-1)(1-\Phi)F_{CCBBAa}(i-1)+0.125M_{CcBBaa}(i-1)(1-\Phi)F_{ccBbAa}(i-1)+0.25M_{CcBBaa}(i-$
$1)\Phi F_{ccBbAa}(i-1)+0.25M_{CcBBaa}(i-1)F_{ccBbAA}(i-1)+0.25M_{CcBBaa}(i-1)(1-\Phi)F_{ccBBAa}(i-1)+$
$0.5M_{CcBBaa}(i-1)\Phi F_{ccBBAa}(i-1)+0.5M_{CcBBaa}(i-1)F_{ccBBAA}(i-1)+0.125M_{CcBBaa}(i-1)(1-\Phi)(1-$
$\Phi)F_{CcBbAa}(i-1)+0.25M_{CcBBaa}(i-1)\Phi(1-\Phi)F_{CcBbAa}(i-1)+0.25M_{CcBBaa}(i-1)(1-\Phi)\Phi F_{CcBbAa}(i-$
$1)+0.5M_{CcBBaa}(i-1)\Phi\Phi F_{CcBbAa}(i-1)+0.25M_{CcBBaa}(i-1)(1-\Phi)F_{CcBbAA}(i-1)+0.5M_{CcBBaa}(i-$
$1)\Phi F_{CcBbAA}(i-1)+0.25M_{CcBBaa}(i-1)(1-\Phi)F_{CcBBAa}(i-1)+0.5M_{CcBBaa}(i-1)\Phi F_{CcBBAa}(i-1)+$
$0.5M_{CcBBaa}(i-1)F_{CcBBAA}(i-1)+0.125M_{CcBBaa}(i-1)(1-\Phi)(1-\Phi)F_{CCBbAa}(i-1)+0.25M_{CcBBaa}(i-$
$1)\Phi(1-\Phi)F_{CCBbAa}(i-1)+0.25M_{CcBBaa}(i-1)(1-\Phi)\Phi F_{CCBbAa}(i-1)+0.5M_{CcBBaa}(i-1)\Phi\Phi F_{CCBbAa}(i-$
$1)+0.25M_{CcBBaa}(i-1)(1-\Phi)F_{CCBbAA}(i-1)+0.5M_{CcBBaa}(i-1)\Phi F_{CCBbAA}(i-1)+0.25M_{CcBBaa}(i-$
$1)(1-\Phi)F_{CCBBAa}(i-1)+0.5M_{CcBBaa}(i-1)\Phi F_{CCBBAa}(i-1)+0.5M_{CcBBaa}(i-1)F_{CCBBAA}(i-1)+$
$0.125(1-\Phi)M_{CcBBAa}(i-1)F_{ccBbaa}(i-1)+0.125(1-\Phi)M_{CcBBAa}(i-1)(1-\Phi)F_{ccBbAa}(i-1)+0.125(1-$
$\Phi)M_{CcBBAa}(i-1)\Phi F_{ccBbAa}(i-1)+0.125(1-\Phi)M_{CcBBAa}(i-1)F_{ccBbAA}(i-1)+0.25(1-\Phi)M_{CcBBAa}(i-$
$1)F_{ccBBaa}(i-1)+0.25(1-\Phi)M_{CcBBAa}(i-1)(1-\Phi)F_{ccBBAa}(i-1)+0.25(1-\Phi)M_{CcBBAa}(i-1)\Phi F_{ccBBAa}(i-$
$1)+0.25(1-\Phi)M_{CcBBAa}(i-1)F_{ccBBAA}(i-1)+0.125(1-\Phi)M_{CcBBAa}(i-1)(1-\Phi)F_{CcBbaa}(i-1)+$
$0.25(1-\Phi)M_{CcBBAa}(i-1)\Phi F_{CcBbaa}(i-1)+0.125(1-\Phi)M_{CcBBAa}(i-1)(1-\Phi)(1-\Phi)F_{CcBbAa}(i-1)+$
$0.25(1-\Phi)M_{CcBBAa}(i-1)\Phi(1-\Phi)F_{CcBbAa}(i-1)+0.125(1-\Phi)M_{CcBBAa}(i-1)(1-\Phi)\Phi F_{CcBbAa}(i-$
$1)+0.25(1-\Phi)M_{CcBBAa}(i-1)\Phi\Phi F_{CcBbAa}(i-1)+0.125(1-\Phi)M_{CcBBAa}(i-1)(1-\Phi)F_{CcBbAA}(i-$
$1)+0.25(1-\Phi)M_{CcBBAa}(i-1)\Phi F_{CcBbAA}(i-1)+0.25(1-\Phi)M_{CcBBAa}(i-1)F_{CcBBaa}(i-1)+0.25(1-$
$\Phi)M_{CcBBAa}(i-1)(1-\Phi)F_{CcBBAa}(i-1)+0.25(1-\Phi)M_{CcBBAa}(i-1)\Phi F_{CcBBAa}(i-1)+0.25(1-$
$\Phi)M_{CcBBAa}(i-1)F_{CcBBAA}(i-1)+0.125(1-\Phi)M_{CcBBAa}(i-1)(1-\Phi)F_{CCBbaa}(i-1)+0.25(1-$
$\Phi)M_{CcBBAa}(i-1)\Phi F_{CCBbaa}(i-1)+0.125(1-\Phi)M_{CcBBAa}(i-1)(1-\Phi)(1-\Phi)F_{CCBbAa}(i-1)+0.25(1-$
$\Phi)M_{CcBBAa}(i-1)\Phi(1-\Phi)F_{CCBbAa}(i-1)+0.25(1-\Phi)M_{CcBBAa}(i-1)(1-\Phi)\Phi F_{CCBbAa}(i-1)+0.25(1-$
$\Phi)M_{CcBBAa}(i-1)\Phi\Phi F_{CCBbAa}(i-1)+0.125(1-\Phi)M_{CcBBAa}(i-1)(1-\Phi)F_{CCBbAA}(i-1)+0.25(1-$
$\Phi)M_{CcBBAa}(i-1)\Phi F_{CCBbAA}(i-1)+0.25(1-\Phi)M_{CcBBAa}(i-1)F_{CCBBaa}(i-1)+0.25(1-\Phi)M_{CcBBAa}(i-$

$1)(1-\Phi)F_{CCBBAa}(i-1)+0.25(1-\Phi)M_{CcBBAa}(i-1)\Phi F_{CCBBAa}(i-1)+0.25(1-\Phi)M_{CcBBAa}(i-$
$1)F_{CCBBAA}(i-1)+0.25\Phi M_{CcBBAa}(i-1)F_{ccBbaa}(i-1)+0.125\Phi M_{CcBBAa}(i-1)(1-\Phi)F_{ccBbAa}(i-1)+$
$0.5\Phi M_{CcBBAa}(i-1)F_{ccBBaa}(i-1)+0.25\Phi M_{CcBBAa}(i-1)(1-\Phi)F_{ccBBAa}(i-1)+0.25\Phi M_{CcBBAa}(i-1)(1-$
$\Phi)F_{CcBbaa}(i-1)+0.5\Phi M_{CcBBAa}(i-1)\Phi F_{CcBbaa}(i-1)+0.125\Phi M_{CcBBAa}(i-1)(1-\Phi)(1-\Phi)F_{CcBbAa}(i-$
$1)+0.25\Phi M_{CcBBAa}(i-1)\Phi(1-\Phi)F_{CcBbAa}(i-1)+0.5\Phi M_{CcBBAa}(i-1)F_{CcBBaa}(i-1)+0.25\Phi M_{CcBBAa}(i-$
$1)(1-\Phi)F_{CcBBAa}(i-1)+0.25\Phi M_{CcBBAa}(i-1)(1-\Phi)F_{CCBbaa}(i-1)+0.5\Phi M_{CcBBAa}(i-1)\Phi F_{CCBbaa}(i-$
$1)+0.125\Phi M_{CcBBAa}(i-1)(1-\Phi)(1-\Phi)F_{CCBbAa}(i-1)+0.25\Phi M_{CcBBAa}(i-1)\Phi(1-\Phi)F_{CCBbAa}(i-1)+$
$0.25\Phi M_{CcBBAa}(i-1)(1-\Phi)\Phi F_{CCBbAa}(i-1)+0.5\Phi M_{CcBBAa}(i-1)F_{CCBBaa}(i-1)+0.25\Phi M_{CcBBAa}(i-$
$1)(1-\Phi)F_{CCBBAa}(i-1)+0.25M_{CcBBAA}(i-1)F_{ccBbaa}(i-1)+0.125M_{CcBBAA}(i-1)(1-\Phi)F_{ccBbAa}(i-1)+$
$0.5M_{CcBBAA}(i-1)F_{ccBBaa}(i-1)+0.25M_{CcBBAA}(i-1)(1-\Phi)F_{ccBBAa}(i-1)+0.25M_{CcBBAA}(i-1)(1-$
$\Phi)F_{CcBbaa}(i-1)+0.5M_{CcBBAA}(i-1)\Phi F_{CcBbaa}(i-1)+0.125M_{CcBBAA}(i-1)(1-\Phi)(1-\Phi)F_{CcBbAa}(i-$
$1)+0.25M_{CcBBAA}(i-1)\Phi(1-\Phi)F_{CcBbAa}(i-1)+0.5M_{CcBBAA}(i-1)F_{CcBBaa}(i-1)+0.25M_{CcBBAA}(i-$
$1)(1-\Phi)F_{CcBBAa}(i-1)+0.25M_{CcBBAA}(i-1)(1-\Phi)F_{CCBbaa}(i-1)+0.5M_{CcBBAA}(i-1)\Phi F_{CCBbaa}(i-$
$1)+0.125M_{CcBBAA}(i-1)(1-\Phi)(1-\Phi)F_{CCBbAa}(i-1)+0.25M_{CcBBAA}(i-1)\Phi(1-\Phi)F_{CCBbAa}(i-1)+$
$0.25M_{CcBBAA}(i-1)(1-\Phi)\Phi F_{CCBbAa}(i-1)+0.5M_{CcBBAA}(i-1)F_{CCBBaa}(i-1)+0.25M_{CcBBAA}(i-$
$1)(1-\Phi)F_{CCBBAa}(i-1)+0.125(1-\Phi)M_{CCBbaa}(i-1)(1-\Phi)F_{ccBbAa}(i-1)+0.25(1-\Phi)M_{CCBbaa}(i-$
$1)\Phi F_{ccBbAa}(i-1)+0.25(1-\Phi)M_{CCBbaa}(i-1)F_{ccBbAA}(i-1)+0.25(1-\Phi)M_{CCBbaa}(i-1)(1-\Phi)F_{ccBBAa}(i-$
$1)+0.5(1-\Phi)M_{CCBbaa}(i-1)\Phi F_{ccBBAa}(i-1)+0.5(1-\Phi)M_{CCBbaa}(i-1)F_{ccBBAA}(i-1)+0.0625(1-$
$\Phi)M_{CCBbaa}(i-1)(1-\Phi)(1-\Phi)F_{CcBbAa}(i-1)+0.125(1-\Phi)M_{CCBbaa}(i-1)\Phi(1-\Phi)F_{CcBbAa}(i-$
$1)+0.125(1-\Phi)M_{CCBbaa}(i-1)(1-\Phi)\Phi F_{CcBbAa}(i-1)+0.25(1-\Phi)M_{CCBbaa}(i-1)\Phi\Phi F_{CcBbAa}(i-$
$1)+0.125(1-\Phi)M_{CCBbaa}(i-1)(1-\Phi)F_{CcBbAA}(i-1)+0.25(1-\Phi)M_{CCBbaa}(i-1)\Phi F_{CcBbAA}(i-$
$1)+0.125(1-\Phi)M_{CCBbaa}(i-1)(1-\Phi)F_{CcBBAa}(i-1)+0.25(1-\Phi)M_{CCBbaa}(i-1)\Phi F_{CcBBAa}(i-1)+$
$0.25(1-\Phi)M_{CCBbaa}(i-1)F_{CcBBAA}(i-1)+0.25\Phi M_{CCBbaa}(i-1)(1-\Phi)F_{ccBbAa}(i-1)+0.5\Phi M_{CCBbaa}(i-$
$1)\Phi F_{ccBbAa}(i-1)+0.5\Phi M_{CCBbaa}(i-1)F_{ccBbAA}(i-1)+0.5\Phi M_{CCBbaa}(i-1)(1-\Phi)F_{ccBBAa}(i-1)+$
$\Phi M_{CCBbaa}(i-1)\Phi F_{ccBBAa}(i-1)+\Phi M_{CCBbaa}(i-1)F_{ccBBAA}(i-1)+0.125\Phi M_{CCBbaa}(i-1)(1-\Phi)(1-$
$\Phi)F_{CcBbAa}(i-1)+0.25\Phi M_{CCBbaa}(i-1)\Phi(1-\Phi)F_{CcBbAa}(i-1)+0.25\Phi M_{CCBbaa}(i-1)(1-\Phi)\Phi F_{CcBbAa}(i-$
$1)+0.5\Phi M_{CCBbaa}(i-1)\Phi\Phi F_{CcBbAa}(i-1)+0.25\Phi M_{CCBbaa}(i-1)(1-\Phi)F_{CcBbAA}(i-1)+0.5\Phi M_{CCBbaa}(i-$
$1)\Phi F_{CcBbAA}(i-1)+0.25\Phi M_{CCBbaa}(i-1)(1-\Phi)F_{CcBBAa}(i-1)+0.5\Phi M_{CCBbaa}(i-1)\Phi F_{CcBBAa}(i-1)+$
$0.5\Phi M_{CCBbaa}(i-1)F_{CcBBAA}(i-1)+0.125(1-\Phi)(1-\Phi)M_{CCBbAa}(i-1)F_{ccBbaa}(i-1)+0.125(1-\Phi)(1-$
$\Phi)M_{CCBbAa}(i-1)(1-\Phi)F_{ccBbAa}(i-1)+0.125(1-\Phi)(1-\Phi)M_{CCBbAa}(i-1)\Phi F_{ccBbAa}(i-1)+0.125(1-$
$\Phi)(1-\Phi)M_{CCBbAa}(i-1)F_{ccBbAA}(i-1)+0.25(1-\Phi)(1-\Phi)M_{CCBbAa}(i-1)F_{ccBBaa}(i-1)+0.25(1-\Phi)(1-$
$\Phi)M_{CCBbAa}(i-1)(1-\Phi)F_{ccBBAa}(i-1)+0.25(1-\Phi)(1-\Phi)M_{CCBbAa}(i-1)\Phi F_{ccBBAa}(i-1)+0.25(1-$
$\Phi)(1-\Phi)M_{CCBbAa}(i-1)F_{ccBBAA}(i-1)+0.0625(1-\Phi)(1-\Phi)M_{CCBbAa}(i-1)(1-\Phi)F_{CcBbaa}(i-$
$1)+0.125(1-\Phi)(1-\Phi)M_{CCBbAa}(i-1)\Phi F_{CcBbaa}(i-1)+0.0625(1-\Phi)(1-\Phi)M_{CCBbAa}(i-1)(1-$
$\Phi)(1-\Phi)F_{CcBbAa}(i-1)+0.125(1-\Phi)(1-\Phi)M_{CCBbAa}(i-1)\Phi(1-\Phi)F_{CcBbAa}(i-1)+0.0625(1-\Phi)(1-$
$\Phi)M_{CCBbAa}(i-1)(1-\Phi)\Phi F_{CcBbAa}(i-1)+0.125(1-\Phi)(1-\Phi)M_{CCBbAa}(i-1)\Phi\Phi F_{CcBbAa}(i-1)+0.0625(1-$
$\Phi)(1-\Phi)M_{CCBbAa}(i-1)(1-\Phi)F_{CcBbAA}(i-1)+0.125(1-\Phi)(1-\Phi)M_{CCBbAa}(i-1)\Phi F_{CcBbAA}(i-$
$1)+0.125(1-\Phi)(1-\Phi)M_{CCBbAa}(i-1)F_{CcBBaa}(i-1)+0.125(1-\Phi)(1-\Phi)M_{CCBbAa}(i-1)(1-$
$\Phi)F_{CcBBAa}(i-1)+0.125(1-\Phi)(1-\Phi)M_{CCBbAa}(i-1)\Phi F_{CcBBAa}(i-1)+0.125(1-\Phi)(1-\Phi)M_{CCBbAa}(i-$
$1)F_{CcBBAA}(i-1)+0.25\Phi(1-\Phi)M_{CCBbAa}(i-1)F_{ccBbaa}(i-1)+0.25\Phi(1-\Phi)M_{CCBbAa}(i-1)(1-$
$\Phi)F_{ccBbAa}(i-1)+0.25\Phi(1-\Phi)M_{CCBbAa}(i-1)\Phi F_{ccBbAa}(i-1)+0.25\Phi(1-\Phi)M_{CCBbAa}(i-1)F_{ccBbAA}(i-$
$1)+0.5\Phi(1-\Phi)M_{CCBbAa}(i-1)F_{ccBBaa}(i-1)+0.5\Phi(1-\Phi)M_{CCBbAa}(i-1)(1-\Phi)F_{ccBBAa}(i-1)+0.5\Phi(1-$
$\Phi)M_{CCBbAa}(i-1)\Phi F_{ccBBAa}(i-1)+0.5\Phi(1-\Phi)M_{CCBbAa}(i-1)F_{ccBBAA}(i-1)+0.125\Phi(1-\Phi)M_{CCBbAa}(i-$
$1)(1-\Phi)F_{CcBbaa}(i-1)+0.25\Phi(1-\Phi)M_{CCBbAa}(i-1)\Phi F_{CcBbaa}(i-1)+0.125\Phi(1-\Phi)M_{CCBbAa}(i-1)(1-$
$\Phi)(1-\Phi)F_{CcBbAa}(i-1)+0.25\Phi(1-\Phi)M_{CCBbAa}(i-1)\Phi(1-\Phi)F_{CcBbAa}(i-1)+0.125\Phi(1-\Phi)M_{CCBbAa}(i-$
$1)(1-\Phi)\Phi F_{CcBbAa}(i-1)+0.25\Phi(1-\Phi)M_{CCBbAa}(i-1)\Phi\Phi F_{CcBbAa}(i-1)+0.125\Phi(1-\Phi)M_{CCBbAa}(i-$
$1)(1-\Phi)F_{CcBbAA}(i-1)+0.25\Phi(1-\Phi)M_{CCBbAa}(i-1)\Phi F_{CcBbAA}(i-1)+0.25\Phi(1-\Phi)M_{CCBbAa}(i-$
$1)F_{CcBBaa}(i-1)+0.25\Phi(1-\Phi)M_{CCBbAa}(i-1)(1-\Phi)F_{CcBBAa}(i-1)+0.25\Phi(1-\Phi)M_{CCBbAa}(i-$
$1)\Phi F_{CcBBAa}(i-1)+0.25\Phi(1-\Phi)M_{CCBbAa}(i-1)F_{CcBBAA}(i-1)+0.25(1-\Phi)\Phi M_{CCBbAa}(i-1)F_{ccBbaa}(i-$
$1)+0.125(1-\Phi)\Phi M_{CCBbAa}(i-1)(1-\Phi)F_{ccBbAa}(i-1)+0.5(1-\Phi)\Phi M_{CCBbAa}(i-1)F_{ccBBaa}(i-1)+0.25(1-$
$\Phi)\Phi M_{CCBbAa}(i-1)(1-\Phi)F_{ccBBAa}(i-1)+0.125(1-\Phi)\Phi M_{CCBbAa}(i-1)(1-\Phi)F_{CcBbaa}(i-1)+0.25(1-$
$\Phi)\Phi M_{CCBbAa}(i-1)\Phi F_{CcBbaa}(i-1)+0.0625(1-\Phi)\Phi M_{CCBbAa}(i-1)(1-\Phi)(1-\Phi)F_{CcBbAa}(i-1)+0.125(1-$
$\Phi)\Phi M_{CCBbAa}(i-1)\Phi(1-\Phi)F_{CcBbAa}(i-1)+0.25(1-\Phi)\Phi M_{CCBbAa}(i-1)F_{CcBBaa}(i-1)+0.125(1-$
$\Phi)\Phi M_{CCBbAa}(i-1)(1-\Phi)F_{CcBBAa}(i-1)+0.5\Phi\Phi M_{CCBbAa}(i-1)F_{ccBbaa}(i-1)+0.25\Phi\Phi M_{CCBbAa}(i-$
$1)(1-\Phi)F_{ccBbAa}(i-1)+\Phi\Phi M_{CCBbAa}(i-1)F_{ccBBaa}(i-1)+0.5\Phi\Phi M_{CCBbAa}(i-1)(1-\Phi)F_{ccBBAa}(i-1)+$
$0.25\Phi\Phi M_{CCBbAa}(i-1)(1-\Phi)F_{CcBbaa}(i-1)+0.5\Phi\Phi M_{CCBbAa}(i-1)\Phi F_{CcBbaa}(i-1)+0.125\Phi\Phi M_{CCBbAa}(i-$
$1)(1-\Phi)(1-\Phi)F_{CcBbAa}(i-1)+0.25\Phi\Phi M_{CCBbAa}(i-1)\Phi(1-\Phi)F_{CcBbAa}(i-1)+0.5\Phi\Phi M_{CCBbAa}(i-$

$1)F_{CcBBaa}(i-1)+0.25\Phi\Phi M_{CCBbAa}(i-1)(1-\Phi)F_{CcBBAa}(i-1)+0.25(1-\Phi)M_{CCBbAA}(i-1)F_{ccBbaa}(i-$
$1)+0.125(1-\Phi)M_{CCBbAA}(i-1)(1-\Phi)F_{ccBbAa}(i-1)+0.5(1-\Phi)M_{CCBbAA}(i-1)F_{ccBBaa}(i-1)+$
$0.25(1-\Phi)M_{CCBbAA}(i-1)(1-\Phi)F_{ccBBAa}(i-1)+0.125(1-\Phi)M_{CCBbAA}(i-1)(1-\Phi)F_{CcBbaa}(i-1)+$
$0.25(1-\Phi)M_{CCBbAA}(i-1)\Phi F_{CcBbaa}(i-1)+0.0625(1-\Phi)M_{CCBbAA}(i-1)(1-\Phi)(1-\Phi)F_{CcBbAa}(i-$
$1)+0.125(1-\Phi)M_{CCBbAA}(i-1)\Phi(1-\Phi)F_{CcBbAa}(i-1)+0.25(1-\Phi)M_{CCBbAA}(i-1)F_{CcBBaa}(i-1)+$
$0.125(1-\Phi)M_{CCBbAA}(i-1)(1-\Phi)F_{CcBBAa}(i-1)+0.5\Phi M_{CCBbAA}(i-1)F_{ccBbaa}(i-1)+0.25\Phi M_{CCBbAA}(i-$
$1)(1-\Phi)F_{ccBbAa}(i-1)+\Phi M_{CCBbAA}(i-1)F_{ccBBaa}(i-1)+0.5\Phi M_{CCBbAA}(i-1)(1-\Phi)F_{ccBBAa}(i-1)+$
$0.25\Phi M_{CCBbAA}(i-1)(1-\Phi)F_{CcBbaa}(i-1)+0.5\Phi M_{CCBbAA}(i-1)\Phi F_{CcBbaa}(i-1)+0.125\Phi M_{CCBbAA}(i-$
$1)(1-\Phi)(1-\Phi)F_{CcBbAa}(i-1)+0.25\Phi M_{CCBbAA}(i-1)\Phi(1-\Phi)F_{CcBbAa}(i-1)+0.5\Phi M_{CCBbAA}(i-$
$1)F_{CcBBaa}(i-1)+0.25\Phi M_{CCBbAA}(i-1)(1-\Phi)F_{CcBBAa}(i-1)+0.25M_{CCBBaa}(i-1)(1-\Phi)F_{ccBbAa}(i-$
$1)+0.5M_{CCBBaa}(i-1)\Phi F_{ccBbAa}(i-1)+0.5M_{CCBBaa}(i-1)F_{ccBbAA}(i-1)+0.5M_{CCBBaa}(i-1)(1-$
$\Phi)F_{ccBBAa}(i-1)+M_{CCBBaa}(i-1)\Phi F_{ccBBAa}(i-1)+M_{CCBBaa}(i-1)F_{ccBBAA}(i-1)+0.125M_{CCBBaa}(i-$
$1)(1-\Phi)(1-\Phi)F_{CcBbAa}(i-1)+0.25M_{CCBBaa}(i-1)\Phi(1-\Phi)F_{CcBbAa}(i-1)+0.25M_{CCBBaa}(i-1)(1-$
$\Phi)\Phi F_{CcBbAa}(i-1)+0.5M_{CCBBaa}(i-1)\Phi\Phi F_{CcBbAa}(i-1)+0.25M_{CCBBaa}(i-1)(1-\Phi)F_{CcBbAA}(i-$
$1)+0.5M_{CCBBaa}(i-1)\Phi F_{CcBbAA}(i-1)+0.25M_{CCBBaa}(i-1)(1-\Phi)F_{CcBBAa}(i-1)+0.5M_{CCBBaa}(i-$
$1)\Phi F_{CcBBAa}(i-1)+0.5M_{CCBBaa}(i-1)F_{CcBBAA}(i-1)+0.25(1-\Phi)M_{CCBBAa}(i-1)F_{ccBbaa}(i-1)+$
$0.25(1-\Phi)M_{CCBBAa}(i-1)(1-\Phi)F_{ccBbAa}(i-1)+0.25(1-\Phi)M_{CCBBAa}(i-1)\Phi F_{ccBbAa}(i-1)+0.25(1-$
$\Phi)M_{CCBBAa}(i-1)F_{ccBbAA}(i-1)+0.5(1-\Phi)M_{CCBBAa}(i-1)F_{ccBBaa}(i-1)+0.5(1-\Phi)M_{CCBBAa}(i-1)(1-$
$\Phi)F_{ccBBAa}(i-1)+0.5(1-\Phi)M_{CCBBAa}(i-1)\Phi F_{ccBBAa}(i-1)+0.5(1-\Phi)M_{CCBBAa}(i-1)F_{ccBBAA}(i-$
$1)+0.125(1-\Phi)M_{CCBBAa}(i-1)(1-\Phi)F_{CcBbaa}(i-1)+0.25(1-\Phi)M_{CCBBAa}(i-1)\Phi F_{CcBbaa}(i-1)+$
$0.125(1-\Phi)M_{CCBBAa}(i-1)(1-\Phi)(1-\Phi)F_{CcBbAa}(i-1)+0.25(1-\Phi)M_{CCBBAa}(i-1)\Phi(1-\Phi)F_{CcBbAa}(i-$
$1)+0.125(1-\Phi)M_{CCBBAa}(i-1)(1-\Phi)\Phi F_{CcBbAa}(i-1)+0.25(1-\Phi)M_{CCBBAa}(i-1)\Phi\Phi F_{CcBbAa}(i-$
$1)+0.125(1-\Phi)M_{CCBBAa}(i-1)(1-\Phi)F_{CcBbAA}(i-1)+0.25(1-\Phi)M_{CCBBAa}(i-1)\Phi F_{CcBbAA}(i-1)+$
$0.25(1-\Phi)M_{CCBBAa}(i-1)F_{CcBBaa}(i-1)+0.25(1-\Phi)M_{CCBBAa}(i-1)(1-\Phi)F_{CcBBAa}(i-1)+0.25(1-$
$\Phi)M_{CCBBAa}(i-1)\Phi F_{CcBBAa}(i-1)+0.25(1-\Phi)M_{CCBBAa}(i-1)F_{CcBBAA}(i-1)+0.5\Phi M_{CCBBAa}(i-$
$1)F_{ccBbaa}(i-1)+0.25\Phi M_{CCBBAa}(i-1)(1-\Phi)F_{ccBbAa}(i-1)+\Phi M_{CCBBAa}(i-1)F_{ccBBaa}(i-1)+$
$0.5\Phi M_{CCBBAa}(i-1)(1-\Phi)F_{ccBBAa}(i-1)+0.25\Phi M_{CCBBAa}(i-1)(1-\Phi)F_{CcBbaa}(i-1)+0.5\Phi M_{CCBBAa}(i-$
$1)\Phi F_{CcBbaa}(i-1)+0.125\Phi M_{CCBBAa}(i-1)(1-\Phi)(1-\Phi)F_{CcBbAa}(i-1)+0.25\Phi M_{CCBBAa}(i-1)\Phi(1-$
$\Phi)F_{CcBbAa}(i-1)+0.5\Phi M_{CCBBAa}(i-1)F_{CcBBaa}(i-1)+0.25\Phi M_{CCBBAa}(i-1)(1-\Phi)F_{CcBBAa}(i-1)+$
$0.5M_{CCBBAA}(i-1)F_{ccBbaa}(i-1)+0.25M_{CCBBAA}(i-1)(1-\Phi)F_{ccBbAa}(i-1)+M_{CCBBAA}(i-1)F_{ccBBaa}(i-$
$1)+0.5M_{CCBBAA}(i-1)(1-\Phi)F_{ccBBAa}(i-1)+0.25M_{CCBBAA}(i-1)(1-\Phi)F_{CcBbaa}(i-1)+0.5M_{CCBBAA}(i-$
$1)\Phi F_{CcBbaa}(i-1)+0.125M_{CCBBAA}(i-1)(1-\Phi)(1-\Phi)F_{CcBbAa}(i-1)+0.25M_{CCBBAA}(i-1)\Phi(1-$
$\Phi)F_{CcBbAa}(i-1)+0.5M_{CCBBAA}(i-1)F_{CcBBaa}(i-1)+0.25M_{CCBBAA}(i-1)(1-\Phi)F_{CcBBAa}(i-1)],$

$\mathrm{M}^{e}_{CcBBAA}=\left(\frac{\bar{\Omega}_{CcBBAA}}{2}\right)[0.03125(1-\Phi)M_{ccBbAa}(i-1)(1-\Phi)(1-\Phi)F_{CcBbAa}(i-1)+0.0625(1-$
$\Phi)M_{ccBbAa}(i-1)\Phi(1-\Phi)F_{CcBbAa}(i-1)+0.0625(1-\Phi)M_{ccBbAa}(i-1)(1-\Phi)\Phi F_{CcBbAa}(i-1)+0.125(1-$
$\Phi)M_{ccBbAa}(i-1)\Phi\Phi F_{CcBbAa}(i-1)+0.0625(1-\Phi)M_{ccBbAa}(i-1)(1-\Phi)F_{CcBbAA}(i-1)+0.125(1-$
$\Phi)M_{ccBbAa}(i-1)\Phi F_{CcBbAA}(i-1)+0.0625(1-\Phi)M_{ccBbAa}(i-1)(1-\Phi)F_{CcBBAa}(i-1)+0.125(1-$
$\Phi)M_{ccBbAa}(i-1)\Phi F_{CcBBAa}(i-1)+0.125(1-\Phi)M_{ccBbAa}(i-1)F_{CcBBAA}(i-1)+0.0625(1-\Phi)M_{ccBbAa}(i-$
$1)(1-\Phi)(1-\Phi)F_{CCBbAa}(i-1)+0.125(1-\Phi)M_{ccBbAa}(i-1)\Phi(1-\Phi)F_{CCBbAa}(i-1)+0.125(1-\Phi)M_{ccBbAa}(i-$
$1)(1-\Phi)\Phi F_{CCBbAa}(i-1)+0.25(1-\Phi)M_{ccBbAa}(i-1)\Phi\Phi F_{CCBbAa}(i-1)+0.125(1-\Phi)M_{ccBbAa}(i-$
$1)(1-\Phi)F_{CCBbAA}(i-1)+0.25(1-\Phi)M_{ccBbAa}(i-1)\Phi F_{CCBbAA}(i-1)+0.125(1-\Phi)M_{ccBbAa}(i-$
$1)(1-\Phi)F_{CCBBAa}(i-1)+0.25(1-\Phi)M_{ccBbAa}(i-1)\Phi F_{CCBBAa}(i-1)+0.25(1-\Phi)M_{ccBbAa}(i-$
$1)F_{CCBBAA}(i-1)+0.0625\Phi M_{ccBbAa}(i-1)(1-\Phi)(1-\Phi)F_{CcBbAa}(i-1)+0.125\Phi M_{ccBbAa}(i-1)\Phi(1-$
$\Phi)F_{CcBbAa}(i-1)+0.125\Phi M_{ccBbAa}(i-1)(1-\Phi)\Phi F_{CcBbAa}(i-1)+0.25\Phi M_{ccBbAa}(i-1)\Phi\Phi F_{CcBbAa}(i-1)+$
$0.125\Phi M_{ccBbAa}(i-1)(1-\Phi)F_{CcBbAA}(i-1)+0.25\Phi M_{ccBbAa}(i-1)\Phi F_{CcBbAA}(i-1)+0.125\Phi M_{ccBbAa}(i-$
$1)(1-\Phi)F_{CcBBAa}(i-1)+0.25\Phi M_{ccBbAa}(i-1)\Phi F_{CcBBAa}(i-1)+0.25\Phi M_{ccBbAa}(i-1)F_{CcBBAA}(i-$
$1)+0.125\Phi M_{ccBbAa}(i-1)(1-\Phi)(1-\Phi)F_{CCBbAa}(i-1)+0.25\Phi M_{ccBbAa}(i-1)\Phi(1-\Phi)F_{CCBbAa}(i-1)+$
$0.25\Phi M_{ccBbAa}(i-1)(1-\Phi)\Phi F_{CCBbAa}(i-1)+0.5\Phi M_{ccBbAa}(i-1)\Phi\Phi F_{CCBbAa}(i-1)+0.25\Phi M_{ccBbAa}(i-$
$1)(1-\Phi)F_{CCBbAA}(i-1)+0.5\Phi M_{ccBbAa}(i-1)\Phi F_{CCBbAA}(i-1)+0.25\Phi M_{ccBbAa}(i-1)(1-\Phi)F_{CCBBAa}(i-$
$1)+0.5\Phi M_{ccBbAa}(i-1)\Phi F_{CCBBAa}(i-1)+0.5\Phi M_{ccBbAa}(i-1)F_{CCBBAA}(i-1)+0.0625M_{ccBbAA}(i-$
$1)(1-\Phi)(1-\Phi)F_{CcBbAa}(i-1)+0.125M_{ccBbAA}(i-1)\Phi(1-\Phi)F_{CcBbAa}(i-1)+0.125M_{ccBbAA}(i-1)(1-$
$\Phi)\Phi F_{CcBbAa}(i-1)+0.25M_{ccBbAA}(i-1)\Phi\Phi F_{CcBbAa}(i-1)+0.125M_{ccBbAA}(i-1)(1-\Phi)F_{CcBbAA}(i-$
$1)+0.25M_{ccBbAA}(i-1)\Phi F_{CcBbAA}(i-1)+0.125M_{ccBbAA}(i-1)(1-\Phi)F_{CcBBAa}(i-1)+0.25M_{ccBbAA}(i-$
$1)\Phi F_{CcBBAa}(i-1)+0.25M_{ccBbAA}(i-1)F_{CcBBAA}(i-1)+0.125M_{ccBbAA}(i-1)(1-\Phi)(1-\Phi)F_{CCBbAa}(i-1)+$
$0.25M_{ccBbAA}(i-1)\Phi(1-\Phi)F_{CCBbAa}(i-1)+0.25M_{ccBbAA}(i-1)(1-\Phi)\Phi F_{CCBbAa}(i-1)+0.5M_{ccBbAA}(i-$
$1)\Phi\Phi F_{CCBbAa}(i-1)+0.25M_{ccBbAA}(i-1)(1-\Phi)F_{CCBbAA}(i-1)+0.5M_{ccBbAA}(i-1)\Phi F_{CCBbAA}(i-$

$1) + 0.25M_{ccBbAA}(i-1)(1-\Phi)F_{CCBBAa}(i-1) + 0.5M_{ccBbAA}(i-1)\Phi F_{CCBBAa}(i-1) + 0.5M_{ccBbAA}(i-$
$1)F_{CCBBAA}(i-1) + 0.0625(1-\Phi)M_{ccBBAa}(i-1)(1-\Phi)(1-\Phi)F_{CcBbAa}(i-1) + 0.125(1-\Phi)M_{ccBBAa}(i-$
$1)\Phi(1-\Phi)F_{CcBbAa}(i-1) + 0.125(1-\Phi)M_{ccBBAa}(i-1)(1-\Phi)\Phi F_{CcBbAa}(i-1) + 0.25(1-\Phi)M_{ccBBAa}(i-$
$1)\Phi\Phi F_{CcBbAa}(i-1) + 0.125(1-\Phi)M_{ccBBAa}(i-1)(1-\Phi)F_{CcBbAA}(i-1) + 0.25(1-\Phi)M_{ccBBAa}(i-$
$1)\Phi F_{CcBbAA}(i-1) + 0.125(1-\Phi)M_{ccBBAa}(i-1)(1-\Phi)F_{CcBBAa}(i-1) + 0.25(1-\Phi)M_{ccBBAa}(i-$
$1)\Phi F_{CcBBAa}(i-1) + 0.25(1-\Phi)M_{ccBBAa}(i-1)F_{CcBBAA}(i-1) + 0.125(1-\Phi)M_{ccBBAa}(i-1)(1-$
$\Phi)(1-\Phi)F_{CCBbAa}(i-1) + 0.25(1-\Phi)M_{ccBBAa}(i-1)\Phi(1-\Phi)F_{CCBbAa}(i-1) + 0.25(1-\Phi)M_{ccBBAa}(i-$
$1)(1-\Phi)\Phi F_{CCBbAa}(i-1) + 0.5(1-\Phi)M_{ccBBAa}(i-1)\Phi\Phi F_{CCBbAa}(i-1) + 0.25(1-\Phi)M_{ccBBAa}(i-$
$1)(1-\Phi)F_{CCBbAA}(i-1) + 0.5(1-\Phi)M_{ccBBAa}(i-1)\Phi F_{CCBbAA}(i-1) + 0.25(1-\Phi)M_{ccBBAa}(i-1)(1-$
$\Phi)F_{CCBBAa}(i-1) + 0.5(1-\Phi)M_{ccBBAa}(i-1)\Phi F_{CCBBAa}(i-1) + 0.5(1-\Phi)M_{ccBBAa}(i-1)F_{CCBBAA}(i-$
$1) + 0.125\Phi M_{ccBBAa}(i-1)(1-\Phi)(1-\Phi)F_{CcBbAa}(i-1) + 0.25\Phi M_{ccBBAa}(i-1)\Phi(1-\Phi)F_{CcBbAa}(i-1) +$
$0.25\Phi M_{ccBBAa}(i-1)(1-\Phi)\Phi F_{CcBbAa}(i-1) + 0.5\Phi M_{ccBBAa}(i-1)\Phi\Phi F_{CcBbAa}(i-1) + 0.25\Phi M_{ccBBAa}(i-$
$1)(1-\Phi)F_{CcBbAA}(i-1) + 0.5\Phi M_{ccBBAa}(i-1)\Phi F_{CcBbAA}(i-1) + 0.25\Phi M_{ccBBAa}(i-1)(1-\Phi)F_{CcBBAa}(i-1) +$
$0.5\Phi M_{ccBBAa}(i-1)\Phi F_{CcBBAa}(i-1) + 0.5\Phi M_{ccBBAa}(i-1)F_{CcBBAA}(i-1) + 0.25\Phi M_{ccBBAa}(i-1)(1-\Phi)(1-$
$\Phi)F_{CCBbAa}(i-1) + 0.5\Phi M_{ccBBAa}(i-1)\Phi(1-\Phi)F_{CCBbAa}(i-1) + 0.5\Phi M_{ccBBAa}(i-1)(1-\Phi)\Phi F_{CCBbAa}(i-$
$1) + \Phi M_{ccBBAa}(i-1)\Phi\Phi F_{CCBbAa}(i-1) + 0.5\Phi M_{ccBBAa}(i-1)(1-\Phi)F_{CCBbAA}(i-1) + \Phi M_{ccBBAa}(i-$
$1)\Phi F_{CCBbAA}(i-1) + 0.5\Phi M_{ccBBAa}(i-1)(1-\Phi)F_{CCBBAa}(i-1) + \Phi M_{ccBBAa}(i-1)\Phi F_{CCBBAa}(i-1) +$
$\Phi M_{ccBBAa}(i-1)F_{CCBBAA}(i-1) + 0.125M_{ccBBAA}(i-1)(1-\Phi)(1-\Phi)F_{CcBbAa}(i-1) + 0.25M_{ccBBAA}(i-$
$1)\Phi(1-\Phi)F_{CcBbAa}(i-1) + 0.25M_{ccBBAA}(i-1)(1-\Phi)\Phi F_{CcBbAa}(i-1) + 0.5M_{ccBBAA}(i-1)\Phi\Phi F_{CcBbAa}(i-$
$1) + 0.25M_{ccBBAA}(i-1)(1-\Phi)F_{CcBbAA}(i-1) + 0.5M_{ccBBAA}(i-1)\Phi F_{CcBbAA}(i-1) + 0.25M_{ccBBAA}(i-$
$1)(1-\Phi)F_{CcBBAa}(i-1) + 0.5M_{ccBBAA}(i-1)\Phi F_{CcBBAa}(i-1) + 0.5M_{ccBBAA}(i-1)F_{CcBBAA}(i-1) +$
$0.25M_{ccBBAA}(i-1)(1-\Phi)(1-\Phi)F_{CCBbAa}(i-1) + 0.5M_{ccBBAA}(i-1)\Phi(1-\Phi)F_{CCBbAa}(i-1) + 0.5M_{ccBBAA}(i-$
$1)(1-\Phi)\Phi F_{CCBbAa}(i-1) + M_{ccBBAA}(i-1)\Phi\Phi F_{CCBbAa}(i-1) + 0.5M_{ccBBAA}(i-1)(1-\Phi)F_{CCBbAA}(i-1) +$
$M_{ccBBAA}(i-1)\Phi F_{CCBbAA}(i-1) + 0.5M_{ccBBAA}(i-1)(1-\Phi)F_{CCBBAa}(i-1) + M_{ccBBAA}(i-1)\Phi F_{CCBBAa}(i-$
$1) + M_{ccBBAA}(i-1)F_{CCBBAA}(i-1) + 0.03125(1-\Phi)(1-\Phi)M_{CcBbAa}(i-1)(1-\Phi)F_{ccBbAa}(i-1) + 0.0625(1-$
$\Phi)(1-\Phi)M_{CcBbAa}(i-1)\Phi F_{ccBbAa}(i-1) + 0.0625(1-\Phi)(1-\Phi)M_{CcBbAa}(i-1)F_{ccBbAA}(i-1) + 0.0625(1-$
$\Phi)(1-\Phi)M_{CcBbAa}(i-1)(1-\Phi)F_{ccBBAa}(i-1) + 0.125(1-\Phi)(1-\Phi)M_{CcBbAa}(i-1)\Phi F_{ccBBAa}(i-1) + 0.125(1-$
$\Phi)(1-\Phi)M_{CcBbAa}(i-1)F_{ccBBAA}(i-1) + 0.03125(1-\Phi)(1-\Phi)M_{CcBbAa}(i-1)(1-\Phi)(1-\Phi)F_{CcBbAa}(i-$
$1) + 0.0625(1-\Phi)(1-\Phi)M_{CcBbAa}(i-1)\Phi(1-\Phi)F_{CcBbAa}(i-1) + 0.0625(1-\Phi)(1-\Phi)M_{CcBbAa}(i-1)(1-$
$\Phi)\Phi F_{CcBbAa}(i-1) + 0.125(1-\Phi)(1-\Phi)M_{CcBbAa}(i-1)\Phi\Phi F_{CcBbAa}(i-1) + 0.0625(1-\Phi)(1-\Phi)M_{CcBbAa}(i-$
$1)(1-\Phi)F_{CcBbAA}(i-1) + 0.125(1-\Phi)(1-\Phi)M_{CcBbAa}(i-1)\Phi F_{CcBbAA}(i-1) + 0.0625(1-\Phi)(1-$
$\Phi)M_{CcBbAa}(i-1)(1-\Phi)F_{CcBBAa}(i-1) + 0.125(1-\Phi)(1-\Phi)M_{CcBbAa}(i-1)\Phi F_{CcBBAa}(i-1) + 0.125(1-$
$\Phi)(1-\Phi)M_{CcBbAa}(i-1)F_{CcBBAA}(i-1) + 0.03125(1-\Phi)(1-\Phi)M_{CcBbAa}(i-1)(1-\Phi)(1-\Phi)F_{CCBbAa}(i-$
$1) + 0.0625(1-\Phi)(1-\Phi)M_{CcBbAa}(i-1)\Phi(1-\Phi)F_{CCBbAa}(i-1) + 0.0625(1-\Phi)(1-\Phi)M_{CcBbAa}(i-1)(1-$
$\Phi)\Phi F_{CCBbAa}(i-1) + 0.125(1-\Phi)(1-\Phi)M_{CcBbAa}(i-1)\Phi\Phi F_{CCBbAa}(i-1) + 0.0625(1-\Phi)(1-\Phi)M_{CcBbAa}(i-$
$1)(1-\Phi)F_{CCBbAA}(i-1) + 0.125(1-\Phi)(1-\Phi)M_{CcBbAa}(i-1)\Phi F_{CCBbAA}(i-1) + 0.0625(1-\Phi)(1-$
$\Phi)M_{CcBbAa}(i-1)(1-\Phi)F_{CCBBAa}(i-1) + 0.125(1-\Phi)(1-\Phi)M_{CcBbAa}(i-1)\Phi F_{CCBBAa}(i-1) + 0.125(1-$
$\Phi)(1-\Phi)M_{CcBbAa}(i-1)F_{CCBBAA}(i-1) + 0.0625\Phi(1-\Phi)M_{CcBbAa}(i-1)(1-\Phi)F_{ccBbAa}(i-1) + 0.125\Phi(1-$
$\Phi)M_{CcBbAa}(i-1)\Phi F_{ccBbAa}(i-1) + 0.125\Phi(1-\Phi)M_{CcBbAa}(i-1)F_{ccBbAA}(i-1) + 0.125\Phi(1-\Phi)M_{CcBbAa}(i-$
$1)(1-\Phi)F_{ccBBAa}(i-1) + 0.25\Phi(1-\Phi)M_{CcBbAa}(i-1)\Phi F_{ccBBAa}(i-1) + 0.25\Phi(1-\Phi)M_{CcBbAa}(i-$
$1)F_{ccBBAA}(i-1) + 0.0625\Phi(1-\Phi)M_{CcBbAa}(i-1)(1-\Phi)(1-\Phi)F_{CcBbAa}(i-1) + 0.125\Phi(1-\Phi)M_{CcBbAa}(i-$
$1)\Phi(1-\Phi)F_{CcBbAa}(i-1) + 0.125\Phi(1-\Phi)M_{CcBbAa}(i-1)(1-\Phi)\Phi F_{CcBbAa}(i-1) + 0.25\Phi(1-\Phi)M_{CcBbAa}(i-$
$1)\Phi\Phi F_{CcBbAa}(i-1) + 0.125\Phi(1-\Phi)M_{CcBbAa}(i-1)(1-\Phi)F_{CcBbAA}(i-1) + 0.25\Phi(1-\Phi)M_{CcBbAa}(i-$
$1)\Phi F_{CcBbAA}(i-1) + 0.125\Phi(1-\Phi)M_{CcBbAa}(i-1)(1-\Phi)F_{CcBBAa}(i-1) + 0.25\Phi(1-\Phi)M_{CcBbAa}(i-$
$1)\Phi F_{CcBBAa}(i-1) + 0.25\Phi(1-\Phi)M_{CcBbAa}(i-1)F_{CcBBAA}(i-1) + 0.0625\Phi(1-\Phi)M_{CcBbAa}(i-1)(1-\Phi)(1-$
$\Phi)F_{CCBbAa}(i-1) + 0.125\Phi(1-\Phi)M_{CcBbAa}(i-1)\Phi(1-\Phi)F_{CCBbAa}(i-1) + 0.125\Phi(1-\Phi)M_{CcBbAa}(i-$
$1)(1-\Phi)\Phi F_{CCBbAa}(i-1) + 0.25\Phi(1-\Phi)M_{CcBbAa}(i-1)\Phi\Phi F_{CCBbAa}(i-1) + 0.125\Phi(1-\Phi)M_{CcBbAa}(i-$
$1)(1-\Phi)F_{CCBbAA}(i-1) + 0.25\Phi(1-\Phi)M_{CcBbAa}(i-1)\Phi F_{CCBbAA}(i-1) + 0.125\Phi(1-\Phi)M_{CcBbAa}(i-$
$1)(1-\Phi)F_{CCBBAa}(i-1) + 0.25\Phi(1-\Phi)M_{CcBbAa}(i-1)\Phi F_{CCBBAa}(i-1) + 0.25\Phi(1-\Phi)M_{CcBbAa}(i-$
$1)F_{CCBBAA}(i-1) + 0.0625(1-\Phi)\Phi M_{CcBbAa}(i-1)(1-\Phi)F_{ccBbAa}(i-1) + 0.125(1-\Phi)\Phi M_{CcBbAa}(i-$
$1)\Phi F_{ccBbAa}(i-1) + 0.125(1-\Phi)\Phi M_{CcBbAa}(i-1)F_{ccBbAA}(i-1) + 0.125(1-\Phi)\Phi M_{CcBbAa}(i-1)(1-$
$\Phi)F_{ccBBAa}(i-1) + 0.25(1-\Phi)\Phi M_{CcBbAa}(i-1)\Phi F_{ccBBAa}(i-1) + 0.25(1-\Phi)\Phi M_{CcBbAa}(i-1)F_{ccBBAA}(i-$
$1) + 0.0625(1-\Phi)\Phi M_{CcBbAa}(i-1)(1-\Phi)(1-\Phi)F_{CcBbAa}(i-1) + 0.125(1-\Phi)\Phi M_{CcBbAa}(i-1)\Phi(1-$
$\Phi)F_{CcBbAa}(i-1) + 0.125(1-\Phi)\Phi M_{CcBbAa}(i-1)(1-\Phi)\Phi F_{CcBbAa}(i-1) + 0.25(1-\Phi)\Phi M_{CcBbAa}(i-$
$1)\Phi\Phi F_{CcBbAa}(i-1) + 0.125(1-\Phi)\Phi M_{CcBbAa}(i-1)(1-\Phi)F_{CcBbAA}(i-1) + 0.25(1-\Phi)\Phi M_{CcBbAa}(i-$
$1)\Phi F_{CcBbAA}(i-1) + 0.125(1-\Phi)\Phi M_{CcBbAa}(i-1)(1-\Phi)F_{CcBBAa}(i-1) + 0.25(1-\Phi)\Phi M_{CcBbAa}(i-$
$1)\Phi F_{CcBBAa}(i-1) + 0.25(1-\Phi)\Phi M_{CcBbAa}(i-1)F_{CcBBAA}(i-1) + 0.0625(1-\Phi)\Phi M_{CcBbAa}(i-1)(1-\Phi)(1-$

$\Phi)F_{CCBbAa}(i-1)+0.125(1-\Phi)\Phi M_{CcBbAa}(i-1)\Phi(1-\Phi)F_{CCBbAa}(i-1)+0.125(1-\Phi)\Phi M_{CcBbAa}(i-$
$1)(1-\Phi)\Phi F_{CCBbAa}(i-1)+0.25(1-\Phi)\Phi M_{CcBbAa}(i-1)\Phi\Phi F_{CCBbAa}(i-1)+0.125(1-\Phi)\Phi M_{CcBbAa}(i-$
$1)(1-\Phi)F_{CCBbAA}(i-1)+0.25(1-\Phi)\Phi M_{CcBbAa}(i-1)\Phi F_{CCBbAA}(i-1)+0.125(1-\Phi)\Phi M_{CcBbAa}(i-$
$1)(1-\Phi)F_{CCBBAa}(i-1)+0.25(1-\Phi)\Phi M_{CcBbAa}(i-1)\Phi F_{CCBBAa}(i-1)+0.25(1-\Phi)\Phi M_{CcBbAa}(i-$
$1)F_{CCBBAA}(i-1)+0.125\Phi\Phi M_{CcBbAa}(i-1)(1-\Phi)F_{ccBbAa}(i-1)+0.25\Phi\Phi M_{CcBbAa}(i-1)\Phi F_{ccBbAa}(i-1)+$
$0.25\Phi\Phi M_{CcBbAa}(i-1)F_{ccBbAA}(i-1)+0.25\Phi\Phi M_{CcBbAa}(i-1)(1-\Phi)F_{ccBBAa}(i-1)+0.5\Phi\Phi M_{CcBbAa}(i-$
$1)\Phi F_{ccBBAa}(i-1)+0.5\Phi\Phi M_{CcBbAa}(i-1)F_{ccBBAA}(i-1)+0.125\Phi\Phi M_{CcBbAa}(i-1)(1-\Phi)(1-\Phi)F_{CcBbAa}(i-$
$1)+0.25\Phi\Phi M_{CcBbAa}(i-1)\Phi(1-\Phi)F_{CcBbAa}(i-1)+0.25\Phi\Phi M_{CcBbAa}(i-1)(1-\Phi)\Phi F_{CcBbAa}(i-1)+$
$0.5\Phi\Phi M_{CcBbAa}(i-1)\Phi\Phi F_{CcBbAa}(i-1)+0.25\Phi\Phi M_{CcBbAa}(i-1)(1-\Phi)F_{CcBbAA}(i-1)+0.5\Phi\Phi M_{CcBbAa}(i-$
$1)\Phi F_{CcBbAA}(i-1)+0.25\Phi\Phi M_{CcBbAa}(i-1)(1-\Phi)F_{CcBBAa}(i-1)+0.5\Phi\Phi M_{CcBbAa}(i-1)\Phi F_{CcBBAa}(i-$
$1)+0.5\Phi\Phi M_{CcBbAa}(i-1)F_{CcBBAA}(i-1)+0.125\Phi\Phi M_{CcBbAa}(i-1)(1-\Phi)(1-\Phi)F_{CCBbAa}(i-1)+$
$0.25\Phi\Phi M_{CcBbAa}(i-1)\Phi(1-\Phi)F_{CCBbAa}(i-1)+0.25\Phi\Phi M_{CcBbAa}(i-1)(1-\Phi)\Phi F_{CCBbAa}(i-1)+$
$0.5\Phi\Phi M_{CcBbAa}(i-1)\Phi\Phi F_{CCBbAa}(i-1)+0.25\Phi\Phi M_{CcBbAa}(i-1)(1-\Phi)F_{CCBbAA}(i-1)+0.5\Phi\Phi M_{CcBbAa}(i-$
$1)\Phi F_{CCBbAA}(i-1)+0.25\Phi\Phi M_{CcBbAa}(i-1)(1-\Phi)F_{CCBBAa}(i-1)+0.5\Phi\Phi M_{CcBbAa}(i-1)\Phi F_{CCBBAa}(i-$
$1)+0.5\Phi\Phi M_{CcBbAa}(i-1)F_{CCBBAA}(i-1)+0.0625(1-\Phi)M_{CcBbAA}(i-1)(1-\Phi)F_{ccBbAa}(i-1)+0.125(1-$
$\Phi)M_{CcBbAA}(i-1)\Phi F_{ccBbAa}(i-1)+0.125(1-\Phi)M_{CcBbAA}(i-1)F_{ccBbAA}(i-1)+0.125(1-\Phi)M_{CcBbAA}(i-$
$1)(1-\Phi)F_{ccBBAa}(i-1)+0.25(1-\Phi)M_{CcBbAA}(i-1)\Phi F_{ccBBAa}(i-1)+0.25(1-\Phi)M_{CcBbAA}(i-1)F_{ccBBAA}(i-$
$1)+0.0625(1-\Phi)M_{CcBbAA}(i-1)(1-\Phi)(1-\Phi)F_{CcBbAa}(i-1)+0.125(1-\Phi)M_{CcBbAA}(i-1)\Phi(1-$
$\Phi)F_{CcBbAa}(i-1)+0.125(1-\Phi)M_{CcBbAA}(i-1)(1-\Phi)\Phi F_{CcBbAa}(i-1)+0.25(1-\Phi)M_{CcBbAA}(i-$
$1)\Phi\Phi F_{CcBbAa}(i-1)+0.125(1-\Phi)M_{CcBbAA}(i-1)(1-\Phi)F_{CcBbAA}(i-1)+0.25(1-\Phi)M_{CcBbAA}(i-$
$1)\Phi F_{CcBbAA}(i-1)+0.125(1-\Phi)M_{CcBbAA}(i-1)(1-\Phi)F_{CcBBAa}(i-1)+0.25(1-\Phi)M_{CcBbAA}(i-$
$1)\Phi F_{CcBBAa}(i-1)+0.25(1-\Phi)M_{CcBbAA}(i-1)F_{CcBBAA}(i-1)+0.0625(1-\Phi)M_{CcBbAA}(i-1)(1-\Phi)(1-$
$\Phi)F_{CCBbAa}(i-1)+0.125(1-\Phi)M_{CcBbAA}(i-1)\Phi(1-\Phi)F_{CCBbAa}(i-1)+0.125(1-\Phi)M_{CcBbAA}(i-$
$1)(1-\Phi)\Phi F_{CCBbAa}(i-1)+0.25(1-\Phi)M_{CcBbAA}(i-1)\Phi\Phi F_{CCBbAa}(i-1)+0.125(1-\Phi)M_{CcBbAA}(i-$
$1)(1-\Phi)F_{CCBbAA}(i-1)+0.25(1-\Phi)M_{CcBbAA}(i-1)\Phi F_{CCBbAA}(i-1)+0.125(1-\Phi)M_{CcBbAA}(i-$
$1)(1-\Phi)F_{CCBBAa}(i-1)+0.25(1-\Phi)M_{CcBbAA}(i-1)\Phi F_{CCBBAa}(i-1)+0.25(1-\Phi)M_{CcBbAA}(i-$
$1)F_{CCBBAA}(i-1)+0.125\Phi M_{CcBbAA}(i-1)(1-\Phi)F_{ccBbAa}(i-1)+0.25\Phi M_{CcBbAA}(i-1)\Phi F_{ccBbAa}(i-1)+$
$0.25\Phi M_{CcBbAA}(i-1)F_{ccBbAA}(i-1)+0.25\Phi M_{CcBbAA}(i-1)(1-\Phi)F_{ccBBAa}(i-1)+0.5\Phi M_{CcBbAA}(i-$
$1)\Phi F_{ccBBAa}(i-1)+0.5\Phi M_{CcBbAA}(i-1)F_{ccBBAA}(i-1)+0.125\Phi M_{CcBbAA}(i-1)(1-\Phi)(1-\Phi)F_{CcBbAa}(i-$
$1)+0.25\Phi M_{CcBbAA}(i-1)\Phi(1-\Phi)F_{CcBbAa}(i-1)+0.25\Phi M_{CcBbAA}(i-1)(1-\Phi)\Phi F_{CcBbAa}(i-1)+$
$0.5\Phi M_{CcBbAA}(i-1)\Phi\Phi F_{CcBbAa}(i-1)+0.25\Phi M_{CcBbAA}(i-1)(1-\Phi)F_{CcBbAA}(i-1)+0.5\Phi M_{CcBbAA}(i-$
$1)\Phi F_{CcBbAA}(i-1)+0.25\Phi M_{CcBbAA}(i-1)(1-\Phi)F_{CcBBAa}(i-1)+0.5\Phi M_{CcBbAA}(i-1)\Phi F_{CcBBAa}(i-$
$1)+0.5\Phi M_{CcBbAA}(i-1)F_{CcBBAA}(i-1)+0.125\Phi M_{CcBbAA}(i-1)(1-\Phi)(1-\Phi)F_{CCBbAa}(i-1)+$
$0.25\Phi M_{CcBbAA}(i-1)\Phi(1-\Phi)F_{CCBbAa}(i-1)+0.25\Phi M_{CcBbAA}(i-1)(1-\Phi)\Phi F_{CCBbAa}(i-1)+0.5\Phi M_{CcBbAA}(i-$
$1)\Phi\Phi F_{CCBbAa}(i-1)+0.25\Phi M_{CcBbAA}(i-1)(1-\Phi)F_{CCBbAA}(i-1)+0.5\Phi M_{CcBbAA}(i-1)\Phi F_{CCBbAA}(i-1)+$
$0.25\Phi M_{CcBbAA}(i-1)(1-\Phi)F_{CCBBAa}(i-1)+0.5\Phi M_{CcBbAA}(i-1)\Phi F_{CCBBAa}(i-1)+0.5\Phi M_{CcBbAA}(i-$
$1)F_{CCBBAA}(i-1)+0.0625(1-\Phi)M_{CcBBAa}(i-1)(1-\Phi)F_{ccBbAa}(i-1)+0.125(1-\Phi)M_{CcBBAa}(i-$
$1)\Phi F_{ccBbAa}(i-1)+0.125(1-\Phi)M_{CcBBAa}(i-1)F_{ccBbAA}(i-1)+0.125(1-\Phi)M_{CcBBAa}(i-1)(1-$
$\Phi)F_{ccBBAa}(i-1)+0.25(1-\Phi)M_{CcBBAa}(i-1)\Phi F_{ccBBAa}(i-1)+0.25(1-\Phi)M_{CcBBAa}(i-1)F_{ccBBAA}(i-1)+$
$0.0625(1-\Phi)M_{CcBBAa}(i-1)(1-\Phi)(1-\Phi)F_{CcBbAa}(i-1)+0.125(1-\Phi)M_{CcBBAa}(i-1)\Phi(1-\Phi)F_{CcBbAa}(i-$
$1)+0.125(1-\Phi)M_{CcBBAa}(i-1)(1-\Phi)\Phi F_{CcBbAa}(i-1)+0.25(1-\Phi)M_{CcBBAa}(i-1)\Phi\Phi F_{CcBbAa}(i-$
$1)+0.125(1-\Phi)M_{CcBBAa}(i-1)(1-\Phi)F_{CcBbAA}(i-1)+0.25(1-\Phi)M_{CcBBAa}(i-1)\Phi F_{CcBbAA}(i-1)+$
$0.125(1-\Phi)M_{CcBBAa}(i-1)(1-\Phi)F_{CcBBAa}(i-1)+0.25(1-\Phi)M_{CcBBAa}(i-1)\Phi F_{CcBBAa}(i-1)+0.25(1-$
$\Phi)M_{CcBBAa}(i-1)F_{CcBBAA}(i-1)+0.0625(1-\Phi)M_{CcBBAa}(i-1)(1-\Phi)(1-\Phi)F_{CCBbAa}(i-1)+0.125(1-$
$\Phi)M_{CcBBAa}(i-1)\Phi(1-\Phi)F_{CCBbAa}(i-1)+0.125(1-\Phi)M_{CcBBAa}(i-1)(1-\Phi)\Phi F_{CCBbAa}(i-1)+0.25(1-$
$\Phi)M_{CcBBAa}(i-1)\Phi\Phi F_{CCBbAa}(i-1)+0.125(1-\Phi)M_{CcBBAa}(i-1)(1-\Phi)F_{CCBbAA}(i-1)+0.25(1-$
$\Phi)M_{CcBBAa}(i-1)\Phi F_{CCBbAA}(i-1)+0.125(1-\Phi)M_{CcBBAa}(i-1)(1-\Phi)F_{CCBBAa}(i-1)+0.25(1-$
$\Phi)M_{CcBBAa}(i-1)\Phi F_{CCBBAa}(i-1)+0.25(1-\Phi)M_{CcBBAa}(i-1)F_{CCBBAA}(i-1)+0.125\Phi M_{CcBBAa}(i-$
$1)(1-\Phi)F_{ccBbAa}(i-1)+0.25\Phi M_{CcBBAa}(i-1)\Phi F_{ccBbAa}(i-1)+0.25\Phi M_{CcBBAa}(i-1)F_{ccBbAA}(i-1)+$
$0.25\Phi M_{CcBBAa}(i-1)(1-\Phi)F_{ccBBAa}(i-1)+0.5\Phi M_{CcBBAa}(i-1)\Phi F_{ccBBAa}(i-1)+0.5\Phi M_{CcBBAa}(i-$
$1)F_{ccBBAA}(i-1)+0.125\Phi M_{CcBBAa}(i-1)(1-\Phi)(1-\Phi)F_{CcBbAa}(i-1)+0.25\Phi M_{CcBBAa}(i-1)\Phi(1-$
$\Phi)F_{CcBbAa}(i-1)+0.25\Phi M_{CcBBAa}(i-1)(1-\Phi)\Phi F_{CcBbAa}(i-1)+0.5\Phi M_{CcBBAa}(i-1)\Phi\Phi F_{CcBbAa}(i-1)+$
$0.25\Phi M_{CcBBAa}(i-1)(1-\Phi)F_{CcBbAA}(i-1)+0.5\Phi M_{CcBBAa}(i-1)\Phi F_{CcBbAA}(i-1)+0.25\Phi M_{CcBBAa}(i-$
$1)(1-\Phi)F_{CcBBAa}(i-1)+0.5\Phi M_{CcBBAa}(i-1)\Phi F_{CcBBAa}(i-1)+0.5\Phi M_{CcBBAa}(i-1)F_{CcBBAA}(i-1)+$
$0.125\Phi M_{CcBBAa}(i-1)(1-\Phi)(1-\Phi)F_{CCBbAa}(i-1)+0.25\Phi M_{CcBBAa}(i-1)\Phi(1-\Phi)F_{CCBbAa}(i-1)+$
$0.25\Phi M_{CcBBAa}(i-1)(1-\Phi)\Phi F_{CCBbAa}(i-1)+0.5\Phi M_{CcBBAa}(i-1)\Phi\Phi F_{CCBbAa}(i-1)+0.25\Phi M_{CcBBAa}(i-$
$1)(1-\Phi)F_{CCBbAA}(i-1)+0.5\Phi M_{CcBBAa}(i-1)\Phi F_{CCBbAA}(i-1)+0.25\Phi M_{CcBBAa}(i-1)(1-\Phi)F_{CCBBAa}(i-$

$1) + 0.5\Phi M_{CcBBAa}(i-1)\Phi F_{CCBBAa}(i-1) + 0.5\Phi M_{CcBBAa}(i-1)F_{CCBBAA}(i-1) + 0.125M_{CcBBAA}(i-$
$1)(1-\Phi)F_{ccBbAa}(i-1) + 0.25M_{CcBBAA}(i-1)\Phi F_{ccBbAa}(i-1) + 0.25M_{CcBBAA}(i-1)F_{ccBbAA}(i-1)+$
$0.25M_{CcBBAA}(i-1)(1-\Phi)F_{ccBBAa}(i-1) + 0.5M_{CcBBAA}(i-1)\Phi F_{ccBBAa}(i-1) + 0.5M_{CcBBAA}(i-$
$1)F_{ccBBAA}(i-1) + 0.125M_{CcBBAA}(i-1)(1-\Phi)(1-\Phi)F_{CcBbAa}(i-1) + 0.25M_{CcBBAA}(i-1)\Phi(1-$
$\Phi)F_{CcBbAa}(i-1)+0.25M_{CcBBAA}(i-1)(1-\Phi)\Phi F_{CcBbAa}(i-1)+0.5M_{CcBBAA}(i-1)\Phi\Phi F_{CcBbAa}(i-1)+$
$0.25M_{CcBBAA}(i-1)(1-\Phi)F_{CcBbAA}(i-1) + 0.5M_{CcBBAA}(i-1)\Phi F_{CcBbAA}(i-1) + 0.25M_{CcBBAA}(i-$
$1)(1-\Phi)F_{CcBBAa}(i-1) + 0.5M_{CcBBAA}(i-1)\Phi F_{CcBBAa}(i-1) + 0.5M_{CcBBAA}(i-1)F_{CcBBAA}(i-1)+$
$0.125M_{CcBBAA}(i-1)(1-\Phi)(1-\Phi)F_{CCBbAa}(i-1) + 0.25M_{CcBBAA}(i-1)\Phi(1-\Phi)F_{CCBbAa}(i-1)+$
$0.25M_{CcBBAA}(i-1)(1-\Phi)\Phi F_{CCBbAa}(i-1)+0.5M_{CcBBAA}(i-1)\Phi\Phi F_{CCBbAa}(i-1)+0.25M_{CcBBAA}(i-$
$1)(1-\Phi)F_{CCBbAA}(i-1)+0.5M_{CcBBAA}(i-1)\Phi F_{CCBbAA}(i-1)+0.25M_{CcBBAA}(i-1)(1-\Phi)F_{CCBBAa}(i-$
$1) + 0.5M_{CcBBAA}(i-1)\Phi F_{CCBBAa}(i-1) + 0.5M_{CcBBAA}(i-1)F_{CCBBAA}(i-1) + 0.0625(1-\Phi)(1-$
$\Phi)M_{CCBbAa}(i-1)(1-\Phi)F_{ccBbAa}(i-1)+0.125(1-\Phi)(1-\Phi)M_{CCBbAa}(i-1)\Phi F_{ccBbAa}(i-1)+0.125(1-$
$\Phi)(1-\Phi)M_{CCBbAa}(i-1)F_{ccBbAA}(i-1) + 0.125(1-\Phi)(1-\Phi)M_{CCBbAa}(i-1)(1-\Phi)F_{ccBBAa}(i-1)+$
$0.25(1-\Phi)(1-\Phi)M_{CCBbAa}(i-1)\Phi F_{ccBBAa}(i-1)+0.25(1-\Phi)(1-\Phi)M_{CCBbAa}(i-1)F_{ccBBAA}(i-1)+$
$0.03125(1-\Phi)(1-\Phi)M_{CCBbAa}(i-1)(1-\Phi)(1-\Phi)F_{CcBbAa}(i-1)+0.0625(1-\Phi)(1-\Phi)M_{CCBbAa}(i-$
$1)\Phi(1-\Phi)F_{CcBbAa}(i-1) + 0.0625(1-\Phi)(1-\Phi)M_{CCBbAa}(i-1)(1-\Phi)\Phi F_{CcBbAa}(i-1) + 0.125(1-$
$\Phi)(1-\Phi)M_{CCBbAa}(i-1)\Phi\Phi F_{CcBbAa}(i-1) + 0.0625(1-\Phi)(1-\Phi)M_{CCBbAa}(i-1)(1-\Phi)F_{CcBbAA}(i-$
$1) + 0.125(1-\Phi)(1-\Phi)M_{CCBbAa}(i-1)\Phi F_{CcBbAA}(i-1) + 0.0625(1-\Phi)(1-\Phi)M_{CCBbAa}(i-1)(1-$
$\Phi)F_{CcBBAa}(i-1)+0.125(1-\Phi)(1-\Phi)M_{CCBbAa}(i-1)\Phi F_{CcBBAa}(i-1)+0.125(1-\Phi)(1-\Phi)M_{CCBbAa}(i-$
$1)F_{CcBBAA}(i-1) + 0.125\Phi(1-\Phi)M_{CCBbAa}(i-1)(1-\Phi)F_{ccBbAa}(i-1) + 0.25\Phi(1-\Phi)M_{CCBbAa}(i-$
$1)\Phi F_{ccBbAa}(i-1) + 0.25\Phi(1-\Phi)M_{CCBbAa}(i-1)F_{ccBbAA}(i-1) + 0.25\Phi(1-\Phi)M_{CCBbAa}(i-1)(1-$
$\Phi)F_{ccBBAa}(i-1)+0.5\Phi(1-\Phi)M_{CCBbAa}(i-1)\Phi F_{ccBBAa}(i-1)+0.5\Phi(1-\Phi)M_{CCBbAa}(i-1)F_{ccBBAA}(i-$
$1) + 0.0625\Phi(1-\Phi)M_{CCBbAa}(i-1)(1-\Phi)(1-\Phi)F_{CcBbAa}(i-1) + 0.125\Phi(1-\Phi)M_{CCBbAa}(i-1)\Phi(1-$
$\Phi)F_{CcBbAa}(i-1) + 0.125\Phi(1-\Phi)M_{CCBbAa}(i-1)(1-\Phi)\Phi F_{CcBbAa}(i-1) + 0.25\Phi(1-\Phi)M_{CCBbAa}(i-$
$1)\Phi\Phi F_{CcBbAa}(i-1) + 0.125\Phi(1-\Phi)M_{CCBbAa}(i-1)(1-\Phi)F_{CcBbAA}(i-1) + 0.25\Phi(1-\Phi)M_{CCBbAa}(i-$
$1)\Phi F_{CcBbAA}(i-1) + 0.125\Phi(1-\Phi)M_{CCBbAa}(i-1)(1-\Phi)F_{CcBBAa}(i-1) + 0.25\Phi(1-\Phi)M_{CCBbAa}(i-$
$1)\Phi F_{CcBBAa}(i-1) + 0.25\Phi(1-\Phi)M_{CCBbAa}(i-1)F_{CcBBAA}(i-1) + 0.125(1-\Phi)\Phi M_{CCBbAa}(i-1)(1-$
$\Phi)F_{ccBbAa}(i-1)+0.25(1-\Phi)\Phi M_{CCBbAa}(i-1)\Phi F_{ccBbAa}(i-1)+0.25(1-\Phi)\Phi M_{CCBbAa}(i-1)F_{ccBbAA}(i-$
$1) + 0.25(1-\Phi)\Phi M_{CCBbAa}(i-1)(1-\Phi)F_{ccBBAa}(i-1) + 0.5(1-\Phi)\Phi M_{CCBbAa}(i-1)\Phi F_{ccBBAa}(i-1)+$
$0.5(1-\Phi)\Phi M_{CCBbAa}(i-1)F_{ccBBAA}(i-1)+0.0625(1-\Phi)\Phi M_{CCBbAa}(i-1)(1-\Phi)(1-\Phi)F_{CcBbAa}(i-1)+$
$0.125(1-\Phi)\Phi M_{CCBbAa}(i-1)\Phi(1-\Phi)F_{CcBbAa}(i-1)+0.125(1-\Phi)\Phi M_{CCBbAa}(i-1)(1-\Phi)\Phi F_{CcBbAa}(i-$
$1)+0.25(1-\Phi)\Phi M_{CCBbAa}(i-1)\Phi\Phi F_{CcBbAa}(i-1)+0.125(1-\Phi)\Phi M_{CCBbAa}(i-1)(1-\Phi)F_{CcBbAA}(i-1)+$
$0.25(1-\Phi)\Phi M_{CCBbAa}(i-1)\Phi F_{CcBbAA}(i-1)+0.125(1-\Phi)\Phi M_{CCBbAa}(i-1)(1-\Phi)F_{CcBBAa}(i-1)+0.25(1-$
$\Phi)\Phi M_{CCBbAa}(i-1)\Phi F_{CcBBAa}(i-1)+0.25(1-\Phi)\Phi M_{CCBbAa}(i-1)F_{CcBBAA}(i-1)+0.25\Phi\Phi M_{CCBbAa}(i-$
$1)(1-\Phi)F_{ccBbAa}(i-1)+0.5\Phi\Phi M_{CCBbAa}(i-1)\Phi F_{ccBbAa}(i-1)+0.5\Phi\Phi M_{CCBbAa}(i-1)F_{ccBbAA}(i-1)+$
$0.5\Phi\Phi M_{CCBbAa}(i-1)(1-\Phi)F_{ccBBAa}(i-1) + \Phi\Phi M_{CCBbAa}(i-1)\Phi F_{ccBBAa}(i-1) + \Phi\Phi M_{CCBbAa}(i-$
$1)F_{ccBBAA}(i-1)+0.125\Phi\Phi M_{CCBbAa}(i-1)(1-\Phi)(1-\Phi)F_{CcBbAa}(i-1)+0.25\Phi\Phi M_{CCBbAa}(i-1)\Phi(1-$
$\Phi)F_{CcBbAa}(i-1)+0.25\Phi\Phi M_{CCBbAa}(i-1)(1-\Phi)\Phi F_{CcBbAa}(i-1)+0.5\Phi\Phi M_{CCBbAa}(i-1)\Phi\Phi F_{CcBbAa}(i-$
$1)+0.25\Phi\Phi M_{CCBbAa}(i-1)(1-\Phi)F_{CcBbAA}(i-1)+0.5\Phi\Phi M_{CCBbAa}(i-1)\Phi F_{CcBbAA}(i-1)+0.25\Phi\Phi M_{CCBbAa}(i-$
$1)(1-\Phi)F_{CcBBAa}(i-1)+0.5\Phi\Phi M_{CCBbAa}(i-1)\Phi F_{CcBBAa}(i-1)+0.5\Phi\Phi M_{CCBbAa}(i-1)F_{CcBBAA}(i-1)+$
$0.125(1-\Phi)M_{CCBbAA}(i-1)(1-\Phi)F_{ccBbAa}(i-1)+0.25(1-\Phi)M_{CCBbAA}(i-1)\Phi F_{ccBbAa}(i-1)+0.25(1-$
$\Phi)M_{CCBbAA}(i-1)F_{ccBbAA}(i-1)+0.25(1-\Phi)M_{CCBbAA}(i-1)(1-\Phi)F_{ccBBAa}(i-1)+0.5(1-\Phi)M_{CCBbAA}(i-$
$1)\Phi F_{ccBBAa}(i-1)+0.5(1-\Phi)M_{CCBbAA}(i-1)F_{ccBBAA}(i-1)+0.0625(1-\Phi)M_{CCBbAA}(i-1)(1-\Phi)(1-$
$\Phi)F_{CcBbAa}(i-1) + 0.125(1-\Phi)M_{CCBbAA}(i-1)\Phi(1-\Phi)F_{CcBbAa}(i-1) + 0.125(1-\Phi)M_{CCBbAA}(i-$
$1)(1-\Phi)\Phi F_{CcBbAa}(i-1) + 0.25(1-\Phi)M_{CCBbAA}(i-1)\Phi\Phi F_{CcBbAa}(i-1) + 0.125(1-\Phi)M_{CCBbAA}(i-$
$1)(1-\Phi)F_{CcBbAA}(i-1) + 0.25(1-\Phi)M_{CCBbAA}(i-1)\Phi F_{CcBbAA}(i-1) + 0.125(1-\Phi)M_{CCBbAA}(i-$
$1)(1-\Phi)F_{CcBBAa}(i-1) + 0.25(1-\Phi)M_{CCBbAA}(i-1)\Phi F_{CcBBAa}(i-1) + 0.25(1-\Phi)M_{CCBbAA}(i-$
$1)F_{CcBBAA}(i-1) + 0.25\Phi M_{CCBbAA}(i-1)(1-\Phi)F_{ccBbAa}(i-1) + 0.5\Phi M_{CCBbAA}(i-1)\Phi F_{ccBbAa}(i-$
$1) + 0.5\Phi M_{CCBbAA}(i-1)F_{ccBbAA}(i-1) + 0.5\Phi M_{CCBbAA}(i-1)(1-\Phi)F_{ccBBAa}(i-1) + \Phi M_{CCBbAA}(i-$
$1)\Phi F_{ccBBAa}(i-1)+\Phi M_{CCBbAA}(i-1)F_{ccBBAA}(i-1)+0.125\Phi M_{CCBbAA}(i-1)(1-\Phi)(1-\Phi)F_{CcBbAa}(i-$
$1) + 0.25\Phi M_{CCBbAA}(i-1)\Phi(1-\Phi)F_{CcBbAa}(i-1) + 0.25\Phi M_{CCBbAA}(i-1)(1-\Phi)\Phi F_{CcBbAa}(i-1)+$
$0.5\Phi M_{CCBbAA}(i-1)\Phi\Phi F_{CcBbAa}(i-1)+0.25\Phi M_{CCBbAA}(i-1)(1-\Phi)F_{CcBbAA}(i-1)+0.5\Phi M_{CCBbAA}(i-$
$1)\Phi F_{CcBbAA}(i-1) + 0.25\Phi M_{CCBbAA}(i-1)(1-\Phi)F_{CcBBAa}(i-1) + 0.5\Phi M_{CCBbAA}(i-1)\Phi F_{CcBBAa}(i-$
$1)+0.5\Phi M_{CCBbAA}(i-1)F_{CcBBAA}(i-1)+0.125(1-\Phi)M_{CCBBAa}(i-1)(1-\Phi)F_{ccBbAa}(i-1)+0.25(1-$
$\Phi)M_{CCBBAa}(i-1)\Phi F_{ccBbAa}(i-1)+0.25(1-\Phi)M_{CCBBAa}(i-1)F_{ccBbAA}(i-1)+0.25(1-\Phi)M_{CCBBAa}(i-$
$1)(1-\Phi)F_{ccBBAa}(i-1)+0.5(1-\Phi)M_{CCBBAa}(i-1)\Phi F_{ccBBAa}(i-1)+0.5(1-\Phi)M_{CCBBAa}(i-1)F_{ccBBAA}(i-$
$1) + 0.0625(1-\Phi)M_{CCBBAa}(i-1)(1-\Phi)(1-\Phi)F_{CcBbAa}(i-1) + 0.125(1-\Phi)M_{CCBBAa}(i-1)\Phi(1-$

$$\Phi)F_{CcBbAa}(i-1) + 0.125(1-\Phi)M_{CCBBAa}(i-1)(1-\Phi)\Phi F_{CcBbAa}(i-1) + 0.25(1-\Phi)M_{CCBBAa}(i-1)\Phi\Phi F_{CcBbAa}(i-1) + 0.125(1-\Phi)M_{CCBBAa}(i-1)(1-\Phi)F_{CcBbAA}(i-1) + 0.25(1-\Phi)M_{CCBBAa}(i-1)\Phi F_{CcBbAA}(i-1) + 0.125(1-\Phi)M_{CCBBAa}(i-1)(1-\Phi)F_{CcBBAa}(i-1) + 0.25(1-\Phi)M_{CCBBAa}(i-1)\Phi F_{CcBBAa}(i-1)+0.25(1-\Phi)M_{CCBBAa}(i-1)F_{CcBBAA}(i-1)+0.25\Phi M_{CCBBAa}(i-1)(1-\Phi)F_{ccBbAa}(i-1) + 0.5\Phi M_{CCBBAa}(i-1)\Phi F_{ccBbAa}(i-1) + 0.5\Phi M_{CCBBAa}(i-1)F_{ccBbAA}(i-1) + 0.5\Phi M_{CCBBAa}(i-1)(1-\Phi)F_{ccBBAa}(i-1) + \Phi M_{CCBBAa}(i-1)\Phi F_{ccBBAa}(i-1) + \Phi M_{CCBBAa}(i-1)F_{ccBBAA}(i-1) + 0.125\Phi M_{CCBBAa}(i-1)(1-\Phi)(1-\Phi)F_{CcBbAa}(i-1) + 0.25\Phi M_{CCBBAa}(i-1)\Phi(1-\Phi)F_{CcBbAa}(i-1) + 0.25\Phi M_{CCBBAa}(i-1)(1-\Phi)\Phi F_{CcBbAa}(i-1)+0.5\Phi M_{CCBBAa}(i-1)\Phi\Phi F_{CcBbAa}(i-1)+0.25\Phi M_{CCBBAa}(i-1)(1-\Phi)F_{CcBbAA}(i-1)+0.5\Phi M_{CCBBAa}(i-1)\Phi F_{CcBbAA}(i-1)+0.25\Phi M_{CCBBAa}(i-1)(1-\Phi)F_{CcBBAa}(i-1) + 0.5\Phi M_{CCBBAa}(i-1)\Phi F_{CcBBAa}(i-1) + 0.5\Phi M_{CCBBAa}(i-1)F_{CcBBAA}(i-1) + 0.25M_{CCBBAA}(i-1)(1-\Phi)F_{ccBbAa}(i-1) + 0.5M_{CCBBAA}(i-1)\Phi F_{ccBbAa}(i-1) + 0.5M_{CCBBAA}(i-1)F_{ccBbAA}(i-1) + 0.5M_{CCBBAA}(i-1)(1-\Phi)F_{ccBBAa}(i-1)+M_{CCBBAA}(i-1)\Phi F_{ccBBAa}(i-1)+M_{CCBBAA}(i-1)F_{ccBBAA}(i-1)+0.125M_{CCBBAA}(i-1)(1-\Phi)(1-\Phi)F_{CcBbAa}(i-1)+0.25M_{CCBBAA}(i-1)\Phi(1-\Phi)F_{CcBbAa}(i-1)+ 0.25M_{CCBBAA}(i-1)(1-\Phi)\Phi F_{CcBbAa}(i-1)+0.5M_{CCBBAA}(i-1)\Phi\Phi F_{CcBbAa}(i-1)+0.25M_{CCBBAA}(i-1)(1-\Phi)F_{CcBbAA}(i-1)+0.5M_{CCBBAA}(i-1)\Phi F_{CcBbAA}(i-1)+0.25M_{CCBBAA}(i-1)(1-\Phi)F_{CcBBAa}(i-1) + 0.5M_{CCBBAA}(i-1)\Phi F_{CcBBAa}(i-1) + 0.5M_{CCBBAA}(i-1)F_{CcBBAA}(i-1)],$$

$$\mathrm{M}^e_{CCbbaa} = \left(\frac{\bar{\Omega}_{CCbbaa}}{2}\right)[0.25M_{Ccbbaa}(i-1)F_{Ccbbaa}(i-1)+0.125M_{Ccbbaa}(i-1)F_{CcbbAa}(i-1)+0.125M_{Ccbbaa}(i-1)(1-\Phi)F_{CcBbaa}(i-1)+0.0625M_{Ccbbaa}(i-1)(1-\Phi)(1-\Phi)F_{CcBbAa}(i-1)+0.5M_{Ccbbaa}(i-1)F_{CCbbaa}(i-1) + 0.25M_{Ccbbaa}(i-1)F_{CCbbAa}(i-1) + 0.25M_{Ccbbaa}(i-1)(1-\Phi)F_{CCBbaa}(i-1) + 0.125M_{Ccbbaa}(i-1)(1-\Phi)(1-\Phi)F_{CCBbAa}(i-1) + 0.125M_{CcbbAa}(i-1)F_{Ccbbaa}(i-1) + 0.0625M_{CcbbAa}(i-1)F_{CcbbAa}(i-1) + 0.0625M_{CcbbAa}(i-1)(1-\Phi)F_{CcBbaa}(i-1) + 0.03125M_{CcbbAa}(i-1)(1-\Phi)(1-\Phi)F_{CcBbAa}(i-1) + 0.25M_{CcbbAa}(i-1)F_{CCbbaa}(i-1) + 0.125M_{CcbbAa}(i-1)F_{CCbbAa}(i-1) + 0.125M_{CcbbAa}(i-1)(1-\Phi)F_{CCBbaa}(i-1)+0.0625M_{CcbbAa}(i-1)(1-\Phi)(1-\Phi)F_{CCBbAa}(i-1)+0.125(1-\Phi)M_{CcBbaa}(i-1)F_{Ccbbaa}(i-1) + 0.0625(1-\Phi)M_{CcBbaa}(i-1)F_{CcbbAa}(i-1) + 0.0625(1-\Phi)M_{CcBbaa}(i-1)(1-\Phi)F_{CcBbaa}(i-1) + 0.03125(1-\Phi)M_{CcBbaa}(i-1)(1-\Phi)(1-\Phi)F_{CcBbAa}(i-1) + 0.25(1-\Phi)M_{CcBbaa}(i-1)F_{CCbbaa}(i-1) + 0.125(1-\Phi)M_{CcBbaa}(i-1)F_{CCbbAa}(i-1) + 0.125(1-\Phi)M_{CcBbaa}(i-1)(1-\Phi)F_{CCBbaa}(i-1) + 0.0625(1-\Phi)M_{CcBbaa}(i-1)(1-\Phi)(1-\Phi)F_{CCBbAa}(i-1)+0.0625(1-\Phi)(1-\Phi)M_{CcBbAa}(i-1)F_{Ccbbaa}(i-1) + 0.03125(1-\Phi)(1-\Phi)M_{CcBbAa}(i-1)F_{CcbbAa}(i-1) + 0.03125(1-\Phi)(1-\Phi)M_{CcBbAa}(i-1)(1-\Phi)F_{CcBbaa}(i-1)+0.015625(1-\Phi)(1-\Phi)M_{CcBbAa}(i-1)(1-\Phi)(1-\Phi)F_{CcBbAa}(i-1)+0.125(1-\Phi)(1-\Phi)M_{CcBbAa}(i-1)F_{CCbbaa}(i-1)+0.0625(1-\Phi)(1-\Phi)M_{CcBbAa}(i-1)F_{CCbbAa}(i-1)+0.0625(1-\Phi)(1-\Phi)M_{CcBbAa}(i-1)(1-\Phi)F_{CCBbaa}(i-1)+0.03125(1-\Phi)(1-\Phi)M_{CcBbAa}(i-1)(1-\Phi)(1-\Phi)F_{CCBbAa}(i-1)+ 0.5M_{CCbbaa}(i-1)F_{Ccbbaa}(i-1)+0.25M_{CCbbaa}(i-1)F_{CcbbAa}(i-1)+0.25M_{CCbbaa}(i-1)(1-\Phi)F_{CcBbaa}(i-1)+0.125M_{CCbbaa}(i-1)(1-\Phi)(1-\Phi)F_{CcBbAa}(i-1)+M_{CCbbaa}(i-1)F_{CCbbaa}(i-1)+0.5M_{CCbbaa}(i-1)F_{CCbbAa}(i-1)+0.5M_{CCbbaa}(i-1)(1-\Phi)F_{CCBbaa}(i-1)+0.25M_{CCbbaa}(i-1)(1-\Phi)(1-\Phi)F_{CCBbAa}(i-1) + 0.25M_{CCbbAa}(i-1)F_{Ccbbaa}(i-1) + 0.125M_{CCbbAa}(i-1)F_{CcbbAa}(i-1) + 0.125M_{CCbbAa}(i-1)(1-\Phi)F_{CcBbaa}(i-1) + 0.0625M_{CCbbAa}(i-1)(1-\Phi)(1-\Phi)F_{CcBbAa}(i-1) + 0.5M_{CCbbAa}(i-1)F_{CCbbaa}(i-1) + 0.25M_{CCbbAa}(i-1)F_{CCbbAa}(i-1) + 0.25M_{CCbbAa}(i-1)(1-\Phi)F_{CCBbaa}(i-1) + 0.125M_{CCbbAa}(i-1)(1-\Phi)(1-\Phi)F_{CCBbAa}(i-1) + 0.25(1-\Phi)M_{CCBbaa}(i-1)F_{Ccbbaa}(i-1) + 0.125(1-\Phi)M_{CCBbaa}(i-1)F_{CcbbAa}(i-1)+0.125(1-\Phi)M_{CCBbaa}(i-1)(1-\Phi)F_{CcBbaa}(i-1)+0.0625(1-\Phi)M_{CCBbaa}(i-1)(1-\Phi)(1-\Phi)F_{CcBbAa}(i-1)+0.5(1-\Phi)M_{CCBbaa}(i-1)F_{CCbbaa}(i-1)+0.25(1-\Phi)M_{CCBbaa}(i-1)F_{CCbbAa}(i-1)+ 0.25(1-\Phi)M_{CCBbaa}(i-1)(1-\Phi)F_{CCBbaa}(i-1)+0.125(1-\Phi)M_{CCBbaa}(i-1)(1-\Phi)(1-\Phi)F_{CCBbAa}(i-1)+0.125(1-\Phi)(1-\Phi)M_{CCBbAa}(i-1)F_{Ccbbaa}(i-1)+0.0625(1-\Phi)(1-\Phi)M_{CCBbAa}(i-1)F_{CcbbAa}(i-1) + 0.0625(1-\Phi)(1-\Phi)M_{CCBbAa}(i-1)(1-\Phi)F_{CcBbaa}(i-1) + 0.03125(1-\Phi)(1-\Phi)M_{CCBbAa}(i-1)(1-\Phi)(1-\Phi)F_{CcBbAa}(i-1) + 0.25(1-\Phi)(1-\Phi)M_{CCBbAa}(i-1)F_{CCbbaa}(i-1) + 0.125(1-\Phi)(1-\Phi)M_{CCBbAa}(i-1)F_{CCbbAa}(i-1)+0.125(1-\Phi)(1-\Phi)M_{CCBbAa}(i-1)(1-\Phi)F_{CCBbaa}(i-1)+0.0625(1-\Phi)(1-\Phi)M_{CCBbAa}(i-1)(1-\Phi)(1-\Phi)F_{CCBbAa}(i-1)],$$

$$\mathrm{M}^e_{CCbbAa} = \left(\frac{\bar{\Omega}_{CCbbAa}}{2}\right)[0.125M_{Ccbbaa}(i-1)F_{CcbbAa}(i-1) + 0.25M_{Ccbbaa}(i-1)F_{CcbbAA}(i-1) + 0.0625M_{Ccbbaa}(i-1)(1-\Phi)(1-\Phi)F_{CcBbAa}(i-1) + 0.125M_{Ccbbaa}(i-1)(1-\Phi)\Phi F_{CcBbAa}(i-1) + 0.125M_{Ccbbaa}(i-1)(1-\Phi)F_{CcBbAA}(i-1)+0.25M_{Ccbbaa}(i-1)F_{CCbbAa}(i-1)+0.5M_{Ccbbaa}(i-1)F_{CCbbAA}(i-1) + 0.125M_{Ccbbaa}(i-1)(1-\Phi)(1-\Phi)F_{CCBbAa}(i-1) + 0.25M_{Ccbbaa}(i-1)(1-\Phi)F_{CCBbAA}(i-1) + 0.125M_{CcbbAa}(i-1)F_{Ccbbaa}(i-1)+0.125M_{CcbbAa}(i-1)F_{CcbbAa}(i-1)+0.125M_{CcbbAa}(i-1)F_{CcbbAA}(i-1) + 0.0625M_{CcbbAa}(i-1)(1-\Phi)F_{CcBbaa}(i-1) + 0.0625M_{CcbbAa}(i-1)(1-\Phi)(1-\Phi)F_{CcBbAa}(i-1) + 0.0625M_{CcbbAa}(i-1)(1-\Phi)\Phi F_{CcBbAa}(i-1)+0.0625M_{CcbbAa}(i-1)(1-\Phi)F_{CcBbAA}(i-1)+0.25M_{CcbbAa}(i-1)F_{CCbbaa}(i-1)+0.25M_{CcbbAa}(i-1)F_{CCbbAa}(i-1)+0.25M_{CcbbAa}(i-1)F_{CCbbAA}(i-1)+0.125M_{CcbbAa}(i-1)(1-\Phi)F_{CCBbaa}(i-1) + 0.125M_{CcbbAa}(i-1)(1-\Phi)(1-\Phi)F_{CCBbAa}(i-1) + 0.125M_{CcbbAa}(i-1)(1-$$

$\Phi)F_{CCBbAA}(i-1)+0.25M_{CcbbAA}(i-1)F_{Ccbbaa}(i-1)+0.125M_{CcbbAA}(i-1)F_{CcbbAa}(i-1)+0.125M_{CcbbAA}(i-$
$1)(1-\Phi)F_{CcBbaa}(i-1)+0.0625M_{CcbbAA}(i-1)(1-\Phi)(1-\Phi)F_{CcBbAa}(i-1)+0.5M_{CcbbAA}(i-1)F_{CCbbaa}(i-$
$1)+0.25M_{CcbbAA}(i-1)F_{CCbbAa}(i-1)+0.25M_{CcbbAA}(i-1)(1-\Phi)F_{CCBbaa}(i-1)+0.125M_{CcbbAA}(i-$
$1)(1-\Phi)(1-\Phi)F_{CCBbAa}(i-1)+0.0625(1-\Phi)M_{CcBbaa}(i-1)F_{CcbbAa}(i-1)+0.125(1-\Phi)M_{CcBbaa}(i-$
$1)F_{CcbbAA}(i-1)+0.03125(1-\Phi)M_{CcBbaa}(i-1)(1-\Phi)(1-\Phi)F_{CcBbAa}(i-1)+0.0625(1-\Phi)M_{CcBbaa}(i-$
$1)(1-\Phi)\Phi F_{CcBbAa}(i-1)+0.0625(1-\Phi)M_{CcBbaa}(i-1)(1-\Phi)F_{CcBbAA}(i-1)+0.125(1-\Phi)M_{CcBbaa}(i-$
$1)F_{CCbbAa}(i-1)+0.25(1-\Phi)M_{CcBbaa}(i-1)F_{CCbbAA}(i-1)+0.0625(1-\Phi)M_{CcBbaa}(i-1)(1-\Phi)(1-$
$\Phi)F_{CCBbAa}(i-1)+0.125(1-\Phi)M_{CcBbaa}(i-1)(1-\Phi)F_{CCBbAA}(i-1)+0.0625(1-\Phi)(1-\Phi)M_{CcBbAa}(i-$
$1)F_{Ccbbaa}(i-1)+0.0625(1-\Phi)(1-\Phi)M_{CcBbAa}(i-1)F_{CcbbAa}(i-1)+0.0625(1-\Phi)(1-\Phi)M_{CcBbAa}(i-$
$1)F_{CcbbAA}(i-1)+0.03125(1-\Phi)(1-\Phi)M_{CcBbAa}(i-1)(1-\Phi)F_{CcBbaa}(i-1)+0.03125(1-\Phi)(1-$
$\Phi)M_{CcBbAa}(i-1)(1-\Phi)(1-\Phi)F_{CcBbAa}(i-1)+0.03125(1-\Phi)(1-\Phi)M_{CcBbAa}(i-1)(1-\Phi)\Phi F_{CcBbAa}(i-$
$1)+0.03125(1-\Phi)(1-\Phi)M_{CcBbAa}(i-1)(1-\Phi)F_{CcBbAA}(i-1)+0.125(1-\Phi)(1-\Phi)M_{CcBbAa}(i-$
$1)F_{CCbbaa}(i-1)+0.125(1-\Phi)(1-\Phi)M_{CcBbAa}(i-1)F_{CCbbAa}(i-1)+0.125(1-\Phi)(1-\Phi)M_{CcBbAa}(i-$
$1)F_{CCbbAA}(i-1)+0.0625(1-\Phi)(1-\Phi)M_{CcBbAa}(i-1)(1-\Phi)F_{CCBbaa}(i-1)+0.0625(1-\Phi)(1-$
$\Phi)M_{CcBbAa}(i-1)(1-\Phi)(1-\Phi)F_{CCBbAa}(i-1)+0.0625(1-\Phi)(1-\Phi)M_{CcBbAa}(i-1)(1-\Phi)F_{CCBbAA}(i-$
$1)+0.125(1-\Phi)\Phi M_{CcBbAa}(i-1)F_{Ccbbaa}(i-1)+0.0625(1-\Phi)\Phi M_{CcBbAa}(i-1)F_{CcbbAa}(i-1)+0.0625(1-$
$\Phi)\Phi M_{CcBbAa}(i-1)(1-\Phi)F_{CcBbaa}(i-1)+0.03125(1-\Phi)\Phi M_{CcBbAa}(i-1)(1-\Phi)(1-\Phi)F_{CcBbAa}(i-$
$1)+0.25(1-\Phi)\Phi M_{CcBbAa}(i-1)F_{CCbbaa}(i-1)+0.125(1-\Phi)\Phi M_{CcBbAa}(i-1)F_{CCbbAa}(i-1)+0.125(1-$
$\Phi)\Phi M_{CcBbAa}(i-1)(1-\Phi)F_{CCBbaa}(i-1)+0.0625(1-\Phi)\Phi M_{CcBbAa}(i-1)(1-\Phi)(1-\Phi)F_{CCBbAa}(i-$
$1)+0.125(1-\Phi)M_{CcBbAA}(i-1)F_{Ccbbaa}(i-1)+0.0625(1-\Phi)M_{CcBbAA}(i-1)F_{CcbbAa}(i-1)+0.0625(1-$
$\Phi)M_{CcBbAA}(i-1)(1-\Phi)F_{CcBbaa}(i-1)+0.03125(1-\Phi)M_{CcBbAA}(i-1)(1-\Phi)(1-\Phi)F_{CcBbAa}(i-1)+0.25(1-$
$\Phi)M_{CcBbAA}(i-1)F_{CCbbaa}(i-1)+0.125(1-\Phi)M_{CcBbAA}(i-1)F_{CCbbAa}(i-1)+0.125(1-\Phi)M_{CcBbAA}(i-$
$1)(1-\Phi)F_{CCBbaa}(i-1)+0.0625(1-\Phi)M_{CcBbAA}(i-1)(1-\Phi)(1-\Phi)F_{CCBbAa}(i-1)+0.25M_{CCbbaa}(i-$
$1)F_{CcbbAa}(i-1)+0.5M_{CCbbaa}(i-1)F_{CcbbAA}(i-1)+0.125M_{CCbbaa}(i-1)(1-\Phi)(1-\Phi)F_{CcBbAa}(i-1)+$
$0.25M_{CCbbaa}(i-1)(1-\Phi)\Phi F_{CcBbAa}(i-1)+0.25M_{CCbbaa}(i-1)(1-\Phi)F_{CcBbAA}(i-1)+0.5M_{CCbbaa}(i-$
$1)F_{CCbbAa}(i-1)+M_{CCbbaa}(i-1)F_{CCbbAA}(i-1)+0.25M_{CCbbaa}(i-1)(1-\Phi)(1-\Phi)F_{CCBbAa}(i-1)+$
$0.5M_{CCbbaa}(i-1)(1-\Phi)F_{CCBbAA}(i-1)+0.25M_{CCbbAa}(i-1)F_{Ccbbaa}(i-1)+0.25M_{CCbbAa}(i-1)F_{CcbbAa}(i-$
$1)+0.25M_{CCbbAa}(i-1)F_{CcbbAA}(i-1)+0.125M_{CCbbAa}(i-1)(1-\Phi)F_{CcBbaa}(i-1)+0.125M_{CCbbAa}(i-$
$1)(1-\Phi)(1-\Phi)F_{CcBbAa}(i-1)+0.125M_{CCbbAa}(i-1)(1-\Phi)\Phi F_{CcBbAa}(i-1)+0.125M_{CCbbAa}(i-1)(1-$
$\Phi)F_{CcBbAA}(i-1)+0.5M_{CCbbAa}(i-1)F_{CCbbaa}(i-1)+0.5M_{CCbbAa}(i-1)F_{CCbbAa}(i-1)+0.5M_{CCbbAa}(i-$
$1)F_{CCbbAA}(i-1)+0.25M_{CCbbAa}(i-1)(1-\Phi)F_{CCBbaa}(i-1)+0.25M_{CCbbAa}(i-1)(1-\Phi)(1-\Phi)F_{CCBbAa}(i-$
$1)+0.25M_{CCbbAa}(i-1)(1-\Phi)F_{CCBbAA}(i-1)+0.5M_{CCbbAA}(i-1)F_{Ccbbaa}(i-1)+0.25M_{CCbbAA}(i-$
$1)F_{CcbbAa}(i-1)+0.25M_{CCbbAA}(i-1)(1-\Phi)F_{CcBbaa}(i-1)+0.125M_{CCbbAA}(i-1)(1-\Phi)(1-\Phi)F_{CcBbAa}(i-$
$1)+M_{CCbbAA}(i-1)F_{CCbbaa}(i-1)+0.5M_{CCbbAA}(i-1)F_{CCbbAa}(i-1)+0.5M_{CCbbAA}(i-1)(1-\Phi)F_{CCBbaa}(i-$
$1)+0.25M_{CCbbAA}(i-1)(1-\Phi)(1-\Phi)F_{CCBbAa}(i-1)+0.125(1-\Phi)M_{CCBbaa}(i-1)F_{CcbbAa}(i-1)+$
$0.25(1-\Phi)M_{CCBbaa}(i-1)F_{CcbbAA}(i-1)+0.0625(1-\Phi)M_{CCBbaa}(i-1)(1-\Phi)(1-\Phi)F_{CcBbAa}(i-1)+$
$0.125(1-\Phi)M_{CCBbaa}(i-1)(1-\Phi)\Phi F_{CcBbAa}(i-1)+0.125(1-\Phi)M_{CCBbaa}(i-1)(1-\Phi)F_{CcBbAA}(i-$
$1)+0.25(1-\Phi)M_{CCBbaa}(i-1)F_{CCbbAa}(i-1)+0.5(1-\Phi)M_{CCBbaa}(i-1)F_{CCbbAA}(i-1)+0.125(1-$
$\Phi)M_{CCBbaa}(i-1)(1-\Phi)(1-\Phi)F_{CCBbAa}(i-1)+0.25(1-\Phi)M_{CCBbaa}(i-1)(1-\Phi)F_{CCBbAA}(i-1)+$
$0.125(1-\Phi)(1-\Phi)M_{CCBbAa}(i-1)F_{Ccbbaa}(i-1)+0.125(1-\Phi)(1-\Phi)M_{CCBbAa}(i-1)F_{CcbbAa}(i-1)+$
$0.125(1-\Phi)(1-\Phi)M_{CCBbAa}(i-1)F_{CcbbAA}(i-1)+0.0625(1-\Phi)(1-\Phi)M_{CCBbAa}(i-1)(1-\Phi)F_{CcBbaa}(i-$
$1)+0.0625(1-\Phi)(1-\Phi)M_{CCBbAa}(i-1)(1-\Phi)(1-\Phi)F_{CcBbAa}(i-1)+0.0625(1-\Phi)(1-\Phi)M_{CCBbAa}(i-$
$1)(1-\Phi)\Phi F_{CcBbAa}(i-1)+0.0625(1-\Phi)(1-\Phi)M_{CCBbAa}(i-1)(1-\Phi)F_{CcBbAA}(i-1)+0.25(1-\Phi)(1-$
$\Phi)M_{CCBbAa}(i-1)F_{CCbbaa}(i-1)+0.25(1-\Phi)(1-\Phi)M_{CCBbAa}(i-1)F_{CCbbAa}(i-1)+0.25(1-\Phi)(1-$
$\Phi)M_{CCBbAa}(i-1)F_{CCbbAA}(i-1)+0.125(1-\Phi)(1-\Phi)M_{CCBbAa}(i-1)(1-\Phi)F_{CCBbaa}(i-1)+0.125(1-\Phi)(1-$
$\Phi)M_{CCBbAa}(i-1)(1-\Phi)(1-\Phi)F_{CCBbAa}(i-1)+0.125(1-\Phi)(1-\Phi)M_{CCBbAa}(i-1)(1-\Phi)F_{CCBbAA}(i-$
$1)+0.25(1-\Phi)\Phi M_{CCBbAa}(i-1)F_{Ccbbaa}(i-1)+0.125(1-\Phi)\Phi M_{CCBbAa}(i-1)F_{CcbbAa}(i-1)+0.125(1-$
$\Phi)\Phi M_{CCBbAa}(i-1)(1-\Phi)F_{CcBbaa}(i-1)+0.0625(1-\Phi)\Phi M_{CCBbAa}(i-1)(1-\Phi)(1-\Phi)F_{CcBbAa}(i-$
$1)+0.5(1-\Phi)\Phi M_{CCBbAa}(i-1)F_{CCbbaa}(i-1)+0.25(1-\Phi)\Phi M_{CCBbAa}(i-1)F_{CCbbAa}(i-1)+0.25(1-$
$\Phi)\Phi M_{CCBbAa}(i-1)(1-\Phi)F_{CCBbaa}(i-1)+0.125(1-\Phi)\Phi M_{CCBbAa}(i-1)(1-\Phi)(1-\Phi)F_{CCBbAa}(i-$
$1)+0.25(1-\Phi)M_{CCBbAA}(i-1)F_{Ccbbaa}(i-1)+0.125(1-\Phi)M_{CCBbAA}(i-1)F_{CcbbAa}(i-1)+0.125(1-$
$\Phi)M_{CCBbAA}(i-1)(1-\Phi)F_{CcBbaa}(i-1)+0.0625(1-\Phi)M_{CCBbAA}(i-1)(1-\Phi)(1-\Phi)F_{CcBbAa}(i-$
$1)+0.5(1-\Phi)M_{CCBbAA}(i-1)F_{CCbbaa}(i-1)+0.25(1-\Phi)M_{CCBbAA}(i-1)F_{CCbbAa}(i-1)+0.25(1-$
$\Phi)M_{CCBbAA}(i-1)(1-\Phi)F_{CCBbaa}(i-1)+0.125(1-\Phi)M_{CCBbAA}(i-1)(1-\Phi)(1-\Phi)F_{CCBbAa}(i-1)],$

$\mathrm{M}^{e}_{CCbbAA}=\left(\frac{\bar{\Omega}_{CCbbAA}}{2}\right)[0.0625M_{CcbbAa}(i-1)F_{CcbbAa}(i-1)+0.125M_{CcbbAa}(i-1)F_{CcbbAA}(i-1)+$
$0.03125M_{CcbbAa}(i-1)(1-\Phi)(1-\Phi)F_{CcBbAa}(i-1)+0.0625M_{CcbbAa}(i-1)(1-\Phi)\Phi F_{CcBbAa}(i-1)+$

$$
0.0625M_{CcbbAa}(i-1)(1-\Phi)F_{CcBbAA}(i-1)+0.125M_{CcbbAa}(i-1)F_{CCbbAa}(i-1)+0.25M_{CcbbAa}(i-1)F_{CCbbAA}(i-1)+0.0625M_{CcbbAa}(i-1)(1-\Phi)(1-\Phi)F_{CCBbAa}(i-1)+0.125M_{CcbbAa}(i-1)(1-\Phi)F_{CCBbAA}(i-1)+0.125M_{CcbbAA}(i-1)F_{CcbbAa}(i-1)+0.25M_{CcbbAA}(i-1)F_{CcbbAA}(i-1)+0.0625M_{CcbbAA}(i-1)(1-\Phi)(1-\Phi)F_{CcBbAa}(i-1)+0.125M_{CcbbAA}(i-1)(1-\Phi)\Phi F_{CcBbAa}(i-1)+0.125M_{CcbbAA}(i-1)(1-\Phi)F_{CcBbAA}(i-1)+0.25M_{CcbbAA}(i-1)F_{CCbbAa}(i-1)+0.5M_{CcbbAA}(i-1)F_{CCbbAA}(i-1)+0.125M_{CcbbAA}(i-1)(1-\Phi)(1-\Phi)F_{CCBbAa}(i-1)+0.25M_{CcbbAA}(i-1)(1-\Phi)F_{CCBbAA}(i-1)+0.03125(1-\Phi)(1-\Phi)M_{CcBbAa}(i-1)F_{CcbbAa}(i-1)+0.0625(1-\Phi)(1-\Phi)M_{CcBbAa}(i-1)F_{CcbbAA}(i-1)+0.015625(1-\Phi)(1-\Phi)M_{CcBbAa}(i-1)(1-\Phi)(1-\Phi)F_{CcBbAa}(i-1)+0.03125(1-\Phi)(1-\Phi)M_{CcBbAa}(i-1)(1-\Phi)\Phi F_{CcBbAa}(i-1)+0.03125(1-\Phi)(1-\Phi)M_{CcBbAa}(i-1)(1-\Phi)F_{CcBbAA}(i-1)+0.0625(1-\Phi)(1-\Phi)M_{CcBbAa}(i-1)F_{CCbbAa}(i-1)+0.125(1-\Phi)(1-\Phi)M_{CcBbAa}(i-1)F_{CCbbAA}(i-1)+0.03125(1-\Phi)(1-\Phi)M_{CcBbAa}(i-1)(1-\Phi)(1-\Phi)F_{CCBbAa}(i-1)+0.0625(1-\Phi)(1-\Phi)M_{CcBbAa}(i-1)(1-\Phi)F_{CCBbAA}(i-1)+0.0625(1-\Phi)\Phi M_{CcBbAa}(i-1)F_{CcbbAa}(i-1)+0.125(1-\Phi)\Phi M_{CcBbAa}(i-1)F_{CcbbAA}(i-1)+0.03125(1-\Phi)\Phi M_{CcBbAa}(i-1)(1-\Phi)(1-\Phi)F_{CcBbAa}(i-1)+0.0625(1-\Phi)\Phi M_{CcBbAa}(i-1)(1-\Phi)\Phi F_{CcBbAa}(i-1)+0.0625(1-\Phi)\Phi M_{CcBbAa}(i-1)(1-\Phi)F_{CcBbAA}(i-1)+0.125(1-\Phi)\Phi M_{CcBbAa}(i-1)F_{CCbbAa}(i-1)+0.25(1-\Phi)\Phi M_{CcBbAa}(i-1)F_{CCbbAA}(i-1)+0.0625(1-\Phi)\Phi M_{CcBbAa}(i-1)(1-\Phi)(1-\Phi)F_{CCBbAa}(i-1)+0.125(1-\Phi)\Phi M_{CcBbAa}(i-1)(1-\Phi)F_{CCBbAA}(i-1)+0.0625(1-\Phi)M_{CcBbAA}(i-1)F_{CcbbAa}(i-1)+0.125(1-\Phi)M_{CcBbAA}(i-1)F_{CcbbAA}(i-1)+0.03125(1-\Phi)M_{CcBbAA}(i-1)(1-\Phi)(1-\Phi)F_{CcBbAa}(i-1)+0.0625(1-\Phi)M_{CcBbAA}(i-1)(1-\Phi)\Phi F_{CcBbAa}(i-1)+0.0625(1-\Phi)M_{CcBbAA}(i-1)(1-\Phi)F_{CcBbAA}(i-1)+0.125(1-\Phi)M_{CcBbAA}(i-1)F_{CCbbAa}(i-1)+0.25(1-\Phi)M_{CcBbAA}(i-1)F_{CCbbAA}(i-1)+0.0625(1-\Phi)M_{CcBbAA}(i-1)(1-\Phi)(1-\Phi)F_{CCBbAa}(i-1)+0.125(1-\Phi)M_{CcBbAA}(i-1)(1-\Phi)F_{CCBbAA}(i-1)+0.125M_{CCbbAa}(i-1)F_{CcbbAa}(i-1)+0.25M_{CCbbAa}(i-1)F_{CcbbAA}(i-1)+0.0625M_{CCbbAa}(i-1)(1-\Phi)(1-\Phi)F_{CcBbAa}(i-1)+0.125M_{CCbbAa}(i-1)(1-\Phi)\Phi F_{CcBbAa}(i-1)+0.125M_{CCbbAa}(i-1)(1-\Phi)F_{CcBbAA}(i-1)+0.25M_{CCbbAa}(i-1)F_{CCbbAa}(i-1)+0.5M_{CCbbAa}(i-1)F_{CCbbAA}(i-1)+0.125M_{CCbbAa}(i-1)(1-\Phi)(1-\Phi)F_{CCBbAa}(i-1)+0.25M_{CCbbAa}(i-1)(1-\Phi)F_{CCBbAA}(i-1)+0.25M_{CCbbAA}(i-1)F_{CcbbAa}(i-1)+0.5M_{CCbbAA}(i-1)F_{CcbbAA}(i-1)+0.125M_{CCbbAA}(i-1)(1-\Phi)(1-\Phi)F_{CcBbAa}(i-1)+0.25M_{CCbbAA}(i-1)(1-\Phi)\Phi F_{CcBbAa}(i-1)+0.25M_{CCbbAA}(i-1)(1-\Phi)F_{CcBbAA}(i-1)+0.5M_{CCbbAA}(i-1)F_{CCbbAa}(i-1)+M_{CCbbAA}(i-1)F_{CCbbAA}(i-1)+0.25M_{CCbbAA}(i-1)(1-\Phi)(1-\Phi)F_{CCBbAa}(i-1)+0.5M_{CCbbAA}(i-1)(1-\Phi)F_{CCBbAA}(i-1)+0.0625(1-\Phi)(1-\Phi)M_{CCBbAa}(i-1)F_{CcbbAa}(i-1)+0.125(1-\Phi)(1-\Phi)M_{CCBbAa}(i-1)F_{CcbbAA}(i-1)+0.03125(1-\Phi)(1-\Phi)M_{CCBbAa}(i-1)(1-\Phi)(1-\Phi)F_{CcBbAa}(i-1)+0.0625(1-\Phi)(1-\Phi)M_{CCBbAa}(i-1)(1-\Phi)\Phi F_{CcBbAa}(i-1)+0.0625(1-\Phi)(1-\Phi)M_{CCBbAa}(i-1)(1-\Phi)F_{CcBbAA}(i-1)+0.125(1-\Phi)(1-\Phi)M_{CCBbAa}(i-1)F_{CCbbAa}(i-1)+0.25(1-\Phi)(1-\Phi)M_{CCBbAa}(i-1)F_{CCbbAA}(i-1)+0.0625(1-\Phi)(1-\Phi)M_{CCBbAa}(i-1)(1-\Phi)(1-\Phi)F_{CCBbAa}(i-1)+0.125(1-\Phi)(1-\Phi)M_{CCBbAa}(i-1)(1-\Phi)F_{CCBbAA}(i-1)+0.125(1-\Phi)\Phi M_{CCBbAa}(i-1)F_{CcbbAa}(i-1)+0.25(1-\Phi)\Phi M_{CCBbAa}(i-1)F_{CcbbAA}(i-1)+0.0625(1-\Phi)\Phi M_{CCBbAa}(i-1)(1-\Phi)(1-\Phi)F_{CcBbAa}(i-1)+0.125(1-\Phi)\Phi M_{CCBbAa}(i-1)(1-\Phi)\Phi F_{CcBbAa}(i-1)+0.125(1-\Phi)\Phi M_{CCBbAa}(i-1)(1-\Phi)F_{CcBbAA}(i-1)+0.25(1-\Phi)\Phi M_{CCBbAa}(i-1)F_{CCbbAa}(i-1)+0.5(1-\Phi)\Phi M_{CCBbAa}(i-1)F_{CCbbAA}(i-1)+0.125(1-\Phi)\Phi M_{CCBbAa}(i-1)(1-\Phi)(1-\Phi)F_{CCBbAa}(i-1)+0.25(1-\Phi)\Phi M_{CCBbAa}(i-1)(1-\Phi)F_{CCBbAA}(i-1)+0.125(1-\Phi)M_{CCBbAA}(i-1)F_{CcbbAa}(i-1)+0.25(1-\Phi)M_{CCBbAA}(i-1)F_{CcbbAA}(i-1)+0.0625(1-\Phi)M_{CCBbAA}(i-1)(1-\Phi)(1-\Phi)F_{CcBbAa}(i-1)+0.125(1-\Phi)M_{CCBbAA}(i-1)(1-\Phi)\Phi F_{CcBbAa}(i-1)+0.125(1-\Phi)M_{CCBbAA}(i-1)(1-\Phi)F_{CcBbAA}(i-1)+0.25(1-\Phi)M_{CCBbAA}(i-1)F_{CCbbAa}(i-1)+0.5(1-\Phi)M_{CCBbAA}(i-1)F_{CCbbAA}(i-1)+0.125(1-\Phi)M_{CCBbAA}(i-1)(1-\Phi)(1-\Phi)F_{CCBbAa}(i-1)+0.25(1-\Phi)M_{CCBbAA}(i-1)(1-\Phi)F_{CCBbAA}(i-1)],
$$

$$
\mathrm{M}^{e}_{CCBbaa}=\left(\frac{\bar{\Omega}_{CCBbaa}}{2}\right)[0.125M_{Ccbbaa}(i-1)(1-\Phi)F_{CcBbaa}(i-1)+0.25M_{Ccbbaa}(i-1)\Phi F_{CcBbaa}(i-1)+0.0625M_{Ccbbaa}(i-1)(1-\Phi)(1-\Phi)F_{CcBbAa}(i-1)+0.125M_{Ccbbaa}(i-1)\Phi(1-\Phi)F_{CcBbAa}(i-1)+0.25M_{Ccbbaa}(i-1)F_{CcBBaa}(i-1)+0.125M_{Ccbbaa}(i-1)(1-\Phi)F_{CcBBAa}(i-1)+0.25M_{Ccbbaa}(i-1)(1-\Phi)F_{CCBbaa}(i-1)+0.5M_{Ccbbaa}(i-1)\Phi F_{CCBbaa}(i-1)+0.125M_{Ccbbaa}(i-1)(1-\Phi)(1-\Phi)F_{CCBbAa}(i-1)+0.25M_{Ccbbaa}(i-1)\Phi(1-\Phi)F_{CCBbAa}(i-1)+0.25M_{Ccbbaa}(i-1)(1-\Phi)\Phi F_{CCBbAa}(i-1)+0.5M_{Ccbbaa}(i-1)F_{CCBBaa}(i-1)+0.25M_{Ccbbaa}(i-1)(1-\Phi)F_{CCBBAa}(i-1)+0.0625M_{CcbbAa}(i-1)(1-\Phi)F_{CcBbaa}(i-1)+0.125M_{CcbbAa}(i-1)\Phi F_{CcBbaa}(i-1)+0.03125M_{CcbbAa}(i-1)(1-\Phi)(1-\Phi)F_{CcBbAa}(i-1)+0.0625M_{CcbbAa}(i-1)\Phi(1-\Phi)F_{CcBbAa}(i-1)+0.125M_{CcbbAa}(i-1)F_{CcBBaa}(i-1)+0.0625M_{CcbbAa}(i-1)(1-\Phi)F_{CcBBAa}(i-1)+0.125M_{CcbbAa}(i-1)(1-\Phi)F_{CCBbaa}(i-1)+0.25M_{CcbbAa}(i-1)\Phi F_{CCBbaa}(i-1)+0.0625M_{CcbbAa}(i-1)(1-\Phi)(1-\Phi)F_{CCBbAa}(i-1)+0.125M_{CcbbAa}(i-1)\Phi(1-\Phi)F_{CCBbAa}(i-1)+0.125M_{CcbbAa}(i-1)(1-\Phi)\Phi F_{CCBbAa}(i-1)+0.25M_{CcbbAa}(i-1)F_{CCBBaa}(i-1)+0.125M_{CcbbAa}(i-1)(1-\Phi)F_{CCBBAa}(i-1)+0.125(1-\Phi)M_{CcBbaa}(i-1)F_{Ccbbaa}(i-1)+0.0625(1-\Phi)M_{CcBbaa}(i-1)F_{CcbbAa}(i-1)+0.125(1-\Phi)M_{CcBbaa}(i-1)(1-\Phi)F_{CcBbaa}(i-1)+0.125(1-\Phi)M_{CcBbaa}(i-1)\Phi F_{CcBbaa}(i-1)+0.0625(1-\Phi)M_{CcBbaa}(i-1)(1-\Phi)(1-\Phi)F_{CcBbAa}(i-1)+0.0625(1-\Phi)M_{CcBbaa}(i-1)\Phi(1-\Phi)F_{CcBbAa}(i-1)+0.125(1-\Phi)M_{CcBbaa}(i-1)F_{CcBBaa}(i-1)+0.0625(1-\Phi)M_{CcBbaa}(i-1)(1-\Phi)F_{CcBBAa}(i-1)+0.25(1-
$$

$$
\Phi)M_{CcBbaa}(i-1)F_{CCbbaa}(i-1)+0.125(1-\Phi)M_{CcBbaa}(i-1)F_{CCbbAa}(i-1)+0.25(1-\Phi)M_{CcBbaa}(i-1)(1-
\Phi)F_{CCBbaa}(i-1)+0.25(1-\Phi)M_{CcBbaa}(i-1)\Phi F_{CCBbaa}(i-1)+0.125(1-\Phi)M_{CcBbaa}(i-1)(1-\Phi)(1-
\Phi)F_{CCBbAa}(i-1)+0.125(1-\Phi)M_{CcBbaa}(i-1)\Phi(1-\Phi)F_{CCBbAa}(i-1)+0.125(1-\Phi)M_{CcBbaa}(i-
1)(1-\Phi)\Phi F_{CCBbAa}(i-1)+0.25(1-\Phi)M_{CcBbaa}(i-1)F_{CCBBaa}(i-1)+0.125(1-\Phi)M_{CcBbaa}(i-
1)(1-\Phi)F_{CCBBAa}(i-1)+0.25\Phi M_{CcBbaa}(i-1)F_{Ccbbaa}(i-1)+0.125\Phi M_{CcBbaa}(i-1)F_{CcbbAa}(i-1)+
0.125\Phi M_{CcBbaa}(i-1)(1-\Phi)F_{CcBbaa}(i-1)+0.0625\Phi M_{CcBbaa}(i-1)(1-\Phi)(1-\Phi)F_{CcBbAa}(i-1)+
0.5\Phi M_{CcBbaa}(i-1)F_{CCbbaa}(i-1)+0.25\Phi M_{CcBbaa}(i-1)F_{CCbbAa}(i-1)+0.25\Phi M_{CcBbaa}(i-1)(1-
\Phi)F_{CCBbaa}(i-1)+0.125\Phi M_{CcBbaa}(i-1)(1-\Phi)(1-\Phi)F_{CCBbAa}(i-1)+0.0625(1-\Phi)(1-\Phi)M_{CcBbAa}(i-
1)F_{Ccbbaa}(i-1)+0.03125(1-\Phi)(1-\Phi)M_{CcBbAa}(i-1)F_{CcbbAa}(i-1)+0.0625(1-\Phi)(1-\Phi)M_{CcBbAa}(i-1)(1-
\Phi)F_{CcBbaa}(i-1)+0.0625(1-\Phi)(1-\Phi)M_{CcBbAa}(i-1)\Phi F_{CcBbaa}(i-1)+0.03125(1-\Phi)(1-\Phi)M_{CcBbAa}(i-
1)(1-\Phi)(1-\Phi)F_{CcBbAa}(i-1)+0.03125(1-\Phi)(1-\Phi)M_{CcBbAa}(i-1)\Phi(1-\Phi)F_{CcBbAa}(i-1)+0.0625(1-
\Phi)(1-\Phi)M_{CcBbAa}(i-1)F_{CcBBaa}(i-1)+0.03125(1-\Phi)(1-\Phi)M_{CcBbAa}(i-1)(1-\Phi)F_{CcBBAa}(i-1)+
0.125(1-\Phi)(1-\Phi)M_{CcBbAa}(i-1)F_{CCbbaa}(i-1)+0.0625(1-\Phi)(1-\Phi)M_{CcBbAa}(i-1)F_{CCbbAa}(i-1)+
0.125(1-\Phi)(1-\Phi)M_{CcBbAa}(i-1)(1-\Phi)F_{CCBbaa}(i-1)+0.125(1-\Phi)(1-\Phi)M_{CcBbAa}(i-1)\Phi F_{CCBbaa}(i-
1)+0.0625(1-\Phi)(1-\Phi)M_{CcBbAa}(i-1)(1-\Phi)(1-\Phi)F_{CCBbAa}(i-1)+0.0625(1-\Phi)(1-\Phi)M_{CcBbAa}(i-
1)\Phi(1-\Phi)F_{CCBbAa}(i-1)+0.0625(1-\Phi)(1-\Phi)M_{CcBbAa}(i-1)(1-\Phi)\Phi F_{CCBbAa}(i-1)+0.125(1-\Phi)(1-
\Phi)M_{CcBbAa}(i-1)F_{CCBBaa}(i-1)+0.0625(1-\Phi)(1-\Phi)M_{CcBbAa}(i-1)(1-\Phi)F_{CCBBAa}(i-1)+0.125\Phi(1-
\Phi)M_{CcBbAa}(i-1)F_{Ccbbaa}(i-1)+0.0625\Phi(1-\Phi)M_{CcBbAa}(i-1)F_{CcbbAa}(i-1)+0.0625\Phi(1-\Phi)M_{CcBbAa}(i-
1)(1-\Phi)F_{CcBbaa}(i-1)+0.03125\Phi(1-\Phi)M_{CcBbAa}(i-1)(1-\Phi)(1-\Phi)F_{CcBbAa}(i-1)+0.25\Phi(1-
\Phi)M_{CcBbAa}(i-1)F_{CCbbaa}(i-1)+0.125\Phi(1-\Phi)M_{CcBbAa}(i-1)F_{CCbbAa}(i-1)+0.125\Phi(1-\Phi)M_{CcBbAa}(i-
1)(1-\Phi)F_{CCBbaa}(i-1)+0.0625\Phi(1-\Phi)M_{CcBbAa}(i-1)(1-\Phi)(1-\Phi)F_{CCBbAa}(i-1)+0.25M_{CcBBaa}(i-
1)F_{Ccbbaa}(i-1)+0.125M_{CcBBaa}(i-1)F_{CcbbAa}(i-1)+0.125M_{CcBBaa}(i-1)(1-\Phi)F_{CcBbaa}(i-1)+
0.0625M_{CcBBaa}(i-1)(1-\Phi)(1-\Phi)F_{CcBbAa}(i-1)+0.5M_{CcBBaa}(i-1)F_{CCbbaa}(i-1)+0.25M_{CcBBaa}(i-
1)F_{CCbbAa}(i-1)+0.25M_{CcBBaa}(i-1)(1-\Phi)F_{CCBbaa}(i-1)+0.125M_{CcBBaa}(i-1)(1-\Phi)(1-\Phi)F_{CCBbAa}(i-
1)+0.125(1-\Phi)M_{CcBBAa}(i-1)F_{Ccbbaa}(i-1)+0.0625(1-\Phi)M_{CcBBAa}(i-1)F_{CcbbAa}(i-1)+0.0625(1-
\Phi)M_{CcBBAa}(i-1)(1-\Phi)F_{CcBbaa}(i-1)+0.03125(1-\Phi)M_{CcBBAa}(i-1)(1-\Phi)(1-\Phi)F_{CcBbAa}(i-
1)+0.25(1-\Phi)M_{CcBBAa}(i-1)F_{CCbbaa}(i-1)+0.125(1-\Phi)M_{CcBBAa}(i-1)F_{CCbbAa}(i-1)+0.125(1-
\Phi)M_{CcBBAa}(i-1)(1-\Phi)F_{CCBbaa}(i-1)+0.0625(1-\Phi)M_{CcBBAa}(i-1)(1-\Phi)(1-\Phi)F_{CCBbAa}(i-1)+
0.25M_{CCbbaa}(i-1)(1-\Phi)F_{CcBbaa}(i-1)+0.5M_{CCbbaa}(i-1)\Phi F_{CcBbaa}(i-1)+0.125M_{CCbbaa}(i-1)(1-
\Phi)(1-\Phi)F_{CcBbAa}(i-1)+0.25M_{CCbbaa}(i-1)\Phi(1-\Phi)F_{CcBbAa}(i-1)+0.5M_{CCbbaa}(i-1)F_{CcBBaa}(i-
1)+0.25M_{CCbbaa}(i-1)(1-\Phi)F_{CcBBAa}(i-1)+0.5M_{CCbbaa}(i-1)(1-\Phi)F_{CCBbaa}(i-1)+M_{CCbbaa}(i-
1)\Phi F_{CCBbaa}(i-1)+0.25M_{CCbbaa}(i-1)(1-\Phi)(1-\Phi)F_{CCBbAa}(i-1)+0.5M_{CCbbaa}(i-1)\Phi(1-\Phi)F_{CCBbAa}(i-
1)+0.5M_{CCbbaa}(i-1)(1-\Phi)\Phi F_{CCBbAa}(i-1)+M_{CCbbaa}(i-1)F_{CCBBaa}(i-1)+0.5M_{CCbbaa}(i-1)(1-
\Phi)F_{CCBBAa}(i-1)+0.125M_{CCbbAa}(i-1)(1-\Phi)F_{CcBbaa}(i-1)+0.25M_{CCbbAa}(i-1)\Phi F_{CcBbaa}(i-1)+
0.0625M_{CCbbAa}(i-1)(1-\Phi)(1-\Phi)F_{CcBbAa}(i-1)+0.125M_{CCbbAa}(i-1)\Phi(1-\Phi)F_{CcBbAa}(i-1)+
0.25M_{CCbbAa}(i-1)F_{CcBBaa}(i-1)+0.125M_{CCbbAa}(i-1)(1-\Phi)F_{CcBBAa}(i-1)+0.25M_{CCbbAa}(i-1)(1-
\Phi)F_{CCBbaa}(i-1)+0.5M_{CCbbAa}(i-1)\Phi F_{CCBbaa}(i-1)+0.125M_{CCbbAa}(i-1)(1-\Phi)(1-\Phi)F_{CCBbAa}(i-1)+
0.25M_{CCbbAa}(i-1)\Phi(1-\Phi)F_{CCBbAa}(i-1)+0.25M_{CCbbAa}(i-1)(1-\Phi)\Phi F_{CCBbAa}(i-1)+0.5M_{CCbbAa}(i-
1)F_{CCBBaa}(i-1)+0.25M_{CCbbAa}(i-1)(1-\Phi)F_{CCBBAa}(i-1)+0.25(1-\Phi)M_{CCBbaa}(i-1)F_{Ccbbaa}(i-
1)+0.125(1-\Phi)M_{CCBbaa}(i-1)F_{CcbbAa}(i-1)+0.25(1-\Phi)M_{CCBbaa}(i-1)(1-\Phi)F_{CcBbaa}(i-1)+
0.25(1-\Phi)M_{CCBbaa}(i-1)\Phi F_{CcBbaa}(i-1)+0.125(1-\Phi)M_{CCBbaa}(i-1)(1-\Phi)(1-\Phi)F_{CcBbAa}(i-
1)+0.125(1-\Phi)M_{CCBbaa}(i-1)\Phi(1-\Phi)F_{CcBbAa}(i-1)+0.25(1-\Phi)M_{CCBbaa}(i-1)F_{CcBBaa}(i-1)+
0.125(1-\Phi)M_{CCBbaa}(i-1)(1-\Phi)F_{CcBBAa}(i-1)+0.5(1-\Phi)M_{CCBbaa}(i-1)F_{CCbbaa}(i-1)+0.25(1-
\Phi)M_{CCBbaa}(i-1)F_{CCbbAa}(i-1)+0.5(1-\Phi)M_{CCBbaa}(i-1)(1-\Phi)F_{CCBbaa}(i-1)+0.5(1-\Phi)M_{CCBbaa}(i-
1)\Phi F_{CCBbaa}(i-1)+0.25(1-\Phi)M_{CCBbaa}(i-1)(1-\Phi)(1-\Phi)F_{CCBbAa}(i-1)+0.25(1-\Phi)M_{CCBbaa}(i-
1)\Phi(1-\Phi)F_{CCBbAa}(i-1)+0.25(1-\Phi)M_{CCBbaa}(i-1)(1-\Phi)\Phi F_{CCBbAa}(i-1)+0.5(1-\Phi)M_{CCBbaa}(i-
1)F_{CCBBaa}(i-1)+0.25(1-\Phi)M_{CCBbaa}(i-1)(1-\Phi)F_{CCBBAa}(i-1)+0.5\Phi M_{CCBbaa}(i-1)F_{Ccbbaa}(i-
1)+0.25\Phi M_{CCBbaa}(i-1)F_{CcbbAa}(i-1)+0.25\Phi M_{CCBbaa}(i-1)(1-\Phi)F_{CcBbaa}(i-1)+0.125\Phi M_{CCBbaa}(i-
1)(1-\Phi)(1-\Phi)F_{CcBbAa}(i-1)+\Phi M_{CCBbaa}(i-1)F_{CCbbaa}(i-1)+0.5\Phi M_{CCBbaa}(i-1)F_{CCbbAa}(i-1)+
0.5\Phi M_{CCBbaa}(i-1)(1-\Phi)F_{CCBbaa}(i-1)+0.25\Phi M_{CCBbaa}(i-1)(1-\Phi)(1-\Phi)F_{CCBbAa}(i-1)+0.125(1-
\Phi)(1-\Phi)M_{CCBbAa}(i-1)F_{Ccbbaa}(i-1)+0.0625(1-\Phi)(1-\Phi)M_{CCBbAa}(i-1)F_{CcbbAa}(i-1)+0.125(1-
\Phi)(1-\Phi)M_{CCBbAa}(i-1)(1-\Phi)F_{CcBbaa}(i-1)+0.125(1-\Phi)(1-\Phi)M_{CCBbAa}(i-1)\Phi F_{CcBbaa}(i-1)+
0.0625(1-\Phi)(1-\Phi)M_{CCBbAa}(i-1)(1-\Phi)(1-\Phi)F_{CcBbAa}(i-1)+0.0625(1-\Phi)(1-\Phi)M_{CCBbAa}(i-1)\Phi(1-
\Phi)F_{CcBbAa}(i-1)+0.125(1-\Phi)(1-\Phi)M_{CCBbAa}(i-1)F_{CcBBaa}(i-1)+0.0625(1-\Phi)(1-\Phi)M_{CCBbAa}(i-
1)(1-\Phi)F_{CcBBAa}(i-1)+0.25(1-\Phi)(1-\Phi)M_{CCBbAa}(i-1)F_{CCbbaa}(i-1)+0.125(1-\Phi)(1-\Phi)M_{CCBbAa}(i-
1)F_{CCbbAa}(i-1)+0.25(1-\Phi)(1-\Phi)M_{CCBbAa}(i-1)(1-\Phi)F_{CCBbaa}(i-1)+0.25(1-\Phi)(1-\Phi)M_{CCBbAa}(i-
1)\Phi F_{CCBbaa}(i-1)+0.125(1-\Phi)(1-\Phi)M_{CCBbAa}(i-1)(1-\Phi)(1-\Phi)F_{CCBbAa}(i-1)+0.125(1-\Phi)(1-
$$

$\Phi)M_{CCBbAa}(i-1)\Phi(1-\Phi)F_{CCBbAa}(i-1)+0.125(1-\Phi)(1-\Phi)M_{CCBbAa}(i-1)(1-\Phi)\Phi F_{CCBbAa}(i-1)+$
$0.25(1-\Phi)(1-\Phi)M_{CCBbAa}(i-1)F_{CCBBaa}(i-1)+0.125(1-\Phi)(1-\Phi)M_{CCBbAa}(i-1)(1-\Phi)F_{CCBBAa}(i-$
$1)+0.25\Phi(1-\Phi)M_{CCBbAa}(i-1)F_{Ccbbaa}(i-1)+0.125\Phi(1-\Phi)M_{CCBbAa}(i-1)F_{CcbbAa}(i-1)+0.125\Phi(1-$
$\Phi)M_{CCBbAa}(i-1)(1-\Phi)F_{CcBbaa}(i-1)+0.0625\Phi(1-\Phi)M_{CCBbAa}(i-1)(1-\Phi)(1-\Phi)F_{CcBbAa}(i-1)+$
$0.5\Phi(1-\Phi)M_{CCBbAa}(i-1)F_{CCbbaa}(i-1)+0.25\Phi(1-\Phi)M_{CCBbAa}(i-1)F_{CCbbAa}(i-1)+0.25\Phi(1-$
$\Phi)M_{CCBbAa}(i-1)(1-\Phi)F_{CCBbaa}(i-1)+0.125\Phi(1-\Phi)M_{CCBbAa}(i-1)(1-\Phi)(1-\Phi)F_{CCBbAa}(i-$
$1)+0.5M_{CCBBaa}(i-1)F_{Ccbbaa}(i-1)+0.25M_{CCBBaa}(i-1)F_{CcbbAa}(i-1)+0.25M_{CCBBaa}(i-1)(1-$
$\Phi)F_{CcBbaa}(i-1)+0.125M_{CCBBaa}(i-1)(1-\Phi)(1-\Phi)F_{CcBbAa}(i-1)+M_{CCBBaa}(i-1)F_{CCbbaa}(i-1)+$
$0.5M_{CCBBaa}(i-1)F_{CCbbAa}(i-1)+0.5M_{CCBBaa}(i-1)(1-\Phi)F_{CCBbaa}(i-1)+0.25M_{CCBBaa}(i-1)(1-\Phi)(1-$
$\Phi)F_{CCBbAa}(i-1)+0.25(1-\Phi)M_{CCBBAa}(i-1)F_{Ccbbaa}(i-1)+0.125(1-\Phi)M_{CCBBAa}(i-1)F_{CcbbAa}(i-1)+$
$0.125(1-\Phi)M_{CCBBAa}(i-1)(1-\Phi)F_{CcBbaa}(i-1)+0.0625(1-\Phi)M_{CCBBAa}(i-1)(1-\Phi)(1-\Phi)F_{CcBbAa}(i-$
$1)+0.5(1-\Phi)M_{CCBBAa}(i-1)F_{CCbbaa}(i-1)+0.25(1-\Phi)M_{CCBBAa}(i-1)F_{CCbbAa}(i-1)+0.25(1-$
$\Phi)M_{CCBBAa}(i-1)(1-\Phi)F_{CCBbaa}(i-1)+0.125(1-\Phi)M_{CCBBAa}(i-1)(1-\Phi)(1-\Phi)F_{CCBbAa}(i-1)],$

$\mathrm{M}^e_{CCBbAa}=\left(\frac{\bar{\Omega}_{CCBbAa}}{2}\right)[0.0625M_{Ccbbaa}(i-1)(1-\Phi)(1-\Phi)F_{CcBbAa}(i-1)+0.125M_{Ccbbaa}(i-1)\Phi(1-$
$\Phi)F_{CcBbAa}(i-1)+0.125M_{Ccbbaa}(i-1)(1-\Phi)\Phi F_{CcBbAa}(i-1)+0.25M_{Ccbbaa}(i-1)\Phi\Phi F_{CcBbAa}(i-$
$1)+0.125M_{Ccbbaa}(i-1)(1-\Phi)F_{CcBbAA}(i-1)+0.25M_{Ccbbaa}(i-1)\Phi F_{CcBbAA}(i-1)+0.125M_{Ccbbaa}(i-$
$1)(1-\Phi)F_{CcBBAa}(i-1)+0.25M_{Ccbbaa}(i-1)\Phi F_{CcBBAa}(i-1)+0.25M_{Ccbbaa}(i-1)F_{CcBBAA}(i-1)+$
$0.125M_{Ccbbaa}(i-1)(1-\Phi)(1-\Phi)F_{CCBbAa}(i-1)+0.25M_{Ccbbaa}(i-1)\Phi(1-\Phi)F_{CCBbAa}(i-1)+0.25M_{Ccbbaa}(i-$
$1)(1-\Phi)\Phi F_{CCBbAa}(i-1)+0.5M_{Ccbbaa}(i-1)\Phi\Phi F_{CCBbAa}(i-1)+0.25M_{Ccbbaa}(i-1)(1-\Phi)F_{CCBbAA}(i-$
$1)+0.5M_{Ccbbaa}(i-1)\Phi F_{CCBbAA}(i-1)+0.25M_{Ccbbaa}(i-1)(1-\Phi)F_{CCBBAa}(i-1)+0.5M_{Ccbbaa}(i-$
$1)\Phi F_{CCBBAa}(i-1)+0.5M_{Ccbbaa}(i-1)F_{CCBBAA}(i-1)+0.0625M_{CcbbAa}(i-1)(1-\Phi)F_{CcBbaa}(i-1)+$
$0.125M_{CcbbAa}(i-1)\Phi F_{CcBbaa}(i-1)+0.0625M_{CcbbAa}(i-1)(1-\Phi)(1-\Phi)F_{CcBbAa}(i-1)+0.125M_{CcbbAa}(i-$
$1)\Phi(1-\Phi)F_{CcBbAa}(i-1)+0.0625M_{CcbbAa}(i-1)(1-\Phi)\Phi F_{CcBbAa}(i-1)+0.125M_{CcbbAa}(i-1)\Phi\Phi F_{CcBbAa}(i-$
$1)+0.0625M_{CcbbAa}(i-1)(1-\Phi)F_{CcBbAA}(i-1)+0.125M_{CcbbAa}(i-1)\Phi F_{CcBbAA}(i-1)+0.125M_{CcbbAa}(i-$
$1)F_{CcBBaa}(i-1)+0.125M_{CcbbAa}(i-1)(1-\Phi)F_{CcBBAa}(i-1)+0.125M_{CcbbAa}(i-1)\Phi F_{CcBBAa}(i-1)+$
$0.125M_{CcbbAa}(i-1)F_{CcBBAA}(i-1)+0.125M_{CcbbAa}(i-1)(1-\Phi)F_{CCBbaa}(i-1)+0.25M_{CcbbAa}(i-$
$1)\Phi F_{CCBbaa}(i-1)+0.125M_{CcbbAa}(i-1)(1-\Phi)(1-\Phi)F_{CCBbAa}(i-1)+0.25M_{CcbbAa}(i-1)\Phi(1-$
$\Phi)F_{CCBbAa}(i-1)+0.25M_{CcbbAa}(i-1)(1-\Phi)\Phi F_{CCBbAa}(i-1)+0.25M_{CcbbAa}(i-1)\Phi\Phi F_{CCBbAa}(i-$
$1)+0.125M_{CcbbAa}(i-1)(1-\Phi)F_{CCBbAA}(i-1)+0.25M_{CcbbAa}(i-1)\Phi F_{CCBbAA}(i-1)+0.25M_{CcbbAa}(i-$
$1)F_{CCBBaa}(i-1)+0.25M_{CcbbAa}(i-1)(1-\Phi)F_{CCBBAa}(i-1)+0.25M_{CcbbAa}(i-1)\Phi F_{CCBBAa}(i-1)+$
$0.25M_{CcbbAa}(i-1)F_{CCBBAA}(i-1)+0.125M_{CcbbAA}(i-1)(1-\Phi)F_{CcBbaa}(i-1)+0.25M_{CcbbAA}(i-$
$1)\Phi F_{CcBbaa}(i-1)+0.0625M_{CcbbAA}(i-1)(1-\Phi)(1-\Phi)F_{CcBbAa}(i-1)+0.125M_{CcbbAA}(i-1)\Phi(1-$
$\Phi)F_{CcBbAa}(i-1)+0.25M_{CcbbAA}(i-1)F_{CcBBaa}(i-1)+0.125M_{CcbbAA}(i-1)(1-\Phi)F_{CcBBAa}(i-1)+$
$0.25M_{CcbbAA}(i-1)(1-\Phi)F_{CCBbaa}(i-1)+0.5M_{CcbbAA}(i-1)\Phi F_{CCBbaa}(i-1)+0.125M_{CcbbAA}(i-$
$1)(1-\Phi)(1-\Phi)F_{CCBbAa}(i-1)+0.25M_{CcbbAA}(i-1)\Phi(1-\Phi)F_{CCBbAa}(i-1)+0.25M_{CcbbAA}(i-1)(1-$
$\Phi)\Phi F_{CCBbAa}(i-1)+0.5M_{CcbbAA}(i-1)F_{CCBBaa}(i-1)+0.25M_{CcbbAA}(i-1)(1-\Phi)F_{CCBBAa}(i-1)+$
$0.0625(1-\Phi)M_{CcBbaa}(i-1)F_{CcbbAa}(i-1)+0.125(1-\Phi)M_{CcBbaa}(i-1)F_{CcbbAA}(i-1)+0.0625(1-$
$\Phi)M_{CcBbaa}(i-1)(1-\Phi)(1-\Phi)F_{CcBbAa}(i-1)+0.0625(1-\Phi)M_{CcBbaa}(i-1)\Phi(1-\Phi)F_{CcBbAa}(i-$
$1)+0.125(1-\Phi)M_{CcBbaa}(i-1)(1-\Phi)\Phi F_{CcBbAa}(i-1)+0.125(1-\Phi)M_{CcBbaa}(i-1)\Phi\Phi F_{CcBbAa}(i-$
$1)+0.125(1-\Phi)M_{CcBbaa}(i-1)(1-\Phi)F_{CcBbAA}(i-1)+0.125(1-\Phi)M_{CcBbaa}(i-1)\Phi F_{CcBbAA}(i-1)+$
$0.0625(1-\Phi)M_{CcBbaa}(i-1)(1-\Phi)F_{CcBBAa}(i-1)+0.125(1-\Phi)M_{CcBbaa}(i-1)\Phi F_{CcBBAa}(i-1)+$
$0.125(1-\Phi)M_{CcBbaa}(i-1)F_{CcBBAA}(i-1)+0.125(1-\Phi)M_{CcBbaa}(i-1)F_{CCbbAa}(i-1)+0.25(1-$
$\Phi)M_{CcBbaa}(i-1)F_{CCbbAA}(i-1)+0.125(1-\Phi)M_{CcBbaa}(i-1)(1-\Phi)(1-\Phi)F_{CCBbAa}(i-1)+0.125(1-$
$\Phi)M_{CcBbaa}(i-1)\Phi(1-\Phi)F_{CCBbAa}(i-1)+0.125(1-\Phi)M_{CcBbaa}(i-1)(1-\Phi)\Phi F_{CCBbAa}(i-1)+0.25(1-$
$\Phi)M_{CcBbaa}(i-1)\Phi\Phi F_{CCBbAa}(i-1)+0.25(1-\Phi)M_{CcBbaa}(i-1)(1-\Phi)F_{CCBbAA}(i-1)+0.25(1-$
$\Phi)M_{CcBbaa}(i-1)\Phi F_{CCBbAA}(i-1)+0.125(1-\Phi)M_{CcBbaa}(i-1)(1-\Phi)F_{CCBBAa}(i-1)+0.25(1-$
$\Phi)M_{CcBbaa}(i-1)\Phi F_{CCBBAa}(i-1)+0.25(1-\Phi)M_{CcBbaa}(i-1)F_{CCBBAA}(i-1)+0.125\Phi M_{CcBbaa}(i-$
$1)F_{CcbbAa}(i-1)+0.25\Phi M_{CcBbaa}(i-1)F_{CcbbAA}(i-1)+0.0625\Phi M_{CcBbaa}(i-1)(1-\Phi)(1-\Phi)F_{CcBbAa}(i-$
$1)+0.125\Phi M_{CcBbaa}(i-1)(1-\Phi)\Phi F_{CcBbAa}(i-1)+0.125\Phi M_{CcBbaa}(i-1)(1-\Phi)F_{CcBbAA}(i-1)+$
$0.25\Phi M_{CcBbaa}(i-1)F_{CCbbAa}(i-1)+0.5\Phi M_{CcBbaa}(i-1)F_{CCbbAA}(i-1)+0.125\Phi M_{CcBbaa}(i-1)(1-$
$\Phi)(1-\Phi)F_{CCBbAa}(i-1)+0.25\Phi M_{CcBbaa}(i-1)(1-\Phi)F_{CCBbAA}(i-1)+0.0625(1-\Phi)(1-\Phi)M_{CcBbAa}(i-$
$1)F_{Ccbbaa}(i-1)+0.0625(1-\Phi)(1-\Phi)M_{CcBbAa}(i-1)F_{CcbbAa}(i-1)+0.0625(1-\Phi)(1-\Phi)M_{CcBbAa}(i-$
$1)F_{CcbbAA}(i-1)+0.0625(1-\Phi)(1-\Phi)M_{CcBbAa}(i-1)(1-\Phi)F_{CcBbaa}(i-1)+0.0625(1-\Phi)(1-\Phi)M_{CcBbAa}(i-$
$1)\Phi F_{CcBbaa}(i-1)+0.0625(1-\Phi)(1-\Phi)M_{CcBbAa}(i-1)(1-\Phi)(1-\Phi)F_{CcBbAa}(i-1)+0.0625(1-\Phi)(1-$
$\Phi)M_{CcBbAa}(i-1)\Phi(1-\Phi)F_{CcBbAa}(i-1)+0.0625(1-\Phi)(1-\Phi)M_{CcBbAa}(i-1)(1-\Phi)\Phi F_{CcBbAa}(i-$
$1)+0.0625(1-\Phi)(1-\Phi)M_{CcBbAa}(i-1)\Phi\Phi F_{CcBbAa}(i-1)+0.0625(1-\Phi)(1-\Phi)M_{CcBbAa}(i-1)(1-$

$\Phi)F_{CcBbAA}(i-1)+0.0625(1-\Phi)(1-\Phi)M_{CcBbAa}(i-1)\Phi F_{CcBbAA}(i-1)+0.0625(1-\Phi)(1-\Phi)M_{CcBbAa}(i-$
$1)F_{CcBBaa}(i-1)+0.0625(1-\Phi)(1-\Phi)M_{CcBbAa}(i-1)(1-\Phi)F_{CcBBAa}(i-1)+0.0625(1-\Phi)(1-$
$\Phi)M_{CcBbAa}(i-1)\Phi F_{CcBBAa}(i-1)+0.0625(1-\Phi)(1-\Phi)M_{CcBbAa}(i-1)F_{CcBBAA}(i-1)+0.125(1-$
$\Phi)(1-\Phi)M_{CcBbAa}(i-1)F_{CCbbaa}(i-1)+0.125(1-\Phi)(1-\Phi)M_{CcBbAa}(i-1)F_{CCbbAa}(i-1)+0.125(1-\Phi)(1-$
$\Phi)M_{CcBbAa}(i-1)F_{CCbbAA}(i-1)+0.125(1-\Phi)(1-\Phi)M_{CcBbAa}(i-1)(1-\Phi)F_{CCBbaa}(i-1)+0.125(1-$
$\Phi)(1-\Phi)M_{CcBbAa}(i-1)\Phi F_{CCBbaa}(i-1)+0.125(1-\Phi)(1-\Phi)M_{CcBbAa}(i-1)(1-\Phi)(1-\Phi)F_{CCBbAa}(i-$
$1)+0.125(1-\Phi)(1-\Phi)M_{CcBbAa}(i-1)\Phi(1-\Phi)F_{CCBbAa}(i-1)+0.125(1-\Phi)(1-\Phi)M_{CcBbAa}(i-$
$1)(1-\Phi)\Phi F_{CCBbAa}(i-1)+0.125(1-\Phi)(1-\Phi)M_{CcBbAa}(i-1)\Phi\Phi F_{CCBbAa}(i-1)+0.125(1-\Phi)(1-$
$\Phi)M_{CcBbAa}(i-1)(1-\Phi)F_{CCBbAA}(i-1)+0.125(1-\Phi)(1-\Phi)M_{CcBbAa}(i-1)\Phi F_{CCBbAA}(i-1)+0.125(1-$
$\Phi)(1-\Phi)M_{CcBbAa}(i-1)F_{CCBBaa}(i-1)+0.125(1-\Phi)(1-\Phi)M_{CcBbAa}(i-1)(1-\Phi)F_{CCBBAa}(i-1)+$
$0.125(1-\Phi)(1-\Phi)M_{CcBbAa}(i-1)\Phi F_{CCBBAa}(i-1)+0.125(1-\Phi)(1-\Phi)M_{CcBbAa}(i-1)F_{CCBBAA}(i-$
$1)+0.125\Phi(1-\Phi)M_{CcBbAa}(i-1)F_{Ccbbaa}(i-1)+0.125\Phi(1-\Phi)M_{CcBbAa}(i-1)F_{CcbbAa}(i-1)+0.125\Phi(1-$
$\Phi)M_{CcBbAa}(i-1)F_{CcbbAA}(i-1)+0.0625\Phi(1-\Phi)M_{CcBbAa}(i-1)(1-\Phi)F_{CcBbaa}(i-1)+0.0625\Phi(1-$
$\Phi)M_{CcBbAa}(i-1)(1-\Phi)(1-\Phi)F_{CcBbAa}(i-1)+0.0625\Phi(1-\Phi)M_{CcBbAa}(i-1)(1-\Phi)\Phi F_{CcBbAa}(i-$
$1)+0.0625\Phi(1-\Phi)M_{CcBbAa}(i-1)(1-\Phi)F_{CcBbAA}(i-1)+0.25\Phi(1-\Phi)M_{CcBbAa}(i-1)F_{CCbbaa}(i-1)+$
$0.25\Phi(1-\Phi)M_{CcBbAa}(i-1)F_{CCbbAa}(i-1)+0.25\Phi(1-\Phi)M_{CcBbAa}(i-1)F_{CCbbAA}(i-1)+0.125\Phi(1-$
$\Phi)M_{CcBbAa}(i-1)(1-\Phi)F_{CCBbaa}(i-1)+0.125\Phi(1-\Phi)M_{CcBbAa}(i-1)(1-\Phi)(1-\Phi)F_{CCBbAa}(i-1)+$
$0.125\Phi(1-\Phi)M_{CcBbAa}(i-1)(1-\Phi)F_{CCBbAA}(i-1)+0.125(1-\Phi)\Phi M_{CcBbAa}(i-1)F_{Ccbbaa}(i-1)+$
$0.0625(1-\Phi)\Phi M_{CcBbAa}(i-1)F_{CcbbAa}(i-1)+0.125(1-\Phi)\Phi M_{CcBbAa}(i-1)(1-\Phi)F_{CcBbaa}(i-1)+$
$0.125(1-\Phi)\Phi M_{CcBbAa}(i-1)\Phi F_{CcBbaa}(i-1)+0.0625(1-\Phi)\Phi M_{CcBbAa}(i-1)(1-\Phi)(1-\Phi)F_{CcBbAa}(i-$
$1)+0.0625(1-\Phi)\Phi M_{CcBbAa}(i-1)\Phi(1-\Phi)F_{CcBbAa}(i-1)+0.125(1-\Phi)\Phi M_{CcBbAa}(i-1)F_{CcBBaa}(i-$
$1)+0.0625(1-\Phi)\Phi M_{CcBbAa}(i-1)(1-\Phi)F_{CcBBAa}(i-1)+0.25(1-\Phi)\Phi M_{CcBbAa}(i-1)F_{CCbbaa}(i-$
$1)+0.125(1-\Phi)\Phi M_{CcBbAa}(i-1)F_{CCbbAa}(i-1)+0.25(1-\Phi)\Phi M_{CcBbAa}(i-1)(1-\Phi)F_{CCBbaa}(i-1)+$
$0.25(1-\Phi)\Phi M_{CcBbAa}(i-1)\Phi F_{CCBbaa}(i-1)+0.125(1-\Phi)\Phi M_{CcBbAa}(i-1)(1-\Phi)(1-\Phi)F_{CCBbAa}(i-1)+$
$0.125(1-\Phi)\Phi M_{CcBbAa}(i-1)\Phi(1-\Phi)F_{CCBbAa}(i-1)+0.125(1-\Phi)\Phi M_{CcBbAa}(i-1)(1-\Phi)\Phi F_{CCBbAa}(i-$
$1)+0.25(1-\Phi)\Phi M_{CcBbAa}(i-1)F_{CCBBaa}(i-1)+0.125(1-\Phi)\Phi M_{CcBbAa}(i-1)(1-\Phi)F_{CCBBAa}(i-$
$1)+0.25\Phi\Phi M_{CcBbAa}(i-1)F_{Ccbbaa}(i-1)+0.125\Phi\Phi M_{CcBbAa}(i-1)F_{CcbbAa}(i-1)+0.125\Phi\Phi M_{CcBbAa}(i-$
$1)(1-\Phi)F_{CcBbaa}(i-1)+0.0625\Phi\Phi M_{CcBbAa}(i-1)(1-\Phi)(1-\Phi)F_{CcBbAa}(i-1)+0.5\Phi\Phi M_{CcBbAa}(i-$
$1)F_{CCbbaa}(i-1)+0.25\Phi\Phi M_{CcBbAa}(i-1)F_{CCbbAa}(i-1)+0.25\Phi\Phi M_{CcBbAa}(i-1)(1-\Phi)F_{CCBbaa}(i-$
$1)+0.125\Phi\Phi M_{CcBbAa}(i-1)(1-\Phi)(1-\Phi)F_{CCBbAa}(i-1)+0.125(1-\Phi)M_{CcBbAA}(i-1)F_{Ccbbaa}(i-$
$1)+0.0625(1-\Phi)M_{CcBbAA}(i-1)F_{CcbbAa}(i-1)+0.125(1-\Phi)M_{CcBbAA}(i-1)(1-\Phi)F_{CcBbaa}(i-1)+$
$0.125(1-\Phi)M_{CcBbAA}(i-1)\Phi F_{CcBbaa}(i-1)+0.0625(1-\Phi)M_{CcBbAA}(i-1)(1-\Phi)(1-\Phi)F_{CcBbAa}(i-$
$1)+0.0625(1-\Phi)M_{CcBbAA}(i-1)\Phi(1-\Phi)F_{CcBbAa}(i-1)+0.125(1-\Phi)M_{CcBbAA}(i-1)F_{CcBBaa}(i-$
$1)+0.0625(1-\Phi)M_{CcBbAA}(i-1)(1-\Phi)F_{CcBBAa}(i-1)+0.25(1-\Phi)M_{CcBbAA}(i-1)F_{CCbbaa}(i-$
$1)+0.125(1-\Phi)M_{CcBbAA}(i-1)F_{CCbbAa}(i-1)+0.25(1-\Phi)M_{CcBbAA}(i-1)(1-\Phi)F_{CCBbaa}(i-1)+$
$0.25(1-\Phi)M_{CcBbAA}(i-1)\Phi F_{CCBbaa}(i-1)+0.125(1-\Phi)M_{CcBbAA}(i-1)(1-\Phi)(1-\Phi)F_{CCBbAa}(i-1)+$
$0.125(1-\Phi)M_{CcBbAA}(i-1)\Phi(1-\Phi)F_{CCBbAa}(i-1)+0.125(1-\Phi)M_{CcBbAA}(i-1)(1-\Phi)\Phi F_{CCBbAa}(i-$
$1)+0.25(1-\Phi)M_{CcBbAA}(i-1)F_{CCBBaa}(i-1)+0.125(1-\Phi)M_{CcBbAA}(i-1)(1-\Phi)F_{CCBBAa}(i-$
$1)+0.25\Phi M_{CcBbAA}(i-1)F_{Ccbbaa}(i-1)+0.125\Phi M_{CcBbAA}(i-1)F_{CcbbAa}(i-1)+0.125\Phi M_{CcBbAA}(i-$
$1)(1-\Phi)F_{CcBbaa}(i-1)+0.0625\Phi M_{CcBbAA}(i-1)(1-\Phi)(1-\Phi)F_{CcBbAa}(i-1)+0.5\Phi M_{CcBbAA}(i-$
$1)F_{CCbbaa}(i-1)+0.25\Phi M_{CcBbAA}(i-1)F_{CCbbAa}(i-1)+0.25\Phi M_{CcBbAA}(i-1)(1-\Phi)F_{CCBbaa}(i-1)+$
$0.125\Phi M_{CcBbAA}(i-1)(1-\Phi)(1-\Phi)F_{CCBbAa}(i-1)+0.125M_{CcBBaa}(i-1)F_{CcbbAa}(i-1)+0.25M_{CcBBaa}(i-$
$1)F_{CcbbAA}(i-1)+0.0625M_{CcBBaa}(i-1)(1-\Phi)(1-\Phi)F_{CcBbAa}(i-1)+0.125M_{CcBBaa}(i-1)(1-$
$\Phi)\Phi F_{CcBbAa}(i-1)+0.125M_{CcBBaa}(i-1)(1-\Phi)F_{CcBbAA}(i-1)+0.25M_{CcBBaa}(i-1)F_{CCbbAa}(i-1)+$
$0.5M_{CcBBaa}(i-1)F_{CCbbAA}(i-1)+0.125M_{CcBBaa}(i-1)(1-\Phi)(1-\Phi)F_{CCBbAa}(i-1)+0.25M_{CcBBaa}(i-$
$1)(1-\Phi)F_{CCBbAA}(i-1)+0.125(1-\Phi)M_{CcBBAa}(i-1)F_{Ccbbaa}(i-1)+0.125(1-\Phi)M_{CcBBAa}(i-1)F_{CcbbAa}(i-$
$1)+0.125(1-\Phi)M_{CcBBAa}(i-1)F_{CcbbAA}(i-1)+0.0625(1-\Phi)M_{CcBBAa}(i-1)(1-\Phi)F_{CcBbaa}(i-$
$1)+0.0625(1-\Phi)M_{CcBBAa}(i-1)(1-\Phi)(1-\Phi)F_{CcBbAa}(i-1)+0.0625(1-\Phi)M_{CcBBAa}(i-1)(1-$
$\Phi)\Phi F_{CcBbAa}(i-1)+0.0625(1-\Phi)M_{CcBBAa}(i-1)(1-\Phi)F_{CcBbAA}(i-1)+0.25(1-\Phi)M_{CcBBAa}(i-$
$1)F_{CCbbaa}(i-1)+0.25(1-\Phi)M_{CcBBAa}(i-1)F_{CCbbAa}(i-1)+0.25(1-\Phi)M_{CcBBAa}(i-1)F_{CCbbAA}(i-$
$1)+0.125(1-\Phi)M_{CcBBAa}(i-1)(1-\Phi)F_{CCBbaa}(i-1)+0.125(1-\Phi)M_{CcBBAa}(i-1)(1-\Phi)(1-$
$\Phi)F_{CCBbAa}(i-1)+0.125(1-\Phi)M_{CcBBAa}(i-1)(1-\Phi)F_{CCBbAA}(i-1)+0.25\Phi M_{CcBBAa}(i-1)F_{Ccbbaa}(i-1)+$
$0.125\Phi M_{CcBBAa}(i-1)F_{CcbbAa}(i-1)+0.125\Phi M_{CcBBAa}(i-1)(1-\Phi)F_{CcBbaa}(i-1)+0.0625\Phi M_{CcBBAa}(i-$
$1)(1-\Phi)(1-\Phi)F_{CcBbAa}(i-1)+0.5\Phi M_{CcBBAa}(i-1)F_{CCbbaa}(i-1)+0.25\Phi M_{CcBBAa}(i-1)F_{CCbbAa}(i-$
$1)+0.25\Phi M_{CcBBAa}(i-1)(1-\Phi)F_{CCBbaa}(i-1)+0.125\Phi M_{CcBBAa}(i-1)(1-\Phi)(1-\Phi)F_{CCBbAa}(i-$
$1)+0.25M_{CcBBAA}(i-1)F_{Ccbbaa}(i-1)+0.125M_{CcBBAA}(i-1)F_{CcbbAa}(i-1)+0.125M_{CcBBAA}(i-$
$1)(1-\Phi)F_{CcBbaa}(i-1)+0.0625M_{CcBBAA}(i-1)(1-\Phi)(1-\Phi)F_{CcBbAa}(i-1)+0.5M_{CcBBAA}(i-$

$1)F_{CCbbaa}(i-1) + 0.25M_{CcBBAA}(i-1)F_{CCbbAa}(i-1) + 0.25M_{CcBBAA}(i-1)(1-\Phi)F_{CCBbaa}(i-1) +$
$0.125M_{CcBBAA}(i-1)(1-\Phi)(1-\Phi)F_{CCBbAa}(i-1) + 0.125M_{CCbbaa}(i-1)(1-\Phi)(1-\Phi)F_{CcBbAa}(i-1) +$
$0.25M_{CCbbaa}(i-1)\Phi(1-\Phi)F_{CcBbAa}(i-1) + 0.25M_{CCbbaa}(i-1)(1-\Phi)\Phi F_{CcBbAa}(i-1) + 0.5M_{CCbbaa}(i-$
$1)\Phi\Phi F_{CcBbAa}(i-1) + 0.25M_{CCbbaa}(i-1)(1-\Phi)F_{CcBbAA}(i-1) + 0.5M_{CCbbaa}(i-1)\Phi F_{CcBbAA}(i-$
$1) + 0.25M_{CCbbaa}(i-1)(1-\Phi)F_{CcBBAa}(i-1) + 0.5M_{CCbbaa}(i-1)\Phi F_{CcBBAa}(i-1) + 0.5M_{CCbbaa}(i-$
$1)F_{CcBBAA}(i-1)+0.25M_{CCbbaa}(i-1)(1-\Phi)(1-\Phi)F_{CCBbAa}(i-1)+0.5M_{CCbbaa}(i-1)\Phi(1-\Phi)F_{CCBbAa}(i-$
$1) + 0.5M_{CCbbaa}(i-1)(1-\Phi)\Phi F_{CCBbAa}(i-1) + M_{CCbbaa}(i-1)\Phi\Phi F_{CCBbAa}(i-1) + 0.5M_{CCbbaa}(i-$
$1)(1-\Phi)F_{CCBbAA}(i-1) + M_{CCbbaa}(i-1)\Phi F_{CCBbAA}(i-1) + 0.5M_{CCbbaa}(i-1)(1-\Phi)F_{CCBBAa}(i-$
$1) + M_{CCbbaa}(i-1)\Phi F_{CCBBAa}(i-1) + M_{CCbbaa}(i-1)F_{CCBBAA}(i-1) + 0.125M_{CCbbAa}(i-1)(1-$
$\Phi)F_{CcBbaa}(i-1)+0.25M_{CCbbAa}(i-1)\Phi F_{CcBbaa}(i-1)+0.125M_{CCbbAa}(i-1)(1-\Phi)(1-\Phi)F_{CcBbAa}(i-1)+$
$0.25M_{CCbbAa}(i-1)\Phi(1-\Phi)F_{CcBbAa}(i-1)+0.125M_{CCbbAa}(i-1)(1-\Phi)\Phi F_{CcBbAa}(i-1)+0.25M_{CCbbAa}(i-$
$1)\Phi\Phi F_{CcBbAa}(i-1) + 0.125M_{CCbbAa}(i-1)(1-\Phi)F_{CcBbAA}(i-1) + 0.25M_{CCbbAa}(i-1)\Phi F_{CcBbAA}(i-$
$1) + 0.25M_{CCbbAa}(i-1)F_{CcBBaa}(i-1) + 0.25M_{CCbbAa}(i-1)(1-\Phi)F_{CcBBAa}(i-1) + 0.25M_{CCbbAa}(i-$
$1)\Phi F_{CcBBAa}(i-1) + 0.25M_{CCbbAa}(i-1)F_{CcBBAA}(i-1) + 0.25M_{CCbbAa}(i-1)(1-\Phi)F_{CCBbaa}(i-1) +$
$0.5M_{CCbbAa}(i-1)\Phi F_{CCBbaa}(i-1) + 0.25M_{CCbbAa}(i-1)(1-\Phi)(1-\Phi)F_{CCBbAa}(i-1) + 0.5M_{CCbbAa}(i-$
$1)\Phi(1-\Phi)F_{CCBbAa}(i-1)+0.5M_{CCbbAa}(i-1)(1-\Phi)\Phi F_{CCBbAa}(i-1)+0.5M_{CCbbAa}(i-1)\Phi\Phi F_{CCBbAa}(i-$
$1) + 0.25M_{CCbbAa}(i-1)(1-\Phi)F_{CCBbAA}(i-1) + 0.5M_{CCbbAa}(i-1)\Phi F_{CCBbAA}(i-1) + 0.5M_{CCbbAa}(i-$
$1)F_{CCBBaa}(i-1) + 0.5M_{CCbbAa}(i-1)(1-\Phi)F_{CCBBAa}(i-1) + 0.5M_{CCbbAa}(i-1)\Phi F_{CCBBAa}(i-1) +$
$0.5M_{CCbbAa}(i-1)F_{CCBBAA}(i-1)+0.25M_{CCbbAA}(i-1)(1-\Phi)F_{CcBbaa}(i-1)+0.5M_{CCbbAA}(i-1)\Phi F_{CcBbaa}(i-$
$1) + 0.125M_{CCbbAA}(i-1)(1-\Phi)(1-\Phi)F_{CcBbAa}(i-1) + 0.25M_{CCbbAA}(i-1)\Phi(1-\Phi)F_{CcBbAa}(i-1) +$
$0.5M_{CCbbAA}(i-1)F_{CcBBaa}(i-1) + 0.25M_{CCbbAA}(i-1)(1-\Phi)F_{CcBBAa}(i-1) + 0.5M_{CCbbAA}(i-1)(1-$
$\Phi)F_{CCBbaa}(i-1) + M_{CCbbAA}(i-1)\Phi F_{CCBbaa}(i-1) + 0.25M_{CCbbAA}(i-1)(1-\Phi)(1-\Phi)F_{CCBbAa}(i-1)+$
$0.5M_{CCbbAA}(i-1)\Phi(1-\Phi)F_{CCBbAa}(i-1) + 0.5M_{CCbbAA}(i-1)(1-\Phi)\Phi F_{CCBbAa}(i-1) + M_{CCbbAA}(i-$
$1)F_{CCBBaa}(i-1) + 0.5M_{CCbbAA}(i-1)(1-\Phi)F_{CCBBAa}(i-1) + 0.125(1-\Phi)M_{CCBbaa}(i-1)F_{CcbbAa}(i-$
$1) + 0.25(1-\Phi)M_{CCBbaa}(i-1)F_{CcbbAA}(i-1) + 0.125(1-\Phi)M_{CCBbaa}(i-1)(1-\Phi)(1-\Phi)F_{CcBbAa}(i-$
$1)+0.125(1-\Phi)M_{CCBbaa}(i-1)\Phi(1-\Phi)F_{CcBbAa}(i-1)+0.25(1-\Phi)M_{CCBbaa}(i-1)(1-\Phi)\Phi F_{CcBbAa}(i-$
$1) + 0.25(1-\Phi)M_{CCBbaa}(i-1)\Phi\Phi F_{CcBbAa}(i-1) + 0.25(1-\Phi)M_{CCBbaa}(i-1)(1-\Phi)F_{CcBbAA}(i-$
$1) + 0.25(1-\Phi)M_{CCBbaa}(i-1)\Phi F_{CcBbAA}(i-1) + 0.125(1-\Phi)M_{CCBbaa}(i-1)(1-\Phi)F_{CcBBAa}(i-$
$1)+0.25(1-\Phi)M_{CCBbaa}(i-1)\Phi F_{CcBBAa}(i-1)+0.25(1-\Phi)M_{CCBbaa}(i-1)F_{CcBBAA}(i-1)+0.25(1-$
$\Phi)M_{CCBbaa}(i-1)F_{CCbbAa}(i-1)+0.5(1-\Phi)M_{CCBbaa}(i-1)F_{CCbbAA}(i-1)+0.25(1-\Phi)M_{CCBbaa}(i-1)(1-$
$\Phi)(1-\Phi)F_{CCBbAa}(i-1)+0.25(1-\Phi)M_{CCBbaa}(i-1)\Phi(1-\Phi)F_{CCBbAa}(i-1)+0.25(1-\Phi)M_{CCBbaa}(i-$
$1)(1-\Phi)\Phi F_{CCBbAa}(i-1) + 0.5(1-\Phi)M_{CCBbaa}(i-1)\Phi\Phi F_{CCBbAa}(i-1) + 0.5(1-\Phi)M_{CCBbaa}(i-$
$1)(1-\Phi)F_{CCBbAA}(i-1)+0.5(1-\Phi)M_{CCBbaa}(i-1)\Phi F_{CCBbAA}(i-1)+0.25(1-\Phi)M_{CCBbaa}(i-1)(1-$
$\Phi)F_{CCBBAa}(i-1)+0.5(1-\Phi)M_{CCBbaa}(i-1)\Phi F_{CCBBAa}(i-1)+0.5(1-\Phi)M_{CCBbaa}(i-1)F_{CCBBAA}(i-1)+$
$0.25\Phi M_{CCBbaa}(i-1)F_{CcbbAa}(i-1)+0.5\Phi M_{CCBbaa}(i-1)F_{CcbbAA}(i-1)+0.125\Phi M_{CCBbaa}(i-1)(1-\Phi)(1-$
$\Phi)F_{CcBbAa}(i-1)+0.25\Phi M_{CCBbaa}(i-1)(1-\Phi)\Phi F_{CcBbAa}(i-1)+0.25\Phi M_{CCBbaa}(i-1)(1-\Phi)F_{CcBbAA}(i-$
$1) + 0.5\Phi M_{CCBbaa}(i-1)F_{CCbbAa}(i-1) + \Phi M_{CCBbaa}(i-1)F_{CCbbAA}(i-1) + 0.25\Phi M_{CCBbaa}(i-1)(1-$
$\Phi)(1-\Phi)F_{CCBbAa}(i-1)+0.5\Phi M_{CCBbaa}(i-1)(1-\Phi)F_{CCBbAA}(i-1)+0.125(1-\Phi)(1-\Phi)M_{CCBbAa}(i-$
$1)F_{Ccbbaa}(i-1) + 0.125(1-\Phi)(1-\Phi)M_{CCBbAa}(i-1)F_{CcbbAa}(i-1) + 0.125(1-\Phi)(1-\Phi)M_{CCBbAa}(i-$
$1)F_{CcbbAA}(i-1)+0.125(1-\Phi)(1-\Phi)M_{CCBbAa}(i-1)(1-\Phi)F_{CcBbaa}(i-1)+0.125(1-\Phi)(1-\Phi)M_{CCBbAa}(i-$
$1)\Phi F_{CcBbaa}(i-1) + 0.125(1-\Phi)(1-\Phi)M_{CCBbAa}(i-1)(1-\Phi)(1-\Phi)F_{CcBbAa}(i-1) + 0.125(1-\Phi)(1-$
$\Phi)M_{CCBbAa}(i-1)\Phi(1-\Phi)F_{CcBbAa}(i-1) + 0.125(1-\Phi)(1-\Phi)M_{CCBbAa}(i-1)(1-\Phi)\Phi F_{CcBbAa}(i-1)+$
$0.125(1-\Phi)(1-\Phi)M_{CCBbAa}(i-1)\Phi\Phi F_{CcBbAa}(i-1)+0.125(1-\Phi)(1-\Phi)M_{CCBbAa}(i-1)(1-\Phi)F_{CcBbAA}(i-$
$1)+0.125(1-\Phi)(1-\Phi)M_{CCBbAa}(i-1)\Phi F_{CcBbAA}(i-1)+0.125(1-\Phi)(1-\Phi)M_{CCBbAa}(i-1)F_{CcBBaa}(i-1)+$
$0.125(1-\Phi)(1-\Phi)M_{CCBbAa}(i-1)(1-\Phi)F_{CcBBAa}(i-1)+0.125(1-\Phi)(1-\Phi)M_{CCBbAa}(i-1)\Phi F_{CcBBAa}(i-$
$1) + 0.125(1-\Phi)(1-\Phi)M_{CCBbAa}(i-1)F_{CcBBAA}(i-1) + 0.25(1-\Phi)(1-\Phi)M_{CCBbAa}(i-1)F_{CCbbaa}(i-$
$1)+0.25(1-\Phi)(1-\Phi)M_{CCBbAa}(i-1)F_{CCbbAa}(i-1)+0.25(1-\Phi)(1-\Phi)M_{CCBbAa}(i-1)F_{CCbbAA}(i-1)+$
$0.25(1-\Phi)(1-\Phi)M_{CCBbAa}(i-1)(1-\Phi)F_{CCBbaa}(i-1)+0.25(1-\Phi)(1-\Phi)M_{CCBbAa}(i-1)\Phi F_{CCBbaa}(i-$
$1) + 0.25(1-\Phi)(1-\Phi)M_{CCBbAa}(i-1)(1-\Phi)(1-\Phi)F_{CCBbAa}(i-1) + 0.25(1-\Phi)(1-\Phi)M_{CCBbAa}(i-$
$1)\Phi(1-\Phi)F_{CCBbAa}(i-1) + 0.25(1-\Phi)(1-\Phi)M_{CCBbAa}(i-1)(1-\Phi)\Phi F_{CCBbAa}(i-1) + 0.25(1-\Phi)(1-$
$\Phi)M_{CCBbAa}(i-1)\Phi\Phi F_{CCBbAa}(i-1)+0.25(1-\Phi)(1-\Phi)M_{CCBbAa}(i-1)(1-\Phi)F_{CCBbAA}(i-1)+0.25(1-$
$\Phi)(1-\Phi)M_{CCBbAa}(i-1)\Phi F_{CCBbAA}(i-1)+0.25(1-\Phi)(1-\Phi)M_{CCBbAa}(i-1)F_{CCBBaa}(i-1)+0.25(1-$
$\Phi)(1-\Phi)M_{CCBbAa}(i-1)(1-\Phi)F_{CCBBAa}(i-1) + 0.25(1-\Phi)(1-\Phi)M_{CCBbAa}(i-1)\Phi F_{CCBBAa}(i-$
$1) + 0.25(1-\Phi)(1-\Phi)M_{CCBbAa}(i-1)F_{CCBBAA}(i-1) + 0.25\Phi(1-\Phi)M_{CCBbAa}(i-1)F_{Ccbbaa}(i-1) +$
$0.25\Phi(1-\Phi)M_{CCBbAa}(i-1)F_{CcbbAa}(i-1) + 0.25\Phi(1-\Phi)M_{CCBbAa}(i-1)F_{CcbbAA}(i-1) + 0.125\Phi(1-$
$\Phi)M_{CCBbAa}(i-1)(1-\Phi)F_{CcBbaa}(i-1) + 0.125\Phi(1-\Phi)M_{CCBbAa}(i-1)(1-\Phi)(1-\Phi)F_{CcBbAa}(i-1) +$
$0.125\Phi(1-\Phi)M_{CCBbAa}(i-1)(1-\Phi)\Phi F_{CcBbAa}(i-1) + 0.125\Phi(1-\Phi)M_{CCBbAa}(i-1)(1-\Phi)F_{CcBbAA}(i-$

$1) + 0.5\Phi(1-\Phi)M_{CCBbAa}(i-1)F_{CCbbaa}(i-1) + 0.5\Phi(1-\Phi)M_{CCBbAa}(i-1)F_{CCbbAa}(i-1) + 0.5\Phi(1-$
$\Phi)M_{CCBbAa}(i-1)F_{CCbbAA}(i-1) + 0.25\Phi(1-\Phi)M_{CCBbAa}(i-1)(1-\Phi)F_{CCBbaa}(i-1) + 0.25\Phi(1-$
$\Phi)M_{CCBbAa}(i-1)(1-\Phi)(1-\Phi)F_{CCBbAa}(i-1) + 0.25\Phi(1-\Phi)M_{CCBbAa}(i-1)(1-\Phi)F_{CCBbAA}(i-1)+$
$0.25(1-\Phi)\Phi M_{CCBbAa}(i-1)F_{Ccbbaa}(i-1) + 0.125(1-\Phi)\Phi M_{CCBbAa}(i-1)F_{CcbbAa}(i-1) + 0.25(1-$
$\Phi)\Phi M_{CCBbAa}(i-1)(1-\Phi)F_{CcBbaa}(i-1) + 0.25(1-\Phi)\Phi M_{CCBbAa}(i-1)\Phi F_{CcBbaa}(i-1) + 0.125(1-$
$\Phi)\Phi M_{CCBbAa}(i-1)(1-\Phi)(1-\Phi)F_{CcBbAa}(i-1)+0.125(1-\Phi)\Phi M_{CCBbAa}(i-1)\Phi(1-\Phi)F_{CcBbAa}(i-1)+$
$0.25(1-\Phi)\Phi M_{CCBbAa}(i-1)F_{CcBBaa}(i-1)+0.125(1-\Phi)\Phi M_{CCBbAa}(i-1)(1-\Phi)F_{CcBBAa}(i-1)+0.5(1-$
$\Phi)\Phi M_{CCBbAa}(i-1)F_{CCbbaa}(i-1)+0.25(1-\Phi)\Phi M_{CCBbAa}(i-1)F_{CCbbAa}(i-1)+0.5(1-\Phi)\Phi M_{CCBbAa}(i-$
$1)(1-\Phi)F_{CCBbaa}(i-1)+0.5(1-\Phi)\Phi M_{CCBbAa}(i-1)\Phi F_{CCBbaa}(i-1)+0.25(1-\Phi)\Phi M_{CCBbAa}(i-1)(1-$
$\Phi)(1-\Phi)F_{CCBbAa}(i-1)+0.25(1-\Phi)\Phi M_{CCBbAa}(i-1)\Phi(1-\Phi)F_{CCBbAa}(i-1)+0.25(1-\Phi)\Phi M_{CCBbAa}(i-$
$1)(1-\Phi)\Phi F_{CCBbAa}(i-1) + 0.5(1-\Phi)\Phi M_{CCBbAa}(i-1)F_{CCBBaa}(i-1) + 0.25(1-\Phi)\Phi M_{CCBbAa}(i-$
$1)(1-\Phi)F_{CCBBAa}(i-1)+0.5\Phi\Phi M_{CCBbAa}(i-1)F_{Ccbbaa}(i-1)+0.25\Phi\Phi M_{CCBbAa}(i-1)F_{CcbbAa}(i-1)+$
$0.25\Phi\Phi M_{CCBbAa}(i-1)(1-\Phi)F_{CcBbaa}(i-1) + 0.125\Phi\Phi M_{CCBbAa}(i-1)(1-\Phi)(1-\Phi)F_{CcBbAa}(i-1)+$
$\Phi\Phi M_{CCBbAa}(i-1)F_{CCbbaa}(i-1) + 0.5\Phi\Phi M_{CCBbAa}(i-1)F_{CCbbAa}(i-1) + 0.5\Phi\Phi M_{CCBbAa}(i-1)(1-$
$\Phi)F_{CCBbaa}(i-1) + 0.25\Phi\Phi M_{CCBbAa}(i-1)(1-\Phi)(1-\Phi)F_{CCBbAa}(i-1) + 0.25(1-\Phi)M_{CCBbAA}(i-$
$1)F_{Ccbbaa}(i-1)+0.125(1-\Phi)M_{CCBbAA}(i-1)F_{CcbbAa}(i-1)+0.25(1-\Phi)M_{CCBbAA}(i-1)(1-\Phi)F_{CcBbaa}(i-$
$1) + 0.25(1-\Phi)M_{CCBbAA}(i-1)\Phi F_{CcBbaa}(i-1) + 0.125(1-\Phi)M_{CCBbAA}(i-1)(1-\Phi)(1-\Phi)F_{CcBbAa}(i-$
$1) + 0.125(1-\Phi)M_{CCBbAA}(i-1)\Phi(1-\Phi)F_{CcBbAa}(i-1) + 0.25(1-\Phi)M_{CCBbAA}(i-1)F_{CcBBaa}(i-1)+$
$0.125(1-\Phi)M_{CCBbAA}(i-1)(1-\Phi)F_{CcBBAa}(i-1) + 0.5(1-\Phi)M_{CCBbAA}(i-1)F_{CCbbaa}(i-1) + 0.25(1-$
$\Phi)M_{CCBbAA}(i-1)F_{CCbbAa}(i-1)+0.5(1-\Phi)M_{CCBbAA}(i-1)(1-\Phi)F_{CCBbaa}(i-1)+0.5(1-\Phi)M_{CCBbAA}(i-$
$1)\Phi F_{CCBbaa}(i-1) + 0.25(1-\Phi)M_{CCBbAA}(i-1)(1-\Phi)(1-\Phi)F_{CCBbAa}(i-1) + 0.25(1-\Phi)M_{CCBbAA}(i-$
$1)\Phi(1-\Phi)F_{CCBbAa}(i-1) + 0.25(1-\Phi)M_{CCBbAA}(i-1)(1-\Phi)\Phi F_{CCBbAa}(i-1) + 0.5(1-\Phi)M_{CCBbAA}(i-$
$1)F_{CCBBaa}(i-1)+0.25(1-\Phi)M_{CCBbAA}(i-1)(1-\Phi)F_{CCBBAa}(i-1)+0.5\Phi M_{CCBbAA}(i-1)F_{Ccbbaa}(i-1)+$
$0.25\Phi M_{CCBbAA}(i-1)F_{CcbbAa}(i-1) + 0.25\Phi M_{CCBbAA}(i-1)(1-\Phi)F_{CcBbaa}(i-1) + 0.125\Phi M_{CCBbAA}(i-$
$1)(1-\Phi)(1-\Phi)F_{CcBbAa}(i-1) + \Phi M_{CCBbAA}(i-1)F_{CCbbaa}(i-1) + 0.5\Phi M_{CCBbAA}(i-1)F_{CCbbAa}(i-$
$1) + 0.5\Phi M_{CCBbAA}(i-1)(1-\Phi)F_{CCBbaa}(i-1) + 0.25\Phi M_{CCBbAA}(i-1)(1-\Phi)(1-\Phi)F_{CCBbAa}(i-1)+$
$0.25M_{CCBBaa}(i-1)F_{CcbbAa}(i-1)+0.5M_{CCBBaa}(i-1)F_{CcbbAA}(i-1)+0.125M_{CCBBaa}(i-1)(1-\Phi)(1-$
$\Phi)F_{CcBbAa}(i-1)+0.25M_{CCBBaa}(i-1)(1-\Phi)\Phi F_{CcBbAa}(i-1)+0.25M_{CCBBaa}(i-1)(1-\Phi)F_{CcBbAA}(i-$
$1) + 0.5M_{CCBBaa}(i-1)F_{CCbbAa}(i-1) + M_{CCBBaa}(i-1)F_{CCbbAA}(i-1) + 0.25M_{CCBBaa}(i-1)(1-\Phi)(1-$
$\Phi)F_{CCBbAa}(i-1) + 0.5M_{CCBBaa}(i-1)(1-\Phi)F_{CCBbAA}(i-1) + 0.25(1-\Phi)M_{CCBBAa}(i-1)F_{Ccbbaa}(i-$
$1) + 0.25(1-\Phi)M_{CCBBAa}(i-1)F_{CcbbAa}(i-1) + 0.25(1-\Phi)M_{CCBBAa}(i-1)F_{CcbbAA}(i-1) + 0.125(1-$
$\Phi)M_{CCBBAa}(i-1)(1-\Phi)F_{CcBbaa}(i-1) + 0.125(1-\Phi)M_{CCBBAa}(i-1)(1-\Phi)(1-\Phi)F_{CcBbAa}(i-1)+$
$0.125(1-\Phi)M_{CCBBAa}(i-1)(1-\Phi)\Phi F_{CcBbAa}(i-1)+0.125(1-\Phi)M_{CCBBAa}(i-1)(1-\Phi)F_{CcBbAA}(i-1)+$
$0.5(1-\Phi)M_{CCBBAa}(i-1)F_{CCbbaa}(i-1)+0.5(1-\Phi)M_{CCBBAa}(i-1)F_{CCbbAa}(i-1)+0.5(1-\Phi)M_{CCBBAa}(i-$
$1)F_{CCbbAA}(i-1)+0.25(1-\Phi)M_{CCBBAa}(i-1)(1-\Phi)F_{CCBbaa}(i-1)+0.25(1-\Phi)M_{CCBBAa}(i-1)(1-\Phi)(1-$
$\Phi)F_{CCBbAa}(i-1)+0.25(1-\Phi)M_{CCBBAa}(i-1)(1-\Phi)F_{CCBbAA}(i-1)+0.5\Phi M_{CCBBAa}(i-1)F_{Ccbbaa}(i-1)+$
$0.25\Phi M_{CCBBAa}(i-1)F_{CcbbAa}(i-1)+0.25\Phi M_{CCBBAa}(i-1)(1-\Phi)F_{CcBbaa}(i-1)+0.125\Phi M_{CCBBAa}(i-$
$1)(1-\Phi)(1-\Phi)F_{CcBbAa}(i-1) + \Phi M_{CCBBAa}(i-1)F_{CCbbaa}(i-1) + 0.5\Phi M_{CCBBAa}(i-1)F_{CCbbAa}(i-$
$1) + 0.5\Phi M_{CCBBAa}(i-1)(1-\Phi)F_{CCBbaa}(i-1) + 0.25\Phi M_{CCBBAa}(i-1)(1-\Phi)(1-\Phi)F_{CCBbAa}(i-$
$1) + 0.5M_{CCBBAA}(i-1)F_{Ccbbaa}(i-1) + 0.25M_{CCBBAA}(i-1)F_{CcbbAa}(i-1) + 0.25M_{CCBBAA}(i-1)(1-$
$\Phi)F_{CcBbaa}(i-1) + 0.125M_{CCBBAA}(i-1)(1-\Phi)(1-\Phi)F_{CcBbAa}(i-1) + M_{CCBBAA}(i-1)F_{CCbbaa}(i-$
$1) + 0.5M_{CCBBAA}(i-1)F_{CCbbAa}(i-1) + 0.5M_{CCBBAA}(i-1)(1-\Phi)F_{CCBbaa}(i-1) + 0.25M_{CCBBAA}(i-$
$1)(1-\Phi)(1-\Phi)F_{CCBbAa}(i-1)],$

$\mathrm{M}^{e}_{CCBbAA} = \left(\frac{\bar{\Omega}_{CCBbAA}}{2}\right)[0.03125M_{CcbbAa}(i-1)(1-\Phi)(1-\Phi)F_{CcBbAa}(i-1) + 0.0625M_{CcbbAa}(i-$
$1)\Phi(1-\Phi)F_{CcBbAa}(i-1)+0.0625M_{CcbbAa}(i-1)(1-\Phi)\Phi F_{CcBbAa}(i-1)+0.125M_{CcbbAa}(i-1)\Phi\Phi F_{CcBbAa}(i-$
$1)+0.0625M_{CcbbAa}(i-1)(1-\Phi)F_{CcBbAA}(i-1)+0.125M_{CcbbAa}(i-1)\Phi F_{CcBbAA}(i-1)+0.0625M_{CcbbAa}(i-$
$1)(1-\Phi)F_{CcBBAa}(i-1) + 0.125M_{CcbbAa}(i-1)\Phi F_{CcBBAa}(i-1) + 0.125M_{CcbbAa}(i-1)F_{CcBBAA}(i-1)+$
$0.0625M_{CcbbAa}(i-1)(1-\Phi)(1-\Phi)F_{CCBbAa}(i-1) + 0.125M_{CcbbAa}(i-1)\Phi(1-\Phi)F_{CCBbAa}(i-1)+$
$0.125M_{CcbbAa}(i-1)(1-\Phi)\Phi F_{CCBbAa}(i-1) + 0.25M_{CcbbAa}(i-1)\Phi\Phi F_{CCBbAa}(i-1) + 0.125M_{CcbbAa}(i-$
$1)(1-\Phi)F_{CCBbAA}(i-1)+0.25M_{CcbbAa}(i-1)\Phi F_{CCBbAA}(i-1)+0.125M_{CcbbAa}(i-1)(1-\Phi)F_{CCBBAa}(i-$
$1) + 0.25M_{CcbbAa}(i-1)\Phi F_{CCBBAa}(i-1) + 0.25M_{CcbbAa}(i-1)F_{CCBBAA}(i-1) + 0.0625M_{CcbbAA}(i-$
$1)(1-\Phi)(1-\Phi)F_{CcBbAa}(i-1) + 0.125M_{CcbbAA}(i-1)\Phi(1-\Phi)F_{CcBbAa}(i-1) + 0.125M_{CcbbAA}(i-1)(1-$
$\Phi)\Phi F_{CcBbAa}(i-1) + 0.25M_{CcbbAA}(i-1)\Phi\Phi F_{CcBbAa}(i-1) + 0.125M_{CcbbAA}(i-1)(1-\Phi)F_{CcBbAA}(i-$
$1) + 0.25M_{CcbbAA}(i-1)\Phi F_{CcBbAA}(i-1) + 0.125M_{CcbbAA}(i-1)(1-\Phi)F_{CcBBAa}(i-1) + 0.25M_{CcbbAA}(i-$
$1)\Phi F_{CcBBAa}(i-1)+0.25M_{CcbbAA}(i-1)F_{CcBBAA}(i-1)+0.125M_{CcbbAA}(i-1)(1-\Phi)(1-\Phi)F_{CCBbAa}(i-1)+$
$0.25M_{CcbbAA}(i-1)\Phi(1-\Phi)F_{CCBbAa}(i-1)+0.25M_{CcbbAA}(i-1)(1-\Phi)\Phi F_{CCBbAa}(i-1)+0.5M_{CcbbAA}(i-$

$1)\Phi\Phi F_{CCBbAa}(i-1) + 0.25M_{CcbbAA}(i-1)(1-\Phi)F_{CCBbAA}(i-1) + 0.5M_{CcbbAA}(i-1)\Phi F_{CCBbAA}(i-$
$1) + 0.25M_{CcbbAA}(i-1)(1-\Phi)F_{CCBBAa}(i-1) + 0.5M_{CcbbAA}(i-1)\Phi F_{CCBBAa}(i-1) + 0.5M_{CcbbAA}(i-$
$1)F_{CCBBAA}(i-1)+0.03125(1-\Phi)(1-\Phi)M_{CcBbAa}(i-1)F_{CcbbAa}(i-1)+0.0625(1-\Phi)(1-\Phi)M_{CcBbAa}(i-$
$1)F_{CcbbAA}(i-1)+0.03125(1-\Phi)(1-\Phi)M_{CcBbAa}(i-1)(1-\Phi)(1-\Phi)F_{CcBbAa}(i-1)+0.03125(1-\Phi)(1-$
$\Phi)M_{CcBbAa}(i-1)\Phi(1-\Phi)F_{CcBbAa}(i-1) + 0.0625(1-\Phi)(1-\Phi)M_{CcBbAa}(i-1)(1-\Phi)\Phi F_{CcBbAa}(i-$
$1) + 0.0625(1-\Phi)(1-\Phi)M_{CcBbAa}(i-1)\Phi\Phi F_{CcBbAa}(i-1) + 0.0625(1-\Phi)(1-\Phi)M_{CcBbAa}(i-1)(1-$
$\Phi)F_{CcBbAA}(i-1)+0.0625(1-\Phi)(1-\Phi)M_{CcBbAa}(i-1)\Phi F_{CcBbAA}(i-1)+0.03125(1-\Phi)(1-\Phi)M_{CcBbAa}(i-$
$1)(1-\Phi)F_{CcBBAa}(i-1) + 0.0625(1-\Phi)(1-\Phi)M_{CcBbAa}(i-1)\Phi F_{CcBBAa}(i-1) + 0.0625(1-\Phi)(1-$
$\Phi)M_{CcBbAa}(i-1)F_{CcBBAA}(i-1) + 0.0625(1-\Phi)(1-\Phi)M_{CcBbAa}(i-1)F_{CCbbAa}(i-1) + 0.125(1-$
$\Phi)(1-\Phi)M_{CcBbAa}(i-1)F_{CCbbAA}(i-1) + 0.0625(1-\Phi)(1-\Phi)M_{CcBbAa}(i-1)(1-\Phi)(1-\Phi)F_{CCBbAa}(i-$
$1) + 0.0625(1-\Phi)(1-\Phi)M_{CcBbAa}(i-1)\Phi(1-\Phi)F_{CCBbAa}(i-1) + 0.0625(1-\Phi)(1-\Phi)M_{CcBbAa}(i-$
$1)(1-\Phi)\Phi F_{CCBbAa}(i-1) + 0.125(1-\Phi)(1-\Phi)M_{CcBbAa}(i-1)\Phi\Phi F_{CCBbAa}(i-1) + 0.125(1-\Phi)(1-$
$\Phi)M_{CcBbAa}(i-1)(1-\Phi)F_{CCBbAA}(i-1)+0.125(1-\Phi)(1-\Phi)M_{CcBbAa}(i-1)\Phi F_{CCBbAA}(i-1)+0.0625(1-$
$\Phi)(1-\Phi)M_{CcBbAa}(i-1)(1-\Phi)F_{CCBBAa}(i-1) + 0.125(1-\Phi)(1-\Phi)M_{CcBbAa}(i-1)\Phi F_{CCBBAa}(i-$
$1) + 0.125(1-\Phi)(1-\Phi)M_{CcBbAa}(i-1)F_{CCBBAA}(i-1) + 0.0625\Phi(1-\Phi)M_{CcBbAa}(i-1)F_{CcbbAa}(i-1)+$
$0.125\Phi(1-\Phi)M_{CcBbAa}(i-1)F_{CcbbAA}(i-1)+0.03125\Phi(1-\Phi)M_{CcBbAa}(i-1)(1-\Phi)(1-\Phi)F_{CcBbAa}(i-1)+$
$0.0625\Phi(1-\Phi)M_{CcBbAa}(i-1)(1-\Phi)\Phi F_{CcBbAa}(i-1)+0.0625\Phi(1-\Phi)M_{CcBbAa}(i-1)(1-\Phi)F_{CcBbAA}(i-$
$1)+0.125\Phi(1-\Phi)M_{CcBbAa}(i-1)F_{CCbbAa}(i-1)+0.25\Phi(1-\Phi)M_{CcBbAa}(i-1)F_{CCbbAA}(i-1)+0.0625\Phi(1-$
$\Phi)M_{CcBbAa}(i-1)(1-\Phi)(1-\Phi)F_{CCBbAa}(i-1) + 0.125\Phi(1-\Phi)M_{CcBbAa}(i-1)(1-\Phi)F_{CCBbAA}(i-1)+$
$0.0625(1-\Phi)\Phi M_{CcBbAa}(i-1)F_{CcbbAa}(i-1) + 0.125(1-\Phi)\Phi M_{CcBbAa}(i-1)F_{CcbbAA}(i-1) + 0.0625(1-$
$\Phi)\Phi M_{CcBbAa}(i-1)(1-\Phi)(1-\Phi)F_{CcBbAa}(i-1) + 0.0625(1-\Phi)\Phi M_{CcBbAa}(i-1)\Phi(1-\Phi)F_{CcBbAa}(i-$
$1) + 0.125(1-\Phi)\Phi M_{CcBbAa}(i-1)(1-\Phi)\Phi F_{CcBbAa}(i-1) + 0.125(1-\Phi)\Phi M_{CcBbAa}(i-1)\Phi\Phi F_{CcBbAa}(i-$
$1) + 0.125(1-\Phi)\Phi M_{CcBbAa}(i-1)(1-\Phi)F_{CcBbAA}(i-1) + 0.125(1-\Phi)\Phi M_{CcBbAa}(i-1)\Phi F_{CcBbAA}(i-$
$1) + 0.0625(1-\Phi)\Phi M_{CcBbAa}(i-1)(1-\Phi)F_{CcBBAa}(i-1) + 0.125(1-\Phi)\Phi M_{CcBbAa}(i-1)\Phi F_{CcBBAa}(i-$
$1) + 0.125(1-\Phi)\Phi M_{CcBbAa}(i-1)F_{CcBBAA}(i-1) + 0.125(1-\Phi)\Phi M_{CcBbAa}(i-1)F_{CCbbAa}(i-1) +$
$0.25(1-\Phi)\Phi M_{CcBbAa}(i-1)F_{CCbbAA}(i-1)+0.125(1-\Phi)\Phi M_{CcBbAa}(i-1)(1-\Phi)(1-\Phi)F_{CCBbAa}(i-1)+$
$0.125(1-\Phi)\Phi M_{CcBbAa}(i-1)\Phi(1-\Phi)F_{CCBbAa}(i-1)+0.125(1-\Phi)\Phi M_{CcBbAa}(i-1)(1-\Phi)\Phi F_{CCBbAa}(i-$
$1) + 0.25(1-\Phi)\Phi M_{CcBbAa}(i-1)\Phi\Phi F_{CCBbAa}(i-1) + 0.25(1-\Phi)\Phi M_{CcBbAa}(i-1)(1-\Phi)F_{CCBbAA}(i-$
$1) + 0.25(1-\Phi)\Phi M_{CcBbAa}(i-1)\Phi F_{CCBbAA}(i-1) + 0.125(1-\Phi)\Phi M_{CcBbAa}(i-1)(1-\Phi)F_{CCBBAa}(i-$
$1) + 0.25(1-\Phi)\Phi M_{CcBbAa}(i-1)\Phi F_{CCBBAa}(i-1) + 0.25(1-\Phi)\Phi M_{CcBbAa}(i-1)F_{CCBBAA}(i-1) +$
$0.125\Phi\Phi M_{CcBbAa}(i-1)F_{CcbbAa}(i-1) + 0.25\Phi\Phi M_{CcBbAa}(i-1)F_{CcbbAA}(i-1) + 0.0625\Phi\Phi M_{CcBbAa}(i-$
$1)(1-\Phi)(1-\Phi)F_{CcBbAa}(i-1) + 0.125\Phi\Phi M_{CcBbAa}(i-1)(1-\Phi)\Phi F_{CcBbAa}(i-1) + 0.125\Phi\Phi M_{CcBbAa}(i-$
$1)(1-\Phi)F_{CcBbAA}(i-1) + 0.25\Phi\Phi M_{CcBbAa}(i-1)F_{CCbbAa}(i-1) + 0.5\Phi\Phi M_{CcBbAa}(i-1)F_{CCbbAA}(i-1)+$
$0.125\Phi\Phi M_{CcBbAa}(i-1)(1-\Phi)(1-\Phi)F_{CCBbAa}(i-1) + 0.25\Phi\Phi M_{CcBbAa}(i-1)(1-\Phi)F_{CCBbAA}(i-1)+$
$0.0625(1-\Phi)M_{CcBbAA}(i-1)F_{CcbbAa}(i-1) + 0.125(1-\Phi)M_{CcBbAA}(i-1)F_{CcbbAA}(i-1) + 0.0625(1-$
$\Phi)M_{CcBbAA}(i-1)(1-\Phi)(1-\Phi)F_{CcBbAa}(i-1) + 0.0625(1-\Phi)M_{CcBbAA}(i-1)\Phi(1-\Phi)F_{CcBbAa}(i-$
$1) + 0.125(1-\Phi)M_{CcBbAA}(i-1)(1-\Phi)\Phi F_{CcBbAa}(i-1) + 0.125(1-\Phi)M_{CcBbAA}(i-1)\Phi\Phi F_{CcBbAa}(i-$
$1) + 0.125(1-\Phi)M_{CcBbAA}(i-1)(1-\Phi)F_{CcBbAA}(i-1) + 0.125(1-\Phi)M_{CcBbAA}(i-1)\Phi F_{CcBbAA}(i-1)+$
$0.0625(1-\Phi)M_{CcBbAA}(i-1)(1-\Phi)F_{CcBBAa}(i-1) + 0.125(1-\Phi)M_{CcBbAA}(i-1)\Phi F_{CcBBAa}(i-1)+$
$0.125(1-\Phi)M_{CcBbAA}(i-1)F_{CcBBAA}(i-1) + 0.125(1-\Phi)M_{CcBbAA}(i-1)F_{CCbbAa}(i-1) + 0.25(1-$
$\Phi)M_{CcBbAA}(i-1)F_{CCbbAA}(i-1) + 0.125(1-\Phi)M_{CcBbAA}(i-1)(1-\Phi)(1-\Phi)F_{CCBbAa}(i-1) + 0.125(1-$
$\Phi)M_{CcBbAA}(i-1)\Phi(1-\Phi)F_{CCBbAa}(i-1)+0.125(1-\Phi)M_{CcBbAA}(i-1)(1-\Phi)\Phi F_{CCBbAa}(i-1)+0.25(1-$
$\Phi)M_{CcBbAA}(i-1)\Phi\Phi F_{CCBbAa}(i-1) + 0.25(1-\Phi)M_{CcBbAA}(i-1)(1-\Phi)F_{CCBbAA}(i-1) + 0.25(1-$
$\Phi)M_{CcBbAA}(i-1)\Phi F_{CCBbAA}(i-1) + 0.125(1-\Phi)M_{CcBbAA}(i-1)(1-\Phi)F_{CCBBAa}(i-1) + 0.25(1-$
$\Phi)M_{CcBbAA}(i-1)\Phi F_{CCBBAa}(i-1) + 0.25(1-\Phi)M_{CcBbAA}(i-1)F_{CCBBAA}(i-1) + 0.125\Phi M_{CcBbAA}(i-$
$1)F_{CcbbAa}(i-1)+0.25\Phi M_{CcBbAA}(i-1)F_{CcbbAA}(i-1)+0.0625\Phi M_{CcBbAA}(i-1)(1-\Phi)(1-\Phi)F_{CcBbAa}(i-$
$1) + 0.125\Phi M_{CcBbAA}(i-1)(1-\Phi)\Phi F_{CcBbAa}(i-1) + 0.125\Phi M_{CcBbAA}(i-1)(1-\Phi)F_{CcBbAA}(i-1) +$
$0.25\Phi M_{CcBbAA}(i-1)F_{CCbbAa}(i-1) + 0.5\Phi M_{CcBbAA}(i-1)F_{CCbbAA}(i-1) + 0.125\Phi M_{CcBbAA}(i-1)(1-$
$\Phi)(1-\Phi)F_{CCBbAa}(i-1) + 0.25\Phi M_{CcBbAA}(i-1)(1-\Phi)F_{CCBbAA}(i-1) + 0.0625(1-\Phi)M_{CcBBAa}(i-$
$1)F_{CcbbAa}(i-1)+0.125(1-\Phi)M_{CcBBAa}(i-1)F_{CcbbAA}(i-1)+0.03125(1-\Phi)M_{CcBBAa}(i-1)(1-\Phi)(1-$
$\Phi)F_{CcBbAa}(i-1)+0.0625(1-\Phi)M_{CcBBAa}(i-1)(1-\Phi)\Phi F_{CcBbAa}(i-1)+0.0625(1-\Phi)M_{CcBBAa}(i-1)(1-$
$\Phi)F_{CcBbAA}(i-1)+0.125(1-\Phi)M_{CcBBAa}(i-1)F_{CCbbAa}(i-1)+0.25(1-\Phi)M_{CcBBAa}(i-1)F_{CCbbAA}(i-1)+$
$0.0625(1-\Phi)M_{CcBBAa}(i-1)(1-\Phi)(1-\Phi)F_{CCBbAa}(i-1)+0.125(1-\Phi)M_{CcBBAa}(i-1)(1-\Phi)F_{CCBbAA}(i-$
$1) + 0.125\Phi M_{CcBBAa}(i-1)F_{CcbbAa}(i-1) + 0.25\Phi M_{CcBBAa}(i-1)F_{CcbbAA}(i-1) + 0.0625\Phi M_{CcBBAa}(i-$
$1)(1-\Phi)(1-\Phi)F_{CcBbAa}(i-1) + 0.125\Phi M_{CcBBAa}(i-1)(1-\Phi)\Phi F_{CcBbAa}(i-1) + 0.125\Phi M_{CcBBAa}(i-$
$1)(1-\Phi)F_{CcBbAA}(i-1) + 0.25\Phi M_{CcBBAa}(i-1)F_{CCbbAa}(i-1) + 0.5\Phi M_{CcBBAa}(i-1)F_{CCbbAA}(i-1)+$
$0.125\Phi M_{CcBBAa}(i-1)(1-\Phi)(1-\Phi)F_{CCBbAa}(i-1) + 0.25\Phi M_{CcBBAa}(i-1)(1-\Phi)F_{CCBbAA}(i-1)+$

$0.125M_{CcBBAA}(i-1)F_{CcbbAa}(i-1)+0.25M_{CcBBAA}(i-1)F_{CcbbAA}(i-1)+0.0625M_{CcBBAA}(i-1)(1-\Phi)(1-$
$\Phi)F_{CcBbAa}(i-1)+0.125M_{CcBBAA}(i-1)(1-\Phi)\Phi F_{CcBbAa}(i-1)+0.125M_{CcBBAA}(i-1)(1-\Phi)F_{CcBbAA}(i-$
$1)+0.25M_{CcBBAA}(i-1)F_{CCbbAa}(i-1)+0.5M_{CcBBAA}(i-1)F_{CCbbAA}(i-1)+0.125M_{CcBBAA}(i-1)(1-$
$\Phi)(1-\Phi)F_{CCBbAa}(i-1)+0.25M_{CcBBAA}(i-1)(1-\Phi)F_{CCBbAA}(i-1)+0.0625M_{CCbbAa}(i-1)(1-\Phi)(1-$
$\Phi)F_{CcBbAa}(i-1)+0.125M_{CCbbAa}(i-1)\Phi(1-\Phi)F_{CcBbAa}(i-1)+0.125M_{CCbbAa}(i-1)(1-\Phi)\Phi F_{CcBbAa}(i-$
$1)+0.25M_{CCbbAa}(i-1)\Phi\Phi F_{CcBbAa}(i-1)+0.125M_{CCbbAa}(i-1)(1-\Phi)F_{CcBbAA}(i-1)+0.25M_{CCbbAa}(i-$
$1)\Phi F_{CcBbAA}(i-1)+0.125M_{CCbbAa}(i-1)(1-\Phi)F_{CcBBAa}(i-1)+0.25M_{CCbbAa}(i-1)\Phi F_{CcBBAa}(i-1)+$
$0.25M_{CCbbAa}(i-1)F_{CcBBAA}(i-1)+0.125M_{CCbbAa}(i-1)(1-\Phi)(1-\Phi)F_{CCBbAa}(i-1)+0.25M_{CCbbAa}(i-$
$1)\Phi(1-\Phi)F_{CCBbAa}(i-1)+0.25M_{CCbbAa}(i-1)(1-\Phi)\Phi F_{CCBbAa}(i-1)+0.5M_{CCbbAa}(i-1)\Phi\Phi F_{CCBbAa}(i-$
$1)+0.25M_{CCbbAa}(i-1)(1-\Phi)F_{CCBbAA}(i-1)+0.5M_{CCbbAa}(i-1)\Phi F_{CCBbAA}(i-1)+0.25M_{CCbbAa}(i-$
$1)(1-\Phi)F_{CCBBAa}(i-1)+0.5M_{CCbbAa}(i-1)\Phi F_{CCBBAa}(i-1)+0.5M_{CCbbAa}(i-1)F_{CCBBAA}(i-1)+$
$0.125M_{CCbbAA}(i-1)(1-\Phi)(1-\Phi)F_{CcBbAa}(i-1)+0.25M_{CCbbAA}(i-1)\Phi(1-\Phi)F_{CcBbAa}(i-1)+$
$0.25M_{CCbbAA}(i-1)(1-\Phi)\Phi F_{CcBbAa}(i-1)+0.5M_{CCbbAA}(i-1)\Phi\Phi F_{CcBbAa}(i-1)+0.25M_{CCbbAA}(i-$
$1)(1-\Phi)F_{CcBbAA}(i-1)+0.5M_{CCbbAA}(i-1)\Phi F_{CcBbAA}(i-1)+0.25M_{CCbbAA}(i-1)(1-\Phi)F_{CcBBAa}(i-1)+$
$0.5M_{CCbbAA}(i-1)\Phi F_{CcBBAa}(i-1)+0.5M_{CCbbAA}(i-1)F_{CcBBAA}(i-1)+0.25M_{CCbbAA}(i-1)(1-\Phi)(1-$
$\Phi)F_{CCBbAa}(i-1)+0.5M_{CCbbAA}(i-1)\Phi(1-\Phi)F_{CCBbAa}(i-1)+0.5M_{CCbbAA}(i-1)(1-\Phi)\Phi F_{CCBbAa}(i-$
$1)+M_{CCbbAA}(i-1)\Phi\Phi F_{CCBbAa}(i-1)+0.5M_{CCbbAA}(i-1)(1-\Phi)F_{CCBbAA}(i-1)+M_{CCbbAA}(i-$
$1)\Phi F_{CCBbAA}(i-1)+0.5M_{CCbbAA}(i-1)(1-\Phi)F_{CCBBAa}(i-1)+M_{CCbbAA}(i-1)\Phi F_{CCBBAa}(i-1)+$
$M_{CCbbAA}(i-1)F_{CCBBAA}(i-1)+0.0625(1-\Phi)(1-\Phi)M_{CCBbAa}(i-1)F_{CcbbAa}(i-1)+0.125(1-\Phi)(1-$
$\Phi)M_{CCBbAa}(i-1)F_{CcbbAA}(i-1)+0.0625(1-\Phi)(1-\Phi)M_{CCBbAa}(i-1)(1-\Phi)(1-\Phi)F_{CcBbAa}(i-$
$1)+0.0625(1-\Phi)(1-\Phi)M_{CCBbAa}(i-1)\Phi(1-\Phi)F_{CcBbAa}(i-1)+0.125(1-\Phi)(1-\Phi)M_{CCBbAa}(i-$
$1)(1-\Phi)\Phi F_{CcBbAa}(i-1)+0.125(1-\Phi)(1-\Phi)M_{CCBbAa}(i-1)\Phi\Phi F_{CcBbAa}(i-1)+0.125(1-\Phi)(1-$
$\Phi)M_{CCBbAa}(i-1)(1-\Phi)F_{CcBbAA}(i-1)+0.125(1-\Phi)(1-\Phi)M_{CCBbAa}(i-1)\Phi F_{CcBbAA}(i-1)+0.0625(1-$
$\Phi)(1-\Phi)M_{CCBbAa}(i-1)(1-\Phi)F_{CcBBAa}(i-1)+0.125(1-\Phi)(1-\Phi)M_{CCBbAa}(i-1)\Phi F_{CcBBAa}(i-1)+$
$0.125(1-\Phi)(1-\Phi)M_{CCBbAa}(i-1)F_{CcBBAA}(i-1)+0.125(1-\Phi)(1-\Phi)M_{CCBbAa}(i-1)F_{CCbbAa}(i-$
$1)+0.25(1-\Phi)(1-\Phi)M_{CCBbAa}(i-1)F_{CCbbAA}(i-1)+0.125(1-\Phi)(1-\Phi)M_{CCBbAa}(i-1)(1-\Phi)(1-$
$\Phi)F_{CCBbAa}(i-1)+0.125(1-\Phi)(1-\Phi)M_{CCBbAa}(i-1)\Phi(1-\Phi)F_{CCBbAa}(i-1)+0.125(1-\Phi)(1-$
$\Phi)M_{CCBbAa}(i-1)(1-\Phi)\Phi F_{CCBbAa}(i-1)+0.25(1-\Phi)(1-\Phi)M_{CCBbAa}(i-1)\Phi\Phi F_{CCBbAa}(i-1)+0.25(1-$
$\Phi)(1-\Phi)M_{CCBbAa}(i-1)(1-\Phi)F_{CCBbAA}(i-1)+0.25(1-\Phi)(1-\Phi)M_{CCBbAa}(i-1)\Phi F_{CCBbAA}(i-1)+$
$0.125(1-\Phi)(1-\Phi)M_{CCBbAa}(i-1)(1-\Phi)F_{CCBBAa}(i-1)+0.25(1-\Phi)(1-\Phi)M_{CCBbAa}(i-1)\Phi F_{CCBBAa}(i-$
$1)+0.25(1-\Phi)(1-\Phi)M_{CCBbAa}(i-1)F_{CCBBAA}(i-1)+0.125\Phi(1-\Phi)M_{CCBbAa}(i-1)F_{CcbbAa}(i-1)+$
$0.25\Phi(1-\Phi)M_{CCBbAa}(i-1)F_{CcbbAA}(i-1)+0.0625\Phi(1-\Phi)M_{CCBbAa}(i-1)(1-\Phi)(1-\Phi)F_{CcBbAa}(i-1)+$
$0.125\Phi(1-\Phi)M_{CCBbAa}(i-1)(1-\Phi)\Phi F_{CcBbAa}(i-1)+0.125\Phi(1-\Phi)M_{CCBbAa}(i-1)(1-\Phi)F_{CcBbAA}(i-$
$1)+0.25\Phi(1-\Phi)M_{CCBbAa}(i-1)F_{CCbbAa}(i-1)+0.5\Phi(1-\Phi)M_{CCBbAa}(i-1)F_{CCbbAA}(i-1)+0.125\Phi(1-$
$\Phi)M_{CCBbAa}(i-1)(1-\Phi)(1-\Phi)F_{CCBbAa}(i-1)+0.25\Phi(1-\Phi)M_{CCBbAa}(i-1)(1-\Phi)F_{CCBbAA}(i-1)+$
$0.125(1-\Phi)\Phi M_{CCBbAa}(i-1)F_{CcbbAa}(i-1)+0.25(1-\Phi)\Phi M_{CCBbAa}(i-1)F_{CcbbAA}(i-1)+0.125(1-$
$\Phi)\Phi M_{CCBbAa}(i-1)(1-\Phi)(1-\Phi)F_{CcBbAa}(i-1)+0.125(1-\Phi)\Phi M_{CCBbAa}(i-1)\Phi(1-\Phi)F_{CcBbAa}(i-$
$1)+0.25(1-\Phi)\Phi M_{CCBbAa}(i-1)(1-\Phi)\Phi F_{CcBbAa}(i-1)+0.25(1-\Phi)\Phi M_{CCBbAa}(i-1)\Phi\Phi F_{CcBbAa}(i-$
$1)+0.25(1-\Phi)\Phi M_{CCBbAa}(i-1)(1-\Phi)F_{CcBbAA}(i-1)+0.25(1-\Phi)\Phi M_{CCBbAa}(i-1)\Phi F_{CcBbAA}(i-1)+$
$0.125(1-\Phi)\Phi M_{CCBbAa}(i-1)(1-\Phi)F_{CcBBAa}(i-1)+0.25(1-\Phi)\Phi M_{CCBbAa}(i-1)\Phi F_{CcBBAa}(i-1)+$
$0.25(1-\Phi)\Phi M_{CCBbAa}(i-1)F_{CcBBAA}(i-1)+0.25(1-\Phi)\Phi M_{CCBbAa}(i-1)F_{CCbbAa}(i-1)+0.5(1-$
$\Phi)\Phi M_{CCBbAa}(i-1)F_{CCbbAA}(i-1)+0.25(1-\Phi)\Phi M_{CCBbAa}(i-1)(1-\Phi)(1-\Phi)F_{CCBbAa}(i-1)+$
$0.25(1-\Phi)\Phi M_{CCBbAa}(i-1)\Phi(1-\Phi)F_{CCBbAa}(i-1)+0.25(1-\Phi)\Phi M_{CCBbAa}(i-1)(1-\Phi)\Phi F_{CCBbAa}(i-$
$1)+0.5(1-\Phi)\Phi M_{CCBbAa}(i-1)\Phi\Phi F_{CCBbAa}(i-1)+0.5(1-\Phi)\Phi M_{CCBbAa}(i-1)(1-\Phi)F_{CCBbAA}(i-$
$1)+0.5(1-\Phi)\Phi M_{CCBbAa}(i-1)\Phi F_{CCBbAA}(i-1)+0.25(1-\Phi)\Phi M_{CCBbAa}(i-1)(1-\Phi)F_{CCBBAa}(i-$
$1)+0.5(1-\Phi)\Phi M_{CCBbAa}(i-1)\Phi F_{CCBBAa}(i-1)+0.5(1-\Phi)\Phi M_{CCBbAa}(i-1)F_{CCBBAA}(i-1)+$
$0.25\Phi\Phi M_{CCBbAa}(i-1)F_{CcbbAa}(i-1)+0.5\Phi\Phi M_{CCBbAa}(i-1)F_{CcbbAA}(i-1)+0.125\Phi\Phi M_{CCBbAa}(i-$
$1)(1-\Phi)(1-\Phi)F_{CcBbAa}(i-1)+0.25\Phi\Phi M_{CCBbAa}(i-1)(1-\Phi)\Phi F_{CcBbAa}(i-1)+0.25\Phi\Phi M_{CCBbAa}(i-$
$1)(1-\Phi)F_{CcBbAA}(i-1)+0.5\Phi\Phi M_{CCBbAa}(i-1)F_{CCbbAa}(i-1)+\Phi\Phi M_{CCBbAa}(i-1)F_{CCbbAA}(i-1)+$
$0.25\Phi\Phi M_{CCBbAa}(i-1)(1-\Phi)(1-\Phi)F_{CCBbAa}(i-1)+0.5\Phi\Phi M_{CCBbAa}(i-1)(1-\Phi)F_{CCBbAA}(i-1)+$
$0.125(1-\Phi)M_{CCBbAA}(i-1)F_{CcbbAa}(i-1)+0.25(1-\Phi)M_{CCBbAA}(i-1)F_{CcbbAA}(i-1)+0.125(1-$
$\Phi)M_{CCBbAA}(i-1)(1-\Phi)(1-\Phi)F_{CcBbAa}(i-1)+0.125(1-\Phi)M_{CCBbAA}(i-1)\Phi(1-\Phi)F_{CcBbAa}(i-$
$1)+0.25(1-\Phi)M_{CCBbAA}(i-1)(1-\Phi)\Phi F_{CcBbAa}(i-1)+0.25(1-\Phi)M_{CCBbAA}(i-1)\Phi\Phi F_{CcBbAa}(i-$
$1)+0.25(1-\Phi)M_{CCBbAA}(i-1)(1-\Phi)F_{CcBbAA}(i-1)+0.25(1-\Phi)M_{CCBbAA}(i-1)\Phi F_{CcBbAA}(i-$
$1)+0.125(1-\Phi)M_{CCBbAA}(i-1)(1-\Phi)F_{CcBBAa}(i-1)+0.25(1-\Phi)M_{CCBbAA}(i-1)\Phi F_{CcBBAa}(i-$
$1)+0.25(1-\Phi)M_{CCBbAA}(i-1)F_{CcBBAA}(i-1)+0.25(1-\Phi)M_{CCBbAA}(i-1)F_{CCbbAa}(i-1)+0.5(1-$
$\Phi)M_{CCBbAA}(i-1)F_{CCbbAA}(i-1)+0.25(1-\Phi)M_{CCBbAA}(i-1)(1-\Phi)(1-\Phi)F_{CCBbAa}(i-1)+0.25(1-$

$$\Phi)M_{CCBbAA}(i-1)\Phi(1-\Phi)F_{CCBbAa}(i-1)+0.25(1-\Phi)M_{CCBbAA}(i-1)(1-\Phi)\Phi F_{CCBbAa}(i-1)+0.5(1-\Phi)M_{CCBbAA}(i-1)\Phi\Phi F_{CCBbAa}(i-1)+0.5(1-\Phi)M_{CCBbAA}(i-1)(1-\Phi)F_{CCBbAA}(i-1)+0.5(1-\Phi)M_{CCBbAA}(i-1)\Phi F_{CCBbAA}(i-1)+0.25(1-\Phi)M_{CCBbAA}(i-1)(1-\Phi)F_{CCBBAa}(i-1)+0.5(1-\Phi)M_{CCBbAA}(i-1)\Phi F_{CCBBAa}(i-1)+0.5(1-\Phi)M_{CCBbAA}(i-1)F_{CCBBAA}(i-1)+0.25\Phi M_{CCBbAA}(i-1)F_{CcbbAa}(i-1)+0.5\Phi M_{CCBbAA}(i-1)F_{CcbbAA}(i-1)+0.125\Phi M_{CCBbAA}(i-1)(1-\Phi)(1-\Phi)F_{CcBbAa}(i-1)+0.25\Phi M_{CCBbAA}(i-1)(1-\Phi)\Phi F_{CcBbAa}(i-1)+0.25\Phi M_{CCBbAA}(i-1)(1-\Phi)F_{CcBbAA}(i-1)+0.5\Phi M_{CCBbAA}(i-1)F_{CCbbAa}(i-1)+\Phi M_{CCBbAA}(i-1)F_{CCbbAA}(i-1)+0.25\Phi M_{CCBbAA}(i-1)(1-\Phi)(1-\Phi)F_{CCBbAa}(i-1)+0.5\Phi M_{CCBbAA}(i-1)(1-\Phi)F_{CCBbAA}(i-1)+0.125(1-\Phi)M_{CCBBAa}(i-1)F_{CcbbAa}(i-1)+0.25(1-\Phi)M_{CCBBAa}(i-1)F_{CcbbAA}(i-1)+0.0625(1-\Phi)M_{CCBBAa}(i-1)(1-\Phi)(1-\Phi)F_{CcBbAa}(i-1)+0.125(1-\Phi)M_{CCBBAa}(i-1)(1-\Phi)\Phi F_{CcBbAa}(i-1)+0.125(1-\Phi)M_{CCBBAa}(i-1)(1-\Phi)F_{CcBbAA}(i-1)+0.25(1-\Phi)M_{CCBBAa}(i-1)F_{CCbbAa}(i-1)+0.5(1-\Phi)M_{CCBBAa}(i-1)F_{CCbbAA}(i-1)+0.125(1-\Phi)M_{CCBBAa}(i-1)(1-\Phi)(1-\Phi)F_{CCBbAa}(i-1)+0.25(1-\Phi)M_{CCBBAa}(i-1)(1-\Phi)F_{CCBbAA}(i-1)+0.25\Phi M_{CCBBAa}(i-1)F_{CcbbAa}(i-1)+0.5\Phi M_{CCBBAa}(i-1)F_{CcbbAA}(i-1)+0.125\Phi M_{CCBBAa}(i-1)(1-\Phi)(1-\Phi)F_{CcBbAa}(i-1)+0.25\Phi M_{CCBBAa}(i-1)(1-\Phi)\Phi F_{CcBbAa}(i-1)+0.25\Phi M_{CCBBAa}(i-1)(1-\Phi)F_{CcBbAA}(i-1)+0.5\Phi M_{CCBBAa}(i-1)F_{CCbbAa}(i-1)+\Phi M_{CCBBAa}(i-1)F_{CCbbAA}(i-1)+0.25\Phi M_{CCBBAa}(i-1)(1-\Phi)(1-\Phi)F_{CCBbAa}(i-1)+0.5\Phi M_{CCBBAa}(i-1)(1-\Phi)F_{CCBbAA}(i-1)+0.25M_{CCBBAA}(i-1)F_{CcbbAa}(i-1)+0.5M_{CCBBAA}(i-1)F_{CcbbAA}(i-1)+0.125M_{CCBBAA}(i-1)(1-\Phi)(1-\Phi)F_{CcBbAa}(i-1)+0.25M_{CCBBAA}(i-1)(1-\Phi)\Phi F_{CcBbAa}(i-1)+0.25M_{CCBBAA}(i-1)(1-\Phi)F_{CcBbAA}(i-1)+0.5M_{CCBBAA}(i-1)F_{CCbbAa}(i-1)+M_{CCBBAA}(i-1)F_{CCbbAA}(i-1)+0.25M_{CCBBAA}(i-1)(1-\Phi)(1-\Phi)F_{CCBbAa}(i-1)+0.5M_{CCBBAA}(i-1)(1-\Phi)F_{CCBbAA}(i-1)],$$

$$\mathrm{M}^{e}_{CCBBaa}=\left(\frac{\bar{\Omega}_{CCBBaa}}{2}\right)[0.0625(1-\Phi)M_{CcBbaa}(i-1)(1-\Phi)F_{CcBbaa}(i-1)+0.125(1-\Phi)M_{CcBbaa}(i-1)\Phi F_{CcBbaa}(i-1)+0.03125(1-\Phi)M_{CcBbaa}(i-1)(1-\Phi)(1-\Phi)F_{CcBbAa}(i-1)+0.0625(1-\Phi)M_{CcBbaa}(i-1)\Phi(1-\Phi)F_{CcBbAa}(i-1)+0.125(1-\Phi)M_{CcBbaa}(i-1)F_{CcBBaa}(i-1)+0.0625(1-\Phi)M_{CcBbaa}(i-1)(1-\Phi)F_{CcBBAa}(i-1)+0.125(1-\Phi)M_{CcBbaa}(i-1)(1-\Phi)F_{CCBbaa}(i-1)+0.25(1-\Phi)M_{CcBbaa}(i-1)\Phi F_{CCBbaa}(i-1)+0.0625(1-\Phi)M_{CcBbaa}(i-1)(1-\Phi)(1-\Phi)F_{CCBbAa}(i-1)+0.125(1-\Phi)M_{CcBbaa}(i-1)\Phi(1-\Phi)F_{CCBbAa}(i-1)+0.125(1-\Phi)M_{CcBbaa}(i-1)(1-\Phi)\Phi F_{CCBbAa}(i-1)+0.25(1-\Phi)M_{CcBbaa}(i-1)F_{CCBBaa}(i-1)+0.125(1-\Phi)M_{CcBbaa}(i-1)(1-\Phi)F_{CCBBAa}(i-1)+0.125\Phi M_{CcBbaa}(i-1)(1-\Phi)F_{CcBbaa}(i-1)+0.25\Phi M_{CcBbaa}(i-1)\Phi F_{CcBbaa}(i-1)+0.0625\Phi M_{CcBbaa}(i-1)(1-\Phi)(1-\Phi)F_{CcBbAa}(i-1)+0.125\Phi M_{CcBbaa}(i-1)\Phi(1-\Phi)F_{CcBbAa}(i-1)+0.25\Phi M_{CcBbaa}(i-1)F_{CcBBaa}(i-1)+0.125\Phi M_{CcBbaa}(i-1)(1-\Phi)F_{CcBBAa}(i-1)+0.25\Phi M_{CcBbaa}(i-1)(1-\Phi)F_{CCBbaa}(i-1)+0.5\Phi M_{CcBbaa}(i-1)\Phi F_{CCBbaa}(i-1)+0.125\Phi M_{CcBbaa}(i-1)(1-\Phi)(1-\Phi)F_{CCBbAa}(i-1)+0.25\Phi M_{CcBbaa}(i-1)\Phi(1-\Phi)F_{CCBbAa}(i-1)+0.25\Phi M_{CcBbaa}(i-1)(1-\Phi)\Phi F_{CCBbAa}(i-1)+0.5\Phi M_{CcBbaa}(i-1)F_{CCBBaa}(i-1)+0.25\Phi M_{CcBbaa}(i-1)(1-\Phi)F_{CCBBAa}(i-1)+0.03125(1-\Phi)(1-\Phi)M_{CcBbAa}(i-1)(1-\Phi)F_{CcBbaa}(i-1)+0.0625(1-\Phi)(1-\Phi)M_{CcBbAa}(i-1)\Phi F_{CcBbaa}(i-1)+0.015625(1-\Phi)(1-\Phi)M_{CcBbAa}(i-1)(1-\Phi)(1-\Phi)F_{CcBbAa}(i-1)+0.03125(1-\Phi)(1-\Phi)M_{CcBbAa}(i-1)\Phi(1-\Phi)F_{CcBbAa}(i-1)+0.0625(1-\Phi)(1-\Phi)M_{CcBbAa}(i-1)F_{CcBBaa}(i-1)+0.03125(1-\Phi)(1-\Phi)M_{CcBbAa}(i-1)(1-\Phi)F_{CcBBAa}(i-1)+0.0625(1-\Phi)(1-\Phi)M_{CcBbAa}(i-1)(1-\Phi)F_{CCBbaa}(i-1)+0.125(1-\Phi)(1-\Phi)M_{CcBbAa}(i-1)\Phi F_{CCBbaa}(i-1)+0.03125(1-\Phi)(1-\Phi)M_{CcBbAa}(i-1)(1-\Phi)(1-\Phi)F_{CCBbAa}(i-1)+0.0625(1-\Phi)(1-\Phi)M_{CcBbAa}(i-1)\Phi(1-\Phi)F_{CCBbAa}(i-1)+0.0625(1-\Phi)(1-\Phi)M_{CcBbAa}(i-1)(1-\Phi)\Phi F_{CCBbAa}(i-1)+0.125(1-\Phi)(1-\Phi)M_{CcBbAa}(i-1)F_{CCBBaa}(i-1)+0.0625(1-\Phi)(1-\Phi)M_{CcBbAa}(i-1)(1-\Phi)F_{CCBBAa}(i-1)+0.0625\Phi(1-\Phi)M_{CcBbAa}(i-1)(1-\Phi)F_{CcBbaa}(i-1)+0.125\Phi(1-\Phi)M_{CcBbAa}(i-1)\Phi F_{CcBbaa}(i-1)+0.03125\Phi(1-\Phi)M_{CcBbAa}(i-1)(1-\Phi)(1-\Phi)F_{CcBbAa}(i-1)+0.0625\Phi(1-\Phi)M_{CcBbAa}(i-1)\Phi(1-\Phi)F_{CcBbAa}(i-1)+0.125\Phi(1-\Phi)M_{CcBbAa}(i-1)F_{CcBBaa}(i-1)+0.0625\Phi(1-\Phi)M_{CcBbAa}(i-1)(1-\Phi)F_{CcBBAa}(i-1)+0.125\Phi(1-\Phi)M_{CcBbAa}(i-1)(1-\Phi)F_{CCBbaa}(i-1)+0.25\Phi(1-\Phi)M_{CcBbAa}(i-1)\Phi F_{CCBbaa}(i-1)+0.0625\Phi(1-\Phi)M_{CcBbAa}(i-1)(1-\Phi)(1-\Phi)F_{CCBbAa}(i-1)+0.125\Phi(1-\Phi)M_{CcBbAa}(i-1)\Phi(1-\Phi)F_{CCBbAa}(i-1)+0.125\Phi(1-\Phi)M_{CcBbAa}(i-1)(1-\Phi)\Phi F_{CCBbAa}(i-1)+0.25\Phi(1-\Phi)M_{CcBbAa}(i-1)F_{CCBBaa}(i-1)+0.125\Phi(1-\Phi)M_{CcBbAa}(i-1)(1-\Phi)F_{CCBBAa}(i-1)+0.125M_{CcBBaa}(i-1)(1-\Phi)F_{CcBbaa}(i-1)+0.25M_{CcBBaa}(i-1)\Phi F_{CcBbaa}(i-1)+0.0625M_{CcBBaa}(i-1)(1-\Phi)(1-\Phi)F_{CcBbAa}(i-1)+0.125M_{CcBBaa}(i-1)\Phi(1-\Phi)F_{CcBbAa}(i-1)+0.25M_{CcBBaa}(i-1)F_{CcBBaa}(i-1)+0.125M_{CcBBaa}(i-1)(1-\Phi)F_{CcBBAa}(i-1)+0.25M_{CcBBaa}(i-1)(1-\Phi)F_{CCBbaa}(i-1)+0.5M_{CcBBaa}(i-1)\Phi F_{CCBbaa}(i-1)+0.125M_{CcBBaa}(i-1)(1-\Phi)(1-\Phi)F_{CCBbAa}(i-1)+0.25M_{CcBBaa}(i-1)\Phi(1-\Phi)F_{CCBbAa}(i-1)+0.25M_{CcBBaa}(i-1)(1-\Phi)\Phi F_{CCBbAa}(i-1)+0.5M_{CcBBaa}(i-1)F_{CCBBaa}(i-1)+0.25M_{CcBBaa}(i-1)(1-\Phi)F_{CCBBAa}(i-1)+0.0625(1-\Phi)M_{CcBBAa}(i-1)(1-\Phi)F_{CcBbaa}(i-1)+0.125(1-\Phi)M_{CcBBAa}(i-1)\Phi F_{CcBbaa}(i-1)+0.03125(1-\Phi)M_{CcBBAa}(i-1)(1-\Phi)(1-\Phi)F_{CcBbAa}(i-1)+0.0625(1-\Phi)M_{CcBBAa}(i-1)\Phi(1-\Phi)F_{CcBbAa}(i-1)+0.125(1-\Phi)M_{CcBBAa}(i-1)F_{CcBBaa}(i-1)+0.0625(1-\Phi)M_{CcBBAa}(i-1)(1-\Phi)F_{CcBBAa}(i-1)+0.125(1-\Phi)M_{CcBBAa}(i-1)(1-\Phi)F_{CCBbaa}(i-1)+0.25(1-\Phi)M_{CcBBAa}(i-1)\Phi F_{CCBbaa}(i-1)+0.0625(1-\Phi)M_{CcBBAa}(i-1)(1-\Phi)(1-\Phi)F_{CCBbAa}(i-1)+$$

$0.125(1-\Phi)M_{CcBBAa}(i-1)\Phi(1-\Phi)F_{CCBbAa}(i-1)+0.125(1-\Phi)M_{CcBBAa}(i-1)(1-\Phi)\Phi F_{CCBbAa}(i-$
$1)+0.25(1-\Phi)M_{CcBBAa}(i-1)F_{CCBBaa}(i-1)+0.125(1-\Phi)M_{CcBBAa}(i-1)(1-\Phi)F_{CCBBAa}(i-$
$1)+0.125(1-\Phi)M_{CCBbaa}(i-1)(1-\Phi)F_{CcBbaa}(i-1)+0.25(1-\Phi)M_{CCBbaa}(i-1)\Phi F_{CcBbaa}(i-1)+$
$0.0625(1-\Phi)M_{CCBbaa}(i-1)(1-\Phi)(1-\Phi)F_{CcBbAa}(i-1)+0.125(1-\Phi)M_{CCBbaa}(i-1)\Phi(1-\Phi)F_{CcBbAa}(i-$
$1)+0.25(1-\Phi)M_{CCBbaa}(i-1)F_{CcBBaa}(i-1)+0.125(1-\Phi)M_{CCBbaa}(i-1)(1-\Phi)F_{CcBBAa}(i-1)+$
$0.25(1-\Phi)M_{CCBbaa}(i-1)(1-\Phi)F_{CCBbaa}(i-1)+0.5(1-\Phi)M_{CCBbaa}(i-1)\Phi F_{CCBbaa}(i-1)+0.125(1-$
$\Phi)M_{CCBbaa}(i-1)(1-\Phi)(1-\Phi)F_{CCBbAa}(i-1)+0.25(1-\Phi)M_{CCBbaa}(i-1)\Phi(1-\Phi)F_{CCBbAa}(i-1)+$
$0.25(1-\Phi)M_{CCBbaa}(i-1)(1-\Phi)\Phi F_{CCBbAa}(i-1)+0.5(1-\Phi)M_{CCBbaa}(i-1)F_{CCBBaa}(i-1)+0.25(1-$
$\Phi)M_{CCBbaa}(i-1)(1-\Phi)F_{CCBBAa}(i-1)+0.25\Phi M_{CCBbaa}(i-1)(1-\Phi)F_{CcBbaa}(i-1)+0.5\Phi M_{CCBbaa}(i-$
$1)\Phi F_{CcBbaa}(i-1)+0.125\Phi M_{CCBbaa}(i-1)(1-\Phi)(1-\Phi)F_{CcBbAa}(i-1)+0.25\Phi M_{CCBbaa}(i-1)\Phi(1-$
$\Phi)F_{CcBbAa}(i-1)+0.5\Phi M_{CCBbaa}(i-1)F_{CcBBaa}(i-1)+0.25\Phi M_{CCBbaa}(i-1)(1-\Phi)F_{CcBBAa}(i-1)+$
$0.5\Phi M_{CCBbaa}(i-1)(1-\Phi)F_{CCBbaa}(i-1)+\Phi M_{CCBbaa}(i-1)\Phi F_{CCBbaa}(i-1)+0.25\Phi M_{CCBbaa}(i-$
$1)(1-\Phi)(1-\Phi)F_{CCBbAa}(i-1)+0.5\Phi M_{CCBbaa}(i-1)\Phi(1-\Phi)F_{CCBbAa}(i-1)+0.5\Phi M_{CCBbaa}(i-$
$1)(1-\Phi)\Phi F_{CCBbAa}(i-1)+\Phi M_{CCBbaa}(i-1)F_{CCBBaa}(i-1)+0.5\Phi M_{CCBbaa}(i-1)(1-\Phi)F_{CCBBAa}(i-$
$1)+0.0625(1-\Phi)(1-\Phi)M_{CCBbAa}(i-1)(1-\Phi)F_{CcBbaa}(i-1)+0.125(1-\Phi)(1-\Phi)M_{CCBbAa}(i-$
$1)\Phi F_{CcBbaa}(i-1)+0.03125(1-\Phi)(1-\Phi)M_{CCBbAa}(i-1)(1-\Phi)(1-\Phi)F_{CcBbAa}(i-1)+0.0625(1-\Phi)(1-$
$\Phi)M_{CCBbAa}(i-1)\Phi(1-\Phi)F_{CcBbAa}(i-1)+0.125(1-\Phi)(1-\Phi)M_{CCBbAa}(i-1)F_{CcBBaa}(i-1)+0.0625(1-$
$\Phi)(1-\Phi)M_{CCBbAa}(i-1)(1-\Phi)F_{CcBBAa}(i-1)+0.125(1-\Phi)(1-\Phi)M_{CCBbAa}(i-1)(1-\Phi)F_{CCBbaa}(i-$
$1)+0.25(1-\Phi)(1-\Phi)M_{CCBbAa}(i-1)\Phi F_{CCBbaa}(i-1)+0.0625(1-\Phi)(1-\Phi)M_{CCBbAa}(i-1)(1-\Phi)(1-$
$\Phi)F_{CCBbAa}(i-1)+0.125(1-\Phi)(1-\Phi)M_{CCBbAa}(i-1)\Phi(1-\Phi)F_{CCBbAa}(i-1)+0.125(1-\Phi)(1-$
$\Phi)M_{CCBbAa}(i-1)(1-\Phi)\Phi F_{CCBbAa}(i-1)+0.25(1-\Phi)(1-\Phi)M_{CCBbAa}(i-1)F_{CCBBaa}(i-1)+0.125(1-$
$\Phi)(1-\Phi)M_{CCBbAa}(i-1)(1-\Phi)F_{CCBBAa}(i-1)+0.125\Phi(1-\Phi)M_{CCBbAa}(i-1)(1-\Phi)F_{CcBbaa}(i-1)+$
$0.25\Phi(1-\Phi)M_{CCBbAa}(i-1)\Phi F_{CcBbaa}(i-1)+0.0625\Phi(1-\Phi)M_{CCBbAa}(i-1)(1-\Phi)(1-\Phi)F_{CcBbAa}(i-$
$1)+0.125\Phi(1-\Phi)M_{CCBbAa}(i-1)\Phi(1-\Phi)F_{CcBbAa}(i-1)+0.25\Phi(1-\Phi)M_{CCBbAa}(i-1)F_{CcBBaa}(i-1)+$
$0.125\Phi(1-\Phi)M_{CCBbAa}(i-1)(1-\Phi)F_{CcBBAa}(i-1)+0.25\Phi(1-\Phi)M_{CCBbAa}(i-1)(1-\Phi)F_{CCBbaa}(i-1)+$
$0.5\Phi(1-\Phi)M_{CCBbAa}(i-1)\Phi F_{CCBbaa}(i-1)+0.125\Phi(1-\Phi)M_{CCBbAa}(i-1)(1-\Phi)(1-\Phi)F_{CCBbAa}(i-1)+$
$0.25\Phi(1-\Phi)M_{CCBbAa}(i-1)\Phi(1-\Phi)F_{CCBbAa}(i-1)+0.25\Phi(1-\Phi)M_{CCBbAa}(i-1)(1-\Phi)\Phi F_{CCBbAa}(i-$
$1)+0.5\Phi(1-\Phi)M_{CCBbAa}(i-1)F_{CCBBaa}(i-1)+0.25\Phi(1-\Phi)M_{CCBbAa}(i-1)(1-\Phi)F_{CCBBAa}(i-1)+$
$0.25M_{CCBBaa}(i-1)(1-\Phi)F_{CcBbaa}(i-1)+0.5M_{CCBBaa}(i-1)\Phi F_{CcBbaa}(i-1)+0.125M_{CCBBaa}(i-1)(1-$
$\Phi)(1-\Phi)F_{CcBbAa}(i-1)+0.25M_{CCBBaa}(i-1)\Phi(1-\Phi)F_{CcBbAa}(i-1)+0.5M_{CCBBaa}(i-1)F_{CcBBaa}(i-1)+$
$0.25M_{CCBBaa}(i-1)(1-\Phi)F_{CcBBAa}(i-1)+0.5M_{CCBBaa}(i-1)(1-\Phi)F_{CCBbaa}(i-1)+M_{CCBBaa}(i-$
$1)\Phi F_{CCBbaa}(i-1)+0.25M_{CCBBaa}(i-1)(1-\Phi)(1-\Phi)F_{CCBbAa}(i-1)+0.5M_{CCBBaa}(i-1)\Phi(1-$
$\Phi)F_{CCBbAa}(i-1)+0.5M_{CCBBaa}(i-1)(1-\Phi)\Phi F_{CCBbAa}(i-1)+M_{CCBBaa}(i-1)F_{CCBBaa}(i-1)+$
$0.5M_{CCBBaa}(i-1)(1-\Phi)F_{CCBBAa}(i-1)+0.125(1-\Phi)M_{CCBBAa}(i-1)(1-\Phi)F_{CcBbaa}(i-1)+0.25(1-$
$\Phi)M_{CCBBAa}(i-1)\Phi F_{CcBbaa}(i-1)+0.0625(1-\Phi)M_{CCBBAa}(i-1)(1-\Phi)(1-\Phi)F_{CcBbAa}(i-1)+$
$0.125(1-\Phi)M_{CCBBAa}(i-1)\Phi(1-\Phi)F_{CcBbAa}(i-1)+0.25(1-\Phi)M_{CCBBAa}(i-1)F_{CcBBaa}(i-1)+$
$0.125(1-\Phi)M_{CCBBAa}(i-1)(1-\Phi)F_{CcBBAa}(i-1)+0.25(1-\Phi)M_{CCBBAa}(i-1)(1-\Phi)F_{CCBbaa}(i-1)+$
$0.5(1-\Phi)M_{CCBBAa}(i-1)\Phi F_{CCBbaa}(i-1)+0.125(1-\Phi)M_{CCBBAa}(i-1)(1-\Phi)(1-\Phi)F_{CCBbAa}(i-1)+$
$0.25(1-\Phi)M_{CCBBAa}(i-1)\Phi(1-\Phi)F_{CCBbAa}(i-1)+0.25(1-\Phi)M_{CCBBAa}(i-1)(1-\Phi)\Phi F_{CCBbAa}(i-$
$1)+0.5(1-\Phi)M_{CCBBAa}(i-1)F_{CCBBaa}(i-1)+0.25(1-\Phi)M_{CCBBAa}(i-1)(1-\Phi)F_{CCBBAa}(i-1)],$

$\mathrm{M}^{e}_{CCBBAa}=\left(\frac{\bar{\Omega}_{CCBBAa}}{2}\right)[0.03125(1-\Phi)M_{CcBbaa}(i-1)(1-\Phi)(1-\Phi)F_{CcBbAa}(i-1)+0.0625(1-$
$\Phi)M_{CcBbaa}(i-1)\Phi(1-\Phi)F_{CcBbAa}(i-1)+0.0625(1-\Phi)M_{CcBbaa}(i-1)(1-\Phi)\Phi F_{CcBbAa}(i-1)+$
$0.125(1-\Phi)M_{CcBbaa}(i-1)\Phi\Phi F_{CcBbAa}(i-1)+0.0625(1-\Phi)M_{CcBbaa}(i-1)(1-\Phi)F_{CcBbAA}(i-1)+$
$0.125(1-\Phi)M_{CcBbaa}(i-1)\Phi F_{CcBbAA}(i-1)+0.0625(1-\Phi)M_{CcBbaa}(i-1)(1-\Phi)F_{CcBBAa}(i-1)+$
$0.125(1-\Phi)M_{CcBbaa}(i-1)\Phi F_{CcBBAa}(i-1)+0.125(1-\Phi)M_{CcBbaa}(i-1)F_{CcBBAA}(i-1)+0.0625(1-$
$\Phi)M_{CcBbaa}(i-1)(1-\Phi)(1-\Phi)F_{CCBbAa}(i-1)+0.125(1-\Phi)M_{CcBbaa}(i-1)\Phi(1-\Phi)F_{CCBbAa}(i-$
$1)+0.125(1-\Phi)M_{CcBbaa}(i-1)(1-\Phi)\Phi F_{CCBbAa}(i-1)+0.25(1-\Phi)M_{CcBbaa}(i-1)\Phi\Phi F_{CCBbAa}(i-$
$1)+0.125(1-\Phi)M_{CcBbaa}(i-1)(1-\Phi)F_{CCBbAA}(i-1)+0.25(1-\Phi)M_{CcBbaa}(i-1)\Phi F_{CCBbAA}(i-$
$1)+0.125(1-\Phi)M_{CcBbaa}(i-1)(1-\Phi)F_{CCBBAa}(i-1)+0.25(1-\Phi)M_{CcBbaa}(i-1)\Phi F_{CCBBAa}(i-$
$1)+0.25(1-\Phi)M_{CcBbaa}(i-1)F_{CCBBAA}(i-1)+0.0625\Phi M_{CcBbaa}(i-1)(1-\Phi)(1-\Phi)F_{CcBbAa}(i-$
$1)+0.125\Phi M_{CcBbaa}(i-1)\Phi(1-\Phi)F_{CcBbAa}(i-1)+0.125\Phi M_{CcBbaa}(i-1)(1-\Phi)\Phi F_{CcBbAa}(i-1)+$
$0.25\Phi M_{CcBbaa}(i-1)\Phi\Phi F_{CcBbAa}(i-1)+0.125\Phi M_{CcBbaa}(i-1)(1-\Phi)F_{CcBbAA}(i-1)+0.25\Phi M_{CcBbaa}(i-$
$1)\Phi F_{CcBbAA}(i-1)+0.125\Phi M_{CcBbaa}(i-1)(1-\Phi)F_{CcBBAa}(i-1)+0.25\Phi M_{CcBbaa}(i-1)\Phi F_{CcBBAa}(i-1)+$
$0.25\Phi M_{CcBbaa}(i-1)F_{CcBBAA}(i-1)+0.125\Phi M_{CcBbaa}(i-1)(1-\Phi)(1-\Phi)F_{CCBbAa}(i-1)+0.25\Phi M_{CcBbaa}(i-$
$1)\Phi(1-\Phi)F_{CCBbAa}(i-1)+0.25\Phi M_{CcBbaa}(i-1)(1-\Phi)\Phi F_{CCBbAa}(i-1)+0.5\Phi M_{CcBbaa}(i-1)\Phi\Phi F_{CCBbAa}(i-$
$1)+0.25\Phi M_{CcBbaa}(i-1)(1-\Phi)F_{CCBbAA}(i-1)+0.5\Phi M_{CcBbaa}(i-1)\Phi F_{CCBbAA}(i-1)+0.25\Phi M_{CcBbaa}(i-$

$1)(1-\Phi)F_{CCBBAa}(i-1)+0.5\Phi M_{CcBbaa}(i-1)\Phi F_{CCBBAa}(i-1)+0.5\Phi M_{CcBbaa}(i-1)F_{CCBBAA}(i-$
$1)+0.03125(1-\Phi)(1-\Phi)M_{CcBbAa}(i-1)(1-\Phi)F_{CcBbaa}(i-1)+0.0625(1-\Phi)(1-\Phi)M_{CcBbAa}(i-$
$1)\Phi F_{CcBbaa}(i-1)+0.03125(1-\Phi)(1-\Phi)M_{CcBbAa}(i-1)(1-\Phi)(1-\Phi)F_{CcBbAa}(i-1)+0.0625(1-\Phi)(1-$
$\Phi)M_{CcBbAa}(i-1)\Phi(1-\Phi)F_{CcBbAa}(i-1)+0.03125(1-\Phi)(1-\Phi)M_{CcBbAa}(i-1)(1-\Phi)\Phi F_{CcBbAa}(i-$
$1)+0.0625(1-\Phi)(1-\Phi)M_{CcBbAa}(i-1)\Phi\Phi F_{CcBbAa}(i-1)+0.03125(1-\Phi)(1-\Phi)M_{CcBbAa}(i-1)(1-$
$\Phi)F_{CcBbAA}(i-1)+0.0625(1-\Phi)(1-\Phi)M_{CcBbAa}(i-1)\Phi F_{CcBbAA}(i-1)+0.0625(1-\Phi)(1-\Phi)M_{CcBbAa}(i-$
$1)F_{CcBBaa}(i-1)+0.0625(1-\Phi)(1-\Phi)M_{CcBbAa}(i-1)(1-\Phi)F_{CcBBAa}(i-1)+0.0625(1-\Phi)(1-$
$\Phi)M_{CcBbAa}(i-1)\Phi F_{CcBBAa}(i-1)+0.0625(1-\Phi)(1-\Phi)M_{CcBbAa}(i-1)F_{CcBBAA}(i-1)+0.0625(1-$
$\Phi)(1-\Phi)M_{CcBbAa}(i-1)(1-\Phi)F_{CCBbaa}(i-1)+0.125(1-\Phi)(1-\Phi)M_{CcBbAa}(i-1)\Phi F_{CCBbaa}(i-1)+$
$0.0625(1-\Phi)(1-\Phi)M_{CcBbAa}(i-1)(1-\Phi)(1-\Phi)F_{CCBbAa}(i-1)+0.125(1-\Phi)(1-\Phi)M_{CcBbAa}(i-$
$1)\Phi(1-\Phi)F_{CCBbAa}(i-1)+0.125(1-\Phi)(1-\Phi)M_{CcBbAa}(i-1)(1-\Phi)\Phi F_{CCBbAa}(i-1)+0.125(1-\Phi)(1-$
$\Phi)M_{CcBbAa}(i-1)\Phi\Phi F_{CCBbAa}(i-1)+0.0625(1-\Phi)(1-\Phi)M_{CcBbAa}(i-1)(1-\Phi)F_{CCBbAA}(i-1)+$
$0.125(1-\Phi)(1-\Phi)M_{CcBbAa}(i-1)\Phi F_{CCBbAA}(i-1)+0.125(1-\Phi)(1-\Phi)M_{CcBbAa}(i-1)F_{CCBBaa}(i-$
$1)+0.125(1-\Phi)(1-\Phi)M_{CcBbAa}(i-1)(1-\Phi)F_{CCBBAa}(i-1)+0.125(1-\Phi)(1-\Phi)M_{CcBbAa}(i-$
$1)\Phi F_{CCBBAa}(i-1)+0.125(1-\Phi)(1-\Phi)M_{CcBbAa}(i-1)F_{CCBBAA}(i-1)+0.0625\Phi(1-\Phi)M_{CcBbAa}(i-$
$1)(1-\Phi)F_{CcBbaa}(i-1)+0.125\Phi(1-\Phi)M_{CcBbAa}(i-1)\Phi F_{CcBbaa}(i-1)+0.0625\Phi(1-\Phi)M_{CcBbAa}(i-1)(1-$
$\Phi)(1-\Phi)F_{CcBbAa}(i-1)+0.125\Phi(1-\Phi)M_{CcBbAa}(i-1)\Phi(1-\Phi)F_{CcBbAa}(i-1)+0.0625\Phi(1-\Phi)M_{CcBbAa}(i-$
$1)(1-\Phi)\Phi F_{CcBbAa}(i-1)+0.125\Phi(1-\Phi)M_{CcBbAa}(i-1)\Phi\Phi F_{CcBbAa}(i-1)+0.0625\Phi(1-\Phi)M_{CcBbAa}(i-$
$1)(1-\Phi)F_{CcBbAA}(i-1)+0.125\Phi(1-\Phi)M_{CcBbAa}(i-1)\Phi F_{CcBbAA}(i-1)+0.125\Phi(1-\Phi)M_{CcBbAa}(i-$
$1)F_{CcBBaa}(i-1)+0.125\Phi(1-\Phi)M_{CcBbAa}(i-1)(1-\Phi)F_{CcBBAa}(i-1)+0.125\Phi(1-\Phi)M_{CcBbAa}(i-$
$1)\Phi F_{CcBBAa}(i-1)+0.125\Phi(1-\Phi)M_{CcBbAa}(i-1)F_{CcBBAA}(i-1)+0.125\Phi(1-\Phi)M_{CcBbAa}(i-1)(1-$
$\Phi)F_{CCBbaa}(i-1)+0.25\Phi(1-\Phi)M_{CcBbAa}(i-1)\Phi F_{CCBbaa}(i-1)+0.125\Phi(1-\Phi)M_{CcBbAa}(i-1)(1-\Phi)(1-$
$\Phi)F_{CCBbAa}(i-1)+0.25\Phi(1-\Phi)M_{CcBbAa}(i-1)\Phi(1-\Phi)F_{CCBbAa}(i-1)+0.25\Phi(1-\Phi)M_{CcBbAa}(i-$
$1)(1-\Phi)\Phi F_{CCBbAa}(i-1)+0.25\Phi(1-\Phi)M_{CcBbAa}(i-1)\Phi\Phi F_{CCBbAa}(i-1)+0.125\Phi(1-\Phi)M_{CcBbAa}(i-$
$1)(1-\Phi)F_{CCBbAA}(i-1)+0.25\Phi(1-\Phi)M_{CcBbAa}(i-1)\Phi F_{CCBbAA}(i-1)+0.25\Phi(1-\Phi)M_{CcBbAa}(i-$
$1)F_{CCBBaa}(i-1)+0.25\Phi(1-\Phi)M_{CcBbAa}(i-1)(1-\Phi)F_{CCBBAa}(i-1)+0.25\Phi(1-\Phi)M_{CcBbAa}(i-$
$1)\Phi F_{CCBBAa}(i-1)+0.25\Phi(1-\Phi)M_{CcBbAa}(i-1)F_{CCBBAA}(i-1)+0.0625(1-\Phi)\Phi M_{CcBbAa}(i-1)(1-$
$\Phi)F_{CcBbaa}(i-1)+0.125(1-\Phi)\Phi M_{CcBbAa}(i-1)\Phi F_{CcBbaa}(i-1)+0.03125(1-\Phi)\Phi M_{CcBbAa}(i-1)(1-\Phi)(1-$
$\Phi)F_{CcBbAa}(i-1)+0.0625(1-\Phi)\Phi M_{CcBbAa}(i-1)\Phi(1-\Phi)F_{CcBbAa}(i-1)+0.125(1-\Phi)\Phi M_{CcBbAa}(i-$
$1)F_{CcBBaa}(i-1)+0.0625(1-\Phi)\Phi M_{CcBbAa}(i-1)(1-\Phi)F_{CcBBAa}(i-1)+0.125(1-\Phi)\Phi M_{CcBbAa}(i-1)(1-$
$\Phi)F_{CCBbaa}(i-1)+0.25(1-\Phi)\Phi M_{CcBbAa}(i-1)\Phi F_{CCBbaa}(i-1)+0.0625(1-\Phi)\Phi M_{CcBbAa}(i-1)(1-\Phi)(1-$
$\Phi)F_{CCBbAa}(i-1)+0.125(1-\Phi)\Phi M_{CcBbAa}(i-1)\Phi(1-\Phi)F_{CCBbAa}(i-1)+0.125(1-\Phi)\Phi M_{CcBbAa}(i-$
$1)(1-\Phi)\Phi F_{CCBbAa}(i-1)+0.25(1-\Phi)\Phi M_{CcBbAa}(i-1)F_{CCBBaa}(i-1)+0.125(1-\Phi)\Phi M_{CcBbAa}(i-1)(1-$
$\Phi)F_{CCBBAa}(i-1)+0.125\Phi\Phi M_{CcBbAa}(i-1)(1-\Phi)F_{CcBbaa}(i-1)+0.25\Phi\Phi M_{CcBbAa}(i-1)\Phi F_{CcBbaa}(i-1)+$
$0.0625\Phi\Phi M_{CcBbAa}(i-1)(1-\Phi)(1-\Phi)F_{CcBbAa}(i-1)+0.125\Phi\Phi M_{CcBbAa}(i-1)\Phi(1-\Phi)F_{CcBbAa}(i-1)+$
$0.25\Phi\Phi M_{CcBbAa}(i-1)F_{CcBBaa}(i-1)+0.125\Phi\Phi M_{CcBbAa}(i-1)(1-\Phi)F_{CcBBAa}(i-1)+0.25\Phi\Phi M_{CcBbAa}(i-$
$1)(1-\Phi)F_{CCBbaa}(i-1)+0.5\Phi\Phi M_{CcBbAa}(i-1)\Phi F_{CCBbaa}(i-1)+0.125\Phi\Phi M_{CcBbAa}(i-1)(1-\Phi)(1-$
$\Phi)F_{CCBbAa}(i-1)+0.25\Phi\Phi M_{CcBbAa}(i-1)\Phi(1-\Phi)F_{CCBbAa}(i-1)+0.25\Phi\Phi M_{CcBbAa}(i-1)(1-\Phi)\Phi F_{CCBbAa}(i-$
$1)+0.5\Phi\Phi M_{CcBbAa}(i-1)F_{CCBBaa}(i-1)+0.25\Phi\Phi M_{CcBbAa}(i-1)(1-\Phi)F_{CCBBAa}(i-1)+0.0625(1-$
$\Phi)M_{CcBbAA}(i-1)(1-\Phi)F_{CcBbaa}(i-1)+0.125(1-\Phi)M_{CcBbAA}(i-1)\Phi F_{CcBbaa}(i-1)+0.03125(1-$
$\Phi)M_{CcBbAA}(i-1)(1-\Phi)(1-\Phi)F_{CcBbAa}(i-1)+0.0625(1-\Phi)M_{CcBbAA}(i-1)\Phi(1-\Phi)F_{CcBbAa}(i-$
$1)+0.125(1-\Phi)M_{CcBbAA}(i-1)F_{CcBBaa}(i-1)+0.0625(1-\Phi)M_{CcBbAA}(i-1)(1-\Phi)F_{CcBBAa}(i-1)+$
$0.125(1-\Phi)M_{CcBbAA}(i-1)(1-\Phi)F_{CCBbaa}(i-1)+0.25(1-\Phi)M_{CcBbAA}(i-1)\Phi F_{CCBbaa}(i-1)+0.0625(1-$
$\Phi)M_{CcBbAA}(i-1)(1-\Phi)(1-\Phi)F_{CCBbAa}(i-1)+0.125(1-\Phi)M_{CcBbAA}(i-1)\Phi(1-\Phi)F_{CCBbAa}(i-$
$1)+0.125(1-\Phi)M_{CcBbAA}(i-1)(1-\Phi)\Phi F_{CCBbAa}(i-1)+0.25(1-\Phi)M_{CcBbAA}(i-1)F_{CCBBaa}(i-$
$1)+0.125(1-\Phi)M_{CcBbAA}(i-1)(1-\Phi)F_{CCBBAa}(i-1)+0.125\Phi M_{CcBbAA}(i-1)(1-\Phi)F_{CcBbaa}(i-$
$1)+0.25\Phi M_{CcBbAA}(i-1)\Phi F_{CcBbaa}(i-1)+0.0625\Phi M_{CcBbAA}(i-1)(1-\Phi)(1-\Phi)F_{CcBbAa}(i-1)+$
$0.125\Phi M_{CcBbAA}(i-1)\Phi(1-\Phi)F_{CcBbAa}(i-1)+0.25\Phi M_{CcBbAA}(i-1)F_{CcBBaa}(i-1)+0.125\Phi M_{CcBbAA}(i-$
$1)(1-\Phi)F_{CcBBAa}(i-1)+0.25\Phi M_{CcBbAA}(i-1)(1-\Phi)F_{CCBbaa}(i-1)+0.5\Phi M_{CcBbAA}(i-1)\Phi F_{CCBbaa}(i-$
$1)+0.125\Phi M_{CcBbAA}(i-1)(1-\Phi)(1-\Phi)F_{CCBbAa}(i-1)+0.25\Phi M_{CcBbAA}(i-1)\Phi(1-\Phi)F_{CCBbAa}(i-1)+$
$0.25\Phi M_{CcBbAA}(i-1)(1-\Phi)\Phi F_{CCBbAa}(i-1)+0.5\Phi M_{CcBbAA}(i-1)F_{CCBBaa}(i-1)+0.25\Phi M_{CcBbAA}(i-$
$1)(1-\Phi)F_{CCBBAa}(i-1)+0.0625M_{CcBBaa}(i-1)(1-\Phi)(1-\Phi)F_{CcBbAa}(i-1)+0.125M_{CcBBaa}(i-1)\Phi(1-$
$\Phi)F_{CcBbAa}(i-1)+0.125M_{CcBBaa}(i-1)(1-\Phi)\Phi F_{CcBbAa}(i-1)+0.25M_{CcBBaa}(i-1)\Phi\Phi F_{CcBbAa}(i-1)+$
$0.125M_{CcBBaa}(i-1)(1-\Phi)F_{CcBbAA}(i-1)+0.25M_{CcBBaa}(i-1)\Phi F_{CcBbAA}(i-1)+0.125M_{CcBBaa}(i-$
$1)(1-\Phi)F_{CcBBAa}(i-1)+0.25M_{CcBBaa}(i-1)\Phi F_{CcBBAa}(i-1)+0.25M_{CcBBaa}(i-1)F_{CcBBAA}(i-1)+$
$0.125M_{CcBBaa}(i-1)(1-\Phi)(1-\Phi)F_{CCBbAa}(i-1)+0.25M_{CcBBaa}(i-1)\Phi(1-\Phi)F_{CCBbAa}(i-1)+$
$0.25M_{CcBBaa}(i-1)(1-\Phi)\Phi F_{CCBbAa}(i-1)+0.5M_{CcBBaa}(i-1)\Phi\Phi F_{CCBbAa}(i-1)+0.25M_{CcBBaa}(i-$

$1)(1-\Phi)F_{CCBbAA}(i-1)+0.5M_{CcBBaa}(i-1)\Phi F_{CCBbAA}(i-1)+0.25M_{CcBBaa}(i-1)(1-\Phi)F_{CCBBAa}(i-$
$1)+0.5M_{CcBBaa}(i-1)\Phi F_{CCBBAa}(i-1)+0.5M_{CcBBaa}(i-1)F_{CCBBAA}(i-1)+0.0625(1-\Phi)M_{CcBBAa}(i-$
$1)(1-\Phi)F_{CcBbaa}(i-1)+0.125(1-\Phi)M_{CcBBAa}(i-1)\Phi F_{CcBbaa}(i-1)+0.0625(1-\Phi)M_{CcBBAa}(i-1)(1-$
$\Phi)(1-\Phi)F_{CcBbAa}(i-1)+0.125(1-\Phi)M_{CcBBAa}(i-1)\Phi(1-\Phi)F_{CcBbAa}(i-1)+0.0625(1-\Phi)M_{CcBBAa}(i-$
$1)(1-\Phi)\Phi F_{CcBbAa}(i-1)+0.125(1-\Phi)M_{CcBBAa}(i-1)\Phi\Phi F_{CcBbAa}(i-1)+0.0625(1-\Phi)M_{CcBBAa}(i-1)(1-$
$\Phi)F_{CcBbAA}(i-1)+0.125(1-\Phi)M_{CcBBAa}(i-1)\Phi F_{CcBbAA}(i-1)+0.125(1-\Phi)M_{CcBBAa}(i-1)F_{CcBBaa}(i-$
$1)+0.125(1-\Phi)M_{CcBBAa}(i-1)(1-\Phi)F_{CcBBAa}(i-1)+0.125(1-\Phi)M_{CcBBAa}(i-1)\Phi F_{CcBBAa}(i-$
$1)+0.125(1-\Phi)M_{CcBBAa}(i-1)F_{CcBBAA}(i-1)+0.125(1-\Phi)M_{CcBBAa}(i-1)(1-\Phi)F_{CCBbaa}(i-1)+$
$0.25(1-\Phi)M_{CcBBAa}(i-1)\Phi F_{CCBbaa}(i-1)+0.125(1-\Phi)M_{CcBBAa}(i-1)(1-\Phi)(1-\Phi)F_{CCBbAa}(i-1)+$
$0.25(1-\Phi)M_{CcBBAa}(i-1)\Phi(1-\Phi)F_{CCBbAa}(i-1)+0.25(1-\Phi)M_{CcBBAa}(i-1)(1-\Phi)\Phi F_{CCBbAa}(i-$
$1)+0.25(1-\Phi)M_{CcBBAa}(i-1)\Phi\Phi F_{CCBbAa}(i-1)+0.125(1-\Phi)M_{CcBBAa}(i-1)(1-\Phi)F_{CCBbAA}(i-$
$1)+0.25(1-\Phi)M_{CcBBAa}(i-1)\Phi F_{CCBbAA}(i-1)+0.25(1-\Phi)M_{CcBBAa}(i-1)F_{CCBBaa}(i-1)+0.25(1-$
$\Phi)M_{CcBBAa}(i-1)(1-\Phi)F_{CCBBAa}(i-1)+0.25(1-\Phi)M_{CcBBAa}(i-1)\Phi F_{CCBBAa}(i-1)+0.25(1-$
$\Phi)M_{CcBBAa}(i-1)F_{CCBBAA}(i-1)+0.125\Phi M_{CcBBAa}(i-1)(1-\Phi)F_{CcBbaa}(i-1)+0.25\Phi M_{CcBBAa}(i-$
$1)\Phi F_{CcBbaa}(i-1)+0.0625\Phi M_{CcBBAa}(i-1)(1-\Phi)(1-\Phi)F_{CcBbAa}(i-1)+0.125\Phi M_{CcBBAa}(i-1)\Phi(1-$
$\Phi)F_{CcBbAa}(i-1)+0.25\Phi M_{CcBBAa}(i-1)F_{CcBBaa}(i-1)+0.125\Phi M_{CcBBAa}(i-1)(1-\Phi)F_{CcBBAa}(i-1)+$
$0.25\Phi M_{CcBBAa}(i-1)(1-\Phi)F_{CCBbaa}(i-1)+0.5\Phi M_{CcBBAa}(i-1)\Phi F_{CCBbaa}(i-1)+0.125\Phi M_{CcBBAa}(i-$
$1)(1-\Phi)(1-\Phi)F_{CCBbAa}(i-1)+0.25\Phi M_{CcBBAa}(i-1)\Phi(1-\Phi)F_{CCBbAa}(i-1)+0.25\Phi M_{CcBBAa}(i-1)(1-$
$\Phi)\Phi F_{CCBbAa}(i-1)+0.5\Phi M_{CcBBAa}(i-1)F_{CCBBaa}(i-1)+0.25\Phi M_{CcBBAa}(i-1)(1-\Phi)F_{CCBBAa}(i-1)+$
$0.125M_{CcBBAA}(i-1)(1-\Phi)F_{CcBbaa}(i-1)+0.25M_{CcBBAA}(i-1)\Phi F_{CcBbaa}(i-1)+0.0625M_{CcBBAA}(i-$
$1)(1-\Phi)(1-\Phi)F_{CcBbAa}(i-1)+0.125M_{CcBBAA}(i-1)\Phi(1-\Phi)F_{CcBbAa}(i-1)+0.25M_{CcBBAA}(i-$
$1)F_{CcBBaa}(i-1)+0.125M_{CcBBAA}(i-1)(1-\Phi)F_{CcBBAa}(i-1)+0.25M_{CcBBAA}(i-1)(1-\Phi)F_{CCBbaa}(i-1)+$
$0.5M_{CcBBAA}(i-1)\Phi F_{CCBbaa}(i-1)+0.125M_{CcBBAA}(i-1)(1-\Phi)(1-\Phi)F_{CCBbAa}(i-1)+0.25M_{CcBBAA}(i-$
$1)\Phi(1-\Phi)F_{CCBbAa}(i-1)+0.25M_{CcBBAA}(i-1)(1-\Phi)\Phi F_{CCBbAa}(i-1)+0.5M_{CcBBAA}(i-1)F_{CCBBaa}(i-$
$1)+0.25M_{CcBBAA}(i-1)(1-\Phi)F_{CCBBAa}(i-1)+0.0625(1-\Phi)M_{CCBbaa}(i-1)(1-\Phi)(1-\Phi)F_{CcBbAa}(i-1)+$
$0.125(1-\Phi)M_{CCBbaa}(i-1)\Phi(1-\Phi)F_{CcBbAa}(i-1)+0.125(1-\Phi)M_{CCBbaa}(i-1)(1-\Phi)\Phi F_{CcBbAa}(i-$
$1)+0.25(1-\Phi)M_{CCBbaa}(i-1)\Phi\Phi F_{CcBbAa}(i-1)+0.125(1-\Phi)M_{CCBbaa}(i-1)(1-\Phi)F_{CcBbAA}(i-$
$1)+0.25(1-\Phi)M_{CCBbaa}(i-1)\Phi F_{CcBbAA}(i-1)+0.125(1-\Phi)M_{CCBbaa}(i-1)(1-\Phi)F_{CcBBAa}(i-1)+$
$0.25(1-\Phi)M_{CCBbaa}(i-1)\Phi F_{CcBBAa}(i-1)+0.25(1-\Phi)M_{CCBbaa}(i-1)F_{CcBBAA}(i-1)+0.125(1-$
$\Phi)M_{CCBbaa}(i-1)(1-\Phi)(1-\Phi)F_{CCBbAa}(i-1)+0.25(1-\Phi)M_{CCBbaa}(i-1)\Phi(1-\Phi)F_{CCBbAa}(i-$
$1)+0.25(1-\Phi)M_{CCBbaa}(i-1)(1-\Phi)\Phi F_{CCBbAa}(i-1)+0.5(1-\Phi)M_{CCBbaa}(i-1)\Phi\Phi F_{CCBbAa}(i-$
$1)+0.25(1-\Phi)M_{CCBbaa}(i-1)(1-\Phi)F_{CCBbAA}(i-1)+0.5(1-\Phi)M_{CCBbaa}(i-1)\Phi F_{CCBbAA}(i-1)+$
$0.25(1-\Phi)M_{CCBbaa}(i-1)(1-\Phi)F_{CCBBAa}(i-1)+0.5(1-\Phi)M_{CCBbaa}(i-1)\Phi F_{CCBBAa}(i-1)+0.5(1-$
$\Phi)M_{CCBbaa}(i-1)F_{CCBBAA}(i-1)+0.125\Phi M_{CCBbaa}(i-1)(1-\Phi)(1-\Phi)F_{CcBbAa}(i-1)+0.25\Phi M_{CCBbaa}(i-$
$1)\Phi(1-\Phi)F_{CcBbAa}(i-1)+0.25\Phi M_{CCBbaa}(i-1)(1-\Phi)\Phi F_{CcBbAa}(i-1)+0.5\Phi M_{CCBbaa}(i-1)\Phi\Phi F_{CcBbAa}(i-$
$1)+0.25\Phi M_{CCBbaa}(i-1)(1-\Phi)F_{CcBbAA}(i-1)+0.5\Phi M_{CCBbaa}(i-1)\Phi F_{CcBbAA}(i-1)+0.25\Phi M_{CCBbaa}(i-$
$1)(1-\Phi)F_{CcBBAa}(i-1)+0.5\Phi M_{CCBbaa}(i-1)\Phi F_{CcBBAa}(i-1)+0.5\Phi M_{CCBbaa}(i-1)F_{CcBBAA}(i-1)+$
$0.25\Phi M_{CCBbaa}(i-1)(1-\Phi)(1-\Phi)F_{CCBbAa}(i-1)+0.5\Phi M_{CCBbaa}(i-1)\Phi(1-\Phi)F_{CCBbAa}(i-1)+$
$0.5\Phi M_{CCBbaa}(i-1)(1-\Phi)\Phi F_{CCBbAa}(i-1)+\Phi M_{CCBbaa}(i-1)\Phi\Phi F_{CCBbAa}(i-1)+0.5\Phi M_{CCBbaa}(i-$
$1)(1-\Phi)F_{CCBbAA}(i-1)+\Phi M_{CCBbaa}(i-1)\Phi F_{CCBbAA}(i-1)+0.5\Phi M_{CCBbaa}(i-1)(1-\Phi)F_{CCBBAa}(i-1)+$
$\Phi M_{CCBbaa}(i-1)\Phi F_{CCBBAa}(i-1)+\Phi M_{CCBbaa}(i-1)F_{CCBBAA}(i-1)+0.0625(1-\Phi)(1-\Phi)M_{CCBbAa}(i-$
$1)(1-\Phi)F_{CcBbaa}(i-1)+0.125(1-\Phi)(1-\Phi)M_{CCBbAa}(i-1)\Phi F_{CcBbaa}(i-1)+0.0625(1-\Phi)(1-$
$\Phi)M_{CCBbAa}(i-1)(1-\Phi)(1-\Phi)F_{CcBbAa}(i-1)+0.125(1-\Phi)(1-\Phi)M_{CCBbAa}(i-1)\Phi(1-\Phi)F_{CcBbAa}(i-$
$1)+0.0625(1-\Phi)(1-\Phi)M_{CCBbAa}(i-1)(1-\Phi)\Phi F_{CcBbAa}(i-1)+0.125(1-\Phi)(1-\Phi)M_{CCBbAa}(i-$
$1)\Phi\Phi F_{CcBbAa}(i-1)+0.0625(1-\Phi)(1-\Phi)M_{CCBbAa}(i-1)(1-\Phi)F_{CcBbAA}(i-1)+0.125(1-\Phi)(1-$
$\Phi)M_{CCBbAa}(i-1)\Phi F_{CcBbAA}(i-1)+0.125(1-\Phi)(1-\Phi)M_{CCBbAa}(i-1)F_{CcBBaa}(i-1)+0.125(1-$
$\Phi)(1-\Phi)M_{CCBbAa}(i-1)(1-\Phi)F_{CcBBAa}(i-1)+0.125(1-\Phi)(1-\Phi)M_{CCBbAa}(i-1)\Phi F_{CcBBAa}(i-$
$1)+0.125(1-\Phi)(1-\Phi)M_{CCBbAa}(i-1)F_{CcBBAA}(i-1)+0.125(1-\Phi)(1-\Phi)M_{CCBbAa}(i-1)(1-$
$\Phi)F_{CCBbaa}(i-1)+0.25(1-\Phi)(1-\Phi)M_{CCBbAa}(i-1)\Phi F_{CCBbaa}(i-1)+0.125(1-\Phi)(1-\Phi)M_{CCBbAa}(i-$
$1)(1-\Phi)(1-\Phi)F_{CCBbAa}(i-1)+0.25(1-\Phi)(1-\Phi)M_{CCBbAa}(i-1)\Phi(1-\Phi)F_{CCBbAa}(i-1)+0.25(1-\Phi)(1-$
$\Phi)M_{CCBbAa}(i-1)(1-\Phi)\Phi F_{CCBbAa}(i-1)+0.25(1-\Phi)(1-\Phi)M_{CCBbAa}(i-1)\Phi\Phi F_{CCBbAa}(i-1)+0.125(1-$
$\Phi)(1-\Phi)M_{CCBbAa}(i-1)(1-\Phi)F_{CCBbAA}(i-1)+0.25(1-\Phi)(1-\Phi)M_{CCBbAa}(i-1)\Phi F_{CCBbAA}(i-1)+$
$0.25(1-\Phi)(1-\Phi)M_{CCBbAa}(i-1)F_{CCBBaa}(i-1)+0.25(1-\Phi)(1-\Phi)M_{CCBbAa}(i-1)(1-\Phi)F_{CCBBAa}(i-$
$1)+0.25(1-\Phi)(1-\Phi)M_{CCBbAa}(i-1)\Phi F_{CCBBAa}(i-1)+0.25(1-\Phi)(1-\Phi)M_{CCBbAa}(i-1)F_{CCBBAA}(i-$
$1)+0.125\Phi(1-\Phi)M_{CCBbAa}(i-1)(1-\Phi)F_{CcBbaa}(i-1)+0.25\Phi(1-\Phi)M_{CCBbAa}(i-1)\Phi F_{CcBbaa}(i-1)+$
$0.125\Phi(1-\Phi)M_{CCBbAa}(i-1)(1-\Phi)(1-\Phi)F_{CcBbAa}(i-1)+0.25\Phi(1-\Phi)M_{CCBbAa}(i-1)\Phi(1-\Phi)F_{CcBbAa}(i-$
$1)+0.125\Phi(1-\Phi)M_{CCBbAa}(i-1)(1-\Phi)\Phi F_{CcBbAa}(i-1)+0.25\Phi(1-\Phi)M_{CCBbAa}(i-1)\Phi\Phi F_{CcBbAa}(i-$

$1) + 0.125\Phi(1-\Phi)M_{CCBbAa}(i-1)(1-\Phi)F_{CcBbAA}(i-1) + 0.25\Phi(1-\Phi)M_{CCBbAa}(i-1)\Phi F_{CcBbAA}(i-$
$1) + 0.25\Phi(1-\Phi)M_{CCBbAa}(i-1)F_{CcBBaa}(i-1) + 0.25\Phi(1-\Phi)M_{CCBbAa}(i-1)(1-\Phi)F_{CcBBAa}(i-1) +$
$0.25\Phi(1-\Phi)M_{CCBbAa}(i-1)\Phi F_{CcBBAa}(i-1) + 0.25\Phi(1-\Phi)M_{CCBbAa}(i-1)F_{CcBBAA}(i-1) + 0.25\Phi(1-$
$\Phi)M_{CCBbAa}(i-1)(1-\Phi)F_{CCBbaa}(i-1) + 0.5\Phi(1-\Phi)M_{CCBbAa}(i-1)\Phi F_{CCBbaa}(i-1) + 0.25\Phi(1-$
$\Phi)M_{CCBbAa}(i-1)(1-\Phi)(1-\Phi)F_{CCBbAa}(i-1) + 0.5\Phi(1-\Phi)M_{CCBbAa}(i-1)\Phi(1-\Phi)F_{CCBbAa}(i-$
$1) + 0.5\Phi(1-\Phi)M_{CCBbAa}(i-1)(1-\Phi)\Phi F_{CCBbAa}(i-1) + 0.5\Phi(1-\Phi)M_{CCBbAa}(i-1)\Phi\Phi F_{CCBbAa}(i-$
$1) + 0.25\Phi(1-\Phi)M_{CCBbAa}(i-1)(1-\Phi)F_{CCBbAA}(i-1) + 0.5\Phi(1-\Phi)M_{CCBbAa}(i-1)\Phi F_{CCBbAA}(i-$
$1) + 0.5\Phi(1-\Phi)M_{CCBbAa}(i-1)F_{CCBBaa}(i-1) + 0.5\Phi(1-\Phi)M_{CCBbAa}(i-1)(1-\Phi)F_{CCBBAa}(i-1) +$
$0.5\Phi(1-\Phi)M_{CCBbAa}(i-1)\Phi F_{CCBBAa}(i-1) + 0.5\Phi(1-\Phi)M_{CCBbAa}(i-1)F_{CCBBAA}(i-1) + 0.125(1-$
$\Phi)\Phi M_{CCBbAa}(i-1)(1-\Phi)F_{CcBbaa}(i-1) + 0.25(1-\Phi)\Phi M_{CCBbAa}(i-1)\Phi F_{CcBbaa}(i-1) + 0.0625(1-$
$\Phi)\Phi M_{CCBbAa}(i-1)(1-\Phi)(1-\Phi)F_{CcBbAa}(i-1) + 0.125(1-\Phi)\Phi M_{CCBbAa}(i-1)\Phi(1-\Phi)F_{CcBbAa}(i-$
$1) + 0.25(1-\Phi)\Phi M_{CCBbAa}(i-1)F_{CcBBaa}(i-1) + 0.125(1-\Phi)\Phi M_{CCBbAa}(i-1)(1-\Phi)F_{CcBBAa}(i-1) +$
$0.25(1-\Phi)\Phi M_{CCBbAa}(i-1)(1-\Phi)F_{CCBbaa}(i-1) + 0.5(1-\Phi)\Phi M_{CCBbAa}(i-1)\Phi F_{CCBbaa}(i-1) + 0.125(1-$
$\Phi)\Phi M_{CCBbAa}(i-1)(1-\Phi)(1-\Phi)F_{CCBbAa}(i-1) + 0.25(1-\Phi)\Phi M_{CCBbAa}(i-1)\Phi(1-\Phi)F_{CCBbAa}(i-$
$1) + 0.25(1-\Phi)\Phi M_{CCBbAa}(i-1)(1-\Phi)\Phi F_{CCBbAa}(i-1) + 0.5(1-\Phi)\Phi M_{CCBbAa}(i-1)F_{CCBBaa}(i-$
$1) + 0.25(1-\Phi)\Phi M_{CCBbAa}(i-1)(1-\Phi)F_{CCBBAa}(i-1) + 0.25\Phi\Phi M_{CCBbAa}(i-1)(1-\Phi)F_{CcBbaa}(i-$
$1) + 0.5\Phi\Phi M_{CCBbAa}(i-1)\Phi F_{CcBbaa}(i-1) + 0.125\Phi\Phi M_{CCBbAa}(i-1)(1-\Phi)(1-\Phi)F_{CcBbAa}(i-1) +$
$0.25\Phi\Phi M_{CCBbAa}(i-1)\Phi(1-\Phi)F_{CcBbAa}(i-1) + 0.5\Phi\Phi M_{CCBbAa}(i-1)F_{CcBBaa}(i-1) + 0.25\Phi\Phi M_{CCBbAa}(i-$
$1)(1-\Phi)F_{CcBBAa}(i-1) + 0.5\Phi\Phi M_{CCBbAa}(i-1)(1-\Phi)F_{CCBbaa}(i-1) + \Phi\Phi M_{CCBbAa}(i-1)\Phi F_{CCBbaa}(i-$
$1) + 0.25\Phi\Phi M_{CCBbAa}(i-1)(1-\Phi)(1-\Phi)F_{CCBbAa}(i-1) + 0.5\Phi\Phi M_{CCBbAa}(i-1)\Phi(1-\Phi)F_{CCBbAa}(i-1) +$
$0.5\Phi\Phi M_{CCBbAa}(i-1)(1-\Phi)\Phi F_{CCBbAa}(i-1) + \Phi\Phi M_{CCBbAa}(i-1)F_{CCBBaa}(i-1) + 0.5\Phi\Phi M_{CCBbAa}(i-$
$1)(1-\Phi)F_{CCBBAa}(i-1) + 0.125(1-\Phi)M_{CCBbAA}(i-1)(1-\Phi)F_{CcBbaa}(i-1) + 0.25(1-\Phi)M_{CCBbAA}(i-$
$1)\Phi F_{CcBbaa}(i-1) + 0.0625(1-\Phi)M_{CCBbAA}(i-1)(1-\Phi)(1-\Phi)F_{CcBbAa}(i-1) + 0.125(1-\Phi)M_{CCBbAA}(i-$
$1)\Phi(1-\Phi)F_{CcBbAa}(i-1) + 0.25(1-\Phi)M_{CCBbAA}(i-1)F_{CcBBaa}(i-1) + 0.125(1-\Phi)M_{CCBbAA}(i-$
$1)(1-\Phi)F_{CcBBAa}(i-1) + 0.25(1-\Phi)M_{CCBbAA}(i-1)(1-\Phi)F_{CCBbaa}(i-1) + 0.5(1-\Phi)M_{CCBbAA}(i-$
$1)\Phi F_{CCBbaa}(i-1) + 0.125(1-\Phi)M_{CCBbAA}(i-1)(1-\Phi)(1-\Phi)F_{CCBbAa}(i-1) + 0.25(1-\Phi)M_{CCBbAA}(i-$
$1)\Phi(1-\Phi)F_{CCBbAa}(i-1) + 0.25(1-\Phi)M_{CCBbAA}(i-1)(1-\Phi)\Phi F_{CCBbAa}(i-1) + 0.5(1-\Phi)M_{CCBbAA}(i-$
$1)F_{CCBBaa}(i-1) + 0.25(1-\Phi)M_{CCBbAA}(i-1)(1-\Phi)F_{CCBBAa}(i-1) + 0.25\Phi M_{CCBbAA}(i-1)(1-$
$\Phi)F_{CcBbaa}(i-1) + 0.5\Phi M_{CCBbAA}(i-1)\Phi F_{CcBbaa}(i-1) + 0.125\Phi M_{CCBbAA}(i-1)(1-\Phi)(1-\Phi)F_{CcBbAa}(i-$
$1) + 0.25\Phi M_{CCBbAA}(i-1)\Phi(1-\Phi)F_{CcBbAa}(i-1) + 0.5\Phi M_{CCBbAA}(i-1)F_{CcBBaa}(i-1) + 0.25\Phi M_{CCBbAA}(i-$
$1)(1-\Phi)F_{CcBBAa}(i-1) + 0.5\Phi M_{CCBbAA}(i-1)(1-\Phi)F_{CCBbaa}(i-1) + \Phi M_{CCBbAA}(i-1)\Phi F_{CCBbaa}(i-$
$1) + 0.25\Phi M_{CCBbAA}(i-1)(1-\Phi)(1-\Phi)F_{CCBbAa}(i-1) + 0.5\Phi M_{CCBbAA}(i-1)\Phi(1-\Phi)F_{CCBbAa}(i-1) +$
$0.5\Phi M_{CCBbAA}(i-1)(1-\Phi)\Phi F_{CCBbAa}(i-1) + \Phi M_{CCBbAA}(i-1)F_{CCBBaa}(i-1) + 0.5\Phi M_{CCBbAA}(i-$
$1)(1-\Phi)F_{CCBBAa}(i-1) + 0.125M_{CCBBaa}(i-1)(1-\Phi)(1-\Phi)F_{CcBbAa}(i-1) + 0.25M_{CCBBaa}(i-1)\Phi(1-$
$\Phi)F_{CcBbAa}(i-1) + 0.25M_{CCBBaa}(i-1)(1-\Phi)\Phi F_{CcBbAa}(i-1) + 0.5M_{CCBBaa}(i-1)\Phi\Phi F_{CcBbAa}(i-1) +$
$0.25M_{CCBBaa}(i-1)(1-\Phi)F_{CcBbAA}(i-1) + 0.5M_{CCBBaa}(i-1)\Phi F_{CcBbAA}(i-1) + 0.25M_{CCBBaa}(i-$
$1)(1-\Phi)F_{CcBBAa}(i-1) + 0.5M_{CCBBaa}(i-1)\Phi F_{CcBBAa}(i-1) + 0.5M_{CCBBaa}(i-1)F_{CcBBAA}(i-1) +$
$0.25M_{CCBBaa}(i-1)(1-\Phi)(1-\Phi)F_{CCBbAa}(i-1) + 0.5M_{CCBBaa}(i-1)\Phi(1-\Phi)F_{CCBbAa}(i-1) +$
$0.5M_{CCBBaa}(i-1)(1-\Phi)\Phi F_{CCBbAa}(i-1) + M_{CCBBaa}(i-1)\Phi\Phi F_{CCBbAa}(i-1) + 0.5M_{CCBBaa}(i-$
$1)(1-\Phi)F_{CCBbAA}(i-1) + M_{CCBBaa}(i-1)\Phi F_{CCBbAA}(i-1) + 0.5M_{CCBBaa}(i-1)(1-\Phi)F_{CCBBAa}(i-$
$1) + M_{CCBBaa}(i-1)\Phi F_{CCBBAa}(i-1) + M_{CCBBaa}(i-1)F_{CCBBAA}(i-1) + 0.125(1-\Phi)M_{CCBBAa}(i-1)(1-$
$\Phi)F_{CcBbaa}(i-1) + 0.25(1-\Phi)M_{CCBBAa}(i-1)\Phi F_{CcBbaa}(i-1) + 0.125(1-\Phi)M_{CCBBAa}(i-1)(1-\Phi)(1-$
$\Phi)F_{CcBbAa}(i-1) + 0.25(1-\Phi)M_{CCBBAa}(i-1)\Phi(1-\Phi)F_{CcBbAa}(i-1) + 0.125(1-\Phi)M_{CCBBAa}(i-1)(1-$
$\Phi)\Phi F_{CcBbAa}(i-1) + 0.25(1-\Phi)M_{CCBBAa}(i-1)\Phi\Phi F_{CcBbAa}(i-1) + 0.125(1-\Phi)M_{CCBBAa}(i-1)(1-$
$\Phi)F_{CcBbAA}(i-1) + 0.25(1-\Phi)M_{CCBBAa}(i-1)\Phi F_{CcBbAA}(i-1) + 0.25(1-\Phi)M_{CCBBAa}(i-1)F_{CcBBaa}(i-$
$1) + 0.25(1-\Phi)M_{CCBBAa}(i-1)(1-\Phi)F_{CcBBAa}(i-1) + 0.25(1-\Phi)M_{CCBBAa}(i-1)\Phi F_{CcBBAa}(i-$
$1) + 0.25(1-\Phi)M_{CCBBAa}(i-1)F_{CcBBAA}(i-1) + 0.25(1-\Phi)M_{CCBBAa}(i-1)(1-\Phi)F_{CCBbaa}(i-1) +$
$0.5(1-\Phi)M_{CCBBAa}(i-1)\Phi F_{CCBbaa}(i-1) + 0.25(1-\Phi)M_{CCBBAa}(i-1)(1-\Phi)(1-\Phi)F_{CCBbAa}(i-1) +$
$0.5(1-\Phi)M_{CCBBAa}(i-1)\Phi(1-\Phi)F_{CCBbAa}(i-1) + 0.5(1-\Phi)M_{CCBBAa}(i-1)(1-\Phi)\Phi F_{CCBbAa}(i-$
$1) + 0.5(1-\Phi)M_{CCBBAa}(i-1)\Phi\Phi F_{CCBbAa}(i-1) + 0.25(1-\Phi)M_{CCBBAa}(i-1)(1-\Phi)F_{CCBbAA}(i-$
$1) + 0.5(1-\Phi)M_{CCBBAa}(i-1)\Phi F_{CCBbAA}(i-1) + 0.5(1-\Phi)M_{CCBBAa}(i-1)F_{CCBBaa}(i-1) + 0.5(1-$
$\Phi)M_{CCBBAa}(i-1)(1-\Phi)F_{CCBBAa}(i-1) + 0.5(1-\Phi)M_{CCBBAa}(i-1)\Phi F_{CCBBAa}(i-1) + 0.5(1-$
$\Phi)M_{CCBBAa}(i-1)F_{CCBBAA}(i-1) + 0.25\Phi M_{CCBBAa}(i-1)(1-\Phi)F_{CcBbaa}(i-1) + 0.5\Phi M_{CCBBAa}(i-$
$1)\Phi F_{CcBbaa}(i-1) + 0.125\Phi M_{CCBBAa}(i-1)(1-\Phi)(1-\Phi)F_{CcBbAa}(i-1) + 0.25\Phi M_{CCBBAa}(i-1)\Phi(1-$
$\Phi)F_{CcBbAa}(i-1) + 0.5\Phi M_{CCBBAa}(i-1)F_{CcBBaa}(i-1) + 0.25\Phi M_{CCBBAa}(i-1)(1-\Phi)F_{CcBBAa}(i-1) +$
$0.5\Phi M_{CCBBAa}(i-1)(1-\Phi)F_{CCBbaa}(i-1) + \Phi M_{CCBBAa}(i-1)\Phi F_{CCBbaa}(i-1) + 0.25\Phi M_{CCBBAa}(i-$
$1)(1-\Phi)(1-\Phi)F_{CCBbAa}(i-1) + 0.5\Phi M_{CCBBAa}(i-1)\Phi(1-\Phi)F_{CCBbAa}(i-1) + 0.5\Phi M_{CCBBAa}(i-1)(1-$

$\Phi)\Phi F_{CCBbAa}(i-1)+\Phi M_{CCBBAa}(i-1)F_{CCBBaa}(i-1)+0.5\Phi M_{CCBBAa}(i-1)(1-\Phi)F_{CCBBAa}(i-1)+$
$0.25M_{CCBBAA}(i-1)(1-\Phi)F_{CcBbaa}(i-1)+0.5M_{CCBBAA}(i-1)\Phi F_{CcBbaa}(i-1)+0.125M_{CCBBAA}(i-1)(1-$
$\Phi)(1-\Phi)F_{CcBbAa}(i-1)+0.25M_{CCBBAA}(i-1)\Phi(1-\Phi)F_{CcBbAa}(i-1)+0.5M_{CCBBAA}(i-1)F_{CcBBaa}(i-1)+$
$0.25M_{CCBBAA}(i-1)(1-\Phi)F_{CcBBAa}(i-1)+0.5M_{CCBBAA}(i-1)(1-\Phi)F_{CCBbaa}(i-1)+M_{CCBBAA}(i-$
$1)\Phi F_{CCBbaa}(i-1)+0.25M_{CCBBAA}(i-1)(1-\Phi)(1-\Phi)F_{CCBbAa}(i-1)+0.5M_{CCBBAA}(i-1)\Phi(1-$
$\Phi)F_{CCBbAa}(i-1)+0.5M_{CCBBAA}(i-1)(1-\Phi)\Phi F_{CCBbAa}(i-1)+M_{CCBBAA}(i-1)F_{CCBBaa}(i-1)+$
$0.5M_{CCBBAA}(i-1)(1-\Phi)F_{CCBBAa}(i-1)],$

$\mathrm{M}^{e}_{CCBBAA}=\left(\frac{\bar{\Omega}_{CCBBAA}}{2}\right)[0.015625(1-\Phi)(1-\Phi)M_{CcBbAa}(i-1)(1-\Phi)(1-\Phi)F_{CcBbAa}(i-1)+$
$0.03125(1-\Phi)(1-\Phi)M_{CcBbAa}(i-1)\Phi(1-\Phi)F_{CcBbAa}(i-1)+0.03125(1-\Phi)(1-\Phi)M_{CcBbAa}(i-$
$1)(1-\Phi)\Phi F_{CcBbAa}(i-1)+0.0625(1-\Phi)(1-\Phi)M_{CcBbAa}(i-1)\Phi\Phi F_{CcBbAa}(i-1)+0.03125(1-\Phi)(1-$
$\Phi)M_{CcBbAa}(i-1)(1-\Phi)F_{CcBbAA}(i-1)+0.0625(1-\Phi)(1-\Phi)M_{CcBbAa}(i-1)\Phi F_{CcBbAA}(i-1)+0.03125(1-$
$\Phi)(1-\Phi)M_{CcBbAa}(i-1)(1-\Phi)F_{CcBBAa}(i-1)+0.0625(1-\Phi)(1-\Phi)M_{CcBbAa}(i-1)\Phi F_{CcBBAa}(i-$
$1)+0.0625(1-\Phi)(1-\Phi)M_{CcBbAa}(i-1)F_{CcBBAA}(i-1)+0.03125(1-\Phi)(1-\Phi)M_{CcBbAa}(i-1)(1-$
$\Phi)(1-\Phi)F_{CCBbAa}(i-1)+0.0625(1-\Phi)(1-\Phi)M_{CcBbAa}(i-1)\Phi(1-\Phi)F_{CCBbAa}(i-1)+0.0625(1-$
$\Phi)(1-\Phi)M_{CcBbAa}(i-1)(1-\Phi)\Phi F_{CCBbAa}(i-1)+0.125(1-\Phi)(1-\Phi)M_{CcBbAa}(i-1)\Phi\Phi F_{CCBbAa}(i-$
$1)+0.0625(1-\Phi)(1-\Phi)M_{CcBbAa}(i-1)(1-\Phi)F_{CCBbAA}(i-1)+0.125(1-\Phi)(1-\Phi)M_{CcBbAa}(i-$
$1)\Phi F_{CCBbAA}(i-1)+0.0625(1-\Phi)(1-\Phi)M_{CcBbAa}(i-1)(1-\Phi)F_{CCBBAa}(i-1)+0.125(1-\Phi)(1-$
$\Phi)M_{CcBbAa}(i-1)\Phi F_{CCBBAa}(i-1)+0.125(1-\Phi)(1-\Phi)M_{CcBbAa}(i-1)F_{CCBBAA}(i-1)+0.03125\Phi(1-$
$\Phi)M_{CcBbAa}(i-1)(1-\Phi)(1-\Phi)F_{CcBbAa}(i-1)+0.0625\Phi(1-\Phi)M_{CcBbAa}(i-1)\Phi(1-\Phi)F_{CcBbAa}(i-1)+$
$0.0625\Phi(1-\Phi)M_{CcBbAa}(i-1)(1-\Phi)\Phi F_{CcBbAa}(i-1)+0.125\Phi(1-\Phi)M_{CcBbAa}(i-1)\Phi\Phi F_{CcBbAa}(i-1)+$
$0.0625\Phi(1-\Phi)M_{CcBbAa}(i-1)(1-\Phi)F_{CcBbAA}(i-1)+0.125\Phi(1-\Phi)M_{CcBbAa}(i-1)\Phi F_{CcBbAA}(i-1)+$
$0.0625\Phi(1-\Phi)M_{CcBbAa}(i-1)(1-\Phi)F_{CcBBAa}(i-1)+0.125\Phi(1-\Phi)M_{CcBbAa}(i-1)\Phi F_{CcBBAa}(i-1)+$
$0.125\Phi(1-\Phi)M_{CcBbAa}(i-1)F_{CcBBAA}(i-1)+0.0625\Phi(1-\Phi)M_{CcBbAa}(i-1)(1-\Phi)(1-\Phi)F_{CCBbAa}(i-1)+$
$0.125\Phi(1-\Phi)M_{CcBbAa}(i-1)\Phi(1-\Phi)F_{CCBbAa}(i-1)+0.125\Phi(1-\Phi)M_{CcBbAa}(i-1)(1-\Phi)\Phi F_{CCBbAa}(i-$
$1)+0.25\Phi(1-\Phi)M_{CcBbAa}(i-1)\Phi\Phi F_{CCBbAa}(i-1)+0.125\Phi(1-\Phi)M_{CcBbAa}(i-1)(1-\Phi)F_{CCBbAA}(i-$
$1)+0.25\Phi(1-\Phi)M_{CcBbAa}(i-1)\Phi F_{CCBbAA}(i-1)+0.125\Phi(1-\Phi)M_{CcBbAa}(i-1)(1-\Phi)F_{CCBBAa}(i-1)+$
$0.25\Phi(1-\Phi)M_{CcBbAa}(i-1)\Phi F_{CCBBAa}(i-1)+0.25\Phi(1-\Phi)M_{CcBbAa}(i-1)F_{CCBBAA}(i-1)+0.03125(1-$
$\Phi)\Phi M_{CcBbAa}(i-1)(1-\Phi)(1-\Phi)F_{CcBbAa}(i-1)+0.0625(1-\Phi)\Phi M_{CcBbAa}(i-1)\Phi(1-\Phi)F_{CcBbAa}(i-1)+$
$0.0625(1-\Phi)\Phi M_{CcBbAa}(i-1)(1-\Phi)\Phi F_{CcBbAa}(i-1)+0.125(1-\Phi)\Phi M_{CcBbAa}(i-1)\Phi\Phi F_{CcBbAa}(i-1)+$
$0.0625(1-\Phi)\Phi M_{CcBbAa}(i-1)(1-\Phi)F_{CcBbAA}(i-1)+0.125(1-\Phi)\Phi M_{CcBbAa}(i-1)\Phi F_{CcBbAA}(i-1)+$
$0.0625(1-\Phi)\Phi M_{CcBbAa}(i-1)(1-\Phi)F_{CcBBAa}(i-1)+0.125(1-\Phi)\Phi M_{CcBbAa}(i-1)\Phi F_{CcBBAa}(i-1)+$
$0.125(1-\Phi)\Phi M_{CcBbAa}(i-1)F_{CcBBAA}(i-1)+0.0625(1-\Phi)\Phi M_{CcBbAa}(i-1)(1-\Phi)(1-\Phi)F_{CCBbAa}(i-1)+$
$0.125(1-\Phi)\Phi M_{CcBbAa}(i-1)\Phi(1-\Phi)F_{CCBbAa}(i-1)+0.125(1-\Phi)\Phi M_{CcBbAa}(i-1)(1-\Phi)\Phi F_{CCBbAa}(i-$
$1)+0.25(1-\Phi)\Phi M_{CcBbAa}(i-1)\Phi\Phi F_{CCBbAa}(i-1)+0.125(1-\Phi)\Phi M_{CcBbAa}(i-1)(1-\Phi)F_{CCBbAA}(i-1)+$
$0.25(1-\Phi)\Phi M_{CcBbAa}(i-1)\Phi F_{CCBbAA}(i-1)+0.125(1-\Phi)\Phi M_{CcBbAa}(i-1)(1-\Phi)F_{CCBBAa}(i-1)+0.25(1-$
$\Phi)\Phi M_{CcBbAa}(i-1)\Phi F_{CCBBAa}(i-1)+0.25(1-\Phi)\Phi M_{CcBbAa}(i-1)F_{CCBBAA}(i-1)+0.0625\Phi\Phi M_{CcBbAa}(i-$
$1)(1-\Phi)(1-\Phi)F_{CcBbAa}(i-1)+0.125\Phi\Phi M_{CcBbAa}(i-1)\Phi(1-\Phi)F_{CcBbAa}(i-1)+0.125\Phi\Phi M_{CcBbAa}(i-$
$1)(1-\Phi)\Phi F_{CcBbAa}(i-1)+0.25\Phi\Phi M_{CcBbAa}(i-1)\Phi\Phi F_{CcBbAa}(i-1)+0.125\Phi\Phi M_{CcBbAa}(i-1)(1-$
$\Phi)F_{CcBbAA}(i-1)+0.25\Phi\Phi M_{CcBbAa}(i-1)\Phi F_{CcBbAA}(i-1)+0.125\Phi\Phi M_{CcBbAa}(i-1)(1-\Phi)F_{CcBBAa}(i-$
$1)+0.25\Phi\Phi M_{CcBbAa}(i-1)\Phi F_{CcBBAa}(i-1)+0.25\Phi\Phi M_{CcBbAa}(i-1)F_{CcBBAA}(i-1)+0.125\Phi\Phi M_{CcBbAa}(i-$
$1)(1-\Phi)(1-\Phi)F_{CCBbAa}(i-1)+0.25\Phi\Phi M_{CcBbAa}(i-1)\Phi(1-\Phi)F_{CCBbAa}(i-1)+0.25\Phi\Phi M_{CcBbAa}(i-$
$1)(1-\Phi)\Phi F_{CCBbAa}(i-1)+0.5\Phi\Phi M_{CcBbAa}(i-1)\Phi\Phi F_{CCBbAa}(i-1)+0.25\Phi\Phi M_{CcBbAa}(i-1)(1-$
$\Phi)F_{CCBbAA}(i-1)+0.5\Phi\Phi M_{CcBbAa}(i-1)\Phi F_{CCBbAA}(i-1)+0.25\Phi\Phi M_{CcBbAa}(i-1)(1-\Phi)F_{CCBBAa}(i-$
$1)+0.5\Phi\Phi M_{CcBbAa}(i-1)\Phi F_{CCBBAa}(i-1)+0.5\Phi\Phi M_{CcBbAa}(i-1)F_{CCBBAA}(i-1)+0.03125(1-$
$\Phi)M_{CcBbAA}(i-1)(1-\Phi)(1-\Phi)F_{CcBbAa}(i-1)+0.0625(1-\Phi)M_{CcBbAA}(i-1)\Phi(1-\Phi)F_{CcBbAa}(i-$
$1)+0.0625(1-\Phi)M_{CcBbAA}(i-1)(1-\Phi)\Phi F_{CcBbAa}(i-1)+0.125(1-\Phi)M_{CcBbAA}(i-1)\Phi\Phi F_{CcBbAa}(i-$
$1)+0.0625(1-\Phi)M_{CcBbAA}(i-1)(1-\Phi)F_{CcBbAA}(i-1)+0.125(1-\Phi)M_{CcBbAA}(i-1)\Phi F_{CcBbAA}(i-1)+$
$0.0625(1-\Phi)M_{CcBbAA}(i-1)(1-\Phi)F_{CcBBAa}(i-1)+0.125(1-\Phi)M_{CcBbAA}(i-1)\Phi F_{CcBBAa}(i-1)+$
$0.125(1-\Phi)M_{CcBbAA}(i-1)F_{CcBBAA}(i-1)+0.0625(1-\Phi)M_{CcBbAA}(i-1)(1-\Phi)(1-\Phi)F_{CCBbAa}(i-1)+$
$0.125(1-\Phi)M_{CcBbAA}(i-1)\Phi(1-\Phi)F_{CCBbAa}(i-1)+0.125(1-\Phi)M_{CcBbAA}(i-1)(1-\Phi)\Phi F_{CCBbAa}(i-$
$1)+0.25(1-\Phi)M_{CcBbAA}(i-1)\Phi\Phi F_{CCBbAa}(i-1)+0.125(1-\Phi)M_{CcBbAA}(i-1)(1-\Phi)F_{CCBbAA}(i-1)+$
$0.25(1-\Phi)M_{CcBbAA}(i-1)\Phi F_{CCBbAA}(i-1)+0.125(1-\Phi)M_{CcBbAA}(i-1)(1-\Phi)F_{CCBBAa}(i-1)+0.25(1-$
$\Phi)M_{CcBbAA}(i-1)\Phi F_{CCBBAa}(i-1)+0.25(1-\Phi)M_{CcBbAA}(i-1)F_{CCBBAA}(i-1)+0.0625\Phi M_{CcBbAA}(i-$
$1)(1-\Phi)(1-\Phi)F_{CcBbAa}(i-1)+0.125\Phi M_{CcBbAA}(i-1)\Phi(1-\Phi)F_{CcBbAa}(i-1)+0.125\Phi M_{CcBbAA}(i-1)(1-$
$\Phi)\Phi F_{CcBbAa}(i-1)+0.25\Phi M_{CcBbAA}(i-1)\Phi\Phi F_{CcBbAa}(i-1)+0.125\Phi M_{CcBbAA}(i-1)(1-\Phi)F_{CcBbAA}(i-1)+$
$0.25\Phi M_{CcBbAA}(i-1)\Phi F_{CcBbAA}(i-1)+0.125\Phi M_{CcBbAA}(i-1)(1-\Phi)F_{CcBBAa}(i-1)+0.25\Phi M_{CcBbAA}(i-$

$1)\Phi F_{CcBBAa}(i-1)+0.25\Phi M_{CcBbAA}(i-1)F_{CcBBAA}(i-1)+0.125\Phi M_{CcBbAA}(i-1)(1-\Phi)(1-\Phi)F_{CCBbAa}(i-$
$1)+0.25\Phi M_{CcBbAA}(i-1)\Phi(1-\Phi)F_{CCBbAa}(i-1)+0.25\Phi M_{CcBbAA}(i-1)(1-\Phi)\Phi F_{CCBbAa}(i-1)+$
$0.5\Phi M_{CcBbAA}(i-1)\Phi\Phi F_{CCBbAa}(i-1)+0.25\Phi M_{CcBbAA}(i-1)(1-\Phi)F_{CCBbAA}(i-1)+0.5\Phi M_{CcBbAA}(i-$
$1)\Phi F_{CCBbAA}(i-1)+0.25\Phi M_{CcBbAA}(i-1)(1-\Phi)F_{CCBBAa}(i-1)+0.5\Phi M_{CcBbAA}(i-1)\Phi F_{CCBBAa}(i-$
$1)+0.5\Phi M_{CcBbAA}(i-1)F_{CCBBAA}(i-1)+0.03125(1-\Phi)M_{CcBBAa}(i-1)(1-\Phi)(1-\Phi)F_{CcBbAa}(i-1)+$
$0.0625(1-\Phi)M_{CcBBAa}(i-1)\Phi(1-\Phi)F_{CcBbAa}(i-1)+0.0625(1-\Phi)M_{CcBBAa}(i-1)(1-\Phi)\Phi F_{CcBbAa}(i-$
$1)+0.125(1-\Phi)M_{CcBBAa}(i-1)\Phi\Phi F_{CcBbAa}(i-1)+0.0625(1-\Phi)M_{CcBBAa}(i-1)(1-\Phi)F_{CcBbAA}(i-$
$1)+0.125(1-\Phi)M_{CcBBAa}(i-1)\Phi F_{CcBbAA}(i-1)+0.0625(1-\Phi)M_{CcBBAa}(i-1)(1-\Phi)F_{CcBBAa}(i-1)+$
$0.125(1-\Phi)M_{CcBBAa}(i-1)\Phi F_{CcBBAa}(i-1)+0.125(1-\Phi)M_{CcBBAa}(i-1)F_{CcBBAA}(i-1)+0.0625(1-$
$\Phi)M_{CcBBAa}(i-1)(1-\Phi)(1-\Phi)F_{CCBbAa}(i-1)+0.125(1-\Phi)M_{CcBBAa}(i-1)\Phi(1-\Phi)F_{CCBbAa}(i-$
$1)+0.125(1-\Phi)M_{CcBBAa}(i-1)(1-\Phi)\Phi F_{CCBbAa}(i-1)+0.25(1-\Phi)M_{CcBBAa}(i-1)\Phi\Phi F_{CCBbAa}(i-$
$1)+0.125(1-\Phi)M_{CcBBAa}(i-1)(1-\Phi)F_{CCBbAA}(i-1)+0.25(1-\Phi)M_{CcBBAa}(i-1)\Phi F_{CCBbAA}(i-$
$1)+0.125(1-\Phi)M_{CcBBAa}(i-1)(1-\Phi)F_{CCBBAa}(i-1)+0.25(1-\Phi)M_{CcBBAa}(i-1)\Phi F_{CCBBAa}(i-$
$1)+0.25(1-\Phi)M_{CcBBAa}(i-1)F_{CCBBAA}(i-1)+0.0625\Phi M_{CcBBAa}(i-1)(1-\Phi)(1-\Phi)F_{CcBbAa}(i-$
$1)+0.125\Phi M_{CcBBAa}(i-1)\Phi(1-\Phi)F_{CcBbAa}(i-1)+0.125\Phi M_{CcBBAa}(i-1)(1-\Phi)\Phi F_{CcBbAa}(i-1)+$
$0.25\Phi M_{CcBBAa}(i-1)\Phi\Phi F_{CcBbAa}(i-1)+0.125\Phi M_{CcBBAa}(i-1)(1-\Phi)F_{CcBbAA}(i-1)+0.25\Phi M_{CcBBAa}(i-$
$1)\Phi F_{CcBbAA}(i-1)+0.125\Phi M_{CcBBAa}(i-1)(1-\Phi)F_{CcBBAa}(i-1)+0.25\Phi M_{CcBBAa}(i-1)\Phi F_{CcBBAa}(i-$
$1)+0.25\Phi M_{CcBBAa}(i-1)F_{CcBBAA}(i-1)+0.125\Phi M_{CcBBAa}(i-1)(1-\Phi)(1-\Phi)F_{CCBbAa}(i-1)+$
$0.25\Phi M_{CcBBAa}(i-1)\Phi(1-\Phi)F_{CCBbAa}(i-1)+0.25\Phi M_{CcBBAa}(i-1)(1-\Phi)\Phi F_{CCBbAa}(i-1)+0.5\Phi M_{CcBBAa}(i-$
$1)\Phi\Phi F_{CCBbAa}(i-1)+0.25\Phi M_{CcBBAa}(i-1)(1-\Phi)F_{CCBbAA}(i-1)+0.5\Phi M_{CcBBAa}(i-1)\Phi F_{CCBbAA}(i-1)+$
$0.25\Phi M_{CcBBAa}(i-1)(1-\Phi)F_{CCBBAa}(i-1)+0.5\Phi M_{CcBBAa}(i-1)\Phi F_{CCBBAa}(i-1)+0.5\Phi M_{CcBBAa}(i-$
$1)F_{CCBBAA}(i-1)+0.0625M_{CcBBAA}(i-1)(1-\Phi)(1-\Phi)F_{CcBbAa}(i-1)+0.125M_{CcBBAA}(i-1)\Phi(1-$
$\Phi)F_{CcBbAa}(i-1)+0.125M_{CcBBAA}(i-1)(1-\Phi)\Phi F_{CcBbAa}(i-1)+0.25M_{CcBBAA}(i-1)\Phi\Phi F_{CcBbAa}(i-1)+$
$0.125M_{CcBBAA}(i-1)(1-\Phi)F_{CcBbAA}(i-1)+0.25M_{CcBBAA}(i-1)\Phi F_{CcBbAA}(i-1)+0.125M_{CcBBAA}(i-$
$1)(1-\Phi)F_{CcBBAa}(i-1)+0.25M_{CcBBAA}(i-1)\Phi F_{CcBBAa}(i-1)+0.25M_{CcBBAA}(i-1)F_{CcBBAA}(i-$
$1)+0.125M_{CcBBAA}(i-1)(1-\Phi)(1-\Phi)F_{CCBbAa}(i-1)+0.25M_{CcBBAA}(i-1)\Phi(1-\Phi)F_{CCBbAa}(i-1)+$
$0.25M_{CcBBAA}(i-1)(1-\Phi)\Phi F_{CCBbAa}(i-1)+0.5M_{CcBBAA}(i-1)\Phi\Phi F_{CCBbAa}(i-1)+0.25M_{CcBBAA}(i-$
$1)(1-\Phi)F_{CCBbAA}(i-1)+0.5M_{CcBBAA}(i-1)\Phi F_{CCBbAA}(i-1)+0.25M_{CcBBAA}(i-1)(1-\Phi)F_{CCBBAa}(i-$
$1)+0.5M_{CcBBAA}(i-1)\Phi F_{CCBBAa}(i-1)+0.5M_{CcBBAA}(i-1)F_{CCBBAA}(i-1)+0.03125(1-\Phi)(1-$
$\Phi)M_{CCBbAa}(i-1)(1-\Phi)(1-\Phi)F_{CcBbAa}(i-1)+0.0625(1-\Phi)(1-\Phi)M_{CCBbAa}(i-1)\Phi(1-\Phi)F_{CcBbAa}(i-$
$1)+0.0625(1-\Phi)(1-\Phi)M_{CCBbAa}(i-1)(1-\Phi)\Phi F_{CcBbAa}(i-1)+0.125(1-\Phi)(1-\Phi)M_{CCBbAa}(i-$
$1)\Phi\Phi F_{CcBbAa}(i-1)+0.0625(1-\Phi)(1-\Phi)M_{CCBbAa}(i-1)(1-\Phi)F_{CcBbAA}(i-1)+0.125(1-\Phi)(1-$
$\Phi)M_{CCBbAa}(i-1)\Phi F_{CcBbAA}(i-1)+0.0625(1-\Phi)(1-\Phi)M_{CCBbAa}(i-1)(1-\Phi)F_{CcBBAa}(i-1)+$
$0.125(1-\Phi)(1-\Phi)M_{CCBbAa}(i-1)\Phi F_{CcBBAa}(i-1)+0.125(1-\Phi)(1-\Phi)M_{CCBbAa}(i-1)F_{CcBBAA}(i-$
$1)+0.0625(1-\Phi)(1-\Phi)M_{CCBbAa}(i-1)(1-\Phi)(1-\Phi)F_{CCBbAa}(i-1)+0.125(1-\Phi)(1-\Phi)M_{CCBbAa}(i-$
$1)\Phi(1-\Phi)F_{CCBbAa}(i-1)+0.125(1-\Phi)(1-\Phi)M_{CCBbAa}(i-1)(1-\Phi)\Phi F_{CCBbAa}(i-1)+0.25(1-\Phi)(1-$
$\Phi)M_{CCBbAa}(i-1)\Phi\Phi F_{CCBbAa}(i-1)+0.125(1-\Phi)(1-\Phi)M_{CCBbAa}(i-1)(1-\Phi)F_{CCBbAA}(i-1)+0.25(1-$
$\Phi)(1-\Phi)M_{CCBbAa}(i-1)\Phi F_{CCBbAA}(i-1)+0.125(1-\Phi)(1-\Phi)M_{CCBbAa}(i-1)(1-\Phi)F_{CCBBAa}(i-1)+$
$0.25(1-\Phi)(1-\Phi)M_{CCBbAa}(i-1)\Phi F_{CCBBAa}(i-1)+0.25(1-\Phi)(1-\Phi)M_{CCBbAa}(i-1)F_{CCBBAA}(i-$
$1)+0.0625\Phi(1-\Phi)M_{CCBbAa}(i-1)(1-\Phi)(1-\Phi)F_{CcBbAa}(i-1)+0.125\Phi(1-\Phi)M_{CCBbAa}(i-1)\Phi(1-$
$\Phi)F_{CcBbAa}(i-1)+0.125\Phi(1-\Phi)M_{CCBbAa}(i-1)(1-\Phi)\Phi F_{CcBbAa}(i-1)+0.25\Phi(1-\Phi)M_{CCBbAa}(i-$
$1)\Phi\Phi F_{CcBbAa}(i-1)+0.125\Phi(1-\Phi)M_{CCBbAa}(i-1)(1-\Phi)F_{CcBbAA}(i-1)+0.25\Phi(1-\Phi)M_{CCBbAa}(i-$
$1)\Phi F_{CcBbAA}(i-1)+0.125\Phi(1-\Phi)M_{CCBbAa}(i-1)(1-\Phi)F_{CcBBAa}(i-1)+0.25\Phi(1-\Phi)M_{CCBbAa}(i-$
$1)\Phi F_{CcBBAa}(i-1)+0.25\Phi(1-\Phi)M_{CCBbAa}(i-1)F_{CcBBAA}(i-1)+0.125\Phi(1-\Phi)M_{CCBbAa}(i-1)(1-$
$\Phi)(1-\Phi)F_{CCBbAa}(i-1)+0.25\Phi(1-\Phi)M_{CCBbAa}(i-1)\Phi(1-\Phi)F_{CCBbAa}(i-1)+0.25\Phi(1-\Phi)M_{CCBbAa}(i-$
$1)(1-\Phi)\Phi F_{CCBbAa}(i-1)+0.5\Phi(1-\Phi)M_{CCBbAa}(i-1)\Phi\Phi F_{CCBbAa}(i-1)+0.25\Phi(1-\Phi)M_{CCBbAa}(i-$
$1)(1-\Phi)F_{CCBbAA}(i-1)+0.5\Phi(1-\Phi)M_{CCBbAa}(i-1)\Phi F_{CCBbAA}(i-1)+0.25\Phi(1-\Phi)M_{CCBbAa}(i-1)(1-$
$\Phi)F_{CCBBAa}(i-1)+0.5\Phi(1-\Phi)M_{CCBbAa}(i-1)\Phi F_{CCBBAa}(i-1)+0.5\Phi(1-\Phi)M_{CCBbAa}(i-1)F_{CCBBAA}(i-$
$1)+0.0625(1-\Phi)\Phi M_{CCBbAa}(i-1)(1-\Phi)(1-\Phi)F_{CcBbAa}(i-1)+0.125(1-\Phi)\Phi M_{CCBbAa}(i-1)\Phi(1-$
$\Phi)F_{CcBbAa}(i-1)+0.125(1-\Phi)\Phi M_{CCBbAa}(i-1)(1-\Phi)\Phi F_{CcBbAa}(i-1)+0.25(1-\Phi)\Phi M_{CCBbAa}(i-$
$1)\Phi\Phi F_{CcBbAa}(i-1)+0.125(1-\Phi)\Phi M_{CCBbAa}(i-1)(1-\Phi)F_{CcBbAA}(i-1)+0.25(1-\Phi)\Phi M_{CCBbAa}(i-$
$1)\Phi F_{CcBbAA}(i-1)+0.125(1-\Phi)\Phi M_{CCBbAa}(i-1)(1-\Phi)F_{CcBBAa}(i-1)+0.25(1-\Phi)\Phi M_{CCBbAa}(i-$
$1)\Phi F_{CcBBAa}(i-1)+0.25(1-\Phi)\Phi M_{CCBbAa}(i-1)F_{CcBBAA}(i-1)+0.125(1-\Phi)\Phi M_{CCBbAa}(i-1)(1-$
$\Phi)(1-\Phi)F_{CCBbAa}(i-1)+0.25(1-\Phi)\Phi M_{CCBbAa}(i-1)\Phi(1-\Phi)F_{CCBbAa}(i-1)+0.25(1-\Phi)\Phi M_{CCBbAa}(i-$
$1)(1-\Phi)\Phi F_{CCBbAa}(i-1)+0.5(1-\Phi)\Phi M_{CCBbAa}(i-1)\Phi\Phi F_{CCBbAa}(i-1)+0.25(1-\Phi)\Phi M_{CCBbAa}(i-$
$1)(1-\Phi)F_{CCBbAA}(i-1)+0.5(1-\Phi)\Phi M_{CCBbAa}(i-1)\Phi F_{CCBbAA}(i-1)+0.25(1-\Phi)\Phi M_{CCBbAa}(i-$
$1)(1-\Phi)F_{CCBBAa}(i-1)+0.5(1-\Phi)\Phi M_{CCBbAa}(i-1)\Phi F_{CCBBAa}(i-1)+0.5(1-\Phi)\Phi M_{CCBbAa}(i-$

$$1)F_{CCBBAA}(i-1)+0.125\Phi\Phi M_{CCBbAa}(i-1)(1-\Phi)(1-\Phi)F_{CcBbAa}(i-1)+0.25\Phi\Phi M_{CCBbAa}(i-1)\Phi(1-\Phi)F_{CcBbAa}(i-1)+0.25\Phi\Phi M_{CCBbAa}(i-1)(1-\Phi)\Phi F_{CcBbAa}(i-1)+0.5\Phi\Phi M_{CCBbAa}(i-1)\Phi\Phi F_{CcBbAa}(i-1)+0.25\Phi\Phi M_{CCBbAa}(i-1)(1-\Phi)F_{CcBbAA}(i-1)+0.5\Phi\Phi M_{CCBbAa}(i-1)\Phi F_{CcBbAA}(i-1)+0.25\Phi\Phi M_{CCBbAa}(i-1)(1-\Phi)F_{CcBBAa}(i-1)+0.5\Phi\Phi M_{CCBbAa}(i-1)\Phi F_{CcBBAa}(i-1)+0.5\Phi\Phi M_{CCBbAa}(i-1)F_{CcBBAA}(i-1)+0.25\Phi\Phi M_{CCBbAa}(i-1)(1-\Phi)(1-\Phi)F_{CCBbAa}(i-1)+0.5\Phi\Phi M_{CCBbAa}(i-1)\Phi(1-\Phi)F_{CCBbAa}(i-1)+0.5\Phi\Phi M_{CCBbAa}(i-1)(1-\Phi)\Phi F_{CCBbAa}(i-1)+\Phi\Phi M_{CCBbAa}(i-1)\Phi\Phi F_{CCBbAa}(i-1)+0.5\Phi\Phi M_{CCBbAa}(i-1)(1-\Phi)F_{CCBbAA}(i-1)+\Phi\Phi M_{CCBbAa}(i-1)\Phi F_{CCBbAA}(i-1)+0.5\Phi\Phi M_{CCBbAa}(i-1)(1-\Phi)F_{CCBBAa}(i-1)+\Phi\Phi M_{CCBbAa}(i-1)\Phi F_{CCBBAa}(i-1)+\Phi\Phi M_{CCBbAa}(i-1)F_{CCBBAA}(i-1)+0.0625(1-\Phi)M_{CCBbAA}(i-1)(1-\Phi)(1-\Phi)F_{CcBbAa}(i-1)+0.125(1-\Phi)M_{CCBbAA}(i-1)\Phi(1-\Phi)F_{CcBbAa}(i-1)+0.125(1-\Phi)M_{CCBbAA}(i-1)(1-\Phi)\Phi F_{CcBbAa}(i-1)+0.25(1-\Phi)M_{CCBbAA}(i-1)\Phi\Phi F_{CcBbAa}(i-1)+0.125(1-\Phi)M_{CCBbAA}(i-1)(1-\Phi)F_{CcBbAA}(i-1)+0.25(1-\Phi)M_{CCBbAA}(i-1)\Phi F_{CcBbAA}(i-1)+0.125(1-\Phi)M_{CCBbAA}(i-1)(1-\Phi)F_{CcBBAa}(i-1)+0.25(1-\Phi)M_{CCBbAA}(i-1)\Phi F_{CcBBAa}(i-1)+0.25(1-\Phi)M_{CCBbAA}(i-1)F_{CcBBAA}(i-1)+0.125(1-\Phi)M_{CCBbAA}(i-1)(1-\Phi)(1-\Phi)F_{CCBbAa}(i-1)+0.25(1-\Phi)M_{CCBbAA}(i-1)\Phi(1-\Phi)F_{CCBbAa}(i-1)+0.25(1-\Phi)M_{CCBbAA}(i-1)(1-\Phi)\Phi F_{CCBbAa}(i-1)+0.5(1-\Phi)M_{CCBbAA}(i-1)\Phi\Phi F_{CCBbAa}(i-1)+0.25(1-\Phi)M_{CCBbAA}(i-1)(1-\Phi)F_{CCBbAA}(i-1)+0.5(1-\Phi)M_{CCBbAA}(i-1)\Phi F_{CCBbAA}(i-1)+0.25(1-\Phi)M_{CCBbAA}(i-1)(1-\Phi)F_{CCBBAa}(i-1)+0.5(1-\Phi)M_{CCBbAA}(i-1)\Phi F_{CCBBAa}(i-1)+0.5(1-\Phi)M_{CCBbAA}(i-1)F_{CCBBAA}(i-1)+0.125\Phi M_{CCBbAA}(i-1)(1-\Phi)(1-\Phi)F_{CcBbAa}(i-1)+0.25\Phi M_{CCBbAA}(i-1)\Phi(1-\Phi)F_{CcBbAa}(i-1)+0.25\Phi M_{CCBbAA}(i-1)(1-\Phi)\Phi F_{CcBbAa}(i-1)+0.5\Phi M_{CCBbAA}(i-1)\Phi\Phi F_{CcBbAa}(i-1)+0.25\Phi M_{CCBbAA}(i-1)(1-\Phi)F_{CcBbAA}(i-1)+0.5\Phi M_{CCBbAA}(i-1)\Phi F_{CcBbAA}(i-1)+0.25\Phi M_{CCBbAA}(i-1)(1-\Phi)F_{CcBBAa}(i-1)+0.5\Phi M_{CCBbAA}(i-1)\Phi F_{CcBBAa}(i-1)+0.5\Phi M_{CCBbAA}(i-1)F_{CcBBAA}(i-1)+0.25\Phi M_{CCBbAA}(i-1)(1-\Phi)(1-\Phi)F_{CCBbAa}(i-1)+0.5\Phi M_{CCBbAA}(i-1)\Phi(1-\Phi)F_{CCBbAa}(i-1)+0.5\Phi M_{CCBbAA}(i-1)(1-\Phi)\Phi F_{CCBbAa}(i-1)+\Phi M_{CCBbAA}(i-1)\Phi\Phi F_{CCBbAa}(i-1)+0.5\Phi M_{CCBbAA}(i-1)(1-\Phi)F_{CCBbAA}(i-1)+\Phi M_{CCBbAA}(i-1)\Phi F_{CCBbAA}(i-1)+0.5\Phi M_{CCBbAA}(i-1)(1-\Phi)F_{CCBBAa}(i-1)+\Phi M_{CCBbAA}(i-1)\Phi F_{CCBBAa}(i-1)+\Phi M_{CCBbAA}(i-1)F_{CCBBAA}(i-1)+0.0625(1-\Phi)M_{CCBBAa}(i-1)(1-\Phi)(1-\Phi)F_{CcBbAa}(i-1)+0.125(1-\Phi)M_{CCBBAa}(i-1)\Phi(1-\Phi)F_{CcBbAa}(i-1)+0.125(1-\Phi)M_{CCBBAa}(i-1)(1-\Phi)\Phi F_{CcBbAa}(i-1)+0.25(1-\Phi)M_{CCBBAa}(i-1)\Phi\Phi F_{CcBbAa}(i-1)+0.125(1-\Phi)M_{CCBBAa}(i-1)(1-\Phi)F_{CcBbAA}(i-1)+0.25(1-\Phi)M_{CCBBAa}(i-1)\Phi F_{CcBbAA}(i-1)+0.125(1-\Phi)M_{CCBBAa}(i-1)(1-\Phi)F_{CcBBAa}(i-1)+0.25(1-\Phi)M_{CCBBAa}(i-1)\Phi F_{CcBBAa}(i-1)+0.25(1-\Phi)M_{CCBBAa}(i-1)F_{CcBBAA}(i-1)+0.125(1-\Phi)M_{CCBBAa}(i-1)(1-\Phi)(1-\Phi)F_{CCBbAa}(i-1)+0.25(1-\Phi)M_{CCBBAa}(i-1)\Phi(1-\Phi)F_{CCBbAa}(i-1)+0.25(1-\Phi)M_{CCBBAa}(i-1)(1-\Phi)\Phi F_{CCBbAa}(i-1)+0.5(1-\Phi)M_{CCBBAa}(i-1)\Phi\Phi F_{CCBbAa}(i-1)+0.25(1-\Phi)M_{CCBBAa}(i-1)(1-\Phi)F_{CCBbAA}(i-1)+0.5(1-\Phi)M_{CCBBAa}(i-1)\Phi F_{CCBbAA}(i-1)+0.25(1-\Phi)M_{CCBBAa}(i-1)(1-\Phi)F_{CCBBAa}(i-1)+0.5(1-\Phi)M_{CCBBAa}(i-1)\Phi F_{CCBBAa}(i-1)+0.5(1-\Phi)M_{CCBBAa}(i-1)F_{CCBBAA}(i-1)+0.125\Phi M_{CCBBAa}(i-1)(1-\Phi)(1-\Phi)F_{CcBbAa}(i-1)+0.25\Phi M_{CCBBAa}(i-1)\Phi(1-\Phi)F_{CcBbAa}(i-1)+0.25\Phi M_{CCBBAa}(i-1)(1-\Phi)\Phi F_{CcBbAa}(i-1)+0.5\Phi M_{CCBBAa}(i-1)\Phi\Phi F_{CcBbAa}(i-1)+0.25\Phi M_{CCBBAa}(i-1)(1-\Phi)F_{CcBbAA}(i-1)+0.5\Phi M_{CCBBAa}(i-1)\Phi F_{CcBbAA}(i-1)+0.25\Phi M_{CCBBAa}(i-1)(1-\Phi)F_{CcBBAa}(i-1)+0.5\Phi M_{CCBBAa}(i-1)\Phi F_{CcBBAa}(i-1)+0.5\Phi M_{CCBBAa}(i-1)F_{CcBBAA}(i-1)+0.25\Phi M_{CCBBAa}(i-1)(1-\Phi)(1-\Phi)F_{CCBbAa}(i-1)+0.5\Phi M_{CCBBAa}(i-1)\Phi(1-\Phi)F_{CCBbAa}(i-1)+0.5\Phi M_{CCBBAa}(i-1)(1-\Phi)\Phi F_{CCBbAa}(i-1)+\Phi M_{CCBBAa}(i-1)\Phi\Phi F_{CCBbAa}(i-1)+0.5\Phi M_{CCBBAa}(i-1)(1-\Phi)F_{CCBbAA}(i-1)+\Phi M_{CCBBAa}(i-1)\Phi F_{CCBbAA}(i-1)+0.5\Phi M_{CCBBAa}(i-1)(1-\Phi)F_{CCBBAa}(i-1)+\Phi M_{CCBBAa}(i-1)\Phi F_{CCBBAa}(i-1)+\Phi M_{CCBBAa}(i-1)F_{CCBBAA}(i-1)+0.125M_{CCBBAA}(i-1)(1-\Phi)(1-\Phi)F_{CcBbAa}(i-1)+0.25M_{CCBBAA}(i-1)\Phi(1-\Phi)F_{CcBbAa}(i-1)+0.25M_{CCBBAA}(i-1)(1-\Phi)\Phi F_{CcBbAa}(i-1)+0.5M_{CCBBAA}(i-1)\Phi\Phi F_{CcBbAa}(i-1)+0.25M_{CCBBAA}(i-1)(1-\Phi)F_{CcBbAA}(i-1)+0.5M_{CCBBAA}(i-1)\Phi F_{CcBbAA}(i-1)+0.25M_{CCBBAA}(i-1)(1-\Phi)F_{CcBBAa}(i-1)+0.5M_{CCBBAA}(i-1)\Phi F_{CcBBAa}(i-1)+0.5M_{CCBBAA}(i-1)F_{CcBBAA}(i-1)+0.25M_{CCBBAA}(i-1)(1-\Phi)(1-\Phi)F_{CCBbAa}(i-1)+0.5M_{CCBBAA}(i-1)\Phi(1-\Phi)F_{CCBbAa}(i-1)+0.5M_{CCBBAA}(i-1)(1-\Phi)\Phi F_{CCBbAa}(i-1)+M_{CCBBAA}(i-1)\Phi\Phi F_{CCBbAa}(i-1)+0.5M_{CCBBAA}(i-1)(1-\Phi)F_{CCBbAA}(i-1)+M_{CCBBAA}(i-1)\Phi F_{CCBbAA}(i-1)+0.5M_{CCBBAA}(i-1)(1-\Phi)F_{CCBBAa}(i-1)+M_{CCBBAA}(i-1)\Phi F_{CCBBAa}(i-1)+M_{CCBBAA}(i-1)F_{CCBBAA}(i-1)],$$

alongside an identical set of equations for females of each genotype.

The second stage is to normalise these proportional frequencies to fill the range from zero to one. We begin by calculating the overall fitness of the entire population by summing the proportional genotype

frequencies resulting from the above equations to give:

$$
\begin{aligned}
\bar{\Omega} = {} & M^e_{ccbbaa} + M^e_{ccbbAa} + M^e_{ccbbAA} + M^e_{ccBbaa} + M^e_{ccBbAa} + M^e_{ccBbAA} + M^e_{ccBBaa} + M^e_{ccBBAa} + M^e_{ccBBAA} \\
& + M^e_{Ccbbaa} + M^e_{CcbbAa} + M^e_{CcbbAA} + M^e_{CcBbaa} + M^e_{CcBbAa} + M^e_{CcBbAA} + M^e_{CcBBaa} + M^e_{CcBBAa} + M^e_{CcBBAA} \\
& + M^e_{CCbbaa} + M^e_{CCbbAa} + M^e_{CCbbAA} + M^e_{CCBbaa} + M^e_{CCBbAa} + M^e_{CCBbAA} + M^e_{CCBBaa} + M^e_{CCBBAa} + M^e_{ccBBAA} \\
& + F^e_{ccbbaa} + F^e_{ccbbAa} + F^e_{ccbbAA} + F^e_{ccBbaa} + F^e_{ccBbAa} + F^e_{ccBbAA} + F^e_{ccBBaa} + F^e_{ccBBAa} + F^e_{ccBBAA} \\
& + F^e_{Ccbbaa} + F^e_{CcbbAa} + F^e_{CcbbAA} + F^e_{CcBbaa} + F^e_{CcBbAa} + F^e_{CcBbAA} + F^e_{CcBBaa} + F^e_{CcBBAa} + F^e_{CcBBAA} \\
& + F^e_{CCbbaa} + F^e_{CCbbAa} + F^e_{CCbbAA} + F^e_{CCBbaa} + F^e_{CCBbAa} + F^e_{CCBbAA} + F^e_{CCBBaa} + F^e_{CCBBAa} + F^e_{ccBBAA}.
\end{aligned}
$$

This is then used as a normalising factor to give the final genotype frequencies for a particular generation as follows:

$$M_{ccbbaa}(i) = M^e_{ccbbaa}/\bar{\Omega}, \quad M_{ccbbAa}(i) = M^e_{ccbbAa}/\bar{\Omega}, \quad M_{ccbbAA}(i) = M^e_{ccbbAA}/\bar{\Omega},$$

$$M_{ccBbaa}(i) = M^e_{ccBbaa}/\bar{\Omega}, \quad M_{ccBbAa}(i) = M^e_{ccBbAa}/\bar{\Omega}, \quad M_{ccBbAA}(i) = M^e_{ccBbAA}/\bar{\Omega},$$

$$M_{ccBBaa}(i) = M^e_{ccBBaa}/\bar{\Omega}, \quad M_{ccBBAa}(i) = M^e_{ccBBAa}/\bar{\Omega}, \quad M_{ccBBAA}(i) = M^e_{ccBBAA}/\bar{\Omega},$$

$$M_{Ccbbaa}(i) = M^e_{Ccbbaa}/\bar{\Omega}, \quad M_{CcbbAa}(i) = M^e_{CcbbAa}/\bar{\Omega}, \quad M_{CcbbAA}(i) = M^e_{CcbbAA}/\bar{\Omega},$$

$$M_{CcBbaa}(i) = M^e_{CcBbaa}/\bar{\Omega}, \quad M_{CcBbAa}(i) = M^e_{CcBbAa}/\bar{\Omega}, \quad M_{CcBbAA}(i) = M^e_{CcBbAA}/\bar{\Omega},$$

$$M_{CcBBaa}(i) = M^e_{CcBBaa}/\bar{\Omega}, \quad M_{CcBBAa}(i) = M^e_{CcBBAa}/\bar{\Omega}, \quad M_{CcBBAA}(i) = M^e_{CcBBAA}/\bar{\Omega},$$

$$M_{CCbbaa}(i) = M^e_{CCbbaa}/\bar{\Omega}, \quad M_{CCbbAa}(i) = M^e_{CCbbAa}/\bar{\Omega}, \quad M_{CCbbAA}(i) = M^e_{CCbbAA}/\bar{\Omega},$$

$$M_{CCBbaa}(i) = M^e_{CCBbaa}/\bar{\Omega}, \quad M_{CCBbAa}(i) = M^e_{CCBbAa}/\bar{\Omega}, \quad M_{CCBbAA}(i) = M^e_{CCBbAA}/\bar{\Omega},$$

$$M_{CCBBaa}(i) = M^e_{CCBBaa}/\bar{\Omega}, \quad M_{CCBBAa}(i) = M^e_{CCBBAa}/\bar{\Omega}, \quad M_{CCBBAA}(i) = M^e_{CCBBAA}/\bar{\Omega},$$

$$F_{ccbbaa}(i) = F^e_{ccbbaa}/\bar{\Omega}, \quad F_{ccbbAa}(i) = F^e_{ccbbAa}/\bar{\Omega}, \quad F_{ccbbAA}(i) = F^e_{ccbbAA}/\bar{\Omega},$$

$$F_{ccBbaa}(i) = F^e_{ccBbaa}/\bar{\Omega}, \quad F_{ccBbAa}(i) = F^e_{ccBbAa}/\bar{\Omega}, \quad F_{ccBbAA}(i) = F^e_{ccBbAA}/\bar{\Omega},$$

$$F_{ccBBaa}(i) = F^e_{ccBBaa}/\bar{\Omega}, \quad F_{ccBBAa}(i) = F^e_{ccBBAa}/\bar{\Omega}, \quad F_{ccBBAA}(i) = F^e_{ccBBAA}/\bar{\Omega},$$

$$F_{Ccbbaa}(i) = F^e_{Ccbbaa}/\bar{\Omega}, \quad F_{CcbbAa}(i) = F^e_{CcbbAa}/\bar{\Omega}, \quad F_{CcbbAA}(i) = F^e_{CcbbAA}/\bar{\Omega},$$

$$F_{CcBbaa}(i) = F^e_{CcBbaa}/\bar{\Omega}, \quad F_{CcBbAa}(i) = F^e_{CcBbAa}/\bar{\Omega}, \quad F_{CcBbAA}(i) = F^e_{CcBbAA}/\bar{\Omega},$$

$$F_{CcBBaa}(i) = F^e_{CcBBaa}/\bar{\Omega}, \quad F_{CcBBAa}(i) = F^e_{CcBBAa}/\bar{\Omega}, \quad F_{CcBBAA}(i) = F^e_{CcBBAA}/\bar{\Omega},$$

$$F_{CCbbaa}(i) = F^e_{CCbbaa}/\bar{\Omega}, \quad F_{CCbbAa}(i) = F^e_{CCbbAa}/\bar{\Omega}, \quad F_{CCbbAA}(i) = F^e_{CCbbAA}/\bar{\Omega},$$

$$F_{CCBbaa}(i) = F^e_{CCBbaa}/\bar{\Omega}, \quad F_{CCBbAa}(i) = F^e_{CCBbAa}/\bar{\Omega}, \quad F_{CCBbAA}(i) = F^e_{CCBbAA}/\bar{\Omega},$$

$$F_{CCBBaa}(i) = F^e_{CCBBaa}/\bar{\Omega}, \quad F_{CCBBAa}(i) = F^e_{CCBBAa}/\bar{\Omega}, \quad F_{CCBBAA}(i) = F^e_{CCBBAA}/\bar{\Omega}.$$

We then insert these values into the proportional genotype frequency equations to calculate the values for the next generation and so on until the desired end point is reached.
