# Supplementary material for: Split drive killer-rescue provides a novel threshold-dependent gene drive
Source: Sci Rep. 2020 Nov 25;10:20520. doi: 10.1038/s41598-020-77544-7 (PMC7689494; doi:10.1038/s41598-020-77544-7)
Supplement: Supplementary file 5 — Supplementary material 5 [file 41598_2020_77544_MOESM5_ESM.pdf]

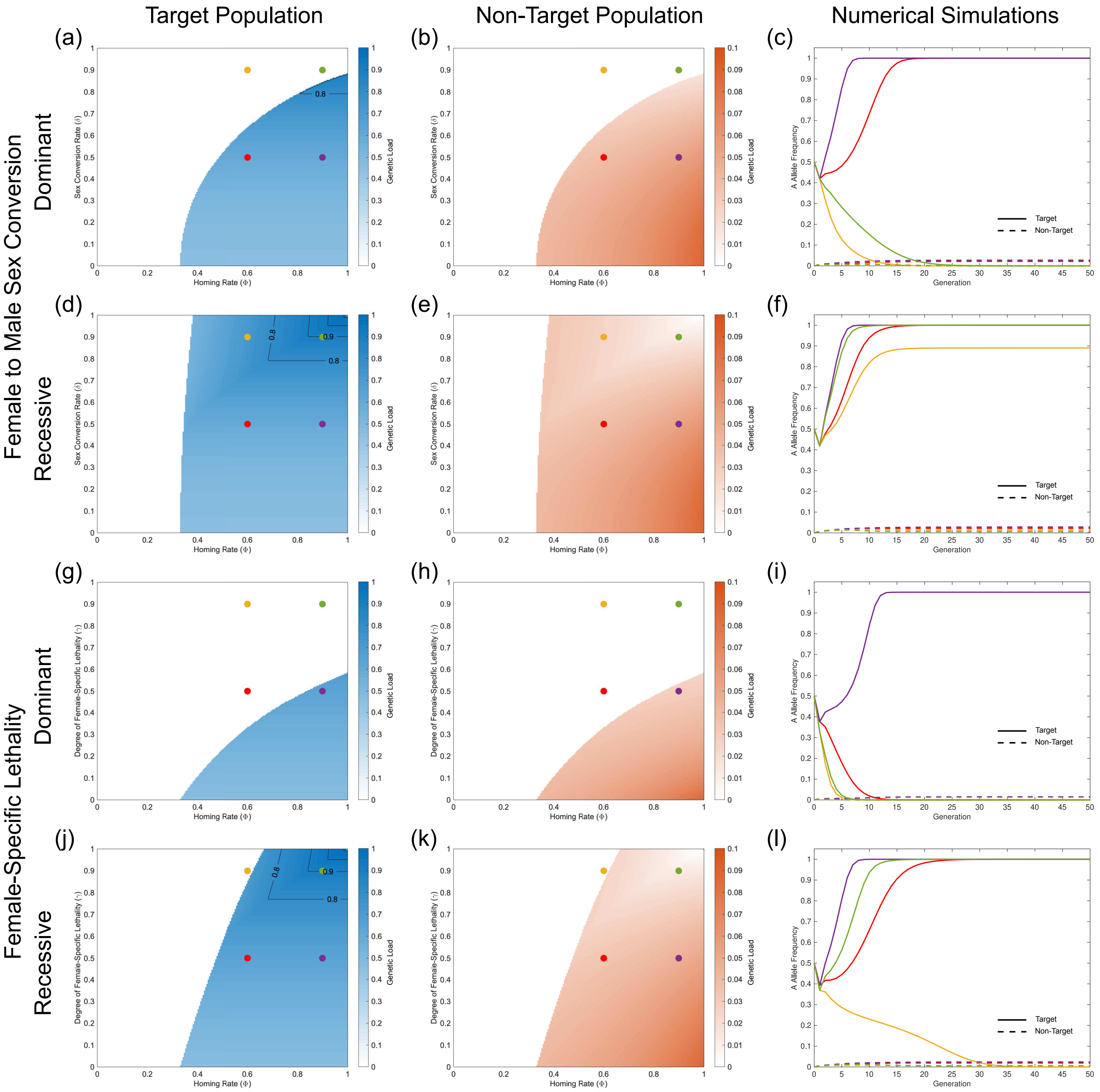

Figure S1: SDKR can, under certain conditions, achieve significant and localised population suppression. Results shown are for strategies based on dominant ((a)-(c) or recessive ((d)-(f)) female to male sex conversion and dominant ((g)-(i)) or recessive ((j)-(l)) female-specific lethality. Heat maps display the equilibrium genetic load imposed on a target (left column; blue) and a non-target population (centre column; orange). Here, the equilibrium state is assessed 1,000 generations after the release of transgenic individuals. The right column shows specific examples for parameter combinations indicated by coloured dots in the left and centre columns (i.e. combinations of homing rate ϕ=0.6 or 0.9 and female to male sex conversion (δ=0.5 or 0.9) or female-specific lethality (γ=0.5 or 0.9)). Solid lines represent transgene allele frequencies in a target population and dashed lines are the equivalent for a non-target population. In all cases, simulations consider a relative fitness of ε=0.85 per construct (applied multiplicatively) and fully penetrant lethal effects (i.e. L=1). Note the difference in scales for colour bars of target and non-target populations. In each case we consider the release of individuals homozygous for both transgenic constructs and fitness costs that are applied multiplicatively. Migration is considered to be unidirectional at a rate of 2% per generation. It is also worth noting that fitness costs associated with transgenic constructs can produce a small genetic load even where sex conversion and/or female-specific lethality are absent
